# Global burden of bacterial antimicrobial resistance 1990–2021: a systematic analysis with forecasts to 2050

**DOI:** 10.1016/S0140-6736(24)01867-1

**Published:** 2024-09-28

**Authors:** GBD 2021 Antimicrobial Resistance Collaborators, Mohsen Naghavi, Stein Emil Vollset, Kevin S Ikuta, Lucien R Swetschinski, Authia P Gray, Eve E Wool, Gisela Robles Aguilar, Tomislav Mestrovic, Georgia Smith, Chieh Han, Rebecca L Hsu, Julian Chalek, Daniel T Araki, Erin Chung, Catalina Raggi, Anna Gershberg Hayoon, Nicole Davis Weaver, Paulina A Lindstedt, Amanda E Smith, Umut Altay, Natalia V Bhattacharjee, Konstantinos Giannakis, Frederick Fell, Barney McManigal, Nattwut Ekapirat, Jessica Andretta Mendes, Tilleye Runghien, Oraya Srimokla, Atef Abdelkader, Sherief Abd-Elsalam, Richard Gyan Aboagye, Hassan Abolhassani, Hasan Abualruz, Usman Abubakar, Hana J Abukhadijah, Salahdein Aburuz, Ahmed Abu-Zaid, Sureerak Achalapong, Isaac Yeboah Addo, Victor Adekanmbi, Temitayo Esther Adeyeoluwa, Qorinah Estiningtyas Sakilah Adnani, Leticia Akua Adzigbli, Muhammad Sohail Afzal, Saira Afzal, Antonella Agodi, Austin J Ahlstrom, Aqeel Ahmad, Sajjad Ahmad, Tauseef Ahmad, Ali Ahmadi, Ayman Ahmed, Haroon Ahmed, Ibrar Ahmed, Mohammed Ahmed, Saeed Ahmed, Syed Anees Ahmed, Mohammed Ahmed Akkaif, Salah Al Awaidy, Yazan Al Thaher, Samer O Alalalmeh, Mohammad T AlBataineh, Wafa A Aldhaleei, Adel Ali Saeed Al-Gheethi, Nma Bida Alhaji, Abid Ali, Liaqat Ali, Syed Shujait Ali, Waad Ali, Kasim Allel, Sabah Al-Marwani, Ahmad Alrawashdeh, Awais Altaf, Alaa B. Al-Tammemi, Jaffar A Al-Tawfiq, Karem H Alzoubi, Walid Adnan Al-Zyoud, Ben Amos, John H Amuasi, Robert Ancuceanu, Jason R Andrews, Abhishek Anil, Iyadunni Adesola Anuoluwa, Saeid Anvari, Anayochukwu Edward Anyasodor, Geminn Louis Carace Apostol, Jalal Arabloo, Mosab Arafat, Aleksandr Y Aravkin, Demelash Areda, Abdulfatai Aremu, Anton A Artamonov, Elizabeth A Ashley, Marvellous O Asika, Seyyed Shamsadin Athari, Maha Moh'd Wahbi Atout, Tewachew Awoke, Sina Azadnajafabad, James Mba Azam, Shahkaar Aziz, Ahmed Y. Azzam, Mahsa Babaei, Francois-Xavier Babin, Muhammad Badar, Atif Amin Baig, Milica Bajcetic, Stephen Baker, Mainak Bardhan, Hiba Jawdat Barqawi, Zarrin Basharat, Afisu Basiru, Mathieu Bastard, Saurav Basu, Nebiyou Simegnew Bayleyegn, Melaku Ashagrie Belete, Olorunjuwon Omolaja Bello, Apostolos Beloukas, James A Berkley, Akshaya Srikanth Bhagavathula, Sonu Bhaskar, Soumitra S Bhuyan, Julia A Bielicki, Nikolay Ivanovich Briko, Colin Stewart Brown, Annie J Browne, Danilo Buonsenso, Yasser Bustanji, Cristina G Carvalheiro, Carlos A Castañeda-Orjuela, Muthia Cenderadewi, Joshua Chadwick, Sandip Chakraborty, Rama Mohan Chandika, Sara Chandy, Vilada Chansamouth, Vijay Kumar Chattu, Anis Ahmad Chaudhary, Patrick R Ching, Hitesh Chopra, Fazle Rabbi Chowdhury, Dinh-Toi Chu, Muhammad Chutiyami, Natalia Cruz-Martins, Alanna Gomes da Silva, Omid Dadras, Xiaochen Dai, Samuel D Darcho, Saswati Das, Fernando Pio De la Hoz, Denise Myriam Dekker, Kuldeep Dhama, Daniel Diaz, Benjamin Felix Rothschild Dickson, Serge Ghislain Djorie, Milad Dodangeh, Sushil Dohare, Klara Georgieva Dokova, Ojas Prakashbhai Doshi, Robert Kokou Dowou, Haneil Larson Dsouza, Susanna J Dunachie, Arkadiusz Marian Dziedzic, Tim Eckmanns, Abdelaziz Ed-Dra, Aziz Eftekharimehrabad, Temitope Cyrus Ekundayo, Iman El Sayed, Muhammed Elhadi, Waseem El-Huneidi, Christelle Elias, Sally J Ellis, Randa Elsheikh, Ibrahim Elsohaby, Chadi Eltaha, Babak Eshrati, Majid Eslami, David William Eyre, Adewale Oluwaseun Fadaka, Adeniyi Francis Fagbamigbe, Ayesha Fahim, Aliasghar Fakhri-Demeshghieh, Folorunso Oludayo Fasina, Modupe Margaret Fasina, Ali Fatehizadeh, Nicholas A Feasey, Alireza Feizkhah, Ginenus Fekadu, Florian Fischer, Ida Fitriana, Karen M Forrest, Celia Fortuna Rodrigues, John E Fuller, Muktar A Gadanya, Márió Gajdács, Aravind P Gandhi, Esteban E Garcia-Gallo, Denise O Garrett, Rupesh K Gautam, Miglas Welay Gebregergis, Mesfin Gebrehiwot, Teferi Gebru Gebremeskel, Christine Geffers, Leonidas Georgalis, Ramy Mohamed Ghazy, Mahaveer Golechha, Davide Golinelli, Melita Gordon, Snigdha Gulati, Rajat Das Gupta, Sapna Gupta, Vijai Kumar Gupta, Awoke Derbie Habteyohannes, Sebastian Haller, Harapan Harapan, Michelle L Harrison, Ahmed I Hasaballah, Ikramul Hasan, Rumina Syeda Hasan, Hamidreza Hasani, Andrea Haekyung Haselbeck, Md Saquib Hasnain, Ikrama Ibrahim Hassan, Shoaib Hassan, Mahgol Sadat Hassan Zadeh Tabatabaei, Khezar Hayat, Jiawei He, Omar E Hegazi, Mohammad Heidari, Kamal Hezam, Ramesh Holla, Marianne Holm, Heidi Hopkins, Md Mahbub Hossain, Mehdi Hosseinzadeh, Sorin Hostiuc, Nawfal R Hussein, Le Duc Huy, Elsa D Ibáñez-Prada, Adalia Ikiroma, Irena M Ilic, Sheikh Mohammed Shariful Islam, Faisal Ismail, Nahlah Elkudssiah Ismail, Chidozie Declan Iwu, Chinwe Juliana Iwu-Jaja, Abdollah Jafarzadeh, Fatoumatta Jaiteh, Reza Jalilzadeh Yengejeh, Roland Dominic G Jamora, Javad Javidnia, Talha Jawaid, Adam W J Jenney, Hyon Jin Jeon, Mohammad Jokar, Nabi Jomehzadeh, Tamas Joo, Nitin Joseph, Zul Kamal, Kehinde Kazeem Kanmodi, Rami S Kantar, James Apollo Kapisi, Ibraheem M Karaye, Yousef Saleh Khader, Himanshu Khajuria, Nauman Khalid, Faham Khamesipour, Ajmal Khan, Mohammad Jobair Khan, Muhammad Tariq Khan, Vishnu Khanal, Feriha Fatima Khidri, Jagdish Khubchandani, Suwimon Khusuwan, Min Seo Kim, Adnan Kisa, Vladimir Andreevich Korshunov, Fiorella Krapp, Ralf Krumkamp, Mohammed Kuddus, Mukhtar Kulimbet, Dewesh Kumar, Emmanuelle A P Kumaran, Ambily Kuttikkattu, Hmwe Hmwe Kyu, Iván Landires, Basira Kankia Lawal, Thao Thi Thu Le, Ingeborg Maria Lederer, Munjae Lee, Seung Won Lee, Alain Lepape, Temesgen Leka Lerango, Virendra S Ligade, Cherry Lim, Stephen S Lim, Liknaw Workie Limenh, Chaojie Liu, Xiaofeng Liu, Xuefeng Liu, Michael J Loftus, Hawraz Ibrahim M Amin, Kelsey Lynn Maass, Sandeep B Maharaj, Mansour Adam Mahmoud, Panagiota Maikanti-Charalampous, Omar M Makram, Kashish Malhotra, Ahmad Azam Malik, Georgia D Mandilara, Florian Marks, Bernardo Alfonso Martinez-Guerra, Miquel Martorell, Hossein Masoumi-Asl, Alexander G Mathioudakis, Juergen May, Theresa A McHugh, James Meiring, Hadush Negash Meles, Addisu Melese, Endalkachew Belayneh Melese, Giuseppe Minervini, Nouh Saad Mohamed, Shafiu Mohammed, Syam Mohan, Ali H Mokdad, Lorenzo Monasta, AmirAli Moodi Ghalibaf, Catrin E Moore, Yousef Moradi, Elias Mossialos, Vincent Mougin, George Duke Mukoro, Francesk Mulita, Berit Muller-Pebody, Efren Murillo-Zamora, Sani Musa, Patrick Musicha, Lillian A Musila, Saravanan Muthupandian, Ahamarshan Jayaraman Nagarajan, Pirouz Naghavi, Firzan Nainu, Tapas Sadasivan Nair, Hastyar Hama Rashid Najmuldeen, Zuhair S Natto, Javaid Nauman, Biswa Prakash Nayak, G Takop Nchanji, Pacifique Ndishimye, Ionut Negoi, Ruxandra Irina Negoi, Seyed Aria Nejadghaderi, QuynhAnh P Nguyen, Efaq Ali Noman, Davis C Nwakanma, Seamus O'Brien, Theresa J Ochoa, Ismail A Odetokun, Oluwaseun Adeolu Ogundijo, Tolulope R Ojo-Akosile, Sylvester Reuben Okeke, Osaretin Christabel Okonji, Andrew T Olagunju, Antonio Olivas-Martinez, Abdulhakeem Abayomi Olorukooba, Peter Olwoch, Kenneth Ikenna Onyedibe, Edgar Ortiz-Brizuela, Olayinka Osuolale, Pradthana Ounchanum, Oyetunde T Oyeyemi, Mahesh Padukudru P A, Jose L Paredes, Romil R Parikh, Jay Patel, Shankargouda Patil, Shrikant Pawar, Anton Y Peleg, Prince Peprah, João Perdigão, Carlo Perrone, Ionela-Roxana Petcu, Koukeo Phommasone, Zahra Zahid Piracha, Dimitri Poddighe, Andrew J Pollard, Ramesh Poluru, Alfredo Ponce-De-Leon, Jagadeesh Puvvula, Farah Naz Qamar, Nameer Hashim Qasim, Clotaire Donatien Rafai, Pankaja Raghav, Leila Rahbarnia, Fakher Rahim, Vafa Rahimi-Movaghar, Mosiur Rahman, Muhammad Aziz Rahman, Hazem Ramadan, Shakthi Kumaran Ramasamy, Pushkal Sinduvadi Ramesh, Pramod W Ramteke, Rishabh Kumar Rana, Usha Rani, Mohammad-Mahdi Rashidi, Devarajan Rathish, Sayaphet Rattanavong, Salman Rawaf, Elrashdy Moustafa Mohamed Redwan, Luis Felipe Reyes, Tamalee Roberts, Julie V Robotham, Victor Daniel Rosenthal, Allen Guy Ross, Nitai Roy, Kristina E Rudd, Cameron John Sabet, Basema Ahmad Saddik, Mohammad Reza Saeb, Umar Saeed, Sahar Saeedi Moghaddam, Weeravoot Saengchan, Mohsen Safaei, Amene Saghazadeh, Narjes Saheb Sharif-Askari, Amirhossein Sahebkar, Soumya Swaroop Sahoo, Maitreyi Sahu, Morteza Saki, Nasir Salam, Zikria Saleem, Mohamed A Saleh, Yoseph Leonardo Samodra, Abdallah M Samy, Aswini Saravanan, Maheswar Satpathy, Austin E Schumacher, Mansour Sedighi, Samroeng Seekaew, Mahan Shafie, Pritik A Shah, Samiah Shahid, Moyad Jamal Shahwan, Sadia Shakoor, Noga Shalev, Muhammad Aaqib Shamim, Mohammad Ali Shamshirgaran, Anas Shamsi, Amin Sharifan, Rajesh P Shastry, Mahabalesh Shetty, Aminu Shittu, Sunil Shrestha, Emmanuel Edwar Siddig, Theologia Sideroglou, Jose Sifuentes-Osornio, Luís Manuel Lopes Rodrigues Silva, Eric A F Simões, Andrew J H Simpson, Amit Singh, Surjit Singh, Robert Sinto, Sameh S M Soliman, Soroush Soraneh, Nicole Stoesser, Temenuga Zhekova Stoeva, Chandan Kumar Swain, Lukasz Szarpak, Sree Sudha T Y, Shima Tabatabai, Celine Tabche, Zanan Mohammed-Ameen Taha, Ker-Kan Tan, Nidanuch Tasak, Nathan Y Tat, Areerat Thaiprakong, Pugazhenthan Thangaraju, Caroline Chepngeno Tigoi, Krishna Tiwari, Marcos Roberto Tovani-Palone, Thang Huu Tran, Munkhtuya Tumurkhuu, Paul Turner, Aniefiok John Udoakang, Arit Udoh, Noor Ullah, Saeed Ullah, Asokan Govindaraj Vaithinathan, Mario Valenti, Theo Vos, Huong T L Vu, Yasir Waheed, Ann Sarah Walker, Judd L Walson, Tri Wangrangsimakul, Kosala Gayan Weerakoon, Heiman F L Wertheim, Phoebe C M Williams, Asrat Arja Wolde, Teresa M Wozniak, Felicia Wu, Zenghong Wu, Mukesh Kumar Kumar Yadav, Sajad Yaghoubi, Zwanden Sule Yahaya, Amir Yarahmadi, Saber Yezli, Yazachew Engida Yismaw, Dong Keon Yon, Chun-Wei Yuan, Hadiza Yusuf, Fathiah Zakham, Giulia Zamagni, Haijun Zhang, Zhi-Jiang Zhang, Magdalena Zielińska, Alimuddin Zumla, Sa'ed H. H Zyoud, Samer H Zyoud, Simon I Hay, Andy Stergachis, Benn Sartorius, Ben S Cooper, Christiane Dolecek, Christopher J L Murray

## Abstract

**Background:**

Antimicrobial resistance (AMR) poses an important global health challenge in the 21st century. A previous study has quantified the global and regional burden of AMR for 2019, followed with additional publications that provided more detailed estimates for several WHO regions by country. To date, there have been no studies that produce comprehensive estimates of AMR burden across locations that encompass historical trends and future forecasts.

**Methods:**

We estimated all-age and age-specific deaths and disability-adjusted life-years (DALYs) attributable to and associated with bacterial AMR for 22 pathogens, 84 pathogen–drug combinations, and 11 infectious syndromes in 204 countries and territories from 1990 to 2021. We collected and used multiple cause of death data, hospital discharge data, microbiology data, literature studies, single drug resistance profiles, pharmaceutical sales, antibiotic use surveys, mortality surveillance, linkage data, outpatient and inpatient insurance claims data, and previously published data, covering 520 million individual records or isolates and 19 513 study-location-years. We used statistical modelling to produce estimates of AMR burden for all locations, including those with no data. Our approach leverages the estimation of five broad component quantities: the number of deaths involving sepsis; the proportion of infectious deaths attributable to a given infectious syndrome; the proportion of infectious syndrome deaths attributable to a given pathogen; the percentage of a given pathogen resistant to an antibiotic of interest; and the excess risk of death or duration of an infection associated with this resistance. Using these components, we estimated disease burden attributable to and associated with AMR, which we define based on two counterfactuals; respectively, an alternative scenario in which all drug-resistant infections are replaced by drug-susceptible infections, and an alternative scenario in which all drug-resistant infections were replaced by no infection. Additionally, we produced global and regional forecasts of AMR burden until 2050 for three scenarios: a reference scenario that is a probabilistic forecast of the most likely future; a Gram-negative drug scenario that assumes future drug development that targets Gram-negative pathogens; and a better care scenario that assumes future improvements in health-care quality and access to appropriate antimicrobials. We present final estimates aggregated to the global, super-regional, and regional level.

**Findings:**

In 2021, we estimated 4·71 million (95% UI 4·23–5·19) deaths were associated with bacterial AMR, including 1·14 million (1·00–1·28) deaths attributable to bacterial AMR. Trends in AMR mortality over the past 31 years varied substantially by age and location. From 1990 to 2021, deaths from AMR decreased by more than 50% among children younger than 5 years yet increased by over 80% for adults 70 years and older. AMR mortality decreased for children younger than 5 years in all super-regions, whereas AMR mortality in people 5 years and older increased in all super-regions. For both deaths associated with and deaths attributable to AMR, meticillin-resistant *Staphylococcus aureus* increased the most globally (from 261 000 associated deaths [95% UI 150 000–372 000] and 57 200 attributable deaths [34 100–80 300] in 1990, to 550 000 associated deaths [500 000–600 000] and 130 000 attributable deaths [113 000–146 000] in 2021). Among Gram-negative bacteria, resistance to carbapenems increased more than any other antibiotic class, rising from 619 000 associated deaths (405 000–834 000) in 1990, to 1·03 million associated deaths (909 000–1·16 million) in 2021, and from 127 000 attributable deaths (82 100–171 000) in 1990, to 216 000 (168 000–264 000) attributable deaths in 2021. There was a notable decrease in non-COVID-related infectious disease in 2020 and 2021. Our forecasts show that an estimated 1·91 million (1·56–2·26) deaths attributable to AMR and 8·22 million (6·85–9·65) deaths associated with AMR could occur globally in 2050. Super-regions with the highest all-age AMR mortality rate in 2050 are forecasted to be south Asia and Latin America and the Caribbean. Increases in deaths attributable to AMR will be largest among those 70 years and older (65·9% [61·2–69·8] of all-age deaths attributable to AMR in 2050). In stark contrast to the strong increase in number of deaths due to AMR of 69·6% (51·5–89·2) from 2022 to 2050, the number of DALYs showed a much smaller increase of 9·4% (–6·9 to 29·0) to 46·5 million (37·7 to 57·3) in 2050. Under the better care scenario, across all age groups, 92·0 million deaths (82·8–102·0) could be cumulatively averted between 2025 and 2050, through better care of severe infections and improved access to antibiotics, and under the Gram-negative drug scenario, 11·1 million AMR deaths (9·08–13·2) could be averted through the development of a Gram-negative drug pipeline to prevent AMR deaths.

**Interpretation:**

This study presents the first comprehensive assessment of the global burden of AMR from 1990 to 2021, with results forecasted until 2050. Evaluating changing trends in AMR mortality across time and location is necessary to understand how this important global health threat is developing and prepares us to make informed decisions regarding interventions. Our findings show the importance of infection prevention, as shown by the reduction of AMR deaths in those younger than 5 years. Simultaneously, our results underscore the concerning trend of AMR burden among those older than 70 years, alongside a rapidly ageing global community. The opposing trends in the burden of AMR deaths between younger and older individuals explains the moderate future increase in global number of DALYs versus number of deaths. Given the high variability of AMR burden by location and age, it is important that interventions combine infection prevention, vaccination, minimisation of inappropriate antibiotic use in farming and humans, and research into new antibiotics to mitigate the number of AMR deaths that are forecasted for 2050.

**Funding:**

UK Department of Health and Social Care's Fleming Fund using UK aid, and the Wellcome Trust.

## Introduction

In 2014, the Review on Antimicrobial Resistance (AMR) projected that 10 million deaths caused by AMR could occur by 2050.^1^ This estimate, while the subject of scientific criticism,^2^ helped position AMR as one of the most pressing threats to health of the 21st century.^3^ In the years since, WHO committed to the 2015 AMR global action plan, AMR was the focus of a high-level UN general assembly in 2016, and an AMR-specific indicator was included as a Sustainable Development Goal: to reduce the percentage of bloodstream infection due to selected antimicrobial-resistant organisms (indicator 3.d.2). Despite the attention AMR has received at the global level, the implementation and funding of national action plans^4^ has been uneven, leading to uncertain progress in attenuating the burden of AMR.^1^

The Review on Antimicrobial Resistance^1^ acknowledged its broad brush estimates would be strengthened by more robust work from academic researchers.^5^ In the decade since its publication, however, global AMR estimates have been sparse. One recent step forward in AMR epidemiology was a global estimate of the burden of bacterial AMR in 2019,^6^ which found that of the roughly 8·9 million deaths due to bacterial infections that year, 1·27 million deaths were attributable to AMR and 4·95 million deaths were associated with AMR.^6^, ^7^ While this estimate has been used to inform research and policy priorities, such as the 2024 WHO Bacterial Priority Pathogens List,^8^ it provides only a cross-section of burden, limiting the ability to understand the historical trend of AMR and evaluate policies implemented to reduce its impact. So far, time trends in AMR have been limited to select high-income locations for a small number of pathogen–drug combinations. These include estimates from the US Centers for Disease Control and Prevention, which evaluated 18 AMR threats in 2013 and 2019, and from Cassini and colleagues,^9^ who produced AMR estimates for the European Union and European Economic Area in 2007 and 2015 for 16 pathogen–drug combinations. Recent estimates indicate that the highest burdens attributable to, and associated with, AMR are in sub-Saharan Africa and south Asia;^6^ however, these are locations without any historical estimates. Identifying the regions with increasing AMR burden would enable the global health community to better target locations with the most urgent need. Documenting the evolving nature of AMR, including the trends in resistance to critical antibiotic classes, such as the carbapenems, can guide antimicrobial stewardship, infection prevention measures, and prioritisation of drug and vaccine development to combat this growing threat.

A recent *Lancet* series on AMR highlighted current paediatric vaccines, investment in water, sanitation, and hygiene (WASH) infrastructure, and infection control practices as strategies to curtail AMR-related deaths in low-income and middle-income countries (LMICs). Previously published global estimates reported a 53% decline in age-standardised sepsis-related deaths from 1990 to 2017.^10^ Much of this was driven by reductions in child mortality^11^ and the prevention of deaths due to lower respiratory infection^12^ and diarrhoea,^13^ causes of death that are often avertable with access to WASH and vaccines.^14^ While this decrease in sepsis mortality might be expected to reduce AMR-related deaths due to sepsis, research suggests demographic shifts over the next century will produce an ageing population,^15^ leading to increasing prevalence of comorbidities and immunosenescence, resulting in a population at greater risk of sepsis related deaths and AMR mortality. Furthermore, the continued overuse of antimicrobials, both in human health and in agriculture, produces an environment that selects for increasingly resistant bacteria.^16^ It is currently unknown how the convergence of these factors might affect future AMR burden because no recent global AMR burden forecasts have been produced. Characterising these trends and producing forecasts of estimates into the future, with alternative scenarios based on policy-related interventions, are needed to better understand the nature of AMR, and to inform research and public health priorities for decades to come.

In this study, we present the first global and regional estimates of AMR mortality over the past three decades, from 1990 to 2021, for 22 pathogens and 16 antimicrobials using two counterfactual scenarios: the counterfactuals of no infection (ie, deaths or disability-adjusted life-years [DALYs] associated with a drug-resistant infection) and drug-sensitive infection (ie, deaths or DALYs directly attributable to the drug resistance of an infection). The Global Burden of Diseases, Injuries, and Risk Factors Study (GBD) forecasting framework allowed us to produce forecasts of AMR burden and impact of alternative scenarios on disease burden.^17^ We present global and regional forecasts of AMR burden to 2050 for a reference scenario (the most likely future), a scenario assuming future drug development targeting Gram-negative pathogens (Gram-negative drug scenario), and a scenario assuming future improvements in health-care quality for sepsis and access to appropriate antimicrobials (better care scenario). This Article was produced as part of the GBD Collaborator Network and in accordance with the GBD protocol.^18^

**Research in context**

**Evidence before this study**

In 2014, the review on antimicrobial resistance (AMR) was established to assess the global issue of rising drug resistance, understand its magnitude, and define steps to address it. The AMR review published an influential analysis in 2016, forecasting that 10 million people could die annually from AMR by 2050. Since then, estimates of the burden of drug-resistant infections covering several pathogens have been published for the USA, Thailand, the EU, and the European Economic Area. No studies, however, provided comprehensive estimates covering all locations and a broad range of pathogen–drug combinations until the global burden of AMR in 2019 study was published. The study estimated that 4·95 million deaths associated with bacterial AMR and 1·27 million deaths attributable to bacterial AMR occurred in 2019. This research was followed by additional studies that detailed more granular country estimates published for the WHO regions of Europe, the Americas, and Africa, showing that deaths from AMR are unevenly distributed among populations. Each regional study, and the larger global study, have provided an essential snapshot of the magnitude of AMR burden for 2019. A 2024 *Lancet* Series on antimicrobial resistance used findings from some of these studies and others to summarise the current state of antibiotic resistance and recommended several global targets to combat the rise of AMR, including a target for 10% reduction in AMR mortality from the 2019 baseline by 2030.

**Added value of this study**

Ascertaining the time trends of AMR burden is of considerable importance to improve our understanding of its current and future threat. Multidecade temporal trends allow us to observe how patterns of AMR mortality are changing and for which populations it may be worsening. Such temporal analyses are essential for monitoring progress towards targeted reductions in AMR burden, for informing prioritisation of AMR interventions, and for assessing the long-term effectiveness of national action plans for tackling AMR. Existing time trends of AMR are limited to select high-income countries and do not include a comprehensive set of pathogen–drug combinations. We provide estimates of deaths and disability-adjusted life-years (DALYs) for 22 pathogens and 11 infectious syndromes for 204 countries and territories from 1990 to 2021. We produced estimates of deaths and DALYs attributable to AMR and associated with AMR. The attributable burden is defined using a counterfactual in which all drug-resistant infections are replaced by equivalent drug-susceptible infections. The associated burden is defined using a counterfactual in which all drug-resistant infections are replaced by no infection. Additionally, using the Global Burden of Disease Study (GBD) 2021 forecasting framework, we produced reference forecasts (the most likely future) for burden attributable to or associated with AMR, and the wider impact of alternative scenarios on disease burden to 2050. This study represents our most robust estimates of AMR burden to date, including updates to our input data, estimates of AMR by time and more granular age groups, and improvements to our statistical models. We build our analysis on estimates of disease, incidence, prevalence, and mortality from GBD, allowing for comparability of deaths from AMR to other leading causes of death and disability throughout the world.

**Implications of all the available evidence**

Our analysis greatly expands the evidence base for time trends of AMR mortality. We show that global mortality from AMR increased minimally between 1990 and 2019, followed by a small decline that occurred during the COVID-19 pandemic. Our reference scenario indicates that deaths from AMR will increase by 2050 if remediation measures are not in place. Our analysis of trends in AMR mortality by age suggests that there is a need for interventions to tackle the increasing burden of AMR in older age groups going forward. Findings from this study provide evidentiary support to policy measures that combat AMR and have the potential to save lives, by adopting strategies that decrease risk of infections through new vaccines, improved quality of health care in hospitals and health centres, improved access to antibiotics and promotion of antibiotic stewardship. The development of new antimicrobials for Gram-negative bacteria should be prioritised, given the large increase in carbapenem-resistance highlighted in this study. New prevention efforts to address AMR must remain a priority for global health policy makers. Our time trend analyses and the methodology used lay the foundation for additional studies to continue forecasting future AMR trends and track the progress of implemented measures in our ongoing efforts to mitigate this important global health challenge. Our forecasts of alternative scenarios show the potential to avert large numbers of deaths over the next quarter century.

## Methods

### Overview

To estimate AMR burden, we used the same broad methodological approach as the global burden of AMR in 2019 study. The detailed methods of that study are published elsewhere^6^ and are summarised here, along with important methodological improvements.

We developed an approach for estimating the burden of AMR that builds on death and select incidence estimates for different underlying conditions from GBD 2021 (appendix 1 pp 16–74, 80).^19^ First, we collected data from multiple sources, ensuring that we included information available by year, age, and location across our ten estimation steps wherever possible. We estimated the AMR burden associated with and attributable to 11 of the 22 modelled infectious syndromes (bloodstream infections; meningitis; lower respiratory infections; endocarditis; peritoneal and intra-abdominal infections; diarrhoea; urinary tract infections and pyelonephritis; infections of bones, joints, and related organs; infections of the skin and subcutaneous systems; tuberculosis; and typhoid, paratyphoid, and invasive non-typhoidal *Salmonella*), 22 bacterial pathogens, 16 drug categories or combinations of drugs for which there is resistance, and 84 pathogen–drug combinations (appendix 1 pp 51–52). We modelled all-age and age-specific deaths and DALYs for 204 countries and territories, and we present aggregated estimates globally and for each of the seven GBD super-regions and 21 GBD regions.^20^ For a complete list of GBD locations by region see appendix 2 (pp 18–35).

### Input data

We sought the widest possible range of data using several data collection strategies. Through our large collaborator networks and through standard data seeking as part of the GBD, we obtained datasets not previously available for AMR research, including multiple cause of death data, hospital discharge, microbiology data with and without patient outcome, studies published in scientific journals, reports from networks that monitor bacteria resistant to antibiotics, pharmaceutical sales, antibiotic use surveys, mortality surveillance, linkage data, outpatient and inpatient linked insurance claims data, and publicly available data.^21^ Each component of our estimation processes had distinct data requirements and the input data used for each step thus differed (table 1). The diverse data sought included the following sources: (1) pharmaceutical companies that run surveillance networks, diagnostic laboratories, and clinical trials; (2) researchers including large, multisite research collaborations; smaller studies; and well established research institutes based in LMICs; (3) public and private hospitals and public health institutes doing diagnostic testing; and (4) global surveillance networks; enhanced surveillance systems; national surveillance systems; and surveillance systems for specific organisms such as *Mycobacterium tuberculosis* and *Neisseria gonorrhoeae*. All sources are listed by data type in appendix 1 (pp 7–16).

**Table 1:** Data inputs by source type, 1990–2009 and 2009–2021, sample size, and study location years

	**1990–2009**		**2010–2021**		**Sample size units**
	**Number of study-GBD location-years**	**Sample size**	**Number of study-GBD location-years**	**Sample size**	
Multiple cause of death	1996	71 766 533	1503	66 804 957	Deaths
Hospital discharge	508	5 052 412	805	4 999 065	Discharges
Outpatient and Inpatient insurance claims	0	0	8	20 991 554	Visits and admissions
Microbial or laboratory data with outcome	359	1 325 029	995	17 646 602	Isolates
Microbial or laboratory data without outcome	1038	13 681 797	3009	97 863 850	Isolates
Literature studies	3321	4 400 616	1545	3 010 191	Cases or isolates
Single drug resistance profiles	816	49 563 600	1482	161 875 741	Isolates
Pharmaceutical sales	883	883	829	829	Study-country-years
Antibiotic use among children younger than 5 years who reported illness	134	57 171	207	200 498	Households surveyed
Mortality surveillance (minimally invasive tissue sampling from Child Health and Mortality Prevention Surveillance)	0	0	37	2460	Isolates
Linkage (mortality only)	27	204 736	11	83 579	Deaths
Cause of death input data	5130	469 412 517	1436	232 349 574	Deaths

Table 1 shows a summary of the distinct data types gathered and for which model component each data type was used. Also shown is the number of unique study-location-years and individual records or isolates available for each data type. Location-years of data refer to unique GBD locations and years for which we have records or isolates. In total, there were 520 million individual records or isolates covering 19 513 study-location-years used as input data to the AMR estimation process. An additional 702 million deaths informed the underlying cause-specific mortality envelope from the GBD that we leveraged when calculating our sepsis mortality envelope. Table 2 shows the number of individual records or isolates used and the number of countries covered in each primary modelling step separately by GBD region. Three of five component models include data from every GBD region, and two of five include data from 13 and 20 of the 21 GBD regions. Our models of sepsis and infectious syndrome fractions of underlying causes are the most geographically sparse, covering 23 countries from 13 regions; the results of which are then applied to the GBD cause-specific mortality envelope informed by data covering 195 countries from all 21 regions.

**Table 2:** Data (cases or deaths) included in each primary modelling step by region and the fraction of countries represented in each region

	**Years**	GBD cause of death database (step 0)*		**Sepsis and infectious syndrome models (step 1)**		**Case fatality rate (step 2)**		**Pathogen distribution (step 3)**		**Prevalence of resistance (step 4)**		**Relative risk (step 5)**	
		**Number of deaths**	**Fraction of countries represented**	**Number of deaths**	**Fraction of countries represented**	**Number of cases**	**Fraction of countries represented**	**Number of cases**	**Fraction of countries represented**	**Number of cases**	**Fraction of countries represented**	**Number of cases**	**Fraction of countries represented**
Andean Latin America	1990–2009	3 820 911	3 of 3	..	..	3480	2 of 3	63 243	3 of 3	121 066	3 of 3	65 964	3 of 3
	2010–2021	1 465 590	3 of 3	..	..	80	1 of 3	2365	2 of 3	227 755	3 of 3	26 900	3 of 3
Australasia	1990–2009	4 528 250	2 of 2	149 697	1 of 2	80 275	1 of 2	25 998	2 of 2	2 164 774	2 of 2	150 952	2 of 2
	2010–2021	1 622 390	2 of 2	167 840	1 of 2	277 427	2 of 2	398 308	2 of 2	3 396 867	2 of 2	592 851	2 of 2
Caribbean	1990–2009	4 558 782	19 of 19	..	..	19 251	2 of 19	62 456	5 of 19	173 074	5 of 19	133 113	5 of 19
	2010–2021	1 418 887	19 of 19	..	..	8432	3 of 19	6102	4 of 19	60 279	6 of 19	30 272	5 of 19
Central Asia	1990–2009	11 961 820	9 of 9	66 771	3 of 9	4887	1 of 9	81 972	6 of 9	101 408	3 of 9	59 934	3 of 9
	2010–2021	3 794 666	9 of 9	..	..	..	..	2234	2 of 9	5949	5 of 9	4596	2 of 9
Central Europe	1990–2009	33 212 410	13 of 13	..	..	1 171 088	10 of 13	3 797 792	10 of 13	3 120 201	12 of 13	3 628 021	12 of 13
	2010–2021	11 429 450	13 of 13	..	..	275 296	10 of 13	114 245	10 of 13	880 145	10 of 13	1 066 039	8 of 13
Central Latin America	1990–2009	23 751 460	9 of 9	2 987 071	2 of 9	525 174	9 of 9	76 286	8 of 9	1 046 062	9 of 9	466 049	9 of 9
	2010–2021	11 011 570	9 of 9	11 787 179	2 of 9	1 453 094	8 of 9	1 467 114	9 of 9	22 822 424	9 of 9	16 158 315	9 of 9
Central sub-Saharan Africa	1990–2009	4120	5 of 6	..	..	..	..	430	4 of 6	28 331	5 of 6	24 855	4 of 6
	2010–2021	1213	5 of 6	..	..	..	..	..	..	1734	3 of 6	139	2 of 6
East Asia	1990–2009	13 921 440	3 of 3	249 992	1 of 3	154 863	2 of 3	66 516	2 of 3	1 501 542	2 of 3	860 165	2 of 3
	2010–2021	53 581 700	3 of 3	1 079 267	1 of 3	907 562	2 of 3	530 432	2 of 3	2 152 800	2 of 3	1 738 085	2 of 3
Eastern Europe	1990–2009	71 077 680	7 of 7	..	..	470 286	5 of 7	203 816	6 of 7	669 146	7 of 7	876 947	7 of 7
	2010–2021	25 203 530	7 of 7	..	..	33 732	3 of 7	18 594	5 of 7	157 873	5 of 7	168 981	4 of 7
Eastern sub-Saharan Africa	1990–2009	228 636	14 of 15	..	..	1 003	1 of 15	7610	10 of 15	133 681	11 of 15	88 268	8 of 15
	2010–2021	27 948	14 of 15	625	3 of 15	19 466	4 of 15	43 724	11 of 15	354 548	13 of 15	273 361	12 of 15
High-income Asia Pacific	1990–2009	33 180 280	4 of 4	..	..	417 693	3 of 4	194 837	3 of 4	121 845 396	4 of 4	121 983 482	4 of 4
	2010–2021	15 927 180	4 of 4	..	..	97 050	2 of 4	20 951	3 of 4	1 325 127	4 of 4	1 343 243	4 of 4
High-income North America	1990–2009	73 876 250	3 of 3	56 181 023	1 of 3	1 808 900	2 of 3	3 296 180	2 of 3	1 326 754	2 of 3	1 288 451	2 of 3
	2010–2021	29 459 200	3 of 3	28 502 039	1 of 3	29 965 525	2 of 3	3 894 238	2 of 3	51 880 035	2 of 3	38 539 427	2 of 3
North Africa and Middle East	1990–2009	4 450 410	21 of 21	62 401	2 of 21	700 309	13 of 21	490 458	19 of 21	2 962 227	21 of 21	3 003 646	21 of 21
	2010–2021	8 233 307	21 of 21	..	..	55 919	7 of 21	13 807	18 of 21	117 890	18 of 21	93 792	16 of 21
Oceania	1990–2009	82 070	11 of 18	..	..	..	..	102	2 of 18	664	2 of 18	599	2 of 18
	2010–2021	30 422	11 of 18	..	..	2212	1 of 18	255	2 of 18	5092	2 of 18	3237	1 of 18
South Asia	1990–2009	14 574 240	5 of 5	22 834	3 of 5	276 726	4 of 5	360 479	4 of 5	4 972 848	5 of 5	4 541 180	5 of 5
	2010–2021	2 238 041	5 of 5	..	..	52 332	2 of 5	113 838	4 of 5	1 122 319	4 of 5	971 269	3 of 5
Southeast Asia	1990–2009	16 095 170	13 of 13	89 276	1 of 13	662 017	8 of 13	364 423	8 of 13	3 471 130	9 of 13	2 478 300	9 of 13
	2010–2021	10 140 950	13 of 13	..	..	45 912	7 of 13	20 488	8 of 13	44 819 274	10 of 13	44 534 172	8 of 13
Southern Latin America	1990–2009	11 202 740	3 of 3	..	..	195 396	2 of 3	28 813	2 of 3	315 381	3 of 3	101 458	3 of 3
	2010–2021	4 101 015	3 of 3	..	..	464 675	2 of 3	187 474	2 of 3	1 610 206	3 of 3	1 496 431	3 of 3
Southern sub-Saharan Africa	1990–2009	6 899 016	6 of 6	3 078 169	1 of 6	66 048	2 of 6	12 458	4 of 6	371 099	4 of 6	39 088	4 of 6
	2010–2021	3 905 966	6 of 6	2 058 541	1 of 6	262 203	2 of 6	198 205	6 of 6	789 849	6 of 6	535 563	6 of 6
Tropical Latin America	1990–2009	27 322 900	2 of 2	18 530 993	1 of 2	238 892	1 of 2	369 451	2 of 2	264 189	2 of 2	339 079	2 of 2
	2010–2021	12 692 420	2 of 2	9 211 493	1 of 2	46 175	1 of 2	101 524	1 of 2	86 099	2 of 2	30 973	2 of 2
Western Europe	1990–2009	114 548 600	24 of 24	5 166 236	2 of 24	1 716 285	19 of 24	1 491 426	21 of 24	4 367 966	20 of 24	4 728 278	22 of 24
	2010–2021	36 034 880	24 of 24	9 610 507	2 of 24	8 303 874	19 of 24	6 104 507	20 of 24	41 382 029	21 of 24	43 421 657	21 of 24
Western sub-Saharan Africa	1990–2009	115 334	19 of 19	..	..	3605	2 of 19	11 950	10 of 19	138 332	14 of 19	74 493	9 of 19
	2010–2021	29 247	19 of 19	409	2 of 19	76 886	6 of 19	6551	13 of 19	282 917	15 of 19	269 633	14 of 19
Total	1990–2021	701 762 090	195 of 204	149 002 363	23 of 204	50 864 030	104 of 204	24 251 653	142 of 204	322 576 481	161 of 204	296 231 257	154 of 204

* We multiplied our results from estimation step 1 on the CoDCorrect estimates from the GBD, which are produced using data from the GBD's Cause of Death database, outlined in this table as step 0.

All data inputs for the models used in steps 1–10 (appendix 1 p 80) were empirical data, not modelled estimates, with the exception of a custom meta-analysis of vaccine probe data that we did to estimate the fraction of pneumonia caused by *Streptococcus pneumoniae* (appendix 1 pp 40–41). All study-level covariates for models, such as age and sex, were extracted from empirical data. All country-level covariates were modelled estimates that were produced previously for GBD^22^, ^32^ and the Antimicrobial Collaborators Network,^7^ or modelled using a method previously described in Browne and colleagues.^24^ Data inputs for each estimation step are described in greater detail below and in appendix 1 (pp 17–20, 32, 36, 50–51, 60). Data input citations are available online.

### Sepsis and infectious syndromes (steps 1 and 2)

First, to define the number of sepsis deaths, we used GBD 2021 cause of death estimates^14^ to determine the number of deaths by age, sex, and location where the pathway to death on the death certificate included sepsis. Sepsis was defined as life-threatening organ dysfunction due to a dysregulated host response to infection.^25^ The methods used to estimate infectious underlying causes of death and sepsis have been previously published^10^ and are summarised in appendix 1 (pp 17–20).

In modelling step one (appendix 1 p 80), multiple cause of death data covering 139 million deaths, 10·1 million hospital discharges with discharge status of death, 288 315 multiple cause of death records linked to hospital records from ten countries and territories, as well as 1805 deaths from Child Health and Mortality Prevention Surveillance sites across seven countries (appendix 1 pp 18–20) were used in mixed effects logistic regression models to predict the fraction of sepsis occurring in each of the 195 GBD underlying causes of death. This approach follows the methods validated by many studies on the epidemiology of sepsis^26^, ^27^, ^28^, ^29^ and was used by Rudd and colleagues.^10^ We then multiplied the fraction of sepsis predicted from the logistic regression models onto GBD cause-age-sex-year-specific and location-specific mortality estimates to determine the mortality envelope for our analysis. Our mortality envelope consisted of all sepsis deaths in non-infectious underlying causes, plus all explicit sepsis deaths with an infectious underlying cause in GBD (appendix 1 pp 22–24). COVID-19 contributed to the sepsis mortality envelope but did not contribute to our estimation of AMR burden.

In modelling step two, details on the pathways of disease provided in the multiple cause of death data or hospital discharge data were used in a second stage of mixed effects logistic regression models to further subdivide sepsis deaths into 22 major infectious syndromes, of which 11 had both bacterial aetiologies for which we estimate AMR burden and sufficient data available for pathogen modelling (appendix 1 p 21). These regressions predicted the fraction of sepsis-related deaths that were caused by a given infectious syndrome, separately for each GBD underlying cause of death, location, age, sex, and year. We used this fraction to subdivide sepsis deaths in non-infectious underlying causes into specific infectious syndromes. For underlying causes of death that are themselves infectious, all deaths were assigned to a single corresponding infectious syndrome (eg, GBD cause “lower respiratory infections” was assigned to infectious syndrome “lower respiratory infections”; appendix 1 pp 22–24).

### Case fatality rates and pathogen distribution for deaths and incident cases (steps 3 and 4)

For analytical step three (appendix 1 p 80), we took data that linked pathogen-specific disease incidence to deaths to develop models for pathogen-specific case-fatality ratios (CFRs) that varied by age, year, location, and infectious syndrome. Depending on data quality and availability, we used four levels of granularity per pathogen-syndrome, estimating (from most detail to least): a unique model for that pathogen-syndrome, a unique intercept for that pathogen-syndrome in a model with data for all pathogens, a pooled model for each given infectious organism type (eg, bacteria, fungus, parasite, and virus), or a pooled model using all data. CFR models were implemented with the RegMod^30^ regression model environment and generated CFRs as a function of the Healthcare Access and Quality (HAQ) Index and various bias-adjusting covariates (appendix 1 pp 32–35).^23^ We then used CFR results to estimate implied cases for each pathogen from mortality-only data sources that otherwise reported useful information on pathogen distributions. In total, the pathogen distribution models leveraged 24 million isolates and cases from 142 countries and territories to estimate the pathogen distribution of each infectious syndrome by age, year, and location, with each dataset including a unique spectrum of pathogens and groups of pathogens. To incorporate these heterogeneous data, we used the multinomial estimation with partial and composite observations modelling environment, which allows for the inclusion of covariates in the network analysis^31^ and for Bayesian priors to be incorporated. To model the infectious syndrome pathogen distribution comprehensively, we estimated, where applicable, the incidence and death proportions attributable to viral, fungal, parasitic, and bacterial pathogens; however, AMR burden was calculated only for selected bacteria for which resistance is clinically relevant and sufficient data are available. More details on this approach are provided in appendix 1 (pp 36–50).

### Prevalence of resistance by pathogen (steps 5 to 7)

The 84 pathogen–antibiotic class combinations were selected by first creating an exhaustive list of all clinically relevant combinations for which we had data, and then eliminating combinations that did not meet minimum data availability and computational feasibility requirements for accurate statistical modelling (appendix 1 p 70). We supplemented microbial datasets from collaborators with aggregate data from systematic reviews and published surveillance reports. The number of isolates assessed for resistance for each pathogen–drug combination is shown in appendix 1 (pp 121–124).

When classifying resistance, we used two categories, susceptible and non-susceptible, the latter of which included dose-dependent, intermediate, and resistant interpretations. We excluded combinations of antibiotics to which bacteria have intrinsic resistance (appendix 1 pp 51–55). Two-thirds of datasets used in the prevalence of resistance modelling reported a laboratory susceptibility interpretation rather than quantitative test values. Data providers used several guidelines (and therefore breakpoints) to interpret test results, which potentially introduced bias. Information on the minimum inhibitory concentration of antibiotics was available in the remaining third of microbiology data sources. This information was used both to categorise bacterial isolates into susceptible and non-susceptible based on the 2023 Clinical and Laboratory Standard Institute (CLSI) breakpoints (our gold standard), and to infer the relationship between proportions of resistance when classifying our data using other versions of CLSI and the European Committee on Antimicrobial Susceptibility Testing guidelines. These relationships were then used to create adjustment factors and correct the data that only reported laboratory interpretations as described in appendix 1 (pp 51–55). Additionally, to account for bias in resistance data provided by tertiary care facilities, we adjusted tertiary rates of resistance by crosswalking them to data from non-tertiary and mixed facilities (appendix 1 pp 51–55).

After adjustments, we employed a two-stage spatiotemporal modelling framework to estimate the prevalence of resistance in each pathogen–drug combination by location-year for 1990–2021. First, we fit a stacked ensemble model between the input data and selected covariates from the list of plausible and health-related covariates available in GBD (appendix 1 pp 56, 102–108). As a second stage, the estimates from the stacked ensemble model were then input into a spatiotemporal Gaussian process regression^32^ model to smooth the estimates in space and time. The exceptions to this modelling approach were made for tuberculosis (both extensively drug-resistant tuberculosis and multidrug-resistant excluding extensively drug-resistant tuberculosis), for which published GBD 2021 estimates were already available.^19^

Given the strong relationship between antibiotic consumption levels and the proliferation of resistance, we modelled antibiotic consumption at the national level to use as a covariate in the stacked ensemble model of prevalence of resistance. Data from 65 Demographic and Health Surveys and 138 Multiple Indicator Cluster Surveys were analysed using model-based geostatistics to quantify antibiotic usage in LMICs. This was combined with pharmaceutical sales data from IQVIA, WHO, and the European Centre for Disease Prevention and Control: using an ensemble spatiotemporal Gaussian process regression model.^24^ Antibiotic use and sales data for 1990–2018 were used to produce a location-year covariate on antibiotic consumption for all 204 countries and territories between 1990 and 2021 included in this study (appendix 1 pp 32–35, 56, 80).^24^

To account for multidrug resistance, we used line-level microbiology data that tested the same isolate for resistance to multiple antibiotics to produce both marginal frequency of resistance and pairwise co-occurrences for each pair of antibiotics. Using the marginal and co-occurrence information, we then found the ‘best' multinomial distribution that matched available data in the least square sense. The method (appendix 1 p 56) reframes the optimisation problem over the space of multivariate probability measures as a least squares optimisation problem over the space of *n*-dimensional probability simplices, using the marginal and pairwise co-occurrence information as data. The method is guaranteed to find a solution even if observations are noisy, or mutually inconsistent. In case of inconsistent observations, the method still provides the multinomial distribution that approximately matches the margins, best in the least squares sense. The approach was applied for every location-year combination of resistance to the antibiotics analysed (appendix 1 p 56).

### Relative risk of death for drug-resistant infection compared with drug-sensitive infections (steps 8 and 9)

Using data from 1238 sources representing 296 million samples from patients with outcome and resistance, we estimated the relative risk of death for each pathogen–drug combination for a resistant infection compared with a drug-sensitive infection using the meta-regression—Bayesian, regularised, trimmed tool (appendix 1 p 80). Because of data sparsity, we estimated the relative risk by antibiotic class, pathogen, and infectious syndrome; we assumed that risk did not vary by location and age, which is consistent with the assumptions made by Cassini and colleagues.^9^ We used a two-stage modelling approach with a mixed effects binomial logistic regression and a mixed effects meta-regression model to estimate relative risk of death for each pathogen–drug combination (appendix 1 pp 60–65). For the non-fatal excess risk, we estimated the relative increase in length of stay associated with a resistant infection compared with a susceptible infection of the same kind (appendix 1 p 80). Data on length of stay were available from 309 sources representing 38 million admissions. We used a similar modelling framework for excess length of stay as we used for relative risk of death with a two-stage nested mixed effects meta-regression model. Due to data sparsity associated with drug-resistant *N gonorrhoeae*, we only produced a non-fatal estimate for this pathogen. To produce burden estimates of multiple pathogen–drug combinations that were mutually exclusive within a given pathogen (and thus could be added), we produced a population attributable fraction (PAF) for each resistance profile with resistance to at least one drug (appendix 1 pp 65–72).

### Computing burden attributable to drug resistance and burden associated with drug-resistant infections (step 10)

We computed two counterfactuals to estimate the drug-resistant burden: the burden attributable to bacterial AMR based on the counterfactual of drug-sensitive infection and the burden associated with bacterial AMR based on the counterfactual of no infection. These methods are described in greater detail in appendix 1 (pp 65–72). Briefly, to estimate the burden attributable to AMR, we first calculated the deaths attributable to resistance by taking, for each underlying cause, the product of cause-specific deaths, the fraction of these deaths co-occurring with sepsis, the fraction of sepsis deaths attributable to each infectious syndrome, the fraction of infectious syndrome deaths attributable to each pathogen, and the mortality PAF for each resistance profile. We used standard GBD methods^19^ to convert age-specific deaths into years of life lost (YLLs) using the standard counterfactual life expectancy at each age.^33^ To calculate attributable years lived with disability (YLDs), we took the product of the infectious syndrome incidence (appendix 1 pp 27–32), the fraction of infectious syndrome incident cases attributable to each pathogen, YLDs per incident case, and the non-fatal PAF. For resistance profiles that had resistance to more than one antibiotic class, we redistributed burden to the individual antibiotic classes proportionally based on excess risk, providing a mutually exclusive burden for each pathogen–drug combination (appendix 1 pp 65–72). To calculate DALYs, we took the sum of YLLs and YLDs. To estimate the overall AMR burden of the drug-sensitive counterfactual, we summed the burden estimates of every pathogen–drug combination. The approach for calculating the fatal burden associated with AMR was identical except we replaced the mortality PAF for each resistance profile with the prevalence of resistance in deaths (appendix 1 pp 65–72).

#### Decomposition of the factors contributing to change in AMR associated deaths

To better understand the relative contributions of various factors to the overall change in deaths associated with AMR, we did a 6-factor decomposition analysis, drawing from methods developed by Das Gupta.^34^, ^35^ This decomposition accounted for the following factors: (1) population growth, (2) the population age structure, (3) the sepsis mortality rate, (4) the proportion of sepsis deaths associated with AMR syndromes, (5) the proportion of AMR syndrome deaths associated with AMR bacteria, and (6) the proportion of AMR bacteria deaths associated with resistance. The decomposition was done for global and GBD super-region level estimates comparing 1990 to 2019. For a detailed description of the methods see appendix 1 (pp 70–72).

### AMR forecasts

IHME's Future Health Scenarios framework produces forecasts of disease burden by cause, age, sex and location from 2022–2050. These estimates of disease burden were combined with forecasts of AMR to forecast the future burden attributable to and associated with AMR. Detailed methods for the Future Health Scenarios framework are described in detail by Vollset and colleague.^17^

#### Forecasting AMR population attributable fractions

We used the historical estimates of deaths due to AMR (attributable deaths) by GBD cause and computed 19 PAFs for GBD Level 2 causes with AMR attributable death counts. We then used a Generalized Ensemble Model to forecast the fraction of cause-specific deaths due to AMR. This model used two main modelling approaches: the weighted annualised rate of change and a two-stage spline model based on the meta-regression—Bayesian, regularised, trimmed tool^31^ to generate 12 difference sub-models (appendix 1 pp 73–74). We then obtained the final AMR PAFs ensemble forecasts by taking a mean over these submodels using the sampling weights from the out-of-sample experiments. Finally, we applied these PAF forecasts to our reference cause-specific forecasts of mortality.^17^

#### Computing future attributable and associated burden

For computing attributable AMR burden, we first multiplied our reference mortality and YLL forecasts for 19 cause groups at the age-sex-location level by the forecasted AMR PAFs described in the section above. Afterwards, we computed AMR-attributable YLDs by applying a scalar to the attributable YLLs using the global ratio of YLL:YLD AMR deaths in 2019.^6^ Finally, we computed AMR-attributable DALYs by taking the sum of AMR-attributable YLLs and YLDs.

For computing associated AMR burden, first, we computed the ratio of AMR-associated deaths to AMR-attributable deaths for 19 cause groups by age-sex-location in 2021. We then multiplied our AMR PAFs by the resulting ratio to calculate associated burden forecasts for each measure using the same methods as computing attributable burden.

#### Developing AMR scenarios

The reference scenario provides a probabilistic forecast of the most likely future. In addition to a reference scenario, we produced two alternative scenarios: the Gram-negative drug​ scenario and the better care scenario. We developed the Gram-negative drug scenario under the assumption that a regular release of new, potent antibiotics targeting Gram-negative bacteria would lead to decreases in AMR burden. To simulate this, we calculated the fraction of AMR burden caused by Gram-negative pathogens and linearly reduced it by 50% of the value observed from 2021 to 2036 (appendix 1 p 74). For the better care scenario, we assumed that better health-care quality for infectious syndromes and access to antibiotics would lead to decreases in both AMR burden and infectious burden not associated with AMR. To calculate the death rates for this scenario for a cause, we used total death rate for a cause, fraction of the cause due to infectious syndrome, and CFRs that varied by age, sex, location, and infectious syndrome (appendix 1 p 74). First, we obtained CFR values for an infectious syndrome that corresponds to the 85th percentile of HAQ Index^23^ in 2021 (HAQ Index=84·16) by location and age group using the age-specific relationship curves. Afterwards, we applied a relative reduction to the cause-specific mortality rate in 2021 (for countries with HAQ Index below 85th percentile) to reach the target CFR by 2030 to replicate the effect of improved treatment outcomes.

### Uncertainty analysis and out-of-sample validation

We propagated uncertainty by combining 100 draws for each location, sex, age, and year from the posterior distribution from each step of the analysis into the final estimates of deaths and infections associated with drug resistance and deaths and infections attributable to drug resistance. We calculated uncertainty intervals as a standard deviation of 1·96 below and above the mean value. Out-of-sample validity estimates are provided in appendix 1 for our models of sepsis, infectious syndrome distribution (pp 27–32), pathogen distribution (p 50), prevalence of resistance (pp 50–58), and relative risk (pp 60–63). Uncertainty intervals for future estimates were computed from the 2·5 and 97·5 percentiles of distributions generated from propagating 500 draws through the multistage computational pipeline. This study complies with the Guidelines for Accurate and Transparent Health Estimates Reporting recommendations (appendix 1 pp 74, 112–113).^36^

### Role of the funding source

The funders of the study had no role in study design, data collection, data analysis, data interpretation, or the writing of the report.

## Results

**Figure 1:**
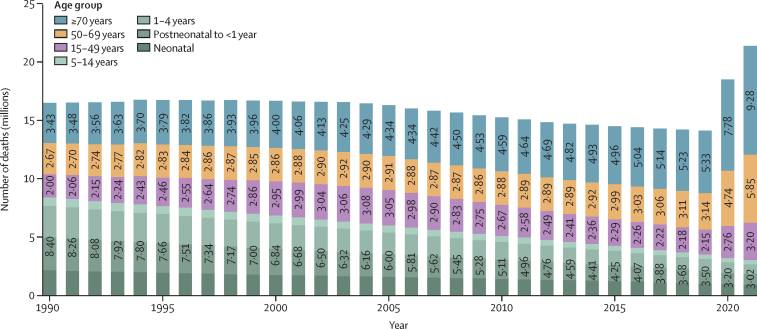
Global time trend of sepsis, by age, 1990–2021 Bar labels represent the number of sepsis deaths in a given year for people aged 0–14 years, 15–49 years, 50–69 years, and ≥70 years. Values for the age group of 0–14 years represent the sum of sepsis deaths among neonates, postneonates to <1 year, 1–4 years, and 5–14 years.

Globally, we estimated 16·5 million (95% UI 15·7–17·3) deaths with sepsis in 1990, which decreased to 14·1 million (13·2–15·1) deaths in 2019, before increasing to 21·4 million (20·3–22·4) deaths in 2021 (figure 1). In children younger than 5 years, deaths with sepsis decreased by more than 60% over the past 31 years, from 7·69 million (7·16–8·22) in 1990 to 3·14 million (2·66–3·62) in 2019, and 2·68 million (2·19–3·18) in 2021. In people 5 years and older, however, deaths with sepsis more than doubled over the study period, from 8·81 million (8·30–9·32) in 1990, to 11·0 million (10·2–11·7) in 2019, and 18·7 (17·8–19·6) million in 2021, of which 7·89 million (7·41–8·38) were due to COVID-19 (appendix 1 p 118).

### AMR burden in 1990–2021

For the 84 bacterial pathogen–drug combinations evaluated in the study, there were 1·06 million (95% UI 0·841–1·27) deaths attributable to AMR and 4·78 million (4·00–5·55) deaths associated with AMR in 1990 (table 2). In 2019, AMR mortality increased to 1·20 million (1·05–1·35) attributable deaths and 4·94 million (4·43–5·45) associated deaths. In 2021, 1·14 (1·00–1·28) million deaths attributable to AMR and 4·71 million (4·23–5·19) deaths associated with AMR occurred, representing a decrease in both estimates from 2019. The global all-age mortality rate attributable to AMR decreased from 19·8 deaths (15·8–23·9) per 100 000 in 1990 to 15·5 deaths (13·6–16·4) per 100 000 in 2019 and 14·5 deaths (12·7–16·2) per 100 000 in 2021. However, the percentage of deaths with sepsis that were associated with AMR increased from 29% in 1990 to 35% globally in 2019, before decreasing to 22% in 2021. Additionally, the percentage of sepsis deaths that were attributable to AMR increased from 6·41% (5·37–7·36) in 1990 to 8·51% (8·00–8·95) globally in 2019, before decreasing to 5·34% (4·95–5·70) in 2021. Excluding COVID-19, 8·47% (8·45–8·49) of infectious deaths in 2021 were attributable to AMR. Table 3, Table 4 show the all-age mortality rate associated with and attributable to AMR in 1990 and 2021, respectively. A decomposition of the drivers of change in global deaths associated with AMR from 1990 to 2019 are presented in table 5.

**Table 3:** Deaths (in counts and all-age rates) associated with and attributable to bacterial antimicrobial resistance, globally, by GBD super-region, for 1990, 2019, 2021 and 2050

	**Associated death counts (thousands)**				**Associated death rates per 100 000**				**Attributable death counts (thousands)**				**Attributable death rate per 100 000**			
	1990	2019	2021	2050	1990	2019	2021	2050	1990	2019	2021	2050	1990	2019	2021	2050
Global	4780 (4000–5550)	4940 (4430–5450)	4710 (4230–5190)	8220 (6850–9650)	89·6 (75·0–104)	63·8 (57·2–70·4)	59·7 (53·6–65·7)	87·7 (73·2–104)	1060 (841–1270)	1200 (1050–1350)	1140 (1000–1280)	1910 (1560–2260)	19·8 (15·8–23·9)	15·5 (13·6–17·4)	14·5 (12·7–16·2)	20·4 (16·6–24·2)
Central Europe, Eastern Europe, and Central Asia	285 (240–330)	281 (252–310)	265 (235–295)	360 (297–420)	67·8 (57·1–78·4)	67·1 (60·1–74·0)	63·4 (56·2–70·6)	90·5 (76·3–106)	63·0 (49·5–76·6)	68·6 (59·2–78·0)	64·0 (55·2–72·8)	82·9 (67·1–98·7)	15·0 (11·8–18·2)	16·4 (14·1–18·6)	15·3 (13·2–17·4)	20·8 (17·0–24·8)
High-income	477 (385–568)	579 (513–644)	553 (489–618)	883 (674–1040)	52·5 (42·4–62·5)	53·3 (47·3–59·3)	50·7 (44·8–56·6)	78·1 (59·8–92·2)	108 (82·9–133)	131 (115–146)	125 (110–140)	192 (146–225)	11·9 (9·12–14·7)	12·0 (10·6–13·5)	11·4 (10·1–12·8)	17·0 (12·9–19·9)
Latin America and Caribbean	247 (210–284)	339 (305–372)	322 (285–360)	650 (520–808)	63·3 (53·8–72·8)	57·9 (52·2–63·6)	54·2 (47·9–60·6)	96·7 (78·0–119)	55·7 (44·4–67·0)	82·4 (72·4–92·5)	78·1 (67·7–88·6)	148 (117–185)	14·3 (11·4–17·2)	14·1 (12·4–15·8)	13·2 (11·4–14·9)	22·1 (17·5–27·2)
North Africa and Middle East	264 (219–310)	243 (211–274)	226 (194–259)	525 (430–641)	78·0 (64·5–91·4)	40·0 (34·8–45·2)	36·3 (31·1–41·5)	61·4 (49·2–73·9)	63·0 (49·4–76·7)	64·7 (54·4–75·1)	60·2 (50·1–70·3)	133 (107–161)	18·6 (14·6–22·6)	10·7 (8·97–12·4)	9·66 (8·05–11·3)	15·5 (12·3–19·0)
South Asia	1400 (1170–1630)	1350 (1200–1500)	1260 (1110–1420)	2400 (1910–2980)	128 (107–149)	74·7 (66·5–83·0)	68·5 (60·2–76·8)	114 (91·3–141)	308 (249–367)	356 (303–408)	335 (282–387)	604 (463–743)	28·2 (22·8–33·6)	19·7 (16·8–22·6)	18·1 (15·3–21·0)	28·8 (22·5–35·6)
Southeast Asia, East Asia, and Oceania	1110 (916–1310)	1140 (995–1290)	1150 (1010–1290)	1940 (1580–2370)	65·8 (54·2–77·5)	52·8 (46·0–59·5)	52·7 (46·3–59·1)	92·0 (75·0–113)	250 (195–304)	271 (235–307)	270 (237–304)	428 (348–511)	14·8 (11·5–18·0)	12·5 (10·9–14·2)	12·4 (10·8–13·9)	20·3 (16·4–24·5)
Sub-Saharan Africa	990 (797–1180)	1010 (811–1200)	923 (732–1110)	1470 (1140–1860)	202 (162–241)	93·2 (75·1–111)	81·5 (64·6–98·4)	69·3 (53·7–87·5)	210 (158–262)	227 (179–276)	209 (161–257)	323 (245–416)	42·8 (32·2–53·3)	21·0 (16·5–25·5)	18·5 (14·2–22·7)	15·3 (11·5–19·5)

**Table 4:** DALYs (in counts and all-age rates) associated with and attributable to bacterial antimicrobial resistance, globally, by GBD super-region, for 1990, 2019, 2021 and 2050

	**Associated DALY counts (thousands)**				**Associated DALY rates per 100 000**				**Attributable DALY counts (thousands)**				**Attributable DALY rates per 100 000**			
	1990	2019	2021	2050	1990	2019	2021	2050	1990	2019	2021	2050	1990	2019	2021	2050
Global	281 000 (232 000–331 000)	196 000 (170 000–221 000)	178 000 (154 000–202 000)	201 000 (165 000–245 000)	5270 (4350–6200)	2530 (2190–2860)	2250 (1950–2550)	2140 (1750–2630)	60900 (47700–74100)	46 800 (39 800–53 700)	42 600 (36 100–49 000)	46 500 (37 700–57 300)	1140 (895–1390)	604 (514–693)	539 (458–621)	496 (399–613)
Central Europe, Eastern Europe, and Central Asia	10500 (8770–12100)	7840 (7010–8670)	7190 (6380–8000)	7310 (6010–8680)	2480 (2080–2880)	1870 (1670–2070)	1720 (1530–1920)	1840 (1530–2180)	2310 (1790–2840)	1940 (1650–2230)	1760 (1500–2030)	1700 (1380–2060)	550 (426–674)	464 (394–533)	422 (359–485)	428 (348–516)
High-income	11 100 (9090–131 00)	11 700 (10 700–12 700)	11 200 (10 200–12 200)	12 800 (10500–14600)	1220 (1000–1450)	1080 (988–1170)	1030 (937–1120)	1130 (931–1290)	2560 (1970–3140)	2700 (2450–2960)	2590 (2340–2830)	2810 (2280–3220)	281 (217–345)	249 (225–273)	237 (214–260)	248 (203–285)
Latin America and Caribbean	13 700 (11 500–15 900)	10 400 (9260–11 400)	9600 (8420–10 800)	12 600 (10 200–15 600)	3510 (2960–4070)	1770 (1580–1950)	1620 (1420–1810)	1880 (1530–2300)	3030 (2390–3670)	2540 (2220–2860)	2350 (2030–2680)	2910 (2330–3620)	776 (612–940)	434 (380–488)	396 (341–451)	433 (347–537)
North Africa and Middle East	17 600 (14 300–20 900)	10 100 (8470–11 600)	8770 (7350–10 200)	13 100 (10400–16 700)	5190 (4210–6170)	1660 (1400–1920)	1410 (1180–1640)	1530 (1210–1930)	4140 (3190–5090)	2670 (2180–3160)	2330 (1900–2760)	3330 (2590–4280)	1220 (940–1500)	441 (360–522)	374 (305–443)	390 (303–493)
South Asia	96 400 (79 300–113 000)	59 200 (51 200–67 100)	52 200 (44 900–59 400)	57 700 (44 900–72 400)	8810 (7250–10 400)	3270 (2840–3710)	2830 (2430–3220)	2750 (2170–3500)	20 800 (16 500–25 000)	15 000 (12 600–17 300)	13 300 (11 100–15 500)	14 400 (10 900–18 200)	1900 (1510–2290)	828 (696–959)	721 (602–839)	687 (527–881)
Southeast Asia, East Asia, and Oceania	58 500 (47 300–69 700)	32 400 (28 700–36 000)	31 400 (28 000–34 800)	35 200 (29 300–42 800)	3460 (2800–4120)	1500 (1330–1670)	1440 (1280–1590)	1670 (1410–2020)	12700 (9750–15600)	7710 (6760–8660)	7430 (6560–8300)	7870 (6580–9480)	751 (577–924)	356 (312–400)	340 (300–380)	374 (311–453)
Sub-Saharan Africa	73 400 (58 000–88 900)	64 100 (49 900–78 300)	57 300 (43 400–71 200)	62 100 (43 100–86 000)	14 900 (11 800–18 100)	5940 (4620–7260)	5060 (3830–6280)	2930 (2100–4160)	15400 (11400–19400)	14200 (10900–17600)	12800 (9450–16100)	13500 (9210–18800)	3140 (2320–3950)	1320 (1010–1630)	1130 (834–1420)	637 (448–899)

**Table 5:** A decomposition analysis that quantifies the effect of different contributory factors on the number of AMR-associated deaths between 1990 and 2019

		**Number of AMR-associated deaths**
AMR-associated deaths, 1990		4 770 000
Factors driving changes in AMR deaths between 1990 and 2019		
	Population growth	+1 740 000
	Age structure	+792 000
	Sepsis death rate	−3 090 000
	Proportion of sepsis deaths associated with AMR syndromes	−181 000
	Proportion of AMR syndrome deaths associated with AMR bacteria	+227 000
	Proportion of AMR bacteria deaths associated with resistance	+675 000
Net change		163 000
AMR-associated deaths, 2019		4 940 000

Each row represents a distinct factor, showing how changes in that factor alone would have influenced the AMR-associated deaths, assuming all the other factors remained constant. For example, if AMR-associated deaths were affected solely by the observed population growth between 1990 and 2019, with no changes to age structure of the population, the proportion of sepsis deaths associated with AMR, or any other factor, AMR-associated deaths would increase by 1 740 000 from 4 770 000 in 1990 to 6 510 000 in 2019. All factors influenced the observed change in AMR-associated deaths; whereas population growth, changes in age structure, and an increased proportion of AMR syndrome deaths due to AMR bacteria and resistance contributed to a rise in deaths, this was counterbalanced by a reduction in deaths from decreasing sepsis death rates. When combining all measured factors, there is a net increase of 163 000 AMR-associated deaths from 1990 to 2019. Factors were decomposed through 2019 to provide a simpler assessment without the added complexities of the COVID-19 pandemic. AMR syndromes are the 11 infectious syndromes for which we assessed the aetiologies. AMR bacteria are the 22 bacterial pathogens for which we quantified AMR burden. Further information is provided in appendix 1 (pp 70–71). AMR=antimicrobial resistance.

The global number of DALYs attributable to AMR decreased between 1990 (60·9 million [95% UI 47·7–74·1]) and 2021 (42·6 million [36·1–49·0]). Over the same period, DALYs associated with AMR also decreased from 281 million (232–331) in 1990 to 178 million (154–202) in 2021. Similarly, the global all-age rate of DALYs attributable to AMR decreased from 1140 per 100 000 population (895–1390) in 1990 to 539 per 100 000 population (458–621) in 2021, and the all-age rate of DALYs associated with AMR decreased from 5270 per 100 000 population (4350–6200) in 1990 to 2250 per 100 000 population (1950–2550) in 2021. Table 3, Table 4 provide estimates of deaths and DALYs from AMR for each counterfactual for 1990, 2005, 2019, 2021, and 2050.

**Figure 2:**
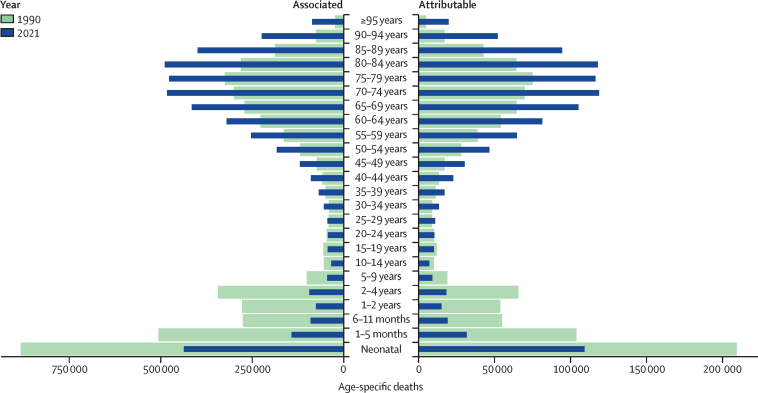
Deaths attributable and associated with antimicrobial resistance, by detailed age group, for 1990 and 2021 Counterfactuals have distinct x-axes.

Among deaths that were attributable to or associated with AMR, there was a notable divergence in trends across age groups (figure 2). Globally, children younger than 5 years had a more than 50% reduction in both attributable (60·4% [95% UI 47·6–73·1]) and associated (63·3% [52·8–73·8]) AMR mortality from 1990 to 2021. By contrast, all age groups from 25 years and older had an increase in AMR mortality over the same period, with adults 70 years and older experiencing a more than 80% increase in both attributable (89·5% [47·4–131·7]) and associated (81·3% [48·3–114·3]) mortality over this time period (figure 2). For children younger than 5 years in 2021, there were 193 000 deaths (144 000–242 000) attributable to AMR globally, a decrease from 488 000 deaths (374 000–602 000) attributable to AMR in 1990. In 2021, there were 840 000 (640 000–1·04 million) AMR-associated deaths in children younger than 5 years globally, compared with 2·29 million (1·85–2·72) associated deaths in 1990. In people 5 years and older, there were 948 000 deaths (837 000–1·06 million) attributable to AMR and 3·87 million (3·48–4·26) deaths associated with AMR in 2021, both increasing from 570 000 (458 000–682 000) attributable and 2·49 million (2·10–2·88) associated AMR deaths in 1990 (figure 2). The fraction of deaths with sepsis attributable to AMR increased in all age groups, with a 18·0% increase (–9·03 to 45·0) in children younger than 5 years and an increase of 35·9% (9·98 to 61·9) in people 5 years and older from 1990 to 2019 (appendix 1 p 118).

**Figure 3:**
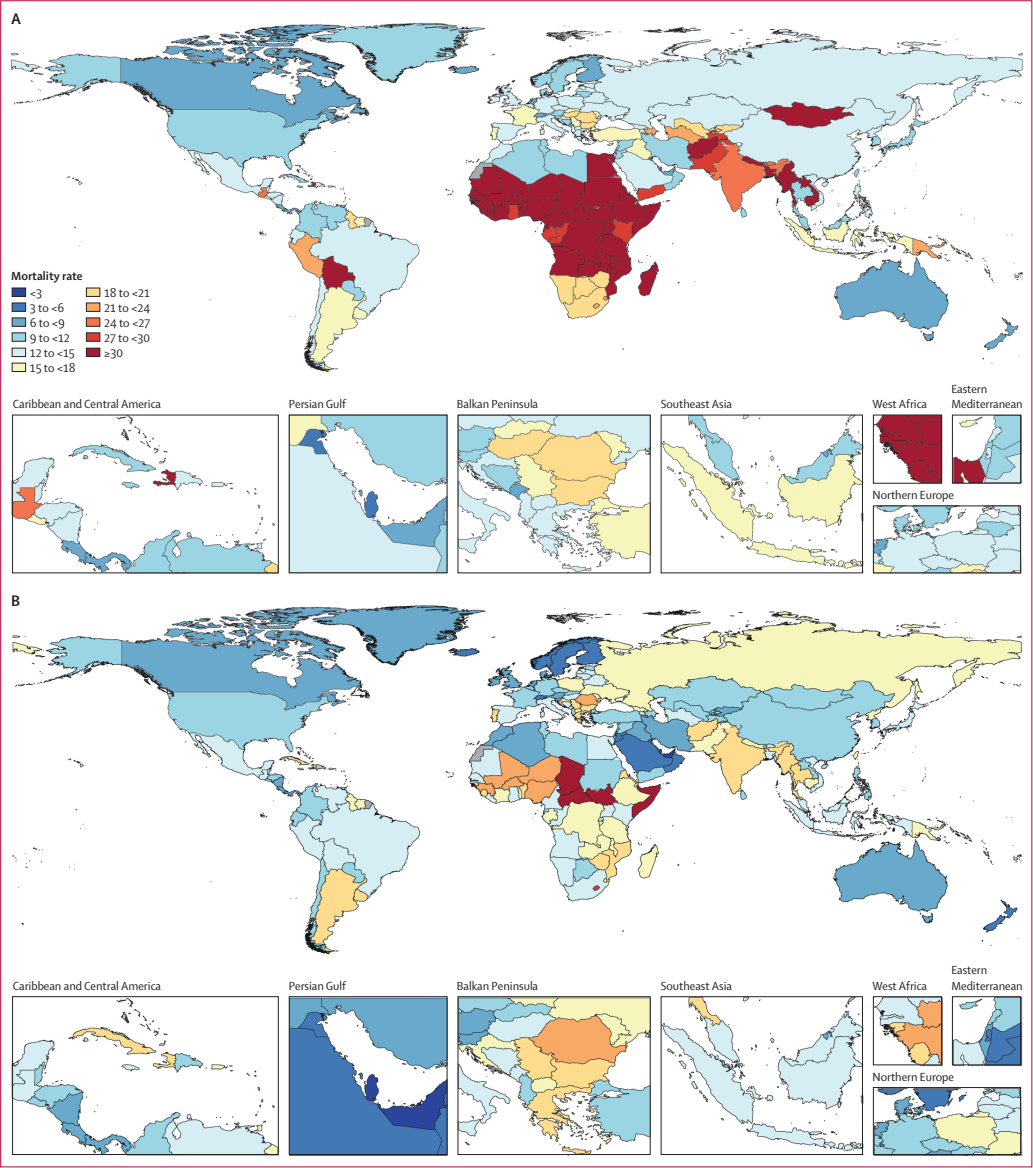

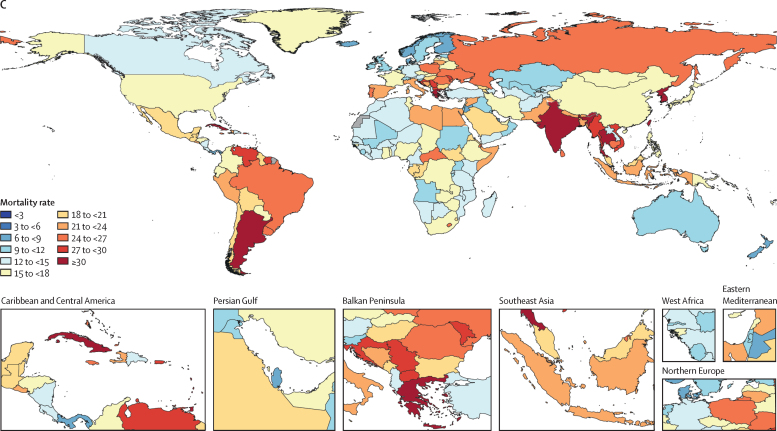
Death rates per 100 000 attributable to AMR, all ages, 1990, 2021, 2050 (A) Death rate attributable to AMR, all ages, 1990. (B) Death rate attributable to AMR, all ages, 2021. (C) Death rate attributable to AMR, all ages, 2050. AMR=antimicrobial resistance.

Attributable AMR deaths decreased in seven regions and increased in 14 regions between 1990 and 2021 (table 3). Five regions had an increase of more than 10 000 deaths attributable to AMR: western sub-Saharan Africa, Tropical Latin America, high-income north America, southeast Asia, and south Asia. For children younger than 5 years, from 1990 to 2021, deaths associated with and attributable to AMR decreased by 9% to 95% in all regions except Oceania. Conversely, in people 5 years and older, AMR mortality increased from 1990 to 2021 in all regions except for western Europe and central Europe. In western sub-Saharan Africa, 58·9% (54·8–63·0) of the attributable AMR mortality occurred in children younger than 5 years, and 15·8% (13·5–18·2) occurred in adults 70 years and older in 2021. In comparison, in the high-income Asia Pacific region, 0·181% (0·1490–0·222) of the attributable AMR mortality occurred in children younger than 5 years and 83·9% (81·7–86·1) of the attributable AMR mortality was in adults 70 years and older for 2021 (figure 2; figure 3B). Table 3 shows the number of deaths attributable to and associated with AMR at the regional level.

**Figure 4:**
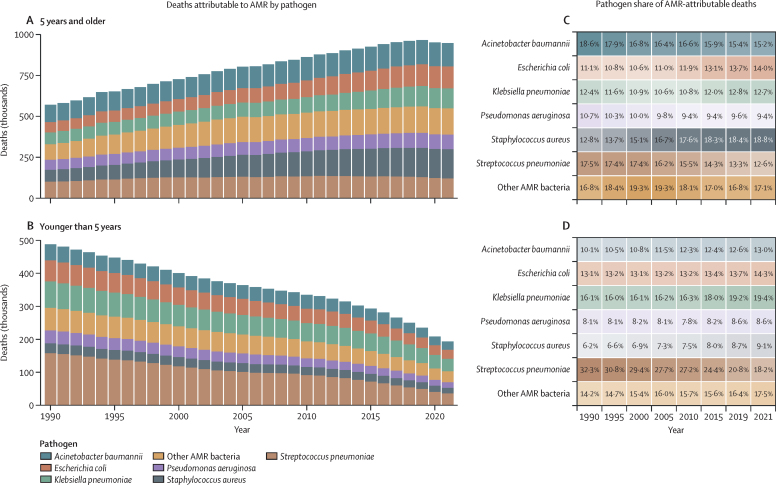
Deaths attributable to AMR by pathogen, global, 1990–2021, for people 5 years and older and those younger than 5 years (A) and (B) represent total AMR-attributable deaths by pathogen. (C) and (D) represent the proportion of total AMR deaths attributable to a given pathogen. Other AMR bacteria are *Citrobacter* spp, *Enterobacter* spp, *Enterococcus faecalis, Enterococcus faecium, Haemophilus influenzae, Morganella* spp, *Mycobacterium tuberculosis, Proteus* spp, non-typhoidal *Salmonella, Salmonella enterica* serovar Typhi, *Salmonella enterica* serovar Paratyphi, *Serratia* spp, *Shigella* spp, group A *Streptococcus* (*Streptococcus pyogenes*), and group B *Streptococcus* (*Streptococcus agalactiae*). AMR=antimicrobial resistance.

There was a global decline in attributable AMR deaths for nine pathogens and an increase in 12 pathogens from 1990 to 2021 (figure 4). The pathogen with the most substantial decline was *S pneumoniae*, with a decrease from 1·30 million (95% UI 1·01–1·58) associated deaths and 258 000 (179 000–336 000) attributable deaths in 1990, to 782 000 (681 000–884 000) associated and 155 000 (122 000–188 000) attributable deaths in 2021. The pathogen with the greatest increase was *Staphylococcus aureus*, rising from 103 000 (73 900–133 000) attributable deaths in 1990 to 196 000 (177 000–215 000) attributable deaths in 2021. Only six pathogens had an attributable AMR burden of at least 100 000 in 2021; in descending order, they were: *S aureus, Acinetobacter baumannii, Escherichia coli, Klebsiella pneumoniae, S pneumoniae*, and *Pseudomonas aeruginosa*.

Among children younger than 5 years, the pathogens with the largest number of deaths attributable to AMR in 2021 were *K pneumoniae, S pneumoniae*, and *E coli* (figure 3). The greatest reduction in AMR deaths in children younger than 5 years globally was due to resistant *S pneumoniae*, which decreased from 158 000 attributable (95% UI 106 000–209 000) deaths and 801 000 associated (604 000–997 000) deaths in 1990, to 35 100 attributable deaths (24 000–46 200) and 176 000 associated (133 000–220 000) deaths in 2021. In contrast, drug-resistant *S pneumoniae* increased in people 5 years and older, rising from 100 000 (70 700–129 000) attributable and 496 000 (393 000–599 000) associated deaths in 1990, to 120 000 (96 200–144 000) attributable and 606 000 (533 000–679 000) associated deaths in 2021. In children younger than 5 years, deaths attributable to AMR decreased for all pathogens except tuberculosis between 1990 to 2021. Conversely, attributable AMR deaths in people 5 years and older increased in all but three pathogens: *Salmonella* Paratyphi*, Salmonella* Typhi, and *Serratia*. Among people 5 years and older, the pathogens with the largest number of deaths attributable to AMR in 2021 were *S aureus, A baumannii*, and *E coli.*

**Figure 5:**
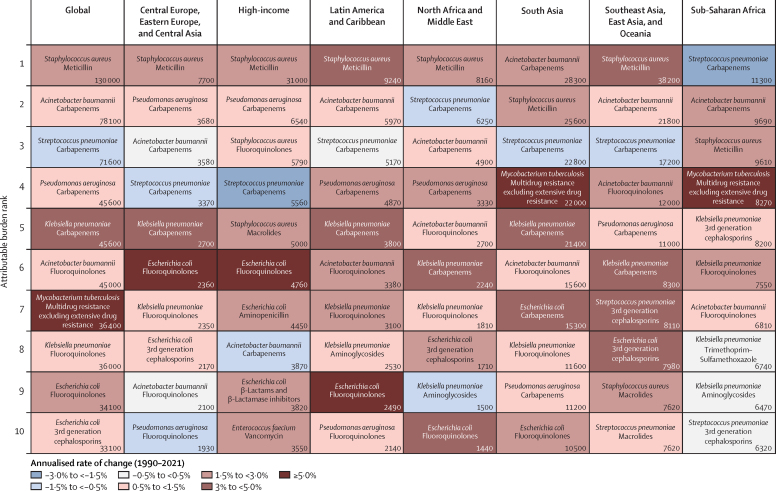
Ten deadliest pathogen–drug combinations, by burden attributable to AMR, global and super-region, in 2021 Cells coloured by annualised rate of change (1990–2021). Total deaths attributable to AMR in 2021 presented in the bottom right of each cell. AMR=antimicrobial resistance.

The pathogen–drug combination with the largest increase in attributable burden globally was meticillin-resistant *S aureus* (MRSA), doubling from 57 200 attributable (34 100–80 300) deaths in 1990 to 130 000 attributable (113 000–146 000) deaths in 2021 (figure 5). In Gram-negative organisms, annual mortality attributable to carbapenem resistance rose by 89 200 deaths (50 900–127 000) from 1990 to 2021, more than any antibiotic class over that period. There were four pathogen–drug combinations with an increase of more than 25 000 attributable annual deaths from 1990 to 2021: MRSA, multidrug-resistant tuberculosis, carbapenem-resistant *K pneumoniae*, and carbapenem-resistant *A baumannii* (appendix 1 pp 135–157). The pathogen–drug combination responsible for both the largest increase in attributable burden from 1990 to 2021 and the largest attributable burden in 2021 was MRSA in five super-regions (figure 5).

### AMR burden forecasts

**Figure 6:**
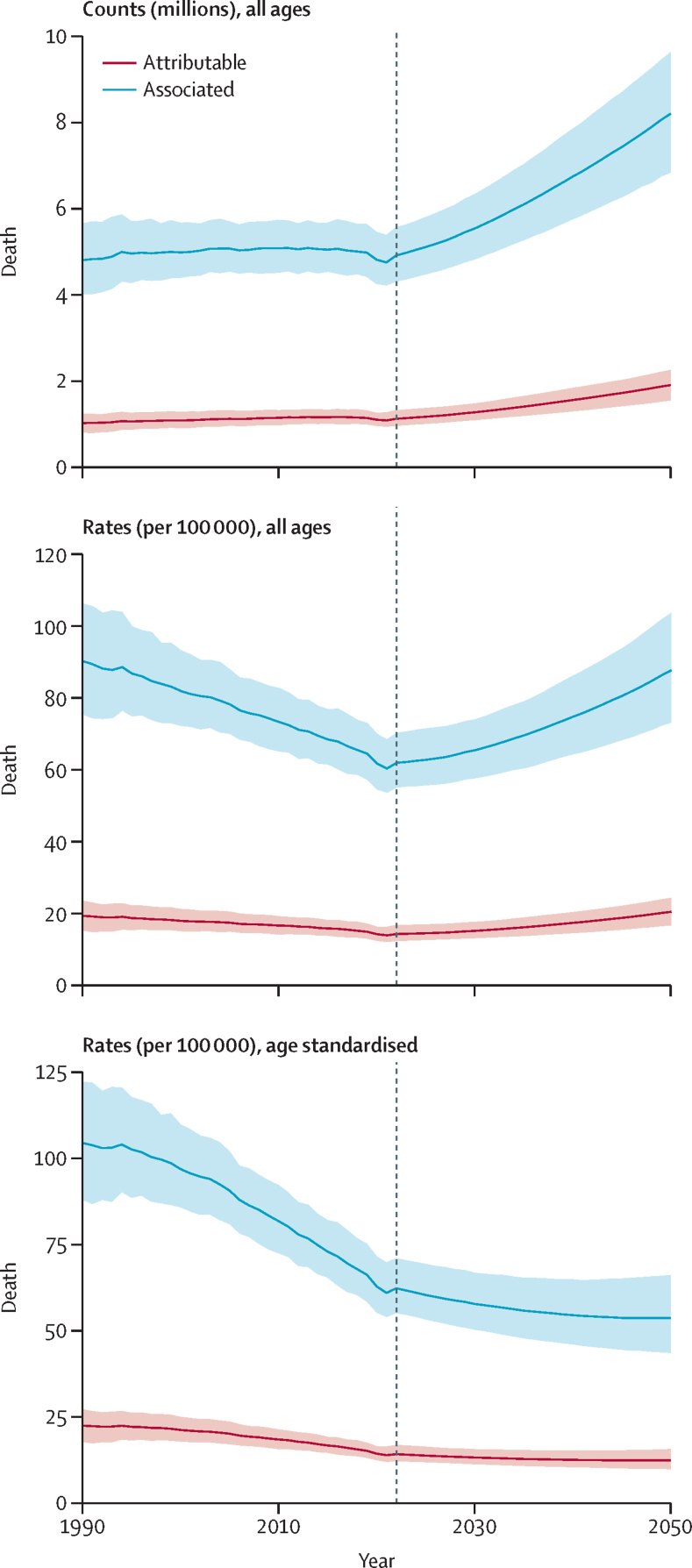
Global attributable and associated AMR burden in the reference scenario, 2022–2050 Shading represents the 95% uncertainty interval. The vertical line is placed at 2021 to distinguish estimates from forecasts.

We estimated that in 2050, there will be 1·91 million (95% UI [1·56–2·26]) annual deaths attributable to AMR globally and 8·22 million (6·85–9·65) annual deaths associated with AMR (table 3; figure 6). Cumulatively from 2025 to 2050, our reference scenario forecasts 39·1 million (33·0–46·0) deaths attributable to AMR and 169 million (145–196) deaths associated with AMR (appendix 1 pp 114–115). DALYs attributable to AMR are forecasted to increase from 42·6 million (35·5–52·5) in 2022 to 46·5 million (37·7–57·3) in 2050. The all-age rate of AMR attributable DALYs is forecasted to decrease, however, from 536 per 100 000 population (450–658) in 2022 to 496 per 100 000 (399–613) in 2050. Patterns in DALYs associated with AMR show a similar trend; with counts increasing from 2022 to 2050, and all-age rates decreasing over the same period (table 4).

The future AMR burden is highest in south Asia, southeast Asia, east Asia, and Oceania, and sub-Saharan Africa, with the forecasted cumulative AMR attributable death burden from 2025 to 2050 at 11·8 million (95% UI 9·43–14·4), 8·96 million (7·45–10·4), and 6·63 million (5·00–8·66), respectively. All-age rates of DALYs attributable to AMR are forecasted to be highest in south Asia in 2050 (687 DALYs per 100 000 [527–881]; table 4).

**Figure 7:**
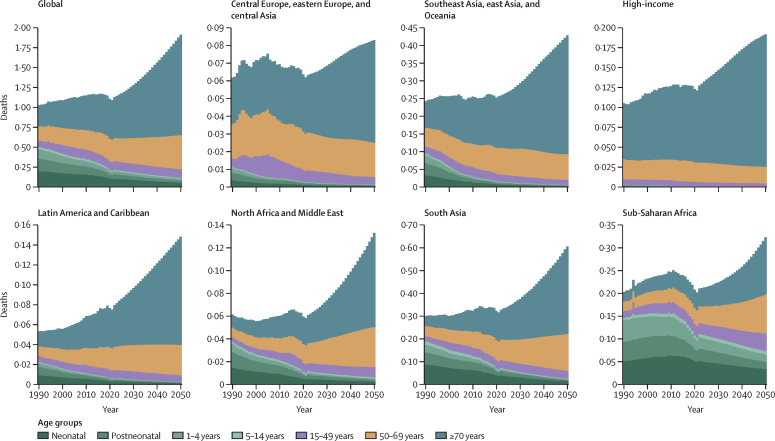
Deaths attributable to AMR by age group and location in the reference scenario, 2022–2050 Units are in millions.

The overall forecasted death numbers attributable to AMR conceal opposite trends by age. Globally and in every super-region, AMR deaths in children younger than 5 years are decreasing, most pronounced in absolute numbers in sub-Saharan Africa and south Asia. For deaths of people 70 years and older, we forecasted an increase from 2022 to 2050 in every super-region. The increase in people 70 years and older was 146% (95% UI 127–167) globally and ranged between 72·0% (61·5–84·1) in the high-income super-region and 234% (197–278) in the north Africa and the Middle East super-region (figure 7). In children younger than 5 years, the number of AMR attributable deaths decreased from 204 000 (150–285) to 103 000 (65·0–156) globally, a decrease of 49·6% (34·1–61·3). Among the super-regions, the decreases in children younger than 5 years ranged from 36·4% (16·4–51·9) in sub-Saharan Africa to 65·9% (55·1–74·9) in south Asia.

The global AMR all-age mortality rate is forecasted to increase from 14·2 per 100 000 (95% UI 12·3–16·7) in 2022 to 20·4 per 100 000 (16·6–24·2) in 2050, while the age-standardised mortality rate is forecasted to decrease from 14·2 per 100 000 (12·2–16·8) in 2022 to 12·4 per 100 000 (9·92–15·6) in 2050 (figure 6), indicating that the forecasted future trends of AMR burden will be largely driven by changes in population size and age structure.

By 2030, the global number of deaths attributable to AMR is forecasted to be 1·28 million (95% UI 1·10–1·48). From 2022 to 2030, the increases in the global number of AMR attributable deaths amount to a 13·4 % (7·8–18·8) increase by 2030, and a 69·6% (51·5–89·2) increase by 2050. In 2030, the all-age mortality rate attributable to AMR is forecasted to be 15·1 deaths (13·1–17·5) per 100 000, which is a 6·3% (0·8–11·8) increase from 2022.

In contrast to the strong increase in number of deaths due to AMR of close to 70% from 2022 to 2050, the number of DALYs showed a much smaller increase of 9·4% (–6·9 to 29·0) from 42·6 million (35·5 to 52·5) in 2022 to 46·5 million (37·7 to 57·3) in 2050.

### Impact of scenarios

**Figure 8:**
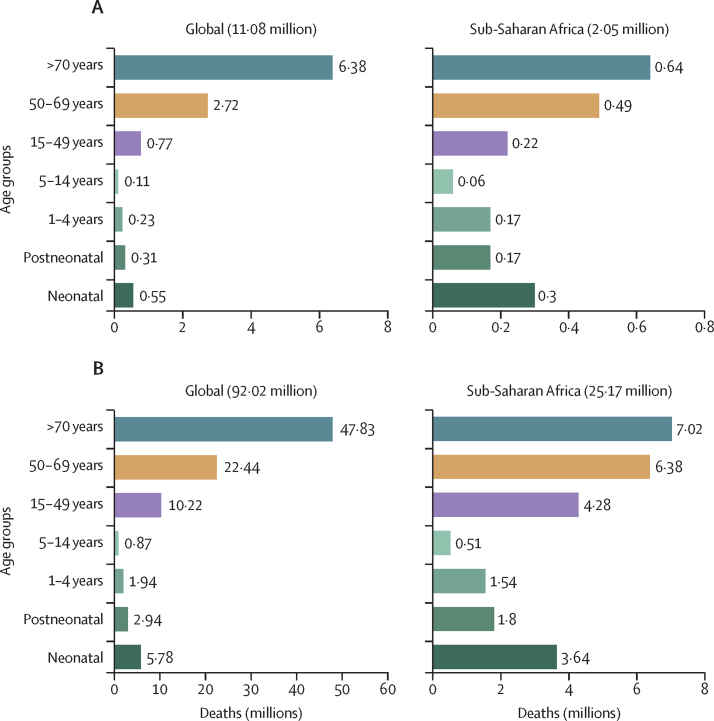
Cumulative deaths averted (in millions) in two different scenarios, by age, global versus sub-Saharan Africa, 2025–2050 (A) Gram-negative drug scenario. (B) Better care scenario.

In the better care scenario, we forecasted that a total of 92·0 million (95% UI 82·8–102) cumulative deaths (inclusive of deaths attrituable to or associated with AMR as well as AMR unrelated deaths) would be averted between 2025 and 2050 (appendix 1 pp 84, 116–117). The greatest benefits would be in the south Asia, sub-Saharan Africa, and southeast Asia, east Asia, and Oceania super-regions with 31·7 million (26·8–37·2), 25·2 million (21·2–29·8), and 18·7 million (14·4–22·8), respectively. Compared with the other GBD super-regions, sub-Saharan Africa was exceptional with a similar number of deaths averted in children younger than 5 years compared with those 70 years and older, with 6·98 million (5·25–8·98) deaths averted in children younger than 5 years versus 7·02 million (6·31–7·61) in the oldest age group (figure 8). The majority of deaths in children younger than 5 years in sub-Saharan Africa were in neonates with 3·64 million (2·94–4·48) deaths averted. In all other super-regions, the number of deaths averted for those 70 years and older far outweighed those in children younger than 5 years.

In the Gram-negative scenario, we forecasted that 11·1 million (95% UI 9·08–13·2) cumulative AMR deaths would be averted between 2025 and 2050 (appendix 1 pp 84, 116–117). The largest reductions would be in south Asia, south-east Asia, east Asia and Oceania, and sub-Saharan Africa, with averted AMR deaths of 3·97 million (3·10–4·88), 2·17 million (1·78–2·68), and 2·05 million (1·53–2·65), respectively. Deaths averted in children younger than 5 years will be largest in sub-Saharan Africa and south Asia, where 637 000 (409–950) and 309 000 (218–454) cumulative AMR deaths were forecasted to be averted, respectively. With the exception of sub-Saharan Africa, all other super-regions will see the majority of averted deaths among those older than 70 years and by far outweighing averted deaths in children younger than 5 years. In sub-Saharan Africa, close to one-third of averted deaths are forecasted both for in children younger than 5 years and those 70 years and older.

## Discussion

We found that, despite a decrease in global sepsis mortality, the global mortality from AMR increased slightly from 1990 to 2019, followed by a slight reduction during the COVID-19 pandemic. In our reference scenario, AMR burden is forecasted to increase to 1·91 million attributable deaths and 8·22 million associated deaths in 2050. This suggests that without additional measures, we will not reach the 10% reduction in AMR mortality proposed in the 10-20-30 by 2030 target.^3^ This study also found a notable divergence in AMR mortality trends by age, with children younger than 5 years having a greater than 50% reduction between 1990 and 2021, whereas adults had an increase in AMR mortality across this time period. We forecasted there will be continued reductions in AMR mortality in children younger than 5 years, but this improvement will be outpaced by the increase in AMR mortality in other age groups, particularly in older adults. Carbapenem resistance in Gram-negative bacteria saw a substantial increase in attributable burden from 1990 to 2021, and under the alternative scenario in which new antimicrobials are developed for Gram-negative bacteria, we describe a forecasted 11·1 million AMR deaths averted by 2050. In a second alternative scenario, with better health-care quality for infectious syndromes and improved access to antibiotics, we forecasted 92·0 million deaths averted by 2050.

The remarkable decline in AMR mortality in children younger than 5 years across the past three decades deserve special attention. Much of this reduction is due to a decrease in drug-resistant *S pneumoniae* and in pathogens commonly spread through faecal–oral transmission (eg, *Salmonella, Shigella*, and enteropathogenic or enterotoxigenic *E coli*). This decline coincides with widespread vaccination efforts and improved access to WASH^37^, ^38^ and serves as an important reminder to the global health community and policy makers that infection prevention might be a highly effective intervention in reducing AMR burden.^39^ These findings reinforce some of the major themes presented in the recent *Lancet* series on AMR,^40^ that preventing infections through existing measures can have an outsized effect on burden through multiple pathways.^39^ First, by preventing infection, there is no opportunity for resistance to affect health loss. Second, the prevention of even antibiotic-susceptible infections leads to a reduction in the number of people receiving antibiotics, which reduces the selection pressure for drug-resistant bacteria. Finally, by improving access to WASH, there is less opportunity for horizontal spread of resistant bacteria across a community. It should be noted, however, that while the overall AMR mortality is decreasing in children younger than 5 years, AMR has had an increasing role in sepsis deaths in this age group. We found that, in 2021, 31.3% of sepsis deaths in children younger than 5 years were associated with drug-resistant bacteria, a 5.18% increase relative to 1990. This suggests that despite the overall improvements in the number of AMR-related deaths, those children who develop sepsis are facing infections that are increasingly difficult to treat. Compounding this issue is the fact that many children are still dying due to a scarcity of access to adequate antibiotics for their infections.^41^, ^42^

The contrasting rise in AMR burden in adults is largely due to the increase in AMR deaths in those 50 years and older, with the greatest increase in those older than 70 years. In this age group, we found that AMR mortality increased by more than 80%. This is noteworthy for several reasons. First, the rising AMR and sepsis mortality in older adults has been masked by traditional global health metrics. For example, GBD and WHO global health estimates both adhere to the principles of the International Classification of Diseases and ascribe a death to a single underlying cause. As the population ages, more people suffer from chronic, non-communicable diseases that contribute to the development of sepsis; yet sepsis deaths among these populations are fully attributed to the underlying non-communicable disease (eg, cancer requiring long-term intravenous access for chemotherapy complicated by a line infection leading to bloodstream infection, sepsis, and death is attributed fully to cancer). Second, interventions that are effective in younger age groups, such as vaccines, might be less effective in older adults. For many vaccines, including the pneumococcal conjugate vaccine, immunosenescence leads to less robust antibody response and lower vaccine efficacy.^43^ This lower efficacy, as well as the lower vaccine coverage, might be why, despite having a pneumococcal vaccine available for older adults,^43^ there was not a decline in AMR burden due to *S pneumoniae*. Third, older adults are more likely to have adverse effects from specific antimicrobials;^44^ as resistance increases, the use of more toxic second-line and third-line treatment options is necessitated, which might not be tolerated in older populations. Fourth, adults are more likely to have comorbidities^45^, ^46^ that lead to immunodeficiencies and increase the risk of opportunistic infections.^47^ This is shown by the rapid increase in diabetes prevalence in older adults and the rise of Gram-negative infection burden.^48^ Finally, older adults are forecasted to be the demographic group that grows the most over the 21st century, with more than 25% of the world's population estimated to be older than 65 years by 2100.^49^ This could have a compounding effect on AMR—increasing the vulnerability to infection while increasing the volume of antimicrobials used—creating greater selection pressure for resistance to emerge.

Beyond the changes to population structure and population growth, discussed above as a driver of changing AMR burden, there were two additional drivers of change: improvement in the sepsis death rate and changes in the aetiological pathogens with increasing prevalence of resistance. Infection prevention and improvements in health-care quality and sepsis management prevented 3 090 000 AMR associated deaths from 1990 to 2019. This is overshadowed by the ageing and growing population, which contributed to an additional 2 530 000 AMR deaths, and changes in the pathogens and prevalence of resistance in pathogens causing sepsis, which led to 902 000 additional AMR deaths. Ultimately, the complex interplay between an older population, improvements in the health-care system, and the emergence of more resistant and deadly pathogens resulted in a net increase of 163 000 deaths associated with AMR from 1990 to 2019.

In 2024, WHO revised the Bacterial Priority Pathogens List^8^ to tackle the evolving challenges of AMR.^6^, ^50^, ^51^ In this most recent iteration, the critical priority designation was given to carbapenem-resistant *A baumannii*, carbapenem-resistant *Enterobacterales*, third-generation cephalosporin-resistant *Enterobacterales*, and rifampicin-resistant *M tuberculosis*.^8^ We estimated that the mortality attributable to all but one critical priority combination increased from 1990 to 2021, with third-generation cephalosporin-resistant *Enterobacterales* increasing from 1990 to 2019, followed by a decrease from 2019 to 2021. Although MRSA was the pathogen–drug combination with the greatest increase in attributable burden from 1990 to 2021, it is classified as a high priority instead of a critical priority.^8^ Part of this designation is due to the assignment of MRSA to high treatability and medium preventability. Indeed, there are several successful evidence-based interventions at the individual, facility, and population level that can reduce the risk of MRSA infection: (1) antimicrobial stewardship, which has been named an essential practice for MRSA infection and transmission prevention, (2) contact precautions for those colonised or infected with MRSA, and (3) MRSA screening and surveillance.^52^ Additionally, decolonisation protocols have been shown to reduce the risk of invasive MRSA infection in selected populations.^53^ Despite these prevention options, and its designation as highly treatable with several alternative antibiotics available, MRSA remains the pathogen–drug combination responsible for the greatest health loss globally in 2021. Our results argue for policies that ensure greater uptake of the already existing measures that have been proven effective at reducing MRSA burden, while continuing to invest in further research focused on prevention and treatment strategies.

In contrast to MRSA, there are fewer evidence-based prevention measures for carbapenem-resistant *Acinetobacter* and *Enterobacterales*, particularly at the population level. Many of the recommended prevention strategies are extrapolated from studies on MRSA^54^ or are facility-based control measures when a common source is identified.^55^ The uncertainty of the efficacy of these prevention strategies makes the substantial rise in carbapenem-resistant Gram-negative bacteria particularly alarming as carbapenems are considered by many to be a last-resort antibiotic, which is reflected in the WHO Bacterial Priority Pathogens List assignment of low treatability with potential future treatments determined to be unlikely. Most future treatments in the later stages of drug development are derivatives of established antimicrobial classes,^56^ and the development of resistance or cross-resistance already present has consistently proven problematic,^57^ with resistance often shown before an antibiotic is commercially released.^58^ Our estimates support the critical designation of carbapenem-resistant *Enterobacterales* and *Acinetobacter* as major causes of health loss globally that are rapidly increasing. Research into prevention measures is urgently needed to understand whether extrapolation of MRSA studies informing many prevention strategies in resistant gram-negative bacteria is reasonable and identify what additional measures can curb the rising mortality from these critical pathogens. Additionally, given the scarce treatment options once infection occurs, investment in drug development for these highly resistant Gram-negative bacteria is needed, while ensuring novel antibiotics are accessible to LMICs.^59^

We found heterogeneity in trends across super-regions and estimated that despite a decrease in AMR mortality over the study period, sub-Saharan Africa was the super-region with the highest mortality rate attributable to AMR in 2021. This reduction in mortality is due to a substantial decline in AMR deaths in children younger than 5 years, and is consistent with the principles described in the recent *Lancet* series, that AMR deaths can be averted with infection prevention and control measures, vaccination, and access to antibiotics.^60^ The persistently high AMR mortality rate in this super-region reflects many of the challenges that LMICs broadly face, which include fragmented health systems, inadequate intensive care units (ICU) facilities, overcrowded ICUs, staff shortages, inadequate universal access to WASH, few trained infection prevention and control personnel, underdeveloped microbiological capacity, and limited access to antibiotics.^61^ Continued investment in this infrastructure—as well as antibiotic stewardship and balancing antibiotic access with excess—are crucial and depend upon many factors, including financial incentives, greater affordability, and improved diagnostic capacity, as identification of the aetiological pathogen and susceptibility testing are crucial for appropriate antimicrobial prescribing.^56^ The effect of these interventions is highlighted by our forecasts that indicate the potential for 92·02 million averted deaths between 2022 and 2050 with improved health-care quality and access to effective antimicrobials.

However, there are challenges in implementing these evidence-based measures. A recent study showed that in 2023, 59·9% of countries that participated in the quadripartite self-assessment survey reported having a monitoring and evaluation plan in place for their National Action Plan on AMR—with only 11·3% of countries funding the implementation of their National Action Plans due to limited financial or human resources, insufficient capacity, and varying levels of political support.^62^ Even among the G7 countries, there are significant areas needing improvement, such as the adoption of WHO Access, Watch, Reserve classification of antimicrobials, full implementation of infection prevention and control measures, the integration of AMR surveillance to include food safety and animal health, as well as accounting for environmental residues of antibiotics and pesticide use.^62^ Correspondingly, it is crucial to continuously emphasise multisectoral engagement in the global fight against AMR. Accelerated investment in microbiology capacity is also needed to facilitate appropriate treatment, surveillance systems, and health metrics, and is essential to monitor progress towards future targets, such as the proposed 10% reduction in global AMR-related deaths.^63^

We found a decline in AMR burden during the COVID-19 pandemic within the years captured by our study (2020 and 2021). The relationship between COVID-19 and AMR is intricate with many mechanisms proposed to describe the relationship.^64^ The use of personal protective equipment, enhanced hand hygiene, and physical distancing (together with a greater number of focal points of infection prevention and control in LMICs) are salient factors that likely contributed to the temporary decrease in the transmission of resistant microorganisms while these measures were in effect.^65^ It is also possible that the pandemic could have substantially decreased AMR mortality by other mechanisms. Firstly, mortality displacement, or a harvesting effect, might have occurred in which older people and those with comorbidities died due to COVID-19 instead of bacterial infections.^66^ Secondly, there was evidence of fewer antibiotic prescriptions in high-income countries in 2020, which might have reduced selective pressure for resistance. Any optimism around the decreases in AMR mortality from 2019 to 2021 should be tempered as many of these effects might be temporary. As travel restrictions have eased, there is evidence that AMR has continued to spread in both LMICs and high-income countries, with LMICs being identified as being potential hotspots of resistant organisms.

Consistent with our previous research, we have produced AMR burden estimates for two counterfactuals that are both informative, particularly when considering the effect of AMR-directed interventions. When considering how vaccination campaigns can mitigate AMR burden, the no infection counterfactual is more informative as one could reasonably expect that those infections with resistant bacteria would be fully prevented. Whereas with new drug development, the infection is still present but now susceptible to the novel antibiotic, so the susceptible infection counterfactual could better describe the benefit.^67^

Our forecasting analysis suggests an increasing burden from AMR in the coming decades, with an estimated 69·6% increase in global deaths attributable to AMR and 67·0% increase in deaths associated with AMR between 2022 and 2050. Although all-age rates show increasing trends, age-standardised rates are forecasted to decrease. This indicates that many of the changes in AMR mortality in the future will be driven by changes in population size and age structure, with increased prevalence of comorbidities that are associated with infectious complications, such as obesity and diabetes. We see the interaction of the diverging trends in children younger than 5 years and older adults in the projected AMR burden to 2050. Although AMR deaths increase in numbers for all ages combined, they show strong declines across every GBD super-region in children younger than 5 years, but a more than a doubling globally and within five of the super-regions in individuals 70 years and older. The convergence of these population changes with increasing comorbidities is concerning, particularly in older adults, and will require interventions that can address the specific challenges in this age group and prepare health systems for the rising AMR burden in older adults.

Our reference forecasts indicate that the 10-20-30 by 2030 targets^68^ are unlikely to be met without added efforts on drug development, infection prevention, better treatment of severe infections, and better access to currently available antibiotics. To meet the target, the AMR mortality rate in 2030 would need to be less than 13·9 attributable AMR deaths per 100 000. This suggests that without additional investment in averting AMR mortality, we are on track to miss the 10% target with a difference of more than 150 000 attributable AMR deaths between the forecasted AMR deaths in 2030 and the 10% reduction target. Our findings from the Gram-negative drug scenario and the better care scenario show the opportunity for progress. Effective development and distribution of new Gram-negative drugs could result in 11·08 million (95% UI 9·08–13·17) AMR deaths averted from 2025 to 2050, accounting for approximately one-third of the cumulative deaths attributable to AMR that were forecasted under the reference scenario. Our better care scenario estimates a substantially larger impact above and beyond averting deaths caused by resistance with 92·02 million (82·81–101·75) cumulative deaths averted from 2025 to 2050 through efforts to improve the management of severe infections, and increase access to antibiotics, particularly in LMICs.

In reviewing the forecasting analyses presented in this study compared with those from the 2014 Review on Antimicrobial Resistance report,^1^ it is important to note that a direct comparison is not possible because the pathogen–drug combinations between the two studies do not perfectly overlap. The only combinations that both estimates include are MRSA, *E coli*, and *K pneumoniae* resistant to third-generation cephalosporins. The 2014 review result of 10 million deaths a year is somewhat larger than our estimate of nearly eight million associated deaths in 2050. However, our estimates include many more bacterial pathogen–drug combinations, whereas the AMR report includes malaria and HIV. Although we cannot explore the differences between the 2050 estimates, there is a clear similarity in the implications from both: AMR is an increasing global health challenge that requires urgent intervention.

The rising mortality due to carbapenem-resistant Gram-negative bacteria is cause for concern,^69^ with problematic phenotypes becoming increasingly common. The pharmaceutical industry has an important role in meeting this challenge with the development of therapeutic agents to overcome multidrug resistant infections. We estimated that the potential effect of new Gram-negative antibiotics in reducing mortality is profound, with a forecasted 11·08 million (95% UI 9·01–13·17) cumulative AMR deaths prevented between 2025 and 2050 in the new Gram-negative drug scenario; however, a total of 28·03 million (23·7–32·8) annual AMR deaths still occur by 2050, even under this scenario's optimistic assumptions. This underscores that drug development targeting the most difficult to treat infections, while important, is not sufficiently comprehensive to address the AMR challenge, as development of further resistance appears to be inevitable. There is a need for a diverse set of interventions to reduce the burden of AMR, including those that will prevent infections, provide better access and quality of care, and improvements in diagnostics and management of comorbid conditions. The number of deaths averted between 2025 and 2050 under the better care scenario is five-times greater than those saved by the Gram-negative drug scenario. This shows that overall improvements to health-care systems, including access to existing antibiotics, could be far more impactful than drug development alone. Of course, framing these scenarios as either drug development or improving health systems is a false dilemma, and there should be investment in both approaches; the more than 50 million deaths averted by improving access to existing antimicrobials is remarkable and supports the recent calls to add AMR and access to antimicrobials as a focus of the Global Fund's mission.^70^, ^71^

This study has several limitations (many of which have been discussed previously), with the most significant being the scarcity of data from some LMICs on the distribution of pathogens by infectious syndrome, the relative risk of resistance for key pathogen–drug combinations, and the number of deaths related to infections. In our study, seven of 204 countries and territories had no data available for any of our modelling components. We also acknowledge a substantial scarcity of data linking laboratory results to outcomes such as mortality, an issue also reflected in our previous research. Such sparsity is mainly due to insufficient laboratory infrastructure and capacity, scarce or absent microbiological facilities, weak health systems and governance and information systems, and scarce resources.^60^, ^72^, ^73^ In some countries, available data are fragmented, undersampled, or not representative of the broader population. Moreover, without accessible and well equipped microbiological facilities, data from medically underserved populations are often unobtainable. Consequently, there is at times a loss of accuracy regarding burden estimates for countries with scarce data and for specific pathogen–drug combinations, and resultantly exceptional difficulties in controlling for biases posed by patient comorbidities and microbiological testing procedures that is often unavailable in the data that we do have. Improving microbiological laboratory capacity is essential for improved patient outcomes. The scarce data in some countries substantially affected our prevalence of resistance and relative risk modelling components, akin to the situation in our previous publications.^6^, ^50^, ^51^, ^60^ We acknowledge that there might be methodological assumptions due to data scarcity, and this could potentially result in overestimation or underestimation of resistance prevalence (eg, pathogen–drug combinations with sparse data could be influenced by outliers in neighbouring country-years). Nevertheless, our overall estimation process remains both comprehensive and consistent, drawing on all available global data; more specifically, when specific country data are scarce, estimates rely on covariates and regional patterns and are supported by out-of-sample predictive validity assessments.

Second, despite our efforts to minimise and account for biases and misclassifications, these might arise when consolidating and standardising data from different providers and origins of infection. We also acknowledge the potential effect of selection bias in passive microbial surveillance data, bias introduced by low rates of diagnostic culture utilisation,^74^ while differences in antimicrobial usage between private and public health-care sectors might lead to incomplete representation of AMR patterns. In LMICs, hospital microbiology data might disproportionately reflect cases of more severe disease and urban populations. Furthermore, although we included data from tertiary care facilities and adjusted for bias in the prevalence of resistance, a large portion of our data originated from facilities with mixed tertiary, non-tertiary, or unknown classifications. Estimated relative risks of death and increases in length of stay associated with resistance are also vulnerable to residual confounding (eg, unreported patient primary diagnoses affecting both vulnerability to infection and risk of death), collider stratification bias (eg, hospitalisation as a collider caused by exposure to infection with resistant bacteria and the outcome of being ill enough to die), and time-varying confounding (eg, treatment and dose). We are also cognisant that coinfections and comorbidities can amplify the risk, severity, and outcomes of resistant infections, and thus additional research is necessary to elucidate their role and specific effects. In addition, evolving diagnostic techniques could introduce some variability in the results, especially when comparing data across different time periods. These limitations underscore the need for standardised protocols and improved data harmonisation efforts to ensure the robustness and reliability of AMR burden analyses.

Our study also faces unique challenges specific to time trend analysis of AMR. One constraint is the uneven temporal distribution of available data from various regions and countries, which complicates the accurate representation of global, regional, and national AMR dynamics—potentially affecting the reliability and interpretation of our time trend analyses. Data reporting levels of resistance before 2000 is exceptionally sparse: in Europe, widespread collaborative monitoring of AMR did not begin until 1998 with the formation of the European Antimicrobial Resistance Surveillance System, in Latin America monitoring began for a select few pathogen–drug combinations in the mid-1990s through the Regional System for Vaccines, and the Latin American Antimicrobial Resistance Surveillance Network, and in the USA, Centers for Disease Control and Prevention-directed monitoring began in 1996. Correspondingly, our estimates of resistance before 2000 rely heavily on modelled temporal trends and covariates. We are also subject to a lack of longitudinal data (both on AMR prevalence patterns and antimicrobial usage) from many LMICs and the sporadic and non-standardised nature of data collection across different regions and health-care settings that further hampers our ability to track changes in pathogen–drug resistance profiles over time. Finally, the COVID-19 pandemic substantially affected all facets of health systems, including AMR surveillance efforts. The reduction in the number of microbiological cultures, elective procedures, and outpatient visits, alongside an increase in ICU admissions and admissions of chronically ill patients, could skew and subsequently bias AMR data used in our study.^65^, ^75^

The high burden of β-lactam resistance, and particularly carbapenem resistance, in *S pneumoniae* deserves further comment. To have a consistent approach across pathogens, we use a β-lactam hierarchy such that an isolate that is resistant to both third-generation cephalosporins and carbapenems has its attributable burden assigned to carbapenems (appendix 1 p 69). This is a stronger assumption for *S pneumoniae* than for the Gram-negative bacteria because the mechanism of resistance in Gram-negative bacteria is more commonly β-lactamases^76^ that often follow this hierarchy. By contrast, β-lactam resistance in *S pneumoniae* is often due to mutations^77^ in the penicillin binding protein; a possible effect of applying this hierarchy to *S pneumoniae* is an overestimation of the burden of carbapenem resistance in *S pneumoniae* relative to other β-lactams, specifically penicillin and third-generation cephalosporins. Additionally, in our framework, the effect of resistance on mortality (ie, the relative risk) borrows strength from other pathogens with resistance to the same antimicrobial class, which is likely a stronger assumption for *S pneumoniae* than the Enterobacterales given the prevalence of input data from Carbapenemase producing carbapenem-resistant enterobacterales and *S pneumoniae*'s distinct mechanism of resistance. We hope that with continued data seeking, future iterations will include models more specific to the nuances of *S pneumoniae*.

Our forecast and scenarios of AMR burden to 2050 are also subject to the limitations in the historical estimates, including data availability and quality, as our forecast trends are built of historical estimates. The forecast models were also produced from estimates of deaths due to resistance overall, rather than forecasting individual pathogens, which could lead to reduced accuracy if there is substantial heterogeneity across pathogen time trends. Our forecast models also do not include the outbreak of new resistant pathogens or superbugs, and might be under-estimates if new pathogens arise or stochastic events occur that lead to a marked increase in infections, and thus resistance. We also made several assumptions in the development of our Gram-negative drug scenario and better care scenario, and thus the effect of these findings is informed by those assumptions. First, we assume that the proportion of AMR infections due to Gram-negative bacteria will remain constant over time and that the development of novel therapeutics and scale-up of a drug pipeline would take 15 years and be effective in reducing Gram-negative deaths by 50%. In our better care scenario, we reduce cause-specific mortality from infectious syndromes to levels of countries at the 85th percentile of the HAQ Index (in the lower tail of HAQ for high-income countries). This scenario captures improvements in the health system beyond better care for severe infectious diseases and improved access to antibiotics, such as improved diagnostic capacity, a more robust health-care workforce, and availability of life-saving technological interventions for people with advanced disease (eg, ventilators). Our forecasts for the better care scenario do not incorporate the effects infectious diseases outside of those that contributed to the AMR mortality envelope (appendix 1 p 20). Due to difficulty calculating the 85th percentile case-fatality rate, typhoid, paratyphoid, and invasive non-typhoidal *Salmonella* was excluded. This scenario additionally assumes adequate stewardship should access to antibiotics improve, which might depend on other factors associated with a high HAQ, such as income and other features of general development.

Despite these limitations, to our knowledge, this study represents the most comprehensive analysis of bacterial AMR burden across time, and the most extensive analysis forecasting the burden of AMR to 2050. It reflects the best and widest range of available data and used models that have been tested and refined over the 30 years of GBD analysis to incorporate diverse data sources. Although each data source alone does not fully address the temporal pattern of AMR burden, their collective use provides a more complete estimate with comprehensive geographical coverage. Therefore, to our knowledge, this study is the first to report changes in AMR burden over time by using attributable and associated counterfactual scenarios while covering a wide array of pathogens and pathogen–drug combinations, with global and regional estimates for 204 countries and territories. With future iterations of this work, we intend to monitor progress towards the 10-20-30 by 2030 target. We also anticipate expanding the scope of pathogen–drug combinations, given the importance of other antimicrobial classes, such as tetracyclines, and the effect of current resistance among fungi, viruses, and parasites. We can also build on this infrastructure to describe the burden of emerging resistant pathogens such as *Candida auris*. As surveillance systems improve with greater data availability and geographic coverage, these estimates will be increasingly precise and could be used to inform WHO empiric antimicrobial recommendations by region.

We found that AMR deaths for both counterfactuals increased slightly from 1990 to 2019, while the all-age mortality rate decreased. Much of this reduction is due to infection prevention, improvements in the management of sepsis, and reductions in sepsis mortality in children younger than 5 years. When looking at the role of AMR in sepsis deaths, we describe a growing and concerning trend that when someone dies from sepsis, the probability that the organism causing infection is drug-resistant is 25% higher in 2019 relative to 1990. Perhaps more importantly, we found rising resistance to critically important antimicrobials. This is most stark with the increase in the burden of carbapenem-resistant Gram-negative organisms but is also reflected in multidrug-resistant tuberculosis. All but one of the pathogen–drug combinations rated by the WHO Bacterial Priority Pathogens List to have low or medium-low treatability had an increasing burden from 1990 to 2021; only fluoroquinolone-resistant *Salmonella* Typhi had a decreasing burden, largely due to decreases in global *Salmonella* Typhi incidence. Our reference scenario mortality estimates suggest a continued increase in AMR mortality, leading to 1·91 million attributable deaths and 8·22 million associated AMR deaths in 2050. Our reference forecasts suggest that without additional measures we will fail to hit the 10% reduction in AMR mortality proposed in the 10-20-30 by 2030 target.

### GBD 2021 Antimicrobial Resistance Collaborators

### Affiliations

### Contributors

### Data sharing

This study follows the Guidelines for Accurate and Transparent Health Estimates Reporting. To download the data used in these analyses, please visit the Global Health Data Exchange at https://ghdx.healthdata.org/record/ihme-data/gbd-2021-bacterial-amr-estimates-forecasts-1990-2050.

## Declaration of interests

U Abubaker reports leadership or fiduciary role in other board, society, committee or advocacy group, unpaid, with the Early Career Pharmaceutical Group of the International Pharmaceutical Federation between January 2021 – December 2023, outside the submitted work. S Afzal reports payment or honoraria for educational events and Webinars with King Edward Medical University and collaborative partners including University of Johns Hopkins, University of California, and University of Massachusetts; participation on a Data Safety Monitoring Board or Advisory Board with National Bioethics Committee Pakistan, King Edward Medical University Institutional Ethical Review Board, and Ethical Review Board Fatima Jinnah Medical University and Sir Ganga Ram Hospital; leadership or fiduciary role in other board, society, committee or advocacy group, paid or unpaid, with Pakistan Association of Medical Editors, Faculty of Public Health Royal Colleges UK (FFPH), with Society of Prevention, Advocacy And Research, King Edward Medical University. (SPARK), and the Pakistan Society of Infectious Diseases; other financial or non-financial interests as Dean of Public Health and Preventive Medicine King Edward Medical University, Chief Editor Annals of King Edward Medical University since 2014, Director Quality Enhancement Cell King Edward Medical University, and Member Research and Publications Higher Education Commission Pakistan; all outside the submitted work. R Ancuceanu reports consulting fees from Abbvie; payment or honoraria for lectures, presentations, speakers bureaus, manuscript writing or educational events from Abbvie, Laropharm, Reckitt, and Merck Romania; support for attending meetings and/or travel from Merck Romania; all outside the submitted work. J R Andrews reports support for the present manuscript from Bill and Melinda Gates Foundation (funding for the Surveillance for Enteric Fever in Asia Project Study); grants or contracts from NIH for typhoid and tuberculosis studies; royalties from UpToDate on typhoid clinical management; outside the submitted work. D T Araki reports support for their participation in the present manuscript through their employment at the Institute for Health Metrics and Evaluation (IHME), and support for attending meetings and/or travel from IHME outside the submitted work. A Beloukas reports grants or contracts from Gilead and GSK/ViiV through their institution; payment or honoraria for lectures, presentations, speakers bureaus, manuscript writing or educational events from Gilead and GSK/ViiV through their institution; support for attending meetings and/or travel from Gilead and GSK/ViiV through their institution; Receipt of FOC reagents from Cepehid; all outside the submitted work. J A Berkley reports support for the present manuscript from the Bill & Melinda Gates Foundation through payments to their university. S Bhaskar reports grants or contracts from Japan Society for the Promotion of Science (JSPS), Japanese Ministry of Education, Culture, Sports, Science and Technology (MEXT), Grant-in-Aid for Scientific Research (KAKENHI) (P23712), JSPS and the Australian Academy of Science, and JSPS International Fellowship (P23712); leadership or fiduciary role in other board, society, committee or advocacy group, paid or unpaid, with Rotary District 9675, Sydney, Australia, Global Health & Migration Hub Community, Global Health Hub Germany, Berlin, Germany, PLOS One, BMC Neurology, Frontiers in Neurology, Frontiers in Stroke, Frontiers in Public Health, Journal of Aging Research & BMC Medical Research Methodology, College of Reviewers, Canadian Institutes of Health Research (CIHR), Government of Canada, World Headache Society, Bengaluru, India, Cariplo Foundation, Milan, Italy, National Cerebral and Cardiovascular Center, Department of Neurology, Suita, Osaka, Japan, and Cardiff University Biobank, Cardiff, UK; all outside the submitted work. C S Brown reports other financial or non-financial interests through participation in short-term, anonymous, and indirect market research with companies Sermo, Atheneum, and M3, all outside the submitted work. E Chung reports support for the present manuscript in part by the National Institutes of Health (NICHD T32HD007233 to EC). S J Dunachie reports support for the present manuscript from UK Fleming Fund at Department of Health and Social Care, Bill & Melinda Gates Foundation, and Wellcome Trust through their employment, and from the UK National Institute of Health and Care Research through a Global Research Professorship (NIHR300791); grants or contracts from UKRI (MR/W02067X/1 and MR/W020653/1), US Defense Threat Reduction Agency, Wellcome Drug Resistant Infections Discretionary Award, and the UK Dept of Health & Social Care; consulting fees from the Scottish Parliament and Wellcome; participation on the Data Monitoring Committee for UK STABILISE study of BCG vaccine in COPD; leadership or fiduciary role in other board, society, committee or advocacy group, paid or unpaid, as a member of New and Emerging Respiratory Virus Threats Advisory Group (NERVTAG), Chair of Wellcome SEDRIC subgroup on data standards and harmonisation in antimicrobial resistance, UK, member of Variant Technical Group for SARS-CoV-2 for UK Health Security Agency, UK, Expert advisor to WHO's Global Antimicrobial Resistance Surveillance System (GLASS), Geneva, Switzerland, and a Member of World Health Organization Guidelines Development Group on Treatment of Ebola, Geneva, Switzerland; all outside the submitted work. T Eckmanns leadership or fiduciary role in other board, society, committee or advocacy group, unpaid, with a speaker working group infection control German society of infection control and microbiology. N A Feasey reports grants from Wellcome Programme Grant: ACORN, and Wellcome Programme Grant: ADILA through payments to their institute, outside the submitted work. I M Ilic reports support for their participation in the current manuscript from Ministry of Education, Science and Technological development, Republic of Serbia, project No 175042, 2011-2023. N E Ismail reports leadership or fiduciary role in other board, society, committee or advocacy group, unpaid, as Bursar and Council Member of the Malaysian Academy of Pharmacy and as a Committee Member of Education Chapter, Malaysian Pharmacists Society; all outside the submitted work. T Joo reports support for their participation in the current manuscript from EU under EU4HEALTH programme (AMR-EDUcare – EduCation on Antimicrobial REsistance for the health workforce). M Lee reports support for their participation in the current manuscript from the Ministry of Education of the Republic of Korea and the National Research Foundation of Korea (NRF-2023S1A3A2A05095298). L Monasta reports support for their participation in the current manuscript from the Italian Ministry of Health (Ricerca Corrente 34/2017) through payments made to the Institute for Maternal and Child Health IRCCS Burlo Garofolo. C E Moore reports participation on a Data Safety Monitoring Board or Advisory Board as a Member of Advisory board for MRC grant, Advisory group for WHO Medically Important Antimicrobial List, Member of the Steering group for the REVIVE study , Member of Advisory board for CABBAG, and as a Member of Advisory board for RADAAR; Leadership or fiduciary role, unpaid, in a microbiology society as Co-chair of Impact and Influence group of Knocking Out AMR project; all outside the submitted work. A J Pollard reports grants or contracts from Gates Foundation, Wellcome, Cepi, MRC, NIHR, AstraZeneca, EC, and the Serum Institute of India, all as payments to their institution; royalties or licenses with AstraZeneca through their institution; consulting fees from Shionogi; leadership or fiduciary role in other board, society, committee or advocacy group, unpaid, as Chair of DHSC's Joint Committee on Vaccination and Immunisation, a Member of WHOs SAGE until 2022, and Chair of WHOs Salmonella TAG; receipt of equipment, materials, drugs, medical writing, gifts or other services from Moderna; all outside the submitted work. L F Reyes reports grants or contracts from GSK, MSD, and Pfizer; consulting fees from GSK, MSD, and Pfizer; payment or honoraria for lectures, presentations, speakers bureaus, manuscript writing or educational events from GSK, MSD, and Pfizer; payment for expert testimony from GSK, MSD, and Pfizer; support for attending meetings and/or travel from GSK and Pfizer; stock or stock options in GSK; all outside the submitted work. Y L Samodra reports grants or contracts from Taipei Medical University; a leadership or fiduciary role in other board, society, committee or advocacy group, paid or unpaid, with the Benang Merah Research Center, Indonesia; and other financial or non-financial interests in bertakon.com as founder; all outside the submitted work. E A F Simões reports grants or contracts from Astra Zeneca Inc, Merck & Co., Pfizer Inc, Icosavax Inc, Johnson and Johnson, and Enanta Pharmaceuticals; consulting fees from Merck & Co., Pfizer Inc, Sanofi Pasteur, Cidara Therapeutics, Icosavax Inc, Nuance Pharmaceuticals, GSK, Enanta, and Gilead; payment or honoraria for lectures, presentations, speakers bureaus, manuscript writing or educational events from Pfizer Inc and Astra Zeneca; support for attending meetings and/or travel from Astra Zeneca, Sanofi, and Pfizer Inc; participation on a Data Safety Monitoring Board or Advisory Board with AbbVie Inc, GlaxoSmithKline plc, and Moderna Inc; all through their institution and outside the submitted work. P Turner reports grants or contracts from the Wellcome Trust through the ACORN AMR surveillance network project (University of Oxford); support for attending meetings and/or travel from the World Health Organization for attendance at the AMR Diagnostic Initiative Meeting (July 2023); all outside the submitted work. P C M Williams reports grants or contracts from National Health and Medical Research Council administered by the University of Sydney; payment or honoraria for lectures, presentations, speakers bureaus, manuscript writing or educational events ECCMID & WSPID for conferences between 2022 and 2024; support for attending meetings and/or travel from ECCMID & WSPID; leadership or fiduciary role in other board, society, committee or advocacy group, paid or unpaid, with the World Society of Paediatric Infectious Diseases and the Australasian and New Zealand Paediatric Infectious Diseases Society; all outside the submitted work. G Zamagni reports support for their participation in the present manuscript from the Italian Ministry of Health (Ricerca Corrente 34/2017), payments made to the Institute for Maternal and Child Health IRCCS Burlo Garofolo. M Zielińska other financial or non-financial interests in AstraZeneca as an employee, outside the submitted work. A Zumla reports support for their participation in the current manuscript from the Pan-African Network on Emerging and Re-Emerging Infections (PANDORA-ID-NET) funded by the EDCTP - the EU Horizon 2020 Framework Programme, the UK NIHR, a Mahathir Science Award, and EU-EDCTP Pascoal Mocumbi Prize; participation on a Data Safety Monitoring Board or Advisory Board, unpaid, with the WHO Infection Prevention and Control Committee; leadership or fiduciary role in other board, society, committee or advocacy group, unpaid, with the University of Bolton School of Medicine; outside the submitted work.
