# Supplementary material for: Global burden of bacterial antimicrobial resistance 1990–2021: a systematic analysis with forecasts to 2050
Source: Lancet. 2024 Sep 28;404(10459):1199–226. doi: 10.1016/S0140-6736(24)01867-1 (PMC11718157; doi:10.1016/S0140-6736(24)01867-1)
Supplement: Supplementary appendix 1 [file mmc1.pdf]

THE LANCET

Supplementary appendix 1

This appendix formed part of the original submission and has been peer reviewed. We post it as supplied by the authors.

Supplement to: GBD 2021 Antimicrobial Resistance Collaborators. Global burden of bacterial antimicrobial resistance 1990–2021: a systematic analysis with forecasts to 2050. *Lancet* 2024; published online Sept 16. https://doi.org/10.1016/S0140-6736(24)01867-1.

Appendix 1: supplementary methods and results for "Global burden of bacterial antimicrobial resistance 1990–2021: a systematic analysis with forecasts to 2050"

This appendix provides further methodological details and supplementary results for "Global burden of bacterial antimicrobial resistance 1990–2021: a systematic analysis with forecasts to 2050". Portions of this appendix have been reproduced or adapted from the appendix of the paper "Global burden of bacterial antimicrobial resistance in 2019: a systematic analysis".[1] References are provided for reproduced sections.

Table of Contents

Section 1: List of abbreviations 5

Section 2: Data sources[1] 7

Section 2.1: Multiple causes of death and vital registration (MCoD-VR) 7

Section 2.2: Hospital discharge 7

Section 2.3: Microbial data with outcome 8

Section 2.4: Microbial data without outcome 9

Section 2.5: Literature studies 12

Section 2.5.1: Maternal sepsis, neonatal sepsis, and LRI aetiology 12

Section 2.5.2: Urinary tract infections aetiology 13

Section 2.5.3: Skin infections aetiology 13

Section 2.5.4: Intra-abdominal infection aetiology 13

Section 2.5.5: Bone and joint infections aetiology 13

Section 2.5.6: Meningitis infection aetiology 13

Section 2.5.7: Relative risk studies for specific drug-bug combinations 13

Section 2.5.8: Prevalence of resistance for specific organisms 14

Section 2.6: Single drug-resistance profiles 15

Section 2.7: Pharmaceutical sales and antibiotic use 16

Section 2.8: Mortality surveillance 16

Section 2.9: Linkage (mortality only) 16

Section 3: Summary of GBD 2021 estimation process[1] 16

Section 3.1: GBD 2021 cause of death estimation process 16

Section 3.2: GBD 2021 non-fatal estimation process 17

Section 4: Sepsis and infectious syndrome estimation[1] 17

Section 4.1: Input data 17

Section 4.1.1: Multiple causes of death 17

Section 4.1.2: Hospital record with multiple diagnoses and discharge status of death 17

Section 4.1.3: Linkage data 17

Section 4.1.4: Mortality surveillance (Child Health and Mortality Prevention Surveillance [CHAMPS]) 17

Section 4.1.5: Claims data with multiple diagnoses and fatal outcome 18

Section 4.2: Data processing 20

Section 4.3: Mapping the data 20

Section 4.4: Intermediate cause and infectious syndrome mapping hierarchy 20

Section 4.4.1: Intermediate cause mapping 20

Section 4.4.2: Informative ranking 21

Section 4.4.3: Two modelling pathways 22

Section 4.5: First pathway: Estimating the envelope of sepsis 22

Section 4.5.1: Sepsis model 22
Section 4.6: Second pathway: Apportioning sepsis to specific infectious syndromes 24
Section 4.6.1: Aggregation to the sepsis mortality envelope 27
Section 4.6.2: Infectious syndromes using GBD 2021 results 27
Section 4.7: Model validation 27
Section 4.7.1: Incidence of infectious syndromes disaggregated by age, sex, location, and year 32
Section 5: Case fatality ratios[1] 32
Section 5.1: Input data 32
Section 5.2: Data processing 32
Section 5.3: Modelling overview 32
Section 5.4: Modelling framework 33
Section 5.5: Predictions and uncertainty 35
Section 6: Pathogen distribution[1] 36
Section 6.1: Input data 36
Section 6.2: Data processing 36
Section 6.2.1: Extraction and standardisation 36
Section 6.2.2: Assigning infectious syndrome 36
Section 6.2.3: Selecting pathogens for estimation 37
Section 6.2.4: Pathogen redistribution and aggregate pathogen categories 37
Section 6.2.5: Contaminants, opportunistic pathogens, and no aetiology detected 40
Section 6.2.6: Polymicrobial infections 40
Section 6.2.7: Estimating unbiased other categories 41
Section 6.2.8: Age-sex splitting 42
Section 6.2.9: Standardising measures 42
Section 6.3: Modelling framework 43
Section 6.3.1: Overview 43
Section 6.3.2: Model priors 46
Section 6.3.3: Outliering 48
Section 6.3.4: Redistribution of "other pathogens" 48
Section 6.4: Exceptions and special handling 48
Section 6.4.1: Diarrhoea 49
Section 6.4.2: Lower Respiratory Infections 49
Section 6.4.3: Infectious syndromes not modelled 49
Section 6.5: Model validation 50
Section 7: Prevalence of resistance[1] 50
Section 7.1: Input data 50
Section 7.1.1: Prevalence of Resistance in Mycobacterium tuberculosis 51

Section 7.2: Data processing 51
Section 7.2.1: Resistance Breakpoint Processing..52
Section 7.2.2: Intrinsic Resistance Processing..53
Section 7.2.3: Tertiary Care Facility Adjustments...53
Section 7.3: Modelling framework..55
Section 7.4: Covariates...56
Section 7.5: Resistance profiles...56
Section 7.6: Model validation 58
Section 8: Relative risk[1]..60
Section 8.1: Input data...60
Section 8.2: Data processing 61
Section 8.3: Modelling overview 61
Section 8.3.1: Fatal modelling 61
Section 8.3.2: Nonfatal Modelling...62
Section 8.4: Model validation 63
Section 9: Counterfactuals and AMR estimation[1] 65
Section 9.1: Estimating associated AMR burden with counterfactual of no infection 65
Section 9.2: Estimating attributable AMR burden with counterfactual of infection with susceptible organism.....68
Section 9.3: Excluded combinations 70
Section 9.4: Decomposition of factors contributing to change in AMR associated deaths 70
Section 9.4.1: Decomposition Methods Overview..71
Section 9.4.2: Decomposition results..71
Section 10: Forecasting AMR...73
Section 10.1: Future health scenarios platform overview 73
Section 10.2: Forecasting AMR population attributable fractions 73
Section 10.3: Computing future attributable and associated AMR burden 73
Section 10.4: Developing AMR alternative scenarios..74
Section 10.4.1: Gram-Negative Drug scenario 74
Section 10.4.2: Better Care scenario 74
Section 11: GATHER compliance 74
Section 12: References 75
Section 13: Appendix tables and figures...80

Section 1: List of abbreviations

Abbreviation	Full phrase
AGAR	Australian Group on Antimicrobial Resistance
AHC	Angkor Hospital for Children
AMASS	AutoMated tool for Antimicrobial resistance Surveillance System
AMR	antimicrobial resistance
APUA	Alliance for the Prudent Use of Antibiotics
ARSP	Antimicrobial Resistance Surveillance Program
ATLAS	Antimicrobial Testing Leadership and Surveillance
AUC	area under the receiver operating characteristics curve
AURA	Antimicrobial Use and Resistance in Australia
AWARE	Assessing Worldwide Antimicrobial Resistance Evaluation
BARNARDS	Burden of Antibiotic Resistance in Neonates from Developing Societies
BD	Becton, Dickinson, and Company
BSI	bloodstream infections
CAESAR	Central Asian and European Surveillance of Antimicrobial Resistance
CAI	community-acquired infection
CDC	Centers for Disease Control and Prevention
CFR	case fatality ratio
CHAIN	Childhood Acute Illness and Nutrition
CHAMPS	Child Health and Mortality Prevention Surveillance
cIAI	complicated intra-abdominal infection
CLSI	Clinical and Laboratory Standards Institute
COMRU	Cambodia Oxford Medical Research Unit
CODEm	cause of death ensemble model
CTMRF	CHILDS Trust Medical Research Foundation
cUTI	complicated urinary tract infection
DALYs	disability-adjusted life-years
DHS	Demographic and Health Surveys
EARS-Net	European Antimicrobial Resistance Surveillance Network
ECDC	European Centre for Disease Prevention and Control
EUCAST	European committee on antimicrobial susceptibility testing
GAM	generalised additive models
GBD	Global Burden of Diseases, Injuries, and Risk Factors Study
GBS	group B Streptococcus
GLASS	Global Antimicrobial Resistance Surveillance System
GLM	generalised linear model
GPR	Gaussian process regression
HAI	hospital-acquired infection

HAQ Index	Healthcare Access and Quality Index
HHS	U.S. Department of Health and Human Services
ICD	International Classification of Diseases
ICU	intensive care unit
INFORM	International Network for Optimal Resistance Monitoring
INICC	International Nosocomial Infection Control Consortium
iNTS	invasive non-typhoidal Salmonella
IORD	Infections in Oxfordshire Research Database
IQVIA	IMS Health and Quintiles
JANIS	Japan Nosocomial Infections Surveillance
KEMRI	Kenya Medical Research Institute
LRI	lower respiratory infection
MAE	mean average error
MCoD	multiple causes of death data
MEPCO	multinomial estimation of partial and composite observations
MICS	Multiple Indicators Cluster Surveys
MITS	minimally invasive tissue sampling
MMO	Myelitis, meningoencephalitis and encephalitis
MR-BRT	meta-regression—Bayesian, regularised, trimmed
MRC	Medical Research Council
NARMS	National Antimicrobial Resistance Monitoring System for Enteric Bacteria
NICD	National Institute for Communicable Diseases
OUCRU	Oxford University Clinical Research Unit
PPS HAI	Point Prevalence Survey on Nosocomial Infections and Antibiotic Use
PRISMA	Preferred Reporting Items for Systematic Reviews and Meta-Analyses
ROC	receiver operating characteristics
RMSE	Root mean square error
SDI	Socio-demographic Index
SEV	summary exposure value
SGUL-GARPEC	St. George's Hospital, University of London - Global Antimicrobial Resistance, Prescribing and Efficacy Among Neonates and Children
SOAR	Survey on Antibiotic Resistance
ST-GPR	spatiotemporal Gaussian process regression
TB	tuberculosis
TESSy	The European Surveillance System
TEST	Tigecycline Evaluation Surveillance Trial
TSAP	Typhoid Fever Surveillance in Africa Program
UI	uncertainty interval
UPCH	Cayetano Heredia University

USDA	U.S. Department of Agriculture
UTI	urinary tract infection
VR	vital registration
WHO	World Health Organization
WRP	Walter Reed Project
YLDs	years lived with disability
YLLs	years of life lost

Section 2: Data sources[1]

The data used for this study can be categorised into the following types: multiple causes of death (MCoD), hospital discharge, linkage, mortality surveillance, literature reviews, microbial, single drug-resistance profiles, pharmaceutical sales, and antibiotic use data; as well as estimates from the Global Burden of Diseases, Injuries, and Risk Factors Study (GBD) 2021.[2] More detailed information on data inputs are available at https://ghdx.healthdata.org/record/ihme-data/gbd-2021-bacterial-amr-estimates-forecasts-1990-2050.

Section 2.1: Multiple causes of death and vital registration (MCoD-VR)

Multiple cause of death (MCoD) data is a type of vital registration obtained from death certificates that contain the underlying cause of death, intermediate and immediate causes of death, and contributing conditions. MCoD data differ from other vital registration (VR) sources because many countries have VR systems that only document and publish the underlying cause of death. MCoD data were used in the sepsis, infectious syndrome, and pathogen distribution component models and data processing, and modelling methods can be found in sections 4 and 6. MCoD-VR data came from the following sources.

- United States National Vital Statistics System
- Brazil Mortality Information System
- National Institute of Statistics (Italy)
- Statistics South Africa
- National Institute of Statistics and Geography (Mexico)
- National Administrative Department of Statistics (Colombia)
- Taiwan Ministry of Health and Welfare
- United Arab Emirates Vital Statistics
- Mongolia Vital Registration

Section 2.2: Hospital discharge

Hospital admissions and discharge data are data sources collected from inpatient hospital and other clinical settings. These data include information on the primary and other diagnosis for each patient, as applicable, and were obtained from the sources listed below. Hospital data were used in the sepsis, infectious syndrome, pathogen distribution, and case fatality ratio component models and data processing, and modelling methods can be found in sections 4–6.

- USA National Hospital Discharge Survey
- USA State Inpatient Databases
- Brazil Hospital Information System
- Italy Hospital Inpatient Discharges
- Sistema Automatizado de Egresos Hospitalarios (Mexico)
- Austria Hospital Inpatient Discharges
- New Zealand National Minimum Dataset
- Georgia Hospital Data

- Mongolia H-Info Health System Data
- India - Mysore JSS Hospital Inpatient Data
- India - Punjab Dayanand Medical College and Hospital Data
- India - Bangalore St. John's Medical College Hospital Data
- Dayanand Medical College and Hospital Inpatient Data (India)
- Pakistan - Aga Khan University Hospital Data
- Libya - Tripoli Central Hospital Data
- Kyrgyzstan - Bishkek Clinical-Related Groups Hospital Claims

Section 2.3: Microbial data with outcome

Microbial data are data sources from hospital and lab networks that collect pathogen cultures from patients. The cultures are tested for both pathogen and the pathogen's resistance to antibiotics. The culture results are linked to patient outcome, diagnoses, or both. Microbial data without these outcomes or diagnoses are listed in section 2.4. These data also include the specimen from which the pathogen was isolated and whether the infection was community- or hospital-acquired, if available. When hospital versus community acquisition was not specified, we used the difference between admission or diagnosis date and the specimen collection date, and if 48 hours or less had passed between those two dates, then the infection was assumed to be community-acquired. We assumed the infection was hospital-acquired when more than 48 hours had passed, consistent with CDC/National Healthcare Safety Network guidelines.[3] Microbial data with outcome were used in the case fatality ratio, pathogen distribution, prevalence of resistance, and relative risk component models and data processing, and modelling methods can be found in sections 5–8. Microbiology data types, with outcome and diagnoses were obtained from the sources below.

- **USA Becton, Dickinson, and Co. (BD) Insights, Research and Analytics Database microbiology test and in-patient hospital data:** data procured by BD via MedMined. Covers a range of regions in the USA from 2011 to 2017.
- **UK Infections in Oxfordshire Research Database (IORD):** patient microbiology and episodes data from Oxford University Hospitals NHS Foundation Trust.
- **International Nosocomial Infection Control Consortium (INICC) surveillance online system:** data from the INICC data collection software. ICU patient microbiology and hospital data from 50 countries across Latin America, Asia, the Middle East, eastern Europe, and Africa from 2009 to 2020.
- **Medical University of Varna, Varna, Bulgaria**: Antimicrobial resistance data from 2014–2020.
- **St. George's Hospital, University of London - Global Antimicrobial Resistance, Prescribing and Efficacy Among Neonates and Children (SGUL-GARPEC) Project bloodstream infection data:** Penta-sponsored global surveillance network focusing on neonatal and paediatric antimicrobial resistance and the organisms causing bloodstream infections.
- **Burden of Antibiotic Resistance in Neonates from Developing Societies (BARNARDS)**: BARNARDS includes locations in Nigeria, South Africa, Pakistan, Rwanda, Bangladesh, Ethiopia, and India from 2015 to 2018.
- **Lao-Oxford-Mahosot Hospital-Wellcome Trust Research Unit (LOMWRU):** information from children and adults with fever who were admitted as inpatients between 1996 and 2019 to Mahosot Hospital, Vientiane, Laos. Microbial analysis was carried out by the Microbiology Laboratory at Mahosot Hospital.
- **Kumasi Centre for Collaborative Research in Tropical Medicine (KCCR), Kumasi, Ghana (together with the Bernhard Nocht Institute for Tropical Medicine):** Data on children and adults with fever admitted as inpatients in Ghana between 2007 and 2015.
- **Vietnam Hospital for Tropical Diseases, Ho Chi Minh City. Hospital-acquired infections in ICU patients:** prospective observational study at the Oxford University Clinical Research Unit (OUCRU) in the Ho Chi Minh City Hospital for Tropical Diseases, Vietnam, from November 2014 to January 2016 to assess the ICU-acquired colonisation and infections among adult patients with more than 48 hours of ICU stay.

- **Medical Research Council (MRC) Unit, The Gambia. Diagnostic antimicrobial susceptibility testing:** information on hospital admission and discharge, pathogens cultured, resistance susceptibility test and antibiotics prescribed between 2005 and 2015 from the MRC Unit, The Gambia, now part of the London School of Hygiene and Tropical Medicine.
- **Cambodia Oxford Medical Research Unit (COMRU) and Angkor Hospital for Children (AHC). Suspected invasive bacterial infection hospitalisations:** reports children aged 0–21 years who were hospitalised with suspected invasive bacterial infection between 2015 and 2018.
- **Taiwan hospital-acquired infections and outcomes:** infectious disease surveillance linked to vital registration from Taiwan (province of China).
- **Childhood Acute Illness and Nutrition (CHAIN) Network antimicrobial resistance data:** CHAIN Network study informs on hospitalised children under 2 years old with acute illness in Bangladesh, Burkina Faso, Pakistan, Kenya, Malawi, and Uganda.
- **Lima, Peru, Cayetano Heredia University (UPCH) antimicrobial resistance data:** data from UPCH hospital sites across Lima, Peru, with discharge disposition for infectious pulmonary disease.
- **Jordan King Abdulla University Hospital culture and sensitivity tests:** information on inpatients at the King Abdulla University Hospital in 2020 part of the Jordan University of Science and Technology.
- **Iran antimicrobial resistance in burn patients and identified in blood, cerebrospinal fluid, and urine cultures:** data from inpatients across different hospital sites in Iran between 2016 and 2020.
- **Dhaka, Bangladesh, Bangabandhu Sheik Mujib Medical University hospital inpatient data:** data from 201 inpatients in 2017 at the Bangabandhu Sheikh Mujib Medical University, Dhaka, Bangladesh.
- **Chiangrai Prachanukroh Hospital, Chiangrai Clinical Research Unit and Mahidol Oxford Tropical Medicine Research Unit:** data from inpatients with positive cultures at the Chiangrai Prachanukroh Hospital from 2017 to 2019.
- **KEMRI/US Army Medical Research Directorate, Kenya.**
- **Chennai, India, Kanchi Kamakoti CHILDS Trust Medical Research Foundation (CTMRF) hospital inpatient data.**
- **Mortality from Bacterial Infections Resistant to Antibiotics (MBIRA):** A case-control trial for optimisation of treatment of resistant *E. coli* and *K. pneumoniae* infections, with retrospective data from six African Hospitals in Nigeria (2011–2016), Ghana (2016), Senegal (2012–2016), Zimbabwe (2012–2017), Kenya (2002–2017), and South Africa (2010–2016).
- **The Surveillance for Enteric Fever in Asia Project (SEAP)** study data from Bangladesh, India, Indonesia, Nepal, and Pakistan provided by the Sabin Vaccine Institute.
- **HCL Lyon Sud Hospital Centre (HCL):** data from France.
- **Instituto Nacional de Ciencias Médicas y Nutrición Salvador Zubirán (INCMNSZ)** hospitalised patients of referral center in Mexico City.
- **Shaheed Benazir Bhutto University Hospital data from Sheringal, Pakistan.**
- **Global Neonatal Sepsis Observational Study (NeoOBS)** is a collaboration between GARDP, St George's University of London, Penta, University of Antwerp, MRC Clinical Trial Unit at University College London, and 19 hospitals mainly in limited-resource settings.
- **United Kingdom Health Security Agency (UKHSA):** mandatory and voluntary reports on invasive infections linked to hospitalisation data and outcomes.
- **Sabana Hospital, Bogota, Colombia:** hospitalised patients of this tertiary hospital.
- **Aga Khan University Hospital from Karachi, Pakistan.**

Section 2.4: Microbial data without outcome

Microbial data were also obtained from laboratories, which do not necessarily link to patients' hospital records or information on their discharge disposition. These sources report specimen or site of infection, pathogens isolated, antimicrobial susceptibility tests, age and gender, and other demographic characteristics. This

information proved useful to inform pathogen distribution and prevalence of resistance component models and data processing, and modelling methods can be found in sections 6 and 7. Microbial data without outcome and diagnoses were obtained from the sources below.

- **SENTRY:** SENTRY Antimicrobial Surveillance Program established by JMI Labs in 1997. Sites are in the USA, Europe, Latin America, parts of Asia, and the Western Pacific.
- **Germany National Point Prevalence Survey on Nosocomial Infections and Antibiotic Use (PPS HAI):** Point Prevalence Survey for 2016 data reporting the pathogen distribution for hospital-acquired infections. Data gathered by the Robert Koch Institute together with Charité Berlin.
- **Madagascar – Foundation Merieux:** data collected from inpatients with positive culture admitted in three hospital sites in Madagascar, funded by Foundation Merieux.
- **AMASS:** data collected in an automated tool by Oxford Tropical Network Research Units.
- **The European Surveillance System (TESSy):** managed by the European Centre for Disease Prevention and Control (ECDC), provided data from the following surveillance systems:
    - European Antimicrobial Resistance Surveillance Network (EARS-Net).
    - Food- and Waterborne Diseases and Zoonoses Surveillance Network.
    - Invasive Pneumococcal Disease Surveillance Network, including discharge disposition.
    - Gonococcal Antimicrobial Surveillance Programme.
    - Healthcare Associated Infections Surveillance Network (ICU protocol), including discharge disposition.
    - European Tuberculosis Surveillance Network.
    - European Surveillance of Antimicrobial Consumption Network.

  For the European Union/European Economic Area (EU/EEA), data were obtained from the European Surveillance System (TESSy) as provided by Austria, Belgium, Croatia, Cyprus, Czechia, Denmark, Estonia, Finland, France, Germany, Greece, Hungary, Iceland, Ireland, Italy, Latvia, Luxembourg, Malta, Netherlands, Norway, Poland, Portugal, Romania, Slovakia, Slovenia, Spain, Sweden, and the UK, and released by the European Centre for Disease Prevention and Control (ECDC).

- **Pfizer ATLAS Programme:** the Antimicrobial Testing Leadership and Surveillance (ATLAS) database includes the Tigecycline Evaluation Surveillance Trial (TEST), the Assessing Worldwide Antimicrobial Resistance Evaluation (AWARE) and the International Network for Optimal Resistance Monitoring (INFORM) programmes. The study spans in coverage across more than 70 countries between 2004 and 2017.
- **Malawi Queen Elizabeth Hospital microbiology tests of blood specimens:** microbiology tests of blood specimens from inpatients at the Queen Elizabeth Hospital in Malawi from 1998 to 2016, part of the Institute of Infection and Global Health, University of Liverpool, in collaboration with the Malawi-Liverpool-Wellcome Trust and the Wellcome Trust Sanger Institute.
- **Central African Republic National Laboratory of Clinical Biology and Public Health:** data collected by the Laboratoire National de Biologie Clinique et de Santé Publique in Central African Republic between 2017 and 2020.
- **The Ethiopian AMR surveillance:** conducted from July 2018 to July 2020 across sentinel surveillance sites and the National AMR Surveillance Coordinating Centre for the Ethiopian Public Health Institute.
- **Japan Nosocomial Infections Surveillance (JANIS):** a national surveillance programme designed to provide basic information on the incidence and prevalence of nosocomial infections and antimicrobial-resistant bacteria in Japanese medical settings. Data available from 2013.
- **Lancet Labs:** data obtained from Lancet Laboratories, a network of private laboratories across different sites in Africa.
- **The Typhoid Fever Surveillance in Africa Program (TSAP):** established by the International Vaccine Institute to obtain comparable incidence data on typhoid fever and invasive non-typhoidal

Salmonella disease in Ghana, Burkina Faso, Ethiopia, Guinea Bissau, Kenya, Madagascar, Senegal, South Africa, Sudan, and Tanzania.

- **Invasive *Salmonella* infections at multiple surveillance sites in the Democratic Republic of the Congo study:** data published as part of the study on invasive *Salmonella* infections at multiple surveillance sites in the Democratic Republic of the Congo between 2011 and 2014.
- **Suva, Fiji, Colonial War Memorial Hospital:** Information on sequential *S. aureus* and Enterobacterial bloodstream infections at the Colonial War Memorial Hospital (analysis by Monash University) in Suva, Fiji, between 21 July 2020 and 29 October 2020.
- **World Health Organization (WHO) Global Tuberculosis Programme.**
- **Germany EARS-Net surveillance data 2017–2018:** data gathered by the Robert Koch Institute together with Charité Berlin.
- **WHO Meningitis surveillance:** sentinel hospital surveillance of suspected meningitis cases among children under 5 years old and positive cultures, provided by the World Health Organization (WHO) Global Rotavirus, Invasive Bacterial Vaccine Preventable Diseases Surveillance Network Collaboration from 2008 to 2020.
- **United States Active Bacterial Core Surveillance (ABCs) Reports:** case reports on health-care-associated infections and community interface infections from the Emerging Infections Program Network coordinated by the Centers for Disease Control and Prevention (CDC).
- **Greece National Reference Centre for Salmonella and Shigella:** *Shigella* data.
- **Oxford University Clinical Research Unit, Hanoi, Vietnam:** *Shigella* data from Bhutan, Cambodia, Thailand, and Vietnam.
- **National Ministry of Health of New Zealand:** linked national registry and microbial data.
- **Austria National Reference Centre for Shigella (AGES):** *Shigella* data.
- **Netherlands National Institute for Public Health and the Environment (RIVM):** *Shigella* data.
- **Cyprus National Reference Laboratory** for Salmonella & Shigella: *Shigella* data.
- **Bulgaria National Center of Infectious and Parasitic Diseases:** *Shigella* data**.**
- **Glaxo Smith Kline (GSK) Survey on Antibiotic Resistance (SOAR)** data from nine European countries: Ukraine, Russia, Slovakia, Bulgaria, Croatia, Romania, Czech Republic, Greece, Serbia.
- **Instituto Nacional de Salud de Colombia and Colombian Ministry of Health** data reported to SIREVA and WHONET surveillance networks.
- **SMART:** Study for Monitoring Antimicrobial Resistance Trends, which monitors complicated intra-abdominal infections (cIAIs), complicated urinary tract infections (cUTIs), and respiratory infections worldwide, funded by Merck & Co.
- **Venatorx (Global Surveillance)**: 50 countries (majority Americas and Europe): Argentina, Australia, Brazil, Canada, Chile, Colombia, Costa Rica, Croatia, Czech Republic, Denmark, Dominican Republic, Ecuador, Finland, France, Germany, Greece, Guatemala, Hong Kong, Hungary, India, Ireland, Israel, Italy, Japan, Kuwait, Latvia, Lithuania, Malaysia, Mexico, Morocco, Netherlands, New Zealand, Nigeria, Panama, Philippines, Poland, Portugal, Puerto Rico, Romania, Russia, Saudi Arabia, Singapore, South Africa, South Korea, Spain, Sweden, Switzerland, Taiwan, Thailand, Turkey, Ukraine, UK, USA, Venezuela, Vietnam.
- **Infectious Diseases Research Collaboration (IDRC):** Data from Tororo, Uganda.
- **Keystone Program** with surveillance data from 27 countries: Austria, Belarus, Belgium, Czech Republic, Denmark, Finland, France, Germany, Greece, Hungary, Ireland, Israel, Italy, Netherlands, Norway, Poland, Portugal, Romania, Russia, Slovenia, Spain, Sweden, Switzerland, Turkey, UK, Ukraine, USA.
- **SIDERO-WT** Programme with surveillance data from 13 countries: Canada, Czech Republic, France, Germany, Greece, Hungary, Italy, Russia, Spain, Sweden, Turkey, UK, and USA.
- **The United States National Respiratory and Enteric Virus Surveillance System by the Centers for Disease Control and Prevention (CDC):** Data from approximately 600 public health and clinical laboratories located throughout the country to monitor temporal and geographical circulation patterns of viral infections.

- **The World Health Organization (WHO) Global Influenza Surveillance and Response System (GISRS):** Data providing epidemiological and virological evidence that informs the introduction of vaccines and monoclonal antibodies from 25 countries across all six WHO regions.
- **The Influenza Hospitalization Surveillance Network (FluSurv-NET):** part of the Respiratory Virus Hospitalization Surveillance Network (RESP-NET) of population-based surveillance for laboratory-confirmed influenza, COVID-19, and respiratory syncytial virus (RSV)-associated hospitalisations through a network of acute care hospitals in 14 states in the USA, convened by the CDC.
- **The World Health Organization (WHO) FluNet:** a global web-based tool that provides weekly number of viruses and subtypes detected across countries in the six WHO regions and allows for tracking the movement of viruses globally.
- **ECDC Flu and RSV surveillance:** the ECDC collects weekly epidemiological and virological data from the EU/EEA Member States. Data are reported by sentinel influenza surveillance systems countries conduct on either universal or a subset of the population in each country. Several countries also conduct surveillance of hospitalised cases. A sample of cases is also tested to determine virus characteristics.
- **Argentina RSV surveillance.**
- **India RSV surveillance.**

Section 2.5: Literature studies

We conducted literature searches to obtain input data for the following components in the analysis: maternal and neonatal sepsis aetiology, lower respiratory infections (LRIs) aetiology, urinary tract infections (UTIs) aetiology, skin infections aetiology, meningitis aetiology and case fatality, intra-abdominal infection aetiology, bone and joint infections aetiology, prevalence of resistance, relative risk, and length of stay. Literature searches were performed on PubMed using the below search strings, and extracted studies covered the time range 1980–2020. Literature was used in the case fatality ratio, pathogen distribution, prevalence of resistance, and relative risk component models and data processing; modelling methods can be found in sections 5–8. Literature studies were also used as input into the modelling of the antibiotic usage covariate.[4]

Section 2.5.1: Maternal sepsis, neonatal sepsis, and LRI aetiology

Aetiology terms, combined with OR:

- Infection (Infect*)
- Microbiology (Microbiolog*)
- Aetiology (Aetiolog*)
- Etiology (Etiolog*)
- Virology (Virolog*)
- Bacteriology (Bacteriolog*)
- Fungus (fung*)

AND

Syndrome terms, combined with OR:

Maternal sepsis

- puerperal sepsis (puerper* sepsis)
- maternal sepsis (matern* sepsis)
- puerperal septicaemia (puerper* septicaemia, American spelling too - septicemia)
- maternal septicaemia (matern* septicaemia, American spelling too - septicemia)
- puerperal infection (puerper* infection)
- maternal infection (matern* infection)
- puerperal bacteraemia (puerper* bacteraemia, American spelling too - bacteremia)
- maternal bacteraemia (matern* bacteraemia, American spelling too - bacteremia)

Neonatal sepsis

- Neonatal sepsis (Neonat* sepsis within 3 or 5 words of each other)

- Neonatal septicaemia (Neonat* septicaemia within 3 or 5 words of each other, American spelling too - septicemia)
- Infant sepsis (Infant* sepsis)
- Infant septicaemia (Infant* septicaemia, American spelling too - septicemia)
- Neonatal bacteraemia (Neonat* bacteraemia, American spelling too - bacteremia)
- Infant bacteraemia (Infant* bacteraemia, American spelling too - bacteremia)

Lower respiratory infections

- LRI
- Lower respiratory infection
- LRTI
- Lower respiratory tract infection
- Pneumonia

Section 2.5.2: Urinary tract infections aetiology

("complicated"[Title/Abstract] OR "uncomplicated"[Title/Abstract]) AND (("Cystitis/etiology"[majr:noexp] OR "Cystitis/microbiology"[majr:noexp]) OR ("Pyelonephritis/etiology"[marj:noexp] OR "Pyelonephritis/microbiology"[majr:noexp]) OR ("Urinary Tract Infections/etiology"[majr:noexp] OR "Urinary Tract Infections/microbiology"[majr:noexp])) OR ("Urinary tract infections"[tiab] AND ("etiology"[tiab] OR "microbiology"[tiab]))

Section 2.5.3: Skin infections aetiology

(("Cellulitis/epidemiology"[majr:noexp] OR "Cellulitis/etiology"[majr:noexp] OR "Cellulitis/microbiology"[majr:noexp]) OR ("Pyoderma/epidemiology"[majr:noexp] OR "Pyoderma/etiology"[marj:noexp] OR "Pyoderma/microbiology"[majr:noexp]) OR "Pressure Ulcer/microbiology"[majr:noexp])

Section 2.5.4: Intra-abdominal infection aetiology

(("Peritonitis/epidemiology"[majr:noexp] OR "Peritonitis /etiology"[majr:noexp] OR "Peritonitis /microbiology"[majr:noexp]) OR ("Intraabdominal infections/epidemiology"[majr:noexp] OR "Intraabdominal infections /etiology"[marj:noexp] OR "Intraabdominal infections /microbiology"[majr:noexp]) OR ("abdominal abscess/epidemiology"[majr:noexp] OR " abdominal abscess /etiology"[majr:noexp] OR "abdominal abscess/microbiology"[majr:noexp]))

Section 2.5.5: Bone and joint infections aetiology

("Osteomyelitis/etiology"[majr:noexp] OR "Osteomyelitis/microbiology"[majr:noexp] NOT 'chronic') OR ("Arthritis, infectious/etiology"[marj:noexp] OR "Arthritis, infectious/microbiology"[majr:noexp] NOT ‘lyme’)

Section 2.5.6: Meningitis infection aetiology

((meningitis[title]) AND (1990/05/01[PDat]: 2018/12/31[PDat]) AND ((etiolog*[title/abstract]) AND Humans[MeSH Terms])

Section 2.5.7: Relative risk studies for specific drug-bug combinations

("Acinetobacter baumannii"[MeSH Terms] AND "carbapenem resistance"[All Fields]) OR ("Acinetobacter baumannii"[MeSH Terms] AND "carbapenem resistant"[All Fields])

('Escherichia coli'[MeSH Terms] AND 'carbapenem resistance'[All Fields]) OR ('Escherichia coli'[MeSH Terms] AND 'carbapenem resistant'[All Fields])

('Escherichia coli'[MeSH Terms] AND 'fluoroquinolone resistance'[All Fields]) OR ('Escherichia coli'[MeSH Terms] AND 'fluoroquinolone resistant'[All Fields])

('Escherichia coli'[MeSH Terms] AND 'third generation cephalosporin'[All Fields]) OR ('Escherichia coli'[MeSH Terms] AND ESBL OR extended-spectrum beta lactamase'[All Fields])

('Klebsiella pneumoniae'[MeSH Terms] AND 'third generation cephalosporin'[All Fields]) OR ('Klebsiella pneumoniae'[MeSH Terms] AND 'ESBL OR extended-spectrum beta lactamase'[All Fields])

('Klebsiella pneumoniae'[MeSH Terms] AND 'carbapenem resistance'[All Fields]) OR ('Klebsiella pneumoniae'[MeSH Terms] AND 'carbapenem resistant'[All Fields])

('Streptococcus pneumoniae'[MeSH Terms] AND 'penicillin resistance'[All Fields]) OR ('Streptococcus pneumoniae'[MeSH Terms] AND 'penicillin resistant'[All Fields])

('Pseudomonas aeruginosa'[MeSH Terms] AND 'carbapenem resistant'[All Fields] AND 'mortality' [MeSH Terms]) OR ('Pseudomonas aeruginosa'[MeSH Terms] AND 'carbapenem resistant' AND 'mortality' [All Fields])

('Enterococcus faec*'[MeSH Terms] AND 'vancomycin-resistant'[All Fields])

("haemophilus influenzae"[MeSH Terms] AND ("penicillin resistance"[MeSH Terms] OR ("penicillin"[All Fields] AND "resistance"[All Fields]) OR "penicillin resistance"[All Fields])) AND ("mortality"[Subheading] OR "mortality"[All Fields] OR "mortality"[MeSH Terms])

("streptococcus agalactiae"[MeSH Terms] AND ("azithromycin resistance"[MeSH Terms] OR ("azithromycin "[All Fields] AND "resistance"[All Fields]) OR " azithromycin resistance"[All Fields] OR "penicillin resistance"[MeSH Terms] OR ("penicillin"[All Fields] AND "resistance"[All Fields]) OR "penicillin resistance"[All Fields] OR "clindamycin resistance"[MeSH Terms] OR ("clindamycin"[All Fields] AND "resistance"[All Fields]) OR "erythromycin resistance"[All Fields] OR "erythromycin resistance"[MeSH Terms] OR ("erythromycin"[All Fields] AND "resistance"[All Fields]) OR "clindamycin resistance"[All Fields]) AND ("mortality"[Subheading] OR "mortality"[All Fields] OR "mortality"[MeSH Terms])

Section 2.5.8: Prevalence of resistance for specific organisms

Medical Subject Heading (MeSH) terms with free text terms in the title and abstract fields for *Escherichia coli*, *Klebsiella pneumoniae*, *Streptococcus pneumoniae*, and *Staphylococcus aureus* with the terms for antimicrobial drug resistance (resistan*, suscept*, surveil*, etc), limited from 1990 up to 2018. The search was undertaken on MEDLINE, Ovid Embase, Global Health, Cochrane Library.

For the 2021 GBD AMR update, two reviews that were modelled on the search terms above were undertaken:

The first rapid review ("AMR5-8 Pathogens") used Medical Subject Heading (MeSH) terms with free text terms in the title and abstract fields for *Acinetobacter baumannii*, *Pseudomonas aeruginosa*, *Enterobacter* species and *Enterococcus* species with the terms for antimicrobial drug resistance (resistan*, suscept*, surveil*, etc) and clinical syndromes (urinary tract infection, UTI, bacteremi*, bacteraemi*, blood stream infection* etc), limited from 1990 to 2018. The search was undertaken on MEDLINE and the Cochrane Database of Systematic Reviews.

The second rapid review ("AMR 11 Pathogens") used Medical Subject Heading (MeSH) terms with free text terms in the title and abstract fields for *Escherichia coli*, *Klebsiella pneumoniae*, *Streptococcus pneumoniae*, *Staphylococcus aureus*, *Acinetobacter baumannii*, *Pseudomonas aeruginosa*, *Enterobacter* species, *Enterococcus* species, *Morganell*a species, *Serratia* species, *Proteus* species with the terms for antimicrobial drug resistance (resistan*, suscept*, surveil*, etc) and clinical syndromes (urinary tract infection, UTI, bacteremi*, bacteraemi*, blood stream infection* etc), limited from 2018 to 2023. The search was undertaken on MEDLINE and the Cochrane Database of Systematic Reviews.

Medical Subject Headings (MeSH) and free text terms for the pathogens of interest (eg, *S* Typhi, *S* Paratyphi A, enteric fever) with terms for antimicrobial resistance (eg, resistan*, suscept*, surveil*). The search was undertaken on MEDLINE, Ovid Embase, Global Health, Cochrane Library, Scopus, Web of Science-Core Collection and LILACS regional WHO database.

Medical Subject Heading (MeSH) terms with free text terms in the title and abstract fields for non-typhoidal *Salmonella* or salmonellosis (non-typhi or nontyph or non-typh Salmonel…) with the terms for antimicrobial drug resistance (resistan*, suscept*, surveil*, etc) and invasive (bloodstream infection, septicaemia, etc.), limited from

1990 up to the search date. The search was undertaken on MEDLINE, Ovid Embase, Global Health, Cochrane Library, Scopus, Web of Science-Core Collection and LILACS regional WHO.

Medical Subject Heading (MeSH) terms with free text terms in the title and abstract fields for *Shigella* or shigellosis with the terms for antimicrobial drug resistance (resistan*, suscept*, surveil*, etc), limited from 1990 up to the search date. The search was undertaken on MEDLINE, Ovid Embase, Global Health, Cochrane Library, Scopus, Web of Science-Core Collection and LILACS regional WHO database.

Medical Subject Heading (MeSH) terms with free text terms in the title and abstract fields for *Neisseria gonorrhoeae*, with the terms for antimicrobial drug resistance (resistan*, suscept*, surveil*, etc), MDR, XDR, limited from 1990 up to the search date. The search was undertaken on MEDLINE, Ovid Embase, Global Health, Cochrane Library, Scopus, Web of Science-Core Collection and LILACS regional WHO database.

Section 2.6: Single drug-resistance profiles

Data sources used to inform single drug-resistance profiles were obtained from surveillance networks and aggregated reports where the full antibiogram of a pathogen for all drugs tested is not reported. Data from these sources generally do not include any individual records linked to a patient outcome. They are used to inform current and past resistance trends for specific pathogen–drug combinations. Single drug-resistance data were used in the prevalence of resistance component model and data processing, and modelling methods can be found in section 7. The data sources for single drug-resistance profiles were obtained from the sources below.

- **GLASS:** Global Antimicrobial Resistance Surveillance System by WHO.
- **CAESAR:** Central Asian and European Surveillance of Antimicrobial Resistance (CAESAR) is a network of national AMR surveillance systems and includes 19 countries in the WHO European Region that are not part of EARS-Net.
- **NARMS:** The National Antimicrobial Resistance Monitoring System for Enteric Bacteria (NARMS) is a collaboration of agencies within the US Department of Health and Human Services (HHS) (FDA and CDC) and the US Department of Agriculture (USDA). It tracks enteric bacteria and selected animal pathogens and their resistance to antimicrobials, and data are available from 1997 onward.
- **SOAR:** Survey on Antibiotic Resistance (SOAR) sponsored by GSK.
- **ReLAVRA and SIREVA:** The Latin American Network for Antimicrobial Resistance Surveillance (ReLAVRA by its Spanish acronym) and the Serotype and Antimicrobial Resistance Surveillance Program (SIREVA by its English acronym), which are coordinated by the Pan-American Health Organization (WHO/PAHO).
- **South Africa National Institute for Communicable Diseases (NICD):** Aggregated data from South Africa’s AMR surveillance in public health care centres which are submitted to GLASS.
- **Surveillance on Invasive Pulmonary Disease by the New Zealand Public Health Action.**
- **Surveillance of Antimicrobial Resistance in Hospital Acquired Infection by Kendokteran Laboratorium, Indonesia.**
- **Alliance for the Prudent Use of Antibiotics (APUA), Nepal.**
- **Hospital Civil de Guadalajara Fray Antonio Alcalde, Mexico.**
- **Australian Group on Antimicrobial Resistance (AGAR).**
- **Antimicrobial Resistance Surveillance Program (ARSP).**
- **Antimicrobial Use and Resistance in Australia (AURA).**
- **Australian Government Department of Health.**
- **Canadian Antimicrobial Resistance Surveillance System.**
- **The China Antimicrobial Surveillance Network.**
- **National Surveillance of Antimicrobial Resistance, Malaysia.**
- **Pakistan Antimicrobial Resistant Network.**

Section 2.7: Pharmaceutical sales and antibiotic use

These data were used to model the antibiotic consumption covariate, which was used as an input in the prevalence of resistance models; full details on this model can be found in section 7. Pharmaceutical sales and antibiotic use data were obtained from the following sources for the years 2000 to 2018 (unless stated otherwise).

- **IMS Health and Quintiles (IQVIA):** antibiotic sales data for 77 countries.
- **Demographic and Health Surveys (DHS):** households health surveys carried out across more than 90 countries; they include questions on antibiotic usage among those who had cough in a period of two weeks before the survey.
- **Multiple Indicators Cluster Surveys (MICS):** households health surveys carried out across more than 90 countries; they include questions on antibiotic usage among those who had cough in a period of two weeks before the survey.
- **European Surveillance of Antimicrobial Consumption Network (ESAC-NET):** antibiotic consumption data for five countries over 101 country-years.
- **WHO report on surveillance of antibiotic consumption: 2016–2018 early implementation:** report on antibiotic consumption for 21 countries over 21 country-years.

Section 2.8: Mortality surveillance

Mortality surveillance data were used in the sepsis, syndrome, and pathogen distribution models; full details on these models can be found in sections 4 and 6. Mortality surveillance data came from the source listed below.

- **Child Health and Mortality Prevention Surveillance (CHAMPS):** Under-5 mortality surveillance sites in South Africa, Mali, Bangladesh, Kenya, Ethiopia, and Mozambique. Researchers use minimally invasive tissue sampling (MITS) to gather information about pathogens involved and are able to discern a more accurate cause of death.

Section 2.9: Linkage (mortality only)

Hospital-linked cause of death records (linkage) data were used in sepsis and infectious syndrome models; full details on these models can be found in section 4. Mortality-only linkage data include:

- **Italy Friuli-Venezia Giulia MCoD data.**
- **New Zealand linked national minimum dataset to mortality collection data.**

Section 2.10 Insurance claims data

Insurance claims data were used in pathogen distribution models, and AMR incidence estimation; full details on these models can be found in section 4, 6 and 9. Insurance claims data include:

- **Poland National Health Fund:** National insurance claims records from 2015 to 2021.

Section 3: Summary of GBD 2021 estimation process[1]

A comprehensive description of data sources, data quality, statistical modelling, and analyses for GBD 2021 have been reported elsewhere.[2] To download the data used in these analyses, please visit the Global health Data Exchange GBD 2021 website (https://ghdx.healthdata.org/gbd-2021/sources). A brief summary of the fatal and non-fatal estimation processes is given below.

Section 3.1: GBD 2021 cause of death estimation process

The overarching steps for the fatal estimation process for each age, sex, location, and year are to first estimate all-cause mortality rates, then calculate cause-specific mortality rates, and finally scale the cause-specific mortality rates to the all-cause mortality rates for internal consistency. First, all-cause mortality is estimated using 22 223 sources as data inputs for under-5 mortality estimation and adult mortality estimation. ST-GPR was used to produce estimates of HIV-free mortality rate for every location-year after adjusting for completeness and other known biases in the input data. Added to this HIV-free mortality rate are the HIV-specific mortality rate and deaths from fatal discontinuities, or shocks, which are events that are stochastic in nature and cannot be modelled using standard GBD modeling tools, such as natural disasters and conflicts. GBD then estimated the cause-specific mortality rates of 288

diseases and injuries using the cause of death ensemble model (CODEm). This cause of death analysis used 56 604 sources in the cause of death (CoD) database. There are eight types of data sources in the CoD database: vital registration, verbal autopsy,[5] cancer registry, police records, sibling history, surveillance, survey/census, and minimally invasive tissue sampling (MITS) diagnoses. VR is considered the most comprehensive source of cause of death data, but less than half the world's population has deaths captured in a VR system (appendix figure S6), so causes of death statistics are supplemented with other data types. These various data sources are largely ICD-coded causes of death and use heterogenous ICD versions so are standardised to GBD causes of death. Once standardised and adjusted for known biases due to ICD classification changes,[6] garbage coding,[6–8] HIV correction,[9] stochastic noise,[2] and completeness,[10] causes of death are modelled using CODEm[11] to determine the cause fraction for each underlying cause of death by age, sex, year, and location. CODEm provides an ensemble prediction based on a combination of candidate models that vary across outcome and covariate combinations chosen for out-of-sample predictive performance. Because each cause is modelled independently, it is possible the sum of these models will not equal the all-cause mortality estimates, so cause-specific results are run through the CoDCorrect process to make cause-specific and all-cause mortality estimates internally consistent. This process rescales cause-specific estimates to the all-cause mortality envelope.

Section 3.2: GBD 2021 non-fatal estimation process

Non-fatal health outcomes are estimated using DisMod-MR 2.1, a Bayesian-regression analytical tool that synthesises various data inputs to produce estimates of disease incidence and prevalence. The data used for this analysis include systematic reviews done at the Institute for Health Metrics and Evaluation (IHME), data from household surveys including the demographic and health surveys, multiple indicator cluster surveys, living standards measurement surveys, reproductive health surveys, administrative claims data, inpatient hospital discharge records, outpatient hospital data, disease registries, programme-level data on disease burden from government agencies, surveillance system data on disease burden, and sources suggested to us by in-country collaborators and surveys identified in major multinational survey data catalogs such as the WHO Central Data Catalog. 75 213 sources were used for this analysis, 37 006 reporting incidence and 22 076 reporting prevalence. Data from these sources are extracted. Pre-modelling bias adjustments are made using crosswalking to account for various sources of bias, such as heterogeneous case definitions and methods of measurement. The pre-modelling bias adjustments are made using the MR-BRT environment, a meta-regression tool that allows for Bayesian priors, regularisation, and trimming and has been described in greater detail previously.[12] Using these bias-adjusted data, an estimate of prevalence and incidence for each cause is produced using the DisMod-MR 2.1 modelling framework. DisMod-MR 2.1 accepts all available data on mortality, incidence, prevalence, and remission and uses a compartmental model to enforce consistency between all quantities.

Section 4: Sepsis and infectious syndrome estimation[1]

Section 4.1: Input data

Section 4.1.1: Multiple causes of death

MCoD data are individual-based records that provide underlying causes of death and one or more intermediate causes in the chain of death. Additionally, each record includes age, sex, residence, and the date of death.

Section 4.1.2: Hospital record with multiple diagnoses and discharge status of death

This type of data is an individual-based hospital record of a patient that provides the main diagnosis and one or more additional diagnoses. Additionally, each record includes age, sex, residence, date of admission, date of discharge, and outcome (dead or alive). Only hospital discharges with discharge status of death were used in this component model, since we aimed to estimate the fraction of deaths that involve infection and the infectious syndrome distribution of those deaths.

Section 4.1.3: Linkage data

Linkage data are generated using probabilistic methods in a defined population that link individual-based hospital data to individual-based MCoD data. Linkage data offer a wider dataset that includes main diagnosis, other diagnoses, underlying cause of death, and intermediate causes of death in the chain.

Section 4.1.4: Mortality surveillance (Child Health and Mortality Prevention Surveillance [CHAMPS])

The CHAMPS network tracks the causes of under-5 mortality and stillbirths at sites in sub-Saharan Africa and south Asia through epidemiological surveillance of under-5 deaths and stillbirths utilising minimally invasive tissue sampling (MITS), laboratory diagnostics including conventional and advanced histopathology and molecular screening of various pathogens, verbal autopsy, and available clinical and demographic data.

Section 4.1.5: Claims data with multiple diagnoses and fatal outcome

Insurance claims data are individual-based records that provide multiple ICD codes related to the recorded health insurance claim. Each record includes age, sex, residence, date of claim, and outcome (dead or alive). Only claims records with outcome status of death were used in this component model.

Table 4.1.5: Input data for calculation of fraction of death by sepsis in different underlying causes

Location	Data type	Years	Year range	Death Records
USA	MCoD	43	1980–2022	81,390,689
	Hospital data with fatal outcome	31	1980–2010	3,292,373
	Linkage data	--	--	--
Brazil	MCoD	24	1999–2022	26,689,660
	Hospital data with fatal outcome	6	2015–2020	1,052,826
	Linkage data	--	--	--
Italy	MCoD	18	2003–2020	10,051,490
	Hospital data with fatal outcome	17	2005–2021	4,124,658
	Linkage data	16	2003–2018	123,050
South Africa	MCoD	20	1997–2016	5,136,003
	Hospital data with fatal outcome	--	--	--
	Linkage data	--	--	--
Mexico	MCoD	13	2009–2022	8,635,151
	Hospital data with fatal outcome	20	2000–2020	892,512
	Linkage data	--	--	--
Colombia	MCoD	25	1998–2022	5,246,587
	Hospital data with fatal outcome	--	--	--
	Linkage data	--	--	--
Taiwan (province of China)	MCoD	10	2008–2017	1,329,259
	Hospital data with fatal outcome	--	--	--

	Linkage data	--	--	--
Austria	MCoD	--	--	--
	Hospital data with fatal outcome	18	2001–2018	477,545
	Linkage data	--	--	--
New Zealand	MCoD	--	--	--
	Hospital data with fatal outcome	10	2011–2020	152,272
	Linkage data	11	2000–2010	165,265
United Arab Emirates	MCoD	5	2014–2018	61975
	Hospital data with fatal outcome	--	--	--
	Linkage data	--	--	--
Georgia	MCoD	--	--	--
	Hospital data with fatal outcome	7	2014–2020	36,084
	Linkage data	--	--	--
Mongolia	MCoD	5	2018–2022	30,676
	Hospital data with fatal outcome	2	2019–2020	2
	Linkage data	--	--	--
India	MCoD	--	--	--
	Hospital data with fatal outcome	4	2014–2017	14,337
	Linkage data	--	--	--
Pakistan	MCoD	--	--	--
	Hospital data with fatal outcome	3	2017–2019	8,433
	Linkage data	--	--	--
Libya	MCoD	--	--	--
	Hospital data with fatal outcome	2	2019–2020	426
	Linkage data	--	--	--
Kyrgyzstan	MCoD	--	--	--
	Hospital data with fatal outcome	1	2012	9
	Linkage data	--	--	--

Philippines	Claims data with fatal outcome	1	2016	89,276
CHAMPS Surveillance Sites: Kenya, Ethiopia, Mozambique, Mali, Bangladesh, Sierra Leone, and South Africa	MITS	6	2017–2022	1,805
Total	MCoD	163	1980–2022	138,571,490
	Hospital data with fatal outcome	121	1980–2022	10,051,477
	Linkage data	27	2000–2018	288,315
	Claims data with fatal outcome	1	2016	89,276
	MITS	6	2017–2022	1,805

Section 4.2: Data processing

Data for the USA, Brazil, Italy, South Africa, and Mexico were extracted at the subnational level by GBD 2021 age groups, sex, year, and causes of death and/or diagnoses, while data for the remaining countries and territories were analysed at the national level. This allowed us to expand the location-years of data that we had for each Socio-demographic Index (SDI)[13] value.

Section 4.3: Mapping the data

Prepared data were mapped to GBD causes. The GBD cause list is a mutually exclusive and collectively exhaustive list of diseases and injuries. The GBD cause list is organised hierarchically to accommodate different purposes and needs of various users. The first two levels aggregate causes into general groupings. At Level 1, there are three cause groups: communicable, maternal, neonatal, and nutritional diseases (Group 1 diseases); non-communicable diseases (Group 2); and injuries (Group 3). These Level 1 aggregates are subdivided at Level 2 of the hierarchy into 22 cause groupings (eg, neonatal disorders, neurological disorders, and transport injuries). The disaggregation into Levels 3 and 4 contains the finest level of detail for causes captured in GBD 2021. See appendix 2, table S1 for the full GBD cause hierarchy by level.

The underlying cause of death or main diagnosis for each record in the data was mapped to a GBD cause. After the mapping of underlying cause, we used the GBD 2021 garbage code redistribution algorithm (see appendix 1, section 3.7 in Naghavi et al.[2]) to ensure that all deaths had a plausible and specific underlying cause of death. The redistribution of garbage codes for underlying causes of death followed the same age and sex restrictions as GBD 2021. We did not redistribute garbage codes in the chain causes because the concept of a garbage code applies only to plausible underlying cause of death (see Rudd et al.[14] and appendix 1, section 3.7 in Naghavi et al.[2]).

Section 4.4: Intermediate cause and infectious syndrome mapping hierarchy

Section 4.4.1: Intermediate cause mapping

Within our modelling framework, we first classified whether each death record included sepsis. Deaths were classified as:

Explicit sepsis (A40, R65.2 in ICD-10 and 039 in ICD-9): Any death which had a specific ICD code for sepsis in the MCoD chain or hospital diagnoses was considered explicit sepsis.[14]

Implicit sepsis: Any death that had an infectious disease code in the underlying cause or cause chain that was not explicitly sepsis was considered implicit sepsis.

Non-sepsis: Any death that did not meet either of the two above criteria (appendix 2, tables S2, S3).

Then, we limited our analysis to only explicit and implicit sepsis deaths, which we classified into infectious syndromes. An infectious syndrome is the infection directly responsible for sepsis and serves as the bridge between the underlying cause of death and sepsis. Infectious syndromes can be both underlying causes of death and intermediate causes of death.

Based on the ICD-coded diagnoses in the multiple cause of death records, we then assigned the deaths to all 22 infectious syndromes and 26 infectious diseases (HIV, malaria, tetanus, neglected tropical diseases estimated by the GBD [Ebola, Zika virus, dengue fever, African trypanosomiasis, leishmaniasis, chagas disease, rabies, yellow fever, cysticercosis, dracunculiasis, echinococcosis, schistosomiasis, trachoma, other neglected tropical diseases], COVID-19, measles, whooping cough, diphtheria, rheumatic heart disease, chlamydia, gonorrhoea, syphilis, and other sexually transmitted infections) that applied. Of the 22 infectious syndromes (table 4.4.2.1), only 11 contributed to AMR burden (table 4.4.1.1). Assignment of deaths to other infectious syndromes and diseases was important to ensure a comprehensive and mutually exclusive assignment of sepsis mortality.

Table 4.4.1.1: Infectious syndromes contributing to AMR

	**Infectious syndrome**
**1**	Bloodstream infections
**2**	Meningitis
**3**	Lower respiratory infections
**4**	Endocarditis
**5**	Peritoneal and intra-abdominal infections
**6**	Diarrhoea
**7**	Urinary tract infections and pyelonephritis
**8**	Infections of bones, joints, and related organs
**9**	Infections of the skin and subcutaneous systems
**10**	Tuberculosis
**11**	Typhoid, paratyphoid, and invasive non-typhoidal Salmonella

Section 4.4.2: Informative ranking

Due to our data often having multiple diagnoses associated with each record, a single case of sepsis could potentially map to multiple candidate infectious syndromes. Because multiple infectious syndrome assignments pose a risk of double counting, we employed an informative ranking hierarchy. The informative ranking allowed us to determine the infectious syndrome that provided the most information on the culprit pathogen. The goal of this hierarchy was to produce the most accurate pathogen burden estimate such that when there were multiple infectious syndromes, we prioritised the syndrome with the most distinctive distribution. For example, bloodstream infections (BSIs) are common infections in sepsis but there is often an earlier source of the infection, such as a urinary tract infection (UTI), cellulitis (skin infection), or lower respiratory infection (LRI), and each has a unique pathogen distribution that provides more information than the distribution of BSI. In the event that a patient record reflected both BSI and LRI, we would assign the infectious syndrome based on which ranked as most informative with respect to the underlying pathogen, in this case LRI (table 4.4.2.1).

Table 4.4.2.1. Level 1 infectious syndrome informative ranking hierarchy. Organised from most informative (top) to least (bottom).

**Rank**	**Infectious syndrome model informative ranking hierarchy**
1	Meningitis
2	Myelitis, meningoencephalitis, and other central nervous system infections*

3	Encephalitis*
4	Endocarditis
5	Carditis, myocarditis, and pericarditis*
6	Peritoneal and intra-abdominal infections
7	Lower respiratory infections
8	Other unspecified respiratory site infections**
9	Upper respiratory infections*
10	Infections of bones, joints, and related organs
11	Typhoid, paratyphoid, and invasive non-typhoidal Salmonella
12	Diarrhoea
13	Hepatitis*
14	Urinary tract infections and pyelonephritis
15	Genital infections*
16	Infections of the skin and subcutaneous systems
17	Oral infections*
18	Eye infections*
19	Tuberculosis
20	Bloodstream infections
21	Other unspecified site infections*
22	Other parasitic infections*

* Infectious syndrome models marked with a ‘*’ do not contribute to the estimate of AMR burden

** “Other unspecified respiratory site infections”, marked with ‘**’, was not considered an infectious syndrome but was included as an additional model that was aggregated into lower respiratory infections.

Section 4.4.3: Two modelling pathways

After mapping the underlying and chain causes of death, our database went through two separate modelling pathways. The first model estimated the fraction of deaths that are sepsis-related in each GBD cause; these sepsis-related deaths for non-infectious GBD causes were combined with GBD deaths for infectious causes to create the total envelope of all deaths where infection plays a role. The second pathway estimated each infectious syndrome as a fraction of sepsis-related mortality in each GBD cause. In the last step of infectious syndrome estimation, the fractions of sepsis by modelled infectious syndromes normalised to one so as not to exceed the sepsis mortality envelope and were multiplied by the sepsis estimate in each GBD cause by country and territory, age, sex, and year in 2021.

For the aforementioned 26 infectious diseases (eg, HIV, malaria, tetanus) we used estimates from GBD 2021. For the 22 infectious syndromes, we ran models for all infectious syndromes except tuberculosis and typhoid, paratyphoid, and invasive non-typhoidal Salmonella as detailed in section 4.6.

Section 4.5: First pathway: Estimating the envelope of sepsis

Section 4.5.1: Sepsis model

We used a mixed-effects binomial logistic regression to model the logit of the fraction of sepsis-related deaths by GBD cause-age-sex-location, consistent with the modelling approach used by Rudd et al.[14] Sex and Healthcare Access and Quality Index (HAQ Index)[15] were included as covariates, and a nested random effect on underlying

cause of death was included. A separate model was run for each GBD 2021 age group (0–6, 7–27, 1-5 months, 6-11 months, 12-23 months, 2-4 years, 5–9, 10–14, 15–19, 20–24, 25–29, 30–34, 35–39, 40–44, 45–49, 50–54, 55–59, 60–64, 65–69, 70–74, 75–79, 80–84, 85–89, 90–94, 95+ years):

$$sepsis\ related\ deaths \sim B(total\ deaths, sepsis\ fraction) \quad (4.5.1.1)$$

$$logit(sepsis\ fraction) = \beta_0 + \beta_1 * HAQ\ Index + \beta_2 * sex + \pi_{level\ 1, level\ 2}$$

Where $\pi_{level\ 1, level\ 2}$ is a nested random effect on underlying cause of death and B represents the binomial distribution. The nested random-effect's structure in the model on underlying cause of death allowed the prediction of sepsis fractions where data were limited by borrowing information from diseases within the same group.

There were 22 groups of underlying causes of death, each categorised by physiological relatedness. We produced our predictions by calculating a point estimate from the model for each GBD location, age group, sex, cause, and year. We calculated uncertainty intervals (UIs) as 1.96 standard deviations above and below the mean (point estimate). Uncertainty is attributable to sample size variability between data sources, data availability, and model specifications.

For all underlying causes of death that are infectious diseases, we used the GBD death estimates as the number of sepsis deaths rather than the modelled sepsis estimate, since infection inherently plays a role in these deaths even if the pathway did not explicitly include sepsis. The causes that impacted AMR burden and their associated infectious syndromes are listed in table 4.5.1.1.

Table 4.5.1.1. Underlying causes that are infectious diseases and their corresponding reported infectious syndromes

GBD cause name	Reported infectious syndrome
Appendicitis	Peritoneal and intra-abdominal infections
Skin diseases	Infections of the skin and subcutaneous systems
Diarrhoeal diseases	Diarrhoea
Endocarditis	Endocarditis
Invasive non-typhoidal Salmonella	Typhoid, paratyphoid, and invasive non-typhoidal Salmonella
Lower respiratory infections	Lower respiratory infections
Maternal sepsis and other maternal infections	Bloodstream infections
Meningitis	Meningitis
Neonatal sepsis and other neonatal infections	Bloodstream infections
Paratyphoid fever	Typhoid, paratyphoid, and invasive non-typhoidal Salmonella
Tuberculosis	Tuberculosis
Typhoid fever	Typhoid, paratyphoid, and invasive non-typhoidal Salmonella
Urinary tract infections and interstitial nephritis	Urinary tract infections and pyelonephritis

For all other causes, we calculated the number of sepsis-related deaths in each year by multiplying our predictions of cause-, age group-, sex-, year-, and location-specific sepsis fractions by GBD 2021 death estimates for 1990–2021. Finally, we aggregated our results to arrive at regional and global sepsis-related mortality in non-infectious underlying causes of death, which we combined with the GBD infectious disease deaths estimates to create the mortality envelope of all deaths related to infection.

Histograms of the available input data by Healthcare, Access, and Quality (HAQ) Index are shown below. MCoD input data are used to estimate the proportion of GBD cause-specific deaths that involve sepsis.

Figure 4.5.1.1. All MCoD input data by HAQ Index

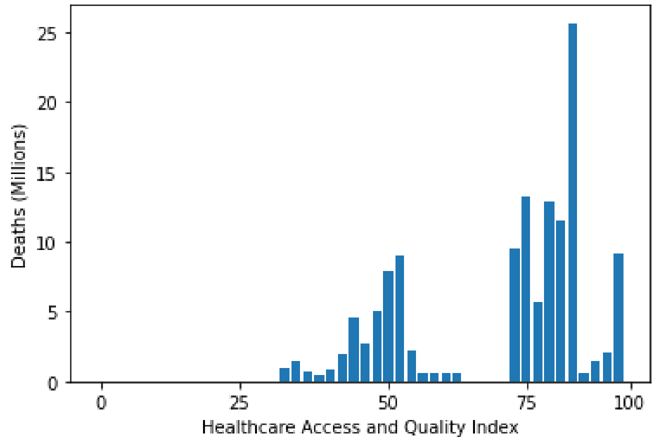

Figure 4.5.1.2. Sepsis-related MCoD input data by HAQ Index

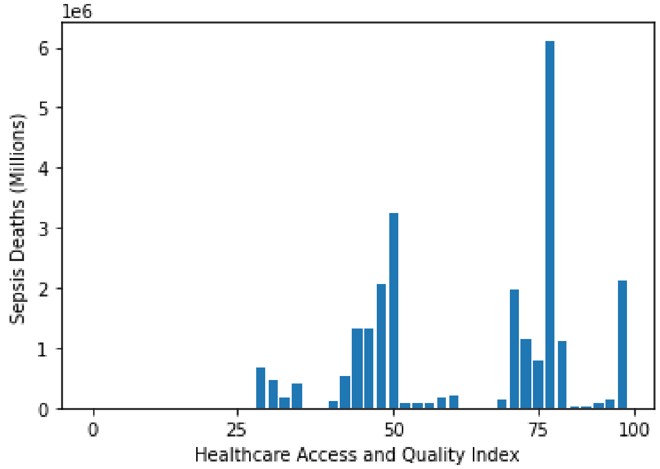

Section 4.6: Second pathway: Apportioning sepsis to specific infectious syndromes

We used a mixed-effects binomial logistic regression to model the logit of the infectious syndrome fraction of sepsis-related mortality by GBD cause. The model covariates varied by infectious syndrome (table 4.6.1): covariates were selected by disease experts based off strong theoretical relationships to the specific infectious syndrome. Where syndromes overlapped with GBD causes, we incorporated covariates selected in the GBD for fatal modelling by CODEm.[11] All models included HAQ Index as a covariate. Future improvements to infectious syndrome models may include incorporating a bespoke covariate selection tool or strategy.

The pathogen distribution for hospital-acquired infections (HAIs) and community-acquired infections (CAIs) differs markedly for some infectious syndromes.[16–20] To more accurately estimate the burden of pathogens responsible for infection, we separated infectious syndromes into hospital-acquired and community-acquired for LRI and UTI. For all ICD-coded administrative datasets (hospital discharge, MCoD, and linkage), we assumed that an infection was community-acquired if it was the primary diagnosis or underlying cause of death. By contrast, an infection was considered hospital-acquired if it was not the primary diagnosis or underlying cause of death. We recognise that this is a strong assumption that will not always be correct; however, there is no established method for determining HAI versus CAI in administrative data.[21,22] Hospital-acquired lower respiratory and urinary tract infections are estimated independently for this study, while GBD estimates are used for community-acquired lower respiratory and urinary tract infections.

We modelled 22 infectious syndromes. Each infectious syndrome model specified a list of GBD causes of death for which the model produced estimates. The ICD codes that make up the GBD causes of death can be found in appendix 2 table S2.

Table 4.6.1: Infectious syndrome model covariates and age groups

Model name	Covariates	Age groups
Sepsis	HAQ Index;[15]<br>sex	GBD 2021 age groups
Meningitis	HAQ Index;[15]<br>sex;<br>PCV3[23] (pneuomococcal conjugate vaccine 3) lagged five-year coverage, COVID-free (proportion);<br>Hib3[23] (Haemophilus influenzae type B vaccine) transformed: population-level coverage, including indirect effects (proportion)	0-59, 60-79, 80+
Encephalitis	HAQ Index;[15]<br>sex	0-14, 15-44, 45-64, 65-69, 70+
Myelitis, meningoencephalitis, and other central nervous system infections	HAQ Index;[15]<br>sex;<br>PCV3 lagged five-year coverage, COVID-free (proportion)	<1 year, 12-23 months, 2-4, 5-9, 10-14, 15-19, 20-24, 25-29, 30-34, 35-39, 40-44, 45-49, 50-54, 55-59, 60-64, 65-69, 70-74, 75-79, 80-84,85-89,90-94, 95+
Peritoneal and intra-abdominal infections	HAQ Index;[15]<br>sex	GBD 2021 age groups
Infections of the skin and subcutaneous systems	HAQ Index;[15]<br>sex;<br>sanitation[24] (proportion with access);<br>improved water source[24] (proportion with access);<br>diabetes fasting plasma glucose (mmol/L), by age	<1 year, 1-4, 5-14, 15-24, 25-34, 35-44, 45-54, 55-64, 65+
Oral infections	HAQ Index;[15]<br>sex;<br>dentists[25] per capita	0-14, 15-59, 60-79, 80+
Eye infections	HAQ Index;[15]<br>sex;<br>sanitation (proportion with access);<br>improved water source (proportion with access)	All Ages
Diarrhoea	HAQ Index;[15]<br>sex;<br>sanitation (proportion with access);<br>improved water source (proportion with access);<br>rotavirus coverage,[26] COVID-inclusive (proportion)	Under 5, 5-19, 20-24, 25-29, 30-44, 45-79, 80+
Hepatitis	HAQ Index;[15]<br>sex;	0-39, 40+

	vaccine-adjusted HbSAg (hepatitis B surface antigen) serporevalence age-standardised,[27]<br>hepatitis C seroprevalence[26] (anti-HCV) age-standardised,<br>intravenous drug use[24] (proportion by age)	
Genital infections	HAQ Index;[15]<br>sex;<br>total fertility rate[13]	10-24, 25-34, 35-39, 40-54, 55-59, 60-64, 65-69, 70-74, 75-79, 80-84, 85-89, 90-94, 95+
Bloodstream infections	HAQ Index;[15]<br>sex	GBD 2021 age groups
Endocarditis	HAQ Index;[15]<br>sex;<br>sanitation (proportion with access);<br>improved water source (proportion with access);<br>intravenous drug use (proportion by age)	0-9, 10-39, 40-44, 45-49, 50+
Other parasitic infections	HAQ Index;[15]<br>sex;<br>sanitation (proportion with access);<br>improved water source (proportion with access)	All Ages
Other unspecified site infections	HAQ Index;[15]<br>sex	Early Neonatal, Late Neonatal, Post Neonatal, 1 year +
Infections of bones, joints, and related organs	HAQ Index;[15]<br>sex	0–9, 10–44, 45–49, 50–54, 55–59, 60–64. 65–69, 70–74, 75–79, 80–84, 85–89, 90–94, 95+
Carditis, myocarditis, and pericarditis	HAQ Index;[15]<br>sex	0-14, 15-24, 25-44, 45-49, 50-54, 55-59, 60-64, 65-69, 70-74, 75-79, 80+
Lower respiratory infections	HAQ Index;[15]<br>sex;[27]<br>inpatient utilisation envelope	GBD 2021 age groups
Upper respiratory infections	HAQ Index;[15]<br>sex;<br>PCV3 lagged five-year coverage, COVID-free (proportion);<br>population density (over 1000 ppl/sqkm, proportion)	0-39, 40-64, 65+
Other unspecified respiratory site infections	HAQ Index;[15]<br>sex;<br>PCV3 lagged five-year coverage, COVID-free (proportion); population density (over 1000 ppl/sqkm, proportion)	0-14, 15-54, 55-64, 65-74, 75-84, 85+

Urinary tract infections and pyelonephritis	HAQ Index;[15]<br>sex;<br>inpatient utilisation envelope	<1 year, 12-23 months, 2-4, 5-9, 10-14, 15-19, 20-24, 25-29, 30-39, 40-44, 45-49, 50-54, 55-59, 60-64, 65-69, 70-74, 75-79, 80-84, 85-89, 90-94, 95+

CAI=community-acquired infection. HAI=hospital-acquired infection. HAQ Index=Healthcare Access and Quality Index. GBD 2021 age groups include early neonatal, late neonatal, 1–5 months, 6–11 months, 12–23 months, 2–4, 5–9, 10–14, 15–19, 20–24, 25–29, 30–34, 35–39, 40–44, 45–49, 50–54, 55–59, 60–64, 65–69, 70–74, 75–79, 80–84, 85–89, 90–94, 95+.

The infectious syndrome models were specified as mixed-effects binomial logistic regressions, one for each infectious syndrome and age group:

$$syndrome\ related\ deaths \sim B(total\ deaths, syndrome\ fraction) \qquad (4.6.2.1)$$

$$logit(syndrome\ fraction) = \beta_0 + \beta_1 * X + \pi_{level\ 1, level\ 2}$$

where β and X are vectors of length $n + 1$ for n covariates and $\pi_{level\ 1,level\ 2}$ is a nested random effect on underlying cause of death. The granularity of the age groups estimated for each infectious syndrome was chosen based on the age pattern of the infectious syndrome and limitations related to data sparsity.

As in the first pathway, we derived our predictions from modelled point estimates and UIs as 1.96 standard deviations above and below the point estimate for each GBD location, age group, sex, and cause in each year.

Section 4.6.1: Aggregation to the sepsis mortality envelope

We calculated the number of deaths attributable to each infectious syndrome in 2021 by multiplying our predictions of cause-, age group-, sex-, year-, and location-specific infectious syndrome fractions by our sepsis-mortality envelope estimates from the first pathway. All infectious syndrome fractions normalised to one prior to multiplication to ensure that we did not exceed the sepsis mortality envelope.

Finally, we aggregated our results to calculate regional and global sepsis-related mortality by infectious syndrome.

Section 4.6.2: Infectious syndromes using GBD 2021 results

Out of the 22 infectious syndromes included in our hierarchy, we did not model tuberculosis (TB) and typhoid, paratyphoid, and invasive non-typhoidal Salmonella. Instead, we used the published results from GBD 2021[2] for these causes of death, as we believe the GBD 2021 estimates fully represent these infectious syndromes because they are usually not intermediate causes of death.

Section 4.7: Model validation

Infectious syndrome modelling aims to predict which cases of infection belong to a specific infectious syndrome, which is a multi-class classification problem. We therefore use the Area Under the Receiver Operating Characteristics (ROC) Curve (AUC) to evaluate model performance. The ROC Curve plots the sensitivity (or true positive rate) against one minus the specificity (or false positive rate) for a given model, and a higher AUC score indicates a better-performing classification model. Accuracy is a related measure which considers the proportion of true positives and true negatives predicted by the model with respect to the total number of predictions.

For out-of-sample validation, we used a 5-fold cross-validation strategy that excluded 20% of the input data described in table 4.1.5 on each iteration. Table 4.7.1 reports the Accuracy and AUC score for each of the age groups within the infectious syndrome models, and table 4.7.2 reports the same metrics for the sepsis models.

Table 4.7.1: Accuracy and AUC score for out-of-sample validation of 11 infectious syndromes models contributing to AMR burden

Model	Age group name	Accuracy	AUC score
Bloodstream infections	Early Neonatal	0.88	0.95

Bloodstream infections	Late Neonatal	0.91	0.98
Bloodstream infections	1-5 months	0.94	0.96
Bloodstream infections	6-11 months	0.94	0.96
Bloodstream infections	12 to 23 months	0.94	0.96
Bloodstream infections	2 to 4	0.93	0.95
Bloodstream infections	5 to 9	0.91	0.95
Bloodstream infections	10 to 14	0.88	0.94
Bloodstream infections	15 to 19	0.87	0.92
Bloodstream infections	20 to 24	0.91	0.94
Bloodstream infections	25 to 29	0.94	0.96
Bloodstream infections	30 to 34	0.94	0.96
Bloodstream infections	35 to 39	0.94	0.95
Bloodstream infections	40 to 44	0.93	0.94
Bloodstream infections	45 to 49	0.92	0.92
Bloodstream infections	50 to 54	0.91	0.90
Bloodstream infections	55 to 59	0.90	0.88
Bloodstream infections	60 to 64	0.90	0.88
Bloodstream infections	65 to 69	0.90	0.87
Bloodstream infections	70 to 74	0.90	0.87
Bloodstream infections	75 to 79	0.91	0.87
Bloodstream infections	80 to 84	0.91	0.87
Bloodstream infections	85 to 89	0.92	0.88
Bloodstream infections	90 to 94	0.92	0.90
Bloodstream infections	95 plus	0.92	0.91
Diarrhoea	Under 5	1.00	0.81
Diarrhoea	5 to 19	0.99	0.71
Diarrhoea	20 to 24	1.00	0.66
Diarrhoea	25 to 29	1.00	0.52
Diarrhoea	30 to 44	1.00	0.50
Diarrhoea	45 to 79	1.00	0.50
Diarrhoea	80 plus	1.00	0.50
Endocarditis	0 to 9	1.00	0.62
Endocarditis	10 to 39	0.99	0.89
Endocarditis	40 to 44	0.99	0.87
Endocarditis	45 to 49	0.99	0.84
Endocarditis	50 plus	0.99	0.83
Infections of bones, joints, and related organs	0 to 9	1.00	0.56
Infections of bones, joints, and related organs	10 to 44	1.00	0.70
Infections of bones, joints, and related organs	45 to 49	1.00	0.77
Infections of bones, joints, and related organs	50 to 54	1.00	0.80
Infections of bones, joints, and related organs	55 to 59	1.00	0.80
Infections of bones, joints, and related organs	60 to 64	1.00	0.83

Infections of bones, joints, and related organs	65 to 69	1.00	0.83
Infections of bones, joints, and related organs	70 to 74	1.00	0.84
Infections of bones, joints, and related organs	75 to 79	1.00	0.85
Infections of bones, joints, and related organs	80 to 84	1.00	0.85
Infections of bones, joints, and related organs	85 to 89	1.00	0.86
Infections of bones, joints, and related organs	90 to 94	1.00	0.87
Infections of bones, joints, and related organs	95 plus	1.00	0.89
Infections of the skin and subcutaneous systems	<1 year	1.00	0.71
Infections of the skin and subcutaneous systems	1 to 4	0.99	0.75
Infections of the skin and subcutaneous systems	5-14 years	0.99	0.62
Infections of the skin and subcutaneous systems	15 to 24	0.99	0.79
Infections of the skin and subcutaneous systems	25 to 34	0.99	0.85
Infections of the skin and subcutaneous systems	35 to 54	0.99	0.86
Infections of the skin and subcutaneous systems	55 to 64	0.98	0.85
Infections of the skin and subcutaneous systems	65 plus	0.98	0.87
Lower respiratory infections	Early Neonatal	0.99	0.79
Lower respiratory infections	Late Neonatal	0.99	0.73
Lower respiratory infections	1-5 months	0.97	0.91
Lower respiratory infections	6-11 months	0.96	0.94
Lower respiratory infections	12 to 23 months	0.96	0.94
Lower respiratory infections	2 to 4	0.95	0.93
Lower respiratory infections	5 to 9	0.93	0.92
Lower respiratory infections	10 to 14	0.92	0.89
Lower respiratory infections	15 to 19	0.91	0.88
Lower respiratory infections	20 to 24	0.94	0.91
Lower respiratory infections	25 to 29	0.96	0.94
Lower respiratory infections	30 to 34	0.96	0.94
Lower respiratory infections	35 to 39	0.96	0.93
Lower respiratory infections	40 to 44	0.95	0.92
Lower respiratory infections	45 to 49	0.94	0.90
Lower respiratory infections	50 to 54	0.94	0.88
Lower respiratory infections	55 to 59	0.94	0.87
Lower respiratory infections	60 to 64	0.93	0.87
Lower respiratory infections	65 to 69	0.93	0.86
Lower respiratory infections	70 to 74	0.93	0.86
Lower respiratory infections	75 to 79	0.94	0.86
Lower respiratory infections	80 to 84	0.94	0.87
Lower respiratory infections	85 to 89	0.95	0.88
Lower respiratory infections	90 to 94	0.95	0.89
Lower respiratory infections	95 plus	0.95	0.90
Meningitis	0 to 59	1.00	0.68
Meningitis	60 to 79	1.00	0.62

Meningitis	80 plus	1.00	0.57
Other unspecified respiratory site infections	0 to 14	1.00	0.85
Other unspecified respiratory site infections	15 to 54	1.00	0.50
Other unspecified respiratory site infections	55 to 64	1.00	0.50
Other unspecified respiratory site infections	65 to 74	1.00	0.50
Other unspecified respiratory site infections	75 to 84	1.00	0.50
Other unspecified respiratory site infections	85 plus	1.00	0.51
Peritoneal and intra-abdominal infections	Early Neonatal	0.99	0.74
Peritoneal and intra-abdominal infections	Late Neonatal	0.99	0.81
Peritoneal and intra-abdominal infections	1-5 months	0.98	0.87
Peritoneal and intra-abdominal infections	6-11 months	0.99	0.85
Peritoneal and intra-abdominal infections	12 to 23 months	0.98	0.83
Peritoneal and intra-abdominal infections	2 to 4	0.99	0.82
Peritoneal and intra-abdominal infections	5 to 9	0.98	0.82
Peritoneal and intra-abdominal infections	10 to 14	0.98	0.82
Peritoneal and intra-abdominal infections	15 to 19	0.97	0.87
Peritoneal and intra-abdominal infections	20 to 24	0.97	0.91
Peritoneal and intra-abdominal infections	25 to 29	0.98	0.93
Peritoneal and intra-abdominal infections	30 to 34	0.98	0.94
Peritoneal and intra-abdominal infections	35 to 39	0.98	0.93
Peritoneal and intra-abdominal infections	40 to 44	0.97	0.92
Peritoneal and intra-abdominal infections	45 to 49	0.97	0.90
Peritoneal and intra-abdominal infections	50 to 54	0.97	0.88
Peritoneal and intra-abdominal infections	55 to 59	0.96	0.87
Peritoneal and intra-abdominal infections	60 to 64	0.96	0.87
Peritoneal and intra-abdominal infections	65 to 69	0.96	0.86
Peritoneal and intra-abdominal infections	70 to 74	0.96	0.86
Peritoneal and intra-abdominal infections	75 to 79	0.97	0.87
Peritoneal and intra-abdominal infections	80 to 84	0.97	0.87
Peritoneal and intra-abdominal infections	85 to 89	0.97	0.87
Peritoneal and intra-abdominal infections	90 to 94	0.98	0.87
Peritoneal and intra-abdominal infections	95 plus	0.98	0.87
Tuberculosis	0 to 59	1.00	0.55
Tuberculosis	60 to 74	1.00	0.78
Tuberculosis	75 plus	1.00	0.81
Typhoid, paratyphoid, and invasive non-typhoidal Salmonella	0 to 39	1.00	0.50
Typhoid, paratyphoid, and invasive non-typhoidal Salmonella	40 to 54	1.00	0.50
Typhoid, paratyphoid, and invasive non-typhoidal Salmonella	55 to 74	1.00	0.50
Typhoid, paratyphoid, and invasive non-typhoidal Salmonella	75 to 84	1.00	0.50
Typhoid, paratyphoid, and invasive non-typhoidal Salmonella	85 plus	1.00	0.50
Urinary tract infections and pyelonephritis	<1 year	1.00	0.66
Urinary tract infections and pyelonephritis	12 to 23 months	1.00	0.74

Urinary tract infections and pyelonephritis	2 to 4	0.99	0.78
Urinary tract infections and pyelonephritis	5 to 9	1.00	0.66
Urinary tract infections and pyelonephritis	10 to 14	0.99	0.62
Urinary tract infections and pyelonephritis	15 to 19	0.99	0.76
Urinary tract infections and pyelonephritis	20 to 24	0.99	0.78
Urinary tract infections and pyelonephritis	25 to 29	0.99	0.81
Urinary tract infections and pyelonephritis	30 to 39	0.99	0.81
Urinary tract infections and pyelonephritis	40 to 44	0.99	0.81
Urinary tract infections and pyelonephritis	45 to 49	0.99	0.82
Urinary tract infections and pyelonephritis	50 to 54	0.99	0.82
Urinary tract infections and pyelonephritis	55 to 59	0.99	0.83
Urinary tract infections and pyelonephritis	60 to 64	0.98	0.83
Urinary tract infections and pyelonephritis	65 to 69	0.98	0.84
Urinary tract infections and pyelonephritis	70 to 74	0.98	0.84
Urinary tract infections and pyelonephritis	75 to 79	0.98	0.85
Urinary tract infections and pyelonephritis	80 to 84	0.98	0.86
Urinary tract infections and pyelonephritis	85 to 89	0.98	0.87
Urinary tract infections and pyelonephritis	90 to 94	0.98	0.89
Urinary tract infections and pyelonephritis	95 plus	0.98	0.90

Table 4.7.2: Accuracy and AUC score for out-of-sample validation of sepsis models

Model	Age group name	Accuracy	AUC score
Sepsis	Early Neonatal	0.92	0.74
Sepsis	Late Neonatal	0.87	0.80
Sepsis	1-5 months	0.89	0.90
Sepsis	6-11 months	0.89	0.92
Sepsis	12 to 23 months	0.90	0.93
Sepsis	2 to 4	0.90	0.94
Sepsis	5 to 9	0.90	0.93
Sepsis	10 to 14	0.91	0.93
Sepsis	15 to 19	0.96	0.95
Sepsis	20 to 24	0.96	0.96
Sepsis	25 to 29	0.95	0.96
Sepsis	30 to 34	0.94	0.96
Sepsis	35 to 39	0.93	0.95
Sepsis	40 to 44	0.93	0.94
Sepsis	45 to 49	0.94	0.92
Sepsis	50 to 54	0.94	0.90
Sepsis	55 to 59	0.94	0.89
Sepsis	60 to 64	0.94	0.88
Sepsis	65 to 69	0.94	0.88
Sepsis	70 to 74	0.95	0.88

Sepsis	75 to 79	0.95	0.88
Sepsis	80 to 84	0.95	0.89
Sepsis	85 to 89	0.95	0.90
Sepsis	90 to 94	0.95	0.92
Sepsis	95 plus	0.96	0.94

Section 4.7.1: Incidence of infectious syndromes disaggregated by age, sex, location, and year

We estimate incidence for the 11 infectious syndromes that contribute to AMR in this study. Incidence for 8 of these 11 infectious syndromes was estimated by dividing the syndrome deaths with syndrome specific case fatality ratios (CFRs; estimation described in section 5, below) ("meningitis", "bloodstream infections", "lower respiratory infections", "endocarditis", "peritoneal and intra-abdominal infections", "infections of bones, joints, and related organs", "infections of the skin and subcutaneous system", "urinary tract infections and pyelonephritis"). For bloodstream infections, peritonitis, endocarditis, and meningitis, we used unadjusted inpatient-population-based CFRs; the remaining CFR-calculated syndromes we adjusted using outpatient-population-based CFRs to estimate incidence. Lastly, 3 syndromes among the 11 do not use CFRs ("diarrhoea"; "typhoid, paratyphoid, and invasive non-typhoidal Salmonella"; "tuberculosis") but instead use incidence-mortality ratio estimates from the analogous GBD underlying cause to scale these syndrome deaths to incidence; for details see section 9.1.

Section 5: Case fatality ratios[1]

Section 5.1: Input data

Case fatality ratios (CFRs) were modelled for the pathogens and infectious syndromes of interest using all available data detailing the organism responsible for infection, the infectious syndrome, and patient outcome. This included hospital and microbial data, totaling 20.0 million isolates and cases, as shown in table S10 (section 13). We additionally included 52 907 cases from literature sources for meningitis, which had been previously extracted for a systematic review in GBD.

Section 5.2: Data processing

All input data sources were processed as described in sections 6.2.1, 6.2.2, 6.2.4 and section 6.2.8, and pathogens of interest were chosen as described in section 6.2.3. Input data for the CFR models were aggregated based on data source, year, GBD location, and age group (as well as whether the infection was hospital- or community-acquired, in the case of the lower respiratory and urinary tract infection models). We further incorporated a binary covariate denoting whether the data source only included intensive care unit (ICU) patients, for which CFRs were expected to be higher. For lower respiratory, meningitis, and bloodstream infections, for which CFRs could be different in neonates, we modelled the following age groups: neonatal, post-neonatal–5 years, 5–50 years, 50–70 years, and 70 years and older. For all other infectious syndromes, we modelled the following age groups: neonatal–5 years, 5–50 years, 50–70 years, and 70 years and older. In the case of lower respiratory and urinary tract infections, we model community-acquired and hospital-acquired infections separately, with unknown infection origin data being used in both models. All infection records with multiple pathogens (ie "polymicrobial infections") were excluded from our case fatality estimation.

Section 5.3: Modelling overview

Pathogen-specific CFRs were modelled separately by infectious syndrome and were calculated as a function of HAQ Index and age. We used the HAQ Index to extrapolate CFRs determined from the input data, which often had a broad but not comprehensive temporal and geographical scope, to all 204 GBD countries and territories for the years 1990-2021.

The pathogens of interest for each infectious syndrome were determined by prevalence in the data and expert opinion to provide case fatality ratios for the pathogens modelled in our pathogen distribution models (see section 6.2.3). Because each data source generally reported only a subset of the evaluated pathogens, the input data for the pathogens varied in geographical coverage; nearly all pathogens were well reported in high-income areas, but some pathogens were not well represented in the smaller subset of data we collected from low- and middle-income locations.

We modelled CFRs for all syndromes using four degrees of granularity to capture the most detail possible on a syndrome-pathogen-specific basis. Table 5.4.1 has a summary of all models run for CFRs. For all syndromes, we

first ran the least granular "all pathogen" model, in which we included all data, including unspecified pathogens. This model provides a baseline estimate of syndrome-level CFR as a function of age and HAQ Index. Our next level of modelling, referred to as the "family" models, included all datapoints broadly classified as belonging to the four pathogen taxonomical categories we characterized in our analysis: bacteria, virus, fungus, and parasite. The family models estimated case fatality as a function of pathogen family in addition to age and HAQ Index. Unspecified pathogens with a distinct family designation (eg, "Unspecified bacteria") were included in these models. Next, specified pathogens were modelled using an "intercept" model. In this process each specific pathogen received a fixed effect coefficient, in addition to those of age and HAQ Index. Finally, for each pathogen that had data inclusive of all age groups for the given syndrome, we attempted to fit a unique, most granular model, referred to as our "individual" models. Correspondingly, the case-fatalities for each pathogen modelled in the "individual" landscape has unique relationship with age and HAQ Index.

Thus, the four levels of models run for each infectious syndrome were (from most to least granular):

- Individual pathogen models including data for specific pathogens.
- An intercept model including all identified pathogens.
- A family model including all data aggregated to their respective taxonomical categories of bacteria, virus, parasite, or fungus.
- An "all pathogen" model that included data for all pathogens (predictions were generated by HAQ Index and age, without a pathogen-specific term).

Table S2 (section 13) details which CFR model framework was used to assess the pathogens for each infectious syndrome. Whenever needed, the CFR for any bacterial pathogen "not explicitly modelled" was estimated using the "family" model for subsequent steps of our modelling processes.

For some infectious syndromes, the relative deadliness of a pathogen may be strongly determined by either the age of the patient or whether the infection was community- or hospital-acquired. For bloodstream infections, meningitis, and lower respiratory infections, we further separated the under 5 years of age category into neonates (0-27 days) and post neonate to 5 years. As is done for our other modelling processes, we also separate community-acquired and hospital-acquired cases in our CFR models for lower respiratory and urinary tract infections. Because some data sources did not provide enough information to infer whether an infection was community- or hospital-acquired, but still included important information on the relative pathogenesis and the difference in CFRs across varying HAQ indices, infections of unknown origin were included in both the community-acquired and hospital-acquired models for these two syndromes.

Section 5.4: Modelling framework

The data were analysed using a splined binomial regression model structure. The main model can be specified as follows:

$$P(y|n,p) = \binom{n}{y} p^y (1-p)^{n-y} = \binom{n}{y} exp\left(y\, log\left(\frac{p}{1-p}\right) + n\, log(1-p)\right), \quad (5.4.1)$$

$$\text{with parameterization } logit(p) = log\left(\frac{p}{1-p}\right) = \langle x, \beta \rangle.,$$

Where logit is the link function, and $\langle x, \beta \rangle$ is the linear predictor, an example with:

- y_i contains numbers of observed deaths source i
- The $\langle x, \beta \rangle$ covariates:
    - in all models:
        - HAQ Index
        - dummy-coded indicator for age group
        - dummy-coded ICU indicator for data source (1 if data source only compiles information on ICU patients, 0 if a mix between ICU/non-ICU patients)
    - in 'intercept' models only:
        - dummy-coded indicator for pathogen
    - in 'family' models only:
        - dummy-coded indicator for pathogen taxonomic category
    - in models evaluating community/hospital-acquired infection (LRI+, UTI):

        - dummy-coded variable indicating source of infection (1 if unknown source, 0 if community- OR hospital-acquired, depending on whether the model is evaluating community or hospital infections)
    - in models that included either *Streptococcus pneumoniae* and/or *Haemophilus influenzae*:
        - continuous variable(s) indicating the vaccine coverage for a given location-year, which is specific to the pathogen in question (PCV3 for *S. pneumoniae* or Hib3 for *H. influenzae*).

The underlying program used to fit the model (RegMod) is described elsewhere.[28] The program allows specification of splines and/or priors on β.

Prior and spline on β for HAQ Index: There was a high degree of skewness in the distribution of HAQ Index values, with available data belonging predominantly to countries and years with an HAQ Index over 0.7. Without adjustment, initial model results indicated unrealistically high CFRs in low HAQ Index countries relative to high HAQ Index countries in some cases. To attenuate the effect of HAQ Index in these models we implemented a cubic spline with Gaussian and uniform priors on the HAQ Index. The spline allows for control of this variable while maintaining some degree of flexibility, with knots at the minimum and maximum HAQ Index values, and for the 20th and 60th percentile. We used a Gaussian prior with mean 0 and standard diviation 0.1. For the individual models the standard deviation of the prior was increased in a reverse relationship to the proportion of cases available to the model in contrast to the total amount of cases available within the syndrome as, $\frac{w_{pathogen}}{w_{syndrome}}$, to prevent the prior from overcoming the individual syndrome-pathogen HAQ Index effect. We used a uniform prior to constrain the slope of the spline variable to be neutral or negative in relation to HAQ Index.

Prior on β for age groups: For age groups other than neonates, to constrain the value to be negative in relation to the reference age of over 70 a uniform prior $was\ used$.

Spline prior on β for vaccine effect where appropriate: For those models including either *Streptococcus pneumoniae* and/or *Haemophilus influenzae* we applied a cubic spline with knots at the minimum and maximum value of vaccine coverage with a uniform prior was used to constrain the slope to be negative as vaccine coverage increases.

To attenuate the possible effects of our spline and prior conditions on HAQ Index, we implemented a scoring system that would flag an individual model to be removed from the prediction sequence by comparing the relative R^2 metric by aggregate age category with the subsequent R^2 metric from the intercept model for the same syndrome and pathogen. In rare cases where the R^2 was found to be lower in the individual model than the intercept model, the intercept model was used intead.

We produced an initial set of predictions which were vetted by infectious disease experts for unrealistic trends. To the flagged combinations we applied an outlier process and generated a new set of models. To identify outliers, we calculated a standardised weighed residual using, $standardised\ residual_i = (\frac{residual_i}{SD_{residuals}})\sqrt{w_i}$. A maximum of 5% of the data in each age-pathogen-syndrome would be removed in this process.

Table 5.4.1: Number of datapoints and parameters estimated in each case fatality ratio model

**Infectious syndrome**	**Sub-model(s)**	**CFR model type**	**Datapoints (source-location-years)**	**Estimated parameters**
Bone+	3 pathogens	Individual	*	5 to 6
Bone+	-	Intercept	7,841	12
Bone+	-	Family	14,907	6
Bone+	-	All pathogen	14,912	5
BSI	28 pathogens	Individual	*	6 to 8
BSI	-	Intercept	479,410	25
BSI	-	Family	579,325	9
BSI	-	All pathogen	602,559	6
Intra-abdominal	17 pathogens	Individual	*	5 to 6

Intra-abdominal	-	Intercept	21,566	25
Intra-abdominal	-	Family	25,814	8
Intra-abdominal	-	All pathogen	25,814	6
LRI	Community-acquired	Intercept	436,285	41
LRI	Community-acquired	Family	419,853	11
LRI	Community-acquired	All pathogen	467,596	8
LRI	26 pathogens; Community-acquired	Individual	*	6 to 9
LRI	Hospital-acquired	Intercept	450,776	40
LRI	Hospital-acquired	Family	456,207	11
LRI	Hospital-acquired	All pathogen	456,207	8
LRI	26 pathogens; Hospital-acquired	Individual	*	6 to 9
Skin	20 pathogens	Individual	*	5 to 6
Skin	-	Intercept	128,509	27
Skin	-	Family	129,471	7
Skin	-	All pathogen	129,471	6
UTI	15 pathogens; community-acquired	Individual	*	7
UTI	Community-acquired	Intercept	22,493	24
UTI	Community-acquired	Family	24,541	8
UTI	Community-acquired	All pathogen	24,541	7
UTI	12 pathogens; hospital-acquired	Individual	*	7
UTI	Hospital-acquired	Intercept	29,188	21
UTI	Hospital-acquired	Family	30,156	8
UTI	Hospital-acquired	All pathogen	30,156	7

Bone+=infections of bones, joints, and related organs. BSI=bloodstream infections. Intra-abdominal=peritoneal and intra-abdominal infections. LRI=lower respiratory infections and all related infections in the thorax. Skin=infections of the skin and subcutaneous systems. UTI=urinary tract infections and pyelonephritis.

*The number of datapoints for individual models vary substantially by pathogen and are subsets of the datapoints included in the intercept model.

Section 5.5: Predictions and uncertainty

Predictions for CFRs were generated for the years 1980–2021 for each AMR infectious syndrome by country, age group, and pathogen as a function of each country's HAQ Index, vaccine coverage for *S. pneumooniae* and *H. influenzae* (for those two pathogens), assuming mixed ICU/non-ICU patients and, in the case of models for UTI and LRI+, that the infection was community- or hospital-acquired (in contrast to infections of unknown origin). For pathogens with insufficient data to estimate a syndrome-specific CFR, predictions were generated using the "family" CFR associated with the infectious syndrome with pathogens classified as either bacteria, viruses, fungi or parasites. Importantly, all the infectious syndrome CFRs we estimate are independent of underlying cause.

Section 6: Pathogen distribution[1]

Section 6.1: Input data

With this model, we aimed to estimate the distribution of pathogens causing each of our 11 major bacterial infectious syndromes. To get input data for this model, we gathered all available data sources described in section 2 that meet the following criteria:

- Sufficient diagnosis (for patient- or admission-level datasets) or sample specimen type (for isolate- or culture-level datasets) information for us to determine the infectious syndrome.
- Information on which pathogen(s) caused the infection or which pathogen(s) were detected in an infectious sample, as determined through culture or genomic-based methods.
- Did not have a strongly biased sampling framework across pathogens (for example, did not deliberately sample until 100 cases of every pathogen of interest had been obtained).

The input data source types that met these criteria were:

- Multiple causes of death data
- Hospital discharge
- Linkage data
- Microbial data with and without outcome information
- Literature studies from the aetiology literature reviews
- Mortality surveillance (Child Health and Mortality Prevention Surveillance [CHAMPS])

From these sources combined, there were a total of 20.5 million isolates and cases. Table S3 (section 13) provides a detailed breakdown of this total by pathogen and syndrome.

Section 6.2: Data processing

Section 6.2.1: Extraction and standardisation

We extracted and standardised the location, year, age, sex, diagnoses, specimen type, pathogens, and hospital- and community-acquired (HAI and CAI) status of each record in every dataset. HAI or CAI status in microbial data was determined as described in section 2.3, while in MCoD, hospital discharge, and linkage data, a record was considered CAI if the infectious syndrome was the primary or underlying diagnosis and HAI otherwise, as described in section 4. These datasets report a variety of metrics, including deaths, admissions, cases, cultures, and isolates. While these metrics are not completely comparable (for example, a single patient may often have multiple cultures taken during a single hospital admission), we chose to standardise them into two categories: "deaths," for any unit associated with an outcome of death, and "cases," for any unit regardless of outcome. We assigned a unique identifier, sample ID, to track each unique unit of analysis whenever a dataset included enough line-level data to make this possible. We did not track the relationship between sample ID and patient or admission, in many cases because this was not possible; an improvement to future analyses may be to track this information and account for multiple isolates or cultures from a single admission. The majority of the data informing culprit pathogen were from microbiological analysis of various isolates, but we also considered antigen testing, such as the urinary pneumococcal antigen, and polymerase chain reaction (PCR)-based testing when assigning the pathogen responsible for infection.

Section 6.2.2: Assigning infectious syndrome

After standardising the data, we mapped every sample ID or tabulated figure in the data to infectious syndrome based on its diagnoses and specimen type. Infectious syndrome was assigned first based on any diagnosis associated with a given sample ID or tabulated figure. For sample IDs or tabulated figures with multiple diagnoses and/or an underlying diagnosis, we followed the rules laid out in section 4 for assigning infectious syndrome based on multiple causes. If a dataset contained no diagnoses or the diagnoses provided no information on infectious syndrome, we assigned infectious syndrome based on specimen type (table 6.2.2.1). This is an imprecise method because a patient may have a sample taken from an organ system that is not the site of their primary infection (most commonly from the blood). Finally, if neither diagnosis nor specimen information provided information on infectious syndrome, we assigned infectious syndrome based on pathogen for a select number of pathogens (table 6.2.2.2).

Table 6.2.2.1: Syndrome assignment based on standardised specimen types

Standard specimen	Assigned to syndrome
Blood	Bloodstream infections
Bone & joint	Infections of bones, joints, and related organs
Cerebrospinal fluid	Meningitis
Endocardial	Endocarditis
Intra-abdominal	Peritoneal and intra-abdominal infections
Lower respiratory	Lower respiratory infections and all related infections in the thorax
Peritoneal	Peritoneal and intra-abdominal infections
Rectal/stool	Diarrhoea
Skin	Infections of the skin and subcutaneous systems
Urinary tract	Urinary tract infections and pyelonephritis
Catheter	Other unspecified site infections, not analysed*
Eye	Eye infections, not analysed*
Genital	Genital infections, not analysed*
Oral	Oral infections, not analysed*
Pericardial	Carditis, myocarditis, and pericarditis, not analysed*
Upper respiratory	Upper respiratory infections, not analysed*
Other and unspecified specimens	Other unspecified site infections, not analysed*

*Please refer to Section 6.4.2 for detail on why certain syndromes were excluded from the analysis

Table 6.2.2.2: Syndrome assignment based on pathogen for entries lacking diagnostic and specimen information

Pathogen	Assigned to syndrome
Salmonella Typhi	Typhoid, paratyphoid, and invasive non-typhoidal Salmonella
Salmonella Paratyphi	Typhoid, paratyphoid, and invasive non-typhoidal Salmonella
Salmonella Typhi or Paratyphi	Typhoid, paratyphoid, and invasive non-typhoidal Salmonella
Non-typhoidal *Salmonella* species	Typhoid, paratyphoid, and invasive non-typhoidal Salmonella
Mycobacterium tuberculosis	Tuberculosis
Neisseria meningitidis	Meningitis

Section 6.2.3: Selecting pathogens for estimation

For each infectious syndrome, we assessed either the 25 most prevalent pathogens by case count in our raw data, or all pathogens with 300 or more cases, whichever number was smaller. In addition to the n pathogens for a given syndrome that we estimate explicitly, we also included an "other specified pathogens" category for every infectious syndrome, to which we mapped all other aetiologies identified in the data. Thus, for a given infectious syndrome, the set of estimated pathogens is mutually exclusive and collectively exhaustive of all possible aetiologies.

For a list of pathogens covered in each infectious syndrome model, please refer to table 6.3.1.2.

Section 6.2.4: Pathogen redistribution and aggregate pathogen categories

A substantial portion of our input data identified pathogens by their genus alone, without species-level identification. For example, ICD code J15.2 designates "Pneumonia due to *Staphylococcus*," without distinguishing whether the

infection was due to Staphylococcus aureus, or another species within the genus. In total, there were more than 400 000 isolates in the input data that lacked species detail. To express these data with the species-level detail required for our analysis, we split these cases into target species and a residual “Genus others” category using a set of proportions generated from all of the available literature, surveillance, and microbial data reporting species detail. We stratified these proportions based on World Bank income level, aggregate age groups, year bins, and infectious syndrome. When there were insufficient input data to inform splitting proportions for a given level of detail, we collapsed (in order) income level, year bin, and then age group to apply a more crude proportion. We did not collapse down infectious syndrome, and if there remained no matching proportions for the row in question, we dropped this row from use in our models.

Pathogen aggregates and distinct species were selected by clinical experts who identified both organisms of most AMR concern and those responsible for characteristic disease (eg, *Treponema pallidum*). The aggregate pathogen categories we used both to redistribute pathogens and for our modelling are reported in table 6.2.4.1.

Table 6.2.4.1: Aggregate pathogen categories for pathogen distribution and case fatality

**Aggregate category**	**Classification**	**Examples**
Acinetobacter others	Non-baumannii *Acinetobacter*	*Acinetobacter calcoaceticus*, *Acinetobacter haemolyticus*, *Acinetobacter lwoffii*
Clostridium others	Non-*difficile*/*tetani Clostridium*	*Clostridium botulinum*, *Clostridium perfringens*, *Clostridium septicum*
Enterococcus others	Non-*faecalis*/*faecium Enterococcus*	*Enterococcus casseliflavus*, *Enterococcus durans*, *Enterococcus gallinarum*
Fungi others	Fungal pathogens not otherwise represented in: *Aspergillus* spp., *Candida* spp., *Cryptococcus* spp., Dermatophytes, *Histoplasma* spp., or *Pneumocystis* spp.	*Blastomyces* spp., *Coccidioides* spp., *Mucor* spp., *Paracoccidioides* spp., *Rhizomucor* spp., *Sporothrix* spp.
Gram-negative others	Gram-negative bacteria not otherwise represented in: *Acinetobacter* spp., *Aeromonas* spp., *Bordetella pertussis*, *Burkholderia* spp., *Campylobacter* spp., *Chlamydia* spp. *Citrobacter* spp., *Enterobacter* spp., *Escherichia* spp., *Haemophilus* spp., *Klebsiella* spp. *Legionella* spp., *Leptospira* spp., *Morganella* spp., *Neisseria* spp., *Proteus* spp., *Pseudomonas* spp., *Salmonella* spp., *Serratia* spp., *Shigella* spp., or *Vibrio cholerae*	*Bacteroides* spp., non-pertussis *Bordetella* spp., *Brucella* spp., *Moraxella* spp., *Pasteurella* spp., *Prevotella* spp., *Providencia* spp., *Stenotrophomonas* spp., non-*cholerae Vibrio* spp., *Yersinia* spp.
Gram-positive others	Gram-positive bacteria not otherwise represented in: *Actinomyces* spp., *Clostridium* spp., *Corynebacterium diphtheriae*, *Enterococcus* spp., *Helicobacter* spp., *Listeria* spp., *Mycobacterium* spp., *Staphylococcus* spp., *Streptococcus* spp., *Treponema pallidum*	*Bacillus* spp., *Lactobacillus* spp., *Micrococcus* spp., *Peptostreptococcus* spp., *Propionibacterium* spp., *Rothia* spp.
Klebsiella others	Non-pneumoniae *Klebsiella*	*Klebsiella aerogenes*, *Klebsiella granulomatis*, *Klebsiella oxytoca*
Mycobacterium others	Non-*tuberculosis*/*leprae Mycobacterium*	*Mycobacterium avium*, *Mycobacterium intracellulare*, *Mycobacterium fortuitum*
Pseudomonas others	Non-*aeruginosa Pseudomonas*	*Pseudomonas fluorescens*, *Pseudomonas luteola*, *Pseudomonas putida*
Streptococcus others	Non-Group A/Group B/*pneumoniae Streptococcus*	*Streptococcus anginosus*, *Streptococcus bovis*, *Streptococcus mitis*, *Streptococcus mutans*, *Streptococcus salivarius*
Virus others	Viral pathogens not otherwise represented in: adenovirus, astrovirus, Chikungunya virus, coronavirus, cytomegalovirus, dengue, Ebola, enterovirus, Epstein Barr virus, hepatitis, herpes virus, HIV, HPV, influenza, measles, molluscum contagiosum virus, mumps, norovirus, rabies,	Human metapneumovirus, orthopoxvirus, parapoxvirus, picornaviruses, poliovirus, retrovirus, rubella

	respiratory syncytial virus, rotavirus, varicella zoster virus, West Nile virus, yellow fever, Zika virus	

Section 6.2.5: Contaminants, opportunistic pathogens, and no aetiology detected

Some pathogens, such as coagulase-negative *Staphylococci* or miscellaneous gram-positive species in urinary tract infections, cause disease so rarely or are so commonly contaminants that we considered them to be contaminants, unlikely to be the true cause of disease. Other pathogens—such as *Cryptococcus*, *Pneumocystis*, and *Toxoplasma*—are opportunistic and are commonly found in immunocompromised patients. In some of such cases, HIV is responsible for the underlying infection and should be the attributable pathogen. More broadly, opportunistic pathogens are prone to oversampling among young and middle-aged adults with immunocompromising conditions as these infections occur in a patient population that is typically more frequently monitored and integrated with local health care systems. Correspondingly, we dropped all contaminants and opportunistic pathogens as reported in table 6.2.5.1 from the analysis, as well as any record listed by treating clinicians in the data as a contaminant.

Table 6.2.5.1: Contaminants and opportunistic pathogens not assessed in pathogen distribution models

**Contaminant/opportunistic pathogen**	**Excluded syndromes**
Adenovirus	Lower respiratory infections
Candida spp.	Lower respiratory infections
Cryptococcus spp.	Meningitis
Gram-positive others	Urinary tract infections and pyelonephritis
Pneumocystis spp.	Bloodstream infections, infections of bones, joints, and related organs
Staphylococcus saprophyticus	All syndromes except for meningitis, urinary tract infections and pyelonephritis
All other coagulase-negative *Staphylococci*	All syndromes except for meningitis
Toxoplasma spp.	Meningitis

We also dropped from the analysis all records where no pathogen was detected, or the patient diagnosis indicated an unspecified bacterium, virus, fungus, or parasite. This assumes that the distribution of pathogens among cases with known aetiology are the same as those with unknown aetiology; in other words, that the probability of detection is the same for every pathogen. This assumption may break down if certain pathogens are more difficult to detect than others, or in cases where a pathogen is irregularly tested for within a laboratory.

Section 6.2.6: Polymicrobial infections

In our previous analysis for 2019, we attributed more than 5% of the sepsis deaths to "polymicrobial infections," which were generally defined as infections involving two or more bacteria, two or more viruses, or two or more fungi. Classifying polymicrobial infections as such was useful for two main reasons: 1) it allowed us to apply case fatality ratios more accurately for these infections, which were typically more severe, and 2) it provided a sense of how prevalent these infections were for a given infectious syndrome. Due to the aggregated nature of this polymicrobial category, however, we were unable to assess the extent to which AMR might have been involved in these infections, leaving polybacterial disease involving important AMR species (eg, *K. pneumoniae* and *E. coli* co-infection) unaccounted for.

To better represent the burden of AMR in this analysis, we retained all pathogens associated with polymicrobial infections in our input data pipeline as discrete entities. Death and case counts related to polymicrobial infections are now evenly allocated to all constituent pathogens involved in that polymicrobial record. For example, if we had hospital data indicating one person died of pneumonia stemming from an influenza and *S. pneumoniae* co-infection, this record would be expressed as two rows with half a death each, one in which the pathogen was influenza, and the other in which the pathogen was *S. pneumoniae*. As such, the *S. pneumoniae* burden related to this infection is passed down to subsequent models where the involvement of AMR can be determined.

Our new approach has a key limitation. After splitting these infections into their constituent pathogens, they are then pooled with mono-pathogen data from the same demographic strata, and we lose information about which of our records were initially polymicrobial. For sources that provide information on deaths, pooled data including these fractional splits will then be scaled using the mono-pathogen specific case fatality ratio (we exclude all polymicrobial infection records from our case fatality estimation). As polymicrobial infections often represent more severe disease in compromised hosts, the use of the mono-pathogen case fatality is almost certainly an underestimate, and an underestimate of CFR will in turn yield an overestimate of implied incidence. However, because the proportion of data that originated as a polymicrobial record is low for any given pathogen, we anticipate the effect of this overestimation is small. Moreover, our decision to attribute a fractional split rather than the entirety of polymicrobial disease to each underlying pathogen further minimises the contribution of polymicrobial records to our input data for specific pathogens.

As a consequence of this methodological change, we no longer estimate the proportion of infectious disease that is polymicrobial. However, the intended purpose of our work has been to quantify AMR burden, and our new method more effectively captures specific pathogens (be they resistant or not) from polymicrobial disease. Future work will attempt to more appropriately quantify the case fatality ratios of these infections to minimise the degree to which the incidence of their constituent pathogens is overestimated.

Section 6.2.7: Estimating unbiased other categories

One of the central challenges of estimating pathogen distributions was that not every data source tested for or reported every possible aetiology of a given infectious syndrome. For example, many literature studies on the aetiologies of meningitis only report on bacterial aetiologies. Some surveillance systems, like the US Centers for Disease Control and Prevention (CDC) Active Bacterial Core surveillance (ABCs), only collect data on certain pathogens of interest. Only certain pathogens are referenced explicitly in the International Classification of Diseases (ICD), limiting which pathogens can be identified from ICD-based data types like MCoD and hospital discharge. Finally, some datasets reported only a subset of the pathogens that we are interested in for a given infectious syndrome, reporting the remaining aetiologies in an aggregate "other" category. These practices have led to inconsistencies in the "other" categories across data sources leading to datasets either over or under-reporting "other."

To address this problem, we maintained a list of data sources that we believe have sufficient testing and reporting to give unbiased estimates of "other" for all syndromes. We dropped any data on "other" that did not come from these data sources. These data sources all had a complete sampling framework (eg, they do not limit the scope of aetologies that they test for) and reported their results without any deliberate aggregation. While we believe this list provided an accurate starting place for the estimation of "other", future work to improve this method would involve a more detailed analysis of sampling framework and reporting categories in each dataset, specific to each infectious syndrome.

There were three major exceptions to this method for handling "other specified pathogens." First, determining the pathogenic aetiology of LRI with microbiology represents challenges that have been well described previously.[29,30] In order to account for this limitation, we utilised a vaccine probe design to inform the *Streptococcus pneumoniae* cause fraction of LRI, consistent with the approach used in the GBD aetiology estimation process.[31,32] In brief, we extracted the vaccine efficacy of the pneumococcal vaccine against all pneumonia from 18 vaccine probe studies with randomised-control trial, before-after, and cohort designs among children and adults. We then calculated the PAF of pneumonia due to *S. pneumoniae* in each study ($Strep\ Base\ PAF$) based on these vaccine efficacies ($VE_{all\ pnuemonia}$), the vaccine efficacy of pneumococcal vaccine against vaccine-type pneumococcal pneumonia as pooled from three studies (two in children and one in adults) (VE_{vtpp}), the percentage of the population covered by the pneumococcal vaccine as modelled in GBD (100% for RCTs) (Cov_{PCV3}),[32] and the percent of serotypes covered by the vaccine[33] ($Cov_{serotype}$) (equation 6.2.6.1). We modelled a global age-specific PAF for *S. pneumoniae* based on these data in the MR-BRT environment and finally adjusted this PAF based on the vaccine coverage in children in every GBD location in 2019 and optimal vaccine efficacy in children ($Strep\ Final\ PAF$) (equation 6.2.7.2). In adults (age 5+), we assumed the effects of vaccination on adults would be primarily indirect from vaccination in children, and included an adjustment factor on the vaccine efficacy to account for this, derived from Grijalva et al.[34]

$$Strep\ Base\ PAF = \frac{VE_{all\ pneumonia}}{VE_{vtpp}Cov_{PCV3}Cov_{serotype}} \qquad (6.2.7.1)$$

$$Strep\ Final\ PAF = \frac{Strep\ Base\ PAF\left(1 - Cov_{PCV3}Cov_{serotype}VE_{PCV3\ Optimal}\right)}{1 - (Strep\ Base\ PAF)Cov_{PCV3}Cov_{serotype}VE_{PCV3\ Optimal}} \quad (6.2.7.2)$$

In this vaccine probe analysis, $(1 - Strep\ Final\ PAF)$ is not consistent with the "other" category in our model, since it includes all non-*S. pneumoniae* aetiologies. We retained all of the data from the vaccine probe analysis as two categories, *S. pneumoniae* and "not *S. pneumoniae*" and addressed the inconsistencies between them and our other data using our modelling framework.

The second major exception involves several literature studies on the proportion of neonatal bacterial meningitis caused by *Streptococcus agalactiae* (Group B *Streptococcus*; GBS). We found that these literature studies were important to our estimation of the pathogen distribution of neonatal meningitis, which is distinct from other age groups because of its high proportion of GBS. However, these studies either only reported or were only extracted with two categories, GBS and "other bacterial, not GBS." We retained both these categories and addressed the inconsistencies between them and our other data by encoding "other bacterial, not GBS" as a composite observation in our modelling framework, as described in section 6.3.1.

The final exception was made for non-specific aggregate designations found in our ICD-coded data. For instance, ICD code A41.5 identifies "Sepsis due to other Gram-negative organisms," with "other" in this case, representing all Gram-negative organisms not elsewhere described by a candidate ICD code. In the context of our analysis, this category could have represented *Acinetobacter baumannii*, *Acinetobacter* others, *Citrobacter* spp., *Klebsiella pneumoniae*, *Klebsiella* others, *Morganella* spp., or *Proteus* spp., none of which have an ICD10 code for BSI, or our "Gram-negative others" category. Correspondingly, ICD code A41.5 was encoded in our work as an amalgam of all the above pathogens/pathogen categories, and we addressed the inconsistencies between this group and our other data using our modelling framework. Similar adjustments were made for ICD code J15.6 "Pneumonia due to other aerobic Gram-negative bacteria," though with a different set of aggregate pathogens as the candidate list for Gram-negative species is slightly different for LRI and BSI in ICD.

Section 6.2.8: Age-sex splitting

We standardised age and sex across all datasets to the following most-detailed groups using the GBD causes of death age-sex splitting algorithm for age:[2] 0–6, 7–27, 1-5 months, 6-11 months, 12-23 months, 2-4 years, 5–9, 10–14, 15–19, 20–24, 25–29, 30–34, 35–39, 40–44, 45–49, 50–54, 55–59, 60–64, 65–69, 70–74, 75–79, 80–84, 85–89, 90–94, 95+ years; and sex: male and female. This algorithm is based on the assumption that age-sex pattern of the death or case rate for a given infectious syndrome or pathogen is inherent to the pathology of the disease and is therefore constant across location and year.

To apply the algorithm, we first calculated distinct age-sex weights for every infectious syndrome and pathogen, separately for deaths and cases. These weights are the aggregate death and case rates across all datasets that report every detailed age-sex group. If we were to use a dataset that only reported some of the detailed age-sex groups, then the unreported age-sex groups would be biased downwards in the weight distribution. Calculating rates based on raw data counts could lead to extremely low rates, since we are typically comparing the entire population of a given location-year to deaths or cases captured within a single study, hospital, or surveillance system. Since the age-sex splitting algorithm only relies on the relative distribution of the weights, however, rather than their absolute level, this bias ultimately had no effect. For any infectious syndrome or pathogen combination for which we did not have enough data to create plausible age-sex weights, we used a set of all-pathogen weights for that infectious syndrome instead.

Since we split cases and deaths independently, it is possible for a detailed age-sex group produced by the splitting algorithm to contain fewer cases than deaths. When this occurred, we capped the deaths to match the cases. For future improvement, a possible solution to this problem may be to split deaths, survivors, and cases without indication of outcome separately.

Section 6.2.9: Standardising measures

The input data sources reported a variety of combinations of measures, including some that reported deaths only, some that reported cases only, and some that reported both cases and deaths. In order to standardise these measures to cases, we estimated infectious syndrome- and pathogen-specific CFRs (see section 5) and used these CFRs to convert all deaths-only datasets to cases. All modelling was done in case space.

Several of our microbial databases came exclusively from ICUs and were therefore heavily biased towards severe illness. In order to mitigate this bias, we dropped all information on cases in ICU-only datasets and recalculated implied cases based on reported deaths and our CFRs. No similar adjustment was made to attempt to account for biases between hospitalised and un-hospitalised populations, although we did account for HAI versus CAI for two infectious syndromes—LRI and UTI—within our modelling framework.

Section 6.3: Modelling framework

Section 6.3.1: Overview

To model the distribution of pathogens for each infectious syndrome, we developed a method for the multinomial estimation of partial and compositional observations (MEPCO). We assumed that the aetiologies of a given infectious syndrome followed a multinomial distribution. Due to inconsistencies in which pathogens are tested for and reported by different data sources, each data source contained partial observations of the possible outcomes of the underlying multinomial distribution. Certain data sources like the vaccine probe estimates and the GBS neonatal meningitis studies represent compositional observations, where pathogens like "not *S. pneumoniae*" and "other bacterial, not GBS" represent aggregates of more detailed pathogens.

In order to use both partial and compositional data, we constructed a network model with the dependent variable as the log ratio of cases between different pathogens and estimated over a flexible parameterisation of multinomial parameters using a maximum likelihood approach. Consider a given infectious syndrome with a multinomial distribution of n mutually exclusive, collectively exhaustive aetiologies with probabilities $p = (p_1, \dots, p_n)$, so that each $p_j \in (0,1)$ and $\sum_j p_j = 1$. The likelihood of an observation of $c = (c_1, \dots, c_n)$, where c_j = number of cases of pathogen j in a total sample of N infections ($\sum_j c_j = N$), is:

$$P(c|p) = N! \prod_{j=1}^{n} \frac{p_j^{c_j}}{c_j!} \qquad (6.3.1.1)$$

We modelled the probabilities using a composition of a link function with a linear predictor:

$$p_{i,j} = exp\left(x_{i,j}^T \beta_j\right) \qquad (6.3.1.2)$$

for observations i, a vector of covariates $x_{i,j}$, and a vector of coefficients β_j for each pathogen j. However, we did not observe these probabilities directly. Rather, we observed ratios between sums of these probabilities, which reduce to ratios between sums of cases within each study. These observations therefore take the form:

$$y_i = \frac{cases\ of\ pathogen\ A}{cases\ of\ pathogen\ B} = \frac{\sum_{j=1}^{n} w_{i,j}^a\, exp\left(x_{i,j}^T \beta_j\right)}{\sum_{j=1}^{n} w_{i,j}^b\, exp\left(x_{i,j}^T \beta_j\right)} \qquad (6.3.1.3)$$

where $w_{i,j}^a$ is a weight of 0 or 1 that selects the mutually exclusive, collectively exhaustive most-detailed pathogens that make up observed pathogen A, which may be a composite observation. For example, for the "other bacterial, non-GBS" pathogen, $w_{i,j}$ would be 1 for *S. aureus*, *S. pneumoniae*, *H. influenzae*, *N. meningitidis*, *Listeria monocytogenes*, *K. pneumoniae*, *E. coli*, and other pathogens and 0 for GBS and virus. We dropped all observations where either the numerator or denominator had 0 observed cases in order to make this calculation and a forthcoming log transform possible. This may bias the model towards overestimating less common pathogens.

Table 6.3.1.1 shows the covariates used for each infectious syndrome model; a typical specification included an intercept term, HAQ Index, a categorical age group dummy for large age bins, and any relevant vaccine coverage proportions by country. It is not possible to infer all coefficients β_j from the observations since they are all relative. However, if we fix all of the coefficients for one pathogen to 0 as a reference group, then we obtain a well-posed inverse problem, as long as there is enough data to estimate the remaining coefficients. Without loss of generality, we assumed $\beta_1 = 0$ for all elements and obtain estimates of the remaining $\beta_2, \dots, \beta_n$ by minimising the sum of the residuals between log-transformed observations y and corresponding log-transformed predictions from equation 6.3.1.3:

$$\min_{\beta_2,\dots,\beta_n} f(\beta) := \sum_i \frac{1}{\sigma_i^2}\left[ln(y_i) - ln\left(\sum_{j=1}^{n} w_{i,j}^a\, exp\left(x_{i,j}^T \beta_j\right)\right) + ln\left(\sum_{j=1}^{n} w_{i,j}^b\, exp\left(x_{i,j}^T \beta_j\right)\right)\right]^2 \qquad (6.3.1.4)$$

where σ_i^2 are variances corresponding to the data points. Equation 6.3.4 is a nonlinear likelihood minimisation problem that that we optimised using a standard implementation of the Gauss-Newton method.[35] We then re-normalised the optimal coefficients to obtain final predictions of the probabilities of each pathogen:

$$p_{i,j} = \frac{exp\left(x_{i,j}^T \beta_j\right)}{\sum_{\hat{j}} exp\left(x_{i,\hat{j}}^T \beta_{\hat{j}}\right)} \quad (6.3.1.5)$$

To quantify the uncertainty of this estimate, we used asymptotic statistics to obtain the posterior distribution of $(\beta_2, \dots, \beta_n)$. Specifically, using the Gauss-Newton Hessian approximation gave us the asymptotic information matrix for all β_j except for the reference pathogen, allowing us to sample draws of $\beta = (\beta_1 = 0, \beta_2, \dots, \beta_n)$. For each β draw and given feature x, we obtained a corresponding draw of p using equation 6.3.1.5.

Finally, to convert $p_{i,j}$ for a given demographic group i from case space to deaths space, we transformed using our CFR estimate for demographic i:

$$\mathrm{p}_{\mathrm{i,j}}^{\mathrm{deaths}} = \frac{\mathrm{p}_{\mathrm{i,j}} \times \mathrm{CFR}_{\mathrm{i}}}{\sum_{\hat{\mathrm{j}}} \mathrm{p}_{\mathrm{i},\hat{\mathrm{j}}} \times \mathrm{CFR}_{\mathrm{i}}} \quad (6.3.1.6)$$

Table 6.3.1.1: Pathogens assessed, pathogen distribution model covariates, and age groups for each infectious syndrome

**Infectious syndrome**	**Pathogens assessed**	**Model covariates**	**Age groups**
Bloodstream infections	*Acinetobacter baumannii*, *Acinetobacter* others, *Burkholderia spp.*, *Candida* spp., *Citrobacter* spp., *Enterobacter* spp., *Enterococcus faecalis*, *Enterococcus faecium*, *Escherichia coli*, Gram-negative others, *Haemophilus influenzae*, *Klebsiella* others, *Klebsiella pneumoniae*, *Morganella* spp., *Mycobacterium* others, *Neisseria meningitidis*, *Proteus* spp., *Pseudomonas aeruginosa*, *Serratia* spp. *Staphylococcus aureus*, Group A *Streptococcus*, Group B *Streptococcus*, *Streptococcus* others, *Streptococcus pneumoniae*, Virus others	HAQ Index,[15] age group, age-standardised proportion of intravenous drug use,[24] Proportion of people who as infants were vaccinated with PCV,[26] Proportion of population age 15 or younger who received PCV vaccine, mean temperature, inpatient utilization envelope	Neonatal, Post-neonatal–5, 5–50, 50–70, 70+
Infections of bones, joints, and related organs	*Acinetobacter baumannii*, *Enterobacter* spp. *Enterococcus faecalis*, *Enterococcus faecium*, *Escherichia coli*, Gram-negative others, *Gram-positive* others, *Klebsiella* others, *Klebsiella pneumoniae*, *Morganella* spp., *Proteus* spp., *Pseudomonas aeruginosa*, *Serratia* spp., *Staphylococcus aureus*, Group A *Streptococcus*, Group B *Streptococcus*, *Streptococcus* others, *Streptococcus pneumoniae*	HAQ Index, age group, Proportion of population age 15 or younger who received PCV vaccine	Under 5, 5–50, 50–70, 70+
Endocarditis	*Candida* spp., *Escherichia coli*, *Klebsiella pneumoniae*, *Pseudomonas aeruginosa*, *Staphylococcus aureus*, Group A *Streptococcus*	HAQ Index, age group	Neonatal, Post-neonatal–5, 5–50, 50–70, 70+

Diarrhoea	Adenovirus, *Aeromonas* spp., Amebiasis, *Campylobacter* spp.., *Clostridium difficile*, *cryptosporidium*, enteropathogenic *Escherichia coli*, enterotoxigenic *Escherichia coli*, non-typhoidal *Salmonella*, norovirus, rotavirus, *Shigella* spp.., *Vibrio cholerae*	Not modelled here. GBD diarrhoea aetiology estimates are used.	GBD most detailed age groups
Lower respiratory infections	*Acinetobacter baumannii*, *Acinetobacter* others, *Aspergillus* spp., *Chlamydia* spp., *Citrobacter* spp., *Enterobacter* spp., *Escherichia coli*, Fungi others, Gram-negative others, *Haemophilus influenzae*, Influenza virus, *Klebsiella* others, *Klebsiella pneumoniae*, *Legionella* spp., *Morganella* spp., *Mycobacterium* others, *Mycoplasma* spp., *Proteus* spp., *Pseudomonas aeruginosa*, Respiratory syncytial virus, *Serratia* spp., *Staphylococcus aureus*, Group A *Streptococcus*, Group B *Streptococcus*, *Streptococcus pneumoniae*	HAQ Index, Proportion of people who as infants were vaccinated with PCV,[26] Proportion of population age 15 or younger who received PCV vaccine, Proportion of people who as infants were vaccinated with Hib3 vaccine,[26] Proportion of population age 15 or younger who received Hib3 vaccine, age group, HAI/CAI	Neonatal,<br>Post-neonatal–5,<br>5–50,<br>50–70,<br>70+
Meningitis	*Acinetobacter baumannii*, *Candida* spp., *Escherichia coli*, Fungi others, *Haemophilus influenzae*, *Klebsiella pneumoniae*, *Listeria* spp., *Neisseria meningitidis*, non-polio enteroviruses, *Pseudomonas aeruginosa*, coagulase-negative *Staphylococcus*, *Staphylococcus aureus*, Group B *Streptococcus*, *Streptococcus* others, *Streptococcus pneumoniae*, Virus others	HAQ Index, Proportion of people who as infants were vaccinated with PCV,[26] Proportion of population age 15 or younger who received PCV vaccine, Proportion of people who as infants were vaccinated with Hib3 vaccine,[26] Proportion of population age 15 or younger who received Hib3 vaccine, age group, proportion of population covered by '10-'15 MenAfriVac rollout[36]	Neonatal,<br>Post-neonatal–5,<br>5-50,<br>50-70,<br>70+
Peritoneal and intra-abdominal infections	*Acinetobacter baumannii*, *Acinetobacter* others, *Actinomyces* spp., *Aeromonas* spp., *Burkholderia* spp., *Candida* spp., *Chlamydia* spp., *Citrobacter* spp., *Entamoeba histolytica*, *Enterobacter* spp., *Enterococcus faecalis*, *Enterococcus faecium*, *Enterococcus* others, *Escherichia coli*, Gram-negative others, *Klebsiella* others, *Klebsiella pneumoniae*, *Morganella* spp. *Proteus* spp., *Pseudomonas aeruginosa*, *Pseudomonas* others, *Serratia* spp., *Staphylococcus aureus*, *Streptococcus* others, *Streptococcus pneumoniae*	HAQ Index, age group	Under 5,<br>5–50,<br>50–70,<br>70+
Infections of the skin and	*Acinetobacter baumannii*, *Actinomyces* spp., *Aeromonas* spp., *Citrobacter* spp., *Clostridium* others, *Enterobacter* spp., *Enterococcus faecalis*, *Enterococcus*	HAQ Index, age group	Under 5,<br>5–50,<br>50–70,<br>70+

subcutaneous systems	*faecium*, *Escherichia coli*, Gram-negative others, *Gram-positive* others, *Klebsiella* others, *Klebsiella pneumoniae*, *Morganella* spp., *Mycobacterium* others, non-polio enteroviruses, *Proteus* spp., *Pseudomonas aeruginosa*, *Serratia* spp., *Staphylococcus aureus* Group A *Streptococcus*, Group B *Streptococcus*, *Streptococcus* others, *Streptococcus pneumoniae*, Virus others		
Urinary tract infections and pyelonephritis	*Acinetobacter baumannii*, *Acinetobacter* others, *Aeromonas* spp., *Burkholderia* spp., *Candida* spp., *Citrobacter* spp., *Enterobacter* spp., *Enterococcus faecalis*, *Enterococcus faecium*, *Enterococcus* others, *Escherichia coli*, *Fungi* others, Gram-negative others, *Klebsiella* others, *Klebsiella pneumoniae*, *Morganella* spp., *Proteus* spp., *Pseudomonas aeruginosa*, *Pseudomonas* others, *Serratia* spp., *coagulase-negative Staphylococcus*, *Staphylococcus aureus*, Group B *Streptococcus*, *Streptococcus* others, *Streptococcus pneumoniae*	HAQ Index, age group, sex, Proportion of population age 15 or younger who received PCV vaccine,[26] HAI/CAI	Under 5, 5–50, 50–70, 70+

Group A *Streptococcus* = *Streptococcus pyogenes*. Group B *Streptococcus* = *Streptococcus agalactiae*. HAQ Index = Healthcare Access and Quality Index. HAI/CAI = hospital-acquired infection/community-acquired infection.

Table 6.3.1.3: Number of data points and parameters in each pathogen distribution model

Infectious syndrome	Number of data points	Number of parameters
Bloodstream infections	147,310	286
Infections of bones, joints, and related organs	5,657	114
Endocarditis	3,187	35
Lower respiratory infections	197,345	286
Meningitis	38,205	187
Peritoneal and intra-abdominal infections	34,812	130
Infections of the skin and subcutaneous systems	32,674	130
Urinary tract infections and pyelonephritis	63,651	208

Section 6.3.2: Model priors

The network regression with covariates framework allowed us to use partial and composite data that reported on one or only a few pathogens, or that reported multiple pathogens aggregated together. Networks, however, can be unstable with sparse data and stable estimates have in some cases required the use of non-diffuse Bayesian priors in these models. In particular, we imposed Gaussian priors with mean 0 and non-zero variance on all coefficients except intercepts, to bias the model away from spurious effects driven by data sparsity. These priors were based on expert opinion and designed with the guiding philosophy that they should minimally alter model results and serve to inhibit overfitting. Given that the various models differed tremendously with respect to their quantity of input data, the prior standard deviation needed to achieve this effect was lower for some infectious syndromes than others. Even

after applying these adjustments, a small set of coefficients still had an outsized and clinically implausible effect on the result; for instance, a pathogen representing 10% of the syndrome distribution in a low healthcare access setting and 65% of the distribution is a high healthcare access setting. In such cases, the standard deviation for the Gaussian prior for that coefficient was lowered in accordance with expert review to achieve a more reasonable fit. Table 6.3.2.1 provides a list of these priors, which could be improved with further empirical validation in the future.

Table 6.3.2.1: Gaussian prior standard deviations for non-intercept coefficients for each pathogen distribution model

**Infectious syndrome**	**Gaussian prior standard deviation**	**Exceptions**
BSI	0.1	0.02 for A. baumannii/HAQ Index coefficient
Bone+	0.4	0.15 for all pathogens/HAQ Index coefficient
Endocarditis	0.5	
Meningitis	0.2	0.02 for Group B Streptococcus/HAQ Index coefficient<br>0.02 for Non-polio enteroviruses/HAQ Index coefficient
LRI+	0.1	0.02 for Mycobacterium others/HAQ Index coefficient
Peritonitis+	0.3	
Skin	0.5	
UTI	0.2	0.27 for all pathogens/sex coefficient

BSI = Bloodstream infections. Bone+ = Infections of bones, joints, and related organs. LRI = Lower respiratory infections. Peritonitis+ = Peritoneal and intra-abdominal infections. Skin = Infections of the skin and subcutaneous systems. UTI = Urinary tract infections and pyelonephritis.

Additionally, there were certain covariates that we assumed would have a monotonic relationship with the proportion of a syndrome attributable to a given pathogen. For example, the proportion of people who as infants were vaccinated with the pneumococcal conjugate vaccine (PCV) was anticipated to be negatively associated with the proportion of LRI that was caused by *Streptococcus pneumoniae*. Such monotonic relationships were enforced by design using uniform priors as outlined in Table 6.3.2.2. Because the network model estimates a coefficient for every combination of covariate and pathogen, covariate estimates for the effect of PCV vaccination on the proportion of disease due to non-pneumococcal pathogens is also quantified. To ensure that these pathogens were not shaped by some unmeasured variable that trended with PCV vaccination, we employed a more restrictive Gaussian prior on these less-directly related pathogens ("less-directly related" because, if the proportion of pneumococcus is to decrease, other pathogens must correspondingly increase in relative space).

Table 6.3.2.2: Pathogen distribution model covariates with uniform priors

**Covariate**	**Pathogen**	**Uniform Prior (Association)**	**Gaussian prior standard deviation for other pathogens**	**Syndromes**
Proportion of people who as infants were vaccinated with PCV	*S. pneumoniae*	(-inf, 0]<br>(Negative)	0.01	BSI, LRI, Meningitis
Proportion of population age 15 or younger who received PCV vaccine	*S. pneumoniae*	(-inf, 0]<br>(Negative)	0.01	BSI, Bone+, LRI, Meningitis, UTI
Proportion of people who as infants were vaccinated with Hib3 vaccine	*H. influenzae*	(-inf, 0]<br>(Negative)	0.01	LRI, Meningitis

Proportion of population age 15 or younger who received Hib3 vaccine	*H. influenzae*	(-inf, 0] (Negative)	0.01	LRI, Meningitis
Proportion of population covered by '10-'15 MenAfriVac rollout	*N. meningitidis*	(-inf, 0] (Negative)	0.02	Meningitis
Mean temperature	*A. baumannii*	[0, inf) (Positive)	0.0001	BSI

BSI = Bloodstream infections. Bone+ = Infections of bones, joints, and related organs. LRI = Lower respiratory infections. UTI = Urinary tract infections and pyelonephritis.

The role of mean temperature in the estimation of proportion of bloodstream infections caused by *Acinetobacter baumannii* deserves special mention. Assessments of *A. baumannii* epidemiology have outlined that the pathogen has strong seasonal variability,[37] is found most in tropical and temperature climates, and that across geographies, the only climatic variable with a consistent association with the pathogen is temperature.[38] Of note is temperature's negative correlation with healthcare access and quality (in aggregate, warmer climates tend to be some of the most resource poor), one of the primary covariates we use to extrapolate our pathogen distributions across time. This negative correlation, in addition to the fact that the data we have for *A. baumannii* is more geographically rather than temporally disperse, determined a highly negative relationship between *A. baumannii* and healthcare access and quality in preliminary models that omitted temperature; in such models, the global death rate of BSI attributable to *A. baumannii* decreased by more than 50% between 1990 and 2021, far more than any other pathogen. After we included temperature as a covariate, the relationship between *A. baumannii* and healthcare access and quality stabilised, and trends in *A. baumannii* were much more consistent with similar Gram-negative pathogens. To mitigate the effect of temperature on other pathogens, we applied an exceptionally strong Gaussian prior as documented in table 6.3.2.1.

Section 6.3.3: Outliering

Pathogen distributions by age, syndrome, and year were reviewed by an expert committee of infectious disease specialists. Input data for pathogens found to make up an unreasonably low or high proportion of the syndrome distribution were reviewed using a varied set of visualizations and metrics (including in and out-of-sample residual estimates). Following this assessment, a select group of data (amounting to no more than 0.05% of the overall input data for any given syndrome) was omitted as outliers.

Section 6.3.4: Redistribution of "other pathogens"

Given computational and statistical limitations of assessing every possible pathogen in every syndrome model, our framework assessed the 25 most prevalent pathogens in the raw data (or all pathogens with over 300 records, if that number was smaller). The remaining pathogens were lumped into a residual "other pathogens" category. To better represent the pathogens encompassed in this "other" category in our results, we proportionally redistributed the "other pathogens" burden to the pathogens from our input data that weren't explicitly estimated (based on the number of cases observed in the data). For example, for bloodstream infections, our data included 12 pathogens in addition to the 25 we explicitly estimated: *Actinomyces* spp., *Aeromonas* spp., *Aspergillus* spp., *Cryptococcus* spp., Other *enterococci*, Other fungi, Other Gram-positives, *Histoplasma* spp., *Leptospira* spp., *Listeria* spp., non-polio enteroviruses, and Other *pseudomonas* species. Thus, the 3.1% of bloodstream infection cases attributed to "other pathogens" in neonates in Sub-Saharan Africa was redistributed to these 12 pathogens in accordance with their prevalence in the input data, with the proportion of burden attributed ranging from 1.2% for Other *enterococci* to 21.7% for Other fungi. The redistribution proportions varied by syndrome but were constant across age, location, and outcome (they were the same for both deaths and cases); future work will attempt to improve this estimation. The proportion of any given syndrome attributable to "other pathogens" was generally under 5%, with the leading non-estimated pathogen generally receiving around 20% of that burden—in other words, the amount of burden ascribed to any specific pathogen via this method was typically very small.

Section 6.4: Exceptions and special handling

There were some notable exceptions and special handling decisions made for individual pathogen distribution models. We hope to address many of these exceptions with more sustainable methods in our future work.

Section 6.4.1: Diarrhoea

In diarrhoea patients, cultures of specimens taken from the gastrointestinal tract, bowels, rectum, or stool are almost always affected by contaminants or pathogens that are not the cause of diarrhoea. For this reason, we believe that our input data and modelling framework are not able to accurately capture the aetiologies of diarrhoea. We chose to use GBD estimates of the aetiologies of diarrhoea in deaths instead of running our own model.[39] These estimates are based on the odds ratio of having diarrhoea given the detection of a pathogen, obtained from the Global Enteric Multicenter Study, therefore removing the influence of any pathogen that does not increase the risk of diarrhea.

A major limitation of using this study is that the GBD diarrhoea aetiology estimates are population attributable fractions (PAFs) for each pathogen. These PAFs may add to greater than 1 and the authors made no attempt to quantify the extent of co-occurrence of pathogens. This is inconsistent with the pathogen distribution estimation method used in our study, which estimates all pathogens as mono-infections. In order to avoid duplication of cases in our framework, we had to make some assumptions about the co-occurrence of pathogens in diarrhoea. We chose to normalise the PAFs to 1 for any demographic where the sum of GBD diarrhoea aetiology PAFs was greater than 1. This assumed that co-occurrence of pathogens was random and that the “other” pathogens category was negligible in these demographics. We made no adjustment to demographics where the PAFs added to less than 1. To convert the fatal PAFs to a distribution of aetiologies in incidence, we rescaled the distribution according to our estimates of the pathogen-specific case fatality ratios of diarrhea, calculated as described in section 5.

Section 6.4.2: Lower Respiratory Infections

GBD 2021 estimated the disease burden of the COVID-19 pandemic as an entity distinct from that of lower respiratory infection, and in the years 2020 and 2021, the envelope of non-COVID LRI deaths decreased substantially (from 2.55M deaths in 2019 to 2.28M in 2020 and 2.18M in 2021). For our analysis we did not assume that the reduction of death in non-COVID LRI differentially affected any particular pathogen; in other words, COVID-19 was not modelled as a shock in our pathogen distributions and the proportion of LRI attributable to each pathogen follows smooth trends from 2019-2021. Nonetheless, it is a possibility that the various interventions observed during the COVID-19 pandemic such as social distancing and mask use affected some pathogens more than others (such as pathogens that are similarly transmitted via droplets/aerosols). While our current methods do not account for this, we will seek to understand this pattern more clearly as better information about the state of non-COVID infectious disease during the pandemic is published and shared.

Section 6.4.3: Infectious syndromes not modelled

For three infectious syndromes that are caused by distinct pathogens whose individual burdens are already estimated in GBD as separate causes of death, we did not run a pathogen distribution model and instead simply used GBD estimates (table 6.4.3.1)

Table 6.4.3.1: Infectious syndromes for which we used GBD estimates to obtain the pathogen distribution

Infectious syndrome	Pathogens	GBD causes
Typhoid, paratyphoid, and invasive non-typhoidal Salmonella	Salmonella Typhi	Typhoid fever
	Salmonella Paratyphi	Paratyphoid fever
	Non-typhoidal Salmonella	Invasive non-typhoidal Salmonella
Tuberculosis	Mycobacterium tuberculosis	Tuberculosismongol
Gonorrhoea and chlamydia	Neisseria gonorrhoeae	Gonococcal infection
	Chlamydia trachomatis	Chlamydial infection

Additionally, we did not estimate pathogen distributions for the following 8 infectious syndromes that overwhelmingly involve viral and other non-bacterial pathogens: Carditis, myocarditis and pericarditis; Encephalitis, myelitis, meningoencephalitis, and other central nervous system infections; Eye infections; Genital infections; Oral infections; Other parasitic infections; Upper respiratory infections; and Unspecified site infections. As the focus of our current work was to estimate the burden of bacterial antimicrobial resistance, these syndromes had limited relevance to the current endeavor. Moreover, unbiased estimation of pathogen distributions for these syndromes was complicated by the fact that many represent mild disease (such as upper respiratory, oral, and eye infections) that is treated based on the presenting symptoms, and the causative pathogen is rarely identified. Correspondingly, those

records that do record the pathogen often represent a biased subsample of our data either involving more immunocompromised patients or more severe disease. Nevertheless, future work will seek to properly identify the pathogens responsible for these infections so that their burden can be appropriately quantified.

Section 6.5: Model validation

To assess model validity, we calculated the root mean square error (RMSE) and coefficient of determination (R^2) for each pathogen distribution model in proportion space for both in-sample and out-of-sample predictions (table 6.5.1). Proportions were predicted for each observation using the specific denominator observed from that study. For example, if a given study reported on only E. coli and S. pneumoniae, the predictions for model validation for this study were calculated as proportions of the total for E. coli and S. pneumoniae. In order to calculate out-of-sample fit, we perform non-exhaustive cross-validation, with each round of the validation holding out 1 country of data at a time. This leave-one-country-out approach simulates the prediction task of estimating the pathogen distribution of a country for which we have no data.

R^2 ranges from 0.743 to 0.927 in-sample and from 0.666 to 0.914 out of sample, indicating good model fit with only modest losses when data are moved out of sample. RMSE ranges from 0.078 to 0.148 in-sample and from 0.091 to 0.178 out of sample. Given that the data are expected to vary from the model predictions according to the observation-level variance, and the fact that the RMSEs are relatively consistent between in-sample and out-of-sample, these RMSEs are reasonable. Overall, these metrics show that these models have good fit and good out-of-sample predictive ability.

Table 6.5.1: In-sample and out-of-sample validation metrics for pathogen distribution models

Infectious syndrome	R^2		RMSE	
	In sample	Out of sample	In sample	Out of sample
Infections of the skin and subcutaneous systems	0.880	0.828	0.139	0.167
Bloodstream infections	0.743	0.723	0.115	0.119
Infections of bones, joints, and related organs	0.919	0.914	0.117	0.121
Endocarditis	0.927	0.870	0.078	0.104
Lower respiratory infections	0.754	0.666	0.130	0.152
Meningitis	0.815	0.733	0.148	0.178
Peritoneal and intra-abdominal infections	0.906	0.899	0.088	0.091
Urinary tract infections and pyelonephritis	0.800	0.794	0.115	0.117

Out of sample metrics calculated using leave-one-country-out cross validation

Section 7: Prevalence of resistance[1]

Section 7.1: Input data

We identified line level and aggregate data on the prevalence of resistance in bacterial pathogens, which were linked to the country and year in which the infection occurred, from datasets obtained from pharmaceutical companies, surveillance networks, academic institutions, and individual hospitals (see section 2). In total, we gathered over 210 million cases for the 84 pathogen–drug combinations we assessed. Table S11 provides a detailed breakdown of this total by pathogen–drug combination.

We supplemented microbiological data with systematic reviews following the Preferred Reporting Items for Systematic Reviews and Meta-Analyses (PRISMA) guidelines,[40] to collect resistance data published from countries and territories where surveillance systems do not routinely collect data to ensure extensive coverage of the pathogen–drug combinations thought to contribute the greatest burden of drug resistant infections, which we termed core pathogen–drug combinations (table 7.2.1). Data on the prevalence of AMR in these pathogen–drug

combinations were extracted from published literature and compiled into comprehensive datasets. The systematic reviews followed similar methodologies; a detailed description can be found either in published literature (*S.* Typhi and *S.* Paratyphi[41]) or in the corresponding PROSPERO records (*E. coli*, *K. pneumoniae*, *S. aureus* and *S. pneumoniae* PROSPERO registration CRD42019145148; *Shigella* species PROSPERO registration CRD42019127603; iNTS PROSPERO registration CRD42020189935; *N. gonorrhoeae* SPF unique identifier osf.io/4vy5n). The *S.* Typhi and *S.* Paratyphi A systematic review was expanded to include non-blood culture isolates for the current analysis. Literature reviews to supplement these data are described in section 2.5.

Forms were created, and screening and data extraction were completed using web-based systematic review software (DistillerSR, Evidence Partners, Ottawa, Canada) for all pathogens except *Salmonella*, for which a smaller number of manuscripts were identified.

To more comprehensively account for the burden of AMR in bacteria, we also estimated the prevalence of resistance for 8 supplementary pathogen–drug combinations for which we did not conduct a systematic literature review. Data for these supplementary combinations were extracted from the datasets obtained from pharmaceutical companies, academic institutes, and individual hospitals using the same processing procedure as was used for the core pathogen–drug combinations. The list of supplementary combinations is presented in table 7.2.2.

Section 7.1.1: Prevalence of Resistance in Mycobacterium tuberculosis

For the prevalence of drug resistance in *Mycobacterium tuberculosis* for multi-drug resistance (MDR, characterised by isoniazid and rifampicin co-resistance) excluding extensive drug resistance (XDR, characterised by resistance to isoniazid, rifampicin, and fluoroquinolone, as well as either aminoglycosides or capreomycin) and XDR, we used previously published GBD results.[2] Notably, GBD MDR excluding XDR TB estimates and the MDR/rifampin mono-resistant TB estimates from WHO differ, primarily because HIV/TB cases are included as part of WHO TB estimates. GBD adjusts the miscoding of deaths cause by HIV and TB in locations with high prevalence of both diseases, such as South Africa, assigning more deaths to HIV/TB (which are attributed to HIV), and these methodological differences lead to lower MDR TB mortality in the GBD burden estimates. An additional difference in estimates for MDR is that WHO includes rifampicin mono-resistance as part of their MDR TB figures.

Section 7.2: Data processing

The prevalence of resistance for each pathogen–drug combination was calculated for each data source, by country and year. All isolates determined to have intermediate resistance were classified as resistant. To determine the prevalence of resistance to a class of antibiotics (eg, fluoroquinolones), resistance, intermediate resistance or dose-dependent-susceptibility to any one of the antibiotics in the class was sufficient to classify an isolate as resistant for line level data (ie, susceptibility data for individual isolates). For aggregate data (ie, the proportion of isolates resistant to various antibiotics), the highest prevalence of resistance to any antibiotic in the class was selected. Multidrug resistance in *Salmonella* species was defined as concurrent resistance to ampicillin/amoxicillin, chloramphenicol, and trimethoprim-sulfamethoxazole; and fluoroquinolone resistance was defined as ciprofloxacin minimum inhibitory concentration of 0.125µg/ml or higher, or nalidixic acid resistance (CLSI breakpoint for *Salmonella* spp. were updated in 2012 to include 0.125 µg/ml as isolates with 'decreased ciprofloxacin susceptibility', and we have considered these as resistant). Nalidixic acid resistance was also used as a proxy for fluoroquinolone non-susceptibility for *Shigella* species.

Table 7.2.1: Core pathogen–drug combinations

Pathogen	Antimicrobial
Acinetobacter baumannii	Aminoglycosides, Anti-pseudomonal penicillin/Beta-lactamase inhibitors, Beta-lactam/Beta-lactamase inhibitors, Carbapenems, Third-generation cephalosporins, Fourth-generation cephalosporins, Fluoroquinolones
Citrobacter species	Aminoglycosides, Anti-pseudomonal penicillin/Beta-lactamase inhibitors, Carbapenems, Third-generation cephalosporins, Fourth-generation cephalosporins, Fluoroquinolones
Enterobacter species	Aminoglycosides, Anti-pseudomonal penicillin/Beta-lactamase inhibitors, Carbapenems, Fourth-generation cephalosporins, Fluoroquinolones, Trimethoprim-Sulfamethoxazole
Enterococcus faecalis	Fluoroquinolones, Vancomycin

Enterococcus faecium	Fluoroquinolones, Vancomycin
Escherichia coli	Aminoglycosides, Aminopenicillin, Beta-lactam/Beta-lactamase inhibitors, Carbapenems, Third-generation cephalosporins, Fluoroquinolones, Trimethoprim-Sulfamethoxazole
Klebsiella pneumoniae	Aminoglycosides, Beta-lactam/Beta-lactamase inhibitors, Carbapenems, Third-generation cephalosporins, Fluoroquinolones, Trimethoprim-Sulfamethoxazole
Morganella species	Third-generation cephalosporins, Fourth-generation cephalosporins, Fluoroquinolones
Proteus species	Aminoglycosides, Aminopenicillins, Third-generation cephalosporins, Fluoroquinolones, Trimethoprim-Sulfamethoxazole
Serratia species	Aminoglycosides, Anti-pseudomonal penicillin/Beta-lactamase inhibitors, Carbapenems, Third-generation cephalosporins, Fourth-generation cephalosporins, Fluoroquinolones
Pseudomonas aeruginosa	Aminoglycosides, Anti-pseudomonal penicillin/Beta-lactamase inhibitors, Carbapenems, Third-generation cephalosporins, Fourth-generation cephalosporins, Fluoroquinolones
Staphylococcus aureus	Fluoroquinolones, Macrolide, Methicillin, Trimethoprim-Sulfamethoxazole, Vancomycin
Streptococcus pneumoniae	Beta-lactam/Beta-lactamase inhibitors, Carbapenems, Third-generation cephalosporins, Fluoroquinolones, Macrolide, Penicillin, Trimethoprim-Sulfamethoxazole
Salmonella Typhi	Fluoroquinolones, Multidrug resistance
Salmonella Paratyphi A	Fluoroquinolones, Multidrug resistance
Invasive non-typhoidal *Salmonella*	Fluoroquinolones
Shigella species	Fluoroquinolones
Neisseria gonorrhoeae	Third-generation cephalosporins

Table 7.2.2: Supplementary pathogen–drug combinations

**Pathogen**	**Antimicrobial**
Group A *Streptococcus*	Macrolide
Group B *Streptococcus*	Fluoroquinolones, Macrolide, Penicillin
Haemophilus influenzae	Aminopenicillin, Third-generation cephalosporins
Neisseria gonorrhoeae	Fluoroquinolones

Group A *Streptococcus* = *Streptococcus pyogenes*. Group B *Streptococcus* = *Streptococcus agalactiae*.

Mycobacterium tuberculosis MDR and XDR follow previously published GBD results[2] instead of the processing steps considered in this section 7.2

Section 7.2.1: Resistance Breakpoint Processing

The prevalence of resistance for each pathogen–drug combination was calculated for each data source, by country and year. Whenever possible, we classified resistance using the most recent CLSI guidelines based on quantitative antimicrobial susceptibility testing (AST) data (MICs and disk zone diameters) provided in the data. When MICs were unavailable, we deferred to lab interpretation provided in the dataset to classify the isolates. All isolates determined to have intermediate or susceptible-dose-dependent resistance were classified as resistant. For the majority of data, only categorical AST results were available, and the guidelines used for the interpretation varied between organizations and years. AST interpretations from different standard organizations were harmonised and adjusted to levels corresponding to CLSI 2023. For this purpose, we calculated adjustment factors for years and guideline organizations (EUCAST and CLSI):

1. We used extracted AST values from all available datasets to calculate fraction of resistance for all the pathogens and antibiotic drug combinations, for each guideline, over multiple years, based on the appropriate breakpoints. For years before 2011 we used the 2011 breakpoints.
2. Using CLSI 2023 as our gold standard, we calculated an adjustment factor for each of the other guidelines based on the following equation:

$$adj = \frac{r_{clsi23}}{r_x}$$

where r = resistance fraction, and x = guideline being adjusted

To apply the adjustment factors, we multiplied the number of resistant cases for a specific source/year/location/pathogen/antibiotic combination with the appropriate adjustment factor. When data sources only reported laboratory interpretations, we assumed they used CLSI guidelines corresponding to the year of sample collection.

Breakpoints for both EUCAST and CLSI guidelines were based on guidelines manuals as recorded in the `AMR` R package by M. Berends.[42] We were unable to locate any breakpoints prior to the year 2011. Correspondingly, all data with laboratory interpretation from years prior to 2011 were assumed to use the breakpoints from 2011.

Section 7.2.2: Intrinsic Resistance Processing

Pathogen and antimicrobial drug combinations that are considered intrinsically resistant were also removed from the prevalence of resistance modelling. The list of the intrinsically resistant drug-bug combinations was compiled based on two sources: the CLSI guidelines[43] and the EUCAST[44] guidelines from 2020. A list of combinations excluded in this manner is provided in Table 7.2.2.1

Table 7.2.2.1 list of Intrinsic resistance combinations

**Pathogen**	**Antimicrobial**	**Antibiotic class**
Proteus spp.	Ampicillin	Aminopenicillin
Proteus spp.	Amoxicillin	Aminopenicillin
Pseudomonas aeruginosa	Ertapenem	Carbapenems
Pseudomonas aeruginosa	Ceftriaxone	Third-generation cephalosporins
Pseudomonas aeruginosa	Cefotaxime	Third-generation cephalosporins
Pseudomonas aeruginosa	Kanamycin	Aminoglycosides

Section 7.2.3: Tertiary Care Facility Adjustments

To account for the elevated levels of resistance found in tertiary care settings, we reviewed all input data used for the prevalence of resistance estimation and classified each data source as either tertiary, non-tertiary, or unknown/mixed designation, which was a commonly used classification for large resistance surveillance networks which don't report on the hospitals they collect data from. We located datasets that either provided facility information at the line-level or reported samples from exclusively tertiary or non-tertiary facilities. Where possible, we used tertiary/non-tertiary assignments from the data providers. When no assignments were available, we classified sites as tertiary, primary, and secondary by following the definitions provided by Jamison et al.[45] in table 7.2.3.1. We first considered hospital name when classifying. If the name did not include any of the terms listed in table 7.2.3.1, we searched the facility website for self-designations of tertiary/non-tertiary (most preferred), number of specialties, and bed-size. We classified facilities with vague names and with no websites or websites with insufficient information as "mixed/unknown"; data from these facilities could contain both tertiary and non-tertiary samples. Finally, we grouped primary and secondary facilities together in the non-tertiary category.

Table 7.2.3.1: Definitions and terms for different levels of hospital

Disease Control Priorities Project: terminology and definitions	Alternative terms commonly found in the literature
Primary-level hospital: few specialties—mainly internal medicine, obstetrics and gynecology, pediatrics, and general surgery, or just general practice; limited laboratory services available for general but not specialised pathological analysis	District hospital<br>Rural hospital<br>Community hospital<br>General hospital
Secondary-level hospital: highly differentiated by function with 5 to 10 clinical specialties; size ranges from 200 to 800 beds; often referred to as a provincial hospital	Regional hospital<br>Provincial hospital (or equivalent administrative area such as county)<br>General hospital
Tertiary-level hospital: highly specialised staff and technical equipment— for example, cardiology, intensive care unit, and specialised imaging units; clinical services highly differentiated by function; could have teaching activities; size ranges from 300 to 1,500 beds	National hospital<br>Central hospital<br>Academic or teaching or university hospital

For systematic review data collected from sub-Saharan Africa, we referred to Maina et al.[46] who identified and defined health facilities at each service delivery level (primary to tertiary) in sub-Saharan Africa using both information from health sector policies and strategic plans for each country in the region. They also undertook further comparative/validation analyses to cross reference the completeness/robustness of their classifications against the corresponding number of facilities reported at each level in the most current country-level health sector strategic plans and other health sector reports. Using this hierarchy by country (please see online table 2 in Maina et al.) we classified facilities in sub-Saharan Africa from the systematic review data as tertiary versus non-tertiary.

The proportion of data classified as originating from a tertiary facility differed substantially by super region, ranging from 0.003% of cases in the high-income super-region to 22.9% of cases in sub-Saharan Africa; this stark difference reaffirmed the importance of adjusting the data. To create robust inputs, data were aggregated by source, year, tertiary/non-tertiary status, and super-region. Because there was no reliable way to determine the mix of hospital types in mixed/unknown data, this data was grouped with non-tertiary. We chose to cluster this data with non-tertiary rather than omit it, as, for some super-regions, the proportion of definitively non-tertiary data was very low (eg, 2% for high-income). After aggregating the data in this way, we created a set of matched pairs, matching every tertiary data point to non-tertiary data for the same pathogen–drug combination from the same super-region collected within 5 years from one another.

Because the variation in resistance between tertiary and non-tertiary data could vary across different parts of the world, we ran a separate crosswalk for each super region and pathogen–drug *super group* combination. Crosswalks are a modeling method commonly used on the GBD to correct data with known biases (eg, alternative case definitions or measurement methods) using adjustments with correction factors estimated by network meta-regressions such as MR-BRT (meta-regression—Bayesian, regularised, and trimmed). MR-BRT allows for the implementation of a varied set of statistical models—linear and non-linear mixed effects models—and fitting procedures.[27] Certain bacteria and antimicrobials were clustered into super groups to provide the models with more robust input data, though, crucially, while a given model would contain several pathogen–drug combinations in its inputs, every matched pair was made comparing tertiary and non-tertiary values for the same combination. Bacteria were classified as follows:

Table 7.2.3.2: Pathogens in each pathogen super group

Pathogen super group	Incorporated pathogens
Gram-positives	*Enterococcus faecalis*, *Enterococcus faecium*, *Enterococcus* spp., Group A *Streptococcus*, Group B *Streptococcus*, *Staphylococcus aureus*, *Streptococcus pneumoniae*

Enteric bacterial pathogens	*Escherichia coli*, non-typhoidal *Salmonella*, *Salmonella* Paratyphi, *Salmonella* Typhi, and *Shigella* spp.
Other Enterobacterales and Pasteurellaceae	*Citrobacter* spp., *Enterobacter* spp., *Haemophilus influenzae*, *Klebsiella pneumoniae*, *Proteus* spp., *Serratia* spp.
Pseudomonadales	*Acinetobacter baumannii*, *Pseudomonas aeruginosa*

Notably, some pathogens were excluded from the tertiary crosswalk procedure because it was believed that infections with such pathogens would be robust to tertiary care bias. These pathogens were: *Mycobacterium tuberculosis* and *Neisseria gonorrhoeae*. Only one group of antimicrobials was clustered to create an antimicrobial super group, the β-lactam group, which was comprised of: aminopenicillin, anti-pseudomonal penicillin, β-lactamase inhibitors, carbapenems, third and fourth generation cephalosporins, methicillin, and penicillin. All other antibiotic classes (aminoglycosides, fluoroquinolones, macrolides, sulfonamides, and vancomycin) each individually comprised their own antimicrobial super group.

To allow us to implement linear models, resistance values were logit-transformed. We used the delta method to compute the standard error of the prevalence of resistance in logit space. To incorporate data with zero resistance, or with total resistance, we applied a 0.1% offset, such that the prevalence of resistance for data with zero resistance was represented as 0.1% and the prevalence of resistance for data with total resistance was represented as 99.9%. We then used the MR-BRT modelling framework to estimate the logit difference of tertiary and non-tertiary data for each super region-pathogen/antimicrobial 'super combination,' including a random effect for each pathogen–drug combination within the super combination and employing a positivity prior to enforce the constraint that the tertiary data exceed or be equal to the non-tertiary data. For the super region-pathogen/antimicrobial super combinations with sparse input data (fewer than 250 matched pairs) we instead used global estimated logit differences between tertiary and non-tertiary data for that pathogen/antimicrobial super combination, employing the same positivity prior.

After modelling the difference between tertiary and non-tertiary data, we implemented the models to adjust all the country-level tertiary input data that was indicated as biased. We then used the adjusted prevalence of resistance estimates from tertiary care facilities and unadjusted prevalence of resistance from non-tertiary/mixed care facilities as data inputs for the prevalence of resistance models. As was done before, resistance values were offset prior to logit-transformation to allow the use of linear models; data with zero resistance or complete resistance was offset by 2%. Exceptions to this offset were made for two combinations, *Staphylococcus aureus*/vancomycin and Group B *Streptococcus*/penicillin, which were anticipated to often have values beneath 2% resistance. For these combinations, we applied a 0.5% offset instead.

Section 7.3: Modelling framework

The prevalence of AMR in each pathogen–drug combination was modelled separately. We selected a range of spatially- and temporally-explicit health and socio-demographic-related covariates with biologically plausible associations to the prevalence of AMR in each pathogen from the Global Health Data Exchange (http://ghdx.healthdata.org/), and from published literature.[4] This list was narrowed down by fitting a lasso penalised regression model between the data and the covariates for each dataset (using the 'glmnet' package version 3.0.2 in R version 3.6.1) and selecting the most influential covariates in each of the pathogen–drug models to be taken forward. The list of covariates included for each pathogen–drug combination is given in table S4.

Due to the high heterogeneity of the input datasets, we outliered data points found to have the most extreme values for the prevalence of resistance. On a first stage, proportions above 99% and below 0.05% of resistant isolates were deemed implausible and excluded from the initial data across the following combinations: *Acinetobacter baumannii* resistant to carbapenems, anti-pseudomonal penicillin, fluoroquinolones; *Staphylococcus aureus* resistant to fluoroquinolones, methicillin, sulfa and vancomycin; *Citrobacter* spp. resistant aminoglycosides, third and fourth generation cephalosporins; *Streptococcus pneumoniae* resistant to beta-lactams, beta-lactams with beta-lactamase inhibitors, and carbapenems; *Escherichia coli* resistant to carbapenem and fluoroquinolones; *Klebsiella pneumoniae* resistant to fluoroquinolones; and *Pseudomonas aeruginosa* resistant to aminoglycosides and third generation cephalosporins. An initial generalised linear model (GLM) with country-fixed effects was fit to the data and covariates and input data points that lay outside of two times the median absolute deviation from the modelled estimate for each location were determined to be outliers and removed. The GLM was fit with nested random effects based on the GBD super-region, region, and country or territory to capture spatial effects, and was fit using the 'lme4' package version 1.1-21 in R version 3.6.1.

After the removal of extreme values, the datasets were used to fit spatiotemporal statistical models of the prevalence of AMR. Firstly, we used a stacked ensemble model to fit the associations between selected covariates and data. For each of the pathogen–drug combinations, we considered the following child models with country-fixed effects for inclusion: generalised additive models (GAM), penalised regression models (elastic-net, ridge, lasso), random forest, cubist, and neural-networks. Models were fit in R version 3.6.1, using the packages ‘CARET’ version 6.085, ‘mgcv’ version 1.8.31, and ‘glmnet’ version 3.0.2. We fit the child models using five-fold cross validation for each combination and selected the best performing, non-correlated child models based on the out-of-sample predictive performance. We then calculated the R^2-weighted mean of the estimates of the child models, constraining the coefficients to sum to one, and used these ensemble estimates to fit a spatiotemporal Gaussian process regression (ST-GPR) model for each pathogen–drug combination.

ST-GPR is described in detail elsewhere.[27] In brief, spatial and temporal weights were applied to the residuals of the stacked ensemble model; these were then added to the modelled estimates to smooth them in time and space. A Gaussian process regression (GPR) was then fit, and the mean prevalence of AMR was calculated from 100 draws of the GPR for each location and year. The 100 draws of the model were taken through to the next stage of calculations to propagate uncertainty throughout.

Section 7.4: Covariates

Supplementary table S4 (section 13) shows all of the covariates used to model the core pathogen–drug combinations and provides citations detailing the methods used to estimate these covariates. Covariates were available in GBD or adapted to be used in this estimation. Antibiotic consumption was considered as a covariate and took as basis estimates between 1990 and 2018. A multiple imputation method was employed (using the ‘mice’ package version 3.16.0 in R version 3.6.1) to project estimates between 2019 and 2021, with the initial set of covariates including the proportion of children with LRI using antibiotics model,[4] antenatal care coverage with at least 4 visits, hospital beds per 1000, latitude, mean temperature, outdoor air pollution, proportion with improved sanitation, total fertility rate, pharmacists per capita, and diabetes prevalence. For the multiple imputation between 2019 and 2021 we also considered the Worldwide Governance Indicators (www.govindicators.org) from The World Bank. The full list of covariates for each pathogen–drug combination was selected following a Least Absolute Shrinkage and Selection Operator penalised regression approach (using the ‘glmnet’ package version 3.0.2 in R version 3.6.1) as explained in section 7.3 above.

Section 7.5: Resistance profiles

To accurately assess the burden associated with resistance to each antibiotic, we needed to first understand the landscape of multidrug-resistant bacteria, for which the burden would be shared across several antibiotics. We therefore estimated, for each bacteria studied, a set of ‘resistance profiles’ characterised as the probabilities for each possible combination of resistance/susceptibility for all of the antibiotics analysed. For example, for a bacterium for which we assessed three antibiotics, we would estimate eight probabilities: SSS, SSR, SRS, RSS, SRR, RSR, RRS, and RRR (S – susceptible, R – resistant). These probabilities encompass the entire set of possibilities of resistance for the bacterium and sum to 1.

For a pathogen for which we assessed n antibiotics, resistance profiles were estimated by optimising over a $2^n - 1$-dimensional probability simplex with $\frac{n(n+1)}{2}$ linear constraints. Every such set of resistance profiles corresponds to a full specification of a multinomial distribution. The target set of constraints were as follows:

- The inferred marginal probability of resistance for each antibiotic (the prevalence of resistance to an antibiotic irrespective of all others analysed) exactly matches the estimates from our prevalence of resistance models. Since there are n antibiotics, this set comprises n constraints.
- The inferred pairwise likelihood of co-resistance for each pair of antibiotics exactly matches the likelihood inferred from the marginal probability of each antibiotic in the pair, and the Pearson correlation of resistance between the two antibiotics observed across all of the laboratory data we compiled. These represent $\frac{n^2-n}{2}$ additional constraints.

The input format for these constraints for an example case with n = 3 is shown in figure 7.5.1.

Figure 7.5.1: Example input matrix for calculating resistance profiles for a pathogen with 3 antibiotic classes (A,B,C)

Prev(A)	Prev(A&B)	Prev(A&C)
-	Prev(B)	Prev(B&C)
-	-	Prev(C)

Prev(X): prevalence of resistance of antibiotic X from ST-GPR model, by location and draw

Prev(X&Y): prevalence of resistance in both X and Y, back calculated from Prev(X), Prev(Y) and the Pearson correlation of X&Y in the lab data with multiple resistance screens.

In the $n = 2$ case, the number of constraints in our framework (3) is equal to the number of unknowns in the probability simplex ($2^n - 1 = 3$), and therefore at most one set of resistance profiles is possible. For all larger values of n, however, the number of unknowns exceeds the number of constraints, and there are infinite potential resistance profiles. Thus, our resistance profiles are generated by solving for a single sample from the probability simplex formed under the established constraints of marginal resistance and co-resistance.

There is no a priori guarantee that the observables generate a feasible solution. To prevent the constraints from delineating an infeasible probability simplex (for example, an input suggesting the individual resistances to antibiotics A and B are both above 90% but the probability of co-resistance to A and B is below 10%), we solved an optimisation problem that identified, for each input matrix, the closest feasible set of input constraints and a corresponding set of resistance profiles that fits these constraints. The 1-simplex in any dimension is specified by

$$\Delta \coloneqq \{p: \quad 0 \leq p_i \leq 1,\ \sum p_i = 1\} \qquad (7.5.1.1)$$

Each marginal observation and each pairwise co-resistance corresponds to a linear constraint, where a sum over a subset of the p in the simplex should be a given value v_i:

$$m_i^T p = v_i \qquad (7.5.1.2)$$

where m_i is a 'mask vector' of zeros and ones, used to pick out the appropriate summands. Overall, there are $\frac{n(n+1)}{2}$ such affine constraints. The optimisation problem we solve is to find the nearest feasible simplex given these constraints:

$$\min_{p \in \Delta} f(p) \coloneqq \sum_{i=1}^{n(n+1)/2} \frac{1}{\sigma_i^2} (m_i^T p - v_i)^2 \qquad (7.5.1.3)$$

Where $\frac{1}{\sigma_i^2}$ can be used to provide importance weights for the data. This is a least squares problem with linear equality and inequality constraints (corresponding to the simplex) and can be solved very efficiently even for relatively large n (such as 10 co-occurring antibiotic classes). The result is guaranteed to return the probability simplex closest to the specified constraint, even if the original set of constraints is infeasible, and corresponding set of resistance profiles that fits this nearest simplex.

To propagate uncertainty, we repeat this procedure for each of the 100 draws we estimate for prevalence of antibiotic resistance. To generate the i-th draw of our resistance profiles, we input the i-th draw of the marginal probability of resistance for each antibiotic analysed for a given pathogen into the probability simplex optimisation algorithm. Updating the marginal probabilities of resistance in turn influences the probabilities of co-resistance, and each element of the input we feed the algorithm is unique to the i-th draw. The optimisation is also initialised randomly for every draw. This process is implemented for each GBD country, resulting in 100 resistance profiles for each country for each pathogen in our analysis.

It is important to note that while we produce resistance profiles unique to each country, the Pearson correlations of co-resistance that we derive from the input data are assumed to be constant across location, year, sex, and infectious syndrome. Due to data sparsity, we cannot currently identify co-resistance patterns in several locations (particularly LMICs) with insufficient or non-existent line-level data; indeed, the data sources providing multiple resistance tests for a single isolate are among the most detailed of those we collected for this research and require exceptional data quality standards that are not easily achieved throughout the world. Identifying differences in patterns of co-resistance by location, year, or infectious syndrome is of considerable interest in the future.

Section 7.6: Model validation

Validation of prevalence of resistance modelling occurs in two instances. For the ensemble estimates, machine-learning candidate models are validated using five random holdout sets, and we select all models correlated below a Pearson correlation coefficient threshold of 0.8 and weight the ensemble based on the R^2 predictive validity for the out-of-sample predictions. These intermediary results are not reported in this paper because they do not pertain to the final prevalence of resistance estimate.

We then validate the entire ensemble ST-GPR process by calculating in-sample and out-of-sample accuracy metrics. Accuracy is measured as the proportion of correctly classified resistant/susceptible isolates based on the modelled estimate and the raw data's prevalence of resistance. As a worked example, if there were 10 isolates with 50% resistance in the raw data and the model predicted 60% resistance for that location, we would have 5 correctly classified resistant samples (true positives), 1 incorrectly classified resistant sample (false positive), and 4 correctly classified susceptible samples (true negatives), for 90% accuracy. For out-of-sample cross-validation, we withheld, at the outset of the ensemble modelling process, a set of 20% of countries with data as a holdout group at each iteration, for 5 total holdout sets. Table 7.6.1 reports the accuracy metric for each pathogen–drug combination. Our in-sample accuracy values range from 87% to 99.5%, while our out-of-sample accuracy values range from 82.7% to 97.9%.

Table 7.6.1: In-sample and out-of-sample accuracy estimates for prevalence of resistance models

Pathogen	Antibiotic class	In-sample accuracy	Out-of-sample accuracy
Acinetobacter baumannii	3GC	0.940	0.889
Acinetobacter baumannii	4GC	0.929	0.922
Acinetobacter baumannii	AG	0.937	0.886
Acinetobacter baumannii	Anti-pseudomonal	0.938	0.932
Acinetobacter baumannii	BL-BLI	0.915	0.890
Acinetobacter baumannii	CP	0.913	0.888
Acinetobacter baumannii	FQ	0.919	0.885
Citrobacter spp.	3GC	0.981	0.966
Citrobacter spp.	4GC	0.991	0.988
Citrobacter spp.	AG	0.981	0.974
Citrobacter spp.	Anti-pseudomonal	0.978	0.915
Citrobacter spp.	CP	0.984	0.947
Citrobacter spp.	FQ	0.984	0.959
Escherichia coli	3GC	0.970	0.964
Escherichia coli	AG	0.974	0.956
Escherichia coli	Aminopenicillin	0.974	0.917
Escherichia coli	BL-BLI	0.921	0.906
Escherichia coli	CP	0.985	0.981
Escherichia coli	FQ	0.952	0.912
Escherichia coli	TMP-SMX	0.950	0.955
Enterococcus faecalis	FQ	0.989	0.956
Enterococcus faecalis	Vancomycin	0.992	0.986
Enterococcus faecium	FQ	0.987	0.980

Enterococcus faecium	Vancomycin	0.975	0.966
Enterobacter spp.	4GC	0.970	0.925
Enterobacter spp.	AG	0.973	0.975
Enterobacter spp.	Anti-pseudomonal	0.976	0.970
Enterobacter spp.	CP	0.961	0.975
Enterobacter spp.	FQ	0.968	0.912
Enterobacter spp.	TMP-SMX	0.971	0.974
Group A *Streptococcus*	Macrolide	0.958	0.963
Group B *Streptococcus*	FQ	0.965	0.950
Group B *Streptococcus*	Macrolide	0.934	0.915
Group B *Streptococcus*	Penicillin	0.986	0.961
Haemophilus influenzae	3GC	0.995	0.961
Haemophilus influenzae	Aminopenicillin	0.987	0.982
Klebsiella pneumoniae	3GC	0.966	0.957
Klebsiella pneumoniae	AG	0.965	0.949
Klebsiella pneumoniae	BL-BLI	0.950	0.935
Klebsiella pneumoniae	CP	0.976	0.977
Klebsiella pneumoniae	FQ	0.931	0.927
Klebsiella pneumoniae	TMP-SMX	0.947	0.945
Morganella spp.	3GC	0.954	0.930
Morganella spp.	4GC	0.990	0.988
Morganella spp.	FQ	0.960	0.967
Neisseria gonorrhoeae	3GC	0.960	0.968
Neisseria gonorrhoeae	FQ	0.957	0.947
non-typhoidal *Salmonella*	FQ	0.961	0.951
Pseudomonas aeruginosa	3GC	0.978	0.976
Pseudomonas aeruginosa	4GC	0.977	0.974
Pseudomonas aeruginosa	AG	0.982	0.952
Pseudomonas aeruginosa	Anti-pseudomonal	0.967	0.928
Pseudomonas aeruginosa	CP	0.955	0.956
Pseudomonas aeruginosa	FQ	0.967	0.924
Proteus spp.	3GC	0.966	0.955
Proteus spp.	AG	0.983	0.937
Proteus spp.	Aminopenicillin	0.993	0.976
Proteus spp.	FQ	0.976	0.891
Proteus spp.	TMP-SMX	0.992	0.911

Staphylococcus aureus	FQ	0.927	0.951
Staphylococcus aureus	Macrolide	0.945	0.940
Staphylococcus aureus	Methicillin	0.949	0.906
Staphylococcus aureus	TMP-SMX	0.989	0.985
Staphylococcus aureus	Vancomycin	0.986	0.982
Salmonella Paratyphi	FQ	0.901	0.891
Salmonella Paratyphi	MDR	0.933	0.824
Salmonella Typhi	FQ	0.913	0.945
Salmonella Typhi	MDR	0.870	0.827
Streptococcus pneumoniae	3GC	0.987	0.983
Streptococcus pneumoniae	BL-BLI	0.974	0.972
Streptococcus pneumoniae	CP	0.978	0.967
Streptococcus pneumoniae	FQ	0.982	0.986
Streptococcus pneumoniae	Macrolide	0.963	0.938
Streptococcus pneumoniae	Penicillin	0.937	0.932
Streptococcus pneumoniae	TMP-SMX	0.962	0.931
Serratia spp.	3GC	0.954	0.883
Serratia spp.	4GC	0.985	0.957
Serratia spp.	AG	0.966	0.979
Serratia spp.	Anti-pseudomonal	0.976	0.885
Serratia spp.	CP	0.976	0.944
Serratia spp.	FQ	0.961	0.903
Shigella spp.	FQ	0.914	0.896

3GC = Third-generation cephalosporins. 4GC = Fourth-generation cephalosporins. AG = Aminoglycosides. Anti-pseudomonal = Anti-pseudomonal penicillin/Beta-Lactamase inhibitors. BL-BLI = Beta Lactam/Beta-lactamase inhibitors. CP = Carbapenems. FQ = Fluoroquinolones. MDR in S. Typhi and Paratyphi = Multi-drug resistance in *Salmonella* Typhi and Paratyphi. TMP-SMX = Trimethoprim-Sulfamethoxazole.

Section 8: Relative risk[1]

Section 8.1: Input data

The input data for the relative risk estimation step included literature data that provided relative risk of death for resistant and susceptible organisms and hospital-based microbiology surveillance data linked to outcomes, as well as other clinical parameters (eg, demographics, diagnoses). Published studies were identified from a recent meta-analysis performed by Cassini and colleagues.[50]

The data inputs for the excess duration estimates were literature data that reported on length of stay for resistant and susceptible organisms and hospital-based microbiology surveillance data that were linked to outcomes as well as various other clinical parameters (eg, demographics, diagnoses). The number of days between a positive specimen date and discharge date was used to obtain the mean duration of infection. We considered days elapsed between admission and discharge as mean duration of stay if this was the only piece of information provided in the study. We also considered median duration of infection or median duration of stay if the study only provided this piece of information.

Section 8.2: Data processing

There were 355 million samples derived from 694 000 deaths, 23 million cases, and 260 million recorded hospital days with infection across 73 countries to inform our relative risk of death estimates. Of the 355 million potential samples, duplicates were dropped as part of the modelling process (modelling details in 7.3). A detailed breakdown of the cases, deaths, and hospital days with infection by pathogen–drug is in table S5 (section 13). Relative risk estimates were extracted from primary literature as were study characteristics that described the adjustments made by the study. For sources with line-level data, we calculated crude relative risks. For literature sources that reported summary metrics such as the mean, median, and interquartile range of length of stay, we used the values as reported. Outliers were assessed via visual inspection of the results and expert opinion, and generally included values with unbelievably low (<0.7) or unbelievably high (>2) relative risks that we attribute to various bias covariates we were unable to control for due to data sparsity.

Section 8.3: Modelling overview

We estimated both fatal and nonfatal relative risks. The purpose of these models was to measure the excess risk of death or increased length of stay in a hospital from an infection with an antibiotic-resistant pathogen compared to a susceptible pathogen. In updating our modelling framework, the greatest challenge was data sparsity. Relative risk is the most data sparse estimation step because of its extensive data inclusion requirements. Input data must have patient outcomes linked to microbiological tests indicating the pathogen(s) isolated from their infection(s) and the results of antibiotic resistance testing. Furthermore, for each pathogen–drug combination, the data must include both cases and/or deaths for susceptible and resistant isolates. Correspondingly, accounting for all the various characteristics that modify relative risk of death and length of stay was out of scope due to the lack of available information across all sources. For example, not all sources reported patient underlying causes of death and/or days spent in the hospital prior to infection. We instead used a two-stage process to generate a sound baseline estimate for relative risk related to a given antibiotic class prior to modeling specific bug-drug combinations in a second series of models.

Section 8.3.1: Fatal modelling

In this two-stage modelling process, we first generated a baseline estimate of relative risk by antibiotic class that accounted for variation by underlying source. Then, in the second stage, we generated estimates of relative risk by unique pathogen–drug–infectious syndrome combinations. Similar to Cassini and colleagues,[49] we assumed that the relative risk of death was non-varying with respect to age, sex, and location.

The stage one models were a series of 13 mixed effect binomial logistic regressions—one for each antibiotic class—with a fixed effect on resistance (susceptible or resistant) and a random effect on source (equation 8.3.1.1). The inclusion of the random effect on source was motivated by substantial heterogeneity between sources. The input data to this stage was crude relative risks by antibiotic class and source calculated as the ratio of the case fatality ratios (CFRs) of resistant infections over susceptible infections. From the stage one models we calculated odds ratios which we converted into relative risks through Zhang and Yu's[51] conversion method. In this way we estimated a baseline relative risk of death by antibiotic class which we then used as the prior on the intercept of the stage two model.

$$logit(death_d) = \beta_0 + \beta_1 \cdot x + u_{j,d} \tag{8.3.1.1}$$

Where x is a binary covariate on the presence of resistance and u is the random effect on source j for antibiotic class d.

The second stage models were a series of MR-BRT meta regressions for each antibiotic class with a fixed effect on infectious syndrome and a random effect on pathogen (Equation 8.3.1.2). The data was too sparse to run a unique model by each pathogen–drug combination of interest and so each model included all data related to a given antibiotic class based on the assumption that pathogens with resistance to a given antibiotic class would have a similar baseline increased risk of death (or intercept in terms of the model). The input data to this stage was crude relative risks by antibiotic class, pathogen, and infectious syndrome, calculated the same as for the stage one models. Assignment of infectious syndrome was categorised into one of four categories: bloodstream infections, lower respiratory infections, urinary tract infections, and all other syndromes. Data with an unspecified infectious syndrome informed the stage one model, which was unique by antibiotic class, but not the stage two model. Additionally, we imposed Gaussian priors with mean 0 and non-zero variance on the coefficient of the infectious syndrome covariate, to bias the model away from spurious effects driven by data sparsity. The Gaussian priors were based on expert opinion and can be improved with further empirical validation and sensitivity analyses in the future.

Furthermore, due to the heterogeneity of the input data, which was impacted by small numbers yielding a wide range of relative risks that could indicate that resistance was highly protective (>30% reduction in risk) or associated with a high increase in risk (>100% increase in risk), we employed four types of stage two models: (i) aggregated across infectious syndrome with weak priors, (ii) aggregated across infectious syndrome with strong priors, (iii) stratified by infectious syndrome with weak priors, and (iv) stratified by infectious syndrome with strong priors. We prioritised models with weak priors (standard deviations > 0.01) and only when the estimate of risk was protective did we use estimates from models with strong priors (standard deviations <= 0.00001). Additionally, we used models with infectious syndrome detail in all cases except for select pathogen–drug combinations identified through expert opinion as having too much variability in the final estimates. For these select pathogen–drug combinations, the MR-BRT model was specified the same as in Equation 8.3.1.2 but without the fixed effect on infectious syndrome.

$$Relative\ Risk_{pathogen_n drug_d} = \beta_0 + \beta_1 \cdot x_1 + \cdots + \beta_n \cdot x_n + u_{pathogen_n} + \epsilon \qquad (8.3.1.2)$$

Where x is a categorical fixed effect on infectious syndrome, u is a random effect on pathogen n within an antibiotic class, ϵ is the measurement error, and d is antibiotic class. From this stage two model, we produced a point estimate and 100 draws to incorporate uncertainty from this estimation step into the final result. We calculated the uncertainty interval as 1.96 standard deviations above and below the mean.

Section 8.3.2: Nonfatal Modelling

For non-fatal estimation, we estimated the excess duration attributable to resistance—comparing the length of hospital stay for an infection with a pathogen resistant to the antibiotic of interest to an infection of the same site with the same organism that was susceptible. For community-acquired infections the entire duration of length of stay was attributed to the infection, for hospital-acquired infections we used the time from first positive culture to time of discharge to estimate length of stay. It is important to note that the afforementioned challenges posed by data sparsity increased significantly in this step as availability of culture dates and discharge dates was limited within our data. To estimate the relative risk of increased length of stay, we used a modelling framework similar to that from the fatal estimation but slightly modified.

Because days of hospital stay is greater than the number of cases, we could not calculate a crude relative risk of length of stay manually. To generate initial estimates of relative risk of increased length of stay after the onset of infection, we used a Poisson regression with a fixed effect on the type of infection (resistant or susceptible) (equation 8.3.2.1).

$$log(\lambda_i) = \beta_0 + \beta_1 \cdot x_i \qquad (8.3.2.1)$$

Where λ_i is the expected count of deaths of infection for observation i and x is the binary coefficient associated with presence of resistance. The coefficient β_1 was extracted as the initial estimate of relative risk of increased length of stay.

Similar to the fatal estimation, we used a two-stage process wherein the stage one models produced a stable baseline of relative risk of increased length of stay by antibiotic class. A binomial logistic regression was not appropriate in this case because the ratio of hospital length of stay and cases is not bounded by 0 and 1. Therefore, we used a MR-BRT regression for the antibiotic class-specific stage one models (equation 8.3.2.2). As before, the results from the stage one models were used as priors on the intercepts of the stage two models.

$$Relative\ Risk_d = \beta_0 + u_i + \epsilon \qquad (8.3.2.2)$$

Where d is the antibiotic class, $u_i$as a random effect on data source, and ϵ is the measurement error.

The stage two models were the same as the stage two models in the fatal modelling framework but without any inclusion of the effect of infectious syndrome due to data sparsity.

The analysis of relative risk followed the definitions of the prevalence of resistance step (section 7) as closely as possible. Both analyses identified resistance to a given antibiotics class if the isolate had an intermediate or resistant interpretation to any one of the antibiotics in that given class. But the analysis of relative risk diverged from the analysis of prevalence of resistance in the following circumstances. First, the relative risk step included molecular resistance testing if this was the only data provided by a study, eg, β-lactamase or mecA positive pathogens; this could potentially misclassify some resistant organisms as sensitive (or vice versa) if they had an alternate mechanism for resistance, such as a porin alteration leading to carbapenem resistance or a non-expressed resistance

mechanism. Second, the relative risk estimate produced was for sterile sites of infection, as there was limited data from non-sterile sites. Third, it was not possible to assess relative risk of multidrug-resistant pathogens because of limited data availability and because it did not fit in the modelling strategy at the antibiotic class level. Instead, the relative risk of each of the components of multidrug-resistant pathogens was calculated and the antibiotic class with the highest relative risk was used; for *Salmonella* Typhi this was relative risk to fluoroquinolones. Fourth, we had limited availability of data on fatalities attributable to *Salmonella* Paratyphi and invasive non-Typhoidal *Salmonella* species; as a result, we used fatal relative risk estimates from *Salmonella* Typhi as a proxy. Fifth, there were limited data on fatalities attributable to resistant *N. gonorrhoeae*, so we excluded the fatal estimate for this pathogen. Finally, the relative risk of *Mycobacterium tuberculosis* was assessed for multidrug and extensively drug-resistant infections as reported at the global level in GBD. We took the ratios of the GBD mortality and incidence ratio of drug-susceptible TB and drug-resistant TB (separately for both MDR and XDR) as a proxy for fatal relative risk. Nonfatal relative risk estimates were taken by year whereas fatal estimates were taken as a single all year aggregate from 1990 to 2021.

Section 8.4: Model validation

We report three summary metrics to evaluate the relative risk of death models: the root-mean squared error (RMSE), the Mean Average Error (MAE) and the percent coverage of observed data within the full variance of the model. These three metrics were calculated using the real relative risk ratio in the whole sample of data and also by holding out 25% of the sample within antibiotic class in 4 iterations. Table 8.4.1 provides details for each of the antibiotic class models evaluated. Large MAE and RMSE values indicate that observed data deviates from the mean model estimate. We also see a large proportion of the data (76% and more) falls within the total variance of each model estimate. This indicates that large deviations from the mean estimate coincide with large variances of the data observed.

Table 8.4.1: In-sample and out-of-sample performance metrics for relative risk of death models

**Antibiotic class**	**Model type**	**Pathogens**	**In Sample**			**Out of Sample**		
			**RMSE**	**MAE**	**coverage**	**RMSE**	**MAE**	**coverage**
Aminoglycosides	no syndrome detail	*Acinetobacter baumannii*, *Citrobacter* spp., *Enterobacter* spp., *Escherichia coli*, *Klebsiella pneumoniae*, *Proteus* spp., *Pseudomonas aeruginosa*, *Serratia* spp.	0.55	0.4	82%	1.13	0.4	82%
Aminopenicillin	with syndrome detail	*Escherichia coli*, *Haemophilus influenzae*, *Proteus* spp.	0.86	0.56	88%	1.48	0.57	90%
Anti-pseudomonal penicillin/Beta-Lactamase inhibitors	no syndrome detail	*Acinetobacter baumannii*, *Citrobacter* spp., *Enterobacter* spp., *Pseudomonas aeruginosa*, *Serratia* spp.	0.97	0.61	94%	1.63	0.61	94%
Beta Lactam/Beta-lactamase inhibitors	no syndrome detail	*Acinetobacter baumannii*, *Enterobacter* spp., *Escherichia coli*, *Klebsiella pneumoniae*, *Serratia* spp., *Streptococcus pneumoniae*	1.02	0.49	97%	1.59	0.49	97%
Carbapenems	no syndrome detail	*Acinetobacter baumannii*, *Citrobacter* spp., *Enterobacter* spp., *Escherichia coli*, *Klebsiella pneumoniae*, *Pseudomonas aeruginosa*,	0.99	0.62	80%	1.66	0.62	80%

		Serratia spp., *Streptococcus pneumoniae*						
Fluoroquinolones	no syndrome detail	*Acinetobacter baumannii*, *Citrobacter* spp., *Enterobacter* spp., *Enterococcus faecalis*, *Enterococcus faecium*, *Escherichia coli*, Group B *Streptococcus*, *Klebsiella pneumoniae*, *Morganella* spp., *Non-typhoidal Salmonella*, *Proteus* spp., *Pseudomonas aeruginosa*, *Salmonella Paratyphi*, *Salmonella Typhi*, *Serratia* spp., *Shigella* spp., *Staphylococcus aureus*, *Streptococcus pneumoniae*	5.99	1.18	80%	6.2	1.18	80%
Fourth-generation cephalosporins	no syndrome detail	*Acinetobacter baumannii*, *Citrobacter* spp., *Enterobacter* spp., *Morganella* spp., *Pseudomonas aeruginosa*, *Serratia* spp.	0.45	0.36	92%	1.06	0.36	92%
Penicillin	no syndrome detail	Group B *Streptococcus*, *Streptococcus pneumoniae*	0.54	0.45	100%	1.28	0.45	100%
Trimethoprim-Sulfamethoxazole	no syndrome detail	*Enterobacter* spp., *Escherichia coli*, *Klebsiella pneumoniae*, *Proteus* spp., *Staphylococcus aureus*, *Streptococcus pneumoniae*	0.74	0.41	83%	1.31	0.41	83%
Third-generation cephalosporins	no syndrome detail	*Acinetobacter baumannii*, *Citrobacter* spp., *Escherichia coli*, *Haemophilus influenzae*, *Klebsiella pneumoniae*, *Morganella* spp., *Proteus* spp., *Pseudomonas aeruginosa*, *Serratia* spp., *Streptococcus pneumoniae*	1.72	0.73	82%	2.22	0.73	82%
Macrolide	no syndrome detail	Group A *Streptococcus*, Group B *Streptococcus*, *Staphylococcus aureus*, *Streptococcus pneumoniae*	0.78	0.48	86%	1.35	0.48	86%
Methicillin	no syndrome detail	*Staphylococcus aureus*	0.78	0.5	76%	1.44	0.5	76%
Vancomycin	no syndrome detail	*Enterococcus faecalis*, *Enterococcus faecium*, *Staphylococcus aureus*	2	0.81	86%	2.5	0.81	86%

This approach for relative risk estimation has a number of limitations, many of which are attributable to data sparsity. First, it is likely that the impact of resistance on mortality is different across locations and ages. In locations where overall health-care access and quality are very poor, the impact of resistance may be smaller because the

management of susceptible infections is sub-optimal. On the other hand, in locations where broad, second- and third-line antimicrobials are not available, one would expect the impact of resistance to be greater. Second, it is possible that the relative risk of death attributable to resistance is different across anatomical sites of infection because of variable penetrance of antibiotics to different anatomical locations. With regard to age, those that are older may have more comorbidities that affect their immune systems and increase the risk of adverse complications from resistant infections. As we continue efforts to expand data collection and reporting, we hope to be able to address these limitations in future iterations.

Section 9: Counterfactuals and AMR estimation[1]

Section 9.1: Estimating associated AMR burden with counterfactual of no infection

We computed two counterfactuals to estimate the drug-resistant burden. First, we estimated the burden of AMR using the counterfactual of no infection. We estimated the fatal burden of individual pathogen–drug combinations by taking the product of the deaths for each underlying cause, fraction of deaths related to infection, infectious syndrome fraction, fatal pathogen fraction, and fatal prevalence of resistance and then summed across all infectious syndromes and underlying causes:

$$Deaths\ with\ Resistance_{Kd} = \sum_{J}\sum_{L} D_J \times S_J \times M_{LJ} \times P_{LK} \times R_{Kd} \quad (9.1.1)$$

where D = deaths, S = fraction related to infection, M = infectious syndrome fraction, P = fatal pathogen fraction, R = fatal prevalence of resistance, J = cause, L = syndrome, K = pathogen, d = drug. To produce an estimate of deaths with resistance to any antibiotic estimated, we employed the same formula but used the fatal prevalence of resistance to any antibiotic using the resistance profiles, described previously in Section 7.5. We calculated the fatal prevalence of resistance R for a given drug d based on the non-fatal prevalence of resistance R' and relative risk of death RR for this drug:

$$R_{Kd} = \frac{R'_{Kd} RR_{Kd}}{(1 - R'_{kd}) + R'_{Kd} RR_{Kd}} \quad (9.1.2)$$

We calculated the fatal prevalence of resistance to any antibiotic estimated based on the non-fatal prevalences of each resistance profile, incorporating all resistance profiles δ that are resistant to at least 1 drug with corresponding relative risks RR_{Kd^*}, determined by the method described below (section 9.2):

$$R_{K,all\ drugs} = \frac{\sum_{\delta} R'_{K\delta} RR_{Kd^*}}{\left(1 - \sum_{\delta} R'_{K\delta}\right) + \sum_{\delta} R'_{K\delta} RR_{Kd^*}} \quad (9.1.3)$$

We then estimated YLLs using standard GBD methods to convert age-sex specific deaths into YLLs.[2]

For the non-fatal estimate, we first estimated the incidence of each infectious syndrome in each underlying cause. For these select infectious syndromes, we simply used the corresponding proxy cause incidence estimated in GBD (table 9.1.1).

Table 9.1.1: Infectious syndromes where we used GBD proxy causes for MI ratio

Infectious syndrome	Proxy cause
Diarrhoea	Diarrhoea
Endocarditis	Endocarditis
Carditis, myocarditis, and pericarditis	Myocarditis
Upper respiratory infections	Upper respiratory infections
Tuberculosis	Tuberculosis
Sexually transmitted infections	Sexually transmitted infections excluding HIV<br>Chlamydia<br>Gonococcal infection<br>Syphilis

Typhoid, paratyphoid, and invasive non-typhoidal Salmonella	Typhoid fever<br>Paratyphoid fever<br>Invasive non-typhoidal Salmonella
Other parasitic infections	Other neglected tropical diseases
Hepatitis	Acute hepatitis<br>Acute hepatitis A<br>Acute hepatitis B<br>Acute hepatitis C<br>Acute hepatitis E
Other unspecified site infections	Other unspecified infectious diseases

For infectious syndromes that do not utilize GBD incidence (table 9.1.2), we divided the infectious syndrome deaths ($D_j \times S_J \times M_{LJ}$) by the syndrome-and pathogen-specific CFRs calculated in section 5 adjusted with a syndrome-specific adjustment factor to account for the bias of our CFR data to reflect inpatient disease. We then aggregated across pathogen using the nonfatal pathogen distribution P' calculated in Section 6. The adjustment factor CR_L, is based on the Poland Healthcare claims database, where we converted claims records into inpatient and outpatient infectious events according to the ICD-10 code, facility designation, and a cause duration as assigned by area experts. We linked all records from 2015 to 2021 by patient to process all infectious events for individuals in the records. Events consisted of unique patients and either one or multiple claims records dependent on the duration period of a given cause. For example, in a chronic cause any patient could only have one event for that given cause in their lifetime. After establishing these events, two ratios were generated. The first ratio, referred to as the "inpatient ratio" was inclusive of all inpatient events (ie those that included at least one inpatient record) and the total number of events, taking the form

$$inpatient\ CR_L = \frac{\sum_J Any\ event\ with\ inpatient\ record_J}{\sum_J All\ Events_J}$$

The second ratio, referred to as the "outpatient to inpatient" ratio, consisted of events that had an outpatient to inpatient transition, and the total number of outpatient events with form

$$outpatient\ to\ inpatient\ CR_L = \frac{\sum_J Any\ event\ with\ outpatient\ to\ inpatient\ instance_J}{\sum_J Any\ event\ with\ an\ outpatient\ record_J}$$

The "inpatient ratio" was used to adjust our CFR models for community acquired infections for lower respiratory and urinary tract infections. Blood stream infections, meningitis, peritoneal and intra-abdominal infections and the hospital-acquired infection models for lower respiratory and urinary tract infections did not have a ratio and utilized unadjusted CFRs. The "outpatient to inpatient ratio" was used in all other syndromes. All adjusted or unadjusted CFRs were vetted for compatibility with published GBD results for consistency.

$$Incidence_{JL} = \frac{D_J S_J M_{LJ}}{\sum_K CFR_{LK} CR_L P'_{LK}} \quad (9.1.4)$$

Table 9.1.2: Incidence estimates as derived from CFRs and healthcare claims adjustments

**Infectious syndrome**	**Adjustment factor used on CFRs**
Meningitis and other bacterial central nervous system infections	None
Lower respiratory infections and other related infections in the thorax – community acquired	Inpatient ratio
Lower respiratory infections and other related infections in the thorax – hospital acquired	None
Sexually transmitted infections	Outpatient to inpatient ratio

Urinary tract infections and pyelonephritis – community acquired	Inpatient Ratio
Urinary tract infections and pyelonephritis – hospital acquired	None
Bloodstream infections	None
Infections of the skin and subcutaneous systems	Outpatient to inpatient ratio
Oral infections	Outpatient to inpatient ratio
Eye infections	Outpatient to inpatient ratio
Infections of bones, joints, and related organs	Outpatient to inpatient ratio
Peritoneal and intra-abdominal infections	None

We then took the product of the infectious syndrome incidence, the non-fatal pathogen fraction, and the non-fatal prevalence of resistance and summed across all infectious syndromes and underlying causes to get incidence with resistance for every pathogen and drug. As with the fatal estimate, to produce an estimate of incident infections with resistance to any antibiotic, we used the same formula and used the non-fatal prevalence of resistance to any antibiotic estimated from the resistance profiles.

We then calculated YLDs for each pathogen. For some GBD causes, we simply used the GBD YLD estimates and multiplied them by the corresponding nonfatal pathogen distribution (table 9.1.3). For all other causes, we multiplied together the infectious syndrome incidence, the non-fatal pathogen fraction, and a syndrome-specific YLDs per incident case rate, calculated using a proxy cause from GBD.[27] To estimate the YLDs per incident case rate, we extracted GBD incidence and YLD estimates for the proxy causes and divided the YLDs by the incidence for each age, sex, and location. Three infectious syndromes are not estimated in the GBD, and therefore have no standard sequelae or disability weights: bloodstream infections, intra-abdominal infections, and bone and joint infections. For the proxy causes for these three syndromes, we used the closest approximate disease as determined by a group of experts in infectious diseases and epidemiology (table 9.1.3). This approach is a significant limitation of the study and should be improved in future work.

Table 9.1.3: Proxy causes used to calculate YLDs per incidence case rate for each infectious syndrome

**Infectious syndrome**	**Proxy cause**
Meningitis and other bacterial central nervous system infections	Meningitis
Lower respiratory infections and other related infections in the thorax	Lower respiratory infections
Urinary tract infections and pyelonephritis	Urinary tract infections and interstitial nephritis
Bloodstream infections	Maternal sepsis and other maternal infections – (*Extrapolated to Males)*<br>Neonatal sepsis and other neonatal infections
Infections of the skin and subcutaneous systems	Bacterial skin diseases<br>Cellulitis<br>Pyoderma<br>Decubitus ulcer
Eye infections	Otitis media
Oral infections	Otitis media
Infections of bones, joints, and related organs	Bacterial skin diseases<br>Musculoskeletal disorders

Peritoneal and intra-abdominal infections	Paralytic ileus and intestinal obstruction
Diarrhoea	Diarrhoea
Endocarditis	Endocarditis
Carditis, myocarditis, and pericarditis	Myocarditis
Upper respiratory infections	Upper respiratory infections
Tuberculosis	Tuberculosis
Genital Infections	Urinary tract infections and interstitial nephritis
Sexually transmitted infections	Sexually transmitted infections excluding HIV<br>Chlamydia<br>Gonococcal infection<br>Syphilis
Typhoid, paratyphoid, and invasive non-typhoidal Salmonella	Typhoid fever<br>Paratyphoid fever<br>Invasive non-typhoidal Salmonella
Other parasitic infections	Other neglected tropical diseases
Hepatitis	Acute hepatitis<br>Acute hepatitis A<br>Acute hepatitis B<br>Acute hepatitis C<br>Acute hepatitis E
Other unspecified site infections	Other unspecified infectious diseases

To get the YLDs associated with resistance for each pathogen, we used the non-fatal prevalences of resistance for each drug and resistance profile and relative length of stay (LOS) for each pathogen–drug combination to calculate the fraction of YLDs associated with resistance for each pathogen, using equations analogous to equations 9.1.2 and 9.1.3. We multiplied this fraction by the YLDs for each pathogen to get YLDs associated with resistance to each pathogen–drug combination and YLDs associated with resistance any antibiotics estimated. We then added YLLs and YLDs to produce the DALY estimate for burden associated with resistance.

Section 9.2: Estimating attributable AMR burden with counterfactual of infection with susceptible organism

For the second counterfactual—comparing resistant to susceptible infections—we calculated mutually exclusive pathogen–drug estimates. To do this, we first estimated the population attributable fraction of deaths ($Mortality\ PAF$) for each resistance profile with resistance to at least 1 drug, δ. The inputs for the PAF were the non-fatal prevalence of the given resistance profile, $R'_{K\delta}$, and the relative risk of death for resistant infection compared to susceptible infection for each drug, RR_{kd}. Because of data sparsity, we were unable to calculate the relative risk for every possible resistance profile, and so instead used the highest relative risk of all of the drugs in the resistance profile. For example, for a resistance profile characterized by resistance to penicillin and fluoroquinolones where the relative risk was 1.1 for penicillin and 1.4 for fluoroquinolones, we would use a relative risk of 1.4 for this profile. The mortality PAF is calculated as a multi-category exposure:

$$Mortality\ PAF_{K\delta} = \frac{R'_{K\delta}(RR_{Kd^*} - 1)}{1 + \sum_{\delta} R'_{K\delta}(RR_{Kd^*} - 1)} \quad (9.2.1)$$

where d^* is the drug in the resistance profile δ with the highest relative risk.

We then took the product of the deaths for each underlying cause, fraction of deaths related to infection, infectious syndrome fraction, fatal pathogen fraction, and the mortality PAF for each resistance profile to get the deaths attributable to resistance for every resistance profile:

$$Deaths\ due\ to\ Resistance_{K\delta} = \sum_{J}\sum_{L} D_J \times S_J \times M_{LJ} \times P_{LK} \times Mortality\ PAF_{K\delta} \qquad (9.2.2)$$

When the resistance profile described resistance to more than one antibiotic, the deaths were then distributed to the component pathogen–drug combinations based on the excess risk of the pathogen–drug combination divided by the sum of the excess risk of all pathogen–drug combinations in the resistance profile. For a resistance profile δ with resistance to drugs $i = 1, \dots, n$:

$$Redistribution\ Weight_{Kd_i} = \frac{RR_{Kd_i} - 1}{\sum_{i}(RR_{Kd_i} - 1)} \qquad (9.2.3)$$

For co-resistance amongst beta-lactam antibiotics (i.e. carbapenems, 4GC, 3GC, antipseudomonal, BL/BLI, aminopenicillins, and penicillin), we used a different approach to redistributing burden. Similar to Cassini et al., we applied a hierarchy such that the burden was categorically attributed to the broadest beta-lactam antibiotic, rather than split the burden between multiple beta-lactam antibiotics.[50] We used the hierarchy in table 9.2.1 to assign burden in the presence of co-occurring beta-lactam resistance. When a pathogen was resistant to multiple beta-lactams and a non-beta-lactam antibiotic, we first applied the hierarchy to determine the ‘highest’ beta-lactam resistance and then generated redistribution weights using only the ‘highest’ beta-lactam and the non-beta-lactams. We then used these attributable death estimates to estimate YLLs using standard GBD methods to convert age-sex specific deaths to YLLs.

A similar approach was taken to estimate non-fatal burden for the counterfactual of antibiotic-susceptible infection. We first assumed that antibiotic resistance has no effect on the attack rate of pathogens; therefore, there are 0 incident cases attributable to resistance and all non-fatal burden comes from increased length of illness. To quantify the extent of this increased length of illness, we first produced a length of stay (LOS) PAF for each resistance profile using the non-fatal prevalence of resistance and relative LOS for resistant infections as compared to susceptible infections in a method analogous to equation 9.2.1. Because of data sparsity, we were unable to calculate the relative LOS for every resistance profile, and so instead used the relative LOS for the drug with the highest relative LOS in the profile. We then took the product of the YLDs for each infectious syndrome, the non-fatal pathogen distribution, and the LOS PAF to produce attributable YLD estimates. This assumes that the attributable LOS PAF is equally applicable to all sequelae, which is an assumption made because of a lack of data on the impact of resistance on the likelihood of different sequelae and the duration of specific sequelae. We then added YLLs and YLDs to produce an estimate of DALYs attributable to resistance.

Table 9.2.1: Beta-lactam hierarchy

Rank	Antibiotic class
1	Carbapenem
2	Antipseudomonal Penicillin/Beta-lactamase Inhibitor
3	Fourth Generation Cephalosporin
4	Third Generation Cephalosporin
5	Beta-lactam/Beta-lactamase Inhibitor
6	Aminopenicillin
7	Penicillin

Because of the optimisation approach used to derive each resistance profile, the prevalence of resistance for a given pathogen–drug as modelled using ensemble ST-GPR (section 7.3), R'_{Kd}, will not necessarily be exactly equal to the sum of all resistance profiles $R'_{K\delta}$ that include resistance to drug d. Due to this inconsistency, in extremely rare cases, an estimate of AMR burden in the susceptible counterfactual may slightly exceed the corresponding estimate of AMR burden in the no infection counterfactual for a specific pathogen–drug. We consider the ensemble ST-GPR estimate to be more accurate than the resistance profiles, since the latter are based on Pearson correlations of multidrug resistance that are calculated from limited microdata and generalised to all locations. For this reason, we cap all individual pathogen–drug estimates of burden for the susceptible counterfactual, which are based on the

resistance profiles, to the burden for the no infection counterfactual, which are based on the ensemble ST-GPR estimates.

Section 9.3: Excluded combinations

Although our approach attempted to be exhaustive and include all clinically-relevant pathogen–drug combinations, there are several combinations we do not produce estimates for. Examples of combinations that have been previously emphasised in the discourse but are not studied here include clarithromycin resistance in Helicobacter pylori and fluoroquinolone resistance in Campylobacter species. These were excluded due to limited data availability as highlighted by a recent study in the European Union that found that, as of 2019, no member countries had implemented publicly accessible, mandatory reporting surveillance programmes for these two pathogen–drug combinations.[52] H. pylori and Campylobacter spp are commonly diagnosed without culture so resistance profiles are uncommon in passive surveillance systems. The burden of H. pylori is not currently estimated in GBD, though some of the consequent diseases are, like peptic ulcer disease and gastric cancer. Producing a burden estimate of H. pylori was outside the scope of this work, and without a pathogen burden estimate, we could not produce an estimate of the burden attributable to clarithromycin-resistant H. pylori. In contrast, GBD does produce an estimate on the burden of Campylobacter spp. There were, however, too few data to produce an estimate on the excess risk of death or duration associated with fluoroquinolone resistance and limited data to inform a global prevalence of resistance estimate. Given these limitations, we did not produce burden estimates for clarithromycin-resistant H. pylori or fluoroquinolone-resistant Campylobacter spp.

Because of the lack of data on risk of death associated with drug-resistant Neisseria gonorrhoeae, we were unable to produce an estimate of the fatal burden of resistance so produce only a non-fatal estimate. Many potential pathogen–drug combinations were excluded due to the spectrum of antimicrobial activity (ie, Vancomycin and *E. coli*), intrinsic resistance (eg, BL/BLI resistance in *Pseudomonas aeruginosa*) or resistance that is exceedingly common (eg, penicillin resistance in *S. aureus*); these combinations were decided by a group of experts in infectious diseases, microbiology, epidemiology, and population health. There were insufficient data to produce a global estimate for many pathogen–drug combinations of interest, such as aminopenicillin resistance in *Enterococcus* spp., fluoroquinolone resistance in *Acinetobacter baumannii*, or colistin resistance in any pathogen estimated. This is largely due to either a lack of regional data to inform the prevalence of resistance component or a lack of microbial data linked to outcomes to inform the measure of excess risk component. A final constraint was the computational burden of estimating more than seven antibiotic classes for a single pathogen. Because of the approach to co-resistance described in section 7.4, each antibiotic class added led to an exponential increase in the computation needs and anything above seven antibiotic classes was not tenable. As additional data are made available, we plan to add clinically relevant combinations and iterate on the computational approach so that we can describe the burden of bacterial AMR more comprehensively.

Lastly, in this iteration of our study, we did not quantify *M. tuberculosis* with mono-resistance to either rifampicin or isoniazid. These combinations were included in previous iterations of our research, however the estimation of a time-series for each of these two combinations proved challenging. Rifampicin and isoniazid mono-resistant TB are the only pathogen–drug combinations that consider the landscape of resistance to other drugs—"mono-resistance" necessitates that the pathogen is resistant to no other antibiotics—and correspondingly we could not include several data sources which did not provide the complete antibiogram (like single drug resistance profiles or literature data). Given the scarcity of data, we were unable to estimate a time series for these combinations at this time. We include resistance burden estimates for multidrug resistant tuberculosis and extensively drug resistant tuberculosis, both of which are characterized by co-occurring isoniazid and rifampicin resistance, for consistency and comparability with the previously published GBD estimates.

Section 9.4: Decomposition of factors contributing to change in AMR associated deaths

To better understand the relative contributions of factors driving the change in AMR associated deaths, we prepared a six-factor decomposition using methods developed by Das Gupta and described in prior studies.[24,53–55] The aim of this decomposition was to isolate the effect of each factor on the change in the number of AMR associated deaths between 1990 and 2019 (the latter year chosen because it excludes the shock effects of the COVID-19 pandemic). The results of the decomposition show the contribution that factor would have made to the change in the number of deaths had all other factors been held constant between two time points. These factors' effects are represented as a positive or negative contribution in death counts. The sum of each factor's effects is equal to the net change between 1990 and 2019.

Section 9.4.1: Decomposition Methods Overview

We prepared a decomposition comparing the change in AMR associated deaths between 1990 and 2019, both globally (Main Text table 4) and by GBD super-region (table 9.4.1). The decomposition was first prepared by all sex, detailed age groups for each factor, which was then summed into an all-age aggregate. For the purposes of this analysis, we identified six factors for the decomposition: (1) population growth, (2) the population age structure, (3) the sepsis mortality rate, (4) the proportion of sepsis deaths associated with the 11 AMR syndromes (table 4.4.1.1), (5) the proportion of AMR syndrome deaths associated with AMR bacteria (table 7.2.1 and 7.2.2), and (6) the proportion of AMR bacteria deaths associated with resistance.

To calculate the effects of population growth and population age structure, we first prepared a five-factor decomposition[24] using the change in age-specific population as one of five factors (along with the four previously stated factors). The change due to the age-specific population was then further decomposed into i) the change due to population growth, which was estimated assuming equal percent population change in all age groups between 1990 to 2019 and ii) change due to population age structure, which was set equal to the residual between the population growth estimate from i) and the estimate of change due to age-specific population from the five-factor analysis.

The other factors represent proportions produced by the component models that contribute to the AMR associated burden estimate (see Methods). The sepsis mortality rate is counts of deaths associated with sepsis divided by population. The proportion of sepsis deaths associated with AMR syndromes is the count of sepsis deaths associated with infectious syndromes contributing to AMR (see table 4.4.1.1), divided by the number of deaths associated with sepsis. The proportion of AMR syndrome deaths associated with AMR bacteria was calculated by dividing bacterial deaths related to the 21 pathogens we estimate AMR burden for by sepsis deaths associated with the 11 aforementioned infectious syndromes. The proportion of AMR bacteria deaths associated with resistance is estimated using deaths where the infection was caused by an AMR bacteria that had resistance divided by deaths associated with the 11 infectious syndromes and 21 bacteria. Because relative risk remains static over time, we did not include this as a factor in our decomposition.

AMR associated deaths can therefore be determined as a product of six factors and decomposed using Das Gupta's decomposition methods:[55]

$$AMR\ associated\ deaths = \alpha * \beta * \gamma * \delta * \varepsilon * \zeta$$

where α = Population growth, β = Population age structure, γ = Sepsis mortality rate, δ = Proportion of sepsis deaths associated with AMR syndromes, ε = Proportion of AMR syndrome deaths associated with AMR bacteria, and ζ = Proportion of AMR bacteria deaths associated with resistance

Section 9.4.2: Decomposition results

Table 9.4.1 presents the results of the decomposition analysis that quantifies the impact of different aggregate factors on the number of AMR-associated deaths between 1990 and 2019, presented at an aggregated all age level, for both sexes, by GBD super-regions. Each row represents a distinct factor, showing how changes in that factor alone would have influenced the AMR-associated deaths, assuming all the other factors remained constant. As an example, in the super region, "Southeast Asia, East Asia, and Oceania", row one shows that if AMR-associated deaths were impacted solely by population growth, with no changes to age structure of the population, the proportion of sepsis deaths associated with AMR, or any other measured factors, AMR-associated deaths would increase by 231,000 deaths—from 1,110,000 in 2019 to 1,340,000 in 2021. All of the factors simultaneously influenced AMR-associated deaths over time, suggesting that while globally population growth, changes in age structure, and an increased proportion of AMR syndrome deaths due to AMR bacteria and resistance contributed to a rise in deaths, this was counterbalanced by a reduction in deaths from decreasing sepsis death rates (Main text table 4).

Table 9.4.1: Decomposition of factors driving change in AMR associated deaths from 1990 to 2019, all ages, all sexes, by GBD super-region

	Southeast Asia, East Asia, and Oceania	Central Europe, Eastern Europe, and Central Asia	High-income	Latin America and Caribbean	North Africa and Middle East	South Asia	Sub-Saharan Africa
**AMR-associated deaths, 1990**	1,110,000	285,000	477,000	247,000	264,000	1,400,000	990,000
Between 1990 and 2019, observed the following changes in associated deaths due to changes in…							
**Population growth**	+231,000	-1,370	+74,500	+97,900	+152,000	+741,000	+909,000
**Age structure**	+607,000	+79,600	+289,000	+124,000	+6,130	-15,000	-213,000
**Sepsis death rate**	-1,050,000	-68,000	-202,030	-150,000	-208,000	-1,400,000	-768,000
**Proportion of sepsis deaths associated with AMR syndromes**	-25,200	-8,210	+6,310	-301	+6,390	+35,500	-50,900
**Proportion of AMR syndrome deaths associated with AMR bacteria**	+59,500	-1,920	-33,800	+4,080	-552	+220,000	+27,300
**Proportion of AMR bacteria deaths associated with resistance**	+209,000	-4,530	-32,500	+15,700	+21,600	+372,000	+111,000
**Net change**	**+28,900**	**-4,460**	**+102,000**	**+91,900**	**-21,900**	**-49,500**	**+16,000**
**AMR-associated deaths, 2019**	1,140,000	281,000	579,000	339,000	264,000	1,350,000	1,010,000

Section 10: Forecasting AMR

The Institute for Health Metrics and Evaluation (IHME) future health scenarios framework uses estimates of disease burden, drivers of disease burden (such as risk exposure), and demographic indicators from the GBD.

Section 10.1: Future health scenarios platform overview

IHME's Future Health Scenarios Team produces forecasted estimates based on past GBD data for 359 causes across 21 regions, seven super-regions, and at the global level, for five-year age groups and sex.[56–58] A detailed description of the GBD framework and retrospective AMR estimates is available in the main text of this paper, the GBD 2021 Lancet series, and multiple AMR-specific publications.[1,2,13,24,27,59–61] Figure S3 provides an overview of the multi-staged forecasting modelling process. Our previous publications on forecasting thoroughly explain the methods used to forecast the independent drivers, risk factors, mortality, demography indicators, and DALYs.[56–58,62] We use the reference forecasts, which is a probabilistic forecast of the most likely future of cause-specific disease burden to estimate both deaths and DALYs associated with AMR (number of deaths and DALYs among people who have a resistant infection) and mortality and DALYs attributable to AMR (number of deaths and DALYs due to AMR). Additionally, we developed two policy-based scenarios of averted mortality burden (number of deaths and DALYs) that can be potentially avoided if a particular policy is implemented.

Section 10.2: Forecasting AMR population attributable fractions

To integrate AMR into the forecasting framework, we utilised historical estimates of deaths due to AMR (attributable deaths) by GBD cause and calculated 19 population attributable fractions (PAFs) for GBD Level 2 causes with AMR attributable death counts.

We forecasted the fraction of cause-specific deaths due to AMR using a Generalized Ensemble Model (GenEM). This model employed 12 different sub-models (or child models), utilizing two main modeling approaches: the weighted annualized rate of change (ARC) and a two-stage spline model based on the meta-regression—Bayesian, regularized, trimmed tool (MR-BRT).[12] Each model had six different recency-weighting parameters ranging from 0 to 2.5.

For the ARC child models, we calculated the age-standardized, sex-specific, and location-specific annual change of the logit-transformed AMR PAF values. To account for the effect of noisy data, we replaced annual changes outside the 2.5th and 97.5th percentiles with those corresponding percentile values. The two-stage MR-BRT child models employed the first stage to fit age-standardized, sex-specific logit of the AMR PAF on SDI:

$$logit\left(AMR\ PAF_{c,s,t}\right) = \beta_0 + \beta_1 spline\left(SDI_{c,t}\right) + \varepsilon_{c,s,t}\ , \qquad (1)$$

where $logit(AMR\ PAF_{c,s,t})$ is the logit of the age-standardized AMR PAF in country c, sex s, and year t, β_0 is an intercept, β_1 is a coefficient matrix, spline is the spline with five knots placed evenly across the distribution of SDI data and assumes both right and left linear tails, and $\varepsilon_{c,s,t}$ is the residual. This was then followed by the second stage, where the logit of the residuals from the first stage was linearly modeled on time (year):

$$logit\left(\varepsilon_{c,s,t}\right) = year_t + \lambda + \Psi_{c,s,t}\ , \qquad (2)$$

where λ is a fixed intercept value and $\psi_{c,s,t}$ is an error term.

The weight of each sub-model was determined by out-of-sample predictive validity experiments. We trained each sub-model using data from 1990–2011 and validated them based on data from 2012–2021. The performance of each child model was measured using root mean square error (RMSE), which was then used to assign sampling weights to each child model.

We generated sub-model forecasts using the 1990–2021 training dataset. For each ARC child model, we used the calculated annual change with the corresponding recency-weighting parameter to produce 2022–2050 AMR PAF forecasts. For the MR-BRT child models, we used forecast SDI values in addition to the recency weights to obtain forecasting values of AMR PAFs based on the model fit.

We then obtained the final AMR PAFs ensemble forecasts by averaging the predictions of the child models using the sampling weights obtained from the out-of-sample experiments.

Section 10.3: Computing future attributable and associated AMR burden

We calculated the following three scenarios of AMR burden shown in table 11.3.1.

To compute the attributable AMR burden, we began by multiplying our reference mortality and YLL forecasts for 19 cause groups at the age-sex-location level by the forecasted AMR PAFs (described in the above section). Next, we applied a scalar to the attributable YLLs using the global ratio of YLL:YLD AMR deaths in 2021 to calculate AMR-attributable YLDs. Finally, we summed AMR-attributable YLL and YLD results to determine AMR-attributable DALYs.

To compute associated AMR burden, we first calculated the ratio of AMR-associated deaths to AMR-attributable deaths for 19 cause groups by age-sex-location in the year 2021. We then used this ratio to multiply our AMR PAFs and calculate associated burden forecasts for each measure in the same manner as we computed attributable burden.

Section 10.4: Developing AMR alternative scenarios

Besides generating a reference forecast, our framework allows us to produce alternative scenarios of disease burden by associating assumed changes in PAFs and CFRs with different scenarios. These scenarios were applied to all locations. If the reference forecast for a particular location was more optimistic than the defined alternative scenario, the reference forecast was used.

Section 10.4.1: Gram-Negative Drug scenario

Gram-Negative Drug scenario is defined as a regular release of new drugs targeting Gram-negative bacteria. For this scenario, we first calculated the fraction of AMR-attributable deaths due to Gram-negative infections in the year 2021 ($fraction_{AMR\_deaths}$). We then multiplied the future reference scenario AMR PAFs by $fraction_{AMR\_deaths}$ and $(1 - fraction_{AMR\_deaths})$ to obtain future Gram-negative PAFs and future non Gram-negative PAFs, respectively.

Afterwards, for the future Gram-negative PAFs, we linearly decreased the gram-negative PAFs using 2021 as a starting point until PAFs value in 2036 is 50% of the PAFs value in 2021, and then hold them constant from 2037 to 2050. Then, we added the non-gram-negative PAFs to scenario gram-negative PAFs to determine the total PAFs based on the Gram-Negative Drug scenario. Finally, we calculated the fraction of this resulted total PAFs ($fraction_{AMR\_PAFs}$) and then multiplied this fraction by the mortality to obtain the number of deaths attributable to AMR for the Gram-Negative Drug scenario.

Section 10.4.2: Better Care scenario

The Better Care scenario is associated with improved case fatality ratios, leveraging retrospective estimates reflective of varying health system strength. To calculate the death rates for this scenario ($m_{c\_scenario,t}$) for a cause c at time t, we used case fatality ratios (CFRs) that varied by age, location, and infectious syndrome, fraction of cause due to infectious syndrome:

$$\mathrm{m_{c\_scenario,t}} = \mathrm{m_{c,t}} \left(1 - \sum_{i=1}^{n_s} \mathrm{F_{cs}}(1 - \mathrm{CFR\_ratio_s})\right)_t$$

where $m_{c,t}$ is the total death rate for a cause c at time t, n_s is the number of infectious syndromes, and CFR_ratio_s is a required relative reduction in CFR for an infectious syndrome, F_{cs} is the fraction of a cause due to an infectious syndrome. The fraction of a cause due to sepsis is accounted for in F_{cs}.

CFR_ratio_s for infectious syndrome was calculated as $CFR_{s,HAQ=84.16}/CFR_{ls,2021}$ where $CFR_{s,HAQ=84.16}$ is CFR value for an infectious syndrome that corresponds to the 85th percentile of Healthcare Access and Quality (HAQ) Index[15] in 2021 (HAQ Index = 84.16) by location and age group; $CFR_{ls,2021}$is the CFR value in 2021 by location and age group. We chose the 85th percentile of HAQ Index to capture the progress required by 2030 in a country where access to and quality of health care needs improvement (HAQ Index in the majority of high-income countries is already above 84.16 in 2021). For obtaining the value of $CFR_{s,HAQ=84.16}$, we used the age-specific relationship of HAQ Index and CFR across 204 countries for 11 major infectious syndromes.

Section 11: GATHER compliance

This study complies with GATHER recommendations.[64] We have documented the steps in our analytical procedures and detailed the data sources used. See section 13 table S6 for the GATHER checklist. The GATHER recommendations can be found on the GATHER website.

Section 12: References

1 Antimicrobial Resistance Collaborators. Global burden of bacterial antimicrobial resistance in 2019: a systematic analysis. *Lancet* 2022; **399**: 629–55.

2 GBD 2021 Causes of Death Collaborators. Global burden of 288 causes of death and life expectancy decomposition in 204 countries and territories and 811 subnational locations, 1990-2021: a systematic analysis for the Global Burden of Disease Study 2021. *Lancet* 2024; **403**: 2100–32.

3 CDC. National Healthcare Safety Network (NHSN) patient safety component manual. CDC, 2021 https://www.cdc.gov/nhsn/pdfs/validation/2021/pcsmanual_2021-508.pdf (accessed Aug 16, 2024).

4 Browne A, Chipeta M, Haines-Woodhouse G, et al. Global antibiotic consumption in humans, 2000 to 2018: a spatial modelling study. *Lancet Planet Health* 2021.

5 Lozano R, Lopez AD, Atkinson C, Naghavi M, Flaxman AD, Murray CJ. Performance of physician-certified verbal autopsies: multisite validation study using clinical diagnostic gold standards. *Popul Health Metr* 2011; **9**: 32.

6 Naghavi M, Makela S, Foreman K, O'Brien J, Pourmalek F, Lozano R. Algorithms for enhancing public health utility of national causes-of-death data. *Popul Health Metr* 2010; **8**: 9.

7 Johnson SC, Cunningham M, Dippenaar IN, et al. Public health utility of cause of death data: applying empirical algorithms to improve data quality. *BMC Med Inf Decis Mak* 2021; **21**: 175.

8 Iburg KM, Mikkelsen L, Adair T, Lopez AD. Are cause of death data fit for purpose? Evidence from 20 countries at different levels of socio-economic development. *PLOS ONE* 2020; **15**: e0237539.

9 Birnbaum JK, Murray CJL, Lozano R. Exposing misclassified HIV/AIDS deaths in South Africa. *Bull World Health Organ* 2011; **89**: 278–85.

10 Murray CJL, Rajaratnam JL, Marcus J, Laakso T, Lopez AD. What can we conclude from death registration? Improved methods for evaluating completeness. *PLOS Med* 2010; **7**: e1000262.

11 Foreman KJ, Lozano R, Lopez AD, Murray CJL. Modeling causes of death: an integrated approach using CODEm. *Popul Health Metr* 2012; **10**: 1.

12 Zheng P, Barber R, Sorensen RJD, Murray CJL, Aravkin AY. Trimmed constrained mixed effects models: formulations and algorithms. *J Comput Graph Stat* 2021; **30**: 544–56.

13 GBD 2021 Demographics Collaborators. Global age-sex-specific mortality, life expectancy, and population estimates in 204 countries and territories and 811 subnational locations, 1950-2021, and the impact of the COVID-19 pandemic: a comprehensive demographic analysis for the Global Burden of Disease Study 2021. *Lancet* 2024; **403**: 1989–2056.

14 Rudd KE, Johnson SC, Agesa KM, *et al.* Global, regional, and national sepsis incidence and mortality, 1990–2017: analysis for the Global Burden of Disease Study. *Lancet* 2020; **395**: 200–11.

15 GBD 2019 Healthcare Access and Quality Collaborators. Assessing performance of the Healthcare Access and Quality Index, overall and by select age groups, for 204 countries and territories, 1990-2019: a systematic analysis from the Global Burden of Disease Study 2019. *Lancet Glob Health* 2022; **10**: e1715–43.

16 Weiner LM, Webb AK, Limbago B, *et al.* Antimicrobial-resistant pathogens associated with healthcare-associated infections: summary of data reported to the National Healthcare Safety Network at the Centers for Disease Control and Prevention, 2011-2014. *Infect Control Hosp Epidemiol* 2016; **37**: 1288–301.

17 Jones RN. Microbial etiologies of hospital-acquired bacterial pneumonia and ventilator-associated bacterial pneumonia. *Clin Infect Dis Off Publ Infect Dis Soc Am* 2010; **51 Suppl 1**: S81-87.

18 Johansson N, Kalin M, Tiveljung-Lindell A, Giske CG, Hedlund J. Etiology of community-acquired pneumonia: increased microbiological yield with new diagnostic methods. *Clin Infect Dis Off Publ Infect Dis Soc Am* 2010; **50**: 202–9.

19 Czaja CA, Scholes D, Hooton TM, Stamm WE. Population-based epidemiologic analysis of acute pyelonephritis. *Clin Infect Dis Off Publ Infect Dis Soc Am* 2007; **45**: 273–80.

20 Nicolle LE. A practical guide to antimicrobial management of complicated urinary tract infection. *Drugs Aging* 2001; **18**: 243–54.

21 Goto M, Ohl ME, Schweizer ML, Perencevich EN. Accuracy of administrative code data for the surveillance of healthcare-associated infections: a systematic review and meta-analysis. *Clin Infect Dis Off Publ Infect Dis Soc Am* 2014; **58**: 688–96.

22 Wright SB, Huskins WC, Dokholyan RS, Goldmann DA, Platt R. Administrative databases provide inaccurate data for surveillance of long-term central venous catheter-associated infections. *Infect Control Hosp Epidemiol* 2003; **24**: 946–9.

23 GBD 2020, Release 1, Vaccine Coverage Collaborators. Measuring routine childhood vaccination coverage in 204 countries and territories, 1980-2019: a systematic analysis for the Global Burden of Disease Study 2020, Release 1. *Lancet* 2021; **398**: 503–21.

24 GBD 2021 Risk Factors Collaborators. Global burden and strength of evidence for 88 risk factors in 204 countries and 811 subnational locations, 1990-2021: a systematic analysis for the Global Burden of Disease Study 2021. *Lancet* 2024; **403**: 2162–203.

25 GBD 2019 Human Resources for Health Collaborators. Measuring the availability of human resources for health and its relationship to universal health coverage: estimates for 204 countries and territories from 1990 to 2019. *Lancet* 2022; **399**: 2129–54.

26 Galles NC, Liu PY, Updike RL, *et al.* Measuring routine childhood vaccination coverage in 204 countries and territories, 1980–2019: a systematic analysis for the Global Burden of Disease Study 2020, Release 1. *Lancet* 2021; **398**: 503–21.

27 GBD 2021 Diseases and Injuries Collaborators. Global incidence, prevalence, years lived with disability (YLDs), disability-adjusted life-years (DALYs), and healthy life expectancy (HALE) for 371 diseases and injuries in 204 countries and territories and 811 subnational locations, 1990-2021: a systematic analysis for the Global Burden of Disease Study 2021. *Lancet* 2024; **403**: 2133–61.

28 IHME Math Sciences. RegMod v0.2.0. 2023 https://github.com/ihmeuw-msca/regmod/tree/v0.2.0. (accessed June 7, 2024).

29 Ewig S, Schlochtermeier M, Göke N, Niederman MS. Applying sputum as a diagnostic tool in pneumonia: limited yield, minimal impact on treatment decisions. *Chest* 2002; **121**: 1486–92.

30 Ogawa H, Kitsios GD, Iwata M, Terasawa T. Sputum gram stain for bacterial pathogen diagnosis in community-acquired pneumonia: a systematic review and Bayesian meta-analysis of diagnostic accuracy and yield. *Clin Infect Dis* 2020; **71**: 499–513.

31 Feikin DR, Scott JAG, Gessner BD. Use of vaccines as probes to define disease burden. *Lancet* 2014; **383**: 1762–70.

32 Troeger C, Blacker B, Khalil IA, *et al.* Estimates of the global, regional, and national morbidity, mortality, and aetiologies of lower respiratory infections in 195 countries, 1990–2016: a systematic analysis for the Global Burden of Disease Study 2016. *Lancet Infect Dis* 2018; **18**: 1191–210.

33 Johnson HL, Deloria-Knoll M, Levine OS, *et al.* Systematic evaluation of serotypes causing invasive pneumococcal disease among children under five: The Pneumococcal Global Serotype Project. *PLOS Med* 2010; **7**: e1000348.

34 Grijalva CG, Nuorti JP, Arbogast PG, Martin SW, Edwards KM, Griffin MR. Decline in pneumonia admissions after routine childhood immunisation with pneumococcal conjugate vaccine in the USA: a time-series analysis. *Lancet* 2007; **369**: 1179–86.

35 Nocedal J, Wright S. Numerical optimization, 2nd edn. New York: Springer-Verlag, 2006 DOI:10.1007/978-0-387-40065-5.

36 Preziosi M, Department of Immunization, Vaccines, and Biologicals (World Health Organization). Update on meningococcal A vaccine development and introduction. Presented at: WHO Product Development for Vaccines Advisory Committee Meeting (PD-VAC). Geneva, Switzerland: WHO, 2015.

37 Kim YA, Kim JJ, Won DJ, Lee K. Seasonal and temperature-associated increase in community-onset Acinetobacter baumannii complex colonization or infection. *Ann Lab Med* 2018; **38**: 266–70.

38 Kritsotakis EI, Groves-Kozhageldiyeva A. A systematic review of the global seasonality of infections caused by *Acinetobacter* species in hospitalized patients. *Clin Microbiol Infect* 2020; **26**: 553–62.

39 GBD Diarrhoeal Diseases Collaborators. Estimates of global, regional, and national morbidity, mortality, and aetiologies of diarrhoeal diseases: a systematic analysis for the Global Burden of Disease Study 2015. *Lancet Infect Dis* 2017; **17**: 909–48.

40 Moher D, Liberati A, Tetzlaff J, Altman DG, The PRISMA Group. Preferred Reporting Items for Systematic Reviews and Meta-Analyses: the PRISMA statement. *PLoS Med* 2009; **6**: e1000097.

41 Browne AJ, Kashef Hamadani BH, Kumaran EAP, *et al.* Drug-resistant enteric fever worldwide, 1990 to 2018: a systematic review and meta-analysis. *BMC Med* 2020; **18**: 1.

42 Clinical and Laboratory Standards Institute. Performance standards for antimicrobial susceptibility testing, 34th ed. Clinical and Laboratory Standards Institute, Wayne, PA https://clsi.org/standards/products/microbiology/documents/m100/ (accessed Aug 29, 2024).

43 Clinical and Laboratory Standards Institute. Performance standards for antimicrobial susceptibility testing. Clinical and Laboratory Standards Institute, Wayne, PA, 2020 https://www.nih.org.pk/wp-content/uploads/2021/02/CLSI-2020.pdf (accessed Aug 27, 2024).

44 The European Committee on Antimicrobial Susceptibility Testing. Breakpoint tables for interpretation of MICs and zone diameters, version 10.0, 2020. https://www.eucast.org/clinical_breakpoints (accessed Aug 27, 2024).

45 Jamison DT, Breman JG, Measham AR, *et al.*, editors. Disease Control Priorities in Developing Countries, 2nd edn. Washington (DC): The International Bank for Reconstruction and Development / The World Bank, 2006 http://www.ncbi.nlm.nih.gov/books/NBK11728/ (accessed Aug 16, 2024).

46 Maina J, Ouma PO, Macharia PM, *et al.* A spatial database of health facilities managed by the public health sector in sub Saharan Africa. *Sci Data* 2019; **6**: 134.

47 Gilbert M, Nicolas G, Cinardi G, *et al.* Global pigs distribution in 2010. 2018. DOI:10.7910/DVN/33N0JG.

48 Gilbert M, Nicolas G, Cinardi G, *et al.* Global distribution data for cattle, buffaloes, horses, sheep, goats, pigs, chickens and ducks in 2010. *Sci Data* 2018; **5**: 180227.

49 Climatic Research Unit, University of East Anglia. CRU TS v. 3.23. Clim. Res. Unit. 2015; published online Sept. https://crudata.uea.ac.uk/cru/data/hrg/cru_ts_3.23/ (accessed Sept 27, 2021).

50 Cassini A, Högberg LD, Plachouras D, *et al.* Attributable deaths and disability-adjusted life-years caused by infections with antibiotic-resistant bacteria in the EU and the European Economic Area in 2015: a population-level modelling analysis. *Lancet Infect Dis* 2019; **19**: 56–66.

51 Zhang J, Yu KF. What's the relative risk? A method of correcting the odds ratio in cohort studies of common outcomes. *JAMA* 1998; **280**: 1690–1.

52 Rajendran NB, Mutters NT, Marasca G, *et al.* Mandatory surveillance and outbreaks reporting of the WHO priority pathogens for research & discovery of new antibiotics in European countries. *Clin Microbiol Infect* 2020; **26**: 943.e1-943.e6.

53 Lastuka A, Breshock MR, McHugh TA, Sogge WT, Swart V, Dieleman JL. U.S. dementia care spending by state: 2010–2019. *Alzheimers Dement* 2024; **20**: 2742–51.

54 Das Gupta P. Standardization and decomposition of rates: a user's manual. U.S. Government Printing Office, Washington, DC: U.S. Bureau of the Census, Current Population Reports, 1993 https://www2.census.gov/library/publications/1993/demographics/p23-186.pdf (accessed Aug 29, 2024).

55 Das Gupta P. Decomposition of the difference between two rates and its consistency when more than two populations are involved. *Math Popul Stud* 2009; **3**: 105–25.

56 Foreman KJ, Marquez N, Dolgert A, *et al.* Forecasting life expectancy, years of life lost, and all-cause and cause-specific mortality for 250 causes of death: reference and alternative scenarios for 2016-40 for 195 countries and territories. *Lancet* 2018; **392**: 2052–90.

57 Vollset SE, Goren E, Yuan C-W, *et al.* Fertility, mortality, migration, and population scenarios for 195 countries and territories from 2017 to 2100: a forecasting analysis for the Global Burden of Disease Study. *Lancet* 2020; **396**: 1285–306.

58 GBD 2021 Forecasting Collaborators. Burden of disease scenarios for 204 countries and territories, 2022–2050: a forecasting analysis for the Global Burden of Disease Study 2021. *Lancet* 2024; **403**: 2204–56.

59 Antimicrobial Resistance Collaborators. The burden of antimicrobial resistance in the Americas in 2019: a cross-country systematic analysis. *Lancet Reg Health Am* 2023; **25**: 100561.

60 Antimicrobial Resistance Collaborators. The burden of bacterial antimicrobial resistance in the WHO African region in 2019: a cross-country systematic analysis. *Lancet Glob Health* 2024; **12**: e201–16.

61 European Antimicrobial Resistance Collaborators. The burden of bacterial antimicrobial resistance in the WHO European region in 2019: a cross-country systematic analysis. *Lancet Public Health* 2022; **7**: e897–913.

62 GBD 2021 Fertility and Forecasting Collaborators. Global fertility in 204 countries and territories, 1950-2021, with forecasts to 2100: a comprehensive demographic analysis for the Global Burden of Disease Study 2021. *Lancet* 2024; **403**: 2057–99.

63 Vollset SE, Ababneh HS, Abate YH, *et al.* Burden of disease scenarios for 204 countries and territories, 2022–2050: a forecasting analysis for the Global Burden of Disease Study 2021. *The Lancet* 2024; **403**: 2204–56.

64 Stevens GA, Alkema L, Black RE, *et al.* Guidelines for Accurate and Transparent Health Estimates Reporting: the GATHER statement. *The Lancet* 2016; **388**: e19–23.

Section 13: Appendix tables and figures

Figure S1: AMR estimation flowchart

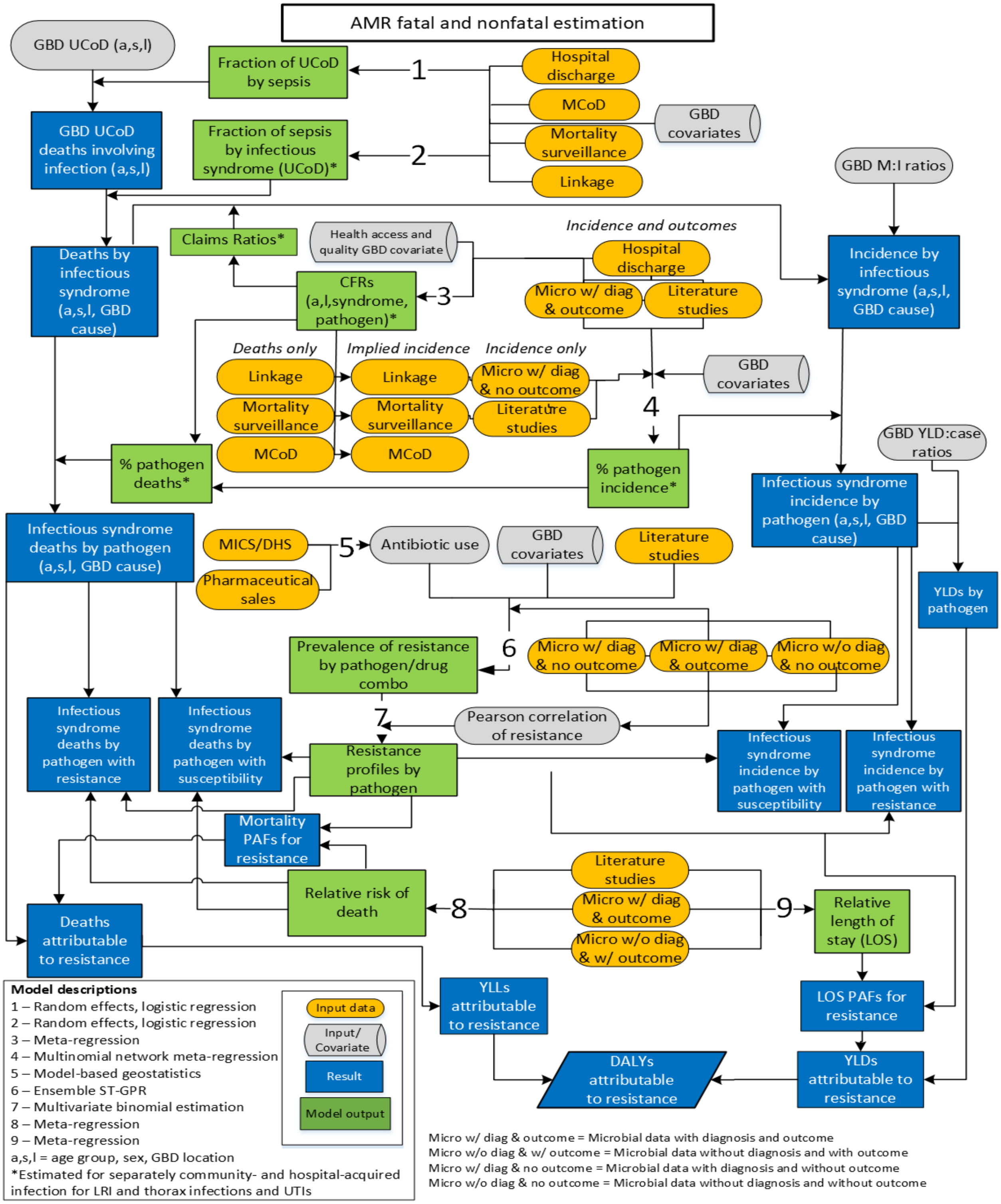

Figure S2: Schematic representation of antimicrobial resistance (AMR) forecasting modelling framework.

AMR = antimicrobial resistance; ART = antiretroviral therapy; YLLs = years of life lost; YLDs = years lived with disability; DALYs = disability-adjusted life years. Blue shading indicates drivers of health burden, green indicates measures of fatal disease burden and demography, purple indicates non-fatal disease burden, and red indicates the total disease burden.

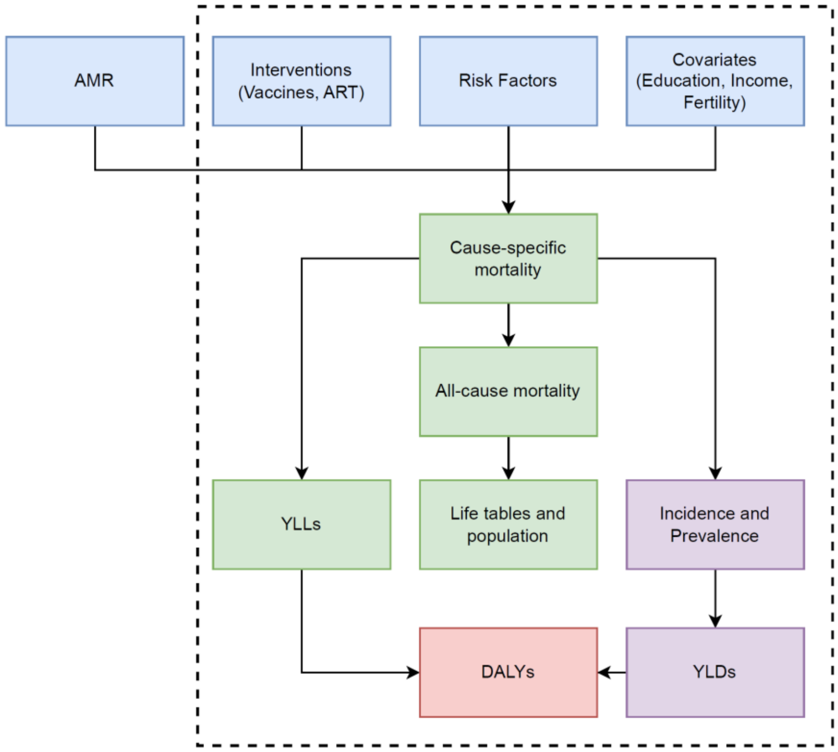

Figure S3: Attributable and associated AMR burden in the reference scenario by GBD super-region, 2022–2050

Shading represents the 95% uncertainty interval. The vertical line is placed at the year 2021 to distinguish estimates from forecasts.

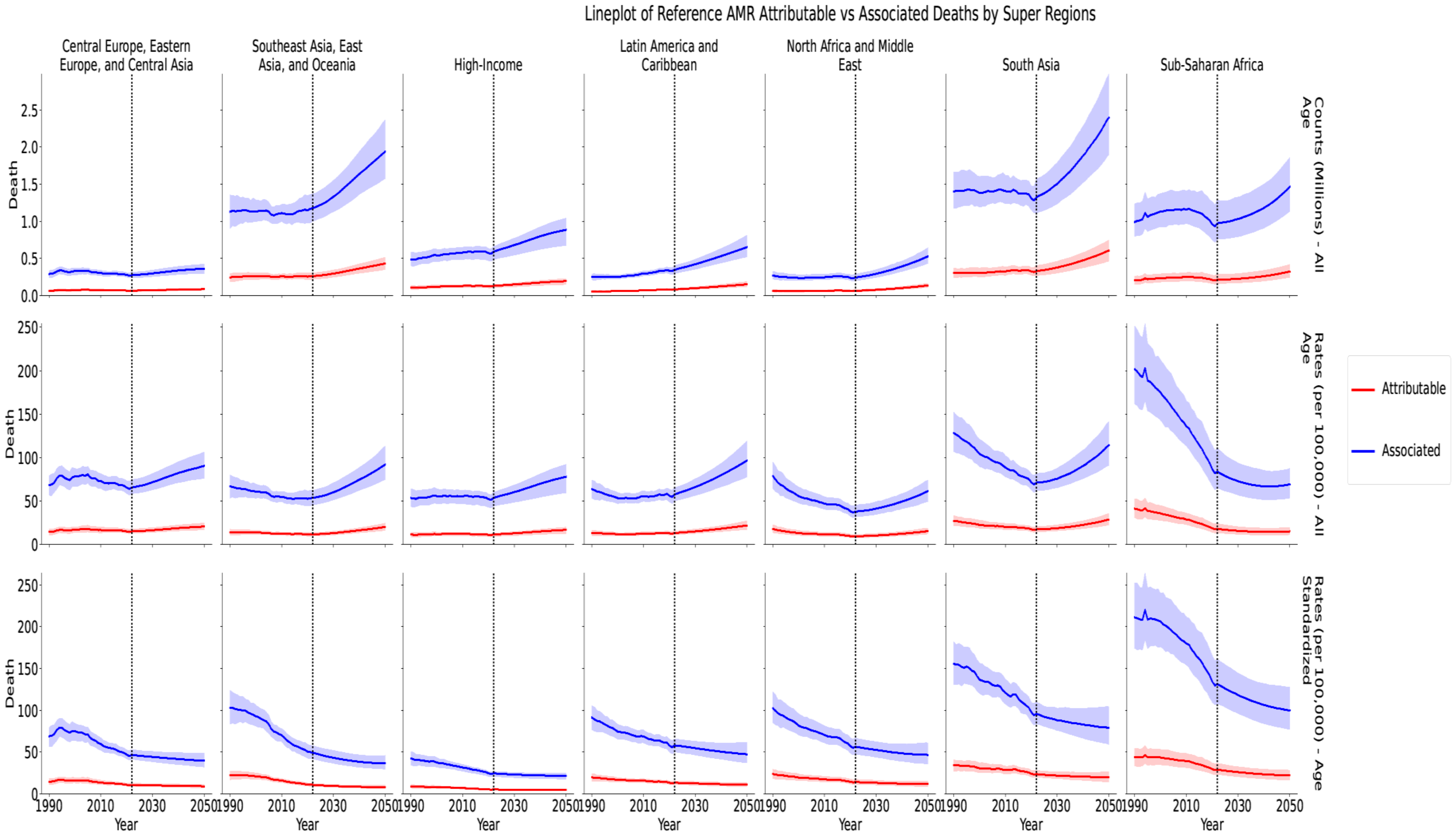

Figure S4: Cumulative deaths averted (in millions) in the (A) Better Care and (B) Gram-Negative Drug scenario compared to reference by location, 2025–2050

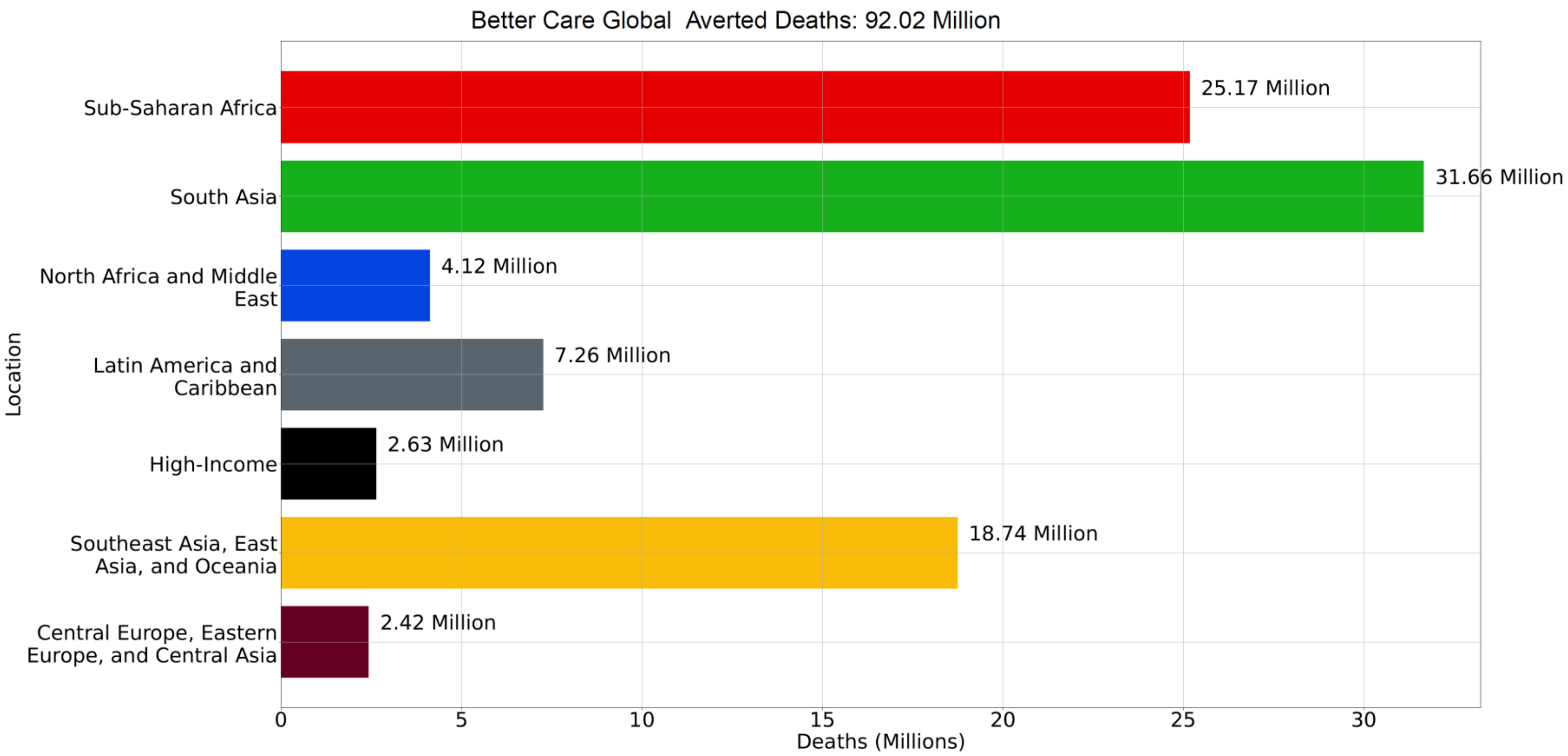

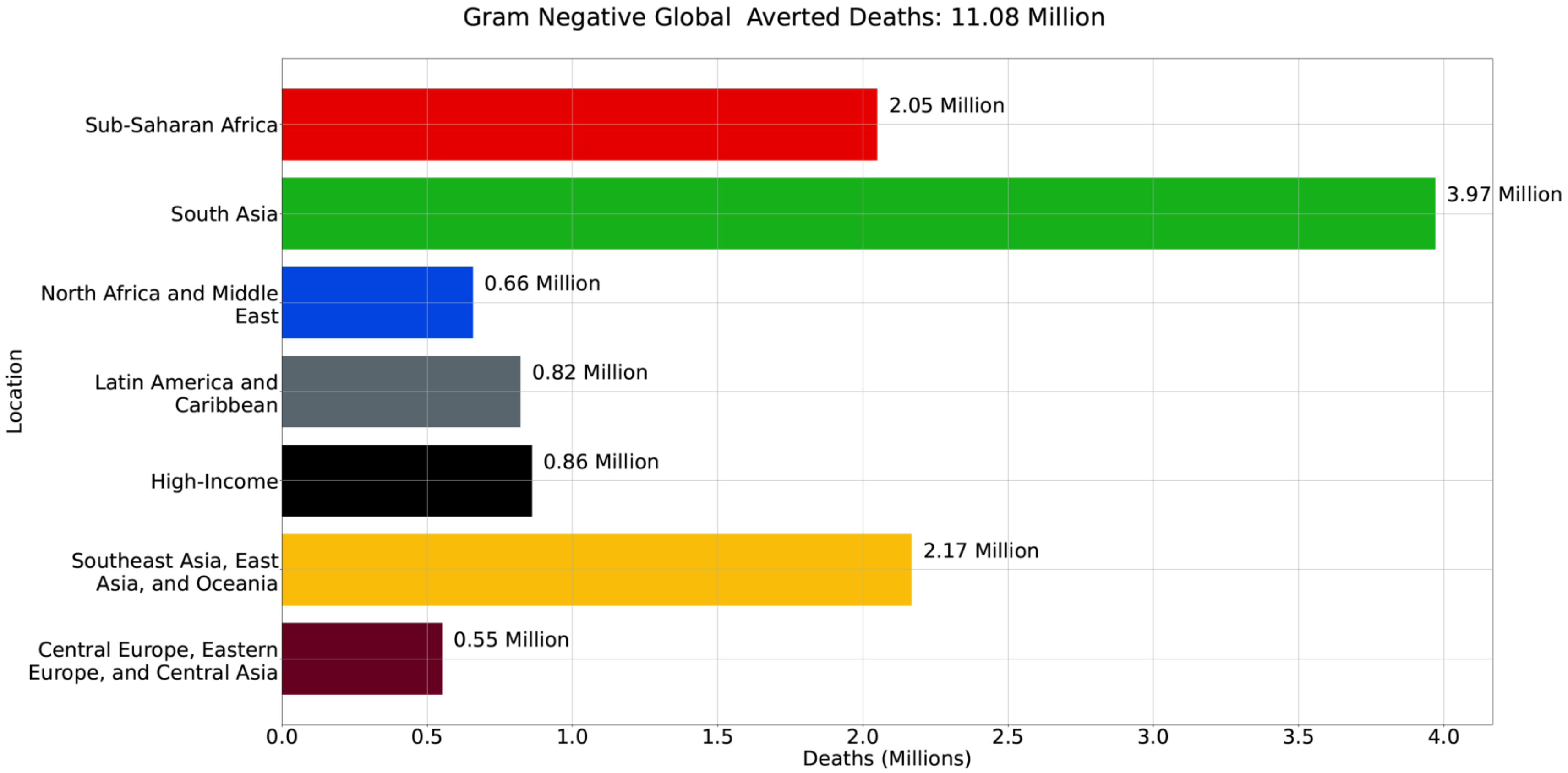
Gram Negative Global Averted Deaths: 11.08 Million
Sub-Saharan Africa
South Asia
North Africa and Middle East
Latin America and Caribbean
High-Income
Southeast Asia, East Asia, and Oceania
Central Europe, Eastern Europe, and Central Asia
Location
2.05 Million
3.97 Million
0.66 Million
0.82 Million
0.86 Million
2.17 Million
0.55 Million
0.0
0.5
1.0
1.5
2.0
2.5
3.0
3.5
4.0
Deaths (Millions)

2298

Table S1: Reported infectious syndromes, contributing infectious syndrome models, and causes of death in modelled syndromes

Reported Infectious Syndromes	Infectious Syndrome Models	Causes in Modelled Syndromes
Bloodstream infections	Bloodstream infections	Cirrhosis and other chronic liver diseases, Chronic kidney disease due to diabetes mellitus type 1, Chronic kidney disease due to diabetes mellitus type 2, Chronic kidney disease due to glomerulonephritis, Chronic kidney disease due to hypertension, Chronic kidney disease due to other and unspecified causes, Other chromosomal abnormalities, Orofacial clefts, Digestive congenital anomalies, Down syndrome, Congenital heart anomalies, Congenital musculoskeletal and limb anomalies, Neural tube defects, Other congenital birth defects, Urogenital congenital anomalies, Atrial fibrillation and flutter, Aortic aneurysm, Alcoholic cardiomyopathy, Other cardiomyopathy, Hypertensive heart disease, Ischemic heart disease, Other cardiovascular and circulatory diseases, Pulmonary Arterial Hypertension, Lower extremity peripheral arterial disease, Intracerebral hemorrhage, Ischemic stroke, Subarachnoid hemorrhage, Non-rheumatic valvular heart disease, Diabetes mellitus type 1, Diabetes mellitus type 2, Gallbladder and biliary diseases, Gastritis and duodenitis, Inguinal, femoral, and abdominal hernia, Ulcerative colitis, Crohn's disease, Paralytic ileus and intestinal obstruction, Other digestive diseases, Pancreatitis, Peptic ulcer disease, Vascular intestinal disorders, Other endocrine, metabolic, blood, and immune disorders, Thyroid diseases, Acute glomerulonephritis, Endometriosis, Uterine fibroids, Other gynecological diseases, Genital prolapse, G6PD deficiency, Other hemoglobinopathies and hemolytic anemias, Sickle cell disorders, Thalassemias, Other unspecified infectious diseases, Non-venomous animal contact, Venomous animal contact, Exposure to forces of nature, Drowning, Electrocution, Falls, Fire, heat, and hot substances, Pulmonary aspiration and foreign body in airway, Foreign body in other body part, Physical violence by firearm, Physical violence by sharp object, Physical violence by other means, Unintentional firearm injuries, Other exposure to mechanical forces, Adverse effects of medical treatment, Environmental heat and cold exposure, Other unintentional injuries (internal), Poisoning by carbon monoxide, Poisoning by other means, Self-harm by firearm, Self-harm by other specified means, Road injuries, Police conflict and executions, Conflict and terrorism, Other intestinal infectious diseases, Ectopic pregnancy, Maternal abortion and miscarriage, Maternal hemorrhage, Maternal hypertensive disorders, Late maternal deaths, Maternal obstructed labor and uterine rupture, Other direct maternal disorders, Maternal sepsis and other maternal infections, Alcohol use disorders, Amphetamine use disorders, Cocaine use disorders, Opioid use disorders, Other drug use disorders, Anorexia nervosa, Other musculoskeletal disorders, Rheumatoid arthritis, Myelodysplastic, myeloproliferative, and other hematopoietic neoplasms, Other benign and in situ neoplasms, Bladder cancer, Malignant neoplasm of bone and articular cartilage, Brain and central nervous system cancer, Breast cancer, Cervical cancer, Colon and rectum cancer, Esophageal cancer, Other eye cancers, Retinoblastoma, Gallbladder and biliary tract cancer, Hodgkin lymphoma, Kidney cancer, Larynx cancer, Leukemia, Liver cancer, Tracheal, bronchus, and lung cancer, Non-Hodgkin lymphoma, Malignant skin melanoma, Mesothelioma, Lip and oral cavity cancer, Multiple myeloma, Nasopharynx cancer, Neuroblastoma and other peripheral nervous cell tumors, Non-melanoma skin cancer, Other malignant neoplasms, Other pharynx cancer, Ovarian cancer, Pancreatic cancer, Prostate cancer, Stomach cancer, Testicular cancer, Thyroid cancer, Soft tissue and other extraosseous sarcomas, Uterine cancer, Neonatal encephalopathy due to birth asphyxia and trauma, Hemolytic disease and other neonatal jaundice, Other neonatal disorders, Neonatal preterm birth, Neonatal sepsis and other neonatal infections, Idiopathic epilepsy, Multiple sclerosis, Motor neuron disease, Other neurological disorders, Parkinson's disease, Other nutritional deficiencies, Protein-energy malnutrition, Otitis media, Asthma, Chronic obstructive pulmonary disease, Interstitial lung disease and pulmonary sarcoidosis, Other chronic respiratory diseases, Pneumoconiosis, Decubitus ulcer, Other skin and subcutaneous diseases, Upper respiratory infections, Other urinary diseases, Urolithiasis
Meningitis	Meningitis	Chronic kidney disease due to diabetes mellitus type 1, Chronic kidney disease due to diabetes mellitus type 2, Intracerebral hemorrhage, Ischemic stroke, Subarachnoid hemorrhage, Diabetes mellitus type 1, Diabetes mellitus type 2, G6PD deficiency, Other hemoglobinopathies and hemolytic anemias, Sickle cell disorders, Thalassemias, Other unspecified infectious diseases, Non-venomous animal contact, Venomous animal contact, Exposure to forces of nature, Falls, Fire, heat, and hot substances, Physical violence by firearm, Physical violence by sharp object, Physical violence by other means, Unintentional firearm injuries, Other exposure to mechanical forces, Adverse effects of medical treatment, Environmental heat and cold exposure, Other unintentional injuries (internal), Self-harm by firearm, Self-harm by other specified means, Road injuries, Police conflict and executions, Conflict and terrorism, Other intestinal infectious diseases, Late maternal deaths,

		Meningitis, Alcohol use disorders, Amphetamine use disorders, Cocaine use disorders, Opioid use disorders, Other drug use disorders, Myelodysplastic, myeloproliferative, and other hematopoietic neoplasms, Other benign and in situ neoplasms, Bladder cancer, Malignant neoplasm of bone and articular cartilage, Brain and central nervous system cancer, Breast cancer, Cervical cancer, Colon and rectum cancer, Esophageal cancer, Other eye cancers, Retinoblastoma, Gallbladder and biliary tract cancer, Hodgkin lymphoma, Kidney cancer, Larynx cancer, Leukemia, Liver cancer, Tracheal, bronchus, and lung cancer, Non-Hodgkin lymphoma, Malignant skin melanoma, Mesothelioma, Lip and oral cavity cancer, Multiple myeloma, Nasopharynx cancer, Neuroblastoma and other peripheral nervous cell tumors, Non-melanoma skin cancer, Other malignant neoplasms, Other pharynx cancer, Ovarian cancer, Pancreatic cancer, Prostate cancer, Stomach cancer, Testicular cancer, Thyroid cancer, Soft tissue and other extraosseous sarcomas, Uterine cancer, Neonatal encephalopathy due to birth asphyxia and trauma, Hemolytic disease and other neonatal jaundice, Other neonatal disorders, Neonatal preterm birth, Idiopathic epilepsy, Multiple sclerosis, Other neurological disorders, Parkinson's disease, Protein-energy malnutrition, Otitis media
	Encephalitis	Intracerebral hemorrhage, Ischemic stroke, Subarachnoid hemorrhage, Encephalitis, Other unspecified infectious diseases, Late maternal deaths, Alcohol use disorders, Anorexia nervosa, Myelodysplastic, myeloproliferative, and other hematopoietic neoplasms, Other benign and in situ neoplasms, Bladder cancer, Malignant neoplasm of bone and articular cartilage, Brain and central nervous system cancer, Breast cancer, Cervical cancer, Colon and rectum cancer, Esophageal cancer, Other eye cancers, Retinoblastoma, Gallbladder and biliary tract cancer, Hodgkin lymphoma, Kidney cancer, Larynx cancer, Leukemia, Liver cancer, Tracheal, bronchus, and lung cancer, Non-Hodgkin lymphoma, Malignant skin melanoma, Mesothelioma, Lip and oral cavity cancer, Multiple myeloma, Nasopharynx cancer, Neuroblastoma and other peripheral nervous cell tumors, Non-melanoma skin cancer, Other malignant neoplasms, Other pharynx cancer, Ovarian cancer, Pancreatic cancer, Prostate cancer, Stomach cancer, Testicular cancer, Thyroid cancer, Soft tissue and other extraosseous sarcomas, Uterine cancer, Neonatal encephalopathy due to birth asphyxia and trauma, Other neonatal disorders, Neonatal preterm birth, Idiopathic epilepsy, Multiple sclerosis, Motor neuron disease, Other neurological disorders, Parkinson's disease, Protein-energy malnutrition, Otitis media
	Myelitis, meningoencephalitis, and other central nervous system infections	Neural tube defects, G6PD deficiency, Other hemoglobinopathies and hemolytic anemias, Sickle cell disorders, Thalassemias, Other unspecified infectious diseases, Non-venomous animal contact, Venomous animal contact, Exposure to forces of nature, Falls, Fire, heat, and hot substances, Pulmonary aspiration and foreign body in airway, Foreign body in other body part, Physical violence by firearm, Physical violence by sharp object, Physical violence by other means, Unintentional firearm injuries, Other exposure to mechanical forces, Adverse effects of medical treatment, Environmental heat and cold exposure, Other unintentional injuries (internal), Self-harm by firearm, Self-harm by other specified means, Road injuries, Police conflict and executions, Conflict and terrorism, Late maternal deaths, Alcohol use disorders, Amphetamine use disorders, Cocaine use disorders, Opioid use disorders, Other drug use disorders, Anorexia nervosa, Myelodysplastic, myeloproliferative, and other hematopoietic neoplasms, Other benign and in situ neoplasms, Bladder cancer, Malignant neoplasm of bone and articular cartilage, Brain and central nervous system cancer, Breast cancer, Cervical cancer, Colon and rectum cancer, Esophageal cancer, Other eye cancers, Retinoblastoma, Gallbladder and biliary tract cancer, Hodgkin lymphoma, Kidney cancer, Larynx cancer, Leukemia, Liver cancer, Tracheal, bronchus, and lung cancer, Non-Hodgkin lymphoma, Malignant skin melanoma, Mesothelioma, Lip and oral cavity cancer, Multiple myeloma, Nasopharynx cancer, Neuroblastoma and other peripheral nervous cell tumors, Non-melanoma skin cancer, Other malignant neoplasms, Other pharynx cancer, Ovarian cancer, Pancreatic cancer, Prostate cancer, Stomach cancer, Testicular cancer, Thyroid cancer, Soft tissue and other extraosseous sarcomas, Uterine cancer, Neonatal encephalopathy due to birth asphyxia and trauma, Hemolytic disease and other neonatal jaundice, Other neonatal disorders, Neonatal preterm birth, Idiopathic epilepsy, Multiple sclerosis, Motor neuron disease, Other neurological disorders, Parkinson's disease, Other nutritional deficiencies, Protein-energy malnutrition, Otitis media
	Eye infections	Chronic kidney disease due to glomerulonephritis, Chronic kidney disease due to hypertension, Chronic kidney disease due to other and unspecified causes, Diabetes mellitus type 1, Diabetes mellitus type 2, Other unspecified infectious diseases, Non-venomous animal contact, Venomous animal contact, Exposure to forces of nature, Falls, Fire, heat, and hot substances, Physical violence by firearm, Physical violence by sharp object, Physical violence by other means, Unintentional firearm injuries, Other exposure to mechanical forces, Adverse effects of medical treatment, Environmental heat and cold exposure, Other unintentional injuries (internal), Self-harm by firearm, Self-harm by other specified means, Road injuries, Police conflict and executions, Conflict and terrorism, Late maternal deaths, Myelodysplastic, myeloproliferative, and other hematopoietic neoplasms, Other benign and in situ neoplasms, Bladder cancer, Malignant neoplasm of bone and articular cartilage, Brain and central nervous system cancer, Breast cancer, Cervical cancer, Colon and rectum cancer, Esophageal cancer, Other eye cancers, Retinoblastoma, Gallbladder and biliary tract cancer, Hodgkin lymphoma, Kidney cancer, Larynx cancer, Leukemia, Liver cancer, Tracheal, bronchus, and lung cancer, Non-Hodgkin lymphoma, Malignant skin melanoma, Mesothelioma, Lip and oral cavity cancer,

		Multiple myeloma, Nasopharynx cancer, Neuroblastoma and other peripheral nervous cell tumors, Non-melanoma skin cancer, Other malignant neoplasms, Other pharynx cancer, Ovarian cancer, Pancreatic cancer, Prostate cancer, Stomach cancer, Testicular cancer, Thyroid cancer, Soft tissue and other extraosseous sarcomas, Uterine cancer, Neonatal encephalopathy due to birth asphyxia and trauma, Hemolytic disease and other neonatal jaundice, Other neonatal disorders, Neonatal preterm birth, Other nutritional deficiencies, Protein-energy malnutrition, Other skin and subcutaneous diseases
	Oral infections	Orofacial clefts, Atrial fibrillation and flutter, Aortic aneurysm, Alcoholic cardiomyopathy, Other cardiomyopathy, Hypertensive heart disease, Ischemic heart disease, Other cardiovascular and circulatory diseases, Pulmonary Arterial Hypertension, Lower extremity peripheral arterial disease, Intracerebral hemorrhage, Ischemic stroke, Subarachnoid hemorrhage, Diabetes mellitus type 1, Diabetes mellitus type 2, Gastritis and duodenitis, Peptic ulcer disease, Other hemoglobinopathies and hemolytic anemias, Sickle cell disorders, Thalassemias, Other unspecified infectious diseases, Non-venomous animal contact, Venomous animal contact, Exposure to forces of nature, Electrocution, Falls, Fire, heat, and hot substances, Pulmonary aspiration and foreign body in airway, Foreign body in other body part, Physical violence by firearm, Physical violence by sharp object, Physical violence by other means, Unintentional firearm injuries, Other exposure to mechanical forces, Adverse effects of medical treatment, Environmental heat and cold exposure, Other unintentional injuries (internal), Self-harm by firearm, Self-harm by other specified means, Road injuries, Police conflict and executions, Conflict and terrorism, Late maternal deaths, Anorexia nervosa, Myelodysplastic, myeloproliferative, and other hematopoietic neoplasms, Other benign and in situ neoplasms, Bladder cancer, Malignant neoplasm of bone and articular cartilage, Brain and central nervous system cancer, Breast cancer, Cervical cancer, Colon and rectum cancer, Esophageal cancer, Other eye cancers, Retinoblastoma, Gallbladder and biliary tract cancer, Hodgkin lymphoma, Kidney cancer, Larynx cancer, Leukemia, Liver cancer, Tracheal, bronchus, and lung cancer, Non-Hodgkin lymphoma, Malignant skin melanoma, Mesothelioma, Lip and oral cavity cancer, Multiple myeloma, Nasopharynx cancer, Neuroblastoma and other peripheral nervous cell tumors, Non-melanoma skin cancer, Other malignant neoplasms, Other pharynx cancer, Ovarian cancer, Pancreatic cancer, Prostate cancer, Stomach cancer, Testicular cancer, Thyroid cancer, Soft tissue and other extraosseous sarcomas, Uterine cancer, Neonatal encephalopathy due to birth asphyxia and trauma, Hemolytic disease and other neonatal jaundice, Other neonatal disorders, Neonatal preterm birth, Idiopathic epilepsy, Multiple sclerosis, Motor neuron disease, Other neurological disorders, Parkinson's disease, Other nutritional deficiencies, Protein-energy malnutrition, Asthma, Chronic obstructive pulmonary disease, Interstitial lung disease and pulmonary sarcoidosis, Other chronic respiratory diseases, Other skin and subcutaneous diseases
	Upper respiratory infections	Chronic kidney disease due to diabetes mellitus type 1, Chronic kidney disease due to diabetes mellitus type 2, Chronic kidney disease due to glomerulonephritis, Chronic kidney disease due to hypertension, Chronic kidney disease due to other and unspecified causes, Other chromosomal abnormalities, Orofacial clefts, Digestive congenital anomalies, Down syndrome, Congenital heart anomalies, Congenital musculoskeletal and limb anomalies, Neural tube defects, Other congenital birth defects, Urogenital congenital anomalies, Atrial fibrillation and flutter, Aortic aneurysm, Alcoholic cardiomyopathy, Other cardiomyopathy, Hypertensive heart disease, Ischemic heart disease, Other cardiovascular and circulatory diseases, Pulmonary Arterial Hypertension, Lower extremity peripheral arterial disease, Intracerebral hemorrhage, Ischemic stroke, Subarachnoid hemorrhage, Diabetes mellitus type 1, Diabetes mellitus type 2, Gallbladder and biliary diseases, Gastritis and duodenitis, Inguinal, femoral, and abdominal hernia, Other digestive diseases, Acute glomerulonephritis, Other gynecological diseases, G6PD deficiency, Other hemoglobinopathies and hemolytic anemias, Sickle cell disorders, Thalassemias, Other unspecified infectious diseases, Non-venomous animal contact, Venomous animal contact, Exposure to forces of nature, Drowning, Falls, Fire, heat, and hot substances, Pulmonary aspiration and foreign body in airway, Foreign body in other body part, Physical violence by firearm, Physical violence by sharp object, Physical violence by other means, Unintentional firearm injuries, Other exposure to mechanical forces, Adverse effects of medical treatment, Environmental heat and cold exposure, Other unintentional injuries (internal), Self-harm by firearm, Self-harm by other specified means, Road injuries, Police conflict and executions, Conflict and terrorism, Late maternal deaths, Alcohol use disorders, Amphetamine use disorders, Cocaine use disorders, Opioid use disorders, Other drug use disorders, Anorexia nervosa, Myelodysplastic, myeloproliferative, and other hematopoietic neoplasms, Other benign and in situ neoplasms, Bladder cancer, Malignant neoplasm of bone and articular cartilage, Brain and central nervous system cancer, Breast cancer, Cervical cancer, Colon and rectum cancer, Esophageal cancer, Other eye cancers, Retinoblastoma, Gallbladder and biliary tract cancer, Hodgkin lymphoma, Kidney cancer, Larynx cancer, Leukemia, Liver cancer, Tracheal, bronchus, and lung cancer, Non-Hodgkin lymphoma, Malignant skin melanoma, Mesothelioma, Lip and oral cavity cancer, Multiple myeloma, Nasopharynx cancer, Neuroblastoma and other peripheral nervous cell tumors, Non-melanoma skin cancer, Other malignant neoplasms, Other pharynx cancer, Ovarian cancer, Pancreatic cancer, Prostate cancer, Stomach cancer, Testicular cancer, Thyroid

		cancer, Soft tissue and other extraosseous sarcomas, Uterine cancer, Neonatal encephalopathy due to birth asphyxia and trauma, Hemolytic disease and other neonatal jaundice, Other neonatal disorders, Neonatal preterm birth, Idiopathic epilepsy, Multiple sclerosis, Motor neuron disease, Other neurological disorders, Parkinson's disease, Other nutritional deficiencies, Protein-energy malnutrition, Otitis media, Asthma, Chronic obstructive pulmonary disease, Interstitial lung disease and pulmonary sarcoidosis, Other chronic respiratory diseases, Pneumoconiosis, Upper respiratory infections
Lower respiratory infections	Lower respiratory infections	Cirrhosis and other chronic liver diseases, Chronic kidney disease due to diabetes mellitus type 1, Chronic kidney disease due to diabetes mellitus type 2, Chronic kidney disease due to glomerulonephritis, Chronic kidney disease due to hypertension, Chronic kidney disease due to other and unspecified causes, Other chromosomal abnormalities, Orofacial clefts, Digestive congenital anomalies, Down syndrome, Congenital heart anomalies, Congenital musculoskeletal and limb anomalies, Neural tube defects, Other congenital birth defects, Urogenital congenital anomalies, Atrial fibrillation and flutter, Aortic aneurysm, Alcoholic cardiomyopathy, Other cardiomyopathy, Hypertensive heart disease, Ischemic heart disease, Other cardiovascular and circulatory diseases, Pulmonary Arterial Hypertension, Lower extremity peripheral arterial disease, Intracerebral hemorrhage, Ischemic stroke, Subarachnoid hemorrhage, Non-rheumatic valvular heart disease, Diabetes mellitus type 1, Diabetes mellitus type 2, Gallbladder and biliary diseases, Gastritis and duodenitis, Inguinal, femoral, and abdominal hernia, Ulcerative colitis, Crohn's disease, Paralytic ileus and intestinal obstruction, Other digestive diseases, Pancreatitis, Peptic ulcer disease, Vascular intestinal disorders, Other endocrine, metabolic, blood, and immune disorders, Thyroid diseases, Acute glomerulonephritis, Endometriosis, Uterine fibroids, Other gynecological diseases, Genital prolapse, G6PD deficiency, Other hemoglobinopathies and hemolytic anemias, Sickle cell disorders, Thalassemias, Other unspecified infectious diseases, Non-venomous animal contact, Venomous animal contact, Exposure to forces of nature, Drowning, Electrocution, Falls, Fire, heat, and hot substances, Pulmonary aspiration and foreign body in airway, Foreign body in other body part, Physical violence by firearm, Physical violence by sharp object, Physical violence by other means, Unintentional firearm injuries, Other exposure to mechanical forces, Adverse effects of medical treatment, Environmental heat and cold exposure, Other unintentional injuries (internal), Poisoning by carbon monoxide, Poisoning by other means, Self-harm by firearm, Self-harm by other specified means, Road injuries, Police conflict and executions, Conflict and terrorism, Other intestinal infectious diseases, Ectopic pregnancy, Maternal abortion and miscarriage, Maternal hemorrhage, Maternal hypertensive disorders, Late maternal deaths, Maternal obstructed labor and uterine rupture, Other direct maternal disorders, Alcohol use disorders, Amphetamine use disorders, Cocaine use disorders, Opioid use disorders, Other drug use disorders, Anorexia nervosa, Other musculoskeletal disorders, Rheumatoid arthritis, Myelodysplastic, myeloproliferative, and other hematopoietic neoplasms, Other benign and in situ neoplasms, Bladder cancer, Malignant neoplasm of bone and articular cartilage, Brain and central nervous system cancer, Breast cancer, Cervical cancer, Colon and rectum cancer, Esophageal cancer, Other eye cancers, Retinoblastoma, Gallbladder and biliary tract cancer, Hodgkin lymphoma, Kidney cancer, Larynx cancer, Leukemia, Liver cancer, Tracheal, bronchus, and lung cancer, Non-Hodgkin lymphoma, Malignant skin melanoma, Mesothelioma, Lip and oral cavity cancer, Multiple myeloma, Nasopharynx cancer, Neuroblastoma and other peripheral nervous cell tumors, Non-melanoma skin cancer, Other malignant neoplasms, Other pharynx cancer, Ovarian cancer, Pancreatic cancer, Prostate cancer, Stomach cancer, Testicular cancer, Thyroid cancer, Soft tissue and other extraosseous sarcomas, Uterine cancer, Neonatal encephalopathy due to birth asphyxia and trauma, Hemolytic disease and other neonatal jaundice, Other neonatal disorders, Neonatal preterm birth, Idiopathic epilepsy, Multiple sclerosis, Motor neuron disease, Other neurological disorders, Parkinson's disease, Other nutritional deficiencies, Protein-energy malnutrition, Otitis media, Asthma, Chronic obstructive pulmonary disease, Interstitial lung disease and pulmonary sarcoidosis, Other chronic respiratory diseases, Pneumoconiosis, Decubitus ulcer, Other skin and subcutaneous diseases, Upper respiratory infections, Other urinary diseases, Urolithiasis
	Other unspecified respiratory site infections	Chronic kidney disease due to diabetes mellitus type 1, Chronic kidney disease due to diabetes mellitus type 2, Chronic kidney disease due to glomerulonephritis, Chronic kidney disease due to hypertension, Chronic kidney disease due to other and unspecified causes, Other chromosomal abnormalities, Orofacial clefts, Digestive congenital anomalies, Down syndrome, Congenital heart anomalies, Congenital musculoskeletal and limb anomalies, Neural tube defects, Other congenital birth defects, Urogenital congenital anomalies, Atrial fibrillation and flutter, Aortic aneurysm, Alcoholic cardiomyopathy, Other cardiomyopathy, Hypertensive heart disease, Ischemic heart disease, Other cardiovascular and circulatory diseases, Pulmonary Arterial Hypertension, Lower extremity peripheral arterial disease, Intracerebral hemorrhage, Ischemic stroke, Subarachnoid hemorrhage, Non-rheumatic valvular heart disease, Diabetes mellitus type 1, Diabetes mellitus type 2, Gallbladder and biliary diseases, Gastritis and duodenitis, Inguinal, femoral, and abdominal hernia, Ulcerative colitis, Crohn's disease, Paralytic ileus and intestinal obstruction, Other digestive diseases, Pancreatitis, Peptic ulcer disease, Vascular intestinal disorders, Other endocrine, metabolic, blood, and immune disorders, Thyroid diseases, Acute glomerulonephritis, Endometriosis, Uterine fibroids, Other

		gynecological diseases, Genital prolapse, G6PD deficiency, Other hemoglobinopathies and hemolytic anemias, Sickle cell disorders, Thalassemias, Other unspecified infectious diseases, Non-venomous animal contact, Venomous animal contact, Exposure to forces of nature, Drowning, Electrocution, Falls, Fire, heat, and hot substances, Pulmonary aspiration and foreign body in airway, Foreign body in other body part, Physical violence by firearm, Physical violence by sharp object, Physical violence by other means, Unintentional firearm injuries, Other exposure to mechanical forces, Adverse effects of medical treatment, Environmental heat and cold exposure, Other unintentional injuries (internal), Poisoning by carbon monoxide, Poisoning by other means, Self-harm by firearm, Self-harm by other specified means, Road injuries, Police conflict and executions, Conflict and terrorism, Other intestinal infectious diseases, Ectopic pregnancy, Maternal abortion and miscarriage, Maternal hypertensive disorders, Late maternal deaths, Maternal obstructed labor and uterine rupture, Other direct maternal disorders, Alcohol use disorders, Amphetamine use disorders, Cocaine use disorders, Opioid use disorders, Other drug use disorders, Anorexia nervosa, Other musculoskeletal disorders, Rheumatoid arthritis, Myelodysplastic, myeloproliferative, and other hematopoietic neoplasms, Other benign and in situ neoplasms, Bladder cancer, Malignant neoplasm of bone and articular cartilage, Brain and central nervous system cancer, Breast cancer, Cervical cancer, Colon and rectum cancer, Esophageal cancer, Other eye cancers, Retinoblastoma, Gallbladder and biliary tract cancer, Hodgkin lymphoma, Kidney cancer, Larynx cancer, Leukemia, Liver cancer, Tracheal, bronchus, and lung cancer, Non-Hodgkin lymphoma, Malignant skin melanoma, Mesothelioma, Lip and oral cavity cancer, Multiple myeloma, Nasopharynx cancer, Neuroblastoma and other peripheral nervous cell tumors, Non-melanoma skin cancer, Other malignant neoplasms, Other pharynx cancer, Ovarian cancer, Pancreatic cancer, Prostate cancer, Stomach cancer, Testicular cancer, Thyroid cancer, Soft tissue and other extraosseous sarcomas, Uterine cancer, Neonatal encephalopathy due to birth asphyxia and trauma, Hemolytic disease and other neonatal jaundice, Other neonatal disorders, Neonatal preterm birth, Idiopathic epilepsy, Multiple sclerosis, Motor neuron disease, Other neurological disorders, Parkinson's disease, Other nutritional deficiencies, Protein-energy malnutrition, Otitis media, Asthma, Chronic obstructive pulmonary disease, Interstitial lung disease and pulmonary sarcoidosis, Other chronic respiratory diseases, Pneumoconiosis, Upper respiratory infections, Other urinary diseases, Urolithiasis
Endocarditis	Endocarditis	Chronic kidney disease due to diabetes mellitus type 1, Chronic kidney disease due to diabetes mellitus type 2, Chronic kidney disease due to glomerulonephritis, Chronic kidney disease due to hypertension, Chronic kidney disease due to other and unspecified causes, Down syndrome, Congenital heart anomalies, Atrial fibrillation and flutter, Aortic aneurysm, Alcoholic cardiomyopathy, Other cardiomyopathy, Endocarditis, Hypertensive heart disease, Ischemic heart disease, Other cardiovascular and circulatory diseases, Pulmonary Arterial Hypertension, Lower extremity peripheral arterial disease, Intracerebral hemorrhage, Ischemic stroke, Subarachnoid hemorrhage, Non-rheumatic valvular heart disease, Diabetes mellitus type 1, Diabetes mellitus type 2, Acute glomerulonephritis, G6PD deficiency, Other hemoglobinopathies and hemolytic anemias, Sickle cell disorders, Thalassemias, Other unspecified infectious diseases, Non-venomous animal contact, Venomous animal contact, Exposure to forces of nature, Falls, Fire, heat, and hot substances, Physical violence by firearm, Physical violence by sharp object, Physical violence by other means, Unintentional firearm injuries, Other exposure to mechanical forces, Adverse effects of medical treatment, Environmental heat and cold exposure, Other unintentional injuries (internal), Self-harm by firearm, Self-harm by other specified means, Road injuries, Police conflict and executions, Conflict and terrorism, Late maternal deaths, Alcohol use disorders, Amphetamine use disorders, Cocaine use disorders, Opioid use disorders, Other drug use disorders, Other musculoskeletal disorders, Rheumatoid arthritis, Myelodysplastic, myeloproliferative, and other hematopoietic neoplasms, Other benign and in situ neoplasms, Bladder cancer, Malignant neoplasm of bone and articular cartilage, Brain and central nervous system cancer, Breast cancer, Cervical cancer, Colon and rectum cancer, Esophageal cancer, Other eye cancers, Retinoblastoma, Gallbladder and biliary tract cancer, Hodgkin lymphoma, Kidney cancer, Larynx cancer, Leukemia, Liver cancer, Tracheal, bronchus, and lung cancer, Non-Hodgkin lymphoma, Malignant skin melanoma, Mesothelioma, Lip and oral cavity cancer, Multiple myeloma, Nasopharynx cancer, Neuroblastoma and other peripheral nervous cell tumors, Non-melanoma skin cancer, Other malignant neoplasms, Other pharynx cancer, Ovarian cancer, Pancreatic cancer, Prostate cancer, Stomach cancer, Testicular cancer, Thyroid cancer, Soft tissue and other extraosseous sarcomas, Uterine cancer, Neonatal encephalopathy due to birth asphyxia and trauma, Hemolytic disease and other neonatal jaundice, Other neonatal disorders, Neonatal preterm birth, Other nutritional deficiencies, Protein-energy malnutrition
	Carditis, myocarditis,	Chronic kidney disease due to diabetes mellitus type 1, Chronic kidney disease due to diabetes mellitus type 2, Chronic kidney disease due to glomerulonephritis, Chronic kidney disease due to hypertension, Chronic kidney disease due to other and unspecified causes, Congenital heart anomalies, Atrial fibrillation and flutter, Aortic aneurysm, Alcoholic cardiomyopathy, Myocarditis, Other cardiomyopathy, Hypertensive heart disease, Ischemic heart disease, Other cardiovascular and circulatory diseases, Pulmonary Arterial Hypertension, Lower extremity peripheral

	and pericarditis	arterial disease, Intracerebral hemorrhage, Ischemic stroke, Subarachnoid hemorrhage, Non-rheumatic valvular heart disease, Diabetes mellitus type 1, Diabetes mellitus type 2, Acute glomerulonephritis, Other unspecified infectious diseases, Fire, heat, and hot substances, Foreign body in other body part, Physical violence by firearm, Physical violence by sharp object, Physical violence by other means, Unintentional firearm injuries, Other exposure to mechanical forces, Adverse effects of medical treatment, Environmental heat and cold exposure, Other unintentional injuries (internal), Self-harm by firearm, Self-harm by other specified means, Road injuries, Police conflict and executions, Conflict and terrorism, Late maternal deaths, Alcohol use disorders, Amphetamine use disorders, Cocaine use disorders, Opioid use disorders, Other drug use disorders, Other musculoskeletal disorders, Rheumatoid arthritis, Myelodysplastic, myeloproliferative, and other hematopoietic neoplasms, Other benign and in situ neoplasms, Bladder cancer, Malignant neoplasm of bone and articular cartilage, Brain and central nervous system cancer, Breast cancer, Cervical cancer, Colon and rectum cancer, Esophageal cancer, Other eye cancers, Retinoblastoma, Gallbladder and biliary tract cancer, Hodgkin lymphoma, Kidney cancer, Larynx cancer, Leukemia, Liver cancer, Tracheal, bronchus, and lung cancer, Non-Hodgkin lymphoma, Malignant skin melanoma, Mesothelioma, Lip and oral cavity cancer, Multiple myeloma, Nasopharynx cancer, Neuroblastoma and other peripheral nervous cell tumors, Non-melanoma skin cancer, Other malignant neoplasms, Other pharynx cancer, Ovarian cancer, Pancreatic cancer, Prostate cancer, Stomach cancer, Testicular cancer, Thyroid cancer, Soft tissue and other extraosseous sarcomas, Uterine cancer, Neonatal encephalopathy due to birth asphyxia and trauma, Hemolytic disease and other neonatal jaundice, Other neonatal disorders, Neonatal preterm birth, Other nutritional deficiencies, Protein-energy malnutrition
Peritoneal and intra-abdominal infections	Peritoneal and intra-abdominal infections	Cirrhosis and other chronic liver diseases, Chronic kidney disease due to diabetes mellitus type 1, Chronic kidney disease due to diabetes mellitus type 2, Chronic kidney disease due to glomerulonephritis, Chronic kidney disease due to hypertension, Chronic kidney disease due to other and unspecified causes, Digestive congenital anomalies, Other congenital birth defects, Atrial fibrillation and flutter, Aortic aneurysm, Alcoholic cardiomyopathy, Other cardiomyopathy, Endocarditis, Hypertensive heart disease, Ischemic heart disease, Other cardiovascular and circulatory diseases, Pulmonary Arterial Hypertension, Lower extremity peripheral arterial disease, Intracerebral hemorrhage, Ischemic stroke, Subarachnoid hemorrhage, Non-rheumatic valvular heart disease, Diabetes mellitus type 1, Diabetes mellitus type 2, Appendicitis, Gallbladder and biliary diseases, Gastritis and duodenitis, Inguinal, femoral, and abdominal hernia, Ulcerative colitis, Crohn's disease, Paralytic ileus and intestinal obstruction, Other digestive diseases, Pancreatitis, Peptic ulcer disease, Vascular intestinal disorders, Acute glomerulonephritis, Endometriosis, Uterine fibroids, Other gynecological diseases, Genital prolapse, G6PD deficiency, Other hemoglobinopathies and hemolytic anemias, Sickle cell disorders, Thalassemias, Other unspecified infectious diseases, Non-venomous animal contact, Venomous animal contact, Exposure to forces of nature, Falls, Fire, heat, and hot substances, Pulmonary aspiration and foreign body in airway, Foreign body in other body part, Physical violence by firearm, Physical violence by sharp object, Physical violence by other means, Unintentional firearm injuries, Other exposure to mechanical forces, Adverse effects of medical treatment, Environmental heat and cold exposure, Other unintentional injuries (internal), Self-harm by firearm, Self-harm by other specified means, Road injuries, Police conflict and executions, Conflict and terrorism, Other intestinal infectious diseases, Ectopic pregnancy, Maternal abortion and miscarriage, Maternal hemorrhage, Maternal hypertensive disorders, Late maternal deaths, Maternal obstructed labor and uterine rupture, Other direct maternal disorders, Alcohol use disorders, Amphetamine use disorders, Cocaine use disorders, Opioid use disorders, Other drug use disorders, Anorexia nervosa, Other musculoskeletal disorders, Rheumatoid arthritis, Myelodysplastic, myeloproliferative, and other hematopoietic neoplasms, Other benign and in situ neoplasms, Bladder cancer, Malignant neoplasm of bone and articular cartilage, Brain and central nervous system cancer, Breast cancer, Cervical cancer, Colon and rectum cancer, Esophageal cancer, Other eye cancers, Retinoblastoma, Gallbladder and biliary tract cancer, Hodgkin lymphoma, Kidney cancer, Larynx cancer, Leukemia, Liver cancer, Tracheal, bronchus, and lung cancer, Non-Hodgkin lymphoma, Malignant skin melanoma, Mesothelioma, Lip and oral cavity cancer, Multiple myeloma, Nasopharynx cancer, Neuroblastoma and other peripheral nervous cell tumors, Non-melanoma skin cancer, Other malignant neoplasms, Other pharynx cancer, Ovarian cancer, Pancreatic cancer, Prostate cancer, Stomach cancer, Testicular cancer, Thyroid cancer, Soft tissue and other extraosseous sarcomas, Uterine cancer, Neonatal encephalopathy due to birth asphyxia and trauma, Hemolytic disease and other neonatal jaundice, Other neonatal disorders, Neonatal preterm birth, Idiopathic epilepsy, Multiple sclerosis, Motor neuron disease, Other neurological disorders, Parkinson's disease, Other nutritional deficiencies, Protein-energy malnutrition, Other urinary diseases, Urolithiasis
	Hepatitis	Cirrhosis and other chronic liver diseases, Digestive congenital anomalies, Gallbladder and biliary diseases, Gastritis and duodenitis, Ulcerative colitis, Crohn's disease, Paralytic ileus and intestinal obstruction, Other digestive diseases, Pancreatitis, Peptic ulcer disease, G6PD deficiency, Other hemoglobinopathies and hemolytic anemias, Sickle cell disorders, Thalassemias, Acute hepatitis A, Acute hepatitis B, Acute hepatitis C, Acute hepatitis E, Other unspecified infectious diseases, Foreign body in other body part, Adverse effects of medical treatment,

		Other intestinal infectious diseases, Ectopic pregnancy, Maternal abortion and miscarriage, Maternal hypertensive disorders, Late maternal deaths, Other direct maternal disorders, Myelodysplastic, myeloproliferative, and other hematopoietic neoplasms, Other benign and in situ neoplasms, Bladder cancer, Malignant neoplasm of bone and articular cartilage, Brain and central nervous system cancer, Breast cancer, Cervical cancer, Colon and rectum cancer, Esophageal cancer, Other eye cancers, Retinoblastoma, Gallbladder and biliary tract cancer, Hodgkin lymphoma, Kidney cancer, Larynx cancer, Leukemia, Liver cancer, Tracheal, bronchus, and lung cancer, Non-Hodgkin lymphoma, Malignant skin melanoma, Mesothelioma, Lip and oral cavity cancer, Multiple myeloma, Nasopharynx cancer, Neuroblastoma and other peripheral nervous cell tumors, Non-melanoma skin cancer, Other malignant neoplasms, Other pharynx cancer, Ovarian cancer, Pancreatic cancer, Prostate cancer, Stomach cancer, Testicular cancer, Thyroid cancer, Soft tissue and other extraosseous sarcomas, Uterine cancer, Other neonatal disorders, Other nutritional deficiencies, Protein-energy malnutrition
Diarrhoea	Diarrhoea	Chronic kidney disease due to diabetes mellitus type 1, Chronic kidney disease due to diabetes mellitus type 2, Chronic kidney disease due to glomerulonephritis, Chronic kidney disease due to hypertension, Chronic kidney disease due to other and unspecified causes, Digestive congenital anomalies, Diabetes mellitus type 1, Diabetes mellitus type 2, Diarrheal diseases, Gastritis and duodenitis, Ulcerative colitis, Crohn's disease, Other digestive diseases, Peptic ulcer disease, Acute glomerulonephritis, Endometriosis, Uterine fibroids, Other gynecological diseases, Genital prolapse, Other unspecified infectious diseases, Exposure to forces of nature, Adverse effects of medical treatment, Other intestinal infectious diseases, Ectopic pregnancy, Maternal abortion and miscarriage, Late maternal deaths, Alcohol use disorders, Amphetamine use disorders, Cocaine use disorders, Opioid use disorders, Other drug use disorders, Anorexia nervosa, Other musculoskeletal disorders, Rheumatoid arthritis, Myelodysplastic, myeloproliferative, and other hematopoietic neoplasms, Other benign and in situ neoplasms, Bladder cancer, Malignant neoplasm of bone and articular cartilage, Brain and central nervous system cancer, Breast cancer, Cervical cancer, Colon and rectum cancer, Esophageal cancer, Other eye cancers, Retinoblastoma, Gallbladder and biliary tract cancer, Hodgkin lymphoma, Kidney cancer, Larynx cancer, Leukemia, Liver cancer, Tracheal, bronchus, and lung cancer, Non-Hodgkin lymphoma, Malignant skin melanoma, Mesothelioma, Lip and oral cavity cancer, Multiple myeloma, Nasopharynx cancer, Neuroblastoma and other peripheral nervous cell tumors, Non-melanoma skin cancer, Other malignant neoplasms, Other pharynx cancer, Ovarian cancer, Pancreatic cancer, Prostate cancer, Stomach cancer, Testicular cancer, Thyroid cancer, Soft tissue and other extraosseous sarcomas, Uterine cancer, Neonatal encephalopathy due to birth asphyxia and trauma, Hemolytic disease and other neonatal jaundice, Other neonatal disorders, Neonatal preterm birth, Idiopathic epilepsy, Multiple sclerosis, Motor neuron disease, Other neurological disorders, Parkinson's disease, Other nutritional deficiencies, Protein-energy malnutrition, Other urinary diseases, Urolithiasis
Urinary tract infections and pyelonephritis	Urinary tract infections and pyelonephritis	Cirrhosis and other chronic liver diseases, Chronic kidney disease due to diabetes mellitus type 1, Chronic kidney disease due to diabetes mellitus type 2, Chronic kidney disease due to glomerulonephritis, Chronic kidney disease due to hypertension, Chronic kidney disease due to other and unspecified causes, Other chromosomal abnormalities, Orofacial clefts, Digestive congenital anomalies, Down syndrome, Congenital heart anomalies, Congenital musculoskeletal and limb anomalies, Neural tube defects, Other congenital birth defects, Urogenital congenital anomalies, Atrial fibrillation and flutter, Aortic aneurysm, Alcoholic cardiomyopathy, Other cardiomyopathy, Hypertensive heart disease, Ischemic heart disease, Other cardiovascular and circulatory diseases, Pulmonary Arterial Hypertension, Lower extremity peripheral arterial disease, Intracerebral hemorrhage, Ischemic stroke, Subarachnoid hemorrhage, Non-rheumatic valvular heart disease, Diabetes mellitus type 1, Diabetes mellitus type 2, Gallbladder and biliary diseases, Gastritis and duodenitis, Inguinal, femoral, and abdominal hernia, Ulcerative colitis, Crohn's disease, Paralytic ileus and intestinal obstruction, Other digestive diseases, Pancreatitis, Peptic ulcer disease, Vascular intestinal disorders, Other endocrine, metabolic, blood, and immune disorders, Thyroid diseases, Acute glomerulonephritis, Endometriosis, Uterine fibroids, Other gynecological diseases, Genital prolapse, G6PD deficiency, Other hemoglobinopathies and hemolytic anemias, Sickle cell disorders, Thalassemias, Other unspecified infectious diseases, Non-venomous animal contact, Venomous animal contact, Exposure to forces of nature, Drowning, Electrocution, Falls, Fire, heat, and hot substances, Pulmonary aspiration and foreign body in airway, Foreign body in other body part, Physical violence by firearm, Physical violence by sharp object, Physical violence by other means, Unintentional firearm injuries, Other exposure to mechanical forces, Adverse effects of medical treatment, Environmental heat and cold exposure, Other unintentional injuries (internal), Poisoning by carbon monoxide, Poisoning by other means, Self-harm by firearm, Self-harm by other specified means, Road injuries, Police conflict and executions, Conflict and terrorism, Other intestinal infectious diseases, Ectopic pregnancy, Maternal abortion and miscarriage, Maternal hemorrhage, Maternal hypertensive disorders, Late maternal deaths, Maternal obstructed labor and uterine rupture, Other direct maternal disorders, Alcohol use disorders, Amphetamine use disorders, Cocaine use disorders, Opioid use disorders, Other drug use disorders, Anorexia nervosa, Other musculoskeletal disorders, Rheumatoid arthritis, Myelodysplastic, myeloproliferative, and

		other hematopoietic neoplasms, Other benign and in situ neoplasms, Bladder cancer, Malignant neoplasm of bone and articular cartilage, Brain and central nervous system cancer, Breast cancer, Cervical cancer, Colon and rectum cancer, Esophageal cancer, Other eye cancers, Retinoblastoma, Gallbladder and biliary tract cancer, Hodgkin lymphoma, Kidney cancer, Larynx cancer, Leukemia, Liver cancer, Tracheal, bronchus, and lung cancer, Non-Hodgkin lymphoma, Malignant skin melanoma, Mesothelioma, Lip and oral cavity cancer, Multiple myeloma, Nasopharynx cancer, Neuroblastoma and other peripheral nervous cell tumors, Non-melanoma skin cancer, Other malignant neoplasms, Other pharynx cancer, Ovarian cancer, Pancreatic cancer, Prostate cancer, Stomach cancer, Testicular cancer, Thyroid cancer, Soft tissue and other extraosseous sarcomas, Uterine cancer, Neonatal encephalopathy due to birth asphyxia and trauma, Hemolytic disease and other neonatal jaundice, Other neonatal disorders, Neonatal preterm birth, Idiopathic epilepsy, Multiple sclerosis, Motor neuron disease, Other neurological disorders, Parkinson's disease, Other nutritional deficiencies, Protein-energy malnutrition, Otitis media, Asthma, Chronic obstructive pulmonary disease, Interstitial lung disease and pulmonary sarcoidosis, Other chronic respiratory diseases, Pneumoconiosis, Decubitus ulcer, Other skin and subcutaneous diseases, Upper respiratory infections, Other urinary diseases, Urolithiasis
	Genital infections	Chronic kidney disease due to glomerulonephritis, Chronic kidney disease due to hypertension, Chronic kidney disease due to other and unspecified causes, Other chromosomal abnormalities, Other congenital birth defects, Urogenital congenital anomalies, Endometriosis, Uterine fibroids, Other gynecological diseases, Genital prolapse, Other unspecified infectious diseases, Electrocution, Foreign body in other body part, Physical violence by sharp object, Physical violence by other means, Unintentional firearm injuries, Other exposure to mechanical forces, Adverse effects of medical treatment, Environmental heat and cold exposure, Other unintentional injuries (internal), Self-harm by firearm, Self-harm by other specified means, Road injuries, Police conflict and executions, Conflict and terrorism, Ectopic pregnancy, Maternal abortion and miscarriage, Maternal hemorrhage, Maternal hypertensive disorders, Late maternal deaths, Other direct maternal disorders, Cervical cancer, Ovarian cancer, Prostate cancer, Testicular cancer, Uterine cancer, Other nutritional deficiencies, Other urinary diseases, Urolithiasis
Infections of bones, joints, and related organs	Infections of bones, joints, and related organs	Chronic kidney disease due to diabetes mellitus type 1, Chronic kidney disease due to diabetes mellitus type 2, Chronic kidney disease due to glomerulonephritis, Chronic kidney disease due to hypertension, Chronic kidney disease due to other and unspecified causes, Other chromosomal abnormalities, Orofacial clefts, Congenital musculoskeletal and limb anomalies, Neural tube defects, Other congenital birth defects, Atrial fibrillation and flutter, Aortic aneurysm, Alcoholic cardiomyopathy, Other cardiomyopathy, Hypertensive heart disease, Ischemic heart disease, Other cardiovascular and circulatory diseases, Pulmonary Arterial Hypertension, Lower extremity peripheral arterial disease, Intracerebral hemorrhage, Ischemic stroke, Subarachnoid hemorrhage, Diabetes mellitus type 1, Diabetes mellitus type 2, Acute glomerulonephritis, G6PD deficiency, Other hemoglobinopathies and hemolytic anemias, Sickle cell disorders, Thalassemias, Other unspecified infectious diseases, Non-venomous animal contact, Venomous animal contact, Exposure to forces of nature, Electrocution, Falls, Fire, heat, and hot substances, Foreign body in other body part, Physical violence by firearm, Physical violence by sharp object, Physical violence by other means, Unintentional firearm injuries, Other exposure to mechanical forces, Adverse effects of medical treatment, Environmental heat and cold exposure, Other unintentional injuries (internal), Self-harm by firearm, Self-harm by other specified means, Road injuries, Police conflict and executions, Conflict and terrorism, Other intestinal infectious diseases, Late maternal deaths, Alcohol use disorders, Amphetamine use disorders, Cocaine use disorders, Opioid use disorders, Other drug use disorders, Other musculoskeletal disorders, Rheumatoid arthritis, Myelodysplastic, myeloproliferative, and other hematopoietic neoplasms, Other benign and in situ neoplasms, Bladder cancer, Malignant neoplasm of bone and articular cartilage, Brain and central nervous system cancer, Breast cancer, Cervical cancer, Colon and rectum cancer, Esophageal cancer, Other eye cancers, Retinoblastoma, Gallbladder and biliary tract cancer, Hodgkin lymphoma, Kidney cancer, Larynx cancer, Leukemia, Liver cancer, Tracheal, bronchus, and lung cancer, Non-Hodgkin lymphoma, Malignant skin melanoma, Mesothelioma, Lip and oral cavity cancer, Multiple myeloma, Nasopharynx cancer, Neuroblastoma and other peripheral nervous cell tumors, Non-melanoma skin cancer, Other malignant neoplasms, Other pharynx cancer, Ovarian cancer, Pancreatic cancer, Prostate cancer, Stomach cancer, Testicular cancer, Thyroid cancer, Soft tissue and other extraosseous sarcomas, Uterine cancer, Neonatal encephalopathy due to birth asphyxia and trauma, Hemolytic disease and other neonatal jaundice, Other neonatal disorders, Neonatal preterm birth, Other nutritional deficiencies, Protein-energy malnutrition, Otitis media, Decubitus ulcer, Other skin and subcutaneous diseases
Infections of the skin and	Infections of the skin and	Chronic kidney disease due to diabetes mellitus type 1, Chronic kidney disease due to diabetes mellitus type 2, Chronic kidney disease due to glomerulonephritis, Chronic kidney disease due to hypertension, Chronic kidney disease due to other and unspecified causes, Orofacial clefts, Neural tube defects, Atrial fibrillation and flutter, Aortic aneurysm, Alcoholic cardiomyopathy, Other cardiomyopathy, Hypertensive heart

subcutaneous systems	subcutaneous systems	disease, Ischemic heart disease, Other cardiovascular and circulatory diseases, Pulmonary Arterial Hypertension, Lower extremity peripheral arterial disease, Intracerebral hemorrhage, Ischemic stroke, Subarachnoid hemorrhage, Diabetes mellitus type 1, Diabetes mellitus type 2, Gastritis and duodenitis, Peptic ulcer disease, Acute glomerulonephritis, Endometriosis, Uterine fibroids, Other gynecological diseases, Genital prolapse, G6PD deficiency, Other hemoglobinopathies and hemolytic anemias, Sickle cell disorders, Thalassemias, Other unspecified infectious diseases, Non-venomous animal contact, Venomous animal contact, Exposure to forces of nature, Electrocution, Falls, Fire, heat, and hot substances, Foreign body in other body part, Physical violence by firearm, Physical violence by sharp object, Physical violence by other means, Unintentional firearm injuries, Other exposure to mechanical forces, Adverse effects of medical treatment, Environmental heat and cold exposure, Other unintentional injuries (internal), Self-harm by firearm, Self-harm by other specified means, Road injuries, Police conflict and executions, Conflict and terrorism, Ectopic pregnancy, Maternal abortion and miscarriage, Late maternal deaths, Alcohol use disorders, Amphetamine use disorders, Cocaine use disorders, Opioid use disorders, Other drug use disorders, Anorexia nervosa, Other musculoskeletal disorders, Rheumatoid arthritis, Myelodysplastic, myeloproliferative, and other hematopoietic neoplasms, Other benign and in situ neoplasms, Bladder cancer, Malignant neoplasm of bone and articular cartilage, Brain and central nervous system cancer, Breast cancer, Cervical cancer, Colon and rectum cancer, Esophageal cancer, Other eye cancers, Retinoblastoma, Gallbladder and biliary tract cancer, Hodgkin lymphoma, Kidney cancer, Larynx cancer, Leukemia, Liver cancer, Tracheal, bronchus, and lung cancer, Non-Hodgkin lymphoma, Malignant skin melanoma, Mesothelioma, Lip and oral cavity cancer, Multiple myeloma, Nasopharynx cancer, Neuroblastoma and other peripheral nervous cell tumors, Non-melanoma skin cancer, Other malignant neoplasms, Other pharynx cancer, Ovarian cancer, Pancreatic cancer, Prostate cancer, Stomach cancer, Testicular cancer, Thyroid cancer, Soft tissue and other extraosseous sarcomas, Uterine cancer, Neonatal encephalopathy due to birth asphyxia and trauma, Hemolytic disease and other neonatal jaundice, Other neonatal disorders, Neonatal preterm birth, Idiopathic epilepsy, Multiple sclerosis, Motor neuron disease, Other neurological disorders, Parkinson's disease, Other nutritional deficiencies, Protein-energy malnutrition, Asthma, Chronic obstructive pulmonary disease, Interstitial lung disease and pulmonary sarcoidosis, Other chronic respiratory diseases, Pyoderma, Cellulitis, Decubitus ulcer, Other skin and subcutaneous diseases, Other urinary diseases, Urolithiasis
Tuberculosis	Tuberculosis	Other unspecified infectious diseases, Other intestinal infectious diseases, Late maternal deaths, Anorexia nervosa, Myelodysplastic, myeloproliferative, and other hematopoietic neoplasms, Other benign and in situ neoplasms, Bladder cancer, Malignant neoplasm of bone and articular cartilage, Brain and central nervous system cancer, Breast cancer, Cervical cancer, Colon and rectum cancer, Esophageal cancer, Other eye cancers, Retinoblastoma, Gallbladder and biliary tract cancer, Hodgkin lymphoma, Kidney cancer, Larynx cancer, Leukemia, Liver cancer, Tracheal, bronchus, and lung cancer, Non-Hodgkin lymphoma, Malignant skin melanoma, Mesothelioma, Lip and oral cavity cancer, Multiple myeloma, Nasopharynx cancer, Neuroblastoma and other peripheral nervous cell tumors, Non-melanoma skin cancer, Other malignant neoplasms, Other pharynx cancer, Ovarian cancer, Pancreatic cancer, Prostate cancer, Stomach cancer, Testicular cancer, Thyroid cancer, Soft tissue and other extraosseous sarcomas, Uterine cancer, Other neonatal disorders, Idiopathic epilepsy, Multiple sclerosis, Motor neuron disease, Other neurological disorders, Parkinson's disease, Other nutritional deficiencies, Protein-energy malnutrition, Tuberculosis
Typhoid, paratyphoid, and invasive non-typhoidal Salmonella	Typhoid, paratyphoid, and invasive non-typhoidal Salmonella	Other unspecified infectious diseases, Invasive Non-typhoidal Salmonella (iNTS), Paratyphoid fever, Typhoid fever
	Other parasitic infections	Chronic kidney disease due to diabetes mellitus type 1, Chronic kidney disease due to diabetes mellitus type 2, Chronic kidney disease due to glomerulonephritis, Chronic kidney disease due to hypertension, Chronic kidney disease due to other and unspecified causes, Atrial fibrillation and flutter, Aortic aneurysm, Alcoholic cardiomyopathy, Other cardiomyopathy, Hypertensive heart disease, Ischemic heart disease, Other cardiovascular and circulatory diseases, Pulmonary Arterial Hypertension, Lower extremity peripheral arterial disease, Intracerebral hemorrhage, Ischemic stroke, Subarachnoid hemorrhage, Diabetes mellitus type 1, Diabetes mellitus type 2, Gallbladder and biliary diseases, Gastritis and duodenitis, Inguinal, femoral, and abdominal hernia, Ulcerative colitis, Crohn's disease, Paralytic ileus and intestinal obstruction, Other digestive diseases, Pancreatitis, Peptic ulcer disease, Vascular intestinal disorders, Acute glomerulonephritis, Endometriosis, Uterine

		fibroids, Other gynecological diseases, Genital prolapse, G6PD deficiency, Other hemoglobinopathies and hemolytic anemias, Sickle cell disorders, Thalassemias, Other unspecified infectious diseases, Non-venomous animal contact, Venomous animal contact, Exposure to forces of nature, Drowning, Falls, Fire, heat, and hot substances, Pulmonary aspiration and foreign body in airway, Foreign body in other body part, Physical violence by firearm, Physical violence by sharp object, Physical violence by other means, Unintentional firearm injuries, Other exposure to mechanical forces, Adverse effects of medical treatment, Environmental heat and cold exposure, Other unintentional injuries (internal), Self-harm by firearm, Self-harm by other specified means, Road injuries, Police conflict and executions, Conflict and terrorism, Other intestinal infectious diseases, Ectopic pregnancy, Maternal abortion and miscarriage, Maternal hemorrhage, Maternal hypertensive disorders, Late maternal deaths, Maternal obstructed labor and uterine rupture, Other direct maternal disorders, Alcohol use disorders, Amphetamine use disorders, Cocaine use disorders, Opioid use disorders, Other drug use disorders, Other musculoskeletal disorders, Rheumatoid arthritis, Myelodysplastic, myeloproliferative, and other hematopoietic neoplasms, Other benign and in situ neoplasms, Bladder cancer, Malignant neoplasm of bone and articular cartilage, Brain and central nervous system cancer, Breast cancer, Cervical cancer, Colon and rectum cancer, Esophageal cancer, Other eye cancers, Retinoblastoma, Gallbladder and biliary tract cancer, Hodgkin lymphoma, Kidney cancer, Larynx cancer, Leukemia, Liver cancer, Tracheal, bronchus, and lung cancer, Non-Hodgkin lymphoma, Malignant skin melanoma, Mesothelioma, Lip and oral cavity cancer, Multiple myeloma, Nasopharynx cancer, Neuroblastoma and other peripheral nervous cell tumors, Non-melanoma skin cancer, Other malignant neoplasms, Other pharynx cancer, Ovarian cancer, Pancreatic cancer, Prostate cancer, Stomach cancer, Testicular cancer, Thyroid cancer, Soft tissue and other extraosseous sarcomas, Uterine cancer, Neonatal encephalopathy due to birth asphyxia and trauma, Hemolytic disease and other neonatal jaundice, Other neonatal disorders, Neonatal preterm birth, Other neurological disorders, Other nutritional deficiencies, Protein-energy malnutrition, Otitis media, Asthma, Chronic obstructive pulmonary disease, Interstitial lung disease and pulmonary sarcoidosis, Other chronic respiratory diseases, Pneumoconiosis, Other skin and subcutaneous diseases, Other urinary diseases, Urolithiasis
	Other unspecified site infections	Cirrhosis and other chronic liver diseases, Chronic kidney disease due to diabetes mellitus type 1, Chronic kidney disease due to diabetes mellitus type 2, Chronic kidney disease due to glomerulonephritis, Chronic kidney disease due to hypertension, Chronic kidney disease due to other and unspecified causes, Other chromosomal abnormalities, Orofacial clefts, Digestive congenital anomalies, Down syndrome, Congenital heart anomalies, Congenital musculoskeletal and limb anomalies, Neural tube defects, Other congenital birth defects, Urogenital congenital anomalies, Atrial fibrillation and flutter, Aortic aneurysm, Alcoholic cardiomyopathy, Other cardiomyopathy, Hypertensive heart disease, Ischemic heart disease, Other cardiovascular and circulatory diseases, Pulmonary Arterial Hypertension, Lower extremity peripheral arterial disease, Intracerebral hemorrhage, Ischemic stroke, Subarachnoid hemorrhage, Non-rheumatic valvular heart disease, Diabetes mellitus type 1, Diabetes mellitus type 2, Gallbladder and biliary diseases, Gastritis and duodenitis, Inguinal, femoral, and abdominal hernia, Ulcerative colitis, Crohn's disease, Paralytic ileus and intestinal obstruction, Other digestive diseases, Pancreatitis, Peptic ulcer disease, Vascular intestinal disorders, Other endocrine, metabolic, blood, and immune disorders, Thyroid diseases, Acute glomerulonephritis, Endometriosis, Uterine fibroids, Other gynecological diseases, Genital prolapse, G6PD deficiency, Other hemoglobinopathies and hemolytic anemias, Sickle cell disorders, Thalassemias, Other unspecified infectious diseases, Non-venomous animal contact, Venomous animal contact, Exposure to forces of nature, Drowning, Electrocution, Falls, Fire, heat, and hot substances, Pulmonary aspiration and foreign body in airway, Foreign body in other body part, Physical violence by firearm, Physical violence by sharp object, Physical violence by other means, Unintentional firearm injuries, Other exposure to mechanical forces, Adverse effects of medical treatment, Environmental heat and cold exposure, Other unintentional injuries (internal), Poisoning by carbon monoxide, Poisoning by other means, Self-harm by firearm, Self-harm by other specified means, Road injuries, Police conflict and executions, Conflict and terrorism, Other intestinal infectious diseases, Ectopic pregnancy, Maternal abortion and miscarriage, Maternal hemorrhage, Maternal hypertensive disorders, Late maternal deaths, Maternal obstructed labor and uterine rupture, Other direct maternal disorders, Alcohol use disorders, Amphetamine use disorders, Cocaine use disorders, Opioid use disorders, Other drug use disorders, Anorexia nervosa, Other musculoskeletal disorders, Rheumatoid arthritis, Myelodysplastic, myeloproliferative, and other hematopoietic neoplasms, Other benign and in situ neoplasms, Bladder cancer, Malignant neoplasm of bone and articular cartilage, Brain and central nervous system cancer, Breast cancer, Cervical cancer, Colon and rectum cancer, Esophageal cancer, Other eye cancers, Retinoblastoma, Gallbladder and biliary tract cancer, Hodgkin lymphoma, Kidney cancer, Larynx cancer, Leukemia, Liver cancer, Tracheal, bronchus, and lung cancer, Non-Hodgkin lymphoma, Malignant skin melanoma, Mesothelioma, Lip and oral cavity cancer, Multiple myeloma, Nasopharynx cancer, Neuroblastoma and other peripheral nervous cell tumors, Non-melanoma skin cancer, Other malignant neoplasms, Other pharynx cancer, Ovarian cancer, Pancreatic cancer, Prostate cancer, Stomach cancer, Testicular cancer, Thyroid cancer, Soft tissue and other extraosseous sarcomas, Uterine cancer, Neonatal encephalopathy due to birth asphyxia and trauma, Hemolytic disease and other neonatal

		jaundice, Other neonatal disorders, Neonatal preterm birth, Idiopathic epilepsy, Multiple sclerosis, Motor neuron disease, Other neurological disorders, Parkinson's disease, Other nutritional deficiencies, Protein-energy malnutrition, Otitis media, Asthma, Chronic obstructive pulmonary disease, Interstitial lung disease and pulmonary sarcoidosis, Other chronic respiratory diseases, Pneumoconiosis, Decubitus ulcer, Other skin and subcutaneous diseases, Upper respiratory infections, Other urinary diseases, Urolithiasis

Table S2: Case fatality ratio modelling framework by pathogen and syndrome

Pathogen	Bone +	BSI	Endocarditis	Intra-abdominal	LRI community-acquired	LRI hospital-acquired	Meningitis	Skin	UTI community-acquired	UTI hospital-acquired
Acinetobacter baumannii	**Family**	**Individual**	--	**Individual**	**Intercept**	**Intercept**	**Family**	**Intercept**	**Intercept**	**Intercept**
Acinetobacter others	--	**Family**	--	**Family**	**Family**	**Family**	--	--	**Family**	**Family**
Actinomyces spp.	--	--	--	**Individual**	**Individual**	**Individual**	--	**Individual**	--	--
Adenovirus	--	--	--	--	**Individual**	**Individual**	--	--	--	--
Aeromonas spp.	--	--	--	**Family**	--	--	--	**Family**	**Family**	**Family**
Aspergillus spp.	--	--	--	--	**Intercept**	**Intercept**	--	--	--	--
Burkholderia spp.	--	**Intercept**	--	**Family**	**Family**	**Family**	--	--	**Family**	**Family**
Candida spp.	--	**Individual**	**Family**	**Individual**	--	--	**Family**	--	**Intercept**	**Intercept**
Chlamydia spp.	--	--	--	**Intercept**	**Intercept**	**Intercept**	--	--	--	--
Citrobacter spp.	--	**Individual**	--	**Intercept**	**Individual**	**Intercept**	--	**Individual**	**Intercept**	**Intercept**
Clostridium others	--	--	--	--	--	--	--	**Intercept**	--	--
coagulase negative *Staphylococcus*	--	--	--	--	--	--	**Family**	--	**Family**	**Family**
Cytomegalovirus	--	--	--	--	**Individual**	**Individual**	--	--	--	--
Entamoeba histolytica	--	--	--	**Individual**	--	--	**Family**	--	--	--
Enterobacter spp.	**Intercept**	**Intercept**	--	**Individual**	**Intercept**	**Intercept**	--	**Intercept**	**Intercept**	**Intercept**
Enterococcus faecalis	**Family**	**Individual**	--	**Intercept**	--	**Individual**	--	**Family**	**Intercept**	**Intercept**
Enterococcus faecium	**Family**	**Intercept**	--	**Individual**	**Individual**	**Individual**	--	**Individual**	**Intercept**	**Intercept**
Enterococcus others	--	--	--	**Family**	--	--	--	--	**Family**	**Family**
Epstein barr virus	--	--	--	--	--	--	--	--	--	--
Escherichia coli	**Intercept**	**Individual**	**Intercept**	**Individual**	**Individual**	**Individual**	**Individual**	**Intercept**	**Intercept**	**Intercept**
Fungi others	--	**Individual**	--	--	**Intercept**	**Individual**	**Individual**	--	**All pathogen**	**All pathogen**
Gram negative others	**Family**	**Intercept**	--	**Intercept**	**Individual**	**Individual**	--	**Intercept**	**Intercept**	**Individual**
Gram negative unspecified	--	**Intercept**	--	--	**Individual**	**Individual**	--	--	--	--

Gram positive others	**Family**	**Individual**	--	--	--	--	--	**Intercept**	--	--
Haemophilus influenzae	--	**Individual**	--	--	**Intercept**	**Individual**	**Intercept**	--	--	--
Histoplasma spp.	--	**Individual**	--	--	**Individual**	**Individual**	--	--	--	--
Influenza virus	--	--	--	--	**Intercept**	**Intercept**	--	--	--	--
Klebsiella others	**Family**	**Intercept**	--	**Intercept**	**Individual**	**Individual**	--	**Intercept**	**Intercept**	**Intercept**
Klebsiella pneumoniae	**Intercept**	**Intercept**	**Family**	**Individual**	**Individual**	**Individual**	**Intercept**	**Intercept**	**Intercept**	**Intercept**
Legionella spp.	--	--	--	--	**Individual**	**Individual**	--	--	--	--
Leptospira spp.	--	**Individual**	--	--	--	--	--	--	--	--
Listeria spp.	--	**Individual**	--	--	--	--	**Individual**	--	--	--
Morganella spp.	**Family**	**Intercept**	--	**Intercept**	**Individual**	**Individual**	--	**Intercept**	**Intercept**	**Intercept**
Mumps	--	--	--	--	--	--	--	--	--	--
Mycobacterium others	--	**Individual**	--	--	**Intercept**	**Intercept**	--	**Individual**	--	--
Mycoplasma spp.	--	--	--	--	**Intercept**	**Intercept**	--	--	--	--
Neisseria meningitidis	--	**Individual**	--	--	--	--	**Intercept**	--	--	--
Non polio enteroviruses	--	--	--	--	--	--	**Intercept**	**Intercept**	--	--
Proteus spp.	**Intercept**	**Intercept**	--	**Individual**	**Individual**	**Intercept**	--	**Intercept**	**Intercept**	**Intercept**
Pseudomonas aeruginosa	**Intercept**	**Individual**	**Intercept**	**Individual**	**Individual**	**Individual**	**Intercept**	**Intercept**	**Intercept**	**Intercept**
Pseudomonas others	--	--	--	**Family**	--	--	--	--	**Family**	**Family**
Respiratory syncytial virus	--	--	--	--	**Intercept**	**Intercept**	--	--	--	--
Serratia spp.	**Family**	**Individual**	--	**Intercept**	**Intercept**	**Intercept**	--	**Intercept**	**Intercept**	**Intercept**
Staphylococcus aureus	**Intercept**	**Individual**	**Intercept**	**Intercept**	**Individual**	**Individual**	**Individual**	**Intercept**	**Intercept**	**Intercept**
Streptococcus group a	**Family**	**Family**	**Intercept**	--	**Family**	**Family**	--	**Individual**	--	--
Streptococcus group b	**Family**	**Family**	--	--	--	--	**Intercept**	**Family**	**Intercept**	**Family**
Streptococcus others	**Family**	**Family**	--	**Intercept**	--	--	**Family**	**Family**	**Family**	**Family**
Streptococcus pneumoniae	**Intercept**	**Individual**	--	**Intercept**	**Individual**	**Individual**	**Individual**	**Intercept**	**Intercept**	**Family**
toxoplasma spp.	--	--	--	--	--	--	--	--	--	--

Virus others	--	**Individual**	--	--	--	**Individual**	**Intercept**	**Intercept**	--	--
Syndrome wide	**All pathogen**	**All pathogen**	**All pathogen**	**All pathogen**	**All pathogen**	**All pathogen**	**All pathogen**	**All pathogen**	**All pathogen**	**All pathogen**

Bone+ = Infections of bones, joints, and related organs. BSI = Bloodstream infections. Endocarditis = Endocarditis, myocarditis, and other infections. Intra-abdominal = Peritoneal and intra-abdominal infections. LRI = Lower respiratory infections and all related infections in the thorax. Meningitis = Meningitis and other bacterial central nervous system infections. Skin = Infections of the skin and subcutaneous systems. UTI = Urinary tract infections and pyelonephritis.

Table S3: Summary of pathogen distribution data

Pathogen	BSI	Bone+	Endocarditis	LRI	Meningitis	Peritonitis+	Skin	UTI	Pathogen Total
Acinetobacter baumannii	75,347	371		211,970	1,872	58,846	8,855	47,252	404,513
Acinetobacter others	11,646			35,138		7,343		7,352	61,480
Actinomyces spp.						995	2,046		3,041
Aeromonas spp.						7,301	1,017	737	9,055
Aspergillus spp.				48,237					48,237
Burkholderia spp.	7,484					1,797		2,101	11,381
Candida spp.	99,383		5,272		608	424		7,057	112,744
Chlamydia spp.				19,715		2,937			22,652
Citrobacter spp.	12,722			46,248		62,855	5,707	87,929	215,461
Clostridium others							28,281		28,281
Coagulase-negative *Staphylococcus*					1,328			9,000	10,328
Entamoeba histolytica						5,976			5,976
Enterobacter spp.	47,063	1,431		150,867		123,460	22,857	118,430	464,108
Enterococcus faecalis	267,398	926				1,674	5,665	47,548	323,212
Enterococcus faecium	170,838	594				4,926	12,454	40,272	229,083
Enterococcus others								658	658
Escherichia coli	2,956,318	3,717	718	315,484	4,455	657,782	85,413	2,041,580	6,065,466
Fungi others				22,214	612			413	23,239
Gram-negative others	82,030	1,276		170,442		39,663	5,751	39,269	338,431

Gram-negatives (ICD)*	521,217			96,100					617,318
Gram-positive others		724					4,829		5,553
Group A *Streptococcus*	21,098	2,566	1,177	14,881			545,617		585,339
Group B *Streptococcus*	14,541	2,237		3,867	5,813		3,606	33,598	63,662
Haemophilus influenzae	11,998			79,464	10,314				101,777
Influenza				159,677					159,677
Klebsiella others	32,576	362		147,724		89,518	5,813	111,165	387,158
Klebsiella pneumoniae	576,807	1,718	467	539,842	2,890	270,742	34,740	605,573	2,032,780
Listeria spp.					1,778				1,778
Legionella spp.				37,318					37,318
Morganella spp.	7,731	624		18,616		38,867	9,447	57,248	132,534
Mycobacterium others	21,826			35,419			2,047		59,292
Mycoplasma spp.				135,093					135,093
Neisseria meningitidis	18,292				25,989				44,281
non-polio Enteroviruses					12,373		62,244		74,617
Other pathogens	30,396	1,061	1,350	88,084	1,991	648	2,688	1,358	127,576
Proteus spp.	35,696	2,080		71,113		90,172	38,067	284,740	521,869
Pseudomonas aeruginosa	356,769	3,263	645	665,153	2,883	196,847	74,552	295,268	1,595,381
Pseudomonas others						5,154		4,411	9,566
Respiratory syncytial virus				170,803					170,803
Serratia spp.	40,305	677		125,867		35,848	11,103	39,500	253,300

Staphylococcus aureus	1,933,908	56,780	9,952	564,593	6,630	11,565	448,750	62,094	3,094,273
Streptococcus others	24,479	2,289			1,815	2,048	5,204	1,857	37,692
Streptococcus pneumoniae	578,534	1,215		559,413	60,843	1,737	1,964	1,743	1,205,449
Virus others	57,009				2,810		674,487		734,306
Syndrome Total	8,013,411	83,910	19,582	4,533,344	145,004	1,719,125	2,103,202	3,948,156	20,566,071

Table excludes modelled estimates used as input data (most notably fractions of *Streptococcus pneumoniae* in LRI estimated from vaccine efficacy studies). Coagulase-negative *staphylococcus* represents *Staphylococcus saprophyticus* in UTI and all coagulase-negative species in Meningitis. Gram-negatives encompassed in ICD BSI are: *Acinetobacter baumannii*, *Acinetobacter* others, *Citrobacter* spp., *Klebsiella pneumoniae*, *Klebsiella* others, *Morganella* spp., *Proteus* spp., or our “Gram-negative others” category. Gram-negatives encompassed in ICD LRI are: *Acinetobacter baumannii*, *Acinetobacter* others, *Citrobacter* spp., *Enterobacter* spp., *Klebsiella* others, *Morganella* spp., *Proteus* spp., *Serratia* spp. or our “Gram-negative others” category. BSI = Bloodstream infections. Bone+ = Infections of bones, joints, and related organs. LRI = Lower respiratory infections. Peritonitis+ = Peritoneal and intra-abdominal infections. Skin = Infections of the skin and subcutaneous systems. UTI = Urinary tract infections and pyelonephritis.

Table S4: Covariates used in the first stage of modelling of proportion of resistance for each pathogen–drug combination

Pathogen	Antibiotic class	Covariates
Acinetobacter baumannii	Aminoglycosides	Fraction of OOP Health Expenditure, Pigs (per capita), Skilled Birth Attendance (proportion), Total Fertility Rate, Intravenous drug use (proportion by age), Dentists per capita, PCV3 lagged five year coverage, COVID-free (proportion), Vaccine adjusted HbSAg serporevalence age standardized, Estimated rate of J01G aminoglycosides consumption in defined daily doses (DDDs) per 1000 population
Acinetobacter baumannii	Anti-pseudomonal penicillin/Beta-Lactamase inhibitors	Diabetes Age-Standardized Prevalence (proportion), Fraction of OOP Health Expenditure, Physicians per capita, Pigs (per capita), Healthcare access and quality index, HIV age-standardized Prevalence, ORS (oral rehydration), Outdoor Air Pollution (PM2.5), Skilled Birth Attendance (proportion)
Acinetobacter baumannii	Beta Lactam/Beta-lactamase inhibitors	Physicians per capita, Proportion of population involved in agricultural activities, ORS (oral rehydration), Outdoor Air Pollution (PM2.5), Pharmacists per capita, Population-weighted mean temperature, Age-standardized SEV for Unsafe sanitation, Rotavirus coverage, COVID-free (proportion), Intravenous drug use (proportion by age)
Acinetobacter baumannii	Carbapenems	Diabetes Age-Standardized Prevalence (proportion), Physicians per capita, Pigs (per capita), Proportion of population involved in agricultural activities, Hospital Beds (per 1000), HIV age-standardized Prevalence, ORS (oral rehydration), Pharmacists per capita, Population-weighted mean temperature
Acinetobacter baumannii	Fluoroquinolones	Diabetes Age-Standardized Prevalence (proportion), Pigs (per capita), Hospital Beds (per 1000), ORS (oral rehydration), Skilled Birth Attendance (proportion), Population-weighted mean temperature, Population Density (over 1000 ppl/sqkm, proportion), Smoking Prevalence, Intravenous drug use (proportion by age)
Acinetobacter baumannii	Fourth-generation cephalosporins	Diabetes Age-Standardized Prevalence (proportion), Fraction of OOP Health Expenditure, Pigs (per capita), Hospital Beds (per 1000), ORS (oral rehydration), Skilled Birth Attendance (proportion), Population-weighted mean temperature, Total Fertility Rate, Age-standardized SEV for Unsafe sanitation
Acinetobacter baumannii	Third-generation cephalosporins	Fraction of OOP Health Expenditure, Pigs (per capita), Hospital Beds (per 1000), ORS (oral rehydration), Skilled Birth Attendance (proportion), Population-weighted mean temperature, Total Fertility Rate, Age-standardized SEV for Unsafe sanitation, Dentists per capita
Citrobacter spp.	Aminoglycosides	Fraction of OOP Health Expenditure, Healthcare access and quality index, Hospital Beds (per 1000), Total Fertility Rate, Antenatal Care (4 visits) Coverage (proportion), Population Density (over 1000 ppl/sqkm, proportion), Diabetes Fasting Plasma Glucose (mmol/L), by age, Hepatitis C Seroprevalence (anti-HCV) age standardized, Hib3 Vaccine Coverage, COVID-free (proportion)
Citrobacter spp.	Anti-pseudomonal penicillin/Beta-Lactamase inhibitors	Physicians per capita, Hospital Beds (per 1000), ORS (oral rehydration), Antenatal Care (4 visits) Coverage (proportion), Population Density (over 1000 ppl/sqkm, proportion), Diabetes Fasting Plasma Glucose (mmol/L), by age, Vaccine adjusted HbSAg serporevalence age standardized, Hib3 Vaccine Coverage, COVID-free (proportion), Hib3 lagged five year coverage, COVID-free (proportion)
Citrobacter spp.	Carbapenems	Fraction of OOP Health Expenditure, Proportion of population involved in agricultural activities, Healthcare access and quality index, Hospital Beds (per 1000), ORS (oral rehydration), Outdoor Air Pollution (PM2.5), Skilled Birth Attendance (proportion), Antenatal Care (4 visits) Coverage (proportion), Rotavirus coverage, COVID-free (proportion)
Citrobacter spp.	Fluoroquinolones	Fraction of OOP Health Expenditure, Physicians per capita, Proportion of population involved in agricultural activities, Healthcare access and quality index, Outdoor Air Pollution (PM2.5), Pharmacists per capita, Age-standardized SEV for Unsafe sanitation, Maternal Education (years per capita), Tuberculosis prevalence (age-standardized)
Citrobacter spp.	Fourth-generation cephalosporins	Diabetes Age-Standardized Prevalence (proportion), Fraction of OOP Health Expenditure, Proportion of population involved in agricultural activities, Average latitude, Outdoor Air Pollution (PM2.5), Smoking Prevalence, Rotavirus coverage, COVID-free (proportion), Intravenous drug use (proportion by age), Hepatitis C Seroprevalence (anti-HCV) age standardized, PCV3 lagged five year coverage, COVID-free (proportion)

Citrobacter spp.	Third-generation cephalosporins	Physicians per capita, Average latitude, Pharmacists per capita, Skilled Birth Attendance (proportion), Total Fertility Rate, Intravenous drug use (proportion by age), Diabetes Fasting Plasma Glucose (mmol/L), by age, Hib3 lagged five year coverage, COVID-free (proportion), Estimated rate of antibiotic consumption in defined daily doses (DDDs) per 1000 population
Enterobacter spp.	Aminoglycosides	Fraction of OOP Health Expenditure, Pigs (per capita), Average latitude, Healthcare access and quality index, Hospital Beds (per 1000), HIV age-standardized Prevalence, Pharmacists per capita, PCV3 lagged five year coverage, COVID-free (proportion), Hib3 Vaccine Coverage, COVID-free (proportion)
Enterobacter spp.	Anti-pseudomonal penicillin/Beta-Lactamase inhibitors	Diabetes Age-Standardized Prevalence (proportion), Fraction of OOP Health Expenditure, Outdoor Air Pollution (PM2.5), Pharmacists per capita, Dentists per capita, PCV3 lagged five year coverage, COVID-free (proportion), Hib3 Vaccine Coverage, COVID-free (proportion), Estimated rate of J01C penicillins consumption in defined daily doses (DDDs) per 1000 population
Enterobacter spp.	Carbapenems	Physicians per capita, Proportion of population involved in agricultural activities, Hospital Beds (per 1000), ORS (oral rehydration), Outdoor Air Pollution (PM2.5), Pharmacists per capita, Population-weighted mean temperature, Total Fertility Rate, Rotavirus coverage, COVID-free (proportion)
Enterobacter spp.	Fluoroquinolones	Physicians per capita, Proportion of population involved in agricultural activities, Average latitude, Hospital Beds (per 1000), Outdoor Air Pollution (PM2.5), Pharmacists per capita, Diabetes Fasting Plasma Glucose (mmol/L), by age
Enterobacter spp.	Fourth-generation cephalosporins	Fraction of OOP Health Expenditure, Proportion of population involved in agricultural activities, Outdoor Air Pollution (PM2.5), Pharmacists per capita, Smoking Prevalence, Tuberculosis prevalence (age-standardized), Dentists per capita, Diabetes Fasting Plasma Glucose (mmol/L), by age, Hib3 Vaccine Coverage, COVID-free (proportion)
Enterobacter spp.	Trimethoprim-Sulfamethoxazole	Fraction of OOP Health Expenditure, Healthcare access and quality index, Outdoor Air Pollution (PM2.5), Age-standardized SEV for Unsafe sanitation, Dentists per capita, Diabetes Fasting Plasma Glucose (mmol/L), by age, PCV3 lagged five year coverage, COVID-free (proportion), Hib3 Vaccine Coverage, COVID-free (proportion), Estimated rate of J01E sulfonamides and trimethoprim consumption in defined daily doses (DDDs) per 1000 population
Enterococcus faecalis	Fluoroquinolones	Hospital Beds (per 1000), Pharmacists per capita, Smoking Prevalence, Tuberculosis prevalence (age-standardized), Intravenous drug use (proportion by age), Dentists per capita, PCV3 lagged five year coverage, COVID-free (proportion)
Enterococcus faecalis	Vancomycin	Fraction of OOP Health Expenditure, Age-standardized SEV for Handwashing, Physicians per capita, Average latitude, Hospital Beds (per 1000), Pharmacists per capita, Smoking Prevalence, Intravenous drug use (proportion by age), Dentists per capita
Enterococcus faecium	Fluoroquinolones	Age-standardized SEV for Handwashing, Average latitude, HIV Prevalence Unadjusted (proportion), Pharmacists per capita, Population-weighted mean temperature, Maternal Education (years per capita), Tuberculosis prevalence (age-standardized), Intravenous drug use (proportion by age), Diabetes Fasting Plasma Glucose (mmol/L), by age, Hib3 lagged five year coverage, COVID-free (proportion)
Enterococcus faecium	Vancomycin	Diabetes Age-Standardized Prevalence (proportion), Age-standardized SEV for Handwashing, Physicians per capita, Healthcare access and quality index, Hospital Beds (per 1000), Rotavirus coverage, COVID-free (proportion), Intravenous drug use (proportion by age), Diabetes Fasting Plasma Glucose (mmol/L), by age, Estimated rate of antibiotic consumption in defined daily doses (DDDs) per 1000 population
Escherichia coli	Aminoglycosides	Diabetes Age-Standardized Prevalence (proportion), Healthcare access and quality index, Hospital Beds (per 1000), Outdoor Air Pollution (PM2.5), Age-standardized SEV for Unsafe sanitation, Maternal Education (years per capita), Smoking Prevalence, Rotavirus coverage, COVID-free (proportion), Hib3 Vaccine Coverage, COVID-free (proportion)
Escherichia coli	Aminopenicillin	Healthcare access and quality index, Hospital Beds (per 1000), Outdoor Air Pollution (PM2.5), Pharmacists per capita, Population Density (over 1000 ppl/sqkm, proportion), Tuberculosis prevalence (age-standardized)
Escherichia coli	Beta Lactam/Beta-lactamase inhibitors	Diabetes Age-Standardized Prevalence (proportion), Fraction of OOP Health Expenditure, Average latitude, ORS (oral rehydration), Outdoor Air Pollution (PM2.5), Age-standardized SEV for Unsafe

		sanitation, Smoking Prevalence, Dentists per capita, Vaccine adjusted HbSAg serporevalence age standardized
Escherichia coli	Carbapenems	Proportion of population involved in agricultural activities, Healthcare access and quality index, Hospital Beds (per 1000), Outdoor Air Pollution (PM2.5), Skilled Birth Attendance (proportion), Population-weighted mean temperature
Escherichia coli	Fluoroquinolones	Diabetes Age-Standardized Prevalence (proportion), Physicians per capita, Proportion of population involved in agricultural activities, Estimated rate of J01M quinolones consumption in defined daily doses (DDDs) per 1000 population
Escherichia coli	Trimethoprim-Sulfamethoxazole	Diabetes Age-Standardized Prevalence (proportion), Fraction of OOP Health Expenditure, Healthcare access and quality index, Hospital Beds (per 1000), Outdoor Air Pollution (PM2.5), Pharmacists per capita, Total Fertility Rate, Age-standardized SEV for Unsafe sanitation, Dentists per capita
Escherichia coli	Third-generation cephalosporins	Proportion of population involved in agricultural activities, Pigs (per capita), Physicians per capita, Diabetes Age-Standardized Prevalence (proportion), Fraction of OOP Health Expenditure, Age-standardized SEV for Handwashing, Estimated rate of J01D other beta-lactams consumption in defined daily doses (DDDs) per 1000 population
Group A *Streptococcus*	Macrolide	Proportion of population involved in agricultural activities, Average latitude, ORS (oral rehydration), Age-standardized SEV for Unsafe sanitation, Population Density (over 1000 ppl/sqkm, proportion), Diabetes Fasting Plasma Glucose (mmol/L), by age, Vaccine adjusted HbSAg serporevalence age standardized, Hib3 lagged five year coverage, COVID-free (proportion)
Group B *Streptococcus*	Fluoroquinolones	Hospital Beds (per 1000), Outdoor Air Pollution (PM2.5), Pharmacists per capita, Population Density (over 1000 ppl/sqkm, proportion), Intravenous drug use (proportion by age), Diabetes Fasting Plasma Glucose (mmol/L), by age, PCV3 lagged five year coverage, COVID-free (proportion), Vaccine adjusted HbSAg serporevalence age standardized,Hib3 Vaccine Coverage, COVID-free (proportion)
Group B *Streptococcus*	Macrolide	Diabetes Age-Standardized Prevalence (proportion), Physicians per capita, Pigs (per capita), Hospital Beds (per 1000), ORS (oral rehydration), Skilled Birth Attendance (proportion), Population Density (over 1000 ppl/sqkm, proportion), Rotavirus coverage, COVID-free (proportion), Vaccine adjusted HbSAg serporevalence age standardized
Group B *Streptococcus*	Penicillin	Pigs (per capita), Hospital Beds (per 1000), Dentists per capita, Vaccine adjusted HbSAg serporevalence age standardized
Haemophilus influenzae	Aminopenicillin	Hospital Beds (per 1000), ORS (oral rehydration), Pharmacists per capita, Population Density (over 1000 ppl/sqkm, proportion), PCV3 lagged five year coverage, COVID-free (proportion), Estimated rate of J01C penicillins consumption in defined daily doses (DDDs) per 1000 population
Haemophilus influenzae	Third-generation cephalosporins	Proportion of population involved in agricultural activities, HIV Prevalence Unadjusted (proportion), Pharmacists per capita, Skilled Birth Attendance (proportion), Population-weighted mean temperature, Smoking Prevalence, Rotavirus coverage, COVID-free (proportion), Tuberculosis prevalence (age-standardized), Dentists per capita
Klebsiella pneumoniae	Aminoglycosides	Fraction of OOP Health Expenditure, Physicians per capita, Healthcare access and quality index, Hospital Beds (per 1000), ORS (oral rehydration), Outdoor Air Pollution (PM2.5), Pharmacists per capita, Population Density (over 1000 ppl/sqkm, proportion), Smoking Prevalence
Klebsiella pneumoniae	Beta Lactam/Beta-lactamase inhibitors	Fraction of OOP Health Expenditure, Age-standardized SEV for Handwashing, Physicians per capita, Average latitude, ORS (oral rehydration), Outdoor Air Pollution (PM2.5), Age-standardized SEV for Unsafe sanitation, Population Density (over 1000 ppl/sqkm, proportion), Smoking Prevalence
Klebsiella pneumoniae	Carbapenems	Estimated rate of antibiotic consumption in defined daily doses (DDDs) per 1000 population, Healthcare access and quality index, Hospital Beds (per 1000), Estimated rate of J01D other beta-lactams consumption in defined daily doses (DDDs) per 1000 population, Average latitude
Klebsiella pneumoniae	Fluoroquinolones	Physicians per capita, Healthcare access and quality index, Hospital Beds (per 1000), ORS (oral rehydration), Outdoor Air Pollution (PM2.5), Pharmacists per capita, Total Fertility Rate, Population Density (over 1000 ppl/sqkm, proportion), Smoking Prevalence
Klebsiella pneumoniae	Trimethoprim-Sulfamethoxazole	Age-standardized SEV for Handwashing, Healthcare access and quality index, Hospital Beds (per 1000), HIV Prevalence Unadjusted (proportion), HIV age-standardized Prevalence, ORS (oral rehydration),

		Outdoor Air Pollution (PM2.5), Dentists per capita, Diabetes Fasting Plasma Glucose (mmol/L), by age, Hib3 Vaccine Coverage, COVID-free (proportion)
Klebsiella pneumoniae	Third-generation cephalosporins	Physicians per capita, Estimated rate of J01D other beta-lactams consumption in defined daily doses (DDDs) per 1000 population, Estimated rate of antibiotic consumption in defined daily doses (DDDs) per 1000 population, HIV Prevalence Unadjusted (proportion), Estimated rate of J01C penicillins consumption in defined daily doses (DDDs) per 1000 population
Morganella spp.	Fluoroquinolones	Physicians per capita, Hospital Beds (per 1000), ORS (oral rehydration), Outdoor Air Pollution (PM2.5), Pharmacists per capita, Population Density (over 1000 ppl/sqkm, proportion), Diabetes Fasting Plasma Glucose (mmol/L), by age, PCV3 lagged five year coverage, COVID-free (proportion), Hib3 Vaccine Coverage, COVID-free (proportion)
Morganella spp.	Fourth-generation cephalosporins	Pigs (per capita), Proportion of population involved in agricultural activities, Healthcare access and quality index, Hospital Beds (per 1000), HIV Prevalence Unadjusted (proportion), ORS (oral rehydration), Outdoor Air Pollution (PM2.5), Total Fertility Rate, Age-standardized SEV for Unsafe sanitation, Antenatal Care (4 visits) Coverage (proportion)
Morganella spp.	Third-generation cephalosporins	Physicians per capita, Pigs (per capita), Average latitude, Hospital Beds (per 1000), HIV age-standardized Prevalence, Skilled Birth Attendance (proportion), Antenatal Care (4 visits) Coverage (proportion), Diabetes Fasting Plasma Glucose (mmol/L), by age, PCV3 lagged five year coverage, COVID-free (proportion), Estimated rate of antibiotic consumption in defined daily doses (DDDs) per 1000 population
Neisseria gonorrhoeae	Fluoroquinolones	Fraction of OOP Health Expenditure, Physicians per capita, Hospital Beds (per 1000), ORS (oral rehydration), Outdoor Air Pollution (PM2.5), Population Density (over 1000 ppl/sqkm, proportion), Rotavirus coverage, COVID-free (proportion), Intravenous drug use (proportion by age), PCV3 lagged five year coverage, COVID-free (proportion), Estimated rate of antibiotic consumption in defined daily doses (DDDs) per 1000 population
Neisseria gonorrhoeae	Third-generation cephalosporins	Average latitude, Hospital Beds (per 1000), HIV Prevalence Unadjusted (proportion), Population-weighted mean temperature, Age-standardized SEV for Unsafe sanitation, Rotavirus coverage, COVID-free (proportion), PCV3 lagged five year coverage, COVID-free (proportion), Vaccine adjusted HbSAg serporevalence age standardized, Estimated rate of J01C penicillins consumption in defined daily doses (DDDs) per 1000 population, Estimated rate of J01D other beta-lactams consumption in defined daily doses (DDDs) per 1000 population
Non-typhoidal *salmonella*	Fluoroquinolones	Physicians per capita, Hospital Beds (per 1000), HIV Prevalence Unadjusted (proportion), HIV age-standardized Prevalence, Population-weighted mean temperature, Total Fertility Rate, Rotavirus coverage, COVID-free (proportion), Intravenous drug use (proportion by age), Dentists per capita, Hib3 Vaccine Coverage, COVID-free (proportion)
Proteus spp.	Aminoglycosides	Physicians per capita, Average latitude, Healthcare access and quality index, Hospital Beds (per 1000), ORS (oral rehydration), Total Fertility Rate, Population Density (over 1000 ppl/sqkm, proportion), Dentists per capita, Vaccine adjusted HbSAg serporevalence age standardized
Proteus spp.	Aminopenicillin	Physicians per capita, HIV age-standardized Prevalence, Outdoor Air Pollution (PM2.5), Population-weighted mean temperature, Total Fertility Rate, Intravenous drug use (proportion by age), Estimated rate of antibiotic consumption in defined daily doses (DDDs) per 1000 population
Proteus spp.	Fluoroquinolones	Hospital Beds (per 1000), Total Fertility Rate, Population Density (over 1000 ppl/sqkm, proportion), Intravenous drug use (proportion by age), Diabetes Fasting Plasma Glucose (mmol/L), by age, Vaccine adjusted HbSAg serporevalence age standardized, Estimated rate of antibiotic consumption in defined daily doses (DDDs) per 1000 population
Proteus spp.	Trimethoprim-Sulfamethoxazole	Fraction of OOP Health Expenditure, Pigs (per capita), ORS (oral rehydration), Outdoor Air Pollution (PM2.5), Total Fertility Rate, Smoking Prevalence, Rotavirus coverage, COVID-free (proportion), Dentists per capita, Vaccine adjusted HbSAg serporevalence age standardized
Proteus spp.	Third-generation cephalosporins	Pigs (per capita), Average latitude, HIV Prevalence Unadjusted (proportion), Outdoor Air Pollution (PM2.5), Skilled Birth Attendance (proportion), Population-weighted mean temperature, Maternal Education (years per capita), Hib3 lagged five year coverage, COVID-free (proportion)

Pseudomonas aeruginosa	Aminoglycosides	Fraction of OOP Health Expenditure, Healthcare access and quality index, ORS (oral rehydration), Pharmacists per capita, Population Density (over 1000 ppl/sqkm, proportion), PCV3 lagged five year coverage, COVID-free (proportion)
Pseudomonas aeruginosa	Anti-pseudomonal penicillin/Beta-Lactamase inhibitors	Fraction of OOP Health Expenditure, Average latitude, Healthcare access and quality index, Hospital Beds (per 1000), ORS (oral rehydration), Pharmacists per capita, Population-weighted mean temperature, Population Density (over 1000 ppl/sqkm, proportion), Hib3 Vaccine Coverage, COVID-free (proportion)
Pseudomonas aeruginosa	Carbapenems	Diabetes Age-Standardized Prevalence (proportion), Fraction of OOP Health Expenditure, Physicians per capita, Hospital Beds (per 1000), ORS (oral rehydration), Pharmacists per capita, Population Density (over 1000 ppl/sqkm, proportion), Smoking Prevalence, Dentists per capita
Pseudomonas aeruginosa	Fluoroquinolones	Healthcare access and quality index, Hospital Beds (per 1000), ORS (oral rehydration), Pharmacists per capita, Population Density (over 1000 ppl/sqkm, proportion), Intravenous drug use (proportion by age), PCV3 lagged five year coverage, COVID-free (proportion), Vaccine adjusted HbSAg serporevalence age standardized
Pseudomonas aeruginosa	Fourth-generation cephalosporins	Diabetes Age-Standardized Prevalence (proportion), Fraction of OOP Health Expenditure, Healthcare access and quality index, Hospital Beds (per 1000), ORS (oral rehydration), Outdoor Air Pollution (PM2.5), Pharmacists per capita, Population Density (over 1000 ppl/sqkm, proportion), Dentists per capita, PCV3 lagged five year coverage, COVID-free (proportion)
Pseudomonas aeruginosa	Third-generation cephalosporins	Fraction of OOP Health Expenditure, Proportion of population involved in agricultural activities, Healthcare access and quality index, Hospital Beds (per 1000), ORS (oral rehydration), Outdoor Air Pollution (PM2.5), Pharmacists per capita, Population Density (over 1000 ppl/sqkm, proportion), PCV3 lagged five year coverage, COVID-free (proportion)
Salmonella Paratyphi	Fluoroquinolones	Diabetes Age-Standardized Prevalence (proportion), Estimated rate of J01M quinolones consumption in defined daily doses (DDDs) per 1000 population
Salmonella Paratyphi	Multi-drug resistance	Age-standardized SEV for Unsafe sanitation, Population-weighted mean temperature, Outdoor Air Pollution (PM2.5), Physicians per capita, Total Fertility Rate
Salmonella Typhi	Fluoroquinolones	Estimated rate of J01M quinolones consumption in defined daily doses (DDDs) per 1000 population, Outdoor Air Pollution (PM2.5), Total Fertility Rate, Estimated rate of antibiotic consumption in defined daily doses (DDDs) per 1000 population
Salmonella Typhi	Multi-drug resistance	Estimated rate of antibiotic consumption in defined daily doses (DDDs) per 1000 population, Population-weighted mean temperature, Antenatal Care (4 visits) Coverage (proportion), Hospital Beds (per 1000), Age-standardized SEV for Unsafe sanitation, Estimated rate of J01C penicillins consumption in defined daily doses (DDDs) per 1000 population, Total Fertility Rate
Serratia spp.	Aminoglycosides	Fraction of OOP Health Expenditure, Average latitude, Healthcare access and quality index, Hospital Beds (per 1000), HIV age-standardized Prevalence, PCV3 lagged five year coverage, COVID-free (proportion), Hib3 Vaccine Coverage, COVID-free (proportion)
Serratia spp.	Anti-pseudomonal penicillin/Beta-Lactamase inhibitors	Physicians per capita, Pigs (per capita), Proportion of population involved in agricultural activities, Healthcare access and quality index, HIV age-standardized Prevalence, Outdoor Air Pollution (PM2.5), Maternal Education (years per capita), Intravenous drug use (proportion by age), Dentists per capita
Serratia spp.	Carbapenems	Proportion of population involved in agricultural activities, Average latitude, ORS (oral rehydration), Maternal Education (years per capita), Smoking Prevalence, Rotavirus coverage, COVID-free (proportion), Hepatitis C Seroprevalence (anti-HCV) age standardized, PCV3 lagged five year coverage, COVID-free (proportion), Hib3 lagged five year coverage, COVID-free (proportion)
Serratia spp.	Fluoroquinolones	Fraction of OOP Health Expenditure, Proportion of population involved in agricultural activities, Average latitude, Healthcare access and quality index, Outdoor Air Pollution (PM2.5), Pharmacists per capita, Maternal Education (years per capita), Diabetes Fasting Plasma Glucose (mmol/L), by age, Hepatitis C Seroprevalence (anti-HCV) age standardized
Serratia spp.	Fourth-generation cephalosporins	Physicians per capita, Proportion of population involved in agricultural activities, Average latitude, Healthcare access and quality index, HIV Prevalence Unadjusted (proportion), ORS (oral rehydration),

		Pharmacists per capita, Maternal Education (years per capita), Diabetes Fasting Plasma Glucose (mmol/L), by age
Serratia spp.	Third-generation cephalosporins	Diabetes Age-Standardized Prevalence (proportion), Fraction of OOP Health Expenditure, Physicians per capita, Pigs (per capita), Healthcare access and quality index, HIV Prevalence Unadjusted (proportion), Outdoor Air Pollution (PM2.5), Skilled Birth Attendance (proportion), Dentists per capita
Shigella spp.	Fluoroquinolones	Age-standardized SEV for Handwashing, ORS (oral rehydration), Pharmacists per capita, Average latitude, Estimated rate of antibiotic consumption in defined daily doses (DDDs) per 1000 population, Estimated rate of J01M quinolones consumption in defined daily doses (DDDs) per 1000 population
Staphylococcus aureus	Fluoroquinolones	Diabetes Age-Standardized Prevalence (proportion), Average latitude, HIV Prevalence Unadjusted (proportion), ORS (oral rehydration), Outdoor Air Pollution (PM2.5), Age-standardized SEV for Unsafe sanitation, Rotavirus coverage, COVID-free (proportion), Intravenous drug use (proportion by age), Diabetes Fasting Plasma Glucose (mmol/L), by age
Staphylococcus aureus	Macrolide	Diabetes Age-Standardized Prevalence (proportion), Pigs (per capita), Average latitude, ORS (oral rehydration), Population Density (over 1000 ppl/sqkm, proportion), Intravenous drug use (proportion by age), Vaccine adjusted HbSAg serporevalence age standardized
Staphylococcus aureus	Methicillin	Diabetes Age-Standardized Prevalence (proportion), Estimated rate of J01C penicillins consumption in defined daily doses (DDDs) per 1000 population, Estimated rate of J01M quinolones consumption in defined daily doses (DDDs) per 1000 population
Staphylococcus aureus	Trimethoprim-Sulfamethoxazole	Fraction of OOP Health Expenditure, Healthcare access and quality index, Hospital Beds (per 1000), Outdoor Air Pollution (PM2.5), Age-standardized SEV for Unsafe sanitation, Maternal Education (years per capita), Tuberculosis prevalence (age-standardized), Dentists per capita, Hepatitis C Seroprevalence (anti-HCV) age standardized
Staphylococcus aureus	Vancomycin	Fraction of OOP Health Expenditure, Physicians per capita, Proportion of population involved in agricultural activities, Hospital Beds (per 1000), HIV age-standardized Prevalence, ORS (oral rehydration), Outdoor Air Pollution (PM2.5), Skilled Birth Attendance (proportion), Antenatal Care (4 visits) Coverage (proportion)
Streptococcus pneumoniae	Beta Lactam/Beta-lactamase inhibitors	Diabetes Age-Standardized Prevalence (proportion), Fraction of OOP Health Expenditure, Physicians per capita, Average latitude, Hospital Beds (per 1000), Outdoor Air Pollution (PM2.5), Pharmacists per capita, Skilled Birth Attendance (proportion), Age-standardized SEV for Unsafe sanitation, Population Density (over 1000 ppl/sqkm, proportion)
Streptococcus pneumoniae	Carbapenems	Diabetes Age-Standardized Prevalence (proportion), Physicians per capita, Outdoor Air Pollution (PM2.5), Skilled Birth Attendance (proportion), Population-weighted mean temperature, Population Density (over 1000 ppl/sqkm, proportion), Rotavirus coverage, COVID-free (proportion), Diabetes Fasting Plasma Glucose (mmol/L), by age, Hepatitis C Seroprevalence (anti-HCV) age standardized
Streptococcus pneumoniae	Fluoroquinolones	Diabetes Age-Standardized Prevalence (proportion), Age-standardized SEV for Handwashing, Physicians per capita, Outdoor Air Pollution (PM2.5), Skilled Birth Attendance (proportion), Age-standardized SEV for Unsafe sanitation, Population Density (over 1000 ppl/sqkm, proportion), Smoking Prevalence, Rotavirus coverage, COVID-free (proportion)
Streptococcus pneumoniae	Macrolide	Diabetes Age-Standardized Prevalence (proportion), Physicians per capita, Hospital Beds (per 1000), Outdoor Air Pollution (PM2.5), Population-weighted mean temperature, Diabetes Fasting Plasma Glucose (mmol/L), by age, Vaccine adjusted HbSAg serporevalence age standardize, Estimated rate of J01F macrolides, lincosamides and streptogramins consumption in defined daily doses (DDDs) per 1000 population
Streptococcus pneumoniae	Penicillin	Fraction of OOP Health Expenditure, Hospital Beds (per 1000), Estimated rate of J01C penicillins consumption in defined daily doses (DDDs) per 1000 population, Maternal Education (years per capita), Pharmacists per capita, Estimated rate of antibiotic consumption in defined daily doses (DDDs) per 1000 population

Streptococcus pneumoniae	Trimethoprim-Sulfamethoxazole	Physicians per capita, Pigs (per capita), Proportion of population involved in agricultural activities, Average latitude, Outdoor Air Pollution (PM2.5), Age-standardized SEV for Unsafe sanitation, Maternal Education (years per capita), Diabetes Fasting Plasma Glucose (mmol/L), by age, PCV3 lagged five year coverage, COVID-free (proportion)
Streptococcus pneumoniae	Third-generation cephalosporins	Diabetes Age-Standardized Prevalence (proportion), Age-standardized SEV for Handwashing, Physicians per capita, Pigs (per capita), Proportion of population involved in agricultural activities, Average latitude, Healthcare access and quality index, HIV age-standardized Prevalence, ORS (oral rehydration), Outdoor Air Pollution (PM2.5)

Table S5: Summary of relative risk data

Antibiotic class	Pathogen	Literature			Microbiology		
		Cases	Deaths	Days of Infection	Cases	Deaths	Days of Infection
Aminoglycosides	*Acinetobacter baumannii*	-	-	-	58,717	5,821	1,350,226
Aminoglycosides	*Citrobacter* spp.	-	-	-	66,515	1,675	795,364
Aminoglycosides	*Enterobacter* spp.	-	-	-	191,998	4,985	3,112,166
Aminoglycosides	*Escherichia coli*	-	-	-	2,188,899	43,629	19,101,791
Aminoglycosides	*Klebsiella pneumoniae*	-	-	-	711,728	22,201	9,059,998
Aminoglycosides	*Proteus* spp.	-	-	-	383,719	7,458	4,358,494
Aminoglycosides	*Pseudomonas aeruginosa*	-	-	-	746,320	23,497	14,479,385
Aminoglycosides	*Serratia* spp.	-	-	-	92,519	2,757	1,803,213
Aminopenicillin	*Escherichia coli*	-	-	-	1,014,811	21,282	8,745,596
Aminopenicillin	*Haemophilus influenzae*	1,403	107	-	6,210	396	43,483
Aminopenicillin	*Proteus* spp.	-	-	-	170,905	3,413	1,934,044
Anti-pseudomonal penicillin/Beta-Lactamase inhibitors	*Acinetobacter baumannii*	-	-	-	18,204	2,454	374,988
Anti-pseudomonal penicillin/Beta-Lactamase inhibitors	*Citrobacter* spp.	-	-	-	25,561	756	307,904
Anti-pseudomonal penicillin/Beta-Lactamase inhibitors	*Enterobacter* spp.	-	-	-	81,729	2,835	1,304,804
Anti-pseudomonal penicillin/Beta-Lactamase inhibitors	*Pseudomonas aeruginosa*	-	-	-	318,376	14,430	6,006,891
Anti-pseudomonal penicillin/Beta-Lactamase inhibitors	*Serratia* spp.	-	-	-	29,701	1,214	624,717
Beta Lactam/Beta-lactamase inhibitors	*Acinetobacter baumannii*	-	-	-	22,291	1,602	515,184
Beta Lactam/Beta-lactamase inhibitors	*Escherichia coli*	-	-	-	1,178,102	26,078	9,985,178
Beta Lactam/Beta-lactamase inhibitors	*Klebsiella pneumoniae*	-	-	-	392,472	12,853	4,737,893
Beta Lactam/Beta-lactamase inhibitors	*Streptococcus pneumoniae*	-	-	-	6,400	256	50,191
Carbapenems	*Acinetobacter baumannii*	2,603	939	-	51,149	10,157	821,619
Carbapenems	*Citrobacter* spp.	-	-	-	44,096	1,712	518,670
Carbapenems	*Enterobacter* spp.	985	318	-	129,218	5,720	2,025,267
Carbapenems	*Escherichia coli*	220	51	-	1,384,533	36,620	12,287,750
Carbapenems	*Klebsiella pneumoniae*	5,019	1,180	-	463,800	21,608	5,874,759
Carbapenems	*Pseudomonas aeruginosa*	6,141	1,234	-	377,712	20,544	7,359,610

Carbapenems	*Serratia* spp.	-	-	-	60,069	2,799	1,172,118
Carbapenems	*Streptococcus pneumoniae*	-	-	-	11,976	433	101,018
Fluoroquinolones	*Acinetobacter baumannii*	-	-	-	44,514	5,040	920,862
Fluoroquinolones	*Citrobacter* spp.	-	-	-	51,125	1,581	572,926
Fluoroquinolones	*Enterobacter* spp.	-	-	-	142,227	4,404	2,157,754
Fluoroquinolones	*Enterococcus faecalis*	-	-	-	13,482	1,456	148,347
Fluoroquinolones	*Enterococcus faecium*	-	-	-	52,718	2,923	800,011
Fluoroquinolones	*Escherichia coli*	5,734	600	-	1,660,159	36,469	14,065,116
Fluoroquinolones	Group B *Streptococcus*	-	-	-	1,973	140	18,042
Fluoroquinolones	*Klebsiella pneumoniae*	-	-	-	533,219	18,867	6,418,092
Fluoroquinolones	*Morganella* spp.	-	-	-	50,872	1,174	575,814
Fluoroquinolones	*Neisseria gonorrhoeae*	-	-	-	179	0	123
Fluoroquinolones	*Non-typhoidal Salmonella*	-	-	-	7,489	67	36,416
Fluoroquinolones	Other *enterococci*	-	-	-	72	14	15,396
Fluoroquinolones	*Proteus* spp.	-	-	-	284,542	6,154	3,196,494
Fluoroquinolones	*Pseudomonas aeruginosa*	-	-	-	474,356	20,427	8,453,680
Fluoroquinolones	*Salmonella* Paratyphi	-	-	-	1,586	1	12,639
Fluoroquinolones	*Salmonella* Typhi	-	-	-	12,065	16	22,713
Fluoroquinolones	*Serratia* spp.	-	-	-	69,775	2,536	1,280,786
Fluoroquinolones	*Shigella* spp.	-	-	-	2,367	6	7,971
Fluoroquinolones	*Staphylococcus aureus*	-	-	-	780,617	18,092	8,498,172
Fluoroquinolones	*Streptococcus pneumoniae*	233	31	-	43,060	1,575	385,880
Fourth-generation cephalosporins	*Acinetobacter baumannii*	-	-	-	29,311	4,759	482,186
Fourth-generation cephalosporins	*Citrobacter* spp.	-	-	-	23,273	656	280,528
Fourth-generation cephalosporins	*Enterobacter* spp.	-	-	-	72,621	2,422	1,177,713
Fourth-generation cephalosporins	*Morganella* spp.	-	-	-	23947	495	295266
Fourth-generation cephalosporins	*Pseudomonas aeruginosa*	-	-	-	284,345	10,529	5,428,710
Fourth-generation cephalosporins	*Serratia* spp.	-	-	-	35,865	1,238	693,294
Macrolide	Group A *Streptococcus*	-	-	-	2,565	438	34,491

Macrolide	Group B *Streptococcus*	432	48	-	2,739	213	30,417
Macrolide	*Staphylococcus aureus*	-	-	-	670,956	15,624	7,915,114
Macrolide	*Streptococcus pneumoniae*	871	123	-	41,700	1,882	375,517
Methicillin	*Staphylococcus aureus*	24,236	4,520	-	960,853	27,728	11,263,054
Penicillin	Group B *Streptococcus*	-	-	-	3,180	250	31,891
Penicillin	*Streptococcus pneumoniae*	9,071	1,539	-	131,214	5,063	476,341
Third-generation cephalosporins	*Acinetobacter baumannii*	-	-	-	54,559	8,485	907,736
Third-generation cephalosporins	*Citrobacter* spp.	-	-	-	55,751	1,910	639,522
Third-generation cephalosporins	*Escherichia coli*	15,170	2,052	-	1,553,658	39,801	13,481,857
Third-generation cephalosporins	*Haemophilus influenzae*	-	-	-	2,532	251	16,050
Third-generation cephalosporins	*Klebsiella pneumoniae*	1,552	417	-	519,223	23,371	6,442,738
Third-generation cephalosporins	*Morganella* spp.	-	-	-	54,598	1,395	624,869
Third-generation cephalosporins	*Neisseria gonorrhoeae*	-	-	-	188	2	177
Third-generation cephalosporins	*Proteus* spp.	-	-	-	260,333	6,352	2,986,334
Third-generation cephalosporins	*Pseudomonas aeruginosa*	1,975	762	-	291,397	14,800	5,293,386
Third-generation cephalosporins	*Serratia* spp.	-	-	-	78,264	3,456	1,451,520
Third-generation cephalosporins	*Streptococcus pneumoniae*	5,201	703	-	154,698	5,199	686,272
Trimethoprim-Sulfamethoxazole	*Enterobacter* spp.	-	-	-	85,985	2,255	1,367,747
Trimethoprim-Sulfamethoxazole	*Escherichia coli*	-	-	-	1,046,932	21,657	8,972,560
Trimethoprim-Sulfamethoxazole	*Klebsiella pneumoniae*	-	-	-	331,976	9,731	4,142,308
Trimethoprim-Sulfamethoxazole	*Proteus* spp.	-	-	-	175,778	3,547	1,975,356
Trimethoprim-Sulfamethoxazole	*Staphylococcus aureus*	-	-	-	724,647	16,338	8,583,645
Trimethoprim-Sulfamethoxazole	*Streptococcus pneumoniae*	-	-	-	28,919	1,066	256,030
Vancomycin	*Enterococcus faecalis*	5,366	1,702	-	12,095	1,915	129,245
Vancomycin	*Enterococcus faecium*	2,516	792	-	90,846	5,519	1,725,901
Vancomycin	Other *enterococci*	1	-	-	83	13	15,914
Vancomycin	*Staphylococcus aureus*	-	-	-	719,473	18,681	8,661,298
Total		88,729	17,117	-	22,978,627	677,168	263,284,542

This table highlights the line level data that we received. Literature sources that provided information on the relative risk of increased length of stay (LOS) provided mean LOS between resistant and susceptible infections or the interquartile range (IQR) which we incorporated as well.

Table S6: GATHER checklist

Item #	Checklist item	Reporting location
Objectives and funding		
1	Define the indicator(s), populations (including age, sex, and geographic entities), and time period(s) for which estimates were made.	Main text summary (methods)
2	List the funding sources for the work.	Main text summary (funding)
Data Inputs		
For all data inputs from multiple sources that are synthesized as part of the study:		
3	Describe how the data were identified and how the data were accessed.	Main text methods section + supplementary appendix (sections 2, 4.1, 5.1, 6.1, 7.1, 8.1)
4	Specify the inclusion and exclusion criteria. Identify all ad-hoc exclusions.	Supplementary appendix (section 2)
5	Provide information on all included data sources and their main characteristics. For each data source used, report reference information or contact name/institution, population represented, data collection method, year(s) of data collection, sex and age range, diagnostic criteria or measurement method, and sample size, as relevant.	Supplementary appendix (section 2). The main characteristics of data, metadata, and/or NIDs available at https://ghdx.healthdata.org/gbd-2021/sources
6	Identify and describe any categories of input data that have potentially important biases (e.g., based on characteristics listed in item 5).	Main text (limitations) + supplementary appendix (biases for input data in each modelling step identified in each section)
For data inputs that contribute to the analysis but were not synthesized as part of the study:		
7	Describe and give sources for any other data inputs.	GBD 2021 estimates (http://ghdx.healthdata.org/gbd-results-tool)
For all data inputs:		
8	Provide all data inputs in a file format from which data can be efficiently extracted (e.g., a spreadsheet rather than a PDF), including all relevant meta-data listed in item 5. For any data inputs that cannot be shared because of ethical or legal reasons, such as third-party ownership, provide a contact name or the name of the institution that retains the right to the data.	Data inputs and/or contact information available at https://ghdx.healthdata.org/gbd-2021/sources
Data analysis		
9	Provide a conceptual overview of the data analysis method. A diagram may be helpful.	Main text methods section + supplementary figure S1 (flowchart of methods)
10	Provide a detailed description of all steps of the analysis, including mathematical formulae. This description should cover, as relevant, data cleaning, data pre-processing, data adjustments and weighting of data sources, and mathematical or statistical model(s).	Supplementary appendix (sections 4-11)

11	Describe how candidate models were evaluated and how the final model(s) were selected.	Supplementary appendix (sections 4-11)
12	Provide the results of an evaluation of model performance, if done, as well as the results of any relevant sensitivity analysis.	Supplementary appendix (sections 4.7, 6.5, 7.6, 8.4)
13	Describe methods for calculating uncertainty of the estimates. State which sources of uncertainty were, and were not, accounted for in the uncertainty analysis.	Main text methods section (uncertainty analysis) + limitations section + supplementary appendix (sections 4-8).
14	State how analytic or statistical source code used to generate estimates can be accessed.	Link to GitHub code found in main text methods section
Results and Discussion		
15	Provide published estimates in a file format from which data can be efficiently extracted.	Published estimates are available in the main text results section and in the supplementary appendix. CSV files are available upon request to the corresponding author
16	Report a quantitative measure of the uncertainty of the estimates (e.g. uncertainty intervals).	Uncertainty intervals are provided for all estimates throughout the main text.
17	Interpret results in light of existing evidence. If updating a previous set of estimates, describe the reasons for changes in estimates.	Main text research in context, introduction, and discussion sections
18	Discuss limitations of the estimates. Include a discussion of any modelling assumptions or data limitations that affect interpretation of the estimates.	Main text limitations section + supplementary appendix

Table S7: Cumulative AMR associated and attributable death and DALY counts in millions, globally and by super-region in the reference scenario, 2025–2050.

Estimates are listed as means with 95% uncertainty intervals in parentheses. Highlighted rows indicate super region results from the GBD location hierarchy. Note that both North Africa and Middle East as well as South Asia are both GBD super regions and regions.

	Deaths		DALYs	
	Associated	Attributable	Associated	Attributable
Global	169.15 (144.89, 195.64)	39.11 (32.97, 46.04)	4,951.13 (4,152.46, 5,900.32)	1,136.87 (940.22, 1,384.85)
Central Europe, Eastern Europe, and Central Asia	8.4 (7.08, 9.75)	1.94 (1.62, 2.27)	191.69 (161.04, 221.65)	44.85 (37.33, 53.39)
Central Asia	1.4 (1.11, 1.74)	0.32 (0.25, 0.42)	46.8 (35.74, 59.8)	10.93 (7.94, 14.65)
Central Europe	2.35 (2.02, 2.68)	0.52 (0.45, 0.6)	43.53 (38.12, 49.47)	9.78 (8.55, 11.35)
Eastern Europe	4.65 (3.79, 5.49)	1.09 (0.87, 1.32)	101.35 (83.97, 120.1)	24.14 (19.28, 29.13)
High income	19.82 (15.55, 22.82)	4.29 (3.36, 4.96)	312.22 (263.33, 347.84)	68.32 (57.28, 76.58)
Australasia	0.4 (0.31, 0.47)	0.08 (0.06, 0.1)	6.08 (5.03, 7.02)	1.26 (1.01, 1.52)
High-income Asia Pacific	3.68 (2.68, 4.6)	0.79 (0.57, 0.98)	48.88 (38.58, 58.43)	10.55 (8.32, 12.72)
High-income North America	6.64 (5.29, 7.76)	1.47 (1.15, 1.72)	118.44 (99.85, 134.65)	26.41 (21.85, 30.29)
Southern Latin America	1.79 (1.49, 2.11)	0.42 (0.35, 0.5)	31.97 (27.03, 37.09)	31.97 (27.03, 37.09)
Western Europe	7.32 (5.77, 8.42)	1.53 (1.19, 1.76)	106.86 (89.54, 119.64)	22.43 (18.64, 25.42)
Latin America and Caribbean	13.12 (10.9, 15.58)	3.01 (2.45, 3.62)	298.29 (249.19, 355.43)	69.02 (57.04, 82.25)
Andean Latin America	1.42 (1.1, 1.81)	0.32 (0.25, 0.41)	32.28 (25.32, 41.77)	7.24 (5.69, 9.38)
Caribbean	1.15 (0.94, 1.43)	0.26 (0.2, 0.34)	31.91 (24.76, 40.9)	7.18 (5.34, 9.53)
Central Latin America	4.89 (4.12, 5.69)	1.14 (0.95, 1.35)	118.44 (98.44, 140.78)	27.7 (22.8, 33.51)
Tropical Latin America	5.66 (4.42, 7.25)	1.3 (0.99, 1.65)	115.66 (93.83, 145.5)	26.89 (21.23, 33.89)
North Africa and Middle East	9.68 (8.1, 11.48)	2.45 (2.01, 2.97)	287.12 (232.13, 352.55)	73.14 (57.9, 91.36)
South Asia	47.2 (38.5, 56.92)	11.83 (9.43, 14.36)	1,420.4 (1,131.88, 1,721.52)	349.38 (272.91, 432.67)
Southeast Asia, East Asia, and Oceania	40.58 (33.64, 47.69)	8.96 (7.45, 10.44)	864.63 (742.25, 1,026.8)	192.6 (165.82, 226.97)
East Asia	24.18 (19.23, 30.32)	5.12 (4.03, 6.41)	444.6 (352.56, 564.8)	94.68 (74.76, 120.86)
Oceania	0.37 (0.26, 0.49)	0.08 (0.06, 0.11)	18.71 (12.79, 26.48)	4.15 (2.69, 5.92)
Southeast Asia	16.04 (13.84, 18.49)	3.76 (3.19, 4.38)	401.32 (340.55, 467.36)	93.77 (77.9, 110.73)
Sub-Saharan Africa	30.35 (23.51, 38.55)	6.63 (5.0, 8.66)	1,576.78 (1,140.36, 2,142.2)	339.57 (243.17, 470.31)

Central sub-Saharan Africa	3.46 (2.51, 4.67)	0.78 (0.53, 1.11)	152.55 (102.12, 218.64)	34.33 (22.65, 50.43)
Eastern sub-Saharan Africa	10.36 (8.34, 13.02)	2.29 (1.79, 2.95)	496.85 (374.58, 671.91)	108.75 (79.93, 148.67)
Southern sub-Saharan Africa	1.64 (1.36, 2.01)	0.37 (0.31, 0.46)	58.68 (46.67, 75.86)	13.22 (10.28, 17.12)
Western sub-Saharan Africa	14.89 (10.8, 19.9)	3.18 (2.28, 4.3)	868.7 (589.98, 1,226.75)	183.27 (123.28, 261.99)

Table S8: Cumulative total deaths avoided/excess in millions, globally and by super-region and scenario, 2025–2050.

Estimates are listed as means with 95% uncertainty intervals in parentheses. Highlighted rows indicate super region results from the GBD location hierarchy. Note that both North Africa and Middle East as well as South Asia are both GBD super regions and regions.

	Gram-Negative Drug	Better Care
Global	11.08 (9.08, 13.17)	92.02 (82.81, 101.75)
Central Europe, Eastern Europe, and Central Asia	0.55 (0.46, 0.66)	2.42 (2.17, 2.72)
Central Asia	0.09 (0.07, 0.11)	0.69 (0.59, 0.84)
Central Europe	0.13 (0.11, 0.15)	0.51 (0.45, 0.58)
Eastern Europe	0.33 (0.26, 0.4)	1.22 (1.05, 1.39)
High income	0.86 (0.66, 1.01)	2.63 (1.66, 3.84)
Australasia	0.02 (0.01, 0.02)	0.01 (-0.02, 0.05)
High-income Asia Pacific	0.12 (0.08, 0.17)	0.17 (-0.11, 0.44)
High-income North America	0.27 (0.2, 0.34)	1.09 (0.27, 1.97)
Southern Latin America	0.1 (0.08, 0.14)	0.37 (0.3, 0.45)
Western Europe	0.24 (0.17, 0.28)	0.8 (0.68, 0.92)
Latin America and Caribbean	0.82 (0.65, 1.02)	7.26 (6.36, 8.23)
Andean Latin America	0.08 (0.06, 0.11)	0.74 (0.61, 0.87)
Caribbean	0.08 (0.06, 0.1)	0.58 (0.49, 0.69)
Central Latin America	0.31 (0.25, 0.37)	2.59 (2.14, 3.16)
Tropical Latin America	0.36 (0.26, 0.49)	3.36 (2.75, 4.06)
North Africa and Middle East	0.66 (0.53, 0.8)	4.12 (3.43, 4.96)
South Asia	3.97 (3.1, 4.88)	31.66 (26.77, 37.24)
Southeast Asia, East Asia, and Oceania	2.17 (1.78, 2.68)	18.74 (14.42, 22.84)
East Asia	0.98 (0.72, 1.42)	8.25 (3.55, 12.51)
Oceania	0.02 (0.02, 0.03)	0.26 (0.21, 0.33)
Southeast Asia	1.16 (0.97, 1.38)	10.23 (9.09, 11.51)

Sub-Saharan Africa	2.05 (1.53, 2.65)	25.17 (21.15, 29.79)
Central sub-Saharan Africa	0.22 (0.15, 0.31)	3.31 (2.47, 4.3)
Eastern sub-Saharan Africa	0.68 (0.53, 0.88)	9.23 (7.68, 11.01)
Southern sub-Saharan Africa	0.09 (0.07, 0.11)	1.82 (1.59, 2.07)
Western sub-Saharan Africa	1.06 (0.76, 1.45)	10.81 (8.73, 13.15)

Table S9: Sepsis deaths and percent attributable to and associated with AMR by age, global, both sexes, 1990, 2019, 2021, and 2021 without COVID-19

Age	Year	Sepsis deaths	Deaths Associated with AMR	Deaths Attributable to AMR	Percent Associated with AMR	Percent Attributable to AMR
All Ages	1990	16,500,000 (15,700,000-17,300,000)	4,780,000 (4,000,000-5,550,000)	1,060,000 (841,000-1,270,000)	28.96 (25.54-32.04)	6.41 (5.37-7.36)
Under 5	1990	7,690,000 (7,160,000-8,220,000)	2,290,000 (1,850,000-2,720,000)	488,000 (374,000-602,000)	29.76 (25.89-33.13)	6.34 (5.22-7.32)
5 plus	1990	8,810,000 (8,300,000-9,320,000)	2,490,000 (2,100,000-2,880,000)	570,000 (458,000-682,000)	28.25 (25.33-30.86)	6.47 (5.52-7.32)
All Ages	2019	14,100,000 (13,200,000-15,100,000)	4,940,000 (4,430,000-5,450,000)	1,200,000 (1,050,000-1,350,000)	35.00 (33.67-36.16)	8.51 (8.00-8.95)
Under 5	2019	3,140,000 (2,660,000-3,620,000)	1,020,000 (808,000-1,240,000)	235,000 (182,000-288,000)	32.55 (30.35-34.16)	7.49 (6.84-7.96)
5 plus	2019	11,000,000 (10,200,000-11,700,000)	3,920,000 (3,540,000-4,300,000)	966,000 (851,000-1,080,000)	35.70 (34.55-36.71)	8.80 (8.32-9.22)
All Ages	2021	21,400,000 (20,300,000-22,400,000)	4,710,000 (4,230,000-5,190,000)	1,140,000 (1,000,000-1,280,000)	22.04 (20.85-23.12)	5.34 (4.95-5.70)
Under 5	2021	2,680,000 (2,190,000-3,180,000)	840,000 (640,000-1,040,000)	193,000 (144,000-242,000)	31.30 (29.23-32.72)	7.20 (6.59-7.62)
5 plus	2021	18,700,000 (17,800,000-19,600,000)	3,870,000 (3,480,000-4,260,000)	948,000 (837,000-1,060,000)	20.71 (19.57-21.75)	5.08 (4.71-5.41)
All Ages	2021 without COVID-19	13,500,000 (12,500,000-14,500,000)	4,710,000 (4,230,000-5,190,000)	1,140,000 (1,000,000-1,280,000)	34.95 (33.93-35.83)	8.47 (8.05-8.84)
Under 5	2021 without COVID-19	2,670,000 (2,180,000-3,160,000)	840,000 (640,000-1,040,000)	193,000 (144,000-242,000)	31.49 (29.42-32.92)	7.25 (6.64-7.67)
5 plus	2021 without COVID-19	10,800,000 (10,000,000-11,600,000)	3,870,000 (3,480,000-4,260,000)	948,000 (837,000-1,060,000)	35.80 (34.79-36.68)	8.78 (8.37-9.13)

Table S10: Summary of case fatality ratio data

Pathogen	Hospital	Microbiology	Total
Acinetobacter baumannii	0	53,896	53,896
Acinetobacter others	0	665	665
Actinomyces spp.	13,846	0	13,846
Adenovirus	43,888	0	43,888
Aeromonas spp.	0	768	768
Aspergillus spp.	80,029	43	80,072
Bacteria others	1,506,446	17,541	1,523,987
Burkholderia spp.	805	1,601	2,406
Candida spp.	724,485	8,281	732,766
Chlamydia spp.	49,837	52	49,889
Citrobacter spp.	0	23,190	23,190
Clostridium others	26,465	6	26,471
Cytomegalovirus	130,196	0	130,196
Entamoeba histolytica	27,757	0	27,757
Enterobacter spp.	0	76,882	76,882
Enterococcus faecalis	9,455	12,968	22,423
Enterococcus faecium	19,484	73,323	92,806
Enterococcus others	689	110	798
Epstein barr virus	126,647	0	126,647
Escherichia coli	2,080,661	967,300	3,047,961
Fungi others	64,063	33	64,096
Gram-negative others	172,418	23,345	195,763
Gram-negative unspecified	377,787	0	377,787
Gram-positive others	7,094	550	7,644
Haemophilus influenzae	89,708	7,784	97,492
Influenza virus	323,663	819	324,482
Klebsiella others	6,458	30,879	37,337
Klebsiella pneumoniae	102,169	293,904	396,073
Legionella spp.	30,784	160	30,944
Listeria spp.	3,859	127	3,986
Morganella spp.	0	17,758	17,758
Mumps	6,885	0	6,885
Mycobacterium others	72,538	3	72,541
Mycoplasma spp.	88,632	25	88,657
Neisseria meningitidis	12,608	645	13,253
Non-polio enteroviruses	150,729	0	150,729
Parasite others	99,762	0	99,762

Proteus spp.	240,216	121,898	362,114
Pseudomonas aeruginosa	698,362	268,879	967,241
Pseudomonas others	6,292	211	6,503
Respiratory syncytial virus	93,234	781	94,015
Serratia spp.	11,378	36,677	48,055
Staphylococcus aureus	2,246,504	733,059	2,979,562
Streptococcus group a	523,503	3,179	526,682
Streptococcus group b	1,412,071	3,400	1,415,471
Streptococcus others	67,710	1,597	69,307
Streptococcus pneumoniae	1,228,660	67,046	1,295,706
Toxoplasma spp.	34,487	0	34,487
Virus others	4,138,165	1	4,138,166
Total	17,150,428	2,849,385	19,999,813

Table S11: Number of isolates tested for antibiotic susceptibility considered as input data in the modelling of prevalence of resistance, by pathogen–drug combination.

Pathogen	Antibiotic class	Literature studies	Single drug resistance profiles	Microbial data	Total
Acinetobacter baumannii	Aminoglycosides	72,505	33,073	162,998	268,576
Acinetobacter baumannii	Anti-pseudomonal penicillin/Beta-Lactamase inhibitors	40,251	16,433	66,835	123,519
Acinetobacter baumannii	Beta Lactam/Beta-lactamase inhibitors	44,970	31,210	150,098	226,278
Acinetobacter baumannii	Carbapenems	33,910	59,202	272,759	365,871
Acinetobacter baumannii	Fluoroquinolones	34,903	32,768	215,209	282,880
Acinetobacter baumannii	Fourth-generation cephalosporins	9,007	17,116	80,872	106,995
Acinetobacter baumannii	Third-generation cephalosporins	51,178	65,419	232,172	348,769
Citrobacter spp.	Aminoglycosides	1,151	154,746	140,498	296,395
Citrobacter spp.	Anti-pseudomonal penicillin/Beta-Lactamase inhibitors	48	219,495	56,550	276,093
Citrobacter spp.	Carbapenems	101	275,463	173,046	448,610
Citrobacter spp.	Fluoroquinolones	404	215,884	145,223	361,511
Citrobacter spp.	Fourth-generation cephalosporins	8	115,926	59,056	174,990
Citrobacter spp.	Third-generation cephalosporins	723	351,680	163,306	515,709
Enterobacter spp.	Aminoglycosides	33,363	566,309	376,519	976,191
Enterobacter spp.	Anti-pseudomonal penicillin/Beta-Lactamase inhibitors	15,857	457,284	165,010	638,151
Enterobacter spp.	Carbapenems	8,654	563,006	494,010	1,065,670
Enterobacter spp.	Fluoroquinolones	11,026	437,718	416,730	865,474
Enterobacter spp.	Fourth-generation cephalosporins	1,666	234,271	163,496	399,433
Enterobacter spp.	Trimethoprim-Sulfamethoxazole	11,740	211,884	163,552	387,176
Enterococcus faecalis	Fluoroquinolones	1,331	635,404	194,438	831,173
Enterococcus faecalis	Vancomycin	712	635,340	287,292	923,344
Enterococcus faecium	Fluoroquinolones	877	149,696	127,759	278,332
Enterococcus faecium	Vancomycin	873	153,229	236,685	390,787
Escherichia coli	Aminoglycosides	234,921	4,697,760	6,264,488	11,197,169

Escherichia coli	Aminopenicillin	131,064	7,350,908	2,974,348	10,456,320
Escherichia coli	Beta Lactam/Beta-lactamase inhibitors	122,325	2,498,300	2,785,833	5,406,458
Escherichia coli	Carbapenems	146,209	13,715,305	5,378,639	19,240,153
Escherichia coli	Fluoroquinolones	124,081	12,417,183	5,145,708	17,686,972
Escherichia coli	Trimethoprim-Sulfamethoxazole	108,917	3,553,408	1,777,372	5,439,697
Escherichia coli	Third-generation cephalosporins	189,860	15,617,877	6,143,380	21,951,117
Group A *Streptococcus*	Macrolide	69	12,881	49,553	62,503
Group B *Streptococcus*	Fluoroquinolones	56	19,776	151,905	171,737
Group B *Streptococcus*	Macrolide	13	18,069	101,995	120,077
Group B *Streptococcus*	Penicillin	29	20,553	60,476	81,058
Haemophilus influenzae	Aminopenicillin	5,990	301,588	45,082	352,660
Haemophilus influenzae	Third-generation cephalosporins	1,207	482,695	140,139	624,041
Klebsiella pneumoniae	Aminoglycosides	90,181	1,909,497	1,887,761	3,887,439
Klebsiella pneumoniae	Beta Lactam/Beta-lactamase inhibitors	47,847	1,023,087	1,007,877	2,078,811
Klebsiella pneumoniae	Carbapenems	49,903	3,743,034	1,911,570	5,704,507
Klebsiella pneumoniae	Fluoroquinolones	51,555	2,903,454	1,626,375	4,581,384
Klebsiella pneumoniae	Trimethoprim-Sulfamethoxazole	33,189	1,047,833	576,830	1,657,852
Klebsiella pneumoniae	Third-generation cephalosporins	69,850	4,429,962	1,895,657	6,395,469
Morganella spp.	Fluoroquinolones	73	12,558	102,636	115,267
Morganella spp.	Fourth-generation cephalosporins	23	6,194	47,239	53,456
Morganella spp.	Third-generation cephalosporins	100	25,132	116,282	141,514
Neisseria gonorrhoeae	Fluoroquinolones	72	337,497	19,469	357,038
Neisseria gonorrhoeae	Third-generation cephalosporins	46	85,659	51,025	136,730
Proteus spp.	Aminoglycosides	8,190	214,294	575,025	797,509
Proteus spp.	Aminopenicillin	4,145	167,880	248,874	420,899
Proteus spp.	Fluoroquinolones	4,008	298,127	471,368	773,503
Proteus spp.	Trimethoprim-Sulfamethoxazole	4,097		251,404	255,501
Proteus spp.	Third-generation cephalosporins	4,918	477,727	492,298	974,943

Pseudomonas aeruginosa	Aminoglycosides	66,697	1,796,063	1,567,433	3,430,193
Pseudomonas aeruginosa	Anti-pseudomonal penicillin/Beta-Lactamase inhibitors	17,470	1,586,814	736,357	2,340,641
Pseudomonas aeruginosa	Carbapenems	28,967	1,905,361	1,397,400	3,331,728
Pseudomonas aeruginosa	Fluoroquinolones	39,556	1,793,144	1,219,550	3,052,250
Pseudomonas aeruginosa	Fourth-generation cephalosporins	10,325	877,629	548,141	1,436,095
Pseudomonas aeruginosa	Third-generation cephalosporins	39,713	1,108,211	1,021,384	2,169,308
Non-typhoidal Salmonellae	Fluoroquinolones	67,948	38,584	21,416	127,948
Salmonella Paratyphi	Fluoroquinolones	11,123		5,548	16,671
Salmonella Paratyphi	Multi-drug resistance	31,383	1,131	5,548	38,062
Salmonella Typhi	Fluoroquinolones	41,718	3,299	30,072	75,089
Salmonella Typhi	Multi-drug resistance	113,258	6,593	3,608	123,459
Serratia spp.	Aminoglycosides	15,926	192,825	196,432	405,183
Serratia spp.	Anti-pseudomonal penicillin/Beta-Lactamase inhibitors	9,974	286,092	69,809	365,875
Serratia spp.	Carbapenems	4,952	338,714	253,139	596,805
Serratia spp.	Fluoroquinolones	5,291	276,210	212,198	493,699
Serratia spp.	Fourth-generation cephalosporins	342	143,154	85,400	228,896
Serratia spp.	Third-generation cephalosporins	10,502	389,175	231,306	630,983
Shigella spp.	Fluoroquinolones	NA	26,170	10,166	36,336
Staphylococcus aureus	Fluoroquinolones	19,755	1,306,936	2,488,499	3,815,190
Staphylococcus aureus	Macrolide	7,342	1,200,571	1,404,602	2,612,515
Staphylococcus aureus	Methicillin	16,454	1,530,965	2,383,921	3,931,340
Staphylococcus aureus	Trimethoprim-Sulfamethoxazole	5,718	980,139	1,061,489	2,047,346
Staphylococcus aureus	Vancomycin	9,673	45,703,585	1,346,952	47,060,210
Streptococcus pneumoniae	Beta Lactam/Beta-lactamase inhibitors	1,029	1,060	185,823	187,912
Streptococcus pneumoniae	Carbapenems	7,094	286,986	294,658	588,738
Streptococcus pneumoniae	Fluoroquinolones	38,449	294,295	471,871	804,615
Streptococcus pneumoniae	Macrolide	27,301	255,955	460,238	743,494
Streptococcus pneumoniae	Penicillin	28,091	375,820	366,413	770,324

Streptococcus pneumoniae	Trimethoprim-Sulfamethoxazole	21,417	14,041	101,547	137,005
Streptococcus pneumoniae	Third-generation cephalosporins	28,950	571,347	554,388	1,154,685

Table S12: Global burden of AMR estimation hierarchy for syndromes, pathogens, and antibiotics

	Syndrome Type	Infectious Syndrome	Pathogen	Modelled Antibiotic classes
**Sepsis Mortality Envelope**	Has estimated bacterial etiologies	Infections of the skin and subcutaneous systems	***Acinetobacter baumannii***	Anti-pseudomonal penicillin/Beta-Lactamase inhibitors, Beta Lactam/Beta-lactamase inhibitors, Carbapenems, Fourth-generation cephalosporins, Third-generation cephalosporins, Aminoglycosides, Fluoroquinolones
			***Citrobacter* spp.**	Aminoglycosides, Anti-pseudomonal penicillin/Beta-Lactamase inhibitors, Carbapenems, Fluoroquinolones, Fourth-generation cephalosporins, Third-generation cephalosporins
			***Enterobacter* spp.**	Aminoglycosides, Anti-pseudomonal penicillin/Beta-Lactamase inhibitors, Carbapenems, Fluoroquinolones, Fourth-generation cephalosporins, Trimethoprim-Sulfamethoxazole
			Enterococcus faecalis	Fluoroquinolones, Vancomycin
			Enterococcus faecium	Fluoroquinolones, Vancomycin
			Escherichia coli	Aminoglycosides, Aminopenicillin, Beta Lactam/Beta-lactamase inhibitors, Carbapenems, Trimethoprim-Sulfamethoxazole, Fluoroquinolones, Third-generation cephalosporins
			**Group A *Streptococcus***	Macrolide
			**Group B *Streptococcus***	Fluoroquinolones, Macrolide, Penicillin
			Haemophilus influenzae	Aminopenicillin, Third-generation cephalosporins
			Klebsiella pneumoniae	Aminoglycosides, Beta Lactam/Beta-lactamase inhibitors, Fluoroquinolones, Trimethoprim-Sulfamethoxazole, Carbapenems, Third-generation cephalosporins
			***Morganella* spp.**	Fluoroquinolones, Fourth-generation cephalosporins, Third-generation cephalosporins
			***Proteus* spp.**	Aminoglycosides, Aminopenicillin, Fluoroquinolones, Trimethoprim-Sulfamethoxazole, Third-generation cephalosporins
			Pseudomonas aeruginosa	Aminoglycosides, Anti-pseudomonal penicillin/Beta-Lactamase inhibitors, Carbapenems, Fluoroquinolones, Fourth-generation cephalosporins, Third-generation cephalosporins
			***Serratia* spp.**	Aminoglycosides, Anti-pseudomonal penicillin/Beta-Lactamase inhibitors, Carbapenems, Fluoroquinolones, Fourth-generation cephalosporins, Third-generation cephalosporins
			Staphylococcus aureus	Fluoroquinolones, Macrolide, Trimethoprim-Sulfamethoxazole, Methicillin, Vancomycin
			Streptococcus pneumoniae	Beta Lactam/Beta-lactamase inhibitors, Carbapenems, Fluoroquinolones, Macrolide, Trimethoprim-Sulfamethoxazole, Third-generation cephalosporins, Penicillin
			Aeromonas spp.	No antibiotics modelled
			Burkholderia spp.	No antibiotics modelled
			Entamoeba histolytica	No antibiotics modelled
			Gram-negative others	No antibiotics modelled
			Gram-positive others	No antibiotics modelled
			Listeria spp.	No antibiotics modelled
			Non-polio enteroviruses	No antibiotics modelled
			Other *Acinetobacter* species	No antibiotics modelled
			Other *Clostridiodes* species	No antibiotics modelled
			Other *enterococci*	No antibiotics modelled
			Other *Klebsiella* species	No antibiotics modelled

			Other *Mycobacterium* species	No antibiotics modelled
			Other *Pseudomonas* species	No antibiotics modelled
			Other *Streptococcus* species	No antibiotics modelled
			Other viruses	No antibiotics modelled
			Actinomyces spp.	No antibiotics modelled
		Bloodstream infections	***Acinetobacter baumannii***	Anti-pseudomonal penicillin/Beta-Lactamase inhibitors, Beta Lactam/Beta-lactamase inhibitors, Carbapenems, Fourth-generation cephalosporins, Third-generation cephalosporins, Aminoglycosides, Fluoroquinolones
			***Citrobacter* spp.**	Aminoglycosides, Anti-pseudomonal penicillin/Beta-Lactamase inhibitors, Carbapenems, Fluoroquinolones, Fourth-generation cephalosporins, Third-generation cephalosporins
			***Enterobacter* spp.**	Aminoglycosides, Anti-pseudomonal penicillin/Beta-Lactamase inhibitors, Carbapenems, Fluoroquinolones, Fourth-generation cephalosporins, Trimethoprim-Sulfamethoxazole
			Enterococcus faecalis	Fluoroquinolones, Vancomycin
			Enterococcus faecium	Fluoroquinolones, Vancomycin
			Escherichia coli	Aminoglycosides, Aminopenicillin, Beta Lactam/Beta-lactamase inhibitors, Carbapenems, Trimethoprim-Sulfamethoxazole, Fluoroquinolones, Third-generation cephalosporins
			**Group A *Streptococcus***	Macrolide
			**Group B *Streptococcus***	Fluoroquinolones, Macrolide, Penicillin
			Haemophilus influenzae	Aminopenicillin, Third-generation cephalosporins
			Klebsiella pneumoniae	Aminoglycosides, Beta Lactam/Beta-lactamase inhibitors, Fluoroquinolones, Trimethoprim-Sulfamethoxazole, Carbapenems, Third-generation cephalosporins
			***Morganella* spp.**	Fluoroquinolones, Fourth-generation cephalosporins, Third-generation cephalosporins
			***Proteus* spp.**	Aminoglycosides, Aminopenicillin, Fluoroquinolones, Trimethoprim-Sulfamethoxazole, Third-generation cephalosporins
			Pseudomonas aeruginosa	Aminoglycosides, Anti-pseudomonal penicillin/Beta-Lactamase inhibitors, Carbapenems, Fluoroquinolones, Fourth-generation cephalosporins, Third-generation cephalosporins
			***Serratia* spp.**	Aminoglycosides, Anti-pseudomonal penicillin/Beta-Lactamase inhibitors, Carbapenems, Fluoroquinolones, Fourth-generation cephalosporins, Third-generation cephalosporins
			Staphylococcus aureus	Fluoroquinolones, Macrolide, Trimethoprim-Sulfamethoxazole, Methicillin, Vancomycin
			Streptococcus pneumoniae	Beta Lactam/Beta-lactamase inhibitors, Carbapenems, Fluoroquinolones, Macrolide, Trimethoprim-Sulfamethoxazole, Third-generation cephalosporins, Penicillin
			Actinomyces spp.	No antibiotics modelled
			Aeromonas spp.	No antibiotics modelled
			Aspergillus spp.	No antibiotics modelled
			Burkholderia spp.	No antibiotics modelled
			Candida spp.	No antibiotics modelled
			Cryptococcus spp.	No antibiotics modelled
			Gram-negative others	No antibiotics modelled
			Gram-positive others	No antibiotics modelled
			Histoplasma spp.	No antibiotics modelled
			Leptospira spp.	No antibiotics modelled

			Listeria spp.	No antibiotics modelled
			Neisseria meningitidis	No antibiotics modelled
			Non-polio enteroviruses	No antibiotics modelled
			Other *Acinetobacter* species	No antibiotics modelled
			Other *enterococci*	No antibiotics modelled
			Other fungi	No antibiotics modelled
			Other *Klebsiella* species	No antibiotics modelled
			Other *Mycobacterium* species	No antibiotics modelled
			Other *Pseudomonas* species	No antibiotics modelled
			Other *Streptococcus* species	No antibiotics modelled
			Other viruses	No antibiotics modelled
		Diarrhoea	**Enteropathogenic *Escherichia coli***	Aminoglycosides, Aminopenicillin, Beta Lactam/Beta-lactamase inhibitors, Carbapenems, Trimethoprim-Sulfamethoxazole, Fluoroquinolones, Third-generation cephalosporins
			**Enterotoxigenic *Escherichia coli***	Aminoglycosides, Aminopenicillin, Beta Lactam/Beta-lactamase inhibitors, Carbapenems, Trimethoprim-Sulfamethoxazole, Fluoroquinolones, Third-generation cephalosporins
			Invasive non-typhoidal Salmonella	Fluoroquinolones
			***Shigella* spp.**	Fluoroquinolones
			Adenovirus	No antibiotics modelled
			Aeromonas spp.	No antibiotics modelled
			Campylobacter spp.	No antibiotics modelled
			Clostridioides difficile	No antibiotics modelled
			Cryptosporidium spp.	No antibiotics modelled
			Entamoeba histolytica	No antibiotics modelled
			Norovirus	No antibiotics modelled
			Other diarrhoeal pathogens	No antibiotics modelled
			Rotavirus	No antibiotics modelled
			Vibrio cholerae	No antibiotics modelled
		Endocarditis	***Acinetobacter baumannii***	Anti-pseudomonal penicillin/Beta-Lactamase inhibitors, Beta Lactam/Beta-lactamase inhibitors, Carbapenems, Fourth-generation cephalosporins, Third-generation cephalosporins, Aminoglycosides, Fluoroquinolones
			***Citrobacter* spp.**	Aminoglycosides, Anti-pseudomonal penicillin/Beta-Lactamase inhibitors, Carbapenems, Fluoroquinolones, Fourth-generation cephalosporins, Third-generation cephalosporins
			***Enterobacter* spp.**	Aminoglycosides, Anti-pseudomonal penicillin/Beta-Lactamase inhibitors, Carbapenems, Fluoroquinolones, Fourth-generation cephalosporins, Trimethoprim-Sulfamethoxazole
			Enterococcus faecalis	Fluoroquinolones, Vancomycin
			Enterococcus faecium	Fluoroquinolones, Vancomycin
			Escherichia coli	Aminoglycosides, Aminopenicillin, Beta Lactam/Beta-lactamase inhibitors, Carbapenems, Trimethoprim-Sulfamethoxazole, Fluoroquinolones, Third-generation cephalosporins

		**Group A *Streptococcus***	Macrolide
		Klebsiella pneumoniae	Aminoglycosides, Beta Lactam/Beta-lactamase inhibitors, Fluoroquinolones, Trimethoprim-Sulfamethoxazole, Carbapenems, Third-generation cephalosporins
		***Morganella* spp.**	Fluoroquinolones, Fourth-generation cephalosporins, Third-generation cephalosporins
		***Proteus* spp.**	Aminoglycosides, Aminopenicillin, Fluoroquinolones, Trimethoprim-Sulfamethoxazole, Third-generation cephalosporins
		Pseudomonas aeruginosa	Aminoglycosides, Anti-pseudomonal penicillin/Beta-Lactamase inhibitors, Carbapenems, Fluoroquinolones, Fourth-generation cephalosporins, Third-generation cephalosporins
		***Serratia* spp.**	Aminoglycosides, Anti-pseudomonal penicillin/Beta-Lactamase inhibitors, Carbapenems, Fluoroquinolones, Fourth-generation cephalosporins, Third-generation cephalosporins
		Staphylococcus aureus	Fluoroquinolones, Macrolide, Trimethoprim-Sulfamethoxazole, Methicillin, Vancomycin
		Streptococcus pneumoniae	Beta Lactam/Beta-lactamase inhibitors, Carbapenems, Fluoroquinolones, Macrolide, Trimethoprim-Sulfamethoxazole, Third-generation cephalosporins, Penicillin
		Candida spp.	No antibiotics modelled
		Gram-negative others	No antibiotics modelled
		Neisseria meningitidis	No antibiotics modelled
		Other *Klebsiella* species	No antibiotics modelled
		Other *Streptococcus* species	No antibiotics modelled
		Toxoplasma spp.	No antibiotics modelled
	Infections of bones, joints, and related organs	***Acinetobacter baumannii***	Anti-pseudomonal penicillin/Beta-Lactamase inhibitors, Beta Lactam/Beta-lactamase inhibitors, Carbapenems, Fourth-generation cephalosporins, Third-generation cephalosporins, Aminoglycosides, Fluoroquinolones
		***Citrobacter* spp.**	Aminoglycosides, Anti-pseudomonal penicillin/Beta-Lactamase inhibitors, Carbapenems, Fluoroquinolones, Fourth-generation cephalosporins, Third-generation cephalosporins
		***Enterobacter* spp.**	Aminoglycosides, Anti-pseudomonal penicillin/Beta-Lactamase inhibitors, Carbapenems, Fluoroquinolones, Fourth-generation cephalosporins, Trimethoprim-Sulfamethoxazole
		Enterococcus faecalis	Fluoroquinolones, Vancomycin
		Enterococcus faecium	Fluoroquinolones, Vancomycin
		Escherichia coli	Aminoglycosides, Aminopenicillin, Beta Lactam/Beta-lactamase inhibitors, Carbapenems, Trimethoprim-Sulfamethoxazole, Fluoroquinolones, Third-generation cephalosporins
		**Group A *Streptococcus***	Macrolide
		**Group B *Streptococcus***	Fluoroquinolones, Macrolide, Penicillin
		Haemophilus influenzae	Aminopenicillin, Third-generation cephalosporins
		Klebsiella pneumoniae	Aminoglycosides, Beta Lactam/Beta-lactamase inhibitors, Fluoroquinolones, Trimethoprim-Sulfamethoxazole, Carbapenems, Third-generation cephalosporins
		***Morganella* spp.**	Fluoroquinolones, Fourth-generation cephalosporins, Third-generation cephalosporins
		***Proteus* spp.**	Aminoglycosides, Aminopenicillin, Fluoroquinolones, Trimethoprim-Sulfamethoxazole, Third-generation cephalosporins
		Pseudomonas aeruginosa	Aminoglycosides, Anti-pseudomonal penicillin/Beta-Lactamase inhibitors, Carbapenems, Fluoroquinolones, Fourth-generation cephalosporins, Third-generation cephalosporins
		***Serratia* spp.**	Aminoglycosides, Anti-pseudomonal penicillin/Beta-Lactamase inhibitors, Carbapenems, Fluoroquinolones, Fourth-generation cephalosporins, Third-generation cephalosporins
		Staphylococcus aureus	Fluoroquinolones, Macrolide, Trimethoprim-Sulfamethoxazole, Methicillin, Vancomycin

			Streptococcus pneumoniae	Beta Lactam/Beta-lactamase inhibitors, Carbapenems, Fluoroquinolones, Macrolide, Trimethoprim-Sulfamethoxazole, Third-generation cephalosporins, Penicillin
			Aeromonas spp.	No antibiotics modelled
			Burkholderia spp.	No antibiotics modelled
			Candida spp.	No antibiotics modelled
			Chlamydia spp.	No antibiotics modelled
			Gram-negative others	No antibiotics modelled
			Gram-positive others	No antibiotics modelled
			Neisseria meningitidis	No antibiotics modelled
			Other *Acinetobacter* species	No antibiotics modelled
			Other *enterococci*	No antibiotics modelled
			Other fungi	No antibiotics modelled
			Other *Klebsiella* species	No antibiotics modelled
			Other *Mycobacterium* species	No antibiotics modelled
			Other *Pseudomonas* species	No antibiotics modelled
			Other *Streptococcus* species	No antibiotics modelled
			Other viruses	No antibiotics modelled
		Lower respiratory infections	***Acinetobacter baumannii***	Anti-pseudomonal penicillin/Beta-Lactamase inhibitors, Beta Lactam/Beta-lactamase inhibitors, Carbapenems, Fourth-generation cephalosporins, Third-generation cephalosporins, Aminoglycosides, Fluoroquinolones
			***Citrobacter* spp.**	Aminoglycosides, Anti-pseudomonal penicillin/Beta-Lactamase inhibitors, Carbapenems, Fluoroquinolones, Fourth-generation cephalosporins, Third-generation cephalosporins
			***Enterobacter* spp.**	Aminoglycosides, Anti-pseudomonal penicillin/Beta-Lactamase inhibitors, Carbapenems, Fluoroquinolones, Fourth-generation cephalosporins, Trimethoprim-Sulfamethoxazole
			Enterococcus faecalis	Fluoroquinolones, Vancomycin
			Enterococcus faecium	Fluoroquinolones, Vancomycin
			Escherichia coli	Aminoglycosides, Aminopenicillin, Beta Lactam/Beta-lactamase inhibitors, Carbapenems, Trimethoprim-Sulfamethoxazole, Fluoroquinolones, Third-generation cephalosporins
			**Group A *Streptococcus***	Macrolide
			**Group B *Streptococcus***	Fluoroquinolones, Macrolide, Penicillin
			Haemophilus influenzae	Aminopenicillin, Third-generation cephalosporins
			Klebsiella pneumoniae	Aminoglycosides, Beta Lactam/Beta-lactamase inhibitors, Fluoroquinolones, Trimethoprim-Sulfamethoxazole, Carbapenems, Third-generation cephalosporins
			***Morganella* spp.**	Fluoroquinolones, Fourth-generation cephalosporins, Third-generation cephalosporins
			***Proteus* spp.**	Aminoglycosides, Aminopenicillin, Fluoroquinolones, Trimethoprim-Sulfamethoxazole, Third-generation cephalosporins
			Pseudomonas aeruginosa	Aminoglycosides, Anti-pseudomonal penicillin/Beta-Lactamase inhibitors, Carbapenems, Fluoroquinolones, Fourth-generation cephalosporins, Third-generation cephalosporins
			***Serratia* spp.**	Aminoglycosides, Anti-pseudomonal penicillin/Beta-Lactamase inhibitors, Carbapenems, Fluoroquinolones, Fourth-generation cephalosporins, Third-generation cephalosporins

			Staphylococcus aureus	Fluoroquinolones, Macrolide, Trimethoprim-Sulfamethoxazole, Methicillin, Vancomycin
			Streptococcus pneumoniae	Beta Lactam/Beta-lactamase inhibitors, Carbapenems, Fluoroquinolones, Macrolide, Trimethoprim-Sulfamethoxazole, Third-generation cephalosporins, Penicillin
			Actinomyces spp.	No antibiotics modelled
			Aeromonas spp.	No antibiotics modelled
			Aspergillus spp.	No antibiotics modelled
			Burkholderia spp.	No antibiotics modelled
			Chlamydia spp.	No antibiotics modelled
			Cryptococcus spp.	No antibiotics modelled
			Cytomegalovirus	No antibiotics modelled
			Gram-negative others	No antibiotics modelled
			Gram-positive others	No antibiotics modelled
			Histoplasma spp.	No antibiotics modelled
			Influenza virus	No antibiotics modelled
			Legionella spp.	No antibiotics modelled
			Mycoplasma spp.	No antibiotics modelled
			Non-polio enteroviruses	No antibiotics modelled
			Other *Acinetobacter* species	No antibiotics modelled
			Other fungi	No antibiotics modelled
			Other *Klebsiella* species	No antibiotics modelled
			Other *Mycobacterium* species	No antibiotics modelled
			Other *Pseudomonas* species	No antibiotics modelled
			Other *Streptococcus* species	No antibiotics modelled
			Other viruses	No antibiotics modelled
			Respiratory syncytial virus	No antibiotics modelled
		Meningitis	***Acinetobacter baumannii***	Anti-pseudomonal penicillin/Beta-Lactamase inhibitors, Beta Lactam/Beta-lactamase inhibitors, Carbapenems, Fourth-generation cephalosporins, Third-generation cephalosporins, Aminoglycosides, Fluoroquinolones
			***Citrobacter* spp.**	Aminoglycosides, Anti-pseudomonal penicillin/Beta-Lactamase inhibitors, Carbapenems, Fluoroquinolones, Fourth-generation cephalosporins, Third-generation cephalosporins
			***Enterobacter* spp.**	Aminoglycosides, Anti-pseudomonal penicillin/Beta-Lactamase inhibitors, Carbapenems, Fluoroquinolones, Fourth-generation cephalosporins, Trimethoprim-Sulfamethoxazole
			Enterococcus faecalis	Fluoroquinolones, Vancomycin
			Enterococcus faecium	Fluoroquinolones, Vancomycin
			Escherichia coli	Aminoglycosides, Aminopenicillin, Beta Lactam/Beta-lactamase inhibitors, Carbapenems, Trimethoprim-Sulfamethoxazole, Fluoroquinolones, Third-generation cephalosporins
			**Group A *Streptococcus***	Macrolide
			**Group B *Streptococcus***	Fluoroquinolones, Macrolide, Penicillin
			Haemophilus influenzae	Aminopenicillin, Third-generation cephalosporins

			Klebsiella pneumoniae	Aminoglycosides, Beta Lactam/Beta-lactamase inhibitors, Fluoroquinolones, Trimethoprim-Sulfamethoxazole, Carbapenems, Third-generation cephalosporins
			***Morganella* spp.**	Fluoroquinolones, Fourth-generation cephalosporins, Third-generation cephalosporins
			***Proteus* spp.**	Aminoglycosides, Aminopenicillin, Fluoroquinolones, Trimethoprim-Sulfamethoxazole, Third-generation cephalosporins
			Pseudomonas aeruginosa	Aminoglycosides, Anti-pseudomonal penicillin/Beta-Lactamase inhibitors, Carbapenems, Fluoroquinolones, Fourth-generation cephalosporins, Third-generation cephalosporins
			***Serratia* spp.**	Aminoglycosides, Anti-pseudomonal penicillin/Beta-Lactamase inhibitors, Carbapenems, Fluoroquinolones, Fourth-generation cephalosporins, Third-generation cephalosporins
			Staphylococcus aureus	Fluoroquinolones, Macrolide, Trimethoprim-Sulfamethoxazole, Methicillin, Vancomycin
			Streptococcus pneumoniae	Beta Lactam/Beta-lactamase inhibitors, Carbapenems, Fluoroquinolones, Macrolide, Trimethoprim-Sulfamethoxazole, Third-generation cephalosporins, Penicillin
			Adenovirus	No antibiotics modelled
			Burkholderia spp.	No antibiotics modelled
			Candida spp.	No antibiotics modelled
			Coagulase-negative *Staphylococcus*	No antibiotics modelled
			Gram-negative others	No antibiotics modelled
			Gram-positive others	No antibiotics modelled
			Listeria spp.	No antibiotics modelled
			Mumps	No antibiotics modelled
			Neisseria meningitidis	No antibiotics modelled
			Non-polio enteroviruses	No antibiotics modelled
			Other *Acinetobacter* species	No antibiotics modelled
			Other fungi	No antibiotics modelled
			Other *Klebsiella* species	No antibiotics modelled
			Other *Pseudomonas* species	No antibiotics modelled
			Other *Streptococcus* species	No antibiotics modelled
			Other viruses	No antibiotics modelled
		Peritoneal and intra-abdominal infections	***Acinetobacter baumannii***	Anti-pseudomonal penicillin/Beta-Lactamase inhibitors, Beta Lactam/Beta-lactamase inhibitors, Carbapenems, Fourth-generation cephalosporins, Third-generation cephalosporins, Aminoglycosides, Fluoroquinolones
			***Citrobacter* spp.**	Aminoglycosides, Anti-pseudomonal penicillin/Beta-Lactamase inhibitors, Carbapenems, Fluoroquinolones, Fourth-generation cephalosporins, Third-generation cephalosporins
			***Enterobacter* spp.**	Aminoglycosides, Anti-pseudomonal penicillin/Beta-Lactamase inhibitors, Carbapenems, Fluoroquinolones, Fourth-generation cephalosporins, Trimethoprim-Sulfamethoxazole
			Enterococcus faecalis	Fluoroquinolones, Vancomycin
			Enterococcus faecium	Fluoroquinolones, Vancomycin
			Escherichia coli	Aminoglycosides, Aminopenicillin, Beta Lactam/Beta-lactamase inhibitors, Carbapenems, Trimethoprim-Sulfamethoxazole, Fluoroquinolones, Third-generation cephalosporins
			**Group A *Streptococcus***	Macrolide

			**Group B *Streptococcus***	Fluoroquinolones, Macrolide, Penicillin
			Klebsiella pneumoniae	Aminoglycosides, Beta Lactam/Beta-lactamase inhibitors, Fluoroquinolones, Trimethoprim-Sulfamethoxazole, Carbapenems, Third-generation cephalosporins
			***Morganella* spp.**	Fluoroquinolones, Fourth-generation cephalosporins, Third-generation cephalosporins
			***Proteus* spp.**	Aminoglycosides, Aminopenicillin, Fluoroquinolones, Trimethoprim-Sulfamethoxazole, Third-generation cephalosporins
			Pseudomonas aeruginosa	Aminoglycosides, Anti-pseudomonal penicillin/Beta-Lactamase inhibitors, Carbapenems, Fluoroquinolones, Fourth-generation cephalosporins, Third-generation cephalosporins
			***Serratia* spp.**	Aminoglycosides, Anti-pseudomonal penicillin/Beta-Lactamase inhibitors, Carbapenems, Fluoroquinolones, Fourth-generation cephalosporins, Third-generation cephalosporins
			Staphylococcus aureus	Fluoroquinolones, Macrolide, Trimethoprim-Sulfamethoxazole, Methicillin, Vancomycin
			Streptococcus pneumoniae	Beta Lactam/Beta-lactamase inhibitors, Carbapenems, Fluoroquinolones, Macrolide, Trimethoprim-Sulfamethoxazole, Third-generation cephalosporins, Penicillin
			Actinomyces spp.	No antibiotics modelled
			Aeromonas spp.	No antibiotics modelled
			Burkholderia spp.	No antibiotics modelled
			Candida spp.	No antibiotics modelled
			Chlamydia spp.	No antibiotics modelled
			Entamoeba histolytica	No antibiotics modelled
			Gram-negative others	No antibiotics modelled
			Gram-positive others	No antibiotics modelled
			Other *Acinetobacter* species	No antibiotics modelled
			Other *enterococci*	No antibiotics modelled
			Other *Klebsiella* species	No antibiotics modelled
			Other *Pseudomonas* species	No antibiotics modelled
			Other *Streptococcus* species	No antibiotics modelled
		Sexually transmitted infections	***Neisseria gonorrhoeae***	Third-generation cephalosporins, Fluoroquinolones
			Chlamydia spp.	No antibiotics modelled
			Treponema pallidum	No antibiotics modelled
		Tuberculosis	***Mycobacterium tuberculosis***	Multi-drug resistance, Extensive drug resistance
		Typhoid, paratyphoid, and invasive non-Typhoidal Salmonella	***Invasive non-typhoidal Salmonella***	Fluoroquinolones
			Salmonella Paratyphi	Fluoroquinolones, Multi-drug resistance
			Salmonella Typhi	Fluoroquinolones, Multi-drug resistance
		Urinary tract infections and pyelonephritis	***Acinetobacter baumannii***	Anti-pseudomonal penicillin/Beta-Lactamase inhibitors, Beta Lactam/Beta-lactamase inhibitors, Carbapenems, Fourth-generation cephalosporins, Third-generation cephalosporins, Aminoglycosides, Fluoroquinolones
			***Citrobacter* spp.**	Aminoglycosides, Anti-pseudomonal penicillin/Beta-Lactamase inhibitors, Carbapenems, Fluoroquinolones, Fourth-generation cephalosporins, Third-generation cephalosporins

			***Enterobacter* spp.**	Aminoglycosides, Anti-pseudomonal penicillin/Beta-Lactamase inhibitors, Carbapenems, Fluoroquinolones, Fourth-generation cephalosporins, Trimethoprim-Sulfamethoxazole
			Enterococcus faecalis	Fluoroquinolones, Vancomycin
			Enterococcus faecium	Fluoroquinolones, Vancomycin
			Escherichia coli	Aminoglycosides, Aminopenicillin, Beta Lactam/Beta-lactamase inhibitors, Carbapenems, Trimethoprim-Sulfamethoxazole, Fluoroquinolones, Third-generation cephalosporins
			**Group A *Streptococcus***	Macrolide
			**Group B *Streptococcus***	Fluoroquinolones, Macrolide, Penicillin
			Haemophilus influenzae	Aminopenicillin, Third-generation cephalosporins
			Klebsiella pneumoniae	Aminoglycosides, Beta Lactam/Beta-lactamase inhibitors, Fluoroquinolones, Trimethoprim-Sulfamethoxazole, Carbapenems, Third-generation cephalosporins
			***Morganella* spp.**	Fluoroquinolones, Fourth-generation cephalosporins, Third-generation cephalosporins
			***Proteus* spp.**	Aminoglycosides, Aminopenicillin, Fluoroquinolones, Trimethoprim-Sulfamethoxazole, Third-generation cephalosporins
			Pseudomonas aeruginosa	Aminoglycosides, Anti-pseudomonal penicillin/Beta-Lactamase inhibitors, Carbapenems, Fluoroquinolones, Fourth-generation cephalosporins, Third-generation cephalosporins
			***Serratia* spp.**	Aminoglycosides, Anti-pseudomonal penicillin/Beta-Lactamase inhibitors, Carbapenems, Fluoroquinolones, Fourth-generation cephalosporins, Third-generation cephalosporins
			Staphylococcus aureus	Fluoroquinolones, Macrolide, Trimethoprim-Sulfamethoxazole, Methicillin, Vancomycin
			Streptococcus pneumoniae	Beta Lactam/Beta-lactamase inhibitors, Carbapenems, Fluoroquinolones, Macrolide, Trimethoprim-Sulfamethoxazole, Third-generation cephalosporins, Penicillin
			Aeromonas spp.	No antibiotics modelled
			Aspergillus spp.	No antibiotics modelled
			Burkholderia spp.	No antibiotics modelled
			Candida spp.	No antibiotics modelled
			Coagulase-negative *Staphylococcus*	No antibiotics modelled
			Gram-negative others	No antibiotics modelled
			Mycoplasma spp.	No antibiotics modelled
			Other *Acinetobacter* species	No antibiotics modelled
			Other *enterococci*	No antibiotics modelled
			Other fungi	No antibiotics modelled
			Other *Klebsiella* species	No antibiotics modelled
			Other *Pseudomonas* species	No antibiotics modelled
			Other *Streptococcus* species	No antibiotics modelled
	Does not have estimated bacterial etiologies	Encephalitis	Not modelled	No antibiotics modelled
		Eye infections	Not modelled	No antibiotics modelled
		Carditis, myocarditis, and pericarditis	Unspecified viruses	No antibiotics modelled
		Genital infections	Not modelled	No antibiotics modelled

		Hepatitis	Not modelled	No antibiotics modelled
		Myelitis, meningoencephalitis, and other central nervous system infections	Not modelled	No antibiotics modelled
		Oral infections	Not modelled	No antibiotics modelled
		Other parasitic infections	Not modelled	No antibiotics modelled
		Unspecified site infections	Not modelled	No antibiotics modelled
		Upper respiratory infections	Not modelled	No antibiotics modelled

Table S13: 82 fatal pathogen-drug combination ranking by burden attributable to AMR in 1990, globally and by super-region

Location	Year	Pathogen	Antibiotic Class	Rank	Attributable AMR Deaths
Global	1990	*Streptococcus pneumoniae*	Carbapenems	1	107,000 (61,800-151,000)
Global	1990	*Staphylococcus aureus*	Methicillin	2	57,200 (34,100-80,300)
Global	1990	*Acinetobacter baumannii*	Carbapenems	3	51,300 (34,100-68,600)
Global	1990	*Streptococcus pneumoniae*	Fluoroquinolones	4	45,300 (20,500-70,100)
Global	1990	*Klebsiella pneumoniae*	3GC	5	31,600 (20,000-43,200)
Global	1990	*Acinetobacter baumannii*	Fluoroquinolones	6	31,200 (25,300-37,200)
Global	1990	*Pseudomonas aeruginosa*	Carbapenems	7	30,200 (18,000-42,500)
Global	1990	*Streptococcus pneumoniae*	TMP-SMX	8	29,200 (0-62,300)
Global	1990	*Klebsiella pneumoniae*	Fluoroquinolones	9	29,200 (20,200-38,100)
Global	1990	*Acinetobacter baumannii*	Anti-pseudomonal	10	29,100 (23,600-34,600)
Global	1990	*Escherichia coli*	TMP-SMX	11	28,500 (19,300-37,700)
Global	1990	*Klebsiella pneumoniae*	Aminoglycosides	12	28,500 (20,400-36,600)
Global	1990	*Streptococcus pneumoniae*	Penicillin	13	25,900 (18,400-33,400)
Global	1990	*Acinetobacter baumannii*	Aminoglycosides	14	24,600 (18,300-31,000)
Global	1990	*Pseudomonas aeruginosa*	Fluoroquinolones	15	24,300 (17,200-31,500)
Global	1990	*Escherichia coli*	Beta-Lactams/Beta-Lactamase Inhib.	16	24,200 (4,690-43,600)
Global	1990	*Streptococcus pneumoniae*	3GC	17	23,900 (17,100-30,800)
Global	1990	*Klebsiella pneumoniae*	TMP-SMX	18	22,500 (11,500-33,500)
Global	1990	*Escherichia coli*	3GC	19	21,700 (8,580-34,900)
Global	1990	*Pseudomonas aeruginosa*	Anti-pseudomonal	20	21,000 (16,100-25,800)
Global	1990	*Klebsiella pneumoniae*	Beta-Lactams/Beta-Lactamase Inhib.	21	19,700 (9,630-29,900)
Global	1990	*Klebsiella pneumoniae*	Carbapenems	22	18,100 (13,300-22,900)
Global	1990	*Pseudomonas aeruginosa*	Aminoglycosides	23	17,000 (10,800-23,200)
Global	1990	*Escherichia coli*	Aminoglycosides	24	16,100 (7,240-24,900)
Global	1990	*Streptococcus pneumoniae*	Macrolides	25	15,400 (8,940-21,800)
Global	1990	*Staphylococcus aureus*	Fluoroquinolones	26	15,100 (5,660-24,500)
Global	1990	*Escherichia coli*	Fluoroquinolones	27	14,100 (8,100-20,200)
Global	1990	*Staphylococcus aureus*	TMP-SMX	28	13,300 (8,010-18,500)
Global	1990	*Escherichia coli*	Carbapenems	29	11,900 (3,280-20,600)
Global	1990	*Staphylococcus aureus*	Macrolides	30	11,500 (7,510-15,500)
Global	1990	*Streptococcus pneumoniae*	Beta-Lactams/Beta-Lactamase Inhib.	31	11,300 (6,950-15,600)
Global	1990	*Escherichia coli*	Aminopenicillin	32	10,400 (5,650-15,200)
Global	1990	*Enterobacter* spp.	Carbapenems	33	9,660 (7,290-12,000)
Global	1990	*Salmonella* Typhi	Fluoroquinolones	34	8,690 (1,220-16,200)
Global	1990	*Acinetobacter baumannii*	3GC	35	7,990 (6,660-9,320)
Global	1990	*Enterobacter* spp.	Anti-pseudomonal	36	7,900 (6,340-9,460)
Global	1990	*Serratia* spp.	Anti-pseudomonal	37	7,760 (6,000-9,530)
Global	1990	*Haemophilus influenzae*	3GC	38	7,470 (413-14,500)
Global	1990	*Enterobacter faecalis*	Fluoroquinolones	39	7,290 (4,810-9,760)
Global	1990	*Shigella* spp.	Fluoroquinolones	40	6,710 (1,190-12,200)
Global	1990	*Serratia* spp.	Aminoglycosides	41	6,660 (4,610-8,710)
Global	1990	*Staphylococcus aureus*	Vancomycin	42	6,250 (2,800-9,710)
Global	1990	*Enterococcus faecium*	Fluoroquinolones	43	6,250 (3,070-9,430)
Global	1990	*Acinetobacter baumannii*	4GC	44	6,130 (4,880-7,370)
Global	1990	*Enterobacter* spp.	Aminoglycosides	45	6,010 (4,110-7,920)
Global	1990	*Haemophilus influenzae*	Aminopenicillin	46	5,450 (4,410-6,500)
Global	1990	*Mycobacterium tuberculosis*	MDR excluding XDR	47	5,300 (0-16,100)
Global	1990	*Pseudomonas aeruginosa*	4GC	48	5,020 (3,070-6,970)
Global	1990	*Salmonella* Typhi	MDR	49	4,880 (648-9,120)
Global	1990	*Acinetobacter baumannii*	Beta-Lactams/Beta-Lactamase Inhib.	50	4,830 (2,870-6,780)
Global	1990	*Serratia* spp.	4GC	51	4,770 (4,040-5,500)
Global	1990	*Enterobacter* spp.	Fluoroquinolones	52	4,750 (2,730-6,770)
Global	1990	*Enterobacter* spp.	4GC	53	4,340 (3,620-5,060)

Global	1990	*Serratia* spp.	Fluoroquinolones	54	4,040 (1,720-6,370)
Global	1990	*Enterococcus faecium*	Vancomycin	55	4,020 (3,070-4,970)
Global	1990	*Citrobacter* spp.	Anti-pseudomonal	56	3,600 (2,900-4,310)
Global	1990	Group B *Streptococcus*	Fluoroquinolones	57	3,350 (1,660-5,040)
Global	1990	*Proteus* spp.	3GC	58	3,210 (1,850-4,580)
Global	1990	*Serratia* spp.	Carbapenems	59	3,190 (2,420-3,960)
Global	1990	*Citrobacter* spp.	Fluoroquinolones	60	3,000 (1,700-4,310)
Global	1990	*Pseudomonas aeruginosa*	3GC	61	2,650 (612-4,680)
Global	1990	*Morganella* spp.	Fluoroquinolones	62	2,600 (1,360-3,840)
Global	1990	*Morganella* spp.	4GC	63	2,580 (1,740-3,420)
Global	1990	Group B *Streptococcus*	Macrolides	64	2,510 (1,580-3,430)
Global	1990	Group B *Streptococcus*	Penicillin	65	2,430 (436-4,430)
Global	1990	*Morganella* spp.	3GC	66	2,330 (1,680-2,980)
Global	1990	*Enterobacter* spp.	TMP-SMX	67	2,290 (363-4,210)
Global	1990	*Non-typhoidal Salmonella*	Fluoroquinolones	68	2,270 (0-5,020)
Global	1990	*Citrobacter* spp.	Carbapenems	69	2,240 (1,090-3,390)
Global	1990	Group A *Streptococcus*	Macrolides	70	2,220 (1,380-3,060)
Global	1990	*Proteus* spp.	Aminopenicillin	71	2,210 (1,700-2,720)
Global	1990	*Salmonella* Paratyphi	MDR	72	2,190 (159-4,230)
Global	1990	*Enterobacter faecalis*	Vancomycin	73	2,060 (0-4,730)
Global	1990	*Proteus* spp.	Aminoglycosides	74	1,810 (1,140-2,470)
Global	1990	*Salmonella* Paratyphi	Fluoroquinolones	75	1,390 (0-2,770)
Global	1990	*Citrobacter* spp.	Aminoglycosides	76	1,350 (1,020-1,670)
Global	1990	*Citrobacter* spp.	3GC	77	1,260 (590-1,920)
Global	1990	*Citrobacter* spp.	4GC	78	1,040 (848-1,230)
Global	1990	*Proteus* spp.	Fluoroquinolones	79	782 (149-1,410)
Global	1990	*Serratia* spp.	3GC	80	761 (0-2,010)
Global	1990	*Proteus* spp.	TMP-SMX	81	494 (0-1,320)
Global	1990	*Mycobacterium tuberculosis*	XDR	82	0 (0-0)
Central Europe, Eastern Europe, and Central Asia	1990	*Streptococcus pneumoniae*	Carbapenems	1	5,380 (3,090-7,680)
Central Europe, Eastern Europe, and Central Asia	1990	*Acinetobacter baumannii*	Carbapenems	2	3,770 (2,690-4,850)
Central Europe, Eastern Europe, and Central Asia	1990	*Staphylococcus aureus*	Methicillin	3	3,640 (1,630-5,660)
Central Europe, Eastern Europe, and Central Asia	1990	*Pseudomonas aeruginosa*	Carbapenems	4	3,000 (1,860-4,130)
Central Europe, Eastern Europe, and Central Asia	1990	*Pseudomonas aeruginosa*	Fluoroquinolones	5	2,340 (1,660-3,020)
Central Europe, Eastern Europe, and Central Asia	1990	*Klebsiella pneumoniae*	Aminoglycosides	6	2,080 (1,560-2,610)
Central Europe, Eastern Europe, and Central Asia	1990	*Acinetobacter baumannii*	Fluoroquinolones	7	1,890 (1,540-2,240)
Central Europe, Eastern Europe, and Central Asia	1990	*Escherichia coli*	TMP-SMX	8	1,770 (1,020-2,530)
Central Europe, Eastern Europe, and Central Asia	1990	*Klebsiella pneumoniae*	Fluoroquinolones	9	1,750 (1,190-2,310)
Central Europe, Eastern Europe, and Central Asia	1990	*Acinetobacter baumannii*	Aminoglycosides	10	1,660 (1,310-2,000)
Central Europe, Eastern Europe, and Central Asia	1990	*Escherichia coli*	Beta-Lactams/Beta-Lactamase Inhib.	11	1,590 (405-2,780)
Central Europe, Eastern Europe, and Central Asia	1990	*Staphylococcus aureus*	Fluoroquinolones	12	1,540 (456-2,630)
Central Europe, Eastern Europe, and Central Asia	1990	*Escherichia coli*	3GC	13	1,470 (707-2,230)
Central Europe, Eastern Europe, and Central Asia	1990	*Klebsiella pneumoniae*	3GC	14	1,470 (878-2,050)
Central Europe, Eastern Europe, and Central Asia	1990	*Staphylococcus aureus*	Macrolides	15	1,420 (944-1,890)
Central Europe, Eastern Europe, and Central Asia	1990	*Streptococcus pneumoniae*	TMP-SMX	16	1,410 (0-3,060)
Central Europe, Eastern Europe, and Central Asia	1990	*Klebsiella pneumoniae*	TMP-SMX	17	1,390 (703-2,070)
Central Europe, Eastern Europe, and Central Asia	1990	*Pseudomonas aeruginosa*	Aminoglycosides	18	1,260 (761-1,750)
Central Europe, Eastern Europe, and Central Asia	1990	*Pseudomonas aeruginosa*	Anti-pseudomonal	19	1,190 (930-1,460)
Central Europe, Eastern Europe, and Central Asia	1990	*Streptococcus pneumoniae*	Penicillin	20	1,110 (831-1,400)
Central Europe, Eastern Europe, and Central Asia	1990	*Streptococcus pneumoniae*	Beta-Lactams/Beta-Lactamase Inhib.	21	1,080 (672-1,490)
Central Europe, Eastern Europe, and Central Asia	1990	*Escherichia coli*	Aminopenicillin	22	1,060 (319-1,800)
Central Europe, Eastern Europe, and Central Asia	1990	*Acinetobacter baumannii*	Anti-pseudomonal	23	1,010 (633-1,390)
Central Europe, Eastern Europe, and Central Asia	1990	*Streptococcus pneumoniae*	Fluoroquinolones	24	899 (328-1,470)
Central Europe, Eastern Europe, and Central Asia	1990	*Enterobacter faecalis*	Fluoroquinolones	25	896 (589-1,200)
Central Europe, Eastern Europe, and Central Asia	1990	*Enterobacter* spp.	Anti-pseudomonal	26	889 (724-1,050)
Central Europe, Eastern Europe, and Central Asia	1990	*Klebsiella pneumoniae*	Carbapenems	27	870 (629-1,110)

Central Europe, Eastern Europe, and Central Asia	1990	*Klebsiella pneumoniae*	Beta-Lactams/Beta-Lactamase Inhib.	28	806 (297-1,310)
Central Europe, Eastern Europe, and Central Asia	1990	*Escherichia coli*	Aminoglycosides	29	786 (236-1,340)
Central Europe, Eastern Europe, and Central Asia	1990	*Streptococcus pneumoniae*	Macrolides	30	779 (428-1,130)
Central Europe, Eastern Europe, and Central Asia	1990	*Enterococcus faecium*	Fluoroquinolones	31	717 (348-1,090)
Central Europe, Eastern Europe, and Central Asia	1990	*Escherichia coli*	Carbapenems	32	695 (63-1,330)
Central Europe, Eastern Europe, and Central Asia	1990	*Staphylococcus aureus*	Vancomycin	33	674 (214-1,130)
Central Europe, Eastern Europe, and Central Asia	1990	*Staphylococcus aureus*	TMP-SMX	34	642 (355-929)
Central Europe, Eastern Europe, and Central Asia	1990	*Serratia* spp.	Aminoglycosides	35	627 (465-789)
Central Europe, Eastern Europe, and Central Asia	1990	*Enterobacter* spp.	Carbapenems	36	625 (436-813)
Central Europe, Eastern Europe, and Central Asia	1990	*Streptococcus pneumoniae*	3GC	37	533 (394-671)
Central Europe, Eastern Europe, and Central Asia	1990	*Serratia* spp.	Anti-pseudomonal	38	527 (420-634)
Central Europe, Eastern Europe, and Central Asia	1990	*Enterobacter* spp.	Fluoroquinolones	39	501 (281-720)
Central Europe, Eastern Europe, and Central Asia	1990	*Enterobacter* spp.	Aminoglycosides	40	498 (295-702)
Central Europe, Eastern Europe, and Central Asia	1990	*Escherichia coli*	Fluoroquinolones	41	498 (89-906)
Central Europe, Eastern Europe, and Central Asia	1990	*Enterobacter* spp.	4GC	42	461 (392-530)
Central Europe, Eastern Europe, and Central Asia	1990	*Acinetobacter baumannii*	3GC	43	430 (313-547)
Central Europe, Eastern Europe, and Central Asia	1990	*Serratia* spp.	4GC	44	396 (345-448)
Central Europe, Eastern Europe, and Central Asia	1990	*Citrobacter* spp.	Anti-pseudomonal	45	389 (338-440)
Central Europe, Eastern Europe, and Central Asia	1990	*Serratia* spp.	Carbapenems	46	273 (208-339)
Central Europe, Eastern Europe, and Central Asia	1990	*Enterobacter* spp.	TMP-SMX	47	250 (34-465)
Central Europe, Eastern Europe, and Central Asia	1990	*Serratia* spp.	Fluoroquinolones	48	244 (97-391)
Central Europe, Eastern Europe, and Central Asia	1990	*Acinetobacter baumannii*	4GC	49	242 (202-282)
Central Europe, Eastern Europe, and Central Asia	1990	*Citrobacter* spp.	4GC	50	239 (209-269)
Central Europe, Eastern Europe, and Central Asia	1990	*Proteus* spp.	Aminoglycosides	51	234 (127-342)
Central Europe, Eastern Europe, and Central Asia	1990	*Proteus* spp.	Aminopenicillin	52	224 (174-274)
Central Europe, Eastern Europe, and Central Asia	1990	*Enterococcus faecium*	Vancomycin	53	222 (164-280)
Central Europe, Eastern Europe, and Central Asia	1990	*Proteus* spp.	3GC	54	219 (107-331)
Central Europe, Eastern Europe, and Central Asia	1990	*Pseudomonas aeruginosa*	4GC	55	215 (119-311)
Central Europe, Eastern Europe, and Central Asia	1990	*Haemophilus influenzae*	Aminopenicillin	56	203 (141-265)
Central Europe, Eastern Europe, and Central Asia	1990	*Citrobacter* spp.	Fluoroquinolones	57	194 (85-304)
Central Europe, Eastern Europe, and Central Asia	1990	*Morganella* spp.	Fluoroquinolones	58	180 (90-269)
Central Europe, Eastern Europe, and Central Asia	1990	*Enterobacter faecalis*	Vancomycin	59	167 (0-395)
Central Europe, Eastern Europe, and Central Asia	1990	*Pseudomonas aeruginosa*	3GC	60	163 (5-320)
Central Europe, Eastern Europe, and Central Asia	1990	*Morganella* spp.	4GC	61	161 (102-220)
Central Europe, Eastern Europe, and Central Asia	1990	*Mycobacterium tuberculosis*	MDR excluding XDR	62	142 (0-471)
Central Europe, Eastern Europe, and Central Asia	1990	*Haemophilus influenzae*	3GC	63	133 (0-369)
Central Europe, Eastern Europe, and Central Asia	1990	*Citrobacter* spp.	Carbapenems	64	127 (60-195)
Central Europe, Eastern Europe, and Central Asia	1990	Group B *Streptococcus*	Fluoroquinolones	65	117 (53-181)
Central Europe, Eastern Europe, and Central Asia	1990	Group B *Streptococcus*	Macrolides	66	97 (61-133)
Central Europe, Eastern Europe, and Central Asia	1990	Group A *Streptococcus*	Macrolides	67	96 (58-134)
Central Europe, Eastern Europe, and Central Asia	1990	*Citrobacter* spp.	Aminoglycosides	68	94 (76-112)
Central Europe, Eastern Europe, and Central Asia	1990	*Proteus* spp.	Fluoroquinolones	69	88 (8-167)
Central Europe, Eastern Europe, and Central Asia	1990	Group B *Streptococcus*	Penicillin	70	58 (0-119)
Central Europe, Eastern Europe, and Central Asia	1990	*Proteus* spp.	TMP-SMX	71	56 (0-158)
Central Europe, Eastern Europe, and Central Asia	1990	*Acinetobacter baumannii*	Beta-Lactams/Beta-Lactamase Inhib.	72	54 (25-82)
Central Europe, Eastern Europe, and Central Asia	1990	*Morganella* spp.	3GC	73	49 (36-62)
Central Europe, Eastern Europe, and Central Asia	1990	*Serratia* spp.	3GC	74	29 (0-88)
Central Europe, Eastern Europe, and Central Asia	1990	*Citrobacter* spp.	3GC	75	24 (0-54)
Central Europe, Eastern Europe, and Central Asia	1990	*Shigella* spp.	Fluoroquinolones	76	11 (0-25)
Central Europe, Eastern Europe, and Central Asia	1990	*Non-typhoidal Salmonella*	Fluoroquinolones	77	5 (0-9)
Central Europe, Eastern Europe, and Central Asia	1990	*Salmonella* Typhi	MDR	78	0 (0-1)
Central Europe, Eastern Europe, and Central Asia	1990	*Salmonella* Typhi	Fluoroquinolones	79	0 (0-0)
Central Europe, Eastern Europe, and Central Asia	1990	*Salmonella* Paratyphi	Fluoroquinolones	80	0 (0-0)
Central Europe, Eastern Europe, and Central Asia	1990	*Salmonella* Paratyphi	MDR	81	0 (0-0)
Central Europe, Eastern Europe, and Central Asia	1990	*Mycobacterium tuberculosis*	XDR	82	0 (0-0)
High-income	1990	*Staphylococcus aureus*	Methicillin	1	13,800 (7,290-20,300)

High-income	1990	*Streptococcus pneumoniae*	Carbapenems	2	11,500 (6,890-16,100)
High-income	1990	*Pseudomonas aeruginosa*	Carbapenems	3	5,570 (3,570-7,570)
High-income	1990	*Acinetobacter baumannii*	Carbapenems	4	4,920 (3,150-6,680)
High-income	1990	*Staphylococcus aureus*	Fluoroquinolones	5	4,470 (1,710-7,240)
High-income	1990	*Streptococcus pneumoniae*	Penicillin	6	4,370 (2,980-5,750)
High-income	1990	*Pseudomonas aeruginosa*	Fluoroquinolones	7	2,930 (2,050-3,820)
High-income	1990	*Acinetobacter baumannii*	Fluoroquinolones	8	2,900 (2,330-3,460)
High-income	1990	*Staphylococcus aureus*	Macrolides	9	2,750 (1,830-3,680)
High-income	1990	*Klebsiella pneumoniae*	Fluoroquinolones	10	2,430 (1,650-3,200)
High-income	1990	*Escherichia coli*	Beta-Lactams/Beta-Lactamase Inhib.	11	2,310 (780-3,830)
High-income	1990	*Klebsiella pneumoniae*	Carbapenems	12	2,200 (1,670-2,730)
High-income	1990	*Acinetobacter baumannii*	Anti-pseudomonal	13	2,070 (1,750-2,390)
High-income	1990	*Escherichia coli*	Aminopenicillin	14	2,070 (763-3,380)
High-income	1990	*Escherichia coli*	TMP-SMX	15	2,030 (1,130-2,920)
High-income	1990	*Acinetobacter baumannii*	Aminoglycosides	16	2,030 (1,420-2,640)
High-income	1990	*Streptococcus pneumoniae*	TMP-SMX	17	2,020 (0-4,430)
High-income	1990	*Streptococcus pneumoniae*	Macrolides	18	1,950 (1,220-2,690)
High-income	1990	*Pseudomonas aeruginosa*	Aminoglycosides	19	1,680 (1,010-2,360)
High-income	1990	*Klebsiella pneumoniae*	TMP-SMX	20	1,610 (812-2,410)
High-income	1990	*Enterococcus faecium*	Fluoroquinolones	21	1,560 (808-2,320)
High-income	1990	*Enterobacter faecalis*	Fluoroquinolones	22	1,510 (984-2,030)
High-income	1990	*Enterobacter* spp.	Anti-pseudomonal	23	1,510 (1,210-1,800)
High-income	1990	*Escherichia coli*	3GC	24	1,500 (729-2,260)
High-income	1990	*Klebsiella pneumoniae*	Aminoglycosides	25	1,460 (962-1,970)
High-income	1990	*Pseudomonas aeruginosa*	Anti-pseudomonal	26	1,440 (1,040-1,830)
High-income	1990	*Enterococcus faecium*	Vancomycin	27	1,440 (1,110-1,770)
High-income	1990	*Streptococcus pneumoniae*	Fluoroquinolones	28	1,420 (621-2,220)
High-income	1990	*Escherichia coli*	Aminoglycosides	29	1,280 (589-1,980)
High-income	1990	*Enterobacter* spp.	Carbapenems	30	1,160 (883-1,430)
High-income	1990	*Staphylococcus aureus*	Vancomycin	31	1,100 (466-1,730)
High-income	1990	*Escherichia coli*	Fluoroquinolones	32	1,000 (320-1,690)
High-income	1990	*Acinetobacter baumannii*	Beta-Lactams/Beta-Lactamase Inhib.	33	975 (643-1,310)
High-income	1990	*Escherichia coli*	Carbapenems	34	887 (255-1,520)
High-income	1990	*Staphylococcus aureus*	TMP-SMX	35	823 (477-1,170)
High-income	1990	*Klebsiella pneumoniae*	3GC	36	789 (442-1,140)
High-income	1990	*Enterobacter* spp.	Fluoroquinolones	37	735 (412-1,060)
High-income	1990	*Acinetobacter baumannii*	4GC	38	721 (491-950)
High-income	1990	*Enterobacter* spp.	Aminoglycosides	39	674 (400-948)
High-income	1990	*Acinetobacter baumannii*	3GC	40	668 (576-759)
High-income	1990	*Haemophilus influenzae*	Aminopenicillin	41	657 (468-845)
High-income	1990	*Proteus* spp.	3GC	42	646 (348-943)
High-income	1990	*Streptococcus pneumoniae*	3GC	43	605 (417-794)
High-income	1990	*Klebsiella pneumoniae*	Beta-Lactams/Beta-Lactamase Inhib.	44	580 (129-1,030)
High-income	1990	*Pseudomonas aeruginosa*	4GC	45	539 (387-690)
High-income	1990	*Citrobacter* spp.	Anti-pseudomonal	46	503 (433-572)
High-income	1990	*Serratia* spp.	Aminoglycosides	47	470 (309-631)
High-income	1990	*Proteus* spp.	Aminopenicillin	48	448 (349-547)
High-income	1990	*Serratia* spp.	Carbapenems	49	427 (336-518)
High-income	1990	*Serratia* spp.	Anti-pseudomonal	50	408 (322-494)
High-income	1990	*Enterobacter* spp.	TMP-SMX	51	387 (48-727)
High-income	1990	*Morganella* spp.	Fluoroquinolones	52	336 (165-508)
High-income	1990	*Streptococcus pneumoniae*	Beta-Lactams/Beta-Lactamase Inhib.	53	335 (186-485)
High-income	1990	*Serratia* spp.	Fluoroquinolones	54	324 (124-524)
High-income	1990	*Proteus* spp.	Aminoglycosides	55	291 (149-433)
High-income	1990	*Citrobacter* spp.	Fluoroquinolones	56	284 (130-437)
High-income	1990	*Enterobacter faecalis*	Vancomycin	57	249 (10-487)

High-income	1990	*Enterobacter* spp.	4GC	58	244 (182-305)
High-income	1990	*Pseudomonas aeruginosa*	3GC	59	211 (9-412)
High-income	1990	*Serratia* spp.	3GC	60	166 (0-401)
High-income	1990	Group A *Streptococcus*	Macrolides	61	162 (97-226)
High-income	1990	Group B *Streptococcus*	Macrolides	62	161 (100-221)
High-income	1990	*Mycobacterium tuberculosis*	MDR excluding XDR	63	155 (0-427)
High-income	1990	*Citrobacter* spp.	Carbapenems	64	144 (66-221)
High-income	1990	*Morganella* spp.	4GC	65	132 (81-182)
High-income	1990	*Proteus* spp.	Fluoroquinolones	66	131 (6-257)
High-income	1990	*Citrobacter* spp.	4GC	67	131 (114-148)
High-income	1990	*Morganella* spp.	3GC	68	120 (86-153)
High-income	1990	*Serratia* spp.	4GC	69	117 (89-146)
High-income	1990	*Citrobacter* spp.	3GC	70	114 (14-215)
High-income	1990	*Haemophilus influenzae*	3GC	71	109 (0-267)
High-income	1990	*Proteus* spp.	TMP-SMX	72	78 (0-215)
High-income	1990	Group B *Streptococcus*	Fluoroquinolones	73	69 (31-107)
High-income	1990	*Citrobacter* spp.	Aminoglycosides	74	67 (53-81)
High-income	1990	Group B *Streptococcus*	Penicillin	75	56 (6-105)
High-income	1990	*Non-typhoidal Salmonella*	Fluoroquinolones	76	8 (0-16)
High-income	1990	*Salmonella* Typhi	MDR	77	1 (0-2)
High-income	1990	*Shigella* spp.	Fluoroquinolones	78	1 (0-2)
High-income	1990	*Salmonella* Typhi	Fluoroquinolones	79	0 (0-0)
High-income	1990	*Salmonella* Paratyphi	Fluoroquinolones	80	0 (0-0)
High-income	1990	*Salmonella* Paratyphi	MDR	81	0 (0-0)
High-income	1990	*Mycobacterium tuberculosis*	XDR	82	0 (0-0)
Latin America and Caribbean	1990	*Streptococcus pneumoniae*	Carbapenems	1	5,170 (2,770-7,570)
Latin America and Caribbean	1990	*Acinetobacter baumannii*	Carbapenems	2	3,930 (2,770-5,090)
Latin America and Caribbean	1990	*Staphylococcus aureus*	Methicillin	3	2,690 (1,290-4,080)
Latin America and Caribbean	1990	*Streptococcus pneumoniae*	Penicillin	4	2,380 (1,620-3,130)
Latin America and Caribbean	1990	*Acinetobacter baumannii*	Fluoroquinolones	5	2,120 (1,730-2,520)
Latin America and Caribbean	1990	*Pseudomonas aeruginosa*	Carbapenems	6	2,100 (1,300-2,900)
Latin America and Caribbean	1990	*Streptococcus pneumoniae*	TMP-SMX	7	2,060 (0-4,470)
Latin America and Caribbean	1990	*Klebsiella pneumoniae*	Aminoglycosides	8	2,000 (1,470-2,530)
Latin America and Caribbean	1990	*Acinetobacter baumannii*	Aminoglycosides	9	1,950 (1,510-2,380)
Latin America and Caribbean	1990	*Pseudomonas aeruginosa*	Fluoroquinolones	10	1,760 (1,250-2,270)
Latin America and Caribbean	1990	*Klebsiella pneumoniae*	3GC	11	1,660 (1,060-2,260)
Latin America and Caribbean	1990	*Acinetobacter baumannii*	Anti-pseudomonal	12	1,520 (1,150-1,900)
Latin America and Caribbean	1990	*Escherichia coli*	TMP-SMX	13	1,280 (824-1,730)
Latin America and Caribbean	1990	*Klebsiella pneumoniae*	TMP-SMX	14	1,270 (649-1,890)
Latin America and Caribbean	1990	*Klebsiella pneumoniae*	Fluoroquinolones	15	1,250 (851-1,650)
Latin America and Caribbean	1990	*Pseudomonas aeruginosa*	Anti-pseudomonal	16	1,230 (996-1,470)
Latin America and Caribbean	1990	*Streptococcus pneumoniae*	Fluoroquinolones	17	1,220 (426-2,020)
Latin America and Caribbean	1990	*Klebsiella pneumoniae*	Beta-Lactams/Beta-Lactamase Inhib.	18	1,110 (521-1,700)
Latin America and Caribbean	1990	*Klebsiella pneumoniae*	Carbapenems	19	1,020 (750-1,290)
Latin America and Caribbean	1990	*Escherichia coli*	3GC	20	1,010 (491-1,530)
Latin America and Caribbean	1990	*Pseudomonas aeruginosa*	Aminoglycosides	21	978 (621-1,340)
Latin America and Caribbean	1990	*Streptococcus pneumoniae*	3GC	22	915 (666-1,160)
Latin America and Caribbean	1990	*Staphylococcus aureus*	TMP-SMX	23	896 (554-1,240)
Latin America and Caribbean	1990	*Escherichia coli*	Aminoglycosides	24	814 (429-1,200)
Latin America and Caribbean	1990	*Escherichia coli*	Beta-Lactams/Beta-Lactamase Inhib.	25	804 (156-1,450)
Latin America and Caribbean	1990	*Staphylococcus aureus*	Macrolides	26	710 (462-959)
Latin America and Caribbean	1990	*Staphylococcus aureus*	Fluoroquinolones	27	700 (251-1,150)
Latin America and Caribbean	1990	*Acinetobacter baumannii*	3GC	28	604 (488-721)
Latin America and Caribbean	1990	*Streptococcus pneumoniae*	Macrolides	29	581 (312-850)
Latin America and Caribbean	1990	*Escherichia coli*	Aminopenicillin	30	509 (248-770)
Latin America and Caribbean	1990	*Enterobacter* spp.	Anti-pseudomonal	31	506 (403-609)

Latin America and Caribbean	1990	*Acinetobacter baumannii*	4GC	32	500 (443-556)
Latin America and Caribbean	1990	*Enterobacter* spp.	Aminoglycosides	33	490 (345-635)
Latin America and Caribbean	1990	*Escherichia coli*	Fluoroquinolones	34	482 (240-724)
Latin America and Caribbean	1990	*Escherichia coli*	Carbapenems	35	473 (143-803)
Latin America and Caribbean	1990	*Serratia* spp.	Aminoglycosides	36	403 (282-524)
Latin America and Caribbean	1990	*Staphylococcus aureus*	Vancomycin	37	398 (204-593)
Latin America and Caribbean	1990	*Enterococcus faecium*	Vancomycin	38	390 (303-478)
Latin America and Caribbean	1990	*Enterobacter* spp.	Carbapenems	39	387 (291-483)
Latin America and Caribbean	1990	*Serratia* spp.	Anti-pseudomonal	40	334 (263-406)
Latin America and Caribbean	1990	*Enterobacter faecalis*	Fluoroquinolones	41	326 (218-433)
Latin America and Caribbean	1990	*Streptococcus pneumoniae*	Beta-Lactams/Beta-Lactamase Inhib.	42	326 (138-514)
Latin America and Caribbean	1990	*Enterococcus faecium*	Fluoroquinolones	43	306 (149-463)
Latin America and Caribbean	1990	*Enterobacter* spp.	Fluoroquinolones	44	283 (165-400)
Latin America and Caribbean	1990	*Serratia* spp.	4GC	45	250 (212-287)
Latin America and Caribbean	1990	*Serratia* spp.	Fluoroquinolones	46	235 (100-370)
Latin America and Caribbean	1990	*Haemophilus influenzae*	Aminopenicillin	47	219 (161-277)
Latin America and Caribbean	1990	*Enterobacter faecalis*	Vancomycin	48	198 (0-431)
Latin America and Caribbean	1990	*Citrobacter* spp.	Anti-pseudomonal	49	188 (155-222)
Latin America and Caribbean	1990	*Serratia* spp.	Carbapenems	50	182 (139-225)
Latin America and Caribbean	1990	*Pseudomonas aeruginosa*	4GC	51	179 (102-256)
Latin America and Caribbean	1990	*Haemophilus influenzae*	3GC	52	177 (0-427)
Latin America and Caribbean	1990	*Enterobacter* spp.	4GC	53	158 (132-184)
Latin America and Caribbean	1990	*Proteus* spp.	3GC	54	156 (78-234)
Latin America and Caribbean	1990	*Proteus* spp.	Aminopenicillin	55	149 (117-182)
Latin America and Caribbean	1990	*Pseudomonas aeruginosa*	3GC	56	146 (62-229)
Latin America and Caribbean	1990	*Morganella* spp.	Fluoroquinolones	57	140 (70-210)
Latin America and Caribbean	1990	*Citrobacter* spp.	Fluoroquinolones	58	132 (63-200)
Latin America and Caribbean	1990	*Enterobacter* spp.	TMP-SMX	59	119 (18-219)
Latin America and Caribbean	1990	*Proteus* spp.	Aminoglycosides	60	100 (63-137)
Latin America and Caribbean	1990	*Mycobacterium tuberculosis*	MDR excluding XDR	61	95 (0-275)
Latin America and Caribbean	1990	*Morganella* spp.	4GC	62	94 (60-128)
Latin America and Caribbean	1990	*Non-typhoidal Salmonella*	Fluoroquinolones	63	94 (0-207)
Latin America and Caribbean	1990	Group B *Streptococcus*	Macrolides	64	92 (56-127)
Latin America and Caribbean	1990	*Citrobacter* spp.	4GC	65	87 (72-102)
Latin America and Caribbean	1990	*Citrobacter* spp.	Carbapenems	66	78 (36-121)
Latin America and Caribbean	1990	*Citrobacter* spp.	Aminoglycosides	67	76 (59-92)
Latin America and Caribbean	1990	Group B *Streptococcus*	Penicillin	68	73 (0-147)
Latin America and Caribbean	1990	*Shigella* spp.	Fluoroquinolones	69	63 (0-131)
Latin America and Caribbean	1990	Group B *Streptococcus*	Fluoroquinolones	70	53 (23-83)
Latin America and Caribbean	1990	*Serratia* spp.	3GC	71	52 (0-135)
Latin America and Caribbean	1990	*Morganella* spp.	3GC	72	51 (37-65)
Latin America and Caribbean	1990	Group A *Streptococcus*	Macrolides	73	48 (29-67)
Latin America and Caribbean	1990	*Acinetobacter baumannii*	Beta-Lactams/Beta-Lactamase Inhib.	74	46 (24-67)
Latin America and Caribbean	1990	*Proteus* spp.	Fluoroquinolones	75	29 (0-58)
Latin America and Caribbean	1990	*Proteus* spp.	TMP-SMX	76	27 (0-74)
Latin America and Caribbean	1990	*Citrobacter* spp.	3GC	77	24 (3-45)
Latin America and Caribbean	1990	*Salmonella* Typhi	MDR	78	20 (0-41)
Latin America and Caribbean	1990	*Salmonella* Typhi	Fluoroquinolones	79	3 (1-5)
Latin America and Caribbean	1990	*Salmonella* Paratyphi	Fluoroquinolones	80	1 (0-1)
Latin America and Caribbean	1990	*Salmonella* Paratyphi	MDR	81	0 (0-1)
Latin America and Caribbean	1990	*Mycobacterium tuberculosis*	XDR	82	0 (0-0)
North Africa and Middle East	1990	*Streptococcus pneumoniae*	Carbapenems	1	8,570 (4,710-12,400)
North Africa and Middle East	1990	*Acinetobacter baumannii*	Carbapenems	2	3,540 (2,510-4,580)
North Africa and Middle East	1990	*Staphylococcus aureus*	Methicillin	3	3,420 (1,950-4,890)
North Africa and Middle East	1990	*Streptococcus pneumoniae*	Beta-Lactams/Beta-Lactamase Inhib.	4	3,160 (1,960-4,370)
North Africa and Middle East	1990	*Streptococcus pneumoniae*	TMP-SMX	5	2,080 (0-4,370)

North Africa and Middle East	1990	*Klebsiella pneumoniae*	Aminoglycosides	6	2,010 (1,450-2,560)
North Africa and Middle East	1990	*Acinetobacter baumannii*	Fluoroquinolones	7	2,000 (1,620-2,380)
North Africa and Middle East	1990	*Streptococcus pneumoniae*	Penicillin	8	1,920 (1,280-2,550)
North Africa and Middle East	1990	*Streptococcus pneumoniae*	Fluoroquinolones	9	1,860 (700-3,030)
North Africa and Middle East	1990	*Klebsiella pneumoniae*	3GC	10	1,860 (1,130-2,580)
North Africa and Middle East	1990	*Pseudomonas aeruginosa*	Carbapenems	11	1,770 (1,040-2,490)
North Africa and Middle East	1990	*Acinetobacter baumannii*	Aminoglycosides	12	1,520 (1,140-1,900)
North Africa and Middle East	1990	*Klebsiella pneumoniae*	TMP-SMX	13	1,420 (730-2,120)
North Africa and Middle East	1990	*Klebsiella pneumoniae*	Fluoroquinolones	14	1,420 (935-1,910)
North Africa and Middle East	1990	*Acinetobacter baumannii*	Anti-pseudomonal	15	1,400 (1,030-1,770)
North Africa and Middle East	1990	*Klebsiella pneumoniae*	Beta-Lactams/Beta-Lactamase Inhib.	16	1,360 (635-2,080)
North Africa and Middle East	1990	*Pseudomonas aeruginosa*	Anti-pseudomonal	17	1,350 (1,050-1,660)
North Africa and Middle East	1990	*Pseudomonas aeruginosa*	Fluoroquinolones	18	1,350 (947-1,760)
North Africa and Middle East	1990	*Streptococcus pneumoniae*	3GC	19	1,340 (930-1,740)
North Africa and Middle East	1990	*Escherichia coli*	TMP-SMX	20	1,240 (818-1,660)
North Africa and Middle East	1990	*Pseudomonas aeruginosa*	Aminoglycosides	21	1,100 (679-1,510)
North Africa and Middle East	1990	*Escherichia coli*	Beta-Lactams/Beta-Lactamase Inhib.	22	1,030 (110-1,950)
North Africa and Middle East	1990	*Escherichia coli*	3GC	23	1,030 (516-1,540)
North Africa and Middle East	1990	*Streptococcus pneumoniae*	Macrolides	24	947 (518-1,380)
North Africa and Middle East	1990	*Klebsiella pneumoniae*	Carbapenems	25	868 (631-1,110)
North Africa and Middle East	1990	*Staphylococcus aureus*	Vancomycin	26	811 (443-1,180)
North Africa and Middle East	1990	*Escherichia coli*	Aminoglycosides	27	711 (310-1,110)
North Africa and Middle East	1990	*Staphylococcus aureus*	TMP-SMX	28	685 (416-953)
North Africa and Middle East	1990	*Acinetobacter baumannii*	3GC	29	670 (448-891)
North Africa and Middle East	1990	*Staphylococcus aureus*	Fluoroquinolones	30	626 (252-1,000)
North Africa and Middle East	1990	*Escherichia coli*	Carbapenems	31	592 (115-1,070)
North Africa and Middle East	1990	*Escherichia coli*	Fluoroquinolones	32	577 (264-891)
North Africa and Middle East	1990	*Enterobacter* spp.	Anti-pseudomonal	33	528 (415-642)
North Africa and Middle East	1990	*Staphylococcus aureus*	Macrolides	34	484 (312-656)
North Africa and Middle East	1990	*Escherichia coli*	Aminopenicillin	35	451 (233-669)
North Africa and Middle East	1990	*Serratia* spp.	Anti-pseudomonal	36	424 (305-543)
North Africa and Middle East	1990	*Acinetobacter baumannii*	4GC	37	384 (325-442)
North Africa and Middle East	1990	*Pseudomonas aeruginosa*	4GC	38	381 (216-545)
North Africa and Middle East	1990	*Serratia* spp.	Aminoglycosides	39	381 (259-502)
North Africa and Middle East	1990	*Enterobacter faecalis*	Fluoroquinolones	40	352 (226-478)
North Africa and Middle East	1990	*Enterobacter* spp.	Aminoglycosides	41	342 (225-460)
North Africa and Middle East	1990	*Enterobacter* spp.	Carbapenems	42	326 (237-415)
North Africa and Middle East	1990	*Haemophilus influenzae*	3GC	43	324 (0-754)
North Africa and Middle East	1990	*Enterococcus faecium*	Fluoroquinolones	44	247 (114-381)
North Africa and Middle East	1990	*Serratia* spp.	Carbapenems	45	243 (184-302)
North Africa and Middle East	1990	*Serratia* spp.	Fluoroquinolones	46	227 (92-362)
North Africa and Middle East	1990	*Haemophilus influenzae*	Aminopenicillin	47	226 (172-279)
North Africa and Middle East	1990	*Enterobacter* spp.	4GC	48	201 (164-238)
North Africa and Middle East	1990	*Enterococcus faecium*	Vancomycin	49	201 (151-251)
North Africa and Middle East	1990	*Citrobacter* spp.	Anti-pseudomonal	50	185 (143-228)
North Africa and Middle East	1990	*Pseudomonas aeruginosa*	3GC	51	185 (52-317)
North Africa and Middle East	1990	*Enterobacter faecalis*	Vancomycin	52	180 (0-399)
North Africa and Middle East	1990	*Proteus* spp.	3GC	53	168 (93-243)
North Africa and Middle East	1990	*Enterobacter* spp.	Fluoroquinolones	54	167 (94-241)
North Africa and Middle East	1990	Group B *Streptococcus*	Penicillin	55	159 (4-313)
North Africa and Middle East	1990	*Morganella* spp.	Fluoroquinolones	56	145 (72-218)
North Africa and Middle East	1990	*Enterobacter* spp.	TMP-SMX	57	140 (22-259)
North Africa and Middle East	1990	*Citrobacter* spp.	Fluoroquinolones	58	140 (72-209)
North Africa and Middle East	1990	Group B *Streptococcus*	Fluoroquinolones	59	137 (62-211)
North Africa and Middle East	1990	*Proteus* spp.	Aminopenicillin	60	118 (80-155)
North Africa and Middle East	1990	*Morganella* spp.	3GC	61	115 (83-148)

North Africa and Middle East	1990	*Serratia* spp.	4GC	62	107 (86-127)
North Africa and Middle East	1990	Group B *Streptococcus*	Macrolides	63	105 (66-145)
North Africa and Middle East	1990	*Salmonella* Typhi	MDR	64	101 (0-217)
North Africa and Middle East	1990	*Proteus* spp.	Aminoglycosides	65	101 (59-142)
North Africa and Middle East	1990	Group A *Streptococcus*	Macrolides	66	95 (58-131)
North Africa and Middle East	1990	*Citrobacter* spp.	Carbapenems	67	88 (41-135)
North Africa and Middle East	1990	*Non-typhoidal Salmonella*	Fluoroquinolones	68	88 (0-198)
North Africa and Middle East	1990	*Morganella* spp.	4GC	69	76 (48-105)
North Africa and Middle East	1990	*Citrobacter* spp.	Aminoglycosides	70	73 (55-92)
North Africa and Middle East	1990	*Shigella* spp.	Fluoroquinolones	71	68 (0-149)
North Africa and Middle East	1990	*Citrobacter* spp.	4GC	72	67 (52-82)
North Africa and Middle East	1990	*Serratia* spp.	3GC	73	58 (0-159)
North Africa and Middle East	1990	*Acinetobacter baumannii*	Beta-Lactams/Beta-Lactamase Inhib.	74	52 (12-93)
North Africa and Middle East	1990	*Mycobacterium tuberculosis*	MDR excluding XDR	75	50 (0-136)
North Africa and Middle East	1990	*Proteus* spp.	Fluoroquinolones	76	39 (3-76)
North Africa and Middle East	1990	*Citrobacter* spp.	3GC	77	36 (5-66)
North Africa and Middle East	1990	*Proteus* spp.	TMP-SMX	78	28 (0-76)
North Africa and Middle East	1990	*Salmonella* Typhi	Fluoroquinolones	79	25 (0-50)
North Africa and Middle East	1990	*Salmonella* Paratyphi	Fluoroquinolones	80	0 (0-1)
North Africa and Middle East	1990	*Salmonella* Paratyphi	MDR	81	0 (0-1)
North Africa and Middle East	1990	*Mycobacterium tuberculosis*	XDR	82	0 (0-0)
South Asia	1990	*Streptococcus pneumoniae*	Carbapenems	1	26,800 (14,500-39,000)
South Asia	1990	*Acinetobacter baumannii*	Carbapenems	2	16,400 (10,800-22,000)
South Asia	1990	*Staphylococcus aureus*	Methicillin	3	14,600 (9,910-19,200)
South Asia	1990	*Klebsiella pneumoniae*	3GC	4	12,800 (8,130-17,500)
South Asia	1990	*Acinetobacter baumannii*	Fluoroquinolones	5	10,800 (8,840-12,800)
South Asia	1990	*Streptococcus pneumoniae*	3GC	6	10,700 (7,710-13,700)
South Asia	1990	*Escherichia coli*	3GC	7	10,200 (4,030-16,300)
South Asia	1990	*Streptococcus pneumoniae*	Fluoroquinolones	8	10,100 (4,090-16,200)
South Asia	1990	*Klebsiella pneumoniae*	Aminoglycosides	9	9,920 (7,100-12,700)
South Asia	1990	*Escherichia coli*	TMP-SMX	10	9,860 (6,550-13,200)
South Asia	1990	*Klebsiella pneumoniae*	Fluoroquinolones	11	9,220 (6,530-11,900)
South Asia	1990	*Acinetobacter baumannii*	Aminoglycosides	12	8,300 (6,280-10,300)
South Asia	1990	*Salmonella* Typhi	Fluoroquinolones	13	8,160 (1,170-15,100)
South Asia	1990	*Streptococcus pneumoniae*	TMP-SMX	14	7,960 (0-17,000)
South Asia	1990	*Klebsiella pneumoniae*	Carbapenems	15	7,200 (5,200-9,190)
South Asia	1990	*Pseudomonas aeruginosa*	Carbapenems	16	7,170 (3,990-10,400)
South Asia	1990	*Pseudomonas aeruginosa*	Fluoroquinolones	17	6,760 (4,770-8,740)
South Asia	1990	*Acinetobacter baumannii*	Anti-pseudomonal	18	6,740 (4,720-8,760)
South Asia	1990	*Klebsiella pneumoniae*	TMP-SMX	19	6,730 (3,460-10,000)
South Asia	1990	*Escherichia coli*	Fluoroquinolones	20	6,200 (3,890-8,510)
South Asia	1990	*Escherichia coli*	Aminoglycosides	21	5,950 (2,600-9,300)
South Asia	1990	*Escherichia coli*	Beta-Lactams/Beta-Lactamase Inhib.	22	5,730 (70-11,400)
South Asia	1990	*Pseudomonas aeruginosa*	Aminoglycosides	23	5,240 (3,420-7,050)
South Asia	1990	*Pseudomonas aeruginosa*	Anti-pseudomonal	24	5,080 (3,980-6,170)
South Asia	1990	*Klebsiella pneumoniae*	Beta-Lactams/Beta-Lactamase Inhib.	25	4,440 (2,360-6,510)
South Asia	1990	*Escherichia coli*	Carbapenems	26	4,390 (1,230-7,540)
South Asia	1990	*Staphylococcus aureus*	TMP-SMX	27	3,960 (2,430-5,490)
South Asia	1990	*Salmonella* Typhi	MDR	28	3,950 (600-7,300)
South Asia	1990	*Streptococcus pneumoniae*	Penicillin	29	3,830 (1,720-5,930)
South Asia	1990	*Shigella* spp.	Fluoroquinolones	30	3,440 (808-6,070)
South Asia	1990	*Staphylococcus aureus*	Fluoroquinolones	31	2,780 (1,130-4,420)
South Asia	1990	*Serratia* spp.	Anti-pseudomonal	32	2,210 (1,590-2,830)
South Asia	1990	*Salmonella* Paratyphi	MDR	33	2,180 (160-4,190)
South Asia	1990	*Acinetobacter baumannii*	3GC	34	2,170 (1,730-2,610)
South Asia	1990	*Streptococcus pneumoniae*	Macrolides	35	2,000 (1,050-2,940)

South Asia	1990	*Enterobacter faecalis*	Fluoroquinolones	36	1,970 (1,300-2,640)
South Asia	1990	*Serratia* spp.	Aminoglycosides	37	1,960 (1,300-2,610)
South Asia	1990	*Enterobacter* spp.	Aminoglycosides	38	1,910 (1,340-2,490)
South Asia	1990	*Pseudomonas aeruginosa*	4GC	39	1,880 (1,160-2,590)
South Asia	1990	*Staphylococcus aureus*	Macrolides	40	1,870 (1,180-2,560)
South Asia	1990	*Enterobacter* spp.	Anti-pseudomonal	41	1,850 (1,430-2,270)
South Asia	1990	*Haemophilus influenzae*	3GC	42	1,770 (0-3,550)
South Asia	1990	*Enterobacter* spp.	4GC	43	1,510 (1,220-1,800)
South Asia	1990	*Enterobacter* spp.	Carbapenems	44	1,490 (1,090-1,880)
South Asia	1990	*Escherichia coli*	Aminopenicillin	45	1,440 (755-2,130)
South Asia	1990	*Haemophilus influenzae*	Aminopenicillin	46	1,360 (1,040-1,690)
South Asia	1990	*Salmonella* Paratyphi	Fluoroquinolones	47	1,360 (0-2,720)
South Asia	1990	*Enterococcus faecium*	Fluoroquinolones	48	1,350 (613-2,080)
South Asia	1990	*Morganella* spp.	4GC	49	1,270 (874-1,670)
South Asia	1990	*Acinetobacter baumannii*	4GC	50	1,230 (895-1,570)
South Asia	1990	*Enterobacter* spp.	Fluoroquinolones	51	1,130 (648-1,620)
South Asia	1990	*Non-typhoidal Salmonella*	Fluoroquinolones	52	1,100 (0-2,600)
South Asia	1990	*Proteus* spp.	3GC	53	1,070 (662-1,480)
South Asia	1990	*Citrobacter* spp.	Carbapenems	54	1,060 (526-1,590)
South Asia	1990	*Serratia* spp.	Fluoroquinolones	55	1,040 (408-1,670)
South Asia	1990	*Morganella* spp.	3GC	56	1,030 (705-1,360)
South Asia	1990	Group B *Streptococcus*	Fluoroquinolones	57	992 (470-1,510)
South Asia	1990	*Citrobacter* spp.	Fluoroquinolones	58	963 (596-1,330)
South Asia	1990	*Enterococcus faecium*	Vancomycin	59	938 (703-1,170)
South Asia	1990	*Morganella* spp.	Fluoroquinolones	60	816 (435-1,200)
South Asia	1990	*Citrobacter* spp.	3GC	61	787 (486-1,090)
South Asia	1990	*Serratia* spp.	4GC	62	715 (569-862)
South Asia	1990	*Serratia* spp.	Carbapenems	63	679 (494-864)
South Asia	1990	*Staphylococcus aureus*	Vancomycin	64	666 (286-1,050)
South Asia	1990	*Proteus* spp.	Aminoglycosides	65	665 (472-857)
South Asia	1990	*Acinetobacter baumannii*	Beta-Lactams/Beta-Lactamase Inhib.	66	613 (230-996)
South Asia	1990	Group B *Streptococcus*	Macrolides	67	611 (379-843)
South Asia	1990	*Enterobacter* spp.	TMP-SMX	68	591 (100-1,080)
South Asia	1990	*Enterobacter faecalis*	Vancomycin	69	562 (0-1,330)
South Asia	1990	*Citrobacter* spp.	Anti-pseudomonal	70	521 (377-666)
South Asia	1990	*Citrobacter* spp.	Aminoglycosides	71	517 (365-670)
South Asia	1990	*Mycobacterium tuberculosis*	MDR excluding XDR	72	468 (0-1,910)
South Asia	1990	Group B *Streptococcus*	Penicillin	73	457 (0-918)
South Asia	1990	Group A *Streptococcus*	Macrolides	74	421 (262-581)
South Asia	1990	*Pseudomonas aeruginosa*	3GC	75	376 (1-751)
South Asia	1990	*Serratia* spp.	3GC	76	291 (0-821)
South Asia	1990	*Proteus* spp.	Fluoroquinolones	77	282 (97-467)
South Asia	1990	*Proteus* spp.	Aminopenicillin	78	220 (149-291)
South Asia	1990	*Streptococcus pneumoniae*	Beta-Lactams/Beta-Lactamase Inhib.	79	184 (94-274)
South Asia	1990	*Citrobacter* spp.	4GC	80	158 (109-206)
South Asia	1990	*Proteus* spp.	TMP-SMX	81	143 (0-376)
South Asia	1990	*Mycobacterium tuberculosis*	XDR	82	0 (0-0)
Southeast Asia, East Asia, and Oceania	1990	*Streptococcus pneumoniae*	Carbapenems	1	24,600 (13,800-35,500)
Southeast Asia, East Asia, and Oceania	1990	*Streptococcus pneumoniae*	Fluoroquinolones	2	19,000 (9,430-28,500)
Southeast Asia, East Asia, and Oceania	1990	*Staphylococcus aureus*	Methicillin	3	14,200 (8,980-19,500)
Southeast Asia, East Asia, and Oceania	1990	*Acinetobacter baumannii*	Carbapenems	4	14,100 (8,910-19,200)
Southeast Asia, East Asia, and Oceania	1990	*Acinetobacter baumannii*	Anti-pseudomonal	5	11,900 (9,820-14,000)
Southeast Asia, East Asia, and Oceania	1990	*Klebsiella pneumoniae*	Fluoroquinolones	6	8,590 (5,880-11,300)
Southeast Asia, East Asia, and Oceania	1990	*Pseudomonas aeruginosa*	Carbapenems	7	8,380 (4,820-11,900)
Southeast Asia, East Asia, and Oceania	1990	*Acinetobacter baumannii*	Fluoroquinolones	8	7,030 (5,550-8,520)
Southeast Asia, East Asia, and Oceania	1990	*Klebsiella pneumoniae*	3GC	9	6,710 (4,200-9,220)

Southeast Asia, East Asia, and Oceania	1990	*Acinetobacter baumannii*	Aminoglycosides	10	6,150 (4,330-7,960)
Southeast Asia, East Asia, and Oceania	1990	*Pseudomonas aeruginosa*	Fluoroquinolones	11	6,060 (4,190-7,930)
Southeast Asia, East Asia, and Oceania	1990	*Streptococcus pneumoniae*	Macrolides	12	5,990 (3,640-8,350)
Southeast Asia, East Asia, and Oceania	1990	*Streptococcus pneumoniae*	Penicillin	13	5,750 (3,430-8,070)
Southeast Asia, East Asia, and Oceania	1990	*Pseudomonas aeruginosa*	Anti-pseudomonal	14	5,350 (3,950-6,750)
Southeast Asia, East Asia, and Oceania	1990	*Klebsiella pneumoniae*	Aminoglycosides	15	4,990 (3,360-6,620)
Southeast Asia, East Asia, and Oceania	1990	*Escherichia coli*	Beta-Lactams/Beta-Lactamase Inhib.	16	4,940 (1,120-8,750)
Southeast Asia, East Asia, and Oceania	1990	*Escherichia coli*	TMP-SMX	17	4,750 (3,110-6,380)
Southeast Asia, East Asia, and Oceania	1990	*Streptococcus pneumoniae*	TMP-SMX	18	4,560 (0-9,670)
Southeast Asia, East Asia, and Oceania	1990	*Klebsiella pneumoniae*	Beta-Lactams/Beta-Lactamase Inhib.	19	4,440 (1,810-7,070)
Southeast Asia, East Asia, and Oceania	1990	*Enterobacter* spp.	Carbapenems	20	4,040 (3,080-5,000)
Southeast Asia, East Asia, and Oceania	1990	*Mycobacterium tuberculosis*	MDR excluding XDR	21	3,840 (0-12,400)
Southeast Asia, East Asia, and Oceania	1990	*Staphylococcus aureus*	Fluoroquinolones	22	3,820 (1,410-6,230)
Southeast Asia, East Asia, and Oceania	1990	*Klebsiella pneumoniae*	TMP-SMX	23	3,510 (1,750-5,260)
Southeast Asia, East Asia, and Oceania	1990	*Staphylococcus aureus*	Macrolides	24	3,330 (2,150-4,510)
Southeast Asia, East Asia, and Oceania	1990	*Escherichia coli*	Fluoroquinolones	25	3,290 (1,810-4,770)
Southeast Asia, East Asia, and Oceania	1990	*Staphylococcus aureus*	TMP-SMX	26	3,170 (1,850-4,500)
Southeast Asia, East Asia, and Oceania	1990	*Escherichia coli*	3GC	27	3,130 (1,050-5,210)
Southeast Asia, East Asia, and Oceania	1990	*Streptococcus pneumoniae*	3GC	28	3,110 (2,100-4,130)
Southeast Asia, East Asia, and Oceania	1990	*Pseudomonas aeruginosa*	Aminoglycosides	29	3,090 (1,780-4,400)
Southeast Asia, East Asia, and Oceania	1990	*Escherichia coli*	Aminoglycosides	30	2,770 (1,260-4,280)
Southeast Asia, East Asia, and Oceania	1990	*Klebsiella pneumoniae*	Carbapenems	31	2,600 (1,850-3,340)
Southeast Asia, East Asia, and Oceania	1990	*Escherichia coli*	Aminopenicillin	32	2,300 (1,530-3,070)
Southeast Asia, East Asia, and Oceania	1990	*Streptococcus pneumoniae*	Beta-Lactams/Beta-Lactamase Inhib.	33	2,020 (721-3,330)
Southeast Asia, East Asia, and Oceania	1990	*Serratia* spp.	4GC	34	2,010 (1,680-2,340)
Southeast Asia, East Asia, and Oceania	1990	*Serratia* spp.	Anti-pseudomonal	35	1,850 (1,480-2,230)
Southeast Asia, East Asia, and Oceania	1990	*Escherichia coli*	Carbapenems	36	1,830 (588-3,080)
Southeast Asia, East Asia, and Oceania	1990	*Enterobacter* spp.	Anti-pseudomonal	37	1,820 (1,460-2,180)
Southeast Asia, East Asia, and Oceania	1990	*Enterococcus faecium*	Fluoroquinolones	38	1,730 (853-2,600)
Southeast Asia, East Asia, and Oceania	1990	*Haemophilus influenzae*	Aminopenicillin	39	1,570 (1,160-1,980)
Southeast Asia, East Asia, and Oceania	1990	*Serratia* spp.	Aminoglycosides	40	1,540 (1,070-2,010)
Southeast Asia, East Asia, and Oceania	1990	*Enterobacter faecalis*	Fluoroquinolones	41	1,470 (972-1,960)
Southeast Asia, East Asia, and Oceania	1990	*Enterobacter* spp.	Fluoroquinolones	42	1,410 (821-1,990)
Southeast Asia, East Asia, and Oceania	1990	*Staphylococcus aureus*	Vancomycin	43	1,300 (628-1,980)
Southeast Asia, East Asia, and Oceania	1990	*Pseudomonas aeruginosa*	3GC	44	1,220 (377-2,070)
Southeast Asia, East Asia, and Oceania	1990	*Enterobacter* spp.	Aminoglycosides	45	1,210 (841-1,580)
Southeast Asia, East Asia, and Oceania	1990	Group B *Streptococcus*	Fluoroquinolones	46	1,170 (587-1,750)
Southeast Asia, East Asia, and Oceania	1990	*Acinetobacter baumannii*	Beta-Lactams/Beta-Lactamase Inhib.	47	1,160 (733-1,590)
Southeast Asia, East Asia, and Oceania	1990	*Serratia* spp.	Fluoroquinolones	48	1,150 (521-1,780)
Southeast Asia, East Asia, and Oceania	1990	Group A *Streptococcus*	Macrolides	49	1,090 (675-1,510)
Southeast Asia, East Asia, and Oceania	1990	Group B *Streptococcus*	Macrolides	50	838 (524-1,150)
Southeast Asia, East Asia, and Oceania	1990	*Pseudomonas aeruginosa*	4GC	51	830 (486-1,170)
Southeast Asia, East Asia, and Oceania	1990	*Citrobacter* spp.	Fluoroquinolones	52	821 (475-1,170)
Southeast Asia, East Asia, and Oceania	1990	*Enterobacter* spp.	4GC	53	804 (659-948)
Southeast Asia, East Asia, and Oceania	1990	*Citrobacter* spp.	Anti-pseudomonal	54	786 (615-956)
Southeast Asia, East Asia, and Oceania	1990	*Acinetobacter baumannii*	4GC	55	783 (633-932)
Southeast Asia, East Asia, and Oceania	1990	*Proteus* spp.	Aminopenicillin	56	769 (590-948)
Southeast Asia, East Asia, and Oceania	1990	*Serratia* spp.	Carbapenems	57	704 (523-886)
Southeast Asia, East Asia, and Oceania	1990	*Morganella* spp.	3GC	58	661 (486-836)
Southeast Asia, East Asia, and Oceania	1990	*Morganella* spp.	4GC	59	598 (387-808)
Southeast Asia, East Asia, and Oceania	1990	*Enterococcus faecium*	Vancomycin	60	582 (415-749)
Southeast Asia, East Asia, and Oceania	1990	*Morganella* spp.	Fluoroquinolones	61	571 (293-849)
Southeast Asia, East Asia, and Oceania	1990	*Proteus* spp.	3GC	62	511 (252-771)
Southeast Asia, East Asia, and Oceania	1990	*Haemophilus influenzae*	3GC	63	495 (0-1,260)
Southeast Asia, East Asia, and Oceania	1990	*Citrobacter* spp.	Carbapenems	64	485 (219-751)
Southeast Asia, East Asia, and Oceania	1990	*Shigella* spp.	Fluoroquinolones	65	471 (0-973)

Southeast Asia, East Asia, and Oceania	1990	*Enterobacter* spp.	TMP-SMX	66	439 (71-807)
Southeast Asia, East Asia, and Oceania	1990	*Enterobacter faecalis*	Vancomycin	67	437 (0-1,080)
Southeast Asia, East Asia, and Oceania	1990	*Acinetobacter baumannii*	3GC	68	424 (361-486)
Southeast Asia, East Asia, and Oceania	1990	*Non-typhoidal Salmonella*	Fluoroquinolones	69	416 (0-974)
Southeast Asia, East Asia, and Oceania	1990	*Salmonella* Typhi	MDR	70	373 (1-745)
Southeast Asia, East Asia, and Oceania	1990	Group B *Streptococcus*	Penicillin	71	350 (28-672)
Southeast Asia, East Asia, and Oceania	1990	*Salmonella* Typhi	Fluoroquinolones	72	315 (22-607)
Southeast Asia, East Asia, and Oceania	1990	*Citrobacter* spp.	Aminoglycosides	73	246 (192-301)
Southeast Asia, East Asia, and Oceania	1990	*Proteus* spp.	Aminoglycosides	74	210 (121-300)
Southeast Asia, East Asia, and Oceania	1990	*Citrobacter* spp.	3GC	75	184 (30-339)
Southeast Asia, East Asia, and Oceania	1990	*Proteus* spp.	Fluoroquinolones	76	137 (3-272)
Southeast Asia, East Asia, and Oceania	1990	*Citrobacter* spp.	4GC	77	107 (84-130)
Southeast Asia, East Asia, and Oceania	1990	*Proteus* spp.	TMP-SMX	78	107 (0-286)
Southeast Asia, East Asia, and Oceania	1990	*Serratia* spp.	3GC	79	49 (0-137)
Southeast Asia, East Asia, and Oceania	1990	*Salmonella* Paratyphi	Fluoroquinolones	80	15 (0-33)
Southeast Asia, East Asia, and Oceania	1990	*Salmonella* Paratyphi	MDR	81	6 (0-36)
Southeast Asia, East Asia, and Oceania	1990	*Mycobacterium tuberculosis*	XDR	82	0 (0-0)
Sub-Saharan Africa	1990	*Streptococcus pneumoniae*	Carbapenems	1	24,500 (14,300-34,600)
Sub-Saharan Africa	1990	*Streptococcus pneumoniae*	Fluoroquinolones	2	10,800 (4,470-17,100)
Sub-Saharan Africa	1990	*Streptococcus pneumoniae*	TMP-SMX	3	9,150 (0-19,400)
Sub-Saharan Africa	1990	*Escherichia coli*	Beta-Lactams/Beta-Lactamase Inhib.	4	7,770 (607-14,900)
Sub-Saharan Africa	1990	*Escherichia coli*	TMP-SMX	5	7,580 (4,960-10,200)
Sub-Saharan Africa	1990	*Klebsiella pneumoniae*	Beta-Lactams/Beta-Lactamase Inhib.	6	7,010 (3,250-10,800)
Sub-Saharan Africa	1990	*Streptococcus pneumoniae*	3GC	7	6,740 (4,460-9,020)
Sub-Saharan Africa	1990	*Streptococcus pneumoniae*	Penicillin	8	6,580 (3,920-9,240)
Sub-Saharan Africa	1990	*Klebsiella pneumoniae*	TMP-SMX	9	6,570 (3,390-9,750)
Sub-Saharan Africa	1990	*Klebsiella pneumoniae*	3GC	10	6,250 (3,790-8,710)
Sub-Saharan Africa	1990	*Klebsiella pneumoniae*	Aminoglycosides	11	6,020 (4,190-7,850)
Sub-Saharan Africa	1990	*Pseudomonas aeruginosa*	Anti-pseudomonal	12	5,310 (3,970-6,650)
Sub-Saharan Africa	1990	*Staphylococcus aureus*	Methicillin	13	4,850 (2,170-7,520)
Sub-Saharan Africa	1990	*Acinetobacter baumannii*	Carbapenems	14	4,680 (2,800-6,570)
Sub-Saharan Africa	1990	*Klebsiella pneumoniae*	Fluoroquinolones	15	4,530 (2,980-6,070)
Sub-Saharan Africa	1990	*Acinetobacter baumannii*	Fluoroquinolones	16	4,480 (3,540-5,420)
Sub-Saharan Africa	1990	*Haemophilus influenzae*	3GC	17	4,460 (850-8,070)
Sub-Saharan Africa	1990	*Acinetobacter baumannii*	Anti-pseudomonal	18	4,420 (3,820-5,020)
Sub-Saharan Africa	1990	*Streptococcus pneumoniae*	Beta-Lactams/Beta-Lactamase Inhib.	19	4,150 (2,300-6,000)
Sub-Saharan Africa	1990	*Escherichia coli*	Aminoglycosides	20	3,760 (1,180-6,350)
Sub-Saharan Africa	1990	*Pseudomonas aeruginosa*	Aminoglycosides	21	3,680 (2,380-4,980)
Sub-Saharan Africa	1990	*Escherichia coli*	3GC	22	3,410 (0-6,920)
Sub-Saharan Africa	1990	*Klebsiella pneumoniae*	Carbapenems	23	3,360 (2,450-4,270)
Sub-Saharan Africa	1990	*Pseudomonas aeruginosa*	Fluoroquinolones	24	3,120 (2,140-4,110)
Sub-Saharan Africa	1990	*Streptococcus pneumoniae*	Macrolides	25	3,110 (1,610-4,610)
Sub-Saharan Africa	1990	*Staphylococcus aureus*	TMP-SMX	26	3,080 (1,870-4,290)
Sub-Saharan Africa	1990	*Escherichia coli*	Carbapenems	27	3,040 (694-5,390)
Sub-Saharan Africa	1990	*Acinetobacter baumannii*	3GC	28	3,020 (2,590-3,450)
Sub-Saharan Africa	1990	*Acinetobacter baumannii*	Aminoglycosides	29	3,020 (1,970-4,060)
Sub-Saharan Africa	1990	*Shigella* spp.	Fluoroquinolones	30	2,650 (262-5,050)
Sub-Saharan Africa	1990	*Escherichia coli*	Aminopenicillin	31	2,600 (830-4,360)
Sub-Saharan Africa	1990	*Acinetobacter baumannii*	4GC	32	2,270 (1,590-2,950)
Sub-Saharan Africa	1990	*Pseudomonas aeruginosa*	Carbapenems	33	2,250 (1,120-3,390)
Sub-Saharan Africa	1990	*Escherichia coli*	Fluoroquinolones	34	2,070 (870-3,280)
Sub-Saharan Africa	1990	*Serratia* spp.	Anti-pseudomonal	35	2,000 (1,510-2,500)
Sub-Saharan Africa	1990	*Acinetobacter baumannii*	Beta-Lactams/Beta-Lactamase Inhib.	36	1,920 (1,100-2,740)
Sub-Saharan Africa	1990	*Enterobacter* spp.	Carbapenems	37	1,630 (1,160-2,100)
Sub-Saharan Africa	1990	*Staphylococcus aureus*	Vancomycin	38	1,310 (461-2,150)
Sub-Saharan Africa	1990	Group B *Streptococcus*	Penicillin	39	1,280 (381-2,180)

Sub-Saharan Africa	1990	*Serratia* spp.	Aminoglycosides	40	1,280 (859-1,700)
Sub-Saharan Africa	1990	*Haemophilus influenzae*	Aminopenicillin	41	1,220 (751-1,680)
Sub-Saharan Africa	1990	*Serratia* spp.	4GC	42	1,180 (966-1,390)
Sub-Saharan Africa	1990	*Staphylococcus aureus*	Fluoroquinolones	43	1,140 (363-1,910)
Sub-Saharan Africa	1990	*Citrobacter* spp.	Anti-pseudomonal	44	1,030 (772-1,290)
Sub-Saharan Africa	1990	*Pseudomonas aeruginosa*	4GC	45	1,000 (543-1,460)
Sub-Saharan Africa	1990	*Enterobacter* spp.	4GC	46	961 (778-1,140)
Sub-Saharan Africa	1990	*Staphylococcus aureus*	Macrolides	47	921 (569-1,270)
Sub-Saharan Africa	1990	*Enterobacter* spp.	Aminoglycosides	48	882 (588-1,180)
Sub-Saharan Africa	1990	*Serratia* spp.	Fluoroquinolones	49	823 (340-1,310)
Sub-Saharan Africa	1990	Group B *Streptococcus*	Fluoroquinolones	50	811 (403-1,220)
Sub-Saharan Africa	1990	*Enterobacter* spp.	Anti-pseudomonal	51	803 (608-998)
Sub-Saharan Africa	1990	*Enterobacter faecalis*	Fluoroquinolones	52	768 (494-1,040)
Sub-Saharan Africa	1990	*Serratia* spp.	Carbapenems	53	685 (511-860)
Sub-Saharan Africa	1990	Group B *Streptococcus*	Macrolides	54	601 (380-822)
Sub-Saharan Africa	1990	*Non-typhoidal Salmonella*	Fluoroquinolones	55	559 (40-1,080)
Sub-Saharan Africa	1990	*Mycobacterium tuberculosis*	MDR excluding XDR	56	554 (0-1,470)
Sub-Saharan Africa	1990	*Enterobacter* spp.	Fluoroquinolones	57	526 (295-756)
Sub-Saharan Africa	1990	*Citrobacter* spp.	Fluoroquinolones	58	468 (237-699)
Sub-Saharan Africa	1990	*Proteus* spp.	3GC	59	441 (253-629)
Sub-Saharan Africa	1990	*Salmonella* Typhi	MDR	60	435 (0-952)
Sub-Saharan Africa	1990	*Morganella* spp.	Fluoroquinolones	61	412 (185-638)
Sub-Saharan Africa	1990	*Enterobacter* spp.	TMP-SMX	62	361 (62-659)
Sub-Saharan Africa	1990	*Pseudomonas aeruginosa*	3GC	63	343 (0-726)
Sub-Saharan Africa	1990	*Enterococcus faecium*	Fluoroquinolones	64	339 (145-533)
Sub-Saharan Africa	1990	Group A *Streptococcus*	Macrolides	65	310 (191-428)
Sub-Saharan Africa	1990	*Morganella* spp.	3GC	66	298 (207-390)
Sub-Saharan Africa	1990	*Proteus* spp.	Aminopenicillin	67	284 (201-367)
Sub-Saharan Africa	1990	*Citrobacter* spp.	Aminoglycosides	68	271 (200-342)
Sub-Saharan Africa	1990	*Enterobacter faecalis*	Vancomycin	69	263 (0-645)
Sub-Saharan Africa	1990	*Citrobacter* spp.	Carbapenems	70	261 (123-400)
Sub-Saharan Africa	1990	*Citrobacter* spp.	4GC	71	252 (188-316)
Sub-Saharan Africa	1990	*Enterococcus faecium*	Vancomycin	72	250 (190-310)
Sub-Saharan Africa	1990	*Morganella* spp.	4GC	73	243 (148-338)
Sub-Saharan Africa	1990	*Proteus* spp.	Aminoglycosides	74	208 (124-292)
Sub-Saharan Africa	1990	*Salmonella* Typhi	Fluoroquinolones	75	181 (2-359)
Sub-Saharan Africa	1990	*Serratia* spp.	3GC	76	115 (0-292)
Sub-Saharan Africa	1990	*Citrobacter* spp.	3GC	77	85 (14-155)
Sub-Saharan Africa	1990	*Proteus* spp.	Fluoroquinolones	78	74 (3-146)
Sub-Saharan Africa	1990	*Proteus* spp.	TMP-SMX	79	55 (0-149)
Sub-Saharan Africa	1990	*Salmonella* Paratyphi	MDR	80	9 (0-64)
Sub-Saharan Africa	1990	*Salmonella* Paratyphi	Fluoroquinolones	81	8 (0-17)
Sub-Saharan Africa	1990	*Mycobacterium tuberculosis*	XDR	82	0 (0-0)

Table S14: 82 fatal pathogen-drug combination ranking by burden attributable to AMR in 2021, globally and by super-regions

Location	Year	Pathogen	Antibiotic Class	Rank	Attributable AMR Deaths
Global	2021	*Staphylococcus aureus*	Methicillin	1	130,000 (113,000-146,000)
Global	2021	*Acinetobacter baumannii*	Carbapenems	2	78,100 (62,400-93,900)
Global	2021	*Streptococcus pneumoniae*	Carbapenems	3	71,600 (51,000-92,100)
Global	2021	*Pseudomonas aeruginosa*	Carbapenems	4	45,600 (31,600-59,700)
Global	2021	*Klebsiella pneumoniae*	Carbapenems	5	45,600 (35,700-55,500)
Global	2021	*Acinetobacter baumannii*	Fluoroquinolones	6	45,000 (36,800-53,300)
Global	2021	*Mycobacterium tuberculosis*	MDR excluding XDR	7	36,400 (0-86,800)
Global	2021	*Klebsiella pneumoniae*	Fluoroquinolones	8	36,000 (25,500-46,400)
Global	2021	*Escherichia coli*	Fluoroquinolones	9	34,100 (22,900-45,400)
Global	2021	*Escherichia coli*	3GC	10	33,100 (22,500-43,700)
Global	2021	*Escherichia coli*	Carbapenems	11	27,900 (22,600-33,200)
Global	2021	*Klebsiella pneumoniae*	Aminoglycosides	12	27,900 (20,900-34,900)
Global	2021	*Staphylococcus aureus*	Fluoroquinolones	13	25,600 (10,100-41,200)
Global	2021	*Acinetobacter baumannii*	Aminoglycosides	14	24,200 (18,800-29,600)
Global	2021	*Escherichia coli*	TMP-SMX	15	24,100 (15,100-33,200)
Global	2021	*Pseudomonas aeruginosa*	Fluoroquinolones	16	23,800 (16,400-31,300)
Global	2021	*Staphylococcus aureus*	Macrolides	17	21,600 (14,400-28,700)
Global	2021	*Klebsiella pneumoniae*	3GC	18	21,200 (13,400-29,100)
Global	2021	*Streptococcus pneumoniae*	Macrolides	19	20,000 (12,300-27,800)
Global	2021	*Klebsiella pneumoniae*	TMP-SMX	20	18,300 (9,230-27,300)
Global	2021	*Streptococcus pneumoniae*	3GC	21	16,300 (11,500-21,100)
Global	2021	*Pseudomonas aeruginosa*	Anti-pseudomonal	22	16,000 (13,100-18,900)
Global	2021	*Streptococcus pneumoniae*	TMP-SMX	23	15,500 (0-33,400)
Global	2021	*Escherichia coli*	Aminopenicillin	24	14,900 (11,400-18,300)
Global	2021	*Escherichia coli*	Beta-Lactams/Beta-Lactamase Inhib.	25	14,800 (5,020-24,500)
Global	2021	*Pseudomonas aeruginosa*	Aminoglycosides	26	14,200 (9,980-18,400)
Global	2021	*Streptococcus pneumoniae*	Penicillin	27	13,300 (10,400-16,100)
Global	2021	*Streptococcus pneumoniae*	Fluoroquinolones	28	13,200 (5,550-20,800)
Global	2021	*Enterobacter faecalis*	Fluoroquinolones	29	12,400 (8,300-16,500)
Global	2021	*Staphylococcus aureus*	TMP-SMX	30	12,300 (7,450-17,200)
Global	2021	*Escherichia coli*	Aminoglycosides	31	11,800 (8,110-15,500)
Global	2021	*Enterococcus faecium*	Vancomycin	32	11,000 (9,090-13,000)
Global	2021	*Enterococcus faecium*	Fluoroquinolones	33	10,500 (5,520-15,400)
Global	2021	*Enterobacter* spp.	Carbapenems	34	9,780 (7,640-11,900)
Global	2021	*Klebsiella pneumoniae*	Beta-Lactams/Beta-Lactamase Inhib.	35	9,380 (4,130-14,600)
Global	2021	*Acinetobacter baumannii*	Anti-pseudomonal	36	8,370 (6,890-9,860)
Global	2021	*Enterobacter* spp.	Anti-pseudomonal	37	7,470 (6,210-8,740)
Global	2021	*Acinetobacter baumannii*	3GC	38	7,460 (6,220-8,690)
Global	2021	*Staphylococcus aureus*	Vancomycin	39	7,110 (5,000-9,220)
Global	2021	*Serratia* spp.	Anti-pseudomonal	40	5,520 (4,440-6,590)
Global	2021	*Serratia* spp.	Carbapenems	41	5,160 (4,010-6,310)
Global	2021	*Streptococcus pneumoniae*	Beta-Lactams/Beta-Lactamase Inhib.	42	5,160 (2,870-7,450)
Global	2021	*Enterobacter* spp.	Fluoroquinolones	43	5,040 (2,860-7,220)
Global	2021	*Haemophilus influenzae*	Aminopenicillin	44	4,770 (3,660-5,880)
Global	2021	*Pseudomonas aeruginosa*	4GC	45	4,360 (3,340-5,380)
Global	2021	*Proteus* spp.	3GC	46	4,230 (2,820-5,650)
Global	2021	*Proteus* spp.	Aminopenicillin	47	4,210 (3,330-5,090)
Global	2021	Group B *Streptococcus*	Fluoroquinolones	48	4,160 (1,920-6,390)
Global	2021	*Shigella* spp.	Fluoroquinolones	49	3,960 (748-7,180)
Global	2021	*Serratia* spp.	Aminoglycosides	50	3,780 (2,550-5,010)
Global	2021	*Enterobacter* spp.	4GC	51	3,720 (3,110-4,330)
Global	2021	*Citrobacter* spp.	Carbapenems	52	3,650 (2,180-5,130)
Global	2021	*Enterobacter* spp.	Aminoglycosides	53	3,590 (2,490-4,700)
Global	2021	*Morganella* spp.	Fluoroquinolones	54	3,590 (1,850-5,320)
Global	2021	*Citrobacter* spp.	Anti-pseudomonal	55	3,490 (2,910-4,060)
Global	2021	*Citrobacter* spp.	Fluoroquinolones	56	3,300 (2,030-4,580)

Global	2021	*Acinetobacter baumannii*	4GC	57	3,290 (2,770-3,810)
Global	2021	*Salmonella* Typhi	Fluoroquinolones	58	3,260 (464-6,050)
Global	2021	*Mycobacterium tuberculosis*	XDR	59	3,250 (176-6,330)
Global	2021	*Haemophilus influenzae*	3GC	60	3,190 (538-5,840)
Global	2021	*Enterobacter faecalis*	Vancomycin	61	2,990 (1,600-4,380)
Global	2021	*Acinetobacter baumannii*	Beta-Lactams/Beta-Lactamase Inhib.	62	2,860 (1,870-3,860)
Global	2021	Group B *Streptococcus*	Macrolides	63	2,720 (1,720-3,710)
Global	2021	*Morganella* spp.	4GC	64	2,710 (1,980-3,450)
Global	2021	Group A *Streptococcus*	Macrolides	65	2,700 (1,640-3,750)
Global	2021	*Proteus* spp.	Aminoglycosides	66	2,680 (1,870-3,490)
Global	2021	*Salmonella* Typhi	MDR	67	2,680 (181-5,180)
Global	2021	*Proteus* spp.	Fluoroquinolones	68	2,570 (1,050-4,100)
Global	2021	*Serratia* spp.	Fluoroquinolones	69	2,490 (779-4,200)
Global	2021	*Serratia* spp.	4GC	70	2,300 (1,890-2,710)
Global	2021	*Pseudomonas aeruginosa*	3GC	71	2,190 (1,290-3,080)
Global	2021	*Non-typhoidal Salmonella*	Fluoroquinolones	72	1,950 (160-3,740)
Global	2021	*Enterobacter* spp.	TMP-SMX	73	1,900 (246-3,550)
Global	2021	*Salmonella* Paratyphi	Fluoroquinolones	74	1,670 (270-3,070)
Global	2021	Group B *Streptococcus*	Penicillin	75	1,390 (480-2,300)
Global	2021	*Morganella* spp.	3GC	76	1,260 (918-1,600)
Global	2021	*Citrobacter* spp.	Aminoglycosides	77	1,070 (828-1,310)
Global	2021	*Citrobacter* spp.	4GC	78	996 (808-1,180)
Global	2021	*Citrobacter* spp.	3GC	79	814 (523-1,110)
Global	2021	*Proteus* spp.	TMP-SMX	80	735 (0-1,890)
Global	2021	*Serratia* spp.	3GC	81	535 (47-1,020)
Global	2021	*Salmonella* Paratyphi	MDR	82	110 (0-233)
Central Europe, Eastern Europe, and Central Asia	2021	*Staphylococcus aureus*	Methicillin	1	7,700 (6,070-9,330)
Central Europe, Eastern Europe, and Central Asia	2021	*Pseudomonas aeruginosa*	Carbapenems	2	3,680 (2,560-4,800)
Central Europe, Eastern Europe, and Central Asia	2021	*Acinetobacter baumannii*	Carbapenems	3	3,580 (2,860-4,310)
Central Europe, Eastern Europe, and Central Asia	2021	*Streptococcus pneumoniae*	Carbapenems	4	3,370 (2,220-4,520)
Central Europe, Eastern Europe, and Central Asia	2021	*Klebsiella pneumoniae*	Carbapenems	5	2,700 (2,040-3,350)
Central Europe, Eastern Europe, and Central Asia	2021	*Escherichia coli*	Fluoroquinolones	6	2,360 (1,310-3,410)
Central Europe, Eastern Europe, and Central Asia	2021	*Klebsiella pneumoniae*	Fluoroquinolones	7	2,350 (1,670-3,030)
Central Europe, Eastern Europe, and Central Asia	2021	*Escherichia coli*	3GC	8	2,170 (1,420-2,910)
Central Europe, Eastern Europe, and Central Asia	2021	*Acinetobacter baumannii*	Fluoroquinolones	9	2,100 (1,720-2,490)
Central Europe, Eastern Europe, and Central Asia	2021	*Pseudomonas aeruginosa*	Fluoroquinolones	10	1,930 (1,350-2,510)
Central Europe, Eastern Europe, and Central Asia	2021	*Mycobacterium tuberculosis*	MDR excluding XDR	11	1,920 (0-4,520)
Central Europe, Eastern Europe, and Central Asia	2021	*Escherichia coli*	Aminopenicillin	12	1,730 (1,200-2,250)
Central Europe, Eastern Europe, and Central Asia	2021	*Klebsiella pneumoniae*	Aminoglycosides	13	1,720 (1,310-2,140)
Central Europe, Eastern Europe, and Central Asia	2021	*Escherichia coli*	TMP-SMX	14	1,540 (859-2,220)
Central Europe, Eastern Europe, and Central Asia	2021	*Staphylococcus aureus*	Fluoroquinolones	15	1,280 (499-2,060)
Central Europe, Eastern Europe, and Central Asia	2021	*Enterobacter faecalis*	Fluoroquinolones	16	1,220 (797-1,640)
Central Europe, Eastern Europe, and Central Asia	2021	*Escherichia coli*	Beta-Lactams/Beta-Lactamase Inhib.	17	1,130 (306-1,950)
Central Europe, Eastern Europe, and Central Asia	2021	*Staphylococcus aureus*	Macrolides	18	1,060 (715-1,410)
Central Europe, Eastern Europe, and Central Asia	2021	*Pseudomonas aeruginosa*	Aminoglycosides	19	1,040 (716-1,370)
Central Europe, Eastern Europe, and Central Asia	2021	*Klebsiella pneumoniae*	TMP-SMX	20	1,020 (512-1,520)
Central Europe, Eastern Europe, and Central Asia	2021	*Acinetobacter baumannii*	Aminoglycosides	21	991 (755-1,230)
Central Europe, Eastern Europe, and Central Asia	2021	*Enterococcus faecium*	Fluoroquinolones	22	963 (501-1,420)
Central Europe, Eastern Europe, and Central Asia	2021	*Mycobacterium tuberculosis*	XDR	23	940 (30-1,850)
Central Europe, Eastern Europe, and Central Asia	2021	*Klebsiella pneumoniae*	3GC	24	906 (545-1,270)
Central Europe, Eastern Europe, and Central Asia	2021	*Pseudomonas aeruginosa*	Anti-pseudomonal	25	874 (732-1,020)
Central Europe, Eastern Europe, and Central Asia	2021	*Escherichia coli*	Carbapenems	26	845 (666-1,020)
Central Europe, Eastern Europe, and Central Asia	2021	*Enterococcus faecium*	Vancomycin	27	794 (627-961)
Central Europe, Eastern Europe, and Central Asia	2021	*Streptococcus pneumoniae*	TMP-SMX	28	757 (0-1,660)
Central Europe, Eastern Europe, and Central Asia	2021	*Enterobacter* spp.	Anti-pseudomonal	29	754 (626-882)
Central Europe, Eastern Europe, and Central Asia	2021	*Escherichia coli*	Aminoglycosides	30	689 (463-914)
Central Europe, Eastern Europe, and Central Asia	2021	*Staphylococcus aureus*	Vancomycin	31	577 (463-691)
Central Europe, Eastern Europe, and Central Asia	2021	*Streptococcus pneumoniae*	Macrolides	32	572 (337-807)
Central Europe, Eastern Europe, and Central Asia	2021	*Enterobacter* spp.	Carbapenems	33	444 (326-562)
Central Europe, Eastern Europe, and Central Asia	2021	*Streptococcus pneumoniae*	Penicillin	34	387 (312-461)

Central Europe, Eastern Europe, and Central Asia	2021	*Serratia* spp.	Anti-pseudomonal	35	358 (296-420)
Central Europe, Eastern Europe, and Central Asia	2021	*Enterobacter* spp.	Fluoroquinolones	36	335 (187-484)
Central Europe, Eastern Europe, and Central Asia	2021	*Staphylococcus aureus*	TMP-SMX	37	330 (187-473)
Central Europe, Eastern Europe, and Central Asia	2021	*Klebsiella pneumoniae*	Beta-Lactams/Beta-Lactamase Inhib.	38	330 (114-546)
Central Europe, Eastern Europe, and Central Asia	2021	*Enterobacter* spp.	4GC	39	317 (265-368)
Central Europe, Eastern Europe, and Central Asia	2021	*Proteus* spp.	Aminopenicillin	40	289 (222-355)
Central Europe, Eastern Europe, and Central Asia	2021	*Streptococcus pneumoniae*	Beta-Lactams/Beta-Lactamase Inhib.	41	288 (160-415)
Central Europe, Eastern Europe, and Central Asia	2021	*Citrobacter* spp.	Anti-pseudomonal	42	275 (233-317)
Central Europe, Eastern Europe, and Central Asia	2021	*Enterobacter faecalis*	Vancomycin	43	261 (134-388)
Central Europe, Eastern Europe, and Central Asia	2021	*Streptococcus pneumoniae*	3GC	44	254 (185-324)
Central Europe, Eastern Europe, and Central Asia	2021	*Proteus* spp.	Aminoglycosides	45	248 (164-331)
Central Europe, Eastern Europe, and Central Asia	2021	*Citrobacter* spp.	Fluoroquinolones	46	243 (133-353)
Central Europe, Eastern Europe, and Central Asia	2021	*Enterobacter* spp.	Aminoglycosides	47	239 (156-321)
Central Europe, Eastern Europe, and Central Asia	2021	*Serratia* spp.	Carbapenems	48	239 (188-290)
Central Europe, Eastern Europe, and Central Asia	2021	*Proteus* spp.	Fluoroquinolones	49	237 (73-400)
Central Europe, Eastern Europe, and Central Asia	2021	*Serratia* spp.	Aminoglycosides	50	233 (151-316)
Central Europe, Eastern Europe, and Central Asia	2021	*Streptococcus pneumoniae*	Fluoroquinolones	51	225 (88-362)
Central Europe, Eastern Europe, and Central Asia	2021	*Morganella* spp.	Fluoroquinolones	52	224 (113-335)
Central Europe, Eastern Europe, and Central Asia	2021	*Acinetobacter baumannii*	3GC	53	224 (165-282)
Central Europe, Eastern Europe, and Central Asia	2021	*Acinetobacter baumannii*	Anti-pseudomonal	54	211 (144-279)
Central Europe, Eastern Europe, and Central Asia	2021	*Serratia* spp.	4GC	55	211 (180-242)
Central Europe, Eastern Europe, and Central Asia	2021	*Proteus* spp.	3GC	56	204 (127-281)
Central Europe, Eastern Europe, and Central Asia	2021	*Pseudomonas aeruginosa*	4GC	57	200 (158-243)
Central Europe, Eastern Europe, and Central Asia	2021	*Serratia* spp.	Fluoroquinolones	58	165 (50-281)
Central Europe, Eastern Europe, and Central Asia	2021	*Enterobacter* spp.	TMP-SMX	59	162 (15-309)
Central Europe, Eastern Europe, and Central Asia	2021	*Citrobacter* spp.	4GC	60	146 (124-167)
Central Europe, Eastern Europe, and Central Asia	2021	*Citrobacter* spp.	Carbapenems	61	138 (75-200)
Central Europe, Eastern Europe, and Central Asia	2021	*Pseudomonas aeruginosa*	3GC	62	132 (73-191)
Central Europe, Eastern Europe, and Central Asia	2021	*Haemophilus influenzae*	3GC	63	125 (4-246)
Central Europe, Eastern Europe, and Central Asia	2021	Group A *Streptococcus*	Macrolides	64	125 (76-174)
Central Europe, Eastern Europe, and Central Asia	2021	*Acinetobacter baumannii*	4GC	65	120 (91-149)
Central Europe, Eastern Europe, and Central Asia	2021	*Haemophilus influenzae*	Aminopenicillin	66	112 (78-145)
Central Europe, Eastern Europe, and Central Asia	2021	*Morganella* spp.	4GC	67	106 (70-142)
Central Europe, Eastern Europe, and Central Asia	2021	Group B *Streptococcus*	Macrolides	68	105 (67-142)
Central Europe, Eastern Europe, and Central Asia	2021	Group B *Streptococcus*	Fluoroquinolones	69	96 (37-156)
Central Europe, Eastern Europe, and Central Asia	2021	*Citrobacter* spp.	Aminoglycosides	70	96 (76-116)
Central Europe, Eastern Europe, and Central Asia	2021	*Proteus* spp.	TMP-SMX	71	72 (0-191)
Central Europe, Eastern Europe, and Central Asia	2021	*Morganella* spp.	3GC	72	62 (45-79)
Central Europe, Eastern Europe, and Central Asia	2021	Group B *Streptococcus*	Penicillin	73	42 (6-77)
Central Europe, Eastern Europe, and Central Asia	2021	*Citrobacter* spp.	3GC	74	36 (10-62)
Central Europe, Eastern Europe, and Central Asia	2021	*Serratia* spp.	3GC	75	31 (0-73)
Central Europe, Eastern Europe, and Central Asia	2021	*Acinetobacter baumannii*	Beta-Lactams/Beta-Lactamase Inhib.	76	25 (13-36)
Central Europe, Eastern Europe, and Central Asia	2021	*Shigella* spp.	Fluoroquinolones	77	11 (0-24)
Central Europe, Eastern Europe, and Central Asia	2021	*Non-typhoidal Salmonella*	Fluoroquinolones	78	2 (0-4)
Central Europe, Eastern Europe, and Central Asia	2021	*Salmonella* Typhi	MDR	79	0 (0-0)
Central Europe, Eastern Europe, and Central Asia	2021	*Salmonella* Paratyphi	Fluoroquinolones	80	0 (0-0)
Central Europe, Eastern Europe, and Central Asia	2021	*Salmonella* Typhi	Fluoroquinolones	81	0 (0-0)
Central Europe, Eastern Europe, and Central Asia	2021	*Salmonella* Paratyphi	MDR	82	0 (0-0)
High-income	2021	*Staphylococcus aureus*	Methicillin	1	31,000 (26,300-35,800)
High-income	2021	*Pseudomonas aeruginosa*	Carbapenems	2	6,540 (4,630-8,450)
High-income	2021	*Staphylococcus aureus*	Fluoroquinolones	3	5,790 (2,440-9,150)
High-income	2021	*Streptococcus pneumoniae*	Carbapenems	4	5,560 (4,420-6,700)
High-income	2021	*Staphylococcus aureus*	Macrolides	5	5,000 (3,340-6,670)
High-income	2021	*Escherichia coli*	Fluoroquinolones	6	4,760 (2,830-6,680)
High-income	2021	*Escherichia coli*	Aminopenicillin	7	4,450 (3,440-5,460)
High-income	2021	*Acinetobacter baumannii*	Carbapenems	8	3,870 (3,110-4,640)
High-income	2021	*Escherichia coli*	Beta-Lactams/Beta-Lactamase Inhib.	9	3,820 (1,190-6,450)
High-income	2021	*Enterococcus faecium*	Vancomycin	10	3,550 (2,990-4,100)
High-income	2021	*Escherichia coli*	3GC	11	3,480 (2,280-4,680)
High-income	2021	*Pseudomonas aeruginosa*	Fluoroquinolones	12	3,190 (2,200-4,190)

High-income	2021	*Escherichia coli*	TMP-SMX	13	2,950 (1,640-4,270)
High-income	2021	*Enterococcus faecium*	Fluoroquinolones	14	2,520 (1,410-3,630)
High-income	2021	*Klebsiella pneumoniae*	Aminoglycosides	15	2,380 (1,660-3,110)
High-income	2021	*Acinetobacter baumannii*	Fluoroquinolones	16	2,370 (1,940-2,810)
High-income	2021	*Klebsiella pneumoniae*	Fluoroquinolones	17	2,060 (1,460-2,670)
High-income	2021	*Klebsiella pneumoniae*	Carbapenems	18	1,980 (1,540-2,430)
High-income	2021	*Pseudomonas aeruginosa*	Anti-pseudomonal	19	1,850 (1,490-2,200)
High-income	2021	*Streptococcus pneumoniae*	Macrolides	20	1,590 (959-2,230)
High-income	2021	*Enterobacter* spp.	Anti-pseudomonal	21	1,590 (1,290-1,890)
High-income	2021	*Staphylococcus aureus*	Vancomycin	22	1,580 (1,250-1,900)
High-income	2021	*Escherichia coli*	Carbapenems	23	1,350 (1,100-1,600)
High-income	2021	*Enterobacter faecalis*	Fluoroquinolones	24	1,240 (802-1,670)
High-income	2021	*Klebsiella pneumoniae*	TMP-SMX	25	1,060 (541-1,570)
High-income	2021	*Acinetobacter baumannii*	Aminoglycosides	26	1,050 (817-1,280)
High-income	2021	*Proteus* spp.	Aminopenicillin	27	1,020 (769-1,270)
High-income	2021	*Klebsiella pneumoniae*	3GC	28	1,020 (603-1,430)
High-income	2021	*Escherichia coli*	Aminoglycosides	29	1,010 (644-1,380)
High-income	2021	*Pseudomonas aeruginosa*	Aminoglycosides	30	969 (708-1,230)
High-income	2021	*Klebsiella pneumoniae*	Beta-Lactams/Beta-Lactamase Inhib.	31	875 (402-1,350)
High-income	2021	*Haemophilus influenzae*	Aminopenicillin	32	797 (628-965)
High-income	2021	*Streptococcus pneumoniae*	TMP-SMX	33	765 (0-1,680)
High-income	2021	*Enterobacter* spp.	Carbapenems	34	756 (606-905)
High-income	2021	*Staphylococcus aureus*	TMP-SMX	35	675 (428-922)
High-income	2021	*Citrobacter* spp.	Anti-pseudomonal	36	570 (462-678)
High-income	2021	*Acinetobacter baumannii*	Anti-pseudomonal	37	544 (455-633)
High-income	2021	*Streptococcus pneumoniae*	Penicillin	38	528 (407-649)
High-income	2021	*Streptococcus pneumoniae*	Fluoroquinolones	39	507 (291-724)
High-income	2021	*Proteus* spp.	3GC	40	494 (328-659)
High-income	2021	*Acinetobacter baumannii*	3GC	41	489 (410-568)
High-income	2021	*Enterobacter faecalis*	Vancomycin	42	455 (303-606)
High-income	2021	*Morganella* spp.	Fluoroquinolones	43	425 (204-645)
High-income	2021	*Proteus* spp.	Fluoroquinolones	44	406 (163-650)
High-income	2021	*Enterobacter* spp.	Fluoroquinolones	45	385 (220-549)
High-income	2021	*Serratia* spp.	Anti-pseudomonal	46	377 (296-458)
High-income	2021	*Serratia* spp.	Carbapenems	47	351 (271-430)
High-income	2021	Group B *Streptococcus*	Macrolides	48	312 (197-427)
High-income	2021	*Serratia* spp.	Fluoroquinolones	49	300 (104-496)
High-income	2021	*Citrobacter* spp.	Fluoroquinolones	50	287 (185-388)
High-income	2021	*Pseudomonas aeruginosa*	4GC	51	276 (216-336)
High-income	2021	Group A *Streptococcus*	Macrolides	52	273 (166-381)
High-income	2021	*Proteus* spp.	Aminoglycosides	53	261 (187-335)
High-income	2021	Group B *Streptococcus*	Fluoroquinolones	54	240 (132-347)
High-income	2021	*Citrobacter* spp.	Carbapenems	55	232 (142-321)
High-income	2021	*Morganella* spp.	3GC	56	207 (150-265)
High-income	2021	*Serratia* spp.	Aminoglycosides	57	196 (129-264)
High-income	2021	*Citrobacter* spp.	3GC	58	179 (123-236)
High-income	2021	*Enterobacter* spp.	Aminoglycosides	59	179 (123-234)
High-income	2021	*Morganella* spp.	4GC	60	166 (113-218)
High-income	2021	*Enterobacter* spp.	TMP-SMX	61	160 (22-299)
High-income	2021	*Acinetobacter baumannii*	4GC	62	146 (101-192)
High-income	2021	*Streptococcus pneumoniae*	Beta-Lactams/Beta-Lactamase Inhib.	63	145 (66-224)
High-income	2021	*Acinetobacter baumannii*	Beta-Lactams/Beta-Lactamase Inhib.	64	119 (84-154)
High-income	2021	*Mycobacterium tuberculosis*	MDR excluding XDR	65	115 (0-310)
High-income	2021	*Pseudomonas aeruginosa*	3GC	66	111 (55-168)
High-income	2021	*Proteus* spp.	TMP-SMX	67	111 (0-287)
High-income	2021	*Enterobacter* spp.	4GC	68	106 (85-127)
High-income	2021	*Haemophilus influenzae*	3GC	69	102 (54-150)
High-income	2021	*Streptococcus pneumoniae*	3GC	70	102 (63-140)
High-income	2021	Group B *Streptococcus*	Penicillin	71	74 (58-90)
High-income	2021	*Serratia* spp.	3GC	72	68 (0-150)

High-income	2021	*Citrobacter* spp.	Aminoglycosides	73	61 (47-74)
High-income	2021	*Serratia* spp.	4GC	74	52 (42-62)
High-income	2021	*Mycobacterium tuberculosis*	XDR	75	38 (0-81)
High-income	2021	*Citrobacter* spp.	4GC	76	30 (23-37)
High-income	2021	*Non-typhoidal Salmonella*	Fluoroquinolones	77	12 (0-36)
High-income	2021	*Shigella* spp.	Fluoroquinolones	78	4 (1-6)
High-income	2021	*Salmonella* Paratyphi	Fluoroquinolones	79	0 (0-0)
High-income	2021	*Salmonella* Typhi	MDR	80	0 (0-0)
High-income	2021	*Salmonella* Typhi	Fluoroquinolones	81	0 (0-0)
High-income	2021	*Salmonella* Paratyphi	MDR	82	0 (0-0)
Latin America and Caribbean	2021	*Staphylococcus aureus*	Methicillin	1	9,240 (7,620-10,900)
Latin America and Caribbean	2021	*Acinetobacter baumannii*	Carbapenems	2	5,970 (4,850-7,100)
Latin America and Caribbean	2021	*Streptococcus pneumoniae*	Carbapenems	3	5,170 (3,550-6,790)
Latin America and Caribbean	2021	*Pseudomonas aeruginosa*	Carbapenems	4	4,870 (3,450-6,290)
Latin America and Caribbean	2021	*Klebsiella pneumoniae*	Carbapenems	5	3,800 (2,990-4,610)
Latin America and Caribbean	2021	*Acinetobacter baumannii*	Fluoroquinolones	6	3,380 (2,760-4,000)
Latin America and Caribbean	2021	*Klebsiella pneumoniae*	Fluoroquinolones	7	3,100 (2,190-4,010)
Latin America and Caribbean	2021	*Klebsiella pneumoniae*	Aminoglycosides	8	2,530 (1,880-3,180)
Latin America and Caribbean	2021	*Escherichia coli*	Fluoroquinolones	9	2,490 (1,490-3,500)
Latin America and Caribbean	2021	*Pseudomonas aeruginosa*	Fluoroquinolones	10	2,140 (1,470-2,810)
Latin America and Caribbean	2021	*Escherichia coli*	3GC	11	2,050 (1,310-2,800)
Latin America and Caribbean	2021	*Staphylococcus aureus*	Macrolides	12	2,020 (1,340-2,690)
Latin America and Caribbean	2021	*Escherichia coli*	TMP-SMX	13	1,890 (1,080-2,710)
Latin America and Caribbean	2021	*Acinetobacter baumannii*	Aminoglycosides	14	1,680 (1,300-2,060)
Latin America and Caribbean	2021	*Enterococcus faecium*	Vancomycin	15	1,550 (1,290-1,810)
Latin America and Caribbean	2021	*Klebsiella pneumoniae*	TMP-SMX	16	1,510 (762-2,260)
Latin America and Caribbean	2021	*Klebsiella pneumoniae*	3GC	17	1,450 (887-2,020)
Latin America and Caribbean	2021	*Staphylococcus aureus*	Fluoroquinolones	18	1,400 (549-2,250)
Latin America and Caribbean	2021	*Escherichia coli*	Aminopenicillin	19	1,380 (999-1,760)
Latin America and Caribbean	2021	*Pseudomonas aeruginosa*	Aminoglycosides	20	1,060 (777-1,340)
Latin America and Caribbean	2021	*Streptococcus pneumoniae*	Macrolides	21	1,010 (591-1,430)
Latin America and Caribbean	2021	*Streptococcus pneumoniae*	TMP-SMX	22	1,000 (0-2,180)
Latin America and Caribbean	2021	*Escherichia coli*	Beta-Lactams/Beta-Lactamase Inhib.	23	994 (144-1,840)
Latin America and Caribbean	2021	*Escherichia coli*	Carbapenems	24	985 (730-1,240)
Latin America and Caribbean	2021	*Pseudomonas aeruginosa*	Anti-pseudomonal	25	969 (791-1,150)
Latin America and Caribbean	2021	*Escherichia coli*	Aminoglycosides	26	767 (487-1,050)
Latin America and Caribbean	2021	*Enterobacter faecalis*	Fluoroquinolones	27	733 (504-962)
Latin America and Caribbean	2021	*Streptococcus pneumoniae*	Penicillin	28	727 (498-955)
Latin America and Caribbean	2021	*Enterococcus faecium*	Fluoroquinolones	29	714 (395-1,030)
Latin America and Caribbean	2021	*Proteus* spp.	Aminopenicillin	30	629 (503-756)
Latin America and Caribbean	2021	*Enterobacter* spp.	Anti-pseudomonal	31	617 (507-727)
Latin America and Caribbean	2021	*Staphylococcus aureus*	Vancomycin	32	572 (453-692)
Latin America and Caribbean	2021	*Enterobacter* spp.	Carbapenems	33	554 (440-667)
Latin America and Caribbean	2021	*Klebsiella pneumoniae*	Beta-Lactams/Beta-Lactamase Inhib.	34	503 (160-846)
Latin America and Caribbean	2021	*Staphylococcus aureus*	TMP-SMX	35	425 (265-586)
Latin America and Caribbean	2021	*Serratia* spp.	Carbapenems	36	423 (335-512)
Latin America and Caribbean	2021	*Mycobacterium tuberculosis*	MDR excluding XDR	37	417 (0-1,110)
Latin America and Caribbean	2021	*Acinetobacter baumannii*	Anti-pseudomonal	38	414 (342-485)
Latin America and Caribbean	2021	*Streptococcus pneumoniae*	Fluoroquinolones	39	408 (124-692)
Latin America and Caribbean	2021	*Acinetobacter baumannii*	3GC	40	378 (309-447)
Latin America and Caribbean	2021	*Enterobacter faecalis*	Vancomycin	41	359 (178-539)
Latin America and Caribbean	2021	*Enterobacter* spp.	Fluoroquinolones	42	358 (210-507)
Latin America and Caribbean	2021	*Morganella* spp.	Fluoroquinolones	43	311 (156-466)
Latin America and Caribbean	2021	*Serratia* spp.	Anti-pseudomonal	44	305 (243-366)
Latin America and Caribbean	2021	*Streptococcus pneumoniae*	3GC	45	289 (209-369)
Latin America and Caribbean	2021	*Citrobacter* spp.	Anti-pseudomonal	46	269 (225-313)
Latin America and Caribbean	2021	*Enterobacter* spp.	Aminoglycosides	47	255 (165-344)
Latin America and Caribbean	2021	*Proteus* spp.	3GC	48	250 (150-350)
Latin America and Caribbean	2021	*Citrobacter* spp.	Fluoroquinolones	49	241 (138-343)
Latin America and Caribbean	2021	*Serratia* spp.	Fluoroquinolones	50	232 (81-383)

Latin America and Caribbean	2021	*Enterobacter* spp.	4GC	51	230 (186-273)
Latin America and Caribbean	2021	*Haemophilus influenzae*	Aminopenicillin	52	222 (155-288)
Latin America and Caribbean	2021	*Pseudomonas aeruginosa*	4GC	53	213 (164-261)
Latin America and Caribbean	2021	*Serratia* spp.	Aminoglycosides	54	205 (138-272)
Latin America and Caribbean	2021	*Streptococcus pneumoniae*	Beta-Lactams/Beta-Lactamase Inhib.	55	194 (87-301)
Latin America and Caribbean	2021	*Proteus* spp.	Fluoroquinolones	56	186 (62-310)
Latin America and Caribbean	2021	*Pseudomonas aeruginosa*	3GC	57	176 (65-287)
Latin America and Caribbean	2021	*Proteus* spp.	Aminoglycosides	58	173 (112-235)
Latin America and Caribbean	2021	*Enterobacter* spp.	TMP-SMX	59	170 (17-324)
Latin America and Caribbean	2021	*Citrobacter* spp.	Carbapenems	60	157 (96-218)
Latin America and Caribbean	2021	*Morganella* spp.	4GC	61	150 (102-198)
Latin America and Caribbean	2021	Group B *Streptococcus*	Macrolides	62	148 (92-204)
Latin America and Caribbean	2021	Group B *Streptococcus*	Fluoroquinolones	63	138 (56-220)
Latin America and Caribbean	2021	*Haemophilus influenzae*	3GC	64	108 (38-178)
Latin America and Caribbean	2021	*Acinetobacter baumannii*	4GC	65	106 (87-126)
Latin America and Caribbean	2021	*Serratia* spp.	4GC	66	100 (80-120)
Latin America and Caribbean	2021	Group A *Streptococcus*	Macrolides	67	98 (59-136)
Latin America and Caribbean	2021	Group B *Streptococcus*	Penicillin	68	76 (23-130)
Latin America and Caribbean	2021	*Mycobacterium tuberculosis*	XDR	69	76 (0-174)
Latin America and Caribbean	2021	*Acinetobacter baumannii*	Beta-Lactams/Beta-Lactamase Inhib.	70	68 (39-98)
Latin America and Caribbean	2021	*Citrobacter* spp.	Aminoglycosides	71	63 (49-77)
Latin America and Caribbean	2021	*Proteus* spp.	TMP-SMX	72	59 (0-148)
Latin America and Caribbean	2021	*Morganella* spp.	3GC	73	56 (40-72)
Latin America and Caribbean	2021	*Citrobacter* spp.	4GC	74	50 (41-58)
Latin America and Caribbean	2021	*Citrobacter* spp.	3GC	75	38 (13-62)
Latin America and Caribbean	2021	*Shigella* spp.	Fluoroquinolones	76	31 (3-58)
Latin America and Caribbean	2021	*Serratia* spp.	3GC	77	27 (8-46)
Latin America and Caribbean	2021	*Non-typhoidal Salmonella*	Fluoroquinolones	78	21 (0-54)
Latin America and Caribbean	2021	*Salmonella* Typhi	Fluoroquinolones	79	3 (1-5)
Latin America and Caribbean	2021	*Salmonella* Typhi	MDR	80	3 (0-6)
Latin America and Caribbean	2021	*Salmonella* Paratyphi	Fluoroquinolones	81	1 (0-2)
Latin America and Caribbean	2021	*Salmonella* Paratyphi	MDR	82	0 (0-0)
North Africa and Middle East	2021	*Staphylococcus aureus*	Methicillin	1	8,160 (6,160-10,100)
North Africa and Middle East	2021	*Streptococcus pneumoniae*	Carbapenems	2	6,250 (4,260-8,240)
North Africa and Middle East	2021	*Acinetobacter baumannii*	Carbapenems	3	4,900 (3,910-5,890)
North Africa and Middle East	2021	*Pseudomonas aeruginosa*	Carbapenems	4	3,330 (2,330-4,330)
North Africa and Middle East	2021	*Acinetobacter baumannii*	Fluoroquinolones	5	2,700 (2,180-3,220)
North Africa and Middle East	2021	*Klebsiella pneumoniae*	Carbapenems	6	2,240 (1,680-2,790)
North Africa and Middle East	2021	*Klebsiella pneumoniae*	Fluoroquinolones	7	1,810 (1,250-2,360)
North Africa and Middle East	2021	*Escherichia coli*	3GC	8	1,710 (1,160-2,260)
North Africa and Middle East	2021	*Klebsiella pneumoniae*	Aminoglycosides	9	1,500 (1,100-1,900)
North Africa and Middle East	2021	*Escherichia coli*	Fluoroquinolones	10	1,440 (935-1,950)
North Africa and Middle East	2021	*Acinetobacter baumannii*	Aminoglycosides	11	1,400 (1,050-1,750)
North Africa and Middle East	2021	*Escherichia coli*	Carbapenems	12	1,330 (913-1,740)
North Africa and Middle East	2021	*Pseudomonas aeruginosa*	Fluoroquinolones	13	1,200 (807-1,580)
North Africa and Middle East	2021	*Klebsiella pneumoniae*	3GC	14	1,090 (665-1,510)
North Africa and Middle East	2021	*Escherichia coli*	TMP-SMX	15	1,060 (615-1,510)
North Africa and Middle East	2021	*Staphylococcus aureus*	Fluoroquinolones	16	977 (393-1,560)
North Africa and Middle East	2021	*Klebsiella pneumoniae*	TMP-SMX	17	935 (467-1,400)
North Africa and Middle East	2021	*Pseudomonas aeruginosa*	Aminoglycosides	18	843 (560-1,130)
North Africa and Middle East	2021	*Staphylococcus aureus*	Macrolides	19	818 (533-1,100)
North Africa and Middle East	2021	*Streptococcus pneumoniae*	TMP-SMX	20	791 (0-1,700)
North Africa and Middle East	2021	*Streptococcus pneumoniae*	Macrolides	21	759 (456-1,060)
North Africa and Middle East	2021	*Streptococcus pneumoniae*	Fluoroquinolones	22	752 (324-1,180)
North Africa and Middle East	2021	*Streptococcus pneumoniae*	Beta-Lactams/Beta-Lactamase Inhib.	23	749 (424-1,070)
North Africa and Middle East	2021	*Enterobacter faecalis*	Fluoroquinolones	24	704 (455-953)
North Africa and Middle East	2021	*Staphylococcus aureus*	TMP-SMX	25	617 (375-860)
North Africa and Middle East	2021	*Enterococcus faecium*	Vancomycin	26	612 (479-745)
North Africa and Middle East	2021	*Escherichia coli*	Beta-Lactams/Beta-Lactamase Inhib.	27	581 (165-998)
North Africa and Middle East	2021	*Pseudomonas aeruginosa*	Anti-pseudomonal	28	581 (454-707)

North Africa and Middle East	2021	*Staphylococcus aureus*	Vancomycin	29	570 (344-796)
North Africa and Middle East	2021	*Streptococcus pneumoniae*	Penicillin	30	561 (457-665)
North Africa and Middle East	2021	*Klebsiella pneumoniae*	Beta-Lactams/Beta-Lactamase Inhib.	31	514 (201-828)
North Africa and Middle East	2021	*Streptococcus pneumoniae*	3GC	32	501 (321-680)
North Africa and Middle East	2021	*Enterococcus faecium*	Fluoroquinolones	33	461 (235-686)
North Africa and Middle East	2021	*Enterobacter faecalis*	Vancomycin	34	443 (251-636)
North Africa and Middle East	2021	*Mycobacterium tuberculosis*	MDR excluding XDR	35	439 (0-1,240)
North Africa and Middle East	2021	*Escherichia coli*	Aminopenicillin	36	437 (328-547)
North Africa and Middle East	2021	*Escherichia coli*	Aminoglycosides	37	424 (256-591)
North Africa and Middle East	2021	*Enterobacter* spp.	Carbapenems	38	369 (277-462)
North Africa and Middle East	2021	*Enterobacter* spp.	Anti-pseudomonal	39	357 (287-426)
North Africa and Middle East	2021	*Serratia* spp.	Anti-pseudomonal	40	316 (249-383)
North Africa and Middle East	2021	*Serratia* spp.	Carbapenems	41	283 (220-345)
North Africa and Middle East	2021	*Pseudomonas aeruginosa*	4GC	42	266 (190-343)
North Africa and Middle East	2021	*Enterobacter* spp.	Fluoroquinolones	43	244 (135-354)
North Africa and Middle East	2021	*Proteus* spp.	Aminopenicillin	44	223 (169-277)
North Africa and Middle East	2021	*Acinetobacter baumannii*	4GC	45	219 (155-284)
North Africa and Middle East	2021	*Morganella* spp.	Fluoroquinolones	46	213 (109-318)
North Africa and Middle East	2021	*Proteus* spp.	3GC	47	208 (127-289)
North Africa and Middle East	2021	*Enterobacter* spp.	4GC	48	207 (168-247)
North Africa and Middle East	2021	*Acinetobacter baumannii*	Anti-pseudomonal	49	201 (136-266)
North Africa and Middle East	2021	*Acinetobacter baumannii*	3GC	50	195 (119-270)
North Africa and Middle East	2021	Group B *Streptococcus*	Fluoroquinolones	51	188 (79-296)
North Africa and Middle East	2021	*Pseudomonas aeruginosa*	3GC	52	170 (66-273)
North Africa and Middle East	2021	*Citrobacter* spp.	Fluoroquinolones	53	167 (81-252)
North Africa and Middle East	2021	*Citrobacter* spp.	Anti-pseudomonal	54	151 (123-179)
North Africa and Middle East	2021	*Haemophilus influenzae*	Aminopenicillin	55	144 (102-186)
North Africa and Middle East	2021	*Haemophilus influenzae*	3GC	56	141 (0-323)
North Africa and Middle East	2021	*Serratia* spp.	Fluoroquinolones	57	138 (38-238)
North Africa and Middle East	2021	*Enterobacter* spp.	Aminoglycosides	58	138 (81-195)
North Africa and Middle East	2021	*Serratia* spp.	Aminoglycosides	59	134 (80-188)
North Africa and Middle East	2021	*Proteus* spp.	Aminoglycosides	60	131 (79-183)
North Africa and Middle East	2021	*Citrobacter* spp.	Carbapenems	61	114 (60-167)
North Africa and Middle East	2021	*Proteus* spp.	Fluoroquinolones	62	112 (17-207)
North Africa and Middle East	2021	Group B *Streptococcus*	Macrolides	63	107 (65-148)
North Africa and Middle East	2021	*Enterobacter* spp.	TMP-SMX	64	102 (7-196)
North Africa and Middle East	2021	Group A *Streptococcus*	Macrolides	65	82 (49-116)
North Africa and Middle East	2021	*Morganella* spp.	4GC	66	82 (55-109)
North Africa and Middle East	2021	*Serratia* spp.	4GC	67	76 (61-91)
North Africa and Middle East	2021	Group B *Streptococcus*	Penicillin	68	73 (7-139)
North Africa and Middle East	2021	*Citrobacter* spp.	4GC	69	64 (50-78)
North Africa and Middle East	2021	*Morganella* spp.	3GC	70	62 (44-80)
North Africa and Middle East	2021	*Citrobacter* spp.	Aminoglycosides	71	59 (45-73)
North Africa and Middle East	2021	*Proteus* spp.	TMP-SMX	72	49 (0-128)
North Africa and Middle East	2021	*Shigella* spp.	Fluoroquinolones	73	45 (0-91)
North Africa and Middle East	2021	*Mycobacterium tuberculosis*	XDR	74	44 (0-102)
North Africa and Middle East	2021	*Non-typhoidal Salmonella*	Fluoroquinolones	75	41 (3-79)
North Africa and Middle East	2021	*Citrobacter* spp.	3GC	76	32 (8-57)
North Africa and Middle East	2021	*Salmonella* Typhi	MDR	77	31 (0-69)
North Africa and Middle East	2021	*Acinetobacter baumannii*	Beta-Lactams/Beta-Lactamase Inhib.	78	25 (7-42)
North Africa and Middle East	2021	*Serratia* spp.	3GC	79	25 (0-56)
North Africa and Middle East	2021	*Salmonella* Typhi	Fluoroquinolones	80	20 (2-39)
North Africa and Middle East	2021	*Salmonella* Paratyphi	Fluoroquinolones	81	1 (0-2)
North Africa and Middle East	2021	*Salmonella* Paratyphi	MDR	82	0 (0-0)
South Asia	2021	*Acinetobacter baumannii*	Carbapenems	1	28,300 (22,600-34,100)
South Asia	2021	*Staphylococcus aureus*	Methicillin	2	25,600 (20,900-30,200)
South Asia	2021	*Streptococcus pneumoniae*	Carbapenems	3	22,800 (13,600-31,900)
South Asia	2021	*Mycobacterium tuberculosis*	MDR excluding XDR	4	22,000 (0-52,600)
South Asia	2021	*Klebsiella pneumoniae*	Carbapenems	5	21,400 (16,700-26,100)
South Asia	2021	*Acinetobacter baumannii*	Fluoroquinolones	6	15,600 (12,800-18,500)

South Asia	2021	*Escherichia coli*	Carbapenems	7	15,300 (12,200-18,500)
South Asia	2021	*Klebsiella pneumoniae*	Fluoroquinolones	8	11,600 (8,470-14,800)
South Asia	2021	*Pseudomonas aeruginosa*	Carbapenems	9	11,200 (7,320-15,100)
South Asia	2021	*Escherichia coli*	Fluoroquinolones	10	10,500 (7,480-13,400)
South Asia	2021	*Escherichia coli*	3GC	11	9,650 (6,600-12,700)
South Asia	2021	*Acinetobacter baumannii*	Aminoglycosides	12	9,370 (7,300-11,400)
South Asia	2021	*Klebsiella pneumoniae*	Aminoglycosides	13	8,590 (6,580-10,600)
South Asia	2021	*Staphylococcus aureus*	Fluoroquinolones	14	7,740 (3,080-12,400)
South Asia	2021	*Escherichia coli*	TMP-SMX	15	6,000 (3,910-8,080)
South Asia	2021	*Streptococcus pneumoniae*	Penicillin	16	5,780 (4,340-7,230)
South Asia	2021	*Streptococcus pneumoniae*	Macrolides	17	5,580 (3,440-7,720)
South Asia	2021	*Pseudomonas aeruginosa*	Fluoroquinolones	18	5,390 (3,660-7,110)
South Asia	2021	*Klebsiella pneumoniae*	3GC	19	4,690 (3,000-6,380)
South Asia	2021	*Pseudomonas aeruginosa*	Aminoglycosides	20	4,520 (3,260-5,770)
South Asia	2021	*Enterobacter faecalis*	Fluoroquinolones	21	4,030 (2,790-5,270)
South Asia	2021	*Klebsiella pneumoniae*	TMP-SMX	22	3,950 (2,040-5,860)
South Asia	2021	*Escherichia coli*	Aminoglycosides	23	3,820 (2,530-5,100)
South Asia	2021	*Staphylococcus aureus*	Macrolides	24	3,570 (2,340-4,800)
South Asia	2021	*Streptococcus pneumoniae*	TMP-SMX	25	3,520 (0-7,630)
South Asia	2021	*Staphylococcus aureus*	TMP-SMX	26	3,510 (2,160-4,850)
South Asia	2021	*Pseudomonas aeruginosa*	Anti-pseudomonal	27	3,200 (2,610-3,790)
South Asia	2021	*Enterobacter* spp.	Carbapenems	28	3,030 (2,330-3,720)
South Asia	2021	*Enterococcus faecium*	Vancomycin	29	2,860 (2,270-3,450)
South Asia	2021	*Streptococcus pneumoniae*	Fluoroquinolones	30	2,730 (1,270-4,200)
South Asia	2021	*Salmonella* Typhi	Fluoroquinolones	31	2,520 (385-4,660)
South Asia	2021	*Pseudomonas aeruginosa*	4GC	32	2,240 (1,660-2,820)
South Asia	2021	*Salmonella* Typhi	MDR	33	1,930 (168-3,690)
South Asia	2021	*Escherichia coli*	Beta-Lactams/Beta-Lactamase Inhib.	34	1,900 (532-3,270)
South Asia	2021	*Enterococcus faecium*	Fluoroquinolones	35	1,880 (939-2,830)
South Asia	2021	*Citrobacter* spp.	Carbapenems	36	1,870 (1,100-2,630)
South Asia	2021	*Acinetobacter baumannii*	3GC	37	1,620 (1,350-1,880)
South Asia	2021	*Salmonella* Paratyphi	Fluoroquinolones	38	1,610 (265-2,950)
South Asia	2021	*Shigella* spp.	Fluoroquinolones	39	1,580 (333-2,820)
South Asia	2021	*Morganella* spp.	4GC	40	1,510 (1,140-1,870)
South Asia	2021	*Serratia* spp.	Carbapenems	41	1,480 (1,110-1,840)
South Asia	2021	Group B *Streptococcus*	Fluoroquinolones	42	1,440 (697-2,180)
South Asia	2021	*Serratia* spp.	Aminoglycosides	43	1,380 (933-1,820)
South Asia	2021	*Acinetobacter baumannii*	Anti-pseudomonal	44	1,370 (1,050-1,700)
South Asia	2021	*Enterobacter* spp.	Fluoroquinolones	45	1,350 (774-1,920)
South Asia	2021	*Enterobacter* spp.	Anti-pseudomonal	46	1,340 (1,080-1,600)
South Asia	2021	*Mycobacterium tuberculosis*	XDR	47	1,300 (0-2,710)
South Asia	2021	*Klebsiella pneumoniae*	Beta-Lactams/Beta-Lactamase Inhib.	48	1,300 (451-2,160)
South Asia	2021	*Proteus* spp.	3GC	49	1,300 (897-1,700)
South Asia	2021	*Serratia* spp.	Anti-pseudomonal	50	1,230 (972-1,490)
South Asia	2021	*Enterobacter* spp.	Aminoglycosides	51	1,180 (837-1,520)
South Asia	2021	*Escherichia coli*	Aminopenicillin	52	1,080 (756-1,400)
South Asia	2021	*Morganella* spp.	Fluoroquinolones	53	1,030 (567-1,500)
South Asia	2021	*Enterobacter* spp.	4GC	54	952 (786-1,120)
South Asia	2021	*Haemophilus influenzae*	Aminopenicillin	55	912 (641-1,180)
South Asia	2021	*Citrobacter* spp.	Fluoroquinolones	56	892 (578-1,210)
South Asia	2021	*Proteus* spp.	Aminoglycosides	57	859 (623-1,100)
South Asia	2021	*Proteus* spp.	Fluoroquinolones	58	736 (332-1,140)
South Asia	2021	*Staphylococcus aureus*	Vancomycin	59	732 (426-1,040)
South Asia	2021	*Streptococcus pneumoniae*	3GC	60	710 (429-991)
South Asia	2021	Group B *Streptococcus*	Macrolides	61	638 (397-880)
South Asia	2021	*Enterobacter faecalis*	Vancomycin	62	615 (283-947)
South Asia	2021	*Serratia* spp.	Fluoroquinolones	63	572 (180-963)
South Asia	2021	*Proteus* spp.	Aminopenicillin	64	532 (401-663)
South Asia	2021	*Pseudomonas aeruginosa*	3GC	65	520 (201-840)
South Asia	2021	*Non-typhoidal Salmonella*	Fluoroquinolones	66	496 (0-1,080)

South Asia	2021	*Enterobacter* spp.	TMP-SMX	67	490 (60-920)
South Asia	2021	Group A *Streptococcus*	Macrolides	68	485 (297-673)
South Asia	2021	*Morganella* spp.	3GC	69	449 (320-578)
South Asia	2021	*Haemophilus influenzae*	3GC	70	389 (207-570)
South Asia	2021	*Citrobacter* spp.	3GC	71	371 (257-485)
South Asia	2021	*Citrobacter* spp.	Anti-pseudomonal	72	354 (270-437)
South Asia	2021	*Citrobacter* spp.	Aminoglycosides	73	294 (224-364)
South Asia	2021	*Serratia* spp.	4GC	74	292 (225-359)
South Asia	2021	*Acinetobacter baumannii*	4GC	75	248 (162-335)
South Asia	2021	Group B *Streptococcus*	Penicillin	76	172 (69-274)
South Asia	2021	*Acinetobacter baumannii*	Beta-Lactams/Beta-Lactamase Inhib.	77	153 (62-244)
South Asia	2021	*Serratia* spp.	3GC	78	152 (0-367)
South Asia	2021	*Proteus* spp.	TMP-SMX	79	150 (0-380)
South Asia	2021	*Citrobacter* spp.	4GC	80	113 (79-148)
South Asia	2021	*Streptococcus pneumoniae*	Beta-Lactams/Beta-Lactamase Inhib.	81	105 (7-203)
South Asia	2021	*Salmonella* Paratyphi	MDR	82	102 (0-214)
Southeast Asia, East Asia, and Oceania	2021	*Staphylococcus aureus*	Methicillin	1	38,200 (29,200-47,200)
Southeast Asia, East Asia, and Oceania	2021	*Acinetobacter baumannii*	Carbapenems	2	21,800 (17,100-26,500)
Southeast Asia, East Asia, and Oceania	2021	*Streptococcus pneumoniae*	Carbapenems	3	17,200 (13,100-21,200)
Southeast Asia, East Asia, and Oceania	2021	*Acinetobacter baumannii*	Fluoroquinolones	4	12,000 (9,750-14,300)
Southeast Asia, East Asia, and Oceania	2021	*Pseudomonas aeruginosa*	Carbapenems	5	11,000 (7,480-14,500)
Southeast Asia, East Asia, and Oceania	2021	*Klebsiella pneumoniae*	Carbapenems	6	8,300 (6,470-10,100)
Southeast Asia, East Asia, and Oceania	2021	*Streptococcus pneumoniae*	3GC	7	8,110 (5,330-10,900)
Southeast Asia, East Asia, and Oceania	2021	*Escherichia coli*	3GC	8	7,980 (5,360-10,600)
Southeast Asia, East Asia, and Oceania	2021	*Staphylococcus aureus*	Macrolides	9	7,620 (5,030-10,200)
Southeast Asia, East Asia, and Oceania	2021	*Streptococcus pneumoniae*	Macrolides	10	7,620 (4,530-10,700)
Southeast Asia, East Asia, and Oceania	2021	*Klebsiella pneumoniae*	Fluoroquinolones	11	7,470 (5,240-9,700)
Southeast Asia, East Asia, and Oceania	2021	*Escherichia coli*	Fluoroquinolones	12	7,450 (4,580-10,300)
Southeast Asia, East Asia, and Oceania	2021	*Acinetobacter baumannii*	Aminoglycosides	13	6,370 (4,860-7,880)
Southeast Asia, East Asia, and Oceania	2021	*Staphylococcus aureus*	Fluoroquinolones	14	6,280 (2,110-10,400)
Southeast Asia, East Asia, and Oceania	2021	*Pseudomonas aeruginosa*	Fluoroquinolones	15	5,580 (3,770-7,390)
Southeast Asia, East Asia, and Oceania	2021	*Klebsiella pneumoniae*	Aminoglycosides	16	4,700 (3,390-6,010)
Southeast Asia, East Asia, and Oceania	2021	*Escherichia coli*	TMP-SMX	17	4,680 (2,720-6,640)
Southeast Asia, East Asia, and Oceania	2021	*Escherichia coli*	Carbapenems	18	4,160 (3,280-5,040)
Southeast Asia, East Asia, and Oceania	2021	*Streptococcus pneumoniae*	TMP-SMX	19	4,100 (0-8,850)
Southeast Asia, East Asia, and Oceania	2021	*Escherichia coli*	Aminopenicillin	20	4,030 (2,770-5,290)
Southeast Asia, East Asia, and Oceania	2021	*Pseudomonas aeruginosa*	Anti-pseudomonal	21	4,000 (3,120-4,880)
Southeast Asia, East Asia, and Oceania	2021	*Klebsiella pneumoniae*	3GC	22	3,880 (2,490-5,280)
Southeast Asia, East Asia, and Oceania	2021	*Mycobacterium tuberculosis*	MDR excluding XDR	23	3,290 (0-9,530)
Southeast Asia, East Asia, and Oceania	2021	*Enterococcus faecium*	Fluoroquinolones	24	3,210 (1,640-4,790)
Southeast Asia, East Asia, and Oceania	2021	*Enterobacter faecalis*	Fluoroquinolones	25	3,190 (2,060-4,330)
Southeast Asia, East Asia, and Oceania	2021	*Enterobacter* spp.	Carbapenems	26	3,170 (2,510-3,820)
Southeast Asia, East Asia, and Oceania	2021	*Streptococcus pneumoniae*	Fluoroquinolones	27	3,080 (1,230-4,930)
Southeast Asia, East Asia, and Oceania	2021	*Klebsiella pneumoniae*	TMP-SMX	28	3,050 (1,520-4,570)
Southeast Asia, East Asia, and Oceania	2021	*Escherichia coli*	Beta-Lactams/Beta-Lactamase Inhib.	29	2,830 (657-4,990)
Southeast Asia, East Asia, and Oceania	2021	*Staphylococcus aureus*	TMP-SMX	30	2,680 (1,610-3,750)
Southeast Asia, East Asia, and Oceania	2021	*Acinetobacter baumannii*	Anti-pseudomonal	31	2,670 (1,950-3,400)
Southeast Asia, East Asia, and Oceania	2021	*Escherichia coli*	Aminoglycosides	32	2,200 (1,440-2,960)
Southeast Asia, East Asia, and Oceania	2021	*Pseudomonas aeruginosa*	Aminoglycosides	33	2,100 (1,350-2,860)
Southeast Asia, East Asia, and Oceania	2021	*Acinetobacter baumannii*	3GC	34	2,080 (1,700-2,470)
Southeast Asia, East Asia, and Oceania	2021	*Enterobacter* spp.	Anti-pseudomonal	35	1,960 (1,570-2,360)
Southeast Asia, East Asia, and Oceania	2021	*Staphylococcus aureus*	Vancomycin	36	1,820 (1,070-2,560)
Southeast Asia, East Asia, and Oceania	2021	*Haemophilus influenzae*	Aminopenicillin	37	1,530 (1,110-1,960)
Southeast Asia, East Asia, and Oceania	2021	*Klebsiella pneumoniae*	Beta-Lactams/Beta-Lactamase Inhib.	38	1,500 (583-2,420)
Southeast Asia, East Asia, and Oceania	2021	*Streptococcus pneumoniae*	Penicillin	39	1,500 (1,190-1,810)
Southeast Asia, East Asia, and Oceania	2021	*Streptococcus pneumoniae*	Beta-Lactams/Beta-Lactamase Inhib.	40	1,470 (392-2,540)
Southeast Asia, East Asia, and Oceania	2021	*Acinetobacter baumannii*	Beta-Lactams/Beta-Lactamase Inhib.	41	1,420 (934-1,900)
Southeast Asia, East Asia, and Oceania	2021	*Enterobacter* spp.	Fluoroquinolones	42	1,410 (785-2,040)
Southeast Asia, East Asia, and Oceania	2021	Group A *Streptococcus*	Macrolides	43	1,240 (717-1,770)
Southeast Asia, East Asia, and Oceania	2021	*Proteus* spp.	3GC	44	1,150 (744-1,550)

Southeast Asia, East Asia, and Oceania	2021	*Enterococcus faecium*	Vancomycin	45	1,150 (875-1,420)
Southeast Asia, East Asia, and Oceania	2021	*Serratia* spp.	Carbapenems	46	1,080 (840-1,330)
Southeast Asia, East Asia, and Oceania	2021	*Proteus* spp.	Aminopenicillin	47	1,030 (777-1,290)
Southeast Asia, East Asia, and Oceania	2021	*Citrobacter* spp.	Anti-pseudomonal	48	897 (719-1,070)
Southeast Asia, East Asia, and Oceania	2021	*Acinetobacter baumannii*	4GC	49	891 (703-1,080)
Southeast Asia, East Asia, and Oceania	2021	*Serratia* spp.	Anti-pseudomonal	50	890 (700-1,080)
Southeast Asia, East Asia, and Oceania	2021	*Citrobacter* spp.	Fluoroquinolones	51	799 (519-1,080)
Southeast Asia, East Asia, and Oceania	2021	*Morganella* spp.	Fluoroquinolones	52	788 (375-1,200)
Southeast Asia, East Asia, and Oceania	2021	*Enterobacter* spp.	Aminoglycosides	53	751 (502-999)
Southeast Asia, East Asia, and Oceania	2021	*Citrobacter* spp.	Carbapenems	54	709 (441-977)
Southeast Asia, East Asia, and Oceania	2021	*Enterobacter* spp.	4GC	55	707 (579-835)
Southeast Asia, East Asia, and Oceania	2021	*Mycobacterium tuberculosis*	XDR	56	696 (0-1,690)
Southeast Asia, East Asia, and Oceania	2021	*Proteus* spp.	Aminoglycosides	57	669 (459-879)
Southeast Asia, East Asia, and Oceania	2021	*Proteus* spp.	Fluoroquinolones	58	655 (247-1,060)
Southeast Asia, East Asia, and Oceania	2021	Group B *Streptococcus*	Fluoroquinolones	59	644 (295-994)
Southeast Asia, East Asia, and Oceania	2021	*Serratia* spp.	Aminoglycosides	60	632 (421-843)
Southeast Asia, East Asia, and Oceania	2021	*Enterobacter faecalis*	Vancomycin	61	590 (160-1,020)
Southeast Asia, East Asia, and Oceania	2021	Group B *Streptococcus*	Macrolides	62	582 (362-802)
Southeast Asia, East Asia, and Oceania	2021	*Serratia* spp.	Fluoroquinolones	63	563 (172-954)
Southeast Asia, East Asia, and Oceania	2021	*Salmonella* Typhi	Fluoroquinolones	64	480 (42-917)
Southeast Asia, East Asia, and Oceania	2021	*Pseudomonas aeruginosa*	3GC	65	471 (65-877)
Southeast Asia, East Asia, and Oceania	2021	*Pseudomonas aeruginosa*	4GC	66	467 (304-630)
Southeast Asia, East Asia, and Oceania	2021	*Enterobacter* spp.	TMP-SMX	67	410 (62-758)
Southeast Asia, East Asia, and Oceania	2021	*Morganella* spp.	4GC	68	394 (268-520)
Southeast Asia, East Asia, and Oceania	2021	*Haemophilus influenzae*	3GC	69	385 (157-614)
Southeast Asia, East Asia, and Oceania	2021	*Serratia* spp.	4GC	70	321 (268-373)
Southeast Asia, East Asia, and Oceania	2021	*Citrobacter* spp.	4GC	71	282 (216-347)
Southeast Asia, East Asia, and Oceania	2021	*Morganella* spp.	3GC	72	249 (179-318)
Southeast Asia, East Asia, and Oceania	2021	*Proteus* spp.	TMP-SMX	73	183 (0-470)
Southeast Asia, East Asia, and Oceania	2021	*Citrobacter* spp.	Aminoglycosides	74	160 (122-199)
Southeast Asia, East Asia, and Oceania	2021	*Non-typhoidal Salmonella*	Fluoroquinolones	75	134 (0-285)
Southeast Asia, East Asia, and Oceania	2021	Group B *Streptococcus*	Penicillin	76	124 (38-210)
Southeast Asia, East Asia, and Oceania	2021	*Salmonella* Typhi	MDR	77	107 (9-205)
Southeast Asia, East Asia, and Oceania	2021	*Serratia* spp.	3GC	78	99 (11-188)
Southeast Asia, East Asia, and Oceania	2021	*Shigella* spp.	Fluoroquinolones	79	91 (0-183)
Southeast Asia, East Asia, and Oceania	2021	*Citrobacter* spp.	3GC	80	90 (31-148)
Southeast Asia, East Asia, and Oceania	2021	*Salmonella* Paratyphi	Fluoroquinolones	81	36 (3-70)
Southeast Asia, East Asia, and Oceania	2021	*Salmonella* Paratyphi	MDR	82	4 (0-17)
Sub-Saharan Africa	2021	*Streptococcus pneumoniae*	Carbapenems	1	11,300 (6,650-15,900)
Sub-Saharan Africa	2021	*Acinetobacter baumannii*	Carbapenems	2	9,690 (6,660-12,700)
Sub-Saharan Africa	2021	*Staphylococcus aureus*	Methicillin	3	9,610 (6,520-12,700)
Sub-Saharan Africa	2021	*Mycobacterium tuberculosis*	MDR excluding XDR	4	8,270 (0-21,400)
Sub-Saharan Africa	2021	*Klebsiella pneumoniae*	3GC	5	8,200 (4,840-11,600)
Sub-Saharan Africa	2021	*Klebsiella pneumoniae*	Fluoroquinolones	6	7,550 (4,800-10,300)
Sub-Saharan Africa	2021	*Acinetobacter baumannii*	Fluoroquinolones	7	6,810 (5,320-8,300)
Sub-Saharan Africa	2021	*Klebsiella pneumoniae*	TMP-SMX	8	6,740 (3,360-10,100)
Sub-Saharan Africa	2021	*Klebsiella pneumoniae*	Aminoglycosides	9	6,470 (4,480-8,460)
Sub-Saharan Africa	2021	*Streptococcus pneumoniae*	3GC	10	6,320 (4,000-8,630)
Sub-Saharan Africa	2021	*Escherichia coli*	3GC	11	6,050 (3,160-8,940)
Sub-Saharan Africa	2021	*Escherichia coli*	TMP-SMX	12	6,000 (4,020-7,980)
Sub-Saharan Africa	2021	*Streptococcus pneumoniae*	Fluoroquinolones	13	5,470 (2,000-8,950)
Sub-Saharan Africa	2021	*Klebsiella pneumoniae*	Carbapenems	14	5,180 (3,730-6,630)
Sub-Saharan Africa	2021	*Escherichia coli*	Fluoroquinolones	15	5,160 (3,190-7,140)
Sub-Saharan Africa	2021	*Pseudomonas aeruginosa*	Carbapenems	16	5,010 (2,810-7,220)
Sub-Saharan Africa	2021	*Streptococcus pneumoniae*	TMP-SMX	17	4,550 (0-9,730)
Sub-Saharan Africa	2021	*Pseudomonas aeruginosa*	Anti-pseudomonal	18	4,510 (3,240-5,780)
Sub-Saharan Africa	2021	*Pseudomonas aeruginosa*	Fluoroquinolones	19	4,420 (2,840-6,010)
Sub-Saharan Africa	2021	*Klebsiella pneumoniae*	Beta-Lactams/Beta-Lactamase Inhib.	20	4,350 (1,850-6,850)
Sub-Saharan Africa	2021	*Staphylococcus aureus*	TMP-SMX	21	4,100 (2,380-5,820)
Sub-Saharan Africa	2021	*Escherichia coli*	Carbapenems	22	3,930 (2,050-5,810)

Sub-Saharan Africa	2021	*Streptococcus pneumoniae*	Penicillin	23	3,780 (2,310-5,240)
Sub-Saharan Africa	2021	*Pseudomonas aeruginosa*	Aminoglycosides	24	3,660 (2,340-4,980)
Sub-Saharan Africa	2021	*Escherichia coli*	Beta-Lactams/Beta-Lactamase Inhib.	25	3,520 (854-6,190)
Sub-Saharan Africa	2021	*Acinetobacter baumannii*	Aminoglycosides	26	3,330 (2,300-4,350)
Sub-Saharan Africa	2021	*Acinetobacter baumannii*	Anti-pseudomonal	27	2,950 (2,390-3,520)
Sub-Saharan Africa	2021	*Escherichia coli*	Aminoglycosides	28	2,900 (1,150-4,650)
Sub-Saharan Africa	2021	*Streptococcus pneumoniae*	Macrolides	29	2,890 (1,670-4,110)
Sub-Saharan Africa	2021	*Acinetobacter baumannii*	3GC	30	2,470 (1,990-2,950)
Sub-Saharan Africa	2021	*Streptococcus pneumoniae*	Beta-Lactams/Beta-Lactamase Inhib.	31	2,210 (1,300-3,120)
Sub-Saharan Africa	2021	*Shigella* spp.	Fluoroquinolones	32	2,200 (0-4,480)
Sub-Saharan Africa	2021	*Staphylococcus aureus*	Fluoroquinolones	33	2,130 (619-3,640)
Sub-Saharan Africa	2021	*Serratia* spp.	Anti-pseudomonal	34	2,040 (1,500-2,580)
Sub-Saharan Africa	2021	*Haemophilus influenzae*	3GC	35	1,940 (0-3,940)
Sub-Saharan Africa	2021	*Escherichia coli*	Aminopenicillin	36	1,740 (1,060-2,420)
Sub-Saharan Africa	2021	*Acinetobacter baumannii*	4GC	37	1,560 (1,200-1,920)
Sub-Saharan Africa	2021	*Staphylococcus aureus*	Macrolides	38	1,460 (923-2,000)
Sub-Saharan Africa	2021	*Enterobacter* spp.	Carbapenems	39	1,460 (968-1,950)
Sub-Saharan Africa	2021	Group B *Streptococcus*	Fluoroquinolones	40	1,410 (559-2,270)
Sub-Saharan Africa	2021	*Serratia* spp.	Carbapenems	41	1,310 (947-1,670)
Sub-Saharan Africa	2021	*Enterobacter faecalis*	Fluoroquinolones	42	1,300 (819-1,780)
Sub-Saharan Africa	2021	*Staphylococcus aureus*	Vancomycin	43	1,270 (538-1,990)
Sub-Saharan Africa	2021	*Non-typhoidal Salmonella*	Fluoroquinolones	44	1,250 (177-2,320)
Sub-Saharan Africa	2021	*Serratia* spp.	4GC	45	1,250 (954-1,540)
Sub-Saharan Africa	2021	*Enterobacter* spp.	4GC	46	1,200 (926-1,470)
Sub-Saharan Africa	2021	*Acinetobacter baumannii*	Beta-Lactams/Beta-Lactamase Inhib.	47	1,060 (584-1,530)
Sub-Saharan Africa	2021	*Haemophilus influenzae*	Aminopenicillin	48	1,050 (744-1,350)
Sub-Saharan Africa	2021	*Serratia* spp.	Aminoglycosides	49	1,000 (628-1,370)
Sub-Saharan Africa	2021	*Citrobacter* spp.	Anti-pseudomonal	50	972 (710-1,230)
Sub-Saharan Africa	2021	*Enterobacter* spp.	Fluoroquinolones	51	957 (500-1,410)
Sub-Saharan Africa	2021	*Enterobacter* spp.	Anti-pseudomonal	52	857 (650-1,060)
Sub-Saharan Africa	2021	*Enterobacter* spp.	Aminoglycosides	53	853 (547-1,160)
Sub-Saharan Africa	2021	Group B *Streptococcus*	Penicillin	54	829 (255-1,400)
Sub-Saharan Africa	2021	Group B *Streptococcus*	Macrolides	55	824 (494-1,150)
Sub-Saharan Africa	2021	*Enterococcus faecium*	Fluoroquinolones	56	715 (286-1,140)
Sub-Saharan Africa	2021	*Pseudomonas aeruginosa*	4GC	57	693 (445-940)
Sub-Saharan Africa	2021	*Citrobacter* spp.	Fluoroquinolones	58	675 (325-1,020)
Sub-Saharan Africa	2021	*Proteus* spp.	3GC	59	631 (372-891)
Sub-Saharan Africa	2021	*Salmonella* Typhi	MDR	60	612 (0-1,290)
Sub-Saharan Africa	2021	*Pseudomonas aeruginosa*	3GC	61	608 (166-1,050)
Sub-Saharan Africa	2021	*Morganella* spp.	Fluoroquinolones	62	592 (260-925)
Sub-Saharan Africa	2021	*Serratia* spp.	Fluoroquinolones	63	518 (116-919)
Sub-Saharan Africa	2021	*Enterococcus faecium*	Vancomycin	64	516 (380-651)
Sub-Saharan Africa	2021	*Proteus* spp.	Aminopenicillin	65	483 (353-613)
Sub-Saharan Africa	2021	*Citrobacter* spp.	Carbapenems	66	437 (207-667)
Sub-Saharan Africa	2021	*Enterobacter* spp.	TMP-SMX	67	404 (50-758)
Sub-Saharan Africa	2021	Group A *Streptococcus*	Macrolides	68	391 (232-549)
Sub-Saharan Africa	2021	*Proteus* spp.	Aminoglycosides	69	341 (193-489)
Sub-Saharan Africa	2021	*Citrobacter* spp.	Aminoglycosides	70	334 (240-429)
Sub-Saharan Africa	2021	*Citrobacter* spp.	4GC	71	311 (219-403)
Sub-Saharan Africa	2021	*Morganella* spp.	4GC	72	308 (194-423)
Sub-Saharan Africa	2021	*Enterobacter faecalis*	Vancomycin	73	270 (36-504)
Sub-Saharan Africa	2021	*Proteus* spp.	Fluoroquinolones	74	243 (26-460)
Sub-Saharan Africa	2021	*Salmonella* Typhi	Fluoroquinolones	75	233 (7-459)
Sub-Saharan Africa	2021	*Morganella* spp.	3GC	76	176 (122-231)
Sub-Saharan Africa	2021	*Mycobacterium tuberculosis*	XDR	77	155 (0-315)
Sub-Saharan Africa	2021	*Serratia* spp.	3GC	78	133 (0-354)
Sub-Saharan Africa	2021	*Proteus* spp.	TMP-SMX	79	110 (0-296)
Sub-Saharan Africa	2021	*Citrobacter* spp.	3GC	80	68 (10-126)
Sub-Saharan Africa	2021	*Salmonella* Paratyphi	Fluoroquinolones	81	20 (0-42)
Sub-Saharan Africa	2021	*Salmonella* Paratyphi	MDR	82	4 (0-33)

Table S15: Deaths (in counts and all-age rates) associated with and attributable to bacterial antimicrobial resistance, for those under 5 years of age and over 5 years of age, globally, by GBD super-region, and region, for 1990, 2019, 2021

			Deaths Associated						Attributed					
			Counts (Thousands)			Rate Per 100K			Counts (Thousands)			Rate Per 100K		
**Super Region**	**Region**	**Age**	**1990**	**2019**	**2021**	**1990**	**2019**	**2021**	**1990**	**2019**	**2021**	**1990**	**2019**	**2021**
**Central Europe, Eastern Europe, and Central Asia**	*Overall*	**5 plus**	235 (198-271)	267 (240-295)	255 (226-284)	61.0 (51.5-70.5)	68.3 (61.2-75.3)	65.0 (57.6-72.3)	52.0 (41.2-62.9)	65.4 (56.6-74.3)	61.6 (53.2-69.9)	13.5 (10.7-16.3)	16.7 (14.4-19.0)	15.7 (13.6-17.8)
		**Under 5**	50.3 (40.9-59.7)	13.5 (10.6-16.3)	10.1 (7.86-12.4)	140 (114-166)	49.8 (39.2-60.4)	39.4 (30.6-48.3)	11.0 (8.12-13.9)	3.19 (2.38-4.01)	2.39 (1.76-3.03)	30.7 (22.6-38.8)	11.8 (8.8-14.8)	9.32 (6.86-11.8)
	**C Asia**	**5 plus**	22.5 (18.5-26.6)	32.3 (26.9-37.8)	31.3 (25.5-37.0)	37.7 (30.9-44.5)	38.8 (32.2-45.4)	36.4 (29.7-43.1)	5.24 (3.92-6.57)	8.04 (6.26-9.83)	7.74 (5.98-9.5)	8.77 (6.55-11.0)	9.65 (7.51-11.8)	9.02 (6.96-11.1)
		**Under 5**	32.9 (26.3-39.4)	10.9 (8.34-13.5)	8.23 (6.16-10.3)	345 (276-414)	111 (84.3-137)	82.3 (61.7-103)	7.4 (5.35-9.46)	2.59 (1.86-3.33)	1.94 (1.37-2.51)	77.7 (56.1-99.3)	26.2 (18.8-33.6)	19.4 (13.7-25.1)
	**C Europe**	**5 plus**	81.9 (69.2-94.6)	83.9 (76.0-91.8)	79.8 (71.0-88.6)	70.6 (59.7-81.6)	75.8 (68.7-83.0)	72.7 (64.7-80.8)	18.7 (14.9-22.6)	19.6 (17.4-21.8)	18.6 (16.4-20.7)	16.2 (12.9-19.4)	17.7 (15.8-19.7)	16.9 (15.0-18.9)
		**Under 5**	6.91 (5.64-8.18)	0.682 (0.572-0.792)	0.477 (0.376-0.578)	75.6 (61.7-89.6)	11.9 (9.96-13.8)	8.54 (6.74-10.3)	1.55 (1.18-1.93)	0.154 (0.126-0.182)	0.108 (0.0837-0.131)	17.0 (12.9-21.2)	2.68 (2.19-3.17)	1.93 (1.5-2.35)
	**E Europe**	**5 plus**	131 (111-150)	151 (135-166)	144 (126-161)	62.4 (52.9-71.9)	76.4 (68.5-84.2)	73.0 (64.1-82.0)	28.1 (22.3-33.9)	37.7 (32.2-43.2)	35.3 (29.8-40.7)	13.4 (10.6-16.2)	19.1 (16.3-21.9)	17.9 (15.2-20.7)
		**Under 5**	10.4 (8.53-12.2)	1.79 (1.57-2.02)	1.41 (1.21-1.6)	60.3 (49.5-71.0)	15.8 (13.8-17.7)	13.9 (12.0-15.8)	2.04 (1.52-2.55)	0.438 (0.357-0.52)	0.343 (0.274-0.411)	11.8 (8.83-14.8)	3.85 (3.14-4.57)	3.39 (2.71-4.06)
**High-income**	*Overall*	**5 plus**	464 (375-554)	575 (510-640)	550 (486-615)	54.8 (44.2-65.4)	55.8 (49.5-62.2)	53.1 (46.8-59.3)	105 (80.7-130)	130 (114-145)	124 (109-139)	12.4 (9.52-15.3)	12.6 (11.1-14.1)	12.0 (10.5-13.4)
		**Under 5**	12.5 (10.2-14.9)	3.79 (3.31-4.26)	3.02 (2.54-3.49)	20.4 (16.5-24.2)	6.7 (5.86-7.55)	5.56 (4.68-6.44)	2.82 (2.18-3.46)	0.84 (0.721-0.96)	0.669 (0.556-0.781)	4.59 (3.55-5.63)	1.49 (1.28-1.7)	1.23 (1.02-1.44)
	**Australasia**	**5 plus**	6.06 (4.66-7.47)	8.88 (7.79-9.97)	9.09 (7.9-10.3)	32.4 (24.9-39.9)	31.6 (27.7-35.5)	31.2 (27.1-35.2)	1.29 (0.947-1.64)	1.96 (1.65-2.26)	1.96 (1.63-2.29)	6.89 (5.06-8.73)	6.97 (5.89-8.06)	6.74 (5.61-7.87)
		**Under 5**	0.171 (0.131-0.212)	0.0675 (0.057-0.0781)	0.0514 (0.0406-0.0622)	11.1 (8.49-13.7)	3.7 (3.12-4.27)	2.83 (2.23-3.43)	0.035 (0.0254-0.0446)	0.0143 (0.0115-0.017)	0.0107 (0.00816-0.0132)	2.27 (1.65-2.89)	0.78 (0.629-0.931)	0.588 (0.449-0.727)

	**HI Asia Pac**	**5 plus**	81.6 (67.5-95.7)	112 (95.2-128)	108 (91.8-125)	50.0 (41.4-58.6)	62.3 (53.1-71.5)	60.6 (51.3-69.9)	19.4 (14.8-23.9)	24.4 (20.7-28.1)	23.7 (20.0-27.3)	11.9 (9.09-14.7)	13.6 (11.6-15.7)	13.2 (11.2-15.3)
		**Under 5**	1.87 (1.51-2.24)	0.275 (0.245-0.304)	0.195 (0.173-0.218)	18.3 (14.7-21.9)	3.92 (3.5-4.34)	3.03 (2.68-3.38)	0.451 (0.338-0.563)	0.0605 (0.0525-0.0685)	0.0429 (0.0369-0.0489)	4.41 (3.31-5.51)	0.864 (0.75-0.978)	0.665 (0.572-0.758)
	**HI N Am**	**5 plus**	124 (98.4-150)	177 (160-194)	177 (159-195)	47.8 (37.9-57.8)	51.2 (46.1-56.2)	50.7 (45.5-55.8)	28.9 (21.7-36.0)	41.5 (37.0-46.0)	41.6 (37.0-46.2)	11.1 (8.36-13.9)	12.0 (10.7-13.3)	11.9 (10.6-13.2)
		**Under 5**	3.2 (2.49-3.91)	1.61 (1.38-1.84)	1.37 (1.15-1.59)	14.8 (11.5-18.0)	7.66 (6.57-8.76)	6.67 (5.59-7.76)	0.721 (0.537-0.905)	0.347 (0.293-0.402)	0.295 (0.245-0.346)	3.32 (2.48-4.17)	1.65 (1.39-1.91)	1.44 (1.19-1.69)
	**S Latin Am**	**5 plus**	28.3 (24.6-32.0)	51.4 (47.6-55.2)	43.7 (40.1-47.3)	63.8 (55.4-72.2)	82.7 (76.6-88.8)	68.9 (63.2-74.5)	6.84 (5.64-8.04)	12.7 (11.5-13.8)	10.9 (9.86-12.0)	15.4 (12.7-18.1)	20.4 (18.5-22.3)	17.2 (15.5-18.9)
		**Under 5**	4.02 (3.39-4.64)	0.953 (0.829-1.08)	0.685 (0.518-0.852)	78.1 (65.9-90.2)	20.2 (17.5-22.8)	16.0 (12.1-19.9)	0.912 (0.734-1.09)	0.231 (0.196-0.265)	0.169 (0.127-0.211)	17.7 (14.3-21.2)	4.88 (4.15-5.6)	3.94 (2.96-4.92)
	**W Europe**	**5 plus**	224 (179-270)	226 (198-254)	212 (185-239)	62.0 (49.5-74.6)	54.5 (47.8-61.3)	51.0 (44.4-57.5)	48.9 (37.3-60.5)	49.3 (42.8-55.8)	45.9 (39.9-52.0)	13.5 (10.3-16.7)	11.9 (10.3-13.5)	11.0 (9.58-12.5)
		**Under 5**	3.27 (2.55-3.98)	0.879 (0.764-0.994)	0.716 (0.594-0.838)	14.2 (11.1-17.3)	4.01 (3.49-4.54)	3.37 (2.8-3.95)	0.705 (0.527-0.882)	0.187 (0.16-0.215)	0.151 (0.123-0.178)	3.07 (2.3-3.84)	0.856 (0.729-0.984)	0.71 (0.58-0.84)
**Latin America and Caribbean**	*Overall*	**5 plus**	142 (121-163)	309 (279-339)	299 (265-334)	41.7 (35.6-47.7)	57.6 (52.1-63.1)	54.7 (48.5-61.0)	33.3 (26.8-39.8)	75.4 (66.5-84.4)	72.8 (63.2-82.3)	9.77 (7.86-11.7)	14.1 (12.4-15.7)	13.3 (11.6-15.1)
		**Under 5**	105 (87.1-123)	29.8 (23.1-36.4)	22.9 (17.2-28.6)	212 (176-248)	61.1 (47.5-74.7)	48.3 (36.3-60.4)	22.4 (17.4-27.4)	7.01 (5.4-8.61)	5.38 (4.01-6.76)	45.2 (35.1-55.4)	14.4 (11.1-17.7)	11.4 (8.47-14.3)
	**Andean Latin Am**	**5 plus**	18.3 (15.5-21.1)	36.6 (31.3-42.0)	32.2 (26.3-38.1)	55.9 (47.2-64.6)	63.4 (54.2-72.6)	53.7 (43.9-63.5)	4.14 (3.29-4.99)	8.56 (7.26-9.86)	7.52 (6.16-8.88)	12.7 (10.1-15.2)	14.8 (12.6-17.1)	12.5 (10.3-14.8)
		**Under 5**	19.9 (16.2-23.6)	4.51 (3.48-5.54)	3.25 (2.37-4.12)	377 (308-446)	73.8 (56.9-90.6)	52.8 (38.6-66.9)	4.18 (3.21-5.16)	1.02 (0.786-1.25)	0.732 (0.537-0.926)	79.2 (60.8-97.7)	16.6 (12.9-20.4)	11.9 (8.73-15.0)
	**Caribbean**	**5 plus**	15.1 (12.4-17.9)	28.0 (23.6-32.4)	27.2 (22.3-32.0)	48.6 (39.8-57.4)	65.1 (55.0-75.3)	62.4 (51.2-73.5)	3.66 (2.83-4.49)	6.79 (5.48-8.1)	6.6 (5.21-7.99)	11.8 (9.09-14.4)	15.8 (12.8-18.8)	15.1 (12.0-18.3)
		**Under 5**	10.6 (8.1-13.1)	6.15 (4.63-7.66)	5.44 (3.98-6.9)	257 (196-318)	157 (119-196)	141 (103-178)	2.33 (1.67-3.0)	1.37 (1.0-1.74)	1.22 (0.867-1.58)	56.5 (40.5-72.5)	35.1 (25.6-44.5)	31.7 (22.4-40.9)
	**Central Latin Am**	**5 plus**	53.4 (45.6-61.1)	113 (103-122)	113 (99.2-127)	37.7 (32.2-43.2)	49.1 (45.0-53.1)	48.5 (42.6-54.4)	12.6 (10.1-15.1)	27.7 (24.7-30.7)	27.7 (23.9-31.5)	8.91 (7.13-10.7)	12.1 (10.8-13.4)	11.9 (10.2-13.5)

		**Under 5**	41.9 (34.8-49.0)	11.8 (9.01-14.6)	8.69 (6.25-11.1)	182 (151-213)	55.5 (42.4-68.7)	43.3 (31.1-55.4)	9.13 (7.05-11.2)	2.8 (2.13-3.47)	2.06 (1.49-2.64)	39.7 (30.6-48.7)	13.2 (10.0-16.3)	10.3 (7.41-13.1)
	**Trop Latin Am**	**5 plus**	55.2 (47.3-63.0)	132 (118-146)	127 (112-142)	40.7 (34.9-46.5)	63.8 (57.0-70.7)	60.4 (53.3-67.5)	12.9 (10.4-15.3)	32.4 (28.0-36.7)	31.0 (26.6-35.3)	9.49 (7.68-11.3)	15.7 (13.6-17.8)	14.7 (12.7-16.8)
		**Under 5**	32.6 (26.5-38.7)	7.42 (5.6-9.23)	5.62 (4.21-7.02)	191 (155-226)	42.6 (32.2-53.1)	32.6 (24.5-40.8)	6.76 (5.19-8.32)	1.84 (1.39-2.3)	1.39 (1.03-1.75)	39.6 (30.4-48.7)	10.6 (7.96-13.2)	8.08 (5.99-10.2)
**North Africa and Middle East**		**5 plus**	107 (90.9-124)	196 (173-219)	192 (165-219)	37.3 (31.6-43.0)	36.2 (31.9-40.5)	34.1 (29.4-38.9)	26.6 (21.3-32.0)	52.7 (44.8-60.6)	51.3 (43.0-59.6)	9.25 (7.39-11.1)	9.71 (8.26-11.2)	9.14 (7.66-10.6)
		**Under 5**	157 (125-189)	46.5 (35.6-57.4)	34.4 (26.6-42.2)	307 (244-369)	72.9 (55.8-90.1)	56.3 (43.6-69.0)	36.4 (27.5-45.3)	12.1 (8.99-15.1)	8.87 (6.68-11.1)	71.1 (53.8-88.3)	18.9 (14.1-23.8)	14.5 (10.9-18.1)
**South Asia**		**5 plus**	577 (484-670)	1020 (905-1140)	1010 (866-1150)	61.6 (51.6-71.6)	62.1 (55.0-69.2)	59.6 (51.3-68.0)	130 (107-153)	275 (231-319)	271 (224-318)	13.9 (11.4-16.4)	16.7 (14.1-19.4)	16.1 (13.3-18.8)
		**Under 5**	823 (667-979)	329 (263-396)	258 (196-321)	524 (425-623)	202 (161-243)	163 (123-202)	178 (139-218)	80.4 (63.0-97.8)	63.5 (47.6-79.3)	114 (88.3-139)	49.3 (38.6-60.0)	40.0 (30.0-50.0)
**Southeast Asia, East Asia, and Oceania**	*Overall*	**5 plus**	673 (553-794)	1070 (924-1210)	1090 (953-1230)	44.5 (36.5-52.4)	53.0 (45.9-60.2)	53.4 (46.6-60.2)	158 (123-192)	254 (219-289)	257 (224-290)	10.4 (8.14-12.7)	12.6 (10.9-14.4)	12.5 (10.9-14.1)
		**Under 5**	439 (344-534)	74.0 (59.3-88.6)	59.7 (47.4-71.9)	251 (196-305)	49.4 (39.6-59.2)	43.1 (34.3-52.0)	91.7 (68.1-115)	16.9 (13.3-20.4)	13.7 (10.7-16.6)	52.4 (38.9-65.9)	11.3 (8.9-13.6)	9.89 (7.76-12.0)
	**E Asia**	**5 plus**	483 (389-577)	708 (587-829)	732 (604-859)	43.8 (35.3-52.4)	51.6 (42.8-60.4)	52.5 (43.4-61.7)	115 (87.9-141)	164 (136-192)	166 (137-194)	10.4 (7.98-12.8)	11.9 (9.91-14.0)	11.9 (9.87-13.9)
		**Under 5**	257 (194-319)	18.3 (14.8-21.8)	13.7 (10.8-16.6)	222 (168-276)	20.2 (16.4-24.1)	17.1 (13.5-20.7)	54.8 (39.7-70.0)	3.9 (3.11-4.69)	2.83 (2.21-3.45)	47.4 (34.3-60.4)	4.31 (3.43-5.18)	3.53 (2.76-4.31)
	**Oceania**	**5 plus**	2.37 (1.76-2.97)	4.98 (3.84-6.12)	5.01 (3.83-6.18)	42.7 (31.8-53.6)	43.6 (33.6-53.6)	41.7 (32.0-51.5)	0.546 (0.386-0.706)	1.22 (0.887-1.56)	1.23 (0.89-1.58)	9.84 (6.96-12.7)	10.7 (7.76-13.6)	10.3 (7.42-13.1)
		**Under 5**	3.83 (2.9-4.75)	4.7 (3.36-6.05)	4.05 (2.95-5.15)	381 (289-473)	253 (181-325)	209 (152-266)	0.815 (0.561-1.07)	1.04 (0.682-1.39)	0.897 (0.609-1.19)	81.1 (55.9-106)	55.7 (36.7-74.7)	46.4 (31.5-61.3)
	**SE Asia**	**5 plus**	188 (157-219)	354 (312-397)	356 (313-399)	46.2 (38.5-53.8)	56.5 (49.7-63.3)	55.5 (48.8-62.2)	42.8 (33.8-51.9)	89.1 (76.6-102)	89.7 (77.9-102)	10.5 (8.3-12.7)	14.2 (12.2-16.2)	14.0 (12.1-15.8)
		**Under 5**	179 (142-216)	51.1 (40.3-61.9)	42.1 (32.9-51.3)	307 (243-370)	89.3 (70.4-108)	74.8 (58.5-91.1)	36.1 (26.9-45.3)	12.0 (9.36-14.6)	9.98 (7.75-12.2)	61.9 (46.2-77.7)	20.9 (16.3-25.5)	17.7 (13.8-21.7)

**Sub-Saharan Africa**	*Overall*	**5 plus**	290 (244-335)	482 (405-559)	472 (395-550)	72.2 (60.8-83.5)	53.0 (44.5-61.4)	49.2 (41.1-57.3)	65.2 (51.5-78.9)	113 (90.8-134)	111 (88.8-132)	16.2 (12.8-19.6)	12.4 (9.98-14.8)	11.5 (9.25-13.8)
		**Under 5**	700 (546-855)	524 (394-654)	451 (325-577)	781 (609-953)	309 (233-386)	261 (188-334)	145 (106-184)	115 (84.3-145)	98.7 (69.4-128)	162 (118-205)	67.6 (49.7-85.4)	57.1 (40.1-74.1)
	**C Sub-Sah Africa**	**5 plus**	30.9 (25.0-36.9)	63.3 (49.2-77.3)	63.5 (49.3-77.7)	69.4 (56.0-82.7)	57.9 (45.1-70.8)	54.8 (42.6-67.0)	7.05 (5.28-8.83)	15.3 (11.0-19.6)	15.3 (11.0-19.6)	15.8 (11.8-19.8)	14.0 (10.1-17.9)	13.2 (9.52-16.9)
		**Under 5**	70.3 (52.4-88.2)	42.3 (29.5-55.1)	35.0 (23.4-46.6)	677 (505-849)	202 (141-264)	166 (111-221)	15.1 (10.5-19.7)	9.83 (6.64-13.0)	8.11 (5.19-11.0)	145 (101-189)	47.0 (31.7-62.3)	38.5 (24.6-52.4)
	**E Sub-Sah Africa**	**5 plus**	131 (111-150)	187 (158-215)	186 (157-215)	84.4 (71.9-97.0)	54.3 (46.1-62.6)	51.3 (43.3-59.3)	30.4 (24.3-36.5)	44.0 (35.2-52.7)	43.7 (34.9-52.5)	19.7 (15.7-23.6)	12.8 (10.2-15.3)	12.1 (9.62-14.5)
		**Under 5**	277 (219-335)	154 (117-191)	134 (96.9-171)	768 (607-929)	245 (186-304)	210 (152-268)	60.9 (45.0-76.8)	34.3 (25.6-43.0)	29.8 (21.1-38.5)	169 (125-213)	54.6 (40.7-68.5)	46.7 (33.1-60.3)
	**S Sub-Sah Africa**	**5 plus**	23.3 (19.3-27.4)	46.8 (40.5-53.1)	43.8 (37.6-50.1)	51.9 (42.9-61.0)	66.2 (57.3-75.2)	60.7 (52.0-69.4)	5.37 (4.15-6.59)	11.4 (9.35-13.5)	10.7 (8.67-12.8)	11.9 (9.23-14.7)	16.1 (13.2-19.0)	14.8 (12.0-17.7)
		**Under 5**	22.3 (17.8-26.9)	12.7 (10.1-15.4)	10.5 (7.84-13.2)	299 (238-359)	155 (123-187)	131 (97.6-164)	4.77 (3.55-5.99)	2.81 (2.17-3.45)	2.31 (1.68-2.94)	63.9 (47.6-80.2)	34.2 (26.5-42.0)	28.8 (20.9-36.6)
	**W Sub-Sah Africa**	**5 plus**	105 (85.0-125)	185 (149-221)	179 (142-216)	66.7 (54.0-79.4)	47.9 (38.5-57.3)	43.7 (34.7-52.8)	22.3 (17.1-27.6)	41.9 (32.9-51.0)	40.8 (31.7-49.9)	14.2 (10.9-17.5)	10.9 (8.52-13.2)	9.96 (7.73-12.2)
		**Under 5**	331 (253-408)	315 (235-396)	271 (194-349)	925 (709-1140)	407 (303-511)	339 (242-437)	64.1 (45.9-82.3)	67.6 (49.2-86.0)	58.5 (40.7-76.3)	179 (128-230)	87.2 (63.4-111)	73.1 (50.9-95.4)

Table S16: DALYs (in counts and all-age rates) associated with and attributable to bacterial antimicrobial resistance, for those under 5 years of age and over 5 years of age, globally, by GBD super-region, and region, for 1990, 2019, 2021

			DALYs Associated Counts (Thousands)			Rate Per 100K			Attributed Counts (Thousands)			Rate Per 100K		
**Super Region**	**Region**	**Age**	**1990**	**2019**	**2021**	**1990**	**2019**	**2021**	**1990**	**2019**	**2021**	**1990**	**2019**	**2021**
**Central Europe, Eastern Europe, and Central Asia**	*Overall*	**5 plus**	5940 (5050-6830)	6630 (5970-7280)	6280 (5590-6960)	1540 (1310-1770)	1690 (1530-1860)	1600 (1430-1780)	1320 (1050-1600)	1650 (1420-1890)	1550 (1320-1770)	344 (273-414)	422 (362-483)	395 (338-452)
		**Under 5**	4520 (3670-5360)	1210 (953-1470)	912 (708-1120)	12600 (10200-14900)	4480 (3530-5430)	3550 (2750-4340)	991 (731-1250)	287 (214-361)	216 (159-272)	2760 (2040-3490)	1060 (793-1330)	838 (618-1060)
	**C Asia**	**5 plus**	704 (577-832)	975 (814-1140)	936 (766-1110)	1180 (965-1390)	1170 (976-1360)	1090 (893-1290)	164 (122-206)	246 (189-303)	235 (179-292)	274 (204-344)	295 (226-364)	274 (208-340)
		**Under 5**	2950 (2360-3540)	984 (750-1220)	740 (554-925)	31000 (24800-37100)	9940 (7580-12300)	7400 (5550-9250)	666 (481-850)	233 (167-299)	174 (123-225)	6990 (5050-8920)	2360 (1690-3020)	1740 (1230-2250)
	**C Europe**	**5 plus**	1940 (1650-2230)	1800 (1640-1950)	1700 (1520-1880)	1680 (1420-1930)	1620 (1490-1760)	1550 (1390-1710)	448 (359-537)	426 (383-469)	401 (358-445)	386 (309-463)	385 (346-424)	366 (326-406)
		**Under 5**	622 (508-736)	61.5 (51.6-71.4)	43.1 (34.0-52.1)	6810 (5560-8060)	1070 (899-1240)	771 (609-933)	140 (106-174)	13.9 (11.4-16.4)	9.72 (7.58-11.9)	1530 (1160-1910)	242 (198-286)	174 (136-212)
	**E Europe**	**5 plus**	3290 (2810-3770)	3850 (3470-4230)	3640 (3210-4070)	1570 (1340-1800)	1950 (1760-2140)	1850 (1630-2070)	712 (568-855)	980 (825-1140)	911 (760-1060)	340 (271-409)	496 (417-575)	463 (387-540)
		**Under 5**	936 (769-1100)	162 (142-182)	127 (110-145)	5430 (4460-6400)	1420 (1250-1600)	1260 (1080-1430)	184 (138-230)	39.6 (32.2-46.9)	30.9 (24.8-37.1)	1070 (799-1340)	348 (283-412)	306 (245-366)
**High-income**	*Overall*	**5 plus**	9990 (8130-11800)	11400 (10400-12300)	10900 (9950-11900)	1180 (959-1400)	1100 (1010-1200)	1050 (959-1150)	2300 (1770-2830)	2630 (2370-2880)	2530 (2280-2770)	271 (208-334)	255 (230-280)	244 (220-267)
		**Under 5**	1130 (918-1340)	343 (300-386)	274 (231-317)	1840 (1490-2180)	608 (532-684)	505 (425-584)	255 (197-313)	76.3 (65.5-87.0)	60.8 (50.6-70.9)	415 (321-508)	135 (116-154)	112 (93.3-131)
	**Australasia**	**5 plus**	137 (106-168)	182 (163-201)	186 (165-207)	730 (563-896)	649 (580-717)	639 (566-711)	29.8 (21.9-37.7)	41.3 (35.6-47.1)	41.5 (35.2-47.9)	159 (117-201)	147 (127-168)	142 (121-164)
		**Under 5**	15.5 (11.8-19.1)	6.12 (5.17-7.08)	4.67 (3.69-5.65)	1000 (766-1240)	335 (283-387)	257 (203-311)	3.17 (2.3-4.03)	1.3 (1.05-1.55)	0.974 (0.746-1.2)	206 (149-262)	71.0 (57.3-84.6)	53.6 (41.1-66.2)
	**HI Asia Pac**	**5 plus**	1810 (1500-2110)	1900 (1690-2120)	1850 (1630-2070)	1110 (922-1290)	1060 (942-1180)	1030 (910-1160)	434 (332-535)	424 (372-476)	412 (359-464)	266 (204-328)	236 (207-265)	230 (201-259)

		**Under 5**	169 (136-202)	25.1 (22.4-27.7)	17.9 (15.9-19.9)	1650 (1330-1970)	358 (320-396)	278 (247-309)	40.6 (30.5-50.8)	5.57 (4.86-6.29)	3.98 (3.45-4.51)	398 (299-497)	79.6 (69.4-89.8)	61.7 (53.4-70.0)
	**HI N Am**	**5 plus**	2830 (2260-3400)	4070 (3750-4390)	4060 (3730-4390)	1090 (870-1310)	1180 (1090-1270)	1160 (1070-1260)	664 (498-830)	970 (876-1060)	969 (872-1070)	256 (192-320)	281 (253-308)	277 (249-305)
		**Under 5**	289 (225-352)	145 (125-166)	123 (103-143)	1330 (1040-1630)	690 (592-789)	601 (504-699)	65.1 (48.5-81.7)	31.3 (26.4-36.2)	26.7 (22.1-31.2)	300 (224-377)	149 (126-172)	130 (108-152)
	**S Latin Am**	**5 plus**	671 (585-757)	1070 (1010-1130)	934 (871-997)	1510 (1320-1710)	1720 (1620-1830)	1470 (1370-1570)	164 (136-192)	271 (249-293)	240 (219-260)	370 (306-433)	436 (401-471)	378 (346-410)
		**Under 5**	362 (306-419)	85.9 (74.7-97.0)	61.8 (46.8-76.8)	7040 (5940-8130)	1820 (1580-2050)	1440 (1090-1800)	82.3 (66.3-98.3)	20.8 (17.7-23.9)	15.2 (11.5-19.0)	1600 (1290-1910)	440 (375-505)	356 (268-444)
	**W Europe**	**5 plus**	4540 (3660-5430)	4140 (3730-4550)	3900 (3490-4320)	1260 (1010-1500)	999 (900-1100)	938 (838-1040)	1010 (773-1240)	921 (821-1020)	864 (767-961)	279 (214-344)	222 (198-247)	208 (184-231)
		**Under 5**	296 (231-361)	81.1 (70.5-91.7)	66.3 (54.8-77.8)	1290 (1000-1570)	371 (322-419)	312 (258-366)	63.8 (47.8-79.9)	17.3 (14.7-19.8)	13.9 (11.4-16.4)	278 (208-348)	78.8 (67.4-90.3)	65.6 (53.8-77.3)
**Latin America and Caribbean**	*Overall*	**5 plus**	4290 (3660-4910)	7670 (7050-8300)	7540 (6750-8320)	1260 (1070-1440)	1430 (1310-1550)	1380 (1230-1520)	1010 (814-1210)	1910 (1710-2110)	1870 (1640-2100)	297 (239-355)	356 (318-394)	342 (300-384)
		**Under 5**	9430 (7830-11000)	2680 (2080-3270)	2060 (1550-2570)	19000 (15800-22300)	5500 (4280-6720)	4350 (3260-5440)	2020 (1560-2470)	631 (486-775)	485 (361-608)	4070 (3160-4980)	1300 (999-1590)	1020 (764-1290)
	**Andean Latin Am**	**5 plus**	559 (472-645)	856 (732-980)	778 (632-923)	1710 (1440-1970)	1480 (1270-1700)	1300 (1050-1540)	127 (101-154)	203 (172-235)	185 (150-220)	389 (310-469)	352 (297-407)	308 (250-366)
		**Under 5**	1790 (1460-2110)	406 (314-498)	292 (214-371)	33800 (27600-40000)	6640 (5130-8150)	4750 (3470-6020)	376 (289-464)	91.7 (70.9-113)	65.9 (48.5-83.4)	7130 (5470-8780)	1500 (1160-1840)	1070 (787-1360)
	**Caribbean**	**5 plus**	429 (351-508)	723 (605-840)	701 (571-832)	1380 (1120-1630)	1680 (1410-1960)	1610 (1310-1910)	105 (80.9-129)	179 (144-214)	174 (136-211)	337 (260-415)	416 (334-497)	398 (313-484)
		**Under 5**	953 (727-1180)	552 (416-688)	489 (358-620)	23100 (17600-28500)	14100 (10700-17600)	12600 (9250-16000)	210 (150-269)	123 (90.0-156)	110 (78.0-142)	5070 (3640-6510)	3150 (2300-4000)	2850 (2020-3680)
	**Central Latin Am**	**5 plus**	1670 (1420-1920)	2990 (2770-3210)	3000 (2640-3370)	1180 (1010-1360)	1300 (1210-1400)	1290 (1130-1450)	398 (318-478)	748 (675-821)	748 (647-850)	282 (225-338)	326 (294-358)	321 (278-365)
		**Under 5**	3760 (3120-4400)	1060 (811-1310)	783 (563-1000)	16300 (13600-19100)	5000 (3820-6180)	3900 (2800-4990)	822 (635-1010)	252 (192-313)	186 (134-238)	3570 (2760-4380)	1190 (901-1470)	925 (668-1180)

	**Trop Latin Am**	**5 plus**	1630 (1400-1860)	3110 (2840-3370)	3060 (2760-3350)	1200 (1030-1370)	1510 (1380-1640)	1450 (1310-1590)	381 (309-454)	780 (687-873)	763 (668-857)	281 (228-335)	378 (333-423)	363 (318-408)
		**Under 5**	2930 (2380-3480)	668 (505-831)	506 (380-633)	17200 (13900-20400)	3840 (2900-4780)	2940 (2210-3680)	609 (469-750)	166 (125-207)	125 (93.1-158)	3570 (2740-4390)	956 (719-1190)	728 (541-916)
**North Africa and Middle East**		**5 plus**	3510 (2940-4070)	5880 (5140-6610)	5680 (4850-6510)	1220 (1020-1410)	1080 (948-1220)	1010 (862-1160)	870 (690-1050)	1590 (1340-1830)	1530 (1280-1790)	302 (240-365)	293 (248-338)	272 (227-318)
		**Under 5**	14100 (11200-17000)	4180 (3200-5160)	3090 (2400-3790)	27500 (21900-33100)	6560 (5020-8090)	5060 (3920-6200)	3270 (2480-4070)	1090 (810-1360)	798 (602-995)	6390 (4840-7940)	1700 (1270-2140)	1310 (984-1630)
**South Asia**		**5 plus**	22500 (18000-27100)	29600 (25800-33300)	29000 (24600-33300)	2410 (1920-2890)	1800 (1570-2030)	1720 (1460-1970)	4730 (3820-5640)	7720 (6370-9070)	7590 (6190-9000)	505 (408-603)	469 (387-551)	450 (367-533)
		**Under 5**	73800 (59900-87800)	29600 (23600-35600)	23200 (17600-28800)	47000 (38100-55900)	18200 (14500-21800)	14600 (11100-18200)	16000 (12500-19600)	7240 (5670-8800)	5710 (4290-7140)	10200 (7940-12500)	4440 (3480-5400)	3600 (2700-4500)
**Southeast Asia, East Asia, and Oceania**	*Overall*	**5 plus**	19100 (15600-22500)	25700 (22500-28900)	26000 (22900-29200)	1260 (1030-1490)	1280 (1120-1440)	1270 (1120-1430)	4440 (3460-5420)	6190 (5370-7000)	6190 (5420-6970)	293 (229-358)	307 (267-348)	303 (265-341)
		**Under 5**	39400 (30900-47900)	6660 (5350-7970)	5380 (4280-6480)	22500 (17600-27400)	4450 (3570-5320)	3890 (3090-4680)	8240 (6120-10400)	1520 (1200-1840)	1230 (970-1500)	4710 (3500-5910)	1020 (804-1230)	893 (701-1080)
	**E Asia**	**5 plus**	12900 (10400-15400)	15900 (13300-18600)	16300 (13300-19200)	1170 (944-1400)	1160 (965-1360)	1170 (958-1380)	3050 (2330-3780)	3730 (3100-4360)	3720 (3060-4390)	277 (211-343)	271 (225-317)	267 (220-315)
		**Under 5**	23000 (17400-28600)	1650 (1340-1960)	1240 (975-1500)	19900 (15100-24700)	1820 (1480-2170)	1540 (1220-1870)	4920 (3570-6280)	351 (280-422)	256 (200-312)	4250 (3080-5420)	388 (309-466)	319 (250-389)
	**Oceania**	**5 plus**	84.0 (59.8-108)	171 (128-214)	172 (128-217)	1520 (1080-1950)	1500 (1120-1880)	1440 (1070-1810)	19.2 (13.1-25.2)	41.6 (29.6-53.6)	41.9 (29.6-54.3)	346 (237-454)	364 (259-469)	350 (247-452)
		**Under 5**	344 (261-426)	422 (302-543)	364 (265-463)	34200 (26000-42400)	22700 (16300-29200)	18800 (13700-23900)	73.3 (50.5-96.0)	93.1 (61.4-125)	80.7 (54.8-107)	7300 (5030-9560)	5010 (3300-6720)	4170 (2830-5510)
	**SE Asia**	**5 plus**	6080 (5010-7140)	9620 (8480-10800)	9620 (8420-10800)	1490 (1230-1750)	1530 (1350-1720)	1500 (1310-1690)	1370 (1080-1670)	2420 (2090-2750)	2430 (2100-2760)	337 (265-409)	386 (332-439)	379 (328-430)
		**Under 5**	16000 (12700-19300)	4600 (3640-5570)	3790 (2970-4620)	27500 (21800-33200)	8040 (6340-9730)	6740 (5270-8200)	3240 (2420-4070)	1080 (845-1310)	901 (700-1100)	5570 (4150-6980)	1880 (1470-2290)	1600 (1240-1960)
**Sub-Saharan Africa**	*Overall*	**5 plus**	10800 (8920-12700)	17100 (14100-20200)	16900 (13800-20000)	2700 (2220-3170)	1880 (1550-2220)	1760 (1430-2090)	2430 (1890-2970)	3970 (3150-4780)	3920 (3090-4750)	606 (472-740)	436 (346-526)	408 (322-495)

		**Under 5**	62600 (48800-76400)	47000 (35400-58600)	40400 (29200-51600)	69800 (54400-85200)	27700 (20900-34600)	23400 (16900-29900)	13000 (9480-16500)	10300 (7570-13000)	8850 (6230-11500)	14500 (10600-18400)	6060 (4470-7660)	5120 (3600-6640)
	**C Sub-Sah Africa**	**5 plus**	1150 (917-1370)	2270 (1750-2780)	2270 (1740-2800)	2570 (2060-3080)	2070 (1600-2540)	1960 (1500-2410)	260 (193-328)	547 (392-702)	546 (389-703)	584 (433-735)	501 (359-642)	471 (336-607)
		**Under 5**	6290 (4700-7890)	3800 (2650-4940)	3140 (2100-4180)	60600 (45200-76000)	18200 (12700-23700)	14900 (9990-19900)	1350 (942-1760)	883 (597-1170)	728 (466-991)	13000 (9070-16900)	4220 (2850-5590)	3460 (2210-4700)
	**E Sub-Sah Africa**	**5 plus**	5110 (4240-5980)	6710 (5600-7820)	6720 (5570-7870)	3300 (2740-3870)	1950 (1630-2270)	1850 (1540-2170)	1190 (938-1450)	1570 (1240-1900)	1580 (1240-1910)	772 (606-937)	456 (360-552)	435 (341-528)
		**Under 5**	24800 (19600-30000)	13800 (10500-17200)	12000 (8700-15400)	68700 (54300-83100)	22000 (16700-27300)	18900 (13600-24100)	5460 (4040-6880)	3080 (2300-3860)	2680 (1900-3460)	15100 (11200-19100)	4900 (3660-6150)	4200 (2980-5420)
	**S Sub-Sah Africa**	**5 plus**	832 (685-979)	1490 (1280-1710)	1430 (1210-1650)	1850 (1530-2180)	2110 (1810-2420)	1980 (1670-2280)	193 (149-237)	366 (292-440)	352 (276-428)	429 (331-527)	518 (413-623)	488 (382-593)
		**Under 5**	2010 (1600-2410)	1140 (906-1380)	943 (705-1180)	26800 (21400-32300)	13900 (11000-16800)	11700 (8780-14700)	430 (321-539)	253 (196-310)	208 (151-264)	5750 (4290-7220)	3080 (2380-3780)	2580 (1880-3290)
	**W Sub-Sah Africa**	**5 plus**	3750 (2940-4560)	6660 (5160-8170)	6480 (4910-8060)	2380 (1870-2890)	1730 (1340-2120)	1580 (1200-1970)	786 (590-982)	1480 (1130-1840)	1450 (1080-1810)	499 (375-624)	385 (293-476)	354 (264-443)
		**Under 5**	29500 (22600-36400)	28200 (21000-35400)	24300 (17300-31200)	82500 (63300-102000)	36400 (27100-45700)	30400 (21700-39100)	5740 (4110-7360)	6060 (4410-7700)	5240 (3650-6830)	16000 (11500-20600)	7810 (5690-9930)	6550 (4570-8540)

Table S17: Deaths (in counts and all-age rates) associated with and attributable to bacterial antimicrobial resistance, by GBD region, for 1990, 2019, 2021

	Deaths															
	Associated Counts (Thousands)				Rate Per 100K				Attributed Counts (Thousands)				Rate Per 100K			
**Region**	**1990**	**2019**	**2021**	**2050**	**1990**	**2019**	**2021**	**2050**	**1990**	**2019**	**2021**	**2050**	**1990**	**2019**	**2021**	**2050**
**Andean Latin America**	38.2 (32.1-44.3)	41.2 (35.2-47.1)	35.4 (29.0-41.9)	71.8 (54.2-100)	101 (84.4-117)	64.4 (55.1-73.7)	53.6 (43.8-63.4)	89.5 (69.0-123)	8.33 (6.56-10.1)	9.58 (8.13-11.0)	8.25 (6.76-9.74)	16.0 (12.1-22.0)	21.9 (17.3-26.6)	15.0 (12.7-17.3)	12.5 (10.2-14.7)	19.9 (15.4-27.2)
**Australasia**	6.24 (4.8-7.67)	8.95 (7.85-10.0)	9.14 (7.95-10.3)	19.9 (15.1-23.9)	30.8 (23.7-37.8)	29.9 (26.2-33.5)	29.5 (25.7-33.3)	47.3 (35.6-56.3)	1.33 (0.974-1.68)	1.97 (1.67-2.28)	1.97 (1.64-2.3)	4.1 (3.03-5.08)	6.54 (4.8-8.28)	6.59 (5.57-7.62)	6.38 (5.31-7.44)	9.74 (7.15-12.0)
**Caribbean**	25.8 (20.7-30.8)	34.1 (28.7-39.6)	32.6 (26.6-38.6)	54.0 (42.8-67.3)	73.0 (58.6-87.4)	72.8 (61.1-84.5)	68.7 (56.1-81.4)	109 (87.5-135)	6.0 (4.54-7.45)	8.16 (6.55-9.77)	7.83 (6.14-9.51)	12.2 (9.33-16.1)	17.0 (12.9-21.1)	17.4 (14.0-20.8)	16.5 (12.9-20.0)	24.5 (19.1-31.6)
**Central Asia**	55.4 (45.1-65.8)	43.3 (35.8-50.8)	39.5 (32.3-46.7)	65.8 (51.4-84.3)	80.0 (65.0-94.9)	46.4 (38.3-54.5)	41.2 (33.7-48.7)	54.2 (41.6-69.4)	12.6 (9.31-16.0)	10.6 (8.24-13.0)	9.68 (7.47-11.9)	15.2 (11.4-20.2)	18.2 (13.4-23.1)	11.4 (8.84-14.0)	10.1 (7.8-12.4)	12.5 (9.4-16.4)
**Central Europe**	88.8 (75.0-103)	84.6 (76.6-92.5)	80.2 (71.4-89.1)	95.3 (80.4-109)	71.0 (60.0-82.0)	72.7 (65.9-79.5)	69.6 (61.9-77.3)	101 (84.3-116)	20.3 (16.1-24.5)	19.8 (17.6-21.9)	18.7 (16.5-20.8)	21.2 (17.8-24.5)	16.2 (12.9-19.5)	17.0 (15.1-18.9)	16.2 (14.3-18.1)	22.4 (18.7-25.8)
**Central Latin America**	95.2 (81.0-109)	124 (114-135)	122 (106-137)	241 (202-281)	57.9 (49.3-66.6)	49.6 (45.2-54.0)	48.1 (42.0-54.2)	84.6 (70.1-101)	21.7 (17.2-26.2)	30.5 (27.1-33.9)	29.7 (25.5-34.0)	55.8 (46.4-67.3)	13.2 (10.5-15.9)	12.2 (10.8-13.5)	11.8 (10.1-13.4)	19.6 (16.1-24.0)
**Central Sub-Saharan Africa**	101 (80.8-122)	106 (82.3-129)	98.5 (76.1-121)	177 (125-242)	184 (147-221)	81.1 (63.3-99.0)	71.9 (55.6-88.3)	70.7 (52.7-92.5)	22.1 (16.3-27.9)	25.1 (18.5-31.8)	23.4 (17.0-29.9)	40.1 (27.0-56.9)	40.3 (29.7-50.8)	19.3 (14.2-24.4)	17.1 (12.4-21.8)	16.0 (11.6-21.3)
**Eastern Asia**	740 (602-877)	727 (606-848)	745 (618-872)	1110 (838-1490)	60.8 (49.4-72.1)	49.6 (41.4-57.9)	50.6 (42.0-59.2)	86.1 (65.6-114)	169 (131-208)	168 (140-196)	169 (140-197)	233 (177-311)	13.9 (10.8-17.1)	11.5 (9.55-13.4)	11.4 (9.52-13.4)	18.2 (13.9-24.0)
**Eastern Europe**	141 (120-162)	153 (137-168)	145 (127-163)	199 (159-239)	62.2 (52.8-71.7)	73.1 (65.6-80.6)	70.2 (61.6-78.7)	110 (90.2-130)	30.1 (23.9-36.4)	38.2 (32.6-43.7)	35.6 (30.1-41.1)	46.5 (36.1-56.8)	13.3 (10.5-16.1)	18.3 (15.6-20.9)	17.2 (14.6-19.9)	25.6 (20.5-30.5)
**Eastern Sub-Saharan Africa**	408 (334-482)	341 (281-401)	320 (261-379)	509 (405-637)	214 (175-252)	83.8 (69.1-98.5)	75.1 (61.2-89.0)	64.6 (50.2-81.0)	91.3 (70.0-113)	78.2 (62.6-93.9)	73.5 (57.8-89.2)	113 (87.6-145)	47.9 (36.7-59.0)	19.2 (15.4-23.1)	17.3 (13.6-20.9)	14.4 (10.9-18.2)
**High-income Asia Pacific**	83.5 (69.1-97.8)	112 (95.5-129)	109 (92.0-125)	158 (110-205)	48.1 (39.9-56.4)	60.1 (51.2-69.0)	58.6 (49.6-67.6)	96.2 (66.9-123)	19.8 (15.2-24.4)	24.5 (20.8-28.2)	23.7 (20.1-27.4)	34.5 (24.1-44.1)	11.4 (8.76-14.1)	13.1 (11.2-15.1)	12.8 (10.8-14.8)	21.1 (14.6-27.2)
**High-income North America**	127 (101-154)	179 (161-196)	179 (161-196)	296 (225-357)	45.3 (35.9-54.7)	48.7 (43.9-53.4)	48.2 (43.4-53.1)	73.4 (56.6-88.3)	29.6 (22.3-36.9)	41.8 (37.3-46.3)	41.9 (37.3-46.5)	65.3 (48.7-79.3)	10.5 (7.92-13.1)	11.4 (10.2-12.6)	11.3 (10.1-12.6)	16.2 (12.2-19.6)
**Oceania**	6.19 (4.81-7.58)	9.68 (7.45-11.9)	9.06 (7.05-11.1)	18.3 (12.7-24.8)	94.6 (73.4-116)	72.9 (56.1-89.7)	65.0 (50.6-79.4)	68.9 (49.0-91.2)	1.36 (0.971-1.75)	2.26 (1.62-2.89)	2.13 (1.55-2.71)	4.21 (2.73-5.88)	20.8 (14.8-26.7)	17.0 (12.2-21.7)	15.3 (11.2-19.4)	15.8 (10.6-22.1)

**Southeast Asia**	367 (303-431)	406 (357-454)	398 (351-446)	815 (692-953)	78.8 (65.0-92.6)	59.2 (52.2-66.3)	57.0 (50.2-63.8)	102 (86.8-121)	78.9 (61.4-96.5)	101 (87.0-115)	99.7 (86.5-113)	191 (160-226)	17.0 (13.2-20.7)	14.8 (12.7-16.8)	14.3 (12.4-16.2)	23.9 (20.2-28.6)
**Southern Latin America**	32.3 (28.2-36.5)	52.3 (48.5-56.1)	44.4 (40.7-48.0)	85.7 (69.9-105)	65.3 (56.8-73.7)	78.3 (72.6-84.0)	65.5 (60.2-70.9)	119 (96.1-143)	7.75 (6.4-9.1)	12.9 (11.7-14.1)	11.1 (10.0-12.2)	20.2 (16.2-25.0)	15.6 (12.9-18.4)	19.3 (17.5-21.1)	16.4 (14.8-18.0)	28.0 (22.8-34.1)
**Southern Sub-Saharan Africa**	45.7 (37.6-53.8)	59.5 (51.1-67.9)	54.3 (46.2-62.5)	71.4 (58.5-86.7)	87.1 (71.7-103)	75.5 (64.8-86.1)	67.7 (57.6-77.8)	72.4 (60.9-85.4)	10.1 (7.79-12.5)	14.2 (11.7-16.7)	13.0 (10.5-15.5)	16.2 (13.2-20.0)	19.4 (14.9-23.8)	18.0 (14.8-21.2)	16.2 (13.1-19.3)	16.4 (13.6-20.0)
**Tropical Latin America**	87.7 (74.8-101)	139 (124-154)	133 (117-148)	283 (205-390)	57.5 (49.0-66.0)	62.2 (55.6-68.8)	58.3 (51.6-65.0)	110 (81.8-146)	19.6 (15.8-23.5)	34.2 (29.6-38.8)	32.4 (27.9-36.9)	64.4 (45.7-88.4)	12.9 (10.3-15.4)	15.3 (13.2-17.3)	14.2 (12.2-16.2)	25.0 (18.5-32.4)
**Western Europe**	228 (182-273)	227 (199-255)	213 (185-240)	324 (251-376)	59.2 (47.3-71.1)	52.0 (45.6-58.4)	48.6 (42.4-54.9)	72.0 (54.7-83.0)	49.6 (37.9-61.4)	49.5 (43.0-56.0)	46.1 (40.0-52.1)	67.7 (51.1-78.8)	12.9 (9.85-16.0)	11.3 (9.85-12.8)	10.5 (9.15-11.9)	15.0 (11.4-17.7)
**Western Sub-Saharan Africa**	435 (341-530)	500 (389-612)	451 (341-561)	709 (513-939)	225 (177-274)	108 (83.9-132)	92.0 (69.5-114)	72.4 (53.8-95.6)	86.5 (63.5-109)	110 (83.2-136)	99.3 (73.4-125)	153 (111-207)	44.8 (32.9-56.7)	23.6 (18.0-29.3)	20.3 (15.0-25.6)	15.7 (11.4-21.3)

Table 18: DALYs (in counts and all-age rates) associated with and attributable to bacterial antimicrobial resistance, by GBD region, for 1990, 2019, 2021

	DALYs Associated Counts (Thousands)				Rate Per 100K				Attributed Counts (Thousands)				Rate Per 100K			
**Region**	**1990**	**2019**	**2021**	**2050**	**1990**	**2019**	**2021**	**2050**	**1990**	**2019**	**2021**	**2050**	**1990**	**2019**	**2021**	**2050**
**Andean Latin America**	2350 (1940-2750)	1260 (1070-1460)	1070 (865-1270)	1390 (1050-1880)	6170 (5120-7230)	1970 (1670-2280)	1620 (1310-1930)	1730 (1330-2330)	504 (393-615)	295 (248-342)	251 (203-298)	311 (240-419)	1330 (1030-1620)	462 (388-535)	379 (307-451)	387 (295-526)
**Australasia**	152 (118-187)	188 (169-208)	191 (170-212)	282 (227-331)	751 (581-920)	629 (565-694)	616 (548-685)	670 (537-778)	33.0 (24.3-41.6)	42.6 (36.7-48.6)	42.5 (36.1-48.9)	58.4 (45.3-71.1)	163 (120-205)	142 (123-162)	137 (116-158)	139 (108-169)
**Caribbean**	1380 (1090-1680)	1270 (1040-1510)	1190 (944-1440)	1240 (950-1660)	3920 (3080-4760)	2720 (2220-3220)	2510 (1990-3030)	2500 (1940-3240)	315 (233-397)	302 (238-366)	284 (218-350)	281 (207-379)	892 (659-1120)	644 (507-780)	598 (458-737)	566 (427-750)
**Central Asia**	3660 (2950-4360)	1960 (1590-2330)	1680 (1350-2000)	1830 (1340-2490)	5270 (4260-6290)	2100 (1710-2490)	1750 (1410-2080)	1500 (1100-2010)	830 (605-1050)	479 (365-593)	410 (311-508)	426 (302-605)	1200 (873-1520)	514 (391-636)	428 (325-530)	351 (246-492)
**Central Europe**	2560 (2170-2960)	1860 (1700-2010)	1740 (1560-1930)	1610 (1400-1840)	2050 (1740-2360)	1600 (1460-1730)	1510 (1350-1670)	1700 (1490-1950)	588 (467-709)	440 (395-484)	411 (366-456)	361 (310-421)	470 (373-567)	378 (340-416)	357 (317-396)	381 (330-443)
**Central Latin America**	5440 (4580-6290)	4050 (3630-4470)	3790 (3250-4320)	4980 (4100-5990)	3310 (2780-3830)	1620 (1450-1780)	1500 (1280-1710)	1750 (1430-2130)	1220 (958-1480)	1000 (881-1120)	934 (792-1080)	1160 (938-1440)	742 (583-901)	399 (351-446)	369 (313-426)	410 (329-508)
**Central Sub-Saharan Africa**	7440 (5740-9130)	6060 (4560-7570)	5410 (3990-6840)	6390 (4260-9010)	13500 (10400-16600)	4660 (3500-5820)	3950 (2910-4990)	2550 (1780-3520)	1610 (1160-2070)	1430 (1030-1830)	1270 (891-1660)	1440 (937-2100)	2930 (2110-3760)	1100 (791-1410)	931 (651-1210)	575 (380-822)
**Eastern Asia**	36000 (28700-43200)	17600 (14900-20300)	17500 (14600-20400)	17500 (13400-23700)	2950 (2350-3550)	1200 (1020-1390)	1190 (991-1390)	1360 (1040-1830)	7970 (6070-9880)	4080 (3440-4720)	3980 (3310-4650)	3710 (2830-4960)	655 (498-812)	279 (235-322)	270 (225-315)	289 (222-385)
**Eastern Europe**	4230 (3610-4850)	4010 (3620-4410)	3770 (3330-4210)	3870 (3100-4690)	1870 (1590-2140)	1920 (1730-2110)	1820 (1610-2030)	2130 (1770-2550)	896 (710-1080)	1020 (858-1180)	942 (786-1100)	917 (718-1120)	395 (313-477)	488 (411-565)	456 (380-531)	505 (404-613)
**Eastern Sub-Saharan Africa**	29900 (24000-35800)	20500 (16300-24700)	18700 (14500-23000)	19600 (14500-27100)	15700 (12600-18800)	5050 (4010-6080)	4400 (3410-5390)	2490 (1860-3430)	6660 (5010-8300)	4650 (3620-5680)	4250 (3220-5290)	4320 (3120-5990)	3490 (2630-4350)	1140 (890-1400)	998 (755-1240)	548 (393-761)
**High-income Asia Pacific**	1970 (1650-2300)	1930 (1720-2140)	1870 (1650-2090)	1930 (1470-2450)	1140 (952-1330)	1040 (920-1150)	1010 (888-1130)	1180 (883-1470)	474 (364-584)	430 (377-482)	416 (363-468)	424 (318-530)	274 (210-337)	230 (203-258)	224 (196-252)	259 (191-322)
**High-income North America**	3120 (2500-3740)	4220 (3900-4530)	4180 (3860-4510)	4840 (3890-5670)	1110 (887-1330)	1150 (1060-1240)	1130 (1040-1220)	1200 (992-1390)	729 (549-910)	1000 (906-1100)	996 (898-1090)	1080 (857-1270)	259 (195-323)	273 (247-299)	269 (243-296)	267 (213-313)
**Oceania**	428 (327-528)	594 (442-745)	536 (407-665)	787 (497-1180)	6530 (4990-8060)	4470 (3330-5610)	3850 (2920-4780)	2960 (1870-4290)	92.4 (64.8-120)	135 (93.7-176)	123 (87.4-158)	176 (106-264)	1410 (989-1830)	1010 (705-1320)	881 (628-1130)	661 (401-970)

**Southeast Asia**	22100 (17900-26300)	14200 (12400-16000)	13400 (11700-15200)	17000 (14300-20100)	4750 (3850-5650)	2080 (1810-2340)	1920 (1670-2170)	2130 (1800-2550)	4620 (3530-5710)	3500 (3000-4000)	3330 (2860-3810)	3980 (3310-4760)	992 (758-1230)	511 (438-584)	477 (409-545)	500 (417-604)
**Southern Latin America**	1030 (902-1160)	1160 (1090-1220)	996 (930-1060)	1410 (1160-1700)	2090 (1820-2350)	1730 (1630-1830)	1470 (1370-1570)	1950 (1640-2310)	246 (204-289)	292 (269-315)	255 (233-277)	337 (278-409)	497 (412-583)	436 (402-471)	376 (344-409)	467 (395-559)
**Southern Sub-Saharan Africa**	2840 (2310-3370)	2630 (2210-3060)	2370 (1960-2780)	2160 (1690-2730)	5410 (4400-6430)	3340 (2810-3870)	2950 (2440-3470)	2190 (1750-2690)	623 (474-772)	619 (500-738)	560 (441-679)	488 (376-640)	1190 (904-1470)	784 (634-935)	697 (549-846)	494 (386-627)
**Tropical Latin America**	4560 (3820-5290)	3780 (3420-4130)	3560 (3220-3910)	5000 (3760-6800)	2990 (2510-3470)	1690 (1530-1850)	1570 (1410-1720)	1940 (1520-2500)	990 (785-1200)	947 (828-1070)	888 (776-1000)	1150 (840-1550)	649 (515-784)	423 (370-476)	390 (341-440)	448 (343-575)
**Western Europe**	4840 (3900-5780)	4220 (3810-4630)	3970 (3550-4380)	4360 (3560-4950)	1260 (1010-1500)	967 (873-1060)	907 (813-1000)	969 (779-1100)	1070 (823-1320)	938 (837-1040)	878 (781-975)	915 (741-1050)	279 (214-344)	215 (192-238)	201 (178-223)	203 (163-235)
**Western Sub-Saharan Africa**	33200 (25700-40800)	34900 (26400-43400)	30800 (22500-39100)	33900 (22300-48900)	17200 (13300-21100)	7530 (5700-9360)	6280 (4590-7980)	3460 (2360-5030)	6520 (4720-8330)	7540 (5590-9490)	6690 (4780-8600)	7230 (4670-10400)	3380 (2440-4310)	1630 (1210-2050)	1370 (976-1760)	738 (495-1090)
