# Supplementary material for: Global burden of bacterial antimicrobial resistance 1990–2021: a systematic analysis with forecasts to 2050
Source: Lancet. 2024 Sep 28;404(10459):1199–226. doi: 10.1016/S0140-6736(24)01867-1 (PMC11718157; doi:10.1016/S0140-6736(24)01867-1)
Supplement: Supplementary appendix 2 [file mmc2.pdf]

THE LANCET

Supplementary appendix 2

This appendix formed part of the original submission and has been peer reviewed. We post it as supplied by the authors.

Supplement to: GBD 2021 Antimicrobial Resistance Collaborators. Global burden of bacterial antimicrobial resistance 1990–2021: a systematic analysis with forecasts to 2050. *Lancet* 2024; published online Sept 16. https://doi.org/10.1016/S0140-6736(24)01867-1.

Appendix Table S1: GBD cause hierarchy with levels

All causes	level
Communicable, maternal, neonatal, and nutritional diseases	0
	1
HIV/AIDS and sexually transmitted infections	2
HIV/AIDS	3
HIV/AIDS - Drug-susceptible Tuberculosis	4
extensive drug resistance	4
HIV/AIDS - Extensively drug-resistant Tuberculosis	4
HIV/AIDS resulting in other diseases	4
Sexually transmitted infections excluding HIV	3
Syphilis	4
Chlamydial infection	4
Gonococcal infection	4
Trichomoniasis	4
Genital herpes	4
Other sexually transmitted infections	4
Respiratory infections and tuberculosis	2
Tuberculosis	3
Latent tuberculosis infection	4
Drug-susceptible tuberculosis	4
resistance	4
Extensively drug-resistant tuberculosis	4
Lower respiratory infections	3
Upper respiratory infections	3
Otitis media	3
COVID-19	3
Enteric infections	2
Diarrheal diseases	3
Typhoid and paratyphoid	3
Typhoid fever	4
Paratyphoid fever	4
Invasive Non-typhoidal Salmonella (iNTS)	3
Other intestinal infectious diseases	3
Neglected tropical diseases and malaria	2
Malaria	3
Chagas disease	3
Leishmaniasis	3
Visceral leishmaniasis	4
Cutaneous and mucocutaneous leishmaniasis	4
African trypanosomiasis	3
Schistosomiasis	3
Cysticercosis	3
Cystic echinococcosis	3
Lymphatic filariasis	3
Onchocerciasis	3

Trachoma	3
Dengue	3
Yellow fever	3
Rabies	3
Intestinal nematode infections	3
Ascariasis	4
Trichuriasis	4
Hookworm disease	4
Food-borne trematodiases	3
Leprosy	3
Ebola	3
Zika virus	3
Guinea worm disease	3
Other neglected tropical diseases	3
Other infectious diseases	2
Meningitis	3
Encephalitis	3
Diphtheria	3
Pertussis	3
Tetanus	3
Measles	3
Varicella and herpes zoster	3
Acute hepatitis	3
Acute hepatitis A	4
Acute hepatitis B	4
Acute hepatitis C	4
Acute hepatitis E	4
Other unspecified infectious diseases	3
Maternal and neonatal disorders	2
Maternal disorders	3
Maternal hemorrhage	4
Maternal sepsis and other maternal infections	4
Maternal hypertensive disorders	4
Maternal obstructed labor and uterine rupture	4
Maternal abortion and miscarriage	4
Ectopic pregnancy	4
Indirect maternal deaths	4
Late maternal deaths	4
Maternal deaths aggravated by HIV/AIDS	4
Other direct maternal disorders	4
Neonatal disorders	3
Neonatal preterm birth	4
Neonatal encephalopathy due to birth asphyxia and trauma	4
Neonatal sepsis and other neonatal infections	4
Hemolytic disease and other neonatal jaundice	4
Other neonatal disorders	4

Nutritional deficiencies	2
Protein-energy malnutrition	3
Iodine deficiency	3
Vitamin A deficiency	3
Dietary iron deficiency	3
Other nutritional deficiencies	3
Non-communicable diseases	1
Neoplasms	2
Lip and oral cavity cancer	3
Nasopharynx cancer	3
Other pharynx cancer	3
Esophageal cancer	3
Stomach cancer	3
Colon and rectum cancer	3
Liver cancer	3
Liver cancer due to hepatitis B	4
Liver cancer due to hepatitis C	4
Liver cancer due to alcohol use	4
Liver cancer due to NASH	4
Hepatoblastoma	4
Liver cancer due to other causes	4
Gallbladder and biliary tract cancer	3
Pancreatic cancer	3
Larynx cancer	3
Tracheal, bronchus, and lung cancer	3
Malignant skin melanoma	3
Non-melanoma skin cancer	3
Non-melanoma skin cancer (squamous-cell carcinoma)	4
Non-melanoma skin cancer (basal-cell carcinoma)	4
Soft tissue and other extraosseous sarcomas	3
Malignant neoplasm of bone and articular cartilage	3
Breast cancer	3
Cervical cancer	3
Uterine cancer	3
Ovarian cancer	3
Prostate cancer	3
Testicular cancer	3
Kidney cancer	3
Bladder cancer	3
Brain and central nervous system cancer	3
Eye cancer	3
Retinoblastoma	4
Other eye cancers	4
Neuroblastoma and other peripheral nervous cell tumors	3
Thyroid cancer	3
Mesothelioma	3

Hodgkin lymphoma	3
Non-Hodgkin lymphoma	3
Burkitt lymphoma	4
Other non-Hodgkin lymphoma	4
Multiple myeloma	3
Leukemia	3
Acute lymphoid leukemia	4
Chronic lymphoid leukemia	4
Acute myeloid leukemia	4
Chronic myeloid leukemia	4
Other leukemia	4
Other malignant neoplasms	3
Other neoplasms	3
neoplasms	4
Benign and in situ intestinal neoplasms	4
Benign and in situ cervical and uterine neoplasms	4
Other benign and in situ neoplasms	4
Cardiovascular diseases	2
Rheumatic heart disease	3
Ischemic heart disease	3
Stroke	3
Ischemic stroke	4
Intracerebral hemorrhage	4
Subarachnoid hemorrhage	4
Hypertensive heart disease	3
Non-rheumatic valvular heart disease	3
Non-rheumatic calcific aortic valve disease	4
Non-rheumatic degenerative mitral valve disease	4
Other non-rheumatic valve diseases	4
Cardiomyopathy and myocarditis	3
Myocarditis	4
Alcoholic cardiomyopathy	4
Other cardiomyopathy	4
Pulmonary Arterial Hypertension	3
Atrial fibrillation and flutter	3
Aortic aneurysm	3
Lower extremity peripheral arterial disease	3
Endocarditis	3
Other cardiovascular and circulatory diseases	3
Chronic respiratory diseases	2
Chronic obstructive pulmonary disease	3
Pneumoconiosis	3
Silicosis	4
Asbestosis	4
Coal workers pneumoconiosis	4
Other pneumoconiosis	4

Asthma	3
Interstitial lung disease and pulmonary sarcoidosis	3
Other chronic respiratory diseases	3
Digestive diseases	2
Cirrhosis and other chronic liver diseases	3
Chronic hepatitis B including cirrhosis	4
Chronic hepatitis C including cirrhosis	4
Cirrhosis due to alcohol	4
Nonalcoholic fatty liver disease including cirrhosis	4
Cirrhosis due to other causes	4
Upper digestive system diseases	3
Peptic ulcer disease	4
Gastritis and duodenitis	4
Gastroesophageal reflux disease	4
Appendicitis	3
Paralytic ileus and intestinal obstruction	3
Inguinal, femoral, and abdominal hernia	3
Inflammatory bowel disease	3
Vascular intestinal disorders	3
Gallbladder and biliary diseases	3
Pancreatitis	3
Other digestive diseases	3
Neurological disorders	2
Alzheimer's disease and other dementias	3
Parkinson's disease	3
Idiopathic epilepsy	3
Multiple sclerosis	3
Motor neuron disease	3
Headache disorders	3
Migraine	4
Tension-type headache	4
Other neurological disorders	3
Mental disorders	2
Schizophrenia	3
Depressive disorders	3
Major depressive disorder	4
Dysthymia	4
Bipolar disorder	3
Anxiety disorders	3
Eating disorders	3
Anorexia nervosa	4
Bulimia nervosa	4
Autism spectrum disorders	3
Attention-deficit/hyperactivity disorder	3
Conduct disorder	3
Idiopathic developmental intellectual disability	3

Other mental disorders	3
Substance use disorders	2
Alcohol use disorders	3
Drug use disorders	3
Opioid use disorders	4
Cocaine use disorders	4
Amphetamine use disorders	4
Cannabis use disorders	4
Other drug use disorders	4
Diabetes and kidney diseases	2
Diabetes mellitus	3
Diabetes mellitus type 1	4
Diabetes mellitus type 2	4
Chronic kidney disease	3
Chronic kidney disease due to diabetes mellitus type 1	4
Chronic kidney disease due to diabetes mellitus type 2	4
Chronic kidney disease due to hypertension	4
Chronic kidney disease due to glomerulonephritis	4
Chronic kidney disease due to other and unspecified causes	4
Acute glomerulonephritis	3
Skin and subcutaneous diseases	2
Dermatitis	3
Atopic dermatitis	4
Contact dermatitis	4
Seborrhoeic dermatitis	4
Psoriasis	3
Bacterial skin diseases	3
Cellulitis	4
Pyoderma	4
Scabies	3
Fungal skin diseases	3
Viral skin diseases	3
Acne vulgaris	3
Alopecia areata	3
Pruritus	3
Urticaria	3
Decubitus ulcer	3
Other skin and subcutaneous diseases	3
Sense organ diseases	2
Blindness and vision loss	3
Glaucoma	4
Cataract	4
Age-related macular degeneration	4
Refraction disorders	4
Near vision loss	4
Other vision loss	4

Age-related and other hearing loss	3
Other sense organ diseases	3
Musculoskeletal disorders	2
Rheumatoid arthritis	3
Osteoarthritis	3
Osteoarthritis hip	4
Osteoarthritis knee	4
Osteoarthritis hand	4
Osteoarthritis other	4
Low back pain	3
Neck pain	3
Gout	3
Other musculoskeletal disorders	3
Other non-communicable diseases	2
Congenital birth defects	3
Neural tube defects	4
Congenital heart anomalies	4
Orofacial clefts	4
Down syndrome	4
Turner syndrome	4
Klinefelter syndrome	4
Other chromosomal abnormalities	4
Congenital musculoskeletal and limb anomalies	4
Urogenital congenital anomalies	4
Digestive congenital anomalies	4
Other congenital birth defects	4
Urinary diseases and male infertility	3
Urinary tract infections and interstitial nephritis	4
Urolithiasis	4
Benign prostatic hyperplasia	4
Male infertility	4
Other urinary diseases	4
Gynecological diseases	3
Uterine fibroids	4
Polycystic ovarian syndrome	4
Female infertility	4
Endometriosis	4
Genital prolapse	4
Premenstrual syndrome	4
Other gynecological diseases	4
Hemoglobinopathies and hemolytic anemias	3
Thalassemias	4
Thalassemias trait	4
Sickle cell disorders	4
Sickle cell trait	4
G6PD deficiency	4

G6PD trait	4
Other hemoglobinopathies and hemolytic anemias	4
Endocrine, metabolic, blood, and immune disorders	3
Oral disorders	3
Caries of deciduous teeth	4
Caries of permanent teeth	4
Periodontal diseases	4
Edentulism	4
Other oral disorders	4
Sudden infant death syndrome	3
Injuries	1
Transport injuries	2
Road injuries	3
Pedestrian road injuries	4
Cyclist road injuries	4
Motorcyclist road injuries	4
Motor vehicle road injuries	4
Other road injuries	4
Other transport injuries	3
Unintentional injuries	2
Falls	3
Drowning	3
Fire, heat, and hot substances	3
Poisonings	3
Poisoning by carbon monoxide	4
Poisoning by other means	4
Exposure to mechanical forces	3
Unintentional firearm injuries	4
Other exposure to mechanical forces	4
Adverse effects of medical treatment	3
Animal contact	3
Venomous animal contact	4
Non-venomous animal contact	4
Foreign body	3
Pulmonary aspiration and foreign body in airway	4
Foreign body in eyes	4
Foreign body in other body part	4
Environmental heat and cold exposure	3
Exposure to forces of nature	3
Other unintentional injuries	3
Self-harm and interpersonal violence	2
Self-harm	3
Self-harm by firearm	4
Self-harm by other specified means	4
Interpersonal violence	3
Physical violence by firearm	4

Physical violence by sharp object	4
Sexual violence	4
Physical violence by other means	4
Conflict and terrorism	3
Police conflict and executions	3
Other COVID-19 pandemic-related outcomes	1
Total cancers	1
Total burden related to hepatitis B	1
Total burden related to hepatitis C	1
Total burden related to Non-alcoholic fatty liver disease (NAFLD)	1
Total Cancers excluding Non-melanoma skin cancer	1

Appendix Table S2: List of International Classification of Diseases (ICD) codes mapped to the GBD cause list for causes of death

Cause	ICD10	ICD9
Communicable, maternal, neonatal, and nutritional diseases	A00-A00.9, A01.0-A14, A15-A28.9, A32-A39.9, A48.1-A48.2, A48.4-A48.5, A50-A58, A60-A60.9, A63-A63.8, A65-A65.0, A68-A70, A74, A74.8-A75.9, A77-A96.9, A98-A98.8, B00-B06.9, B10-B10.8, B15-B16.2, B17.0, B17.2, B19.1, B20-B27.9, B29.4, B33-B33.1, B33.3-B33.8, B34.2, B47-B48.8, B50-B53.8, B55.0, B56-B57.5, B60-B60.8, B63, B65-B67.9, B69-B72.0, B74.3-B75, B77-B77.9, B83-B83.8, B90-B91, B94.1, B95-B95.5, B97.2, B97.4-B97.6, C58-C58.0, D50.1-D50.8, D51-D52.0, D52.8-D53.9, D70.3, D89.3, E00-E02, E40-E46.9, E51-E61.9, E63-E64.0, E64.2-E64.9, F02.1, F02.4, F07.1, G00.0-G00.8, G03-G03.8, G04-G05.8, G14-G14.6, G21.3, H70-H70.9, I00, I02, I02.9, I98.0-I98.1, J00-J02.8, J03-J03.8, J04-J04.2, J05-J05.1, J06.0-J06.8, J09-J15.8, J16-J16.9, J20-J21.9, J36-J36.0, J91.0, K52.1-K52.3, K67.0-K67.8, K75.3, K76.3, K77.0, K93.0-K93.1, M03.1, M12.1, M49.0-M49.1, M73.0-M73.1, M89.6, N74.1, N96, N98-N98.9, O00-O07.9, O09-O16.9, O20-O26.9, O28-O36.9, O40-O48.1, O60-O77.9, O80-O92.7, O96-O98.6, O98.8-P04.2, P04.5-P05.9, P07-P15.9, P19-P22.9, P23.0-P23.4, P24-P29.9, P35-P37.2, P37.5-P39.9, P50-P61.9, P70-P70.1, P70.3-P72.9, P74-P78.9, P80-P81.9, P83-P84, P90-P92.9, P94-P94.9, P96, P96.3-P96.4, P96.8, R19.7, U04-U04.9, U06-U07.2, U82-U89, Z16-Z16.3	001-001.9, 002.0-029, 032-034.9, 036-037.9, 040, 040.1-041.0, 042-066.9, 070.0-070.2, 071-075.9, 078.3-078.7, 079-079.7, 080-084.9, 085.0, 086-088, 088.8-088.9, 090-101.6, 104-104.9, 120-124.9, 125.4-125.9, 127-127.1, 128-129.0, 136-136.2, 137-139.0, 181-181.9, 244.2, 260-263.9, 265-269.9, 281.0-281.9, 320.0-320.8, 321-323.9, 381-383.9, 390-390.9, 392, 392.9, 425.6, 460-464.4, 464.8-464.9, 465.0-465.8, 466-469, 470.0, 475-475.9, 476.9, 480-482.8, 483.0-483.9, 484.0-484.7, 487-489, 558.2-558.9, 630-636.9, 638-638.9, 640-679.1, 716.0, 730.4-730.6, 760-760.6, 760.8-768, 768.2-770, 770.1-775.0, 775.4-779.3, 779.6-779.8, V09-V09.9
HIV/AIDS and sexually transmitted infections	A50-A58, A60-A60.9, A63-A63.8, B20-B24.9, B63, F02.4, I98.0, K67.0-K67.2, M03.1, M73.0-M73.1	042-044.9, 054.1, 090-099.9
HIV/AIDS	B20-B24.9, F02.4	042-044.9
HIV/AIDS - Drug-susceptible Tuberculosis	B20.0	
HIV/AIDS - Multidrug-resistant Tuberculosis without extensive drug resistance		
HIV/AIDS - Extensively drug-resistant Tuberculosis		
HIV/AIDS resulting in other diseases	B20, B20.1-B24.9, F02.4	042-044.9
Sexually transmitted infections excluding HIV	A50-A58, A60-A60.9, A63-A63.8, B63, I98.0, K67.0-K67.2, M03.1, M73.0-M73.1	054.1, 090-099.9
Syphilis	A50-A53.9, I98.0, K67.2, M03.1, M73.1	090-097.9
Chlamydial infection	A55-A56.8, K67.0	
Gonococcal infection	A54-A54.9, K67.1, M73.0	098-098.9
Other sexually transmitted infections	A57-A58, A63-A63.8, B63	099-099.9
Respiratory infections and tuberculosis	A10-A14, A15-A19.9, A48.1, A70, B34.2, B90-B90.9, B97.2, B97.4-B97.6, H70-H70.9, J00-J02.8, J03-J03.8, J04-J04.2, J05-J05.1, J06.0-J06.8, J09-J15.8, J16-J16.9, J20-J21.9, J36-J36.0, J91.0, K67.3, K93.0, M49.0, N74.1, P23.0-P23.4, P37.0, U04-U04.9, U07-U07.2, U84.3	010-019.9, 034.0, 079.6, 137-137.9, 138.0-138.9, 320.4, 381-383.9, 460-464.4, 464.8-464.9, 465.0-465.8, 466-469, 470.0, 475-475.9, 476.9, 480-482.8, 483.0-483.9, 484.1-484.2, 484.6-484.7, 487-489, 730.4-730.6
Tuberculosis	A10-A14, A15-A19.9, B90-B90.9, K67.3, K93.0, M49.0, N74.1, P37.0, U84.3	010-019.9, 137-137.9, 138.0-138.9, 320.4, 730.4-730.6
Drug-susceptible tuberculosis	A10-A14, A15-A19.9, B90-B90.9, K67.3, K93.0, M49.0, N74.1, P37.0	010-019.9, 137-137.9, 138.0-138.9, 320.4, 730.4-730.6
Multidrug-resistant tuberculosis without extensive drug resistance	U84.3	
Extensively drug-resistant tuberculosis		
Lower respiratory infections	A48.1, A70, B34.2, B97.2, B97.4-B97.6, J09-J15.8, J16-J16.9, J20-J21.9, J91.0, P23.0-P23.4, U04-U04.9	079.6, 466-469, 470.0, 480-482.8, 483.0-483.9, 484.1-484.2, 484.6-484.7, 487-489
Influenza	J09-J11.8, U04-U04.9	487-489
Pneumococcal pneumonia	J13-J13.9, J15.3-J15.4, J15.6	481-481.9
H influenzae type B pneumonia	J14-J14.0	482.2
Respiratory syncytial virus pneumonia	B97.4-B97.6, J12.1	079.6, 480.1
Other lower respiratory infections	A48.1, A70, B34.2, B97.2, J12-J12.0, J12.2-J12.9, J15-J15.2, J15.5, J15.7-J15.8, J16-J16.9, J20-J21.9, J91.0, P23.0-P23.4	466-469, 470.0, 480-480.0, 480.2-480.9, 482-482.1, 482.3-482.8, 483.0-483.9, 484.1-484.2, 484.6-484.7
Upper respiratory infections	J00-J02.8, J03-J03.8, J04-J04.2, J05-J05.1, J06.0-J06.8, J36-J36.0	034.0, 460-464.4, 464.8-464.9, 465.0-465.8, 475-475.9, 476.9
Otitis media	H70-H70.9	381-383.9
COVID-19	U07-U07.2	
Enteric infections	A00-A00.9, A01.0-A09.9, A80-A80.9, K52.1-K52.3, R19.7	001-001.9, 002.0-009.9, 045-045.9, 138, 558.2-558.9
Diarrheal diseases	A00-A00.9, A02-A02.0, A02.8-A07, A07.2-A07.4, A08-A09.9, K52.1-K52.3, R19.7	001-001.9, 003.8-006.9, 007.4-007.8, 008.2-009.9, 558.2-558.9
Leprosy	A30-A30.9	030-030.9
Typhoid and paratyphoid	A01.0-A01.4	002.0-002.9
Typhoid fever	A01.0	002.0
Paratyphoid fever	A01.1-A01.4	002.1-002.9
Invasive Non-typhoidal Salmonella (iNTS)	A02.1-A02.2	003-003.7
Other intestinal infectious diseases	A07.0-A07.1, A07.8-A07.9, A80-A80.9	007-007.3, 007.9-008.1, 045-045.9, 138
Neglected tropical diseases and malaria	A68-A68.9, A69.2-A69.9, A75-A75.9, A77-A79.9, A82-A82.9, A90-A96.9, A98-A98.8, B33.0-B33.1, B50-B53.8, B55.0, B56-B57.5, B60-B60.8, B65-B67.9, B69-B72.0, B74.3-B75, B77-B77.9, B83-B83.8, K93.1, P37.1, U06-U06.9	060-061.8, 065-066.9, 071-071.9, 080-084.9, 085.0, 086-088, 088.8-088.9, 120-124.9, 125.4-125.9, 127-127.1, 128-129.0, 425.6
Malaria	B50-B53.8	084-084.9
Chagas disease	B57-B57.5, K93.1	086-086.2, 086.9, 425.6
Leishmaniasis	B55.0	085.0
Visceral leishmaniasis	B55.0	085.0
African trypanosomiasis	B56-B56.9	086.3-086.5
Schistosomiasis	B65-B65.9	120-120.9
Cysticercosis	B69-B69.9	123.1
Cystic echinococcosis	B67-B67.4, B67.8-B67.9	122-122.4, 122.8-122.9
Dengue	A90-A91.9	061-061.8
Yellow fever	A95-A95.9	060-060.9
Rabies	A82-A82.9	071-071.9
Intestinal nematode infections	B77-B77.9	127.0
Ascariasis	B77-B77.9	127.0

Appendix Table S2: List of International Classification of Diseases (ICD) codes mapped to the GBD cause list for causes of death

Cause	ICD10	ICD9
Ebola	A98.4	
Zika virus	U06-U06.9	
Other neglected tropical diseases	A68-A68.9, A69.2-A69.9, A75-A75.9, A77-A79.9, A92-A94.0, A96-A96.9, A98-A98.3, A98.5-A98.8, B33.0-B33.1, B60-B60.8, B67.5-B67.7, B70-B71.9, B74.3-B75, B83-B83.8, P37.1	065-066.9, 080-083.9, 087-088, 088.8-088.9, 122.5-122.7, 123-123.0, 123.2-124.9, 125.4-125.6, 125.9, 127, 127.1, 128-129.0
Other infectious diseases	A20-A28.9, A32-A39.9, A48.2, A48.4-A48.5, A65-A65.0, A69-A69.1, A74, A74.8-A74.9, A81-A81.9, A83-A89.9, B00-B06.9, B10-B10.8, B15-B16.2, B17.0, B17.2, B19.1, B25-B27.9, B29.4, B33, B33.3-B33.8, B47-B48.8, B91, B94.1, B95-B95.5, D70.3, D89.3, F02.1, F07.1, G00.0-G00.8, G03-G03.8, G04-G05.8, G14-G14.6, G21.3, I00, I02, I02.9, I98.1, K67.8, K75.3, K76.3, K77.0, M49.1, M89.6, P35-P35.9, P37, P37.2, P37.5-P37.9, U82-U84, U85-U89, Z16-Z16.3	020-029, 032-034, 034.1-034.9, 036-037.9, 040, 040.1-041.0, 046-054.0, 054.2-059.9, 062-064.9, 070.0-070.2, 072-075.9, 078.3-078.7, 079-079.5, 079.7, 100-101.6, 104-104.9, 136-136.2, 139-139.0, 320.0-320.3, 320.5-320.8, 321-323.9, 390-390.9, 392, 392.9, 484.0, 484.3-484.5, 771.0-771.3, V09-V09.9
Meningitis	A39-A39.9, A87-A87.9, G00.0-G00.8, G03-G03.8	036-036.9, 047-049.9, 320.0-320.3, 320.5-320.8, 321-322.9
Encephalitis	A83-A86.4, B94.1, F07.1, G04-G05.8, G21.3	062-064.9, 139.0, 323, 323.4-323.9
Diphtheria	A36-A36.9	032-032.9
Pertussis	A37-A37.9	033-033.9, 484.3
Tetanus	A33-A35.0	037-037.9, 771.3
Measles	B05-B05.9	055-055.9, 484.0
Varicella and herpes zoster	B01-B02.9, P35.8	052-053.9
Acute hepatitis	B15-B16.2, B17.0, B17.2, B19.1, P35.3	070.0-070.2
Acute hepatitis A	B15-B15.9	070.0-070.1
Acute hepatitis B	B16-B16.2, B17.0, B19.1, P35.3	070.2
Acute hepatitis C		
Acute hepatitis E	B17.2	
Other unspecified infectious diseases	A20-A28.9, A32-A32.9, A38-A38.9, A48.2, A48.4-A48.5, A65-A65.0, A69-A69.1, A74, A74.8-A74.9, A81-A81.9, A88-A89.9, B00-B00.9, B03-B04, B06-B06.9, B10-B10.8, B25-B27.9, B29.4, B33, B33.3-B33.8, B47-B48.8, B91, B95-B95.5, D70.3, D89.3, F02.1, G14-G14.6, I00, I02, I02.9, I98.1, K67.8, K75.3, K76.3, K77.0, M49.1, M89.6, P35-P35.2, P35.9, P37, P37.2, P37.5-P37.9, U82-U84, U85-U89, Z16-Z16.3	020-029, 034, 034.1-034.9, 040, 040.1-041.0, 046-046.9, 050-051.9, 054-054.0, 054.2-054.9, 056-059.9, 072-075.9, 078.3-078.7, 079-079.5, 079.7, 100-101.6, 104-104.9, 136-136.2, 139, 323.0-323.3, 390-390.9, 392, 392.9, 484.4-484.5, 771.0-771.2, V09-V09.9
Maternal and neonatal disorders	C58-C58.0, N96, N98-N98.9, O00-O07.9, O09-O16.9, O20-O26.9, O28-O36.9, O40-O48.1, O60-O77.9, O80-O92.7, O96-O98.6, O98.8-P04.2, P04.5-P05.9, P07-P15.9, P19-P22.9, P24-P29.9, P36-P36.9, P38-P39.9, P50-P61.9, P70-P70.1, P70.3-P72.9, P74-P78.9, P80-P81.9, P83-P84, P90-P92.9, P94-P94.9, P96, P96.3-P96.4, P96.8	181-181.9, 630-636.9, 638-638.9, 640-679.1, 760-760.6, 760.8-768, 768.2-770, 770.1-771, 771.4-775.0, 775.4-779.3, 779.6-779.8
Maternal disorders	C58-C58.0, N96, N98-N98.9, O00-O07.9, O09-O16.9, O20-O26.9, O28-O36.9, O40-O48.1, O60-O77.9, O80-O92.7, O96-O98.6, O98.8-O99.9	181-181.9, 630-636.9, 638-638.9, 640-679.1
Maternal hemorrhage	O20-O20.9, O43.2, O44-O46.9, O62-O62.9, O67-O67.9, O70, O72-O72.3	640-641.9, 661-661.9, 665, 666-666.9
Maternal sepsis and other maternal infections	O23-O23.9, O85-O86.8, O91-O91.2	659.3, 670-670.9
Maternal hypertensive disorders	O10-O16.9	642-642.9
Maternal obstructed labor and uterine rupture	O32-O33.9, O64-O66.9, O71-O71.9	652-653.9, 660-660.9, 665.0-665.3
Maternal abortion and miscarriage	N96, O01-O07.9	630-632.9, 634-636.9, 638-638.9, 646.3
Ectopic pregnancy	O00-O00.9	633-633.9
Indirect maternal deaths	O24-O25.3, O98-O98.6, O98.8-O99.9	646-646.2, 646.4-649.9
Late maternal deaths	O96-O97.9	
Maternal deaths aggravated by HIV/AIDS		
Other direct maternal disorders	C58-C58.0, N98-N98.9, O09-O09.9, O21-O22.9, O26-O26.9, O28-O31.8, O34-O36.9, O40-O43.1, O43.8-O43.9, O47-O48.1, O60-O61.9, O63-O63.9, O68-O69.9, O70.0-O70.9, O73-O77.9, O80-O84.9, O87-O90.9, O92-O92.7	181-181.9, 643-645.2, 650-651.9, 654-659.2, 659.4-659.9, 662-664.9, 665.4-665.9, 667-669.9, 671-679.1
Neonatal disorders	P00-P04.2, P04.5-P05.9, P07-P15.9, P19-P22.9, P24-P29.9, P36-P36.9, P38-P39.9, P50-P61.9, P70-P70.1, P70.3-P72.9, P74-P78.9, P80-P81.9, P83-P84, P90-P92.9, P94-P94.9, P96, P96.3-P96.4, P96.8	760-760.6, 760.8-768, 768.2-770, 770.1-771, 771.4-775.0, 775.4-779.3, 779.6-779.8
Neonatal preterm birth	P01.0-P01.1, P05-P05.9, P07-P07.3, P22-P22.9, P25-P28.9, P52-P52.9, P61.2, P77-P77.9, P78.0-P78.9	761.0-761.1, 764-765.9, 769-769.9, 770.2-770.9, 772.1-772.9, 776.6, 777.0-777.9
Neonatal encephalopathy due to birth asphyxia and trauma	P01.7, P02-P03.9, P10-P15.9, P20-P21.9, P24-P24.9, P90-P91.9	761.7-763.9, 767-768, 768.2-768.9, 770.1, 779.0-779.2
Neonatal sepsis and other neonatal infections	P36-P36.9, P38-P39.9	771.4-771.9
Hemolytic disease and other neonatal jaundice	P55-P59.9	773-774.9
Other neonatal disorders	P00-P01, P01.2-P01.6, P01.8-P01.9, P04-P04.2, P04.5-P04.9, P08-P09, P19-P19.9, P29-P29.9, P50-P51.9, P53-P54.9, P60-P61.1, P61.3-P61.9, P70-P70.1, P70.3-P72.9, P74-P76.9, P78, P80-P81.9, P83-P84, P92-P92.9, P94-P94.9, P96, P96.3-P96.4, P96.8	760-760.6, 760.8-761, 761.2-761.6, 766-766.9, 770, 771, 772-772.0, 775-775.0, 775.4-776.5, 776.7-777, 778-779, 779.3, 779.6-779.8
Nutritional deficiencies	D50.1-D50.8, D51-D52.0, D52.8-D53.9, E00-E02, E40-E46.9, E51-E61.9, E63-E64.0, E64.2-E64.9, M12.1	244.2, 260-263.9, 265-269.9, 281.0-281.9, 716.0
Protein-energy malnutrition	E40-E46.9, E64.0	260-263.9
Other nutritional deficiencies	D51-D52.0, D52.8-D53.9, E00-E02, E51-E61.9, E63-E64, E64.2-E64.9, M12.1	244.2, 265-269.9, 281.0-281.9, 716.0

Appendix Table S2: List of International Classification of Diseases (ICD) codes mapped to the GBD cause list for causes of death

Cause	ICD10	ICD9
Non-communicable diseases	A46-A46.0, A66-A67.9, B18-B18.9, B33.2, B86, C00-C13.9, C15-C22.8, C23-C25.9, C30-C34.9, C37-C38.8, C40-C41.9, C43-C45.9, C47-C54.9, C56-C57.8, C60-C63.8, C64-C67.9, C68.0-C68.8, C69.0-C69.8, C70-C73.9, C75-C75.8, C81-C82.9, C83.0-C83.8, C84-C85.0, C85.2-C85.8, C86-C86.6, C88-C91.0, C91.2-C91.3, C91.6, C92-C92.6, C93-C93.1, C93.3, C93.8, C94-C94.5, C94.7-C96.9, D00.1-D00.2, D01.0-D01.3, D02.0-D02.3, D03-D06.9, D07.0-D07.2, D07.4-D07.5, D09.0, D09.2-D09.3, D09.8, D10.0-D10.7, D11-D12.9, D13.0-D13.7, D14.0-D14.3, D15-D16.9, D22-D27.9, D28.0-D28.7, D29.0-D29.8, D30.0-D30.8, D31-D36, D36.1-D36.7, D37.1-D37.5, D38.0-D38.5, D39.1-D39.2, D39.8, D40.0-D40.8, D41.0-D41.8, D42-D43.9, D44.0-D44.8, D45-D47.9, D48.0-D48.6, D49.2-D49.4, D49.6, D55-D58.9, D59.1, D59.3, D59.5, D60-D61.9, D63.1, D64.0, D66-D67, D68.0-D69.4, D69.6-D69.8, D70-D70.0, D70.4-D75.8, D76-D77, D86-D86.9, D89-D89.2, E03-E03.1, E03.3-E06.3, E06.5-E07.1, E10-E11.9, E16.1-E16.9, E20-E23.0, E23.2-E24.1, E24.3-E27.2, E27.4-E34, E34.1-E34.8, E65-E66.0, E66.2-E68, E70-E85.2, E88-E88.2, E88.4-E88.9, F00-F02.0, F02.2-F02.3, F02.8-F03.9, F10-F16.9, F18-F18.9, F24, F50.0-F50.5, G10-G13.8, G20-G20.9, G23-G24, G24.1-G25.0, G25.2-G25.3, G25.5, G25.8-G26.0, G30-G31.9, G35-G37.9, G40-G41.9, G45-G46.8, G47.3, G61-G61.9, G62.1, G70-G72, G72.1-G73.7, G90-G90.9, G95-G95.9, H05.0-H05.1, I01-I01.9, I02.0, I05-I09.9, I11-I13.9, I20-I25.9, I27.0-I27.2, I28-I28.9, I30-I31.1, I31.8-I37.8, I38-I41.9, I42.1-I42.8, I43-I43.9, I47-I48.9, I51.0-I51.4, I60-I63.9, I65-I66.9, I67.0-I67.3, I67.5-I67.7, I68.0-I68.2, I69.0-I69.3, I70.2-I70.8, I71-I73.9, I77-I89.9, I98, I98.2, J30-J35.9, J37-J39.9, J41-J46.9, J60-J63.8, J66-J68.9, J70, J70.8-J70.9, J82, J84-J84.9, J91, J91.8-J92.9, K20-K20.9, K22-K22.6, K22.8-K29.9, K31-K31.8, K35-K38.9, K40-K42.9, K44-K46.9, K50-K52, K52.8-K52.9, K55-K62.6, K62.8-K62.9, K63.5, K64-K64.9, K66.8, K67, K68, K70-K70.3, K71.7, K73-K75, K75.1-K75.2, K75.4-K76.2, K76.4-K77, K77.8, K80-K83.9, K85-K86.9, K90-K90.9, K92.8, K93.8, L00-L05.9, L08-L08.9, L10-L14.0, L51-L51.9, L88-L89.9, L93-L93.2, L97-L98.4, M00-M03.0, M03.2-M03.6, M05-M09.8, M30-M36.8, M40-M43.1, M65-M65.0, M71.0-M71.1, M72.5-M72.6, M80-M82.8, M86.3-M86.4, M87-M87.0, M88-M89.0, M89.5, M89.7-M89.9, N00-N08.8, N10-N12.9, N13.6, N15-N16.8, N18-N18.9, N20-N23.0, N25-N28.1, N29-N30.3, N30.8-N32.0, N32.3-N32.4, N34-N34.3, N36-N36.9, N39-N39.2, N41-N41.9, N44-N44.0, N45-N45.9, N49-N49.9, N60-N60.9, N72-N72.0, N75-N77.8, N80-N81.9, N83-N83.9, N84.0-N84.1, N87-N87.9, P04.3-P04.4, P70.2, P96.0-P96.1, Q00-Q07.9, Q10.4-Q18.9, Q20-Q28.9, Q30-Q36, Q37-Q45.9, Q50-Q87.8, Q89-Q89.8, Q90-Q93.9, Q95-Q99.8, R78.0-R78.5, R95-R95.9, X45-X45.9, X65-X65.9, Y15-Y15.9	035-035.9, 102-103.9, 133-133.6, 135-135.9, 140-148.9, 150-155.1, 155.3-158.9, 160-164.9, 170-175.9, 180-180.9, 182-183.8, 184.0-184.4, 184.8, 185-186.9, 187.1-187.8, 188-188.9, 189.0-189.8, 190-190.8, 191-193.9, 194.1-194.8, 200-202.8, 203-204.0, 204.2, 205-205.3, 206-206.1, 207-208.9, 209.0-209.1, 209.4-209.5, 210.0-210.9, 211.0-211.8, 212.0-212.8, 213-213.9, 217-220.9, 221.0-221.8, 222.0-222.8, 223.0-223.8, 224-228.9, 229.0, 229.8, 230.1-230.8, 231.0-231.2, 232-232.9, 233.0-233.2, 233.4-233.5, 233.7, 234.0-234.8, 235.0, 235.4, 235.6-235.8, 236.0-236.2, 236.4-236.5, 236.7, 237-237.3, 237.5-237.9, 238.0-238.9, 239.2-239.4, 239.6, 240-243.9, 245-246.9, 251-251.2, 251.4-253.6, 253.8-259.1, 259.3-259.9, 270-273.9, 275-276, 277-277.2, 277.4-277.9, 278.0-278.8, 282-284.9, 286-286.5, 286.7-289.0, 289.4-289.7, 290-292.9, 294.1-294.9, 303-303.9, 304.0-304.8, 305.0, 305.2-305.8, 307.1, 327.2-327.8, 330-331.2, 331.5-332.0, 333-337.9, 340-341.9, 345-345.9, 349, 349.2-349.8, 353.8-353.9, 356-356.9, 357.0-357.1, 357.3-357.5, 357.7, 358-359.9, 376.0-376.1, 391-391.9, 392.0, 393-398.9, 402-404.9, 410-414.9, 416.0-416.1, 417-417.9, 420-423, 423.1-423.9, 424.0-424.3, 424.8, 425.0-425.5, 425.7-425.8, 427.0-427.3, 427.6-427.8, 429.0, 430-435.9, 437.0-437.2, 437.4-437.8, 440.2, 440.4, 441-443.9, 446-457, 457.1-457.9, 459, 459.1-459.3, 470, 470.9-474.9, 476-476.1, 477-479, 491-493.9, 495-504.9, 506-506.9, 508-509, 515, 516-517.8, 518.6, 518.9, 519.1-519.4, 530-530.0, 530.2-530.6, 531-536.1, 537-537.6, 537.8, 538, 540-543.9, 550-551.1, 551.3-552.1, 552.3-553.1, 553.3-558.0, 560-560.3, 560.8-560.9, 562-562.1, 564-564.1, 564.5-564.7, 565-566.9, 569.0-569.5, 569.7, 571-571.9, 572.2-573.0, 573.4-577.9, 579-579.2, 579.4-583.9, 585-585.9, 588-590.9, 592-593.8, 594-598.1, 598.8-599.6, 599.8, 601-602.9, 604-604.9, 608.2, 610-610.9, 617-618.9, 620-620.9, 621.4-621.9, 622.1-622.7, 629-629.8, 680-689, 694-695.5, 707-707.9, 710-711.9, 714-714.3, 714.8-714.9, 730.1, 732-732.9, 733.0-733.1, 740-749.0, 749.2-758.9, 759.0-759.8, 760.7, 775.1-775.3, 788.0, 790.3, 798-798.0, E850, E860
Neoplasms	C00-C13.9, C15-C22.8, C23-C25.9, C30-C34.9, C37-C38.8, C40-C41.9, C43-C45.9, C47-C54.9, C56-C57.8, C60-C63.8, C64-C67.9, C68.0-C68.8, C69.0-C69.8, C70-C73.9, C75-C75.8, C81-C82.9, C83.0-C83.8, C84-C85.0, C85.2-C85.8, C86-C86.6, C88-C91.0, C91.2-C91.3, C91.6, C92-C92.6, C93-C93.1, C93.3, C93.8, C94-C94.5, C94.7-C96.9, D00.1-D00.2, D01.0-D01.3, D02.0-D02.3, D03-D06.9, D07.0-D07.2, D07.4-D07.5, D09.0, D09.2-D09.3, D09.8, D10.0-D10.7, D11-D12.9, D13.0-D13.7, D14.0-D14.3, D15-D16.9, D22-D24.9, D26.0-D27.9, D28.0-D28.1, D28.7, D29.0-D29.8, D30.0-D30.8, D31-D36, D36.1-D36.7, D37.1-D37.5, D38.0-D38.5, D39.1-D39.2, D39.8, D40.0-D40.8, D41.0-D41.8, D42-D43.9, D44.0-D44.8, D45-D47.9, D48.0-D48.6, D49.2-D49.4, D49.6, K62.0-K62.1, K63.5, N60-N60.9, N84.0-N84.1, N87-N87.9	140-148.9, 150-155.1, 155.3-158.9, 160-164.9, 170-175.9, 180-180.9, 182-183.8, 184.0-184.4, 184.8, 185-186.9, 187.1-187.8, 188-188.9, 189.0-189.8, 190-190.8, 191-193.9, 194.1-194.8, 200-202.8, 203-204.0, 204.2, 205-205.3, 206-206.1, 207-208.9, 209.0-209.1, 209.4-209.5, 210.0-210.9, 211.0-211.8, 212.0-212.8, 213-213.9, 217-217.8, 219.0, 220-220.9, 221.0-221.8, 222.0-222.8, 223.0-223.8, 224-228.9, 229.0, 229.8, 230.1-230.8, 231.0-231.2, 232-232.9, 233.0-233.2, 233.4-233.5, 233.7, 234.0-234.8, 235.0, 235.4, 235.6-235.8, 236.1-236.2, 236.4-236.5, 236.7, 237-237.3, 237.5-237.9, 238.0-238.9, 239.2-239.4, 239.6, 569.0, 610-610.9, 622.1-622.2, 622.7
Lip and oral cavity cancer	C00-C08.9, D10.0-D10.5, D11-D11.9	140-145.9, 210.0-210.6, 235.0
Nasopharynx cancer	C11-C11.9, D10.6	147-147.9, 210.7-210.9
Other pharynx cancer	C09-C10.9, C12-C13.9, D10.7	146-146.9, 148-148.9
Esophageal cancer	C15-C15.9, D00.1, D13.0	150-150.9, 211.0, 230.1
Stomach cancer	C16-C16.9, D00.2, D13.1, D37.1	151-151.9, 211.1, 230.2
Colon and rectum cancer	C18-C21.9, D01.0-D01.3, D12-D12.9, D37.3-D37.5	153-154.9, 209.1, 209.5, 211.3-211.4, 230.3-230.6, 569.0
Liver cancer	C22-C22.8, D13.4	155-155.1, 155.3-155.9, 211.5
Liver cancer due to hepatitis B		
Liver cancer due to hepatitis C		
Liver cancer due to alcohol use		
Liver cancer due to NASH		
Hepatoblastoma	C22.2	
Liver cancer due to other causes		
Gallbladder and biliary tract cancer	C23-C24.9, D13.5	156-156.9
Pancreatic cancer	C25-C25.9, D13.6-D13.7	157-157.9, 211.6-211.7
Larynx cancer	C32-C32.9, D02.0, D14.1, D38.0	161-161.9, 212.1, 231.0, 235.6
Tracheal, bronchus, and lung cancer	C33-C34.9, D02.1-D02.3, D14.2-D14.3, D38.1	162-162.9, 212.2-212.3, 231.1-231.2, 235.7
Malignant skin melanoma	C43-C43.9, D03-D03.9, D22-D23.9, D48.5	172-172.9
Non-melanoma skin cancer	C44-C44.9, D04-D04.9, D49.2	173-173.9, 222.4, 232-232.9, 238.2
Non-melanoma skin cancer (squamous-cell carcinoma)	C44-C44.9, D04-D04.9, D49.2	173-173.9, 222.4, 232-232.9, 238.2
Soft tissue and other extraosseous sarcomas	C49-C49.9	171-171.9
Malignant neoplasm of bone and articular cartilage	C40-C41.9	170-170.9
Breast cancer	C50-C50.9, D05-D05.9, D24-D24.9, D48.6, D49.3	174-175.9, 217-217.8, 233.0, 238.3, 239.3, 610-610.9
Cervical cancer	C53-C53.9, D06-D06.9, D26.0	180-180.9, 219.0, 233.1, 622.1-622.2, 622.7
Uterine cancer	C54-C54.9, D07.0-D07.2, D26.1-D26.9	182-182.9, 233.2
Ovarian cancer	C56-C56.9, D27-D27.9, D39.1	183-183.0, 220-220.9, 236.2
Prostate cancer	C61-C61.9, D07.5, D29.1, D40.0	185-185.9, 222.2, 236.5
Testicular cancer	C62-C62.9, D29.2-D29.8, D40.1-D40.8	186-186.9, 222.0, 222.3, 236.4
Kidney cancer	C64-C65.9, D30.0-D30.1, D41.0-D41.1	189.0-189.1, 189.5-189.6, 223.0-223.1
Bladder cancer	C67-C67.9, D09.0, D30.3, D41.4-D41.8, D49.4	188-188.9, 223.3, 233.7, 236.7, 239.4
Brain and central nervous system cancer	C70-C72.9, C75.1-C75.3	191-192.9, 194.3-194.4
Eye cancer	C69.0-C69.8	190-190.8

Appendix Table S2: List of International Classification of Diseases (ICD) codes mapped to the GBD cause list for causes of death

Cause	ICD10	ICD9
Retinoblastoma	C69.2	190.5
Other eye cancers	C69.0-C69.1, C69.3-C69.8	190-190.4, 190.6-190.8
Neuroblastoma and other peripheral nervous cell tumors	C47-C47.9	
Thyroid cancer	C73-C73.9, D09.3, D09.8, D34-D34.9, D44.0	193-193.9, 226-226.9
Mesothelioma	C45-C45.9	
Hodgkin lymphoma	C81-C81.9	201-201.9
Non-Hodgkin lymphoma	C82-C82.9, C83.0-C83.8, C84-C85.0, C85.2-C85.8, C86-C86.6, C96-C96.9	200-200.9, 202-202.8
Burkitt lymphoma	C83.7	200.2
Other non-Hodgkin lymphoma	C82-C82.9, C83.0-C83.6, C83.8, C84-C85.0, C85.2-C85.8, C86-C86.6, C96-C96.9	200-200.1, 200.3-200.9, 202-202.8
Multiple myeloma	C88-C90.9	203-203.9
Leukemia	C91-C91.0, C91.2-C91.3, C91.6, C92-C92.6, C93-C93.1, C93.3, C93.8, C94-C94.5, C94.7-C95.9	204-204.0, 204.2, 205-205.3, 206-206.1, 207-208.9
Acute lymphoid leukemia	C91.0, C91.2-C91.3, C91.6	204.0, 204.2
Chronic lymphoid leukemia		
Acute myeloid leukemia	C92.0, C92.3-C92.6, C93.0, C94.0, C94.2, C94.4-C94.5	205.0, 205.2-205.3, 206.0, 207.0, 207.2-207.8
Chronic myeloid leukemia	C92.1-C92.2	205.1
Other leukemia	C93.1, C93.3, C93.8, C94.1, C94.3, C94.7-C94.8	206.1, 207.1, 207.9
Other malignant neoplasms	C17-C17.9, C30-C31.9, C37-C38.8, C48-C48.9, C4A, C51-C52.9, C57-C57.8, C60-C60.9, C63-C63.8, C66-C66.9, C68.0-C68.8, C75-C75.0, C75.4-C75.8, D07.4, D09.2, D13.2-D13.3, D14.0, D15-D16.9, D28.0-D28.1, D28.7, D29.0, D30.2, D30.4-D30.8, D31-D31.9, D35-D35.2, D35.5-D36, D36.1-D36.7, D37.2, D38.2-D38.5, D39.2, D39.8, D41.2-D41.3, D44.1-D44.8, D48.0-D48.4	152-152.9, 158-158.9, 160-160.9, 163-164.9, 183.2-183.8, 184.0-184.4, 184.8, 187.1-187.8, 189.2-189.4, 189.8, 194.1, 194.5-194.8, 209.0, 209.4, 211.2, 211.8, 212.0, 212.4-212.8, 213-213.9, 221.0-221.8, 222.1, 222.8, 223.2, 223.8, 224-224.9, 227-228.9, 229.0, 229.8, 230.7-230.8, 233.4-233.5, 234.0-234.8, 235.4, 235.8, 236.1, 238.0-238.1, 239.2
Other neoplasms	D32-D33.9, D35.3-D35.4, D42-D43.9, D45-D47.9, D49.6, K62.0-K62.1, K63.5, N60-N60.9, N84.0-N84.1, N87-N87.9	225-225.9, 237-237.3, 237.5-237.9, 238.4-238.9, 239.6
Myelodysplastic, myeloproliferative, and other hematopoietic neoplasms	D45-D47.9	238.4-238.9
Other benign and in situ neoplasms	N60-N60.9	
Cardiovascular diseases	B33.2, G45-G46.8, I01-I01.9, I02.0, I05-I09.9, I11-I11.9, I20-I25.9, I27.0, I27.2, I28-I28.9, I30-I31.1, I31.8-I37.8, I38-I41.9, I42.1-I42.8, I43-I43.9, I47-I48.9, I51.0-I51.4, I60-I63.9, I65-I66.9, I67.0-I67.3, I67.5-I67.6, I68.0-I68.2, I69.0-I69.3, I70.2-I70.8, I71-I73.9, I77-I83.9, I86-I89.0, I89.9, I98, K75.1	391-391.9, 392.0, 393-398.9, 402-402.9, 410-414.9, 416.0, 417-417.9, 420-423, 423.1-423.9, 424.0-424.3, 424.8, 425.0-425.5, 425.7-425.8, 427.0-427.3, 427.6-427.8, 429.0, 430-435.9, 437.0-437.2, 437.5-437.8, 440.2, 440.4, 441-443.9, 447-454.9, 456, 456.3-457, 457.1, 457.8-457.9, 459, 459.1-459.3
Rheumatic heart disease	I01-I01.9, I02.0, I05-I09.9	391-391.9, 392.0, 393-398.9
Ischemic heart disease	I20-I25.9	410-414.9
Stroke	G45-G46.8, I60-I63.9, I65-I66.9, I67.0-I67.3, I67.5-I67.6, I68.1-I68.2, I69.0-I69.3	430-435.9, 437.0-437.2, 437.5-437.8
Ischemic stroke	G45-G46.8, I63-I63.9, I65-I66.9, I67.2-I67.3, I67.5-I67.6, I69.3	433-435.9, 437.0-437.1, 437.5-437.8
Intracerebral hemorrhage	I61-I62, I62.1-I62.9, I68.1-I68.2, I69.1-I69.2	431-432.9, 437.2
Subarachnoid hemorrhage	I60-I60.9, I62.0, I67.0-I67.1, I69.0	430-430.9
Hypertensive heart disease	I11-I11.9	402-402.9
Non-rheumatic valvular heart disease	I34-I37.8	424.0-424.3, 424.8
Non-rheumatic calcific aortic valve disease	I35-I35.9	424.1
Non-rheumatic degenerative mitral valve disease	I34-I34.9	424.0
Other non-rheumatic valve diseases	I36-I37.8	424.2-424.3, 424.8
Cardiomyopathy and myocarditis	B33.2, I40-I41.9, I42.1-I42.8, I43-I43.9, I51.4	422-422.9, 425.0-425.5, 425.7-425.8, 429.0
Myocarditis	B33.2, I40-I41.9, I51.4	422-422.9
Alcoholic cardiomyopathy	I42.6	425.5
Other cardiomyopathy	I42.1-I42.5, I42.7-I42.8, I43-I43.9	425.0-425.4, 425.7-425.8, 429.0
Pulmonary Arterial Hypertension	I27.0	416.0
Atrial fibrillation and flutter	I48-I48.9	427.3
Aortic aneurysm	I71-I71.9	441-441.9
Lower extremity peripheral arterial disease	I70.2-I70.8, I73-I73.9	440.2, 440.4, 443.0-443.9
Endocarditis	I33-I33.9, I38-I39.9	421-421.9
Other cardiovascular and circulatory diseases	I27.2, I28-I28.9, I30-I31.1, I31.8-I32.8, I47-I47.9, I51.0-I51.3, I68.0, I72-I72.9, I77-I83.9, I86-I89.0, I89.9, I98, K75.1	417-417.9, 420-420.9, 423, 423.1-423.9, 427.0-427.2, 427.6-427.8, 442-443, 447-454.9, 456, 456.3-457, 457.1, 457.8-457.9, 459, 459.1-459.3
Chronic respiratory diseases	D86-D86.2, D86.9, G47.3, J30-J35.9, J37-J39.9, J41-J46.9, J60-J63.8, J66-J68.9, J70, J70.8-J70.9, J82, J84-J84.9, J91, J91.8-J92.9	135-135.9, 327.2-327.8, 470, 470.9-474.9, 476-476.1, 477-479, 491-493.9, 495-504.9, 506-506.9, 508-509, 515, 516-517.8, 518.6, 518.9, 519.1-519.4
Chronic obstructive pulmonary disease	J41-J44.9	491-492.9, 496-499
Pneumoconiosis	J60-J63.8, J92.0	500-504.9
Silicosis	J62-J62.9	502-502.9, 503.0, 503.9
Asbestosis	J61-J61.0, J92.0	501
Coal workers pneumoconiosis	J60-J60.0	500-500.9, 501.0-501.9
Other pneumoconiosis	J63-J63.8	503, 503.1, 504-504.9
Asthma	J45-J46.9	493-493.9
Interstitial lung disease and pulmonary sarcoidosis	D86-D86.2, D86.9, J84-J84.9	135-135.9, 515, 516-516.9
Other chronic respiratory diseases	G47.3, J30-J35.9, J37-J39.9, J66-J68.9, J70, J70.8-J70.9, J82, J91, J91.8-J92, J92.9	327.2-327.8, 470, 470.9-474.9, 476-476.1, 477-479, 495-495.9, 506-506.9, 508-509, 517-517.8, 518.6, 518.9, 519.1-519.4
Digestive diseases	B18-B18.9, I84-I85.9, I98.2, K20-K20.9, K22-K22.6, K22.8-K29.9, K31-K31.8, K35-K38.9, K40-K42.9, K44-K46.9, K50-K52, K52.8-K52.9, K55-K62, K62.2-K62.6, K62.8-K62.9, K64-K64.9, K66.8, K67, K68, K70-K70.3, K71.7, K73-K75, K75.2, K75.4-K76.2, K76.4-K77, K77.8, K80-K83.9, K85-K86.9, K90-K90.9, K92.8, K93.8, M09.1	455-455.9, 456.0-456.2, 530-530.0, 530.2-530.6, 531-536.1, 537-537.6, 537.8, 538, 540-543.9, 550-551.1, 551.3-552.1, 552.3-553.1, 553.3-558.0, 560-560.3, 560.8-560.9, 562-562.1, 564-564.1, 564.5-564.7, 565-566.9, 569.1-569.5, 569.7, 571-571.9, 572.2-573.0, 573.4-577.9, 579-579.2, 579.4-579.9
Cirrhosis and other chronic liver diseases	B18-B18.9, I85-I85.9, I98.2, K70-K70.3, K71.7, K73-K75, K75.2, K75.4-K76.2, K76.4-K76.9, K77.8	456.0-456.2, 571-571.9, 572.2-573.0, 573.4-573.9
Cirrhosis and other chronic liver diseases due to hepatitis B		
Cirrhosis and other chronic liver diseases due to hepatitis C		

Appendix Table S2: List of International Classification of Diseases (ICD) codes mapped to the GBD cause list for causes of death

Cause	ICD10	ICD9
Cirrhosis and other chronic liver diseases due to alcohol use		
Cirrhosis and other chronic liver diseases due to NAFLD		
Cirrhosis and other chronic liver diseases due to other causes		
Upper digestive system diseases	K25-K29.9	531-535.9
Peptic ulcer disease	K25-K28.9	531-534.9
Gastritis and duodenitis	K29-K29.9	535-535.9
Appendicitis	K35-K37.9, K38.3-K38.9	540-542.9
Paralytic ileus and intestinal obstruction	K56-K56.9	560-560.3, 560.8-560.9
Inguinal, femoral, and abdominal hernia	K40-K42.9, K44-K46.9	550-551.1, 551.3-552.1, 552.3-553.1, 553.3-553.9
Inflammatory bowel disease	K50-K52, K52.8-K52.9, M09.1	555-556.9, 558-558.0, 569.5
Ulcerative colitis	K51-K52, K52.8-K52.9	556-556.9, 558.0
Crohn's disease	K50-K50.9, M09.1	555-555.9
Vascular intestinal disorders	K55-K55.9	557-557.9
Gallbladder and biliary diseases	K80-K83.9	574-576.9
Pancreatitis	K85-K86.9	577-577.9, 579.4
Other digestive diseases	I84-I84.9, K20-K20.9, K22-K22.6, K22.8-K24, K31-K31.8, K38-K38.2, K57-K62, K62.2-K62.6, K62.8-K62.9, K64-K64.9, K66.8, K67, K68, K77, K90-K90.9, K92.8, K93.8	455-455.9, 530-530.0, 530.2-530.6, 536-536.1, 537-537.6, 537.8, 538, 543-543.9, 562-562.1, 564-564.1, 564.5-564.7, 565-566.9, 569.1-569.4, 569.7, 579-579.2, 579.8-579.9
Neurological disorders	F00-F02.0, F02.2-F02.3, F02.8-F03.9, G10-G13.8, G20-G20.9, G23-G24, G24.1-G25.0, G25.2-G25.3, G25.5, G25.8-G26.0, G30-G31.1, G31.8-G31.9, G35-G37.9, G40-G41.9, G61-G61.9, G70-G71.1, G71.3-G72, G72.2-G73.7, G90-G90.9, G95-G95.9, M33-M33.9	290-290.9, 294.1-294.9, 330-331.2, 331.5-332.0, 333-337.9, 340-341.9, 345-345.9, 349, 349.2-349.8, 353.8-353.9, 356-356.9, 357.0-357.1, 357.3-357.4, 357.7, 358-359.9, 775.2
Alzheimer's disease and other dementias	F00-F02.0, F02.8-F03.9, G30-G31.1, G31.8-G31.9	290-290.9, 294.1-294.9, 331-331.2
Parkinson's disease	F02.3, G20-G20.9	332-332.0
Idiopathic epilepsy	G40-G41.9	345-345.9
Multiple sclerosis	G35-G35.9	340-340.9
Motor neuron disease	G12.2-G12.9	335-335.2, 335.8-335.9
Other neurological disorders	F02.2, G10-G12.1, G13-G13.8, G23-G24, G24.1-G25.0, G25.2-G25.3, G25.5, G25.8-G26.0, G36-G37.9, G61-G61.9, G70-G71.1, G71.3-G72, G72.2-G73.7, G90-G90.9, G95-G95.9, M33-M33.9	330-330.9, 331.5-331.9, 333-334.9, 335.3, 336-337.9, 341-341.9, 349, 349.2-349.8, 353.8-353.9, 356-356.9, 357.0-357.1, 357.3-357.4, 357.7, 358-359.9, 775.2
Mental disorders	F24, F50.0-F50.5	307.1
Eating disorders	F50.0-F50.5	307.1
Anorexia nervosa	F50.0-F50.5	307.1
Substance use disorders	E24.4, F10-F16.9, F18-F18.9, G31.2, G62.1, G72.1, P04.3-P04.4, P96.1, Q86.0, R78.0-R78.5, X45-X45.9, X65-X65.9, Y15-Y15.9	291-292.9, 303-303.9, 304.0-304.8, 305.0, 305.2-305.8, 357.5, 760.7, 790.3, E850, E860
Alcohol use disorders	E24.4, F10-F10.9, G31.2, G62.1, G72.1, P04.3, Q86.0, R78.0, X45-X45.9, X65-X65.9, Y15-Y15.9	291-291.9, 303-303.9, 305.0, 357.5, 790.3, E860
Drug use disorders	F11-F16.9, F18-F18.9, P04.4, P96.1, R78.1-R78.5	292-292.9, 304.0-304.8, 305.2-305.8, 760.7, E850
Opioid use disorders	F11-F11.9, P96.1, R78.1	304.0, 305.5
Cocaine use disorders	F14-F14.9, R78.2	304.2, 305.6
Amphetamine use disorders	F15-F15.9	304.4, 305.7
Other drug use disorders	F13-F13.9, F16-F16.9, F18-F18.9, P04.4, R78.3-R78.5	292-292.9, 304.1, 304.5-304.8, 305.3-305.4, 305.8, 760.7
Diabetes and kidney diseases	D63.1, E10-E11.9, I12-I13.9, N00-N08.8, N15.0, N18-N18.9, P70.2, Q61-Q62.8	403-404.9, 580-583.9, 585-585.9, 589-589.9, 753-753.3, 775.1
Diabetes mellitus	E10-E10.1, E10.3-E11.1, E11.3-E11.9, P70.2	775.1
Diabetes mellitus type 1	E10-E10.1, E10.3-E10.9, P70.2	775.1
Diabetes mellitus type 2	E11-E11.1, E11.3-E11.9	
Chronic kidney disease	D63.1, E10.2, E11.2, I12-I13.9, N02-N08.8, N15.0, N18-N18.9, Q61-Q62.8	403-404.9, 581-583.9, 585-585.9, 589-589.9, 753-753.3
Chronic kidney disease due to diabetes mellitus type 1	E10.2	
Chronic kidney disease due to diabetes mellitus type 2	E11.2	
Chronic kidney disease due to hypertension	I12-I13.9	403-404.9
Chronic kidney disease due to glomerulonephritis	N03-N06.9	581-583.9
Chronic kidney disease due to other and unspecified causes	N02-N02.9, N07-N08.8, N15.0, Q61-Q62.8	589-589.9, 753-753.3
Acute glomerulonephritis	N00-N01.9	580-580.9
Skin and subcutaneous diseases	A46-A46.0, A66-A67.9, B86, D86.3, H05.0-H05.1, I89.1-I89.8, L00-L05.9, L08-L08.9, L10-L14.0, L51-L51.9, L88-L89.9, L97-L98.4, M72.5-M72.6	035-035.9, 102-103.9, 133-133.6, 376.0-376.1, 457.2-457.3, 680-689, 694-695.3, 707-707.9
Bacterial skin diseases	A46-A46.0, A66-A67.9, H05.0-H05.1, I89.1-I89.8, L00-L05.9, L08-L08.9, L88, L97-L98.4, M72.5-M72.6	035-035.9, 102-103.9, 376.0-376.1, 457.2-457.3, 680-689
Cellulitis	H05.0, L03-L03.9, M72.5-M72.6	681-682.9
Pyoderma	A46-A46.0, A66-A67.9, H05.1, I89.1-I89.8, L00-L02.9, L04-L05.9, L08-L08.9, L88, L97-L98.4	035-035.9, 102-103.9, 376.0-376.1, 457.2-457.3, 680-680.9, 683-689
Decubitus ulcer	L89-L89.9	707-707.9
Other skin and subcutaneous diseases	D86.3, L10-L14.0, L51-L51.9	694-695.3
Musculoskeletal disorders	I27.1, I67.7, L93-L93.2, M00-M03.0, M03.2-M03.6, M05-M09.0, M09.2-M09.8, M30-M32.9, M34-M36.8, M40-M43.1, M65-M65.0, M71.0-M71.1, M80-M82.8, M86.3-M86.4, M87-M87.0, M88-M89.0, M89.5, M89.7-M89.9	416.1, 437.4, 446-446.9, 695.4-695.5, 710-711.9, 714-714.3, 714.8-714.9, 730.1, 732-732.9, 733.0-733.1
Rheumatoid arthritis	M05-M06.9, M08.0-M08.8	714-714.3, 714.8-714.9
Other musculoskeletal disorders	I27.1, I67.7, L93-L93.2, M00-M03.0, M03.2-M03.6, M07-M08, M08.9-M09.0, M09.2-M09.8, M30-M32.9, M34-M36.8, M40-M43.1, M65-M65.0, M71.0-M71.1, M80-M82.8, M86.3-M86.4, M87-M87.0, M88-M89.0, M89.5, M89.7-M89.9	416.1, 437.4, 446-446.9, 695.4-695.5, 710-711.9, 730.1, 732-732.9, 733.0-733.1

Appendix Table S2: List of International Classification of Diseases (ICD) codes mapped to the GBD cause list for causes of death		
**Cause**	**ICD10**	**ICD9**
Other non-communicable diseases	D25-D26, D28.2, D55-D58.9, D59.1, D59.3, D59.5, D60-D61.9, D64.0, D66-D67, D68.0-D69.4, D69.6-D69.8, D70-D70.0, D70.4-D75.8, D76-D77, D86.8, D89-D89.2, E03-E03.1, E03.3-E06.3, E06.5-E07.1, E16.1-E16.9, E20-E23.0, E23.2-E24.1, E24.3, E24.8-E27.2, E27.4-E34, E34.1-E34.8, E65-E66.0, E66.2-E68, E70-E85.2, E88-E88.2, E88.4-E88.9, G71.2, N10-N12.9, N13.6, N15, N15.1-N16.8, N20-N23.0, N25-N28.1, N29-N30.3, N30.8-N32.0, N32.3-N32.4, N34-N34.3, N36-N36.9, N39-N39.2, N41-N41.9, N44-N44.0, N45-N45.9, N49-N49.9, N72-N72.0, N75-N77.8, N80-N81.9, N83-N83.9, P96.0, Q00-Q07.9, Q10.4-Q18.9, Q20-Q28.9, Q30-Q36, Q37-Q45.9, Q50-Q60.6, Q63-Q86, Q86.1-Q87.8, Q89-Q89.8, Q90-Q93.9, Q95-Q99.8, R95-R95.9	218-219, 219.1-219.9, 236.0, 240-243.9, 245-246.9, 251-251.2, 251.4-253.6, 253.8-259.1, 259.3-259.9, 270-273.9, 275-276, 277-277.2, 277.4-277.9, 278.0-278.8, 282-284.9, 286-286.5, 286.7-289.0, 289.4-289.7, 588-588.9, 590-590.9, 592-593.8, 594-598.1, 598.8-599.6, 599.8, 601-602.9, 604-604.9, 608.2, 617-618.9, 620-620.9, 621.4-621.9, 622.3-622.6, 629-629.8, 740-749.0, 749.2-752.9, 753.4-758.9, 759.0-759.8, 775.3, 788.0, 798-798.0
Congenital birth defects	G71.2, P96.0, Q00-Q07.9, Q10.4-Q18.9, Q20-Q28.9, Q30-Q36, Q37-Q45.9, Q50-Q60.6, Q63-Q86, Q86.1-Q87.8, Q89-Q89.8, Q90-Q93.9, Q95-Q99.8	740-749.0, 749.2-752.9, 753.4-758.9, 759.0-759.8
Neural tube defects	Q00-Q01.9, Q05-Q05.9	740-741.9, 742.0
Congenital heart anomalies	Q20-Q28.9	745-747.9
Orofacial clefts	Q35-Q36, Q37-Q37.9	749-749.0, 749.2-749.9
Down syndrome	Q90-Q90.9	758.0
Other chromosomal abnormalities	Q87-Q87.8, Q91-Q93.9, Q95-Q95.9, Q97-Q97.9, Q99-Q99.8	758, 758.1-758.6, 758.8-758.9
Congenital musculoskeletal and limb anomalies	Q65-Q79, Q79.6-Q79.9	742.5, 754-756.5, 756.8-756.9
Urogenital congenital anomalies	P96.0, Q50-Q60.6, Q63-Q64.9	752-752.9, 753.4-753.9
Digestive congenital anomalies	Q38-Q45.9, Q79.0-Q79.5	750-751.9, 756.6-756.7
Other congenital birth defects	G71.2, Q02-Q04.9, Q06-Q07.9, Q10.4-Q18.9, Q30-Q34.9, Q80-Q86, Q86.1-Q86.8, Q89-Q89.8	742, 742.1-742.4, 742.8-744.9, 748-748.9, 757-757.9, 759.0-759.8
Urinary diseases and male infertility	N10-N12.9, N13.6, N15, N15.1-N16.8, N20-N23.0, N25-N28.1, N29-N30.3, N30.8-N32.0, N32.3-N32.4, N34-N34.3, N36-N36.9, N39-N39.2, N41-N41.9, N44-N44.0, N45-N45.9, N49-N49.9	588-588.9, 590-590.9, 592-593.8, 594-598.1, 598.8-599.6, 599.8, 601-602.9, 604-604.9, 608.2, 788.0
Urinary tract infections and interstitial nephritis	N10-N12.9, N13.6, N15, N15.1-N16.8, N30-N30.3, N30.8-N30.9, N34-N34.3, N39.0-N39.2	590-590.9, 595-595.9, 597-597.9, 599.0
Urolithiasis	N20-N23.0	592-592.9, 594-594.9, 788.0
Other urinary diseases	N25-N28.1, N29-N29.8, N31-N32.0, N32.3-N32.4, N36-N36.9, N39, N41-N41.9, N44-N44.0, N45-N45.9, N49-N49.9	588-588.9, 593-593.8, 596-596.9, 598-598.1, 598.8-599, 599.1-599.6, 599.8, 601-602.9, 604-604.9, 608.2
Gynecological diseases	D25-D26, D28.2, E28.2, N72-N72.0, N75-N77.8, N80-N81.9, N83-N83.9	218-219, 219.1-219.9, 236.0, 256.4, 617-618.9, 620-620.9, 621.4-621.9, 622.3-622.6, 629-629.8
Uterine fibroids	D25-D26, D28.2	218-219, 219.1-219.9, 236.0
Endometriosis	N80-N80.9	617-617.9
Genital prolapse	N81-N81.9	618-618.9
Other gynecological diseases	N72-N72.0, N75-N77.8, N83-N83.9	620-620.9, 621.4-621.9, 622.3-622.6, 629-629.8
Hemoglobinopathies and hemolytic anemias	D55-D58.9, D59.1, D59.3, D59.5, D60-D61.9, D64.0	282-284.9
Thalassemias	D56-D56.9	282.4-282.5
Sickle cell disorders	D57-D57.8	282.6
G6PD deficiency	D55-D55.2	282.2-282.3
Other hemoglobinopathies and hemolytic anemias	D55.3-D55.9, D58-D58.9, D59.1, D59.3, D59.5, D60-D61.9, D64.0	282-282.1, 282.7-284.9
Endocrine, metabolic, blood, and immune disorders	D66-D67, D68.0-D69.4, D69.6-D69.8, D70-D70.0, D70.4-D75.8, D76-D77, D86.8, D89-D89.2, E03-E03.1, E03.3-E06.3, E06.5-E07.1, E16.1-E16.9, E20-E23.0, E23.2-E24.1, E24.3, E24.8-E27.2, E27.4-E28.1, E28.3-E34, E34.1-E34.8, E65-E66.0, E66.2-E68, E70-E85.2, E88-E88.2, E88.4-E88.9	240-243.9, 245-246.9, 251-251.2, 251.4-253.6, 253.8-256.3, 256.8-259.1, 259.3-259.9, 270-273.9, 275-276, 277-277.2, 277.4-277.9, 278.0-278.8, 286-286.5, 286.7-289.0, 289.4-289.7, 775.3
Thyroid diseases	E03-E03.1, E03.3-E06.3, E06.5-E07, E07.1	240-243.9, 245-245.9, 775.3
Other endocrine, metabolic, blood, and immune disorders	D66-D67, D68.0-D69.4, D69.6-D69.8, D70-D70.0, D70.4-D75.8, D76-D77, D86.8, D89-D89.2, E07.0, E16.1-E16.9, E20-E23.0, E23.2-E24.1, E24.3, E24.8-E27.2, E27.4-E28.1, E28.3-E34, E34.1-E34.8, E67-E68, E70-E77.9, E79-E83.9, E85-E85.2, E88-E88.2, E88.4-E88.9	246-246.9, 251-251.2, 251.4-253.6, 253.8-256.3, 256.8-259.1, 259.3-259.9, 270-271.9, 273-273.9, 275-276, 277, 277.1-277.2, 277.4-277.9, 278.2-278.8, 286-286.5, 286.7-289.0, 289.4-289.7
Sudden infant death syndrome	R95-R95.9	798-798.0
Injuries	D52.1, D59.0, D59.2, D59.6, D69.5, D70.1-D70.2, D78-D78.8, E03.2, E06.4, E09-E09.9, E16.0, E23.1, E24.2, E27.3, E36-E36.8, E66.1, E88.3, E89-E89.9, G21.0-G21.1, G24.0, G25.1, G25.4, G25.6-G25.7, G72.0, G93.7, G97-G97.9, I95.2-I95.3, I97-I97.9, I98.9, J70.0-J70.5, J95-J95.9, K43-K43.9, K52.0, K62.7, K91-K91.9, K94-K95.8, L55-L55.9, L56.3, L56.8-L56.9, L58-L58.9, M87.1, N14-N14.4, N30.4, N65-N65.1, N99-N99.9, P93-P93.8, P96.2, P96.5, R50.2, U00-U03, V00-V86.9, V87.2-V87.3, V88.2-V88.3, V90-V98.8, W00-W46.2, W49-W62.9, W64-W70.9, W73-W75.9, W77-W81.9, W83-W94.9, W97.9, W99-X06.9, X08-X39.9, X47-X48.9, X50-X54.9, X57-X58.9, X60-X64.9, X66-X83.9, X85-Y08.9, Y35-Y84.9, Y87.0-Y87.1, Y88-Y88.3, Y89.0-Y89.1	244.0-244.1, 244.3-244.8, 251.3, 253.7, 349.0-349.1, 357.6, 457.0, 518.7, 519.0, 536.4, 539-539.9, 551.2, 552.2, 553.2, 558.1, 564.2-564.4, 569.6, 579.3, 598.2, 779.4-779.5, E800-E807, E830-E838, E840-E849, E856-E857, E861-E865, E867-E869, E870-E876, E878-E879, E880-E886, E888-E928, E930-E979, E990-E999
Transport injuries	V00-V86.9, V87.2-V87.3, V88.2-V88.3, V90-V98.8	E800-E807, E830-E838, E840-E849
Road injuries	V01-V04.9, V06-V80.9, V82-V82.9, V87.2-V87.3	
Pedestrian road injuries	V01-V04.9, V06-V09.9	
Cyclist road injuries	V10-V19.9	
Motorcyclist road injuries	V20-V29.9	
Motor vehicle road injuries	V30-V79.9, V87.2-V87.3	
Other road injuries	V80-V80.9, V82-V82.9	
Other transport injuries	V00-V00.8, V05-V05.9, V81-V81.9, V83-V86.9, V88.2-V88.3, V90-V98.8	E800-E807, E830-E838, E840-E849

Appendix Table S2: List of International Classification of Diseases (ICD) codes mapped to the GBD cause list for causes of death

Cause	ICD10	ICD9
Unintentional injuries	D52.1, D59.0, D59.2, D59.6, D69.5, D70.1-D70.2, D78-D78.8, E03.2, E06.4, E09-E09.9, E16.0, E23.1, E24.2, E27.3, E36-E36.8, E66.1, E88.3, E89-E89.9, G21.0-G21.1, G24.0, G25.1, G25.4, G25.6-G25.7, G72.0, G93.7, G97-G97.9, I95.2-I95.3, I97-I97.9, I98.9, J70.0-J70.5, J95-J95.9, K43-K43.9, K52.0, K62.7, K91-K91.9, K94-K95.8, L55-L55.9, L56.3, L56.8-L56.9, L58-L58.9, M87.1, N14-N14.4, N30.4, N65-N65.1, N99-N99.9, P93-P93.8, P96.2, P96.5, R50.2, W00-W46.2, W49-W62.9, W64-W70.9, W73-W75.9, W77-W81.9, W83-W94.9, W97.9, W99-X06.9, X08-X39.9, X47-X48.9, X50-X54.9, X57-X58.9, Y40-Y84.9, Y88-Y88.3	244.0-244.1, 244.3-244.8, 251.3, 253.7, 349.0-349.1, 357.6, 457.0, 518.7, 519.0, 536.4, 539-539.9, 551.2, 552.2, 553.2, 558.1, 564.2-564.4, 569.6, 579.3, 598.2, 779.4-779.5, E856-E857, E861-E865, E867-E869, E870-E876, E878-E879, E880-E886, E888-E928, E930-E949
Falls	W00-W19.9	E880-E886, E888
Drowning	W65-W70.9, W73-W74.9	E910
Fire, heat, and hot substances	X00-X06.9, X08-X19.9	E890-E899, E924
Poisonings	X47-X48.9	E856-E857, E861-E865, E867-E869
Poisoning by carbon monoxide	X47-X47.9	E862, E868-E869
Poisoning by other means	X48-X48.9	E856-E857, E861, E863-E865, E867
Exposure to mechanical forces	W20-W38.9, W40-W43.9, W45.0-W45.2, W46-W46.2, W49-W52	E916-E922
Unintentional firearm injuries	W32-W34.9	E922
Other exposure to mechanical forces	W20-W31.9, W35-W38.9, W40-W43.9, W45.0-W45.2, W46-W46.2, W49-W52	E916-E921
Still Born	P95-P95.9	768.0-768.1
Adverse effects of medical treatment	D52.1, D59.0, D59.2, D59.6, D69.5, D70.1-D70.2, D78-D78.8, E03.2, E06.4, E09-E09.9, E16.0, E23.1, E24.2, E27.3, E36-E36.8, E66.1, E88.3, E89-E89.9, G21.0-G21.1, G24.0, G25.1, G25.4, G25.6-G25.7, G72.0, G93.7, G97-G97.9, I95.2-I95.3, I97-I97.9, I98.9, J70.0-J70.5, J95-J95.9, K43-K43.9, K52.0, K62.7, K91-K91.9, K94-K95.8, M87.1, N14-N14.4, N30.4, N65-N65.1, N99-N99.9, P93-P93.8, P96.2, P96.5, R50.2, Y40-Y84.9, Y88-Y88.3	244.0-244.1, 244.3-244.8, 251.3, 253.7, 349.0-349.1, 357.6, 457.0, 518.7, 519.0, 536.4, 539-539.9, 551.2, 552.2, 553.2, 558.1, 564.2-564.4, 569.6, 579.3, 598.2, 779.4-779.5, E870-E876, E878-E879, E930-E949
Animal contact	W52.0-W62.9, W64-W64.9, X20-X29.9	E905-E906
Venomous animal contact	X20-X29.9	E905
Non-venomous animal contact	W52.0-W62.9, W64-W64.9	E906
Foreign body	W44-W45, W45.3-W45.9, W75-W75.9, W78-W80.9, W83-W84.9	E911-E915
Pulmonary aspiration and foreign body in airway	W75-W75.9, W78-W80.9, W83-W84.9	E911-E913
Foreign body in other body part	W44-W45, W45.3-W45.9	E914-E915
Electrocution	W85-W87.9	E925
Environmental heat and cold exposure	L55-L55.9, L56.3, L56.8-L56.9, L58-L58.9, W88-W94.9, W97.9, W99-W99.9, X30-X32.9, X39-X39.9	E900-E902, E926
Exposure to forces of nature	X33-X38.9	E907-E909
Garbage Code (GBD Level 1)	A40-A41.9, A48.0, A48.3, A49.0-A49.1, A59-A59.9, A71-A71.9, A74.0, B07-B07.9, B30-B30.9, B35-B36.9, B85-B85.4, B87-B88.9, B94.0, D50-D50.0, D50.9, D62-D63.0, D63.8-D64, D64.1-D65.9, D68, D69.9, E15-E16, E50-E50.9, E64.1, E85.3-E87.6, E87.8-E87.9, F06.2-F06.4, F07.2, F09-F09.9, F19-F23.9, F25-F49, F51-F99.0, G06-G08.0, G32-G32.8, G43-G44.2, G44.4-G44.8, G47-G47.2, G47.4-G47.9, G50-G60.9, G62-G62.0, G62.2-G65.2, G80-G83.9, G89-G89.4, G91-G91.2, G91.4-G93, G93.1-G93.2, G93.4-G93.6, G94.0-G94.8, G99-H05, H05.2-H69.9, H71-H99, I26-I26.9, I31.2-I31.4, I46-I46.9, I50.0-I50.4, I76, I95-I95.1, I95.8-I95.9, J69-J69.9, J80-J80.9, J81.0, J85-J85.3, J86-J86.9, J90-J90.0, J93-J93.1, J93.8-J94.9, J96-J96.9, J98.1-J98.3, K00-K19, K30, K65-K66.1, K66.9, K68.1-K68.9, K71-K71.6, K71.8-K72.9, K75.0, L20-L30.9, L40-L50.9, L52-L54.8, L56-L56.2, L56.4-L56.5, L57-L57.9, L59-L68.9, L70-L76.8, L80-L87.9, L90-L92.9, L94-L96, L98.5-L99.8, M04, M10-M12.0, M12.2-M29, M37-M39, M43.2-M49, M49.2-M64, M65.1-M71, M71.2-M72.4, M72.8-M73, M73.8-M79.9, M83-M86.2, M86.5-M86.9, M87.2-M87.9, M89.1-M89.4, M90-M99.9, N17-N17.9, N19-N19.9, N32.1-N32.2, N32.8-N33.8, N35-N35.9, N37-N37.8, N39.3-N39.8, N42-N43.4, N44.1-N44.8, N46-N48.9, N50-N53.9, N61-N64.9, N82-N82.9, N91-N91.5, N95, N95.1-N95.9, N97-N97.9, R02-R02.9, R03.1-R04, R04.1-R04.9, R07.0, R08-R12.0, R14-R19.6, R19.8-R23, R23.1-R30.9, R32-R50.1, R50.8-R57.9, R58.0-R72.9, R74-R78, R78.6-R94.8, R96-R99.9, U05, U08-U81, U89.9-U99, X40-X44.9, X46-X46.9, X49-X49.9, Y10-Y14.9, Y16-Y19.9, Z00-Z15.8, Z17-unsp.	038-038.9, 040.0, 041.1, 076-078.2, 110-111.9, 125-125.3, 126-126.9, 127.2-127.9, 131-132.9, 133.8-134.9, 136.6, 139.1, 139.9, 247-248, 264-264.9, 274-274.9, 276.0-276.9, 277.3, 280-281, 285-285.9, 286.6, 289.1-289.3, 293, 294-294.0, 295-302.9, 305, 305.9-307.0, 307.2-307.4, 307.6-319.9, 324-327.1, 328-329, 338-339.1, 339.3-339.8, 342-344.9, 346-346.9, 350-353.6, 354-355.9, 360-362, 362.1-376, 376.2-380.9, 384-389.9, 415-415.9, 423.0, 424, 424.4-424.5, 424.9, 427.5, 427.9-428.9, 437.3, 458-458.9, 459.0, 507-507.9, 510-513.9, 518.1-518.3, 520-529.9, 536.3, 536.8-536.9, 537.7, 537.9, 564.8-564.9, 567-568.9, 570-570.9, 572-572.1, 573.1-573.3, 584-584.9, 586-587.9, 603-603.9, 605-608.1, 608.3-609, 611-612.1, 615-616.9, 619-619.9, 621-621.3, 622-622.0, 622.8-623.6, 623.8-624.5, 624.8-628.9, 629.9, 690-693.9, 695.8-706.9, 708-709.9, 712-713.8, 715-716, 716.2-721.6, 721.8-730.0, 730.2-730.3, 730.7-731.9, 733, 733.2-734.2, 737-738, 738.2-739.9, 780-782.4, 782.6-784.6, 784.9, 785.4-786, 786.6, 786.8, 787, 787.3-788, 788.3-790.1, 790.4-796.1, 796.3-797.9, 798.1-799.9, E851-E855, E858, E866, E980-E982, V01-V08, V10-uns
Garbage Code (GBD Level 2)	A14.9, A29-A30.9, A45-A45.9, A47-A48, A48.8-A49, A49.3-A49.9, A61-A62, A72-A73, A76, A97, B08-B09, B11-B14, B28-B29, B31-B32.4, B34-B34.1, B34.3-B34.9, B61-B62, B68-B68.9, B73-B74.2, B76-B76.9, B78-B81.8, B84, B92-B94, B94.8-B94.9, B95.6-B97.1, B97.3, B97.7-B99.9, D59, D59.4, D59.8-D59.9, F17-F17.9, G44.3, G91.3, G93.0, G93.3, I10-I10.9, I15-I15.9, I27, I27.8-I27.9, I50, I50.8-I50.9, I67.4, I70-I70.1, I70.9, I74-I75.8, J81, J81.1, K92.0-K92.2, N70-N71.9, N73-N74.0, N74.2-N74.8, R03-R03.0, R04.0, R05-R06.9, R13-R13.9, R23.0, R58, S00-T98.3, W47-W48, W63, W71-W72, W76-W76.9, W82, W95-W97, W98, X07, X55-X56, X59-X59.9, Y20-Y34.9, Y86-Y87, Y87.2, Y89, Y89.9-Y99.9	000-000.9, 030-030.9, 041.2-041.9, 067-069, 078.8-078.9, 079.8-079.9, 089-089.9, 105-109.9, 119, 136.8-136.9, 139.8, 304, 304.9, 305.1, 339.2, 401-401.9, 405-405.9, 416, 416.2-416.9, 440-440.1, 440.3, 440.8-440.9, 444-445.8, 490-490.9, 494-494.9, 514-514.9, 515.0-515.9, 518-518.0, 518.4-518.5, 518.8, 536.2, 578-578.9, 599.7, 613-614.9, 714.4, 716.1, 721.7, 735-736.9, 738.0-738.1, 784.7-784.8, 786.3, 787.0-787.2, 796.2, 800-E80, E83, E839, E85, E859, E87, E877, E88, E887, E929, E983-E985, E988-E989

Appendix Table S2: List of International Classification of Diseases (ICD) codes mapped to the GBD cause list for causes of death		
**Cause**	**ICD10**	**ICD9**
Garbage Code (GBD Level 3)	A01, A31-A31.9, A42-A44.9, A49.2, A64-A64.0, A99-A99.0, B17, B17.1, B17.8-B17.9, B19-B19.0, B19.2-B19.9, B37-B46.9, B49-B49.9, B55, B55.1-B55.9, B58-B59.9, B89, B94.2, C14-C14.9, C22.9, C26-C29, C35-C36, C39-C39.9, C42, C46-C46.9, C55-C55.9, C57.9, C59, C63.9, C68, C68.9, C74-C74.9, C75.9-C80.9, C83, C83.9, C85.1, C85.9, C87, C94.6, C97-D00.0, D01, D01.4-D02, D02.4-D02.9, D07, D07.3, D07.6-D09, D09.1, D09.7, D09.9-D10, D10.9, D13, D13.9-D14, D14.4, D17-D21.9, D28, D28.9-D29, D29.9-D30, D30.9, D36.0, D36.9-D37.0, D37.6-D38, D38.6-D39.0, D39.7, D39.9-D40, D40.9-D41, D41.9, D44, D44.9, D48, D48.7-D49.1, D49.5, D49.7-D49.9, D54, D75.9, D79-D85, D87-D88, D89.8-D99, E07.8-E08.9, E17-E19, E34.0, E34.9-E35.8, E37-E39, E47-E49., E62, E69, E87.7, E90-E998, F04-F06.1, F06.5-F07.0, F07.8-F08, F50, F50.8-F50.9, G09-G09.9, G15-G19, G21, G21.2, G21.4-G22.0, G27-G29, G33-G34, G38-G39., G42, G48-G49, G66-G69, G74-G79, G84-G88, G93.8-G94, G96-G96.9, G98-G98.9, I00.0, I03-I04., I14-I14., I16-I19, I29-I29.9, I44-I45.9, I49-I49.9, I51, I51.6-I59, I90-I94, I96-I96.9, I98.4-I98.8, I99-ID5.9, J02.9, J03.9, J04.3, J06, J06.9, J40-J40.9, J47-J59, J65-J65.0, J71-J79, J81.9, J83, J85.9, J87-J89, J90.9, J93.6, J97-J98.0, J98.4-J99.8, K21-K21.9, K22.7, K31.9-K34, K39, K47-K49, K53-K54, K63-K63.4, K63.8-K63.9, K69, K70.4-K70.9, K78-K79, K84, K87-K89, K92, K92.9-K93, K96-K99, L06-L07, L09, L15-L19, L31-L39, L69, L77-L79, N09, N13-N13.5, N13.7-N13.9, N24, N28.8-N28.9, N38, N39.9-N40.9, N54-N59, N66-N69, N78-N79, N84, N84.2-N86, N88-N90.9, N92-N94.9, N95.0, O08-O08.9, O17-O19, O27, O37-O39, O49-O59, O78-O79, O93-O95.9, P06, P16-P18, P30-P34.2, P40-P49, P62-P69, P73, P79, P82, P85-P89, P96.9-P99.9, Q08-Q10.3, Q19, Q29-Q29., Q36.0-Q36.9, Q46-Q49, Q88, Q89.9, Q94, Q99.9-R01.2, R07, R07.1-R07.9, R31-R31.9	002, 031-031.9, 039-039.9, 070, 070.4-070.9, 085, 085.1-085.9, 088.0-088.7, 112-118.9, 130-130.9, 136.3-136.5, 149-149.9, 155.2, 159-159.9, 165-169, 176-179.9, 183.9-184, 184.5, 184.9, 187, 187.9, 189, 189.9, 190.9, 195-199.9, 202.9, 209, 209.2-209.3, 209.6-210, 211, 211.9-212, 212.9, 214-216.9, 221, 221.9-222, 222.9-223, 223.9, 229, 229.1, 229.9-230.0, 230.9-231, 231.8-231.9, 233, 233.3, 233.6, 233.9-234, 234.9-235, 235.1-235.3, 235.5, 235.9-236, 236.3, 236.6, 236.9, 237.4, 239-239.1, 239.5, 239.7-239.9, 249-249.9, 259.2, 278, 279-279.9, 293.0-293.9, 331.3-331.4, 332.1-332.9, 347-348.9, 349.9, 357, 357.8-357.9, 399-400.0, 406-409.4, 418-419.9, 426-427, 427.4, 429, 429.2-429.9, 459.5-459.9, 464.5, 465, 465.9, 505-505.9, 519, 519.8-519.9, 530.1, 530.7-530.9, 544-549, 559-559.0, 560.4-560.7, 561, 562.2-563, 569, 569.8-569.9, 591-591.9, 593.9, 599.9-600.9, 623.7, 624.6, 637-637.9, 639-639.9, 749.1, 759, 759.9, 779.9, 782.5, 785-785.3, 786.0-786.2, 786.4-786.5, 786.7, 786.9, 788.1-788.2, E986-E987
Garbage Code (GBD Level 4)	B16.9, B54-B54.0, B64, B82-B82.9, B83.9, C69, C69.9, C91.1, C91.4-C91.5, C91.7-C91.9, C92.7-C92.9, C93.2, C93.5-C93.7, C93.9, E12-E14.9, G00, G00.9-G02.8, G03.9, I37.9, I42-I42.0, I42.9, I51.5, I64-I64.9, I67, I67.8-I68, I68.8-I69, I69.4-I69.9, J07-J08, J15.9, J17-J19.6, J22-J29, J64-J64.9, P23, P23.5-P23.9, P37.3-P37.4, R73-R73.9, V87-V87.1, V87.4-V88.1, V88.4-V89.9, V99-V99.0, X84-X84.9, Y09-Y09.9, Y85-Y85.9	070.3, 194-194.0, 194.9, 204.1, 204.5-204.9, 205.8-205.9, 206.2-206.9, 238, 244, 244.9, 250-250.9, 289.8-289.9, 307.5, 320, 320.9, 357.2, 362.0, 425, 425.9, 429.1, 436-437, 437.9-439.6, 482.9-483, 484, 484.8-486.9, 770.0, 790.2, E808-E829
Other unintentional injuries (internal)	W39-W39.9, W77-W77.9, W81-W81.9, X50-X54.9, X57-X58.9	E903-E904, E923, E927-E928
Self-harm and interpersonal violence	U00-U03, X60-X64.9, X66-X83.9, X85-Y08.9, Y35-Y38.9, Y87.0-Y87.1, Y89.0-Y89.1	E950-E979, E990-E999
Self-harm	X60-X64.9, X66-X83.9, Y87.0	E950-E959
Self-harm by firearm	X72-X74.9	E955
Self-harm by other specified means	X60-X64.9, X66-X71.9, X75-X83.9, Y87.0	E950-E954, E956-E959
Interpersonal violence	X85-Y08.9, Y87.1	E960-E969
Physical violence by firearm	X93-X95.9	E965
Physical violence by sharp object	X99-X99.9	E966
Physical violence by other means	X85-X92.9, X96-X98.9, Y00-Y04.9, Y06-Y08.9, Y87.1	E961-E964, E967-E969
Conflict and terrorism	U00-U03, Y36-Y38.9, Y89.1	E979, E990-E999
Police conflict and executions	Y35-Y35.9, Y89.0	E970-E978
None		

Appendix Table S3: GBD location hierarchy with levels

Global	level
Central Europe, eastern Europe, and central Asia	0
	1
Central Asia	2
Armenia	3
Azerbaijan	3
Georgia	3
Kazakhstan	3
Kyrgyzstan	3
Mongolia	3
Tajikistan	3
Turkmenistan	3
Uzbekistan	3
Central Europe	2
Albania	3
Bosnia and Herzegovina	3
Bulgaria	3
Croatia	3
Czechia	3
Hungary	3
Montenegro	3
North Macedonia	3
Poland	3
Romania	3
Serbia	3
Slovakia	3
Slovenia	3
Eastern Europe	2
Belarus	3
Estonia	3
Latvia	3
Lithuania	3
Moldova	3
Russia	3
Ukraine	3
High income	1
Australasia	2
Australia	3
New Zealand	3
High-income Asia Pacific	2
Brunei	3
Japan	3
Aichi	4
Akita	4
Aomori	4

Chiba	4
Ehime	4
Fukui	4
Fukuoka	4
Fukushima	4
Gifu	4
Gunma	4
Hiroshima	4
Hokkaidō	4
Hyōgo	4
Ibaraki	4
Ishikawa	4
Iwate	4
Kagawa	4
Kagoshima	4
Kanagawa	4
Kōchi	4
Kumamoto	4
Kyōto	4
Mie	4
Miyagi	4
Miyazaki	4
Nagano	4
Nagasaki	4
Nara	4
Niigata	4
Ōita	4
Okayama	4
Okinawa	4
Ōsaka	4
Saga	4
Saitama	4
Shiga	4
Shimane	4
Shizuoka	4
Tochigi	4
Tokushima	4
Tōkyō	4
Tottori	4
Toyama	4
Wakayama	4
Yamagata	4
Yamaguchi	4
Yamanashi	4
South Korea	3
Singapore	3

High-income North America	2
Canada	3
Greenland	3
USA	3
Alabama	4
Alaska	4
Arizona	4
Arkansas	4
California	4
Colorado	4
Connecticut	4
Delaware	4
Washington, DC	4
Florida	4
Georgia	4
Hawaii	4
Idaho	4
Illinois	4
Indiana	4
Iowa	4
Kansas	4
Kentucky	4
Louisiana	4
Maine	4
Maryland	4
Massachusetts	4
Michigan	4
Minnesota	4
Mississippi	4
Missouri	4
Montana	4
Nebraska	4
Nevada	4
New Hampshire	4
New Jersey	4
New Mexico	4
New York	4
North Carolina	4
North Dakota	4
Ohio	4
Oklahoma	4
Oregon	4
Pennsylvania	4
Rhode Island	4
South Carolina	4
South Dakota	4

Tennessee	4
Texas	4
Utah	4
Vermont	4
Virginia	4
Washington	4
West Virginia	4
Wisconsin	4
Wyoming	4
Southern Latin America	2
Argentina	3
Chile	3
Uruguay	3
Western Europe	2
Andorra	3
Austria	3
Belgium	3
Cyprus	3
Denmark	3
Finland	3
France	3
Germany	3
Greece	3
Iceland	3
Ireland	3
Israel	3
Italy	3
Abruzzo	4
Basilicata	4
Calabria	4
Campania	4
Emilia-Romagna	4
Friuli-Venezia Giulia	4
Lazio	4
Liguria	4
Lombardia	4
Marche	4
Molise	4
Piemonte	4
Provincia autonoma di Bolzano	4
Provincia autonoma di Trento	4
Puglia	4
Sardegna	4
Sicilia	4
Toscana	4
Umbria	4

Valle d'Aosta	4
Veneto	4
Luxembourg	3
Malta	3
Monaco	3
Netherlands	3
Norway	3
Agder	4
Innlandet	4
Møre og Romsdal	4
Nordland	4
Oslo	4
Rogaland	4
Troms og Finnmark	4
Trøndelag	4
Vestfold og Telemark	4
Vestland	4
Viken	4
Portugal	3
San Marino	3
Spain	3
Sweden	3
Stockholm	4
Sweden except Stockholm	4
Switzerland	3
UK	3
England	4
East Midlands	5
Derby	6
Derbyshire	6
Leicester	6
Leicestershire	6
Lincolnshire	6
Northamptonshire	6
Nottingham	6
Nottinghamshire	6
Rutland	6
East of England	5
Bedford	6
Cambridgeshire	6
Central Bedfordshire	6
Essex	6
Hertfordshire	6
Luton	6
Norfolk	6
Peterborough	6

Southend-on-Sea	6
Suffolk	6
Thurrock	6
Greater London	5
Barking and Dagenham	6
Barnet	6
Bexley	6
Brent	6
Bromley	6
Camden	6
Croydon	6
Ealing	6
Enfield	6
Greenwich	6
Hackney	6
Hammersmith and Fulham	6
Haringey	6
Harrow	6
Havering	6
Hillingdon	6
Hounslow	6
Islington	6
Kensington and Chelsea	6
Kingston upon Thames	6
Lambeth	6
Lewisham	6
Merton	6
Newham	6
Redbridge	6
Richmond upon Thames	6
Southwark	6
Sutton	6
Tower Hamlets	6
Waltham Forest	6
Wandsworth	6
Westminster	6
North East England	5
County Durham	6
Darlington	6
Gateshead	6
Hartlepool	6
Middlesbrough	6
Newcastle upon Tyne	6
North Tyneside	6
Northumberland	6
Redcar and Cleveland	6

South Tyneside	6
Stockton-on-Tees	6
Sunderland	6
North West England	5
Blackburn with Darwen	6
Blackpool	6
Bolton	6
Bury	6
Cheshire East	6
Cheshire West and Chester	6
Cumbria	6
Halton	6
Knowsley	6
Lancashire	6
Liverpool	6
Manchester	6
Oldham	6
Rochdale	6
Salford	6
Sefton	6
St Helens	6
Stockport	6
Tameside	6
Trafford	6
Warrington	6
Wigan	6
Wirral	6
South East England	5
Bracknell Forest	6
Brighton and Hove	6
Buckinghamshire	6
East Sussex	6
Hampshire	6
Isle of Wight	6
Kent	6
Medway	6
Milton Keynes	6
Oxfordshire	6
Portsmouth	6
Reading	6
Slough	6
Southampton	6
Surrey	6
West Berkshire	6
West Sussex	6
Windsor and Maidenhead	6

Wokingham	6
South West England	5
Bath and North East Somerset	6
Bournemouth	6
Bristol, City of	6
Cornwall	6
Devon	6
Dorset	6
Gloucestershire	6
North Somerset	6
Plymouth	6
Poole	6
Somerset	6
South Gloucestershire	6
Swindon	6
Torbay	6
Wiltshire	6
West Midlands	5
Birmingham	6
Coventry	6
Dudley	6
Herefordshire, County of	6
Sandwell	6
Shropshire	6
Solihull	6
Staffordshire	6
Stoke-on-Trent	6
Telford and Wrekin	6
Walsall	6
Warwickshire	6
Wolverhampton	6
Worcestershire	6
Yorkshire and the Humber	5
Barnsley	6
Bradford	6
Calderdale	6
Doncaster	6
East Riding of Yorkshire	6
Kingston upon Hull, City of	6
Kirklees	6
Leeds	6
North East Lincolnshire	6
North Lincolnshire	6
North Yorkshire	6
Rotherham	6
Sheffield	6

Wakefield	6
York	6
Northern Ireland	4
Scotland	4
Wales	4
Latin America and Caribbean	1
Andean Latin America	2
Bolivia	3
Ecuador	3
Peru	3
Caribbean	2
Antigua and Barbuda	3
The Bahamas	3
Barbados	3
Belize	3
Bermuda	3
Cuba	3
Dominica	3
Dominican Republic	3
Grenada	3
Guyana	3
Haiti	3
Jamaica	3
Puerto Rico	3
Saint Kitts and Nevis	3
Saint Lucia	3
Saint Vincent and the Grenadines	3
Suriname	3
Trinidad and Tobago	3
Virgin Islands	3
Central Latin America	2
Colombia	3
Costa Rica	3
El Salvador	3
Guatemala	3
Honduras	3
Mexico	3
Aguascalientes	4
Baja California	4
Baja California Sur	4
Campeche	4
Chiapas	4
Chihuahua	4
Coahuila	4
Colima	4
Durango	4

Guanajuato	4
Guerrero	4
Hidalgo	4
Jalisco	4
México	4
Mexico City	4
Michoacán de Ocampo	4
Morelos	4
Nayarit	4
Nuevo León	4
Oaxaca	4
Puebla	4
Querétaro	4
Quintana Roo	4
San Luis Potosí	4
Sinaloa	4
Sonora	4
Tabasco	4
Tamaulipas	4
Tlaxcala	4
Veracruz de Ignacio de la Llave	4
Yucatán	4
Zacatecas	4
Nicaragua	3
Panama	3
Venezuela	3
Tropical Latin America	2
Brazil	3
Acre	4
Alagoas	4
Amapá	4
Amazonas	4
Bahia	4
Ceará	4
Distrito Federal	4
Espírito Santo	4
Goiás	4
Maranhão	4
Mato Grosso	4
Mato Grosso do Sul	4
Minas Gerais	4
Pará	4
Paraíba	4
Paraná	4
Pernambuco	4
Piauí	4

Rio de Janeiro	4
Rio Grande do Norte	4
Rio Grande do Sul	4
Rondônia	4
Roraima	4
Santa Catarina	4
São Paulo	4
Sergipe	4
Tocantins	4
Paraguay	3
North Africa and Middle East	1
North Africa and Middle East	2
Afghanistan	3
Algeria	3
Bahrain	3
Egypt	3
Iran	3
Alborz	4
Ardebil	4
Bushehr	4
Chahar Mahaal and Bakhtiari	4
East Azarbayejan	4
Fars	4
Gilan	4
Golestan	4
Hamadan	4
Hormozgan	4
Ilam	4
Isfahan	4
Kerman	4
Kermanshah	4
Khorasan-e-Razavi	4
Khuzestan	4
Kohgiluyeh and Boyer-Ahmad	4
Kurdistan	4
Lorestan	4
Markazi	4
Mazandaran	4
North Khorasan	4
Qazvin	4
Qom	4
Semnan	4
Sistan and Baluchistan	4
South Khorasan	4
Tehran	4
West Azarbayejan	4

Yazd	4
Zanjan	4
Iraq	3
Jordan	3
Kuwait	3
Lebanon	3
Libya	3
Morocco	3
Oman	3
Palestine	3
Qatar	3
Saudi Arabia	3
Sudan	3
Syria	3
Tunisia	3
Türkiye	3
United Arab Emirates	3
Yemen	3
South Asia	1
South Asia	2
Bangladesh	3
Bhutan	3
India	3
Nepal	3
Pakistan	3
Azad Jammu & Kashmir	4
Balochistan	4
Gilgit-Baltistan	4
Islamabad Capital Territory	4
Khyber Pakhtunkhwa	4
Punjab	4
Sindh	4
Southeast Asia, east Asia, and Oceania	1
East Asia	2
China	3
North Korea	3
Taiwan (province of China)	3
Oceania	2
American Samoa	3
Cook Islands	3
Fiji	3
Guam	3
Kiribati	3
Marshall Islands	3
Federated States of Micronesia	3
Nauru	3

Niue	3
Northern Mariana Islands	3
Palau	3
Papua New Guinea	3
Samoa	3
Solomon Islands	3
Tokelau	3
Tonga	3
Tuvalu	3
Vanuatu	3
Southeast Asia	2
Cambodia	3
Indonesia	3
Aceh	4
Bali	4
Bangka-Belitung Islands	4
Banten	4
Bengkulu	4
Gorontalo	4
Jakarta	4
Jambi	4
West Java	4
Central Java	4
East Java	4
West Kalimantan	4
South Kalimantan	4
Central Kalimantan	4
East Kalimantan	4
North Kalimantan	4
Riau Islands	4
Lampung	4
Maluku	4
North Maluku	4
West Nusa Tenggara	4
East Nusa Tenggara	4
Papua	4
West Papua	4
Riau	4
West Sulawesi	4
South Sulawesi	4
Central Sulawesi	4
Southeast Sulawesi	4
North Sulawesi	4
West Sumatra	4
South Sumatra	4
North Sumatra	4

Yogyakarta	4
Laos	3
Malaysia	3
Maldives	3
Mauritius	3
Myanmar	3
Philippines	3
Abra	4
Agusan Del Norte	4
Agusan Del Sur	4
Aklan	4
Albay	4
Antique	4
Apayao	4
Aurora	4
Basilan	4
Bataan	4
Batanes	4
Batangas	4
Benguet	4
Biliran	4
Bohol	4
Bukidnon	4
Bulacan	4
Cagayan	4
Camarines Norte	4
Camarines Sur	4
Camiguin	4
Capiz	4
Catanduanes	4
Cavite	4
Cebu	4
Cotabato (North Cotabato)	4
Davao de Oro	4
Davao Del Norte	4
Davao Del Sur	4
Davao Occidental	4
Davao Oriental	4
Dinagat Islands	4
Eastern Samar	4
Guimaras	4
Ifugao	4
Ilocos Norte	4
Ilocos Sur	4
Iloilo	4
Isabela	4

Kalinga	4
La Union	4
Laguna	4
Lanao Del Norte	4
Lanao Del Sur	4
Leyte	4
Maguindanao	4
Marinduque	4
Masbate	4
Misamis Occidental	4
Misamis Oriental	4
Mountain Province	4
National Capital Region	4
Negros Occidental	4
Negros Oriental	4
Northern Samar	4
Nueva Ecija	4
Nueva Vizcaya	4
Occidental Mindoro	4
Oriental Mindoro	4
Palawan	4
Pampanga	4
Pangasinan	4
Quezon	4
Quirino	4
Rizal	4
Romblon	4
Samar (Western Samar)	4
Sarangani	4
Siquijor	4
Sorsogon	4
South Cotabato	4
Southern Leyte	4
Sultan Kudarat	4
Sulu	4
Surigao Del Norte	4
Surigao Del Sur	4
Tarlac	4
Tawi-Tawi	4
Zambales	4
Zamboanga Del Norte	4
Zamboanga Del Sur	4
Zamboanga Sibugay	4
Seychelles	3
Sri Lanka	3
Thailand	3

Timor-Leste	3
Viet Nam	3
Sub-Saharan Africa	1
Central sub-Saharan Africa	2
Angola	3
Central African Republic	3
Congo (Brazzaville)	3
DR Congo	3
Equatorial Guinea	3
Gabon	3
Eastern sub-Saharan Africa	2
Burundi	3
Comoros	3
Djibouti	3
Eritrea	3
Ethiopia	3
Addis Ababa	4
Afar	4
Amhara	4
Benishangul-Gumuz	4
Dire Dawa	4
Gambella	4
Harari	4
Oromia	4
Somali	4
Southern Nations, Nationalities, and Peoples	4
Tigray	4
Kenya	3
Baringo	4
Bomet	4
Bungoma	4
Busia	4
Elgeyo Marakwet	4
Embu	4
Garissa	4
Homa Bay	4
Isiolo	4
Kajiado	4
Kakamega	4
Kericho	4
Kiambu	4
Kilifi	4
Kirinyaga	4
Kisii	4
Kisumu	4
Kitui	4

Kwale	4
Laikipia	4
Lamu	4
Machakos	4
Makueni	4
Mandera	4
Marsabit	4
Meru	4
Migori	4
Mombasa	4
Murang'a	4
Nairobi	4
Nakuru	4
Nandi	4
Narok	4
Nyamira	4
Nyandarua	4
Nyeri	4
Samburu	4
Siaya	4
Taita Taveta	4
Tana River	4
Tharaka Nithi	4
Trans Nzoia	4
Turkana	4
Uasin Gishu	4
Vihiga	4
Wajir	4
West Pokot	4
Madagascar	3
Malawi	3
Mozambique	3
Rwanda	3
Somalia	3
South Sudan	3
Uganda	3
Tanzania	3
Zambia	3
Southern sub-Saharan Africa	2
Botswana	3
Eswatini	3
Lesotho	3
Namibia	3
South Africa	3
Eastern Cape	4
Free State	4

Gauteng	4
KwaZulu-Natal	4
Limpopo	4
Mpumalanga	4
North West	4
Northern Cape	4
Western Cape	4
Zimbabwe	3
Western sub-Saharan Africa	2
Benin	3
Burkina Faso	3
Cabo Verde	3
Cameroon	3
Chad	3
Côte d'Ivoire	3
The Gambia	3
Ghana	3
Guinea	3
Guinea-Bissau	3
Liberia	3
Mali	3
Mauritania	3
Niger	3
Nigeria	3
São Tomé and Príncipe	3
Senegal	3
Sierra Leone	3
Togo	3

Table S4. List of infectious syndromes mapped to International Classification of Diseases (ICD) codes

Cause Name	ICD 10 Codes	ICD 9 Codes
Acute glomerulonephritis	N00, N00.0, N00.1, N00.2, N00.3, N00.4, N00.5, N00.6, N00.7, N00.8, N00.9, N01, N01.0, N01.1, N01.2, N01.3, N01.4, N01.5, N01.6, N01.7, N01.8, N01.9	580, 580.0, 580.4, 580.8, 580.81, 580.89, 580.9
Acute hepatitis A	B15, B15.0, B15.9	070.0, 070.1
Acute hepatitis B	B16, B16.0, B16.1, B16.2, B17.0, B19.1, B19.10, B19.11, P35.3	070.2, 070.20, 070.21, 070.42, 070.52
Acute hepatitis C		
Acute hepatitis E	B17.2	070.43, 070.53
Adverse effects of medical treatment	N30.4, N30.40, N30.41, P93, P93.0, P93.8, Y40, Y40.0, Y40.1, Y40.2, Y40.3, Y40.4, Y40.5, Y40.6, Y40.7, Y40.8, Y40.9, Y41, Y41.0, Y41.1, Y41.2, Y41.3, Y41.4, Y41.5, Y41.8, Y41.9, Y42, Y42.0, Y42.1, Y42.2, Y42.3, Y42.4, Y42.5, Y42.6, Y42.7, Y42.8, Y42.9, Y43, Y43.0, Y43.1, Y43.2, Y43.3, Y43.4, Y43.5, Y43.6, Y43.8, Y43.9, Y44, Y44.0, Y44.1, Y44.2, Y44.3, Y44.4, Y44.5, Y44.6, Y44.7, Y44.9, Y45, Y45.0, Y45.1, Y45.2, Y45.3, Y45.4, Y45.5, Y45.8, Y45.9, Y46, Y46.0, Y46.1, Y46.2, Y46.3, Y46.4, Y46.5, Y46.6, Y46.7, Y46.8, Y47, Y47.0, Y47.1, Y47.2, Y47.3, Y47.4, Y47.5, Y47.8, Y47.9, Y48, Y48.0, Y48.1, Y48.2, Y48.3, Y48.4, Y48.5, Y49, Y49.0, Y49.1, Y49.2, Y49.3, Y49.4, Y49.5, Y49.6, Y49.7, Y49.8, Y49.9, Y50, Y50.0, Y50.1, Y50.2, Y50.8, Y50.9, Y51, Y51.0, Y51.1, Y51.2, Y51.3, Y51.4, Y51.5, Y51.6, Y51.7, Y51.8, Y51.9, Y52, Y52.0, Y52.1, Y52.2, Y52.3, Y52.4, Y52.5, Y52.6, Y52.7, Y52.8, Y52.9, Y53, Y53.0, Y53.1, Y53.2, Y53.3, Y53.4, Y53.5, Y53.6, Y53.7, Y53.8, Y53.9, Y54, Y54.0, Y54.1, Y54.2, Y54.3, Y54.4, Y54.5, Y54.6, Y54.7, Y54.8, Y54.9, Y55, Y55.0, Y55.1, Y55.2, Y55.3, Y55.4, Y55.5, Y55.6, Y55.7, Y56, Y56.0, Y56.1, Y56.2, Y56.3, Y56.4, Y56.5, Y56.6, Y56.7, Y56.8, Y56.9, Y57, Y57.0, Y57.1, Y57.2, Y57.3, Y57.4, Y57.5, Y57.6, Y57.7, Y57.8, Y57.9, Y58, Y58.0, Y58.1, Y58.2, Y58.3, Y58.4, Y58.5, Y58.6, Y58.8, Y58.9, Y59, Y59.0, Y59.1, Y59.2, Y59.3, Y59.8, Y59.9, Y60, Y60.0, Y60.1, Y60.2, Y60.3, Y60.4, Y60.5, Y60.6, Y60.7, Y60.8, Y60.9, Y61, Y61.0, Y61.1, Y61.2, Y61.3, Y61.4, Y61.5, Y61.6, Y61.7, Y61.8, Y61.9, Y62, Y62.0, Y62.1, Y62.2, Y62.3, Y62.4, Y62.5, Y62.6, Y62.8, Y62.9, Y63, Y63.0, Y63.1, Y63.2, Y63.3, Y63.4, Y63.5, Y63.6, Y63.8, Y63.9, Y64, Y64.0, Y64.1, Y64.8, Y64.9, Y65, Y65.0, Y65.1, Y65.2, Y65.3, Y65.4, Y65.5, Y65.51, Y65.52, Y65.53, Y65.8, Y66, Y69, Y69.2, Y69.9, Y70, Y70.0, Y70.1, Y70.2, Y70.3, Y70.8, Y71, Y71.0, Y71.1, Y71.2, Y71.3, Y71.8, Y72, Y72.0, Y72.1, Y72.2, Y72.3, Y72.8, Y73, Y73.0, Y73.1, Y73.2, Y73.3, Y73.8, Y74, Y74.0, Y74.1, Y74.2, Y74.3, Y74.8, Y75, Y75.0, Y75.1, Y75.2, Y75.3, Y75.8, Y76, Y76.0, Y76.1, Y76.2, Y76.3, Y76.8, Y76.9, Y77, Y77.0, Y77.1, Y77.2, Y77.3, Y77.8, Y78, Y78.0, Y78.1, Y78.2, Y78.3, Y78.8, Y79, Y79.0, Y79.1, Y79.2, Y79.3, Y79.8, Y80, Y80.0, Y80.1, Y80.2, Y80.3, Y80.8, Y81, Y81.0, Y81.1, Y81.2, Y81.3, Y81.8, Y82, Y82.0, Y82.1, Y82.2, Y82.3, Y82.8, Y82.9, Y83, Y83.0, Y83.1, Y83.2, Y83.3, Y83.4, Y83.5, Y83.6, Y83.8, Y83.9, Y84, Y84.0, Y84.1, Y84.2	331.81, 349.0, 349.1, 349.31, 359.24, 457.0, 595.82, E870, E870.0, E870.1, E870.2, E870.3, E870.4, E870.5, E870.6, E870.7, E870.8, E870.9, E871, E871.0, E871.1, E871.2, E871.3, E871.4, E871.5, E871.6, E871.7, E871.8, E871.9, E872, E872.0, E872.1, E872.2, E872.3, E872.4, E872.5, E872.6, E872.8, E872.9, E873, E873.0, E873.1, E873.2, E873.3, E873.4, E873.5, E873.6, E873.8, E873.9, E874, E874.0, E874.1, E874.2, E874.3, E874.4, E874.5, E874.8, E874.9, E875, E875.0, E875.1, E875.2, E875.8, E875.9, E876, E876.0, E876.1, E876.2, E876.3, E876.4, E876.5, E876.6, E876.7, E876.8, E876.9, E878, E878.0, E878.1, E878.2, E878.3, E878.4, E878.5, E878.6, E878.8, E878.9, E879, E879.0, E879.1, E879.2, E879.3, E879.4, E879.5, E879.6, E879.7, E879.8, E879.9, E930, E930.0, E930.1, E930.2, E930.3, E930.4, E930.5, E930.6, E930.7, E930.8, E930.9, E931, E931.0, E931.1, E931.2, E931.3, E931.4, E931.5, E931.6, E931.7, E931.8, E931.9, E932, E932.0, E932.1, E932.2, E932.3, E932.4, E932.5, E932.6, E932.7, E932.8, E932.9, E933, E933.0, E933.1, E933.2, E933.3, E933.4, E933.5, E933.6, E933.7, E933.8, E933.9, E934, E934.0, E934.1, E934.2, E934.3, E934.4, E934.5, E934.6, E934.7, E934.8, E934.9, E935, E935.0, E935.1, E935.2, E935.3, E935.4, E935.5, E935.6, E935.7, E935.8, E935.9, E936, E936.0, E936.1, E936.2, E936.3, E936.4, E937, E937.0, E937.1, E937.2, E937.3, E937.4, E937.5, E937.6, E937.8, E937.9, E938, E938.0, E938.1, E938.2, E938.3, E938.4, E938.5, E938.6, E938.7, E938.9, E939, E939.0, E939.1, E939.2, E939.3, E939.4, E939.5, E939.6, E939.7, E939.8, E939.9, E940, E940.0, E940.1, E940.8, E940.9, E941, E941.0, E941.1, E941.2, E941.3, E941.9, E942, E942.0, E942.1, E942.2, E942.3, E942.4, E942.5, E942.6, E942.7, E942.8, E942.9, E943, E943.0, E943.1, E943.2, E943.3, E943.4, E943.5, E943.6, E943.8, E943.9, E944, E944.0, E944.1, E944.2, E944.3, E944.4, E944.5, E944.6, E944.7, E945, E945.0, E945.1, E945.2, E945.3, E945.4, E945.5, E945.6, E945.7, E945.8, E946, E946.0, E946.1, E946.2, E946.3, E946.4, E946.5, E946.6, E946.7, E946.8, E946.9, E947, E947.0, E947.1, E947.2, E947.3, E947.4, E947.8, E947.9, E948, E948.0, E948.1, E948.2, E948.3, E948.4, E948.5, E948.6, E948.8, E948.9, E949, E949.0, E949.1, E949.2, E949.3, E949.4, E949.5, E949.6, E949.7, E949.9
African trypanosomiasis	B56, B56.0, B56.1, B56.9	086.3, 086.4, 086.5
Alcohol use disorders	E24.4, F10, F10.0, F10.1, F10.10, F10.12, F10.120, F10.121, F10.129, F10.14, F10.15, F10.150, F10.151, F10.159, F10.18, F10.180, F10.181, F10.182, F10.188, F10.19, F10.2, F10.20, F10.21, F10.22, F10.220, F10.221, F10.229, F10.23, F10.230, F10.231, F10.232, F10.239, F10.24, F10.25, F10.250, F10.251, F10.259, F10.26, F10.27, F10.28, F10.280, F10.281, F10.282, F10.288, F10.29, F10.3, F10.4, F10.5, F10.6, F10.7, F10.8, F10.9, F10.92, F10.920, F10.921, F10.929, F10.94, F10.95, F10.950, F10.951, F10.959, F10.96, F10.97, F10.98, F10.980, F10.981, F10.982, F10.988, F10.99, G31.2, G62.1, G72.1, P04.3, Q86.0, R78.0, X45, X45.0, X45.1, X45.2, X45.3, X45.4, X45.5, X45.6, X45.7, X45.8, X45.9, X65, X65.0, X65.1, X65.2, X65.3, X65.4, X65.5, X65.6, X65.7, X65.8, X65.9, Y15, Y15.0, Y15.1, Y15.2, Y15.3, Y15.4, Y15.5, Y15.6, Y15.7, Y15.8, Y15.9	291, 291.0, 291.1, 291.2, 291.3, 291.4, 291.5, 291.8, 291.81, 291.82, 291.89, 291.9, 303, 303.0, 303.00, 303.01, 303.02, 303.03, 303.1, 303.2, 303.9, 303.90, 303.91, 303.92, 303.93, 305.0, 305.00, 305.01, 305.02, 305.03, 357.5, 790.3, E860, E860.0, E860.00, E860.01, E860.02, E860.03, E860.04, E860.05, E860.06, E860.07, E860.08, E860.09, E860.1, E860.10, E860.11, E860.12, E860.13, E860.14, E860.15, E860.16, E860.17, E860.18, E860.19
Alcoholic cardiomyopathy	I42.6	425.5
Amphetamine use disorders	F15, F15.0, F15.1, F15.10, F15.12, F15.120, F15.121, F15.122, F15.129, F15.14, F15.15, F15.150, F15.151, F15.159, F15.18, F15.180, F15.181, F15.182, F15.188, F15.19, F15.2, F15.20, F15.21, F15.22, F15.220, F15.221, F15.222, F15.229, F15.23, F15.24, F15.25, F15.250, F15.251, F15.259, F15.28, F15.280, F15.281, F15.282, F15.288, F15.29, F15.3, F15.4, F15.5, F15.6, F15.7, F15.8, F15.9, F15.90, F15.92, F15.920, F15.921, F15.922, F15.929, F15.93, F15.94, F15.95, F15.950, F15.951, F15.959, F15.98, F15.980, F15.981, F15.982, F15.988, F15.99	304.4, 304.40, 304.41, 304.42, 304.43, 305.7, 305.70, 305.71, 305.72, 305.73
Anorexia nervosa	F50.0, F50.00, F50.01, F50.02, F50.1, F50.2, F50.3, F50.4, F50.5	307.1, 307.51, 307.54
Aortic aneurysm	I71, I71.0, I71.00, I71.01, I71.02, I71.03, I71.1, I71.2, I71.3, I71.4, I71.5, I71.6, I71.8, I71.9	441, 441.0, 441.00, 441.01, 441.02, 441.03, 441.1, 441.2, 441.3, 441.4, 441.5, 441.6, 441.7, 441.9

Table S4. List of infectious syndromes mapped to International Classification of Diseases (ICD) codes

Cause Name	ICD 10 Codes	ICD 9 Codes
Appendicitis	K35, K35.0, K35.1, K35.2, K35.3, K35.8, K35.80, K35.89, K35.9, K36, K36.0, K37, K37.0, K37.9, K38.3, K38.8, K38.9	540, 540.0, 540.1, 540.9, 541, 541.0, 541.1, 541.2, 541.3, 541.9, 542, 542.0, 542.1, 542.9
Asthma	J45, J45.0, J45.1, J45.2, J45.20, J45.21, J45.22, J45.3, J45.30, J45.31, J45.32, J45.4, J45.40, J45.41, J45.42, J45.5, J45.50, J45.51, J45.52, J45.8, J45.9, J45.90, J45.901, J45.902, J45.909, J45.99, J45.990, J45.991, J45.998, J46, J46.0, J46.9	493, 493.0, 493.00, 493.01, 493.02, 493.1, 493.10, 493.11, 493.12, 493.2, 493.20, 493.21, 493.22, 493.3, 493.4, 493.8, 493.81, 493.82, 493.9, 493.90, 493.91, 493.92
Atrial fibrillation and flutter	I48, I48.0, I48.1, I48.2, I48.3, I48.4, I48.9, I48.91, I48.92	427.3, 427.31, 427.32
Bladder cancer	C67, C67.0, C67.1, C67.2, C67.3, C67.4, C67.5, C67.6, C67.7, C67.8, C67.9, D09.0, D30.3, D41.4, D41.7, D41.8, D49.4	188, 188.0, 188.1, 188.2, 188.3, 188.4, 188.5, 188.6, 188.7, 188.8, 188.9, 223.3, 233.7, 236.7, 239.4
Brain and central nervous system cancer	C70, C70.0, C70.1, C70.5, C70.6, C70.9, C71, C71.0, C71.1, C71.2, C71.3, C71.4, C71.5, C71.6, C71.7, C71.8, C71.9, C72, C72.0, C72.1, C72.2, C72.20, C72.21, C72.22, C72.3, C72.30, C72.31, C72.32, C72.4, C72.40, C72.41, C72.42, C72.5, C72.50, C72.59, C72.8, C72.9, C75.1, C75.2, C75.3	191, 191.0, 191.1, 191.2, 191.3, 191.4, 191.5, 191.6, 191.7, 191.8, 191.9, 192, 192.0, 192.1, 192.2, 192.3, 192.8, 192.9, 194.3, 194.4
Breast cancer	C50, C50.0, C50.01, C50.011, C50.012, C50.019, C50.02, C50.021, C50.022, C50.029, C50.1, C50.11, C50.111, C50.112, C50.119, C50.12, C50.121, C50.122, C50.129, C50.2, C50.21, C50.211, C50.212, C50.219, C50.22, C50.221, C50.222, C50.229, C50.3, C50.31, C50.311, C50.312, C50.319, C50.32, C50.321, C50.322, C50.329, C50.4, C50.41, C50.411, C50.412, C50.419, C50.42, C50.421, C50.422, C50.429, C50.5, C50.51, C50.511, C50.512, C50.519, C50.52, C50.521, C50.522, C50.529, C50.6, C50.61, C50.611, C50.612, C50.619, C50.62, C50.621, C50.622, C50.629, C50.7, C50.8, C50.81, C50.811, C50.812, C50.819, C50.82, C50.821, C50.822, C50.829, C50.9, C50.91, C50.911, C50.912, C50.919, C50.92, C50.921, C50.922, C50.929, D05, D05.0, D05.00, D05.01, D05.02, D05.1, D05.10, D05.11, D05.12, D05.7, D05.8, D05.80, D05.81, D05.82, D05.9, D05.90, D05.91, D05.92, D24, D24.0, D24.1, D24.2, D24.9, D48.6, D48.60, D48.61, D48.62, D49.3	174, 174.0, 174.1, 174.2, 174.3, 174.4, 174.5, 174.6, 174.8, 174.9, 175, 175.0, 175.3, 175.9, 217, 217.0, 217.8, 233.0, 238.3, 239.3, 610, 610.0, 610.1, 610.2, 610.3, 610.4, 610.8, 610.9
Cellulitis	H05.0, H05.00, H05.01, H05.011, H05.012, H05.013, H05.019, L03, L03.0, L03.01, L03.011, L03.012, L03.019, L03.02, L03.021, L03.022, L03.029, L03.03, L03.031, L03.032, L03.039, L03.04, L03.041, L03.042, L03.049, L03.1, L03.11, L03.111, L03.112, L03.113, L03.114, L03.115, L03.116, L03.119, L03.12, L03.121, L03.122, L03.123, L03.124, L03.125, L03.126, L03.129, L03.2, L03.21, L03.211, L03.212, L03.22, L03.221, L03.222, L03.3, L03.31, L03.311, L03.312, L03.313, L03.314, L03.315, L03.316, L03.317, L03.319, L03.32, L03.321, L03.322, L03.323, L03.324, L03.325, L03.326, L03.327, L03.329, L03.8, L03.81, L03.811, L03.818, L03.89, L03.891, L03.898, L03.9, L03.90, L03.91, M72.5, M72.6	681, 681.0, 681.00, 681.01, 681.02, 681.1, 681.10, 681.11, 681.2, 681.9, 682, 682.0, 682.1, 682.2, 682.3, 682.4, 682.5, 682.6, 682.7, 682.8, 682.9, 728.86
Cervical cancer	C53, C53.0, C53.1, C53.3, C53.4, C53.8, C53.9, D06, D06.0, D06.1, D06.7, D06.9, D26.0	180, 180.0, 180.1, 180.2, 180.3, 180.4, 180.5, 180.6, 180.8, 180.9, 219.0, 233.1, 622.1, 622.10, 622.11, 622.12, 622.2, 622.7
Chagas disease	B57, B57.0, B57.1, B57.2, B57.3, B57.30, B57.31, B57.32, B57.39, B57.4, B57.40, B57.41, B57.42, B57.49, B57.5, K93.1	086, 086.0, 086.1, 086.2, 086.9, 425.6
Chlamydial infection	A55, A56, A56.0, A56.00, A56.01, A56.02, A56.09, A56.1, A56.11, A56.19, A56.2, A56.3, A56.4, A56.8, K67.0	
Chronic kidney disease due to diabetes mellitus type 1	E10.2, E10.21, E10.22, E10.29	250.41, 250.43
Chronic kidney disease due to diabetes mellitus type 2	E11.2, E11.21, E11.22, E11.29	250.40, 250.42
Chronic kidney disease due to glomerulonephritis	N03, N03.0, N03.1, N03.2, N03.3, N03.4, N03.5, N03.6, N03.7, N03.8, N03.9, N04, N04.0, N04.1, N04.2, N04.3, N04.4, N04.5, N04.6, N04.7, N04.8, N04.9, N05, N05.0, N05.1, N05.2, N05.3, N05.4, N05.5, N05.6, N05.7, N05.8, N05.9, N06, N06.0, N06.1, N06.2, N06.3, N06.4, N06.5, N06.6, N06.7, N06.8, N06.9	581, 581.0, 581.1, 581.2, 581.3, 581.8, 581.81, 581.89, 581.9, 582, 582.0, 582.1, 582.2, 582.4, 582.8, 582.81, 582.89, 582.9, 583, 583.0, 583.1, 583.2, 583.4, 583.6, 583.7, 583.8, 583.81, 583.89, 583.9
Chronic kidney disease due to hypertension	I12, I12.0, I12.1, I12.2, I12.9, I13, I13.0, I13.1, I13.10, I13.11, I13.2, I13.9	403, 403.0, 403.00, 403.01, 403.1, 403.10, 403.11, 403.6, 403.9, 403.90, 403.91, 404, 404.0, 404.00, 404.01, 404.02, 404.03, 404.1, 404.10, 404.11, 404.12, 404.13, 404.9, 404.90, 404.91, 404.92, 404.93

Table S4. List of infectious syndromes mapped to International Classification of Diseases (ICD) codes

Cause Name	ICD 10 Codes	ICD 9 Codes
Chronic kidney disease due to other and unspecified causes	N02, N02.0, N02.1, N02.2, N02.3, N02.4, N02.5, N02.6, N02.7, N02.8, N02.9, N07, N07.0, N07.1, N07.2, N07.3, N07.4, N07.5, N07.6, N07.7, N07.8, N07.9, N08, N08.0, N08.1, N08.2, N08.3, N08.4, N08.5, N08.8, N15.0, Q61, Q61.0, Q61.00, Q61.01, Q61.02, Q61.1, Q61.11, Q61.19, Q61.2, Q61.3, Q61.4, Q61.5, Q61.8, Q61.9, Q62, Q62.0, Q62.1, Q62.10, Q62.11, Q62.12, Q62.2, Q62.3, Q62.31, Q62.32, Q62.39, Q62.4, Q62.5, Q62.6, Q62.60, Q62.61, Q62.62, Q62.63, Q62.69, Q62.7, Q62.8	589, 589.0, 589.1, 589.4, 589.9, 753, 753.0, 753.1, 753.10, 753.11, 753.12, 753.13, 753.14, 753.15, 753.16, 753.17, 753.19, 753.2, 753.20, 753.21, 753.22, 753.23, 753.29, 753.3
Chronic obstructive pulmonary disease	J41, J41.0, J41.1, J41.8, J42, J42.0, J42.1, J42.4, J42.6, J42.9, J43, J43.0, J43.1, J43.2, J43.8, J43.9, J44, J44.0, J44.1, J44.8, J44.9	491, 491.0, 491.1, 491.2, 491.20, 491.21, 491.22, 491.8, 491.9, 492, 492.0, 492.1, 492.2, 492.8, 492.9, 496, 496.0, 496.1, 496.6, 496.9, 497, 497.0, 498, 499
Cirrhosis and other chronic liver diseases	B18, B18.0, B18.1, B18.2, B18.8, B18.9, I85, I85.0, I85.00, I85.01, I85.1, I85.10, I85.11, I85.9, I98.2, K70, K70.0, K70.1, K70.10, K70.11, K70.2, K70.3, K70.30, K70.31, K71.7, K73, K73.0, K73.1, K73.2, K73.8, K73.9, K74, K74.0, K74.1, K74.2, K74.3, K74.4, K74.5, K74.6, K74.60, K74.69, K74.7, K74.8, K74.9, K75, K75.2, K75.4, K75.8, K75.81, K75.89, K75.9, K76, K76.0, K76.1, K76.2, K76.4, K76.5, K76.6, K76.7, K76.8, K76.81, K76.89, K76.9, K77.8	070.22, 070.23, 070.54, 456.0, 456.1, 456.2, 456.20, 456.21, 571, 571.0, 571.1, 571.2, 571.3, 571.4, 571.40, 571.5, 571.6, 571.8, 571.9, 572.2, 572.3, 572.4, 572.5, 572.6, 572.8, 572.9, 573, 573.0, 573.4, 573.5, 573.8, 573.9
Cocaine use disorders	F14, F14.0, F14.1, F14.10, F14.12, F14.120, F14.121, F14.122, F14.129, F14.14, F14.15, F14.150, F14.151, F14.159, F14.18, F14.180, F14.181, F14.182, F14.188, F14.19, F14.2, F14.20, F14.21, F14.22, F14.220, F14.221, F14.222, F14.229, F14.23, F14.24, F14.25, F14.250, F14.251, F14.259, F14.28, F14.280, F14.281, F14.282, F14.288, F14.29, F14.3, F14.4, F14.5, F14.6, F14.7, F14.8, F14.9, F14.90, F14.92, F14.920, F14.921, F14.922, F14.929, F14.94, F14.95, F14.950, F14.951, F14.959, F14.98, F14.980, F14.981, F14.982, F14.988, F14.99, R78.2	304.2, 304.20, 304.21, 304.22, 304.23, 305.6, 305.60, 305.61, 305.62, 305.63
Colon and rectum cancer	C18, C18.0, C18.1, C18.2, C18.3, C18.4, C18.5, C18.6, C18.7, C18.8, C18.9, C19, C19.0, C19.9, C20, C20.0, C20.8, C20.9, C21, C21.0, C21.1, C21.2, C21.8, C21.9, D01.0, D01.1, D01.2, D01.3, D12, D12.0, D12.1, D12.2, D12.3, D12.4, D12.5, D12.6, D12.7, D12.8, D12.9, D37.3, D37.4, D37.5	153, 153.0, 153.1, 153.2, 153.3, 153.4, 153.5, 153.6, 153.7, 153.8, 153.9, 154, 154.0, 154.1, 154.2, 154.3, 154.4, 154.8, 154.9, 209.1, 209.10, 209.11, 209.12, 209.13, 209.14, 209.15, 209.16, 209.17, 209.5, 209.50, 209.51, 209.52, 209.53, 209.54, 209.55, 209.56, 209.57, 211.3, 211.4, 230.3, 230.4, 230.5, 230.6, 569.0, 569.43, 569.44, 569.84, 569.85
Conflict and terrorism	U00, U01, U02, U03, Y38, Y38.0, Y38.1, Y38.2, Y38.3, Y38.4, Y38.5, Y38.6, Y38.7, Y38.8, Y38.80, Y38.81, Y38.811, Y38.812, Y38.89, Y38.891, Y38.892, Y38.893, Y38.9	E999.1
Congenital heart anomalies	Q20, Q20.0, Q20.1, Q20.2, Q20.3, Q20.4, Q20.5, Q20.6, Q20.8, Q20.9, Q21, Q21.0, Q21.1, Q21.2, Q21.3, Q21.4, Q21.8, Q21.9, Q22, Q22.0, Q22.1, Q22.2, Q22.3, Q22.4, Q22.5, Q22.6, Q22.8, Q22.9, Q23, Q23.0, Q23.1, Q23.2, Q23.3, Q23.4, Q23.8, Q23.9, Q24, Q24.0, Q24.1, Q24.2, Q24.3, Q24.4, Q24.5, Q24.6, Q24.8, Q24.9, Q25, Q25.0, Q25.1, Q25.2, Q25.3, Q25.4, Q25.5, Q25.6, Q25.7, Q25.71, Q25.72, Q25.79, Q25.8, Q25.9, Q26, Q26.0, Q26.1, Q26.2, Q26.3, Q26.4, Q26.5, Q26.6, Q26.8, Q26.9, Q27, Q27.0, Q27.1, Q27.2, Q27.3, Q27.30, Q27.31, Q27.32, Q27.33, Q27.34, Q27.39, Q27.4, Q27.8, Q27.9, Q28, Q28.0, Q28.1, Q28.2, Q28.3, Q28.8, Q28.9	745, 745.0, 745.1, 745.10, 745.11, 745.12, 745.19, 745.2, 745.3, 745.4, 745.5, 745.6, 745.60, 745.61, 745.69, 745.7, 745.8, 745.9, 746, 746.0, 746.00, 746.01, 746.02, 746.09, 746.1, 746.2, 746.3, 746.4, 746.5, 746.6, 746.7, 746.8, 746.81, 746.82, 746.83, 746.84, 746.85, 746.86, 746.87, 746.89, 746.9, 747, 747.0, 747.1, 747.10, 747.11, 747.2, 747.20, 747.21, 747.22, 747.29, 747.3, 747.4, 747.40, 747.41, 747.42, 747.49, 747.5, 747.6, 747.60, 747.61, 747.62, 747.63, 747.64, 747.69, 747.8, 747.81, 747.82, 747.83, 747.89, 747.9

Table S4. List of infectious syndromes mapped to International Classification of Diseases (ICD) codes

Cause Name	ICD 10 Codes	ICD 9 Codes
Congenital musculoskeletal and limb anomalies	Q65, Q65.0, Q65.00, Q65.01, Q65.02, Q65.1, Q65.2, Q65.3, Q65.30, Q65.31, Q65.32, Q65.4, Q65.5, Q65.6, Q65.8, Q65.81, Q65.82, Q65.89, Q65.9, Q66, Q66.0, Q66.1, Q66.2, Q66.3, Q66.4, Q66.5, Q66.50, Q66.51, Q66.52, Q66.6, Q66.7, Q66.8, Q66.80, Q66.81, Q66.82, Q66.89, Q66.9, Q67, Q67.0, Q67.1, Q67.2, Q67.3, Q67.4, Q67.5, Q67.6, Q67.7, Q67.8, Q68, Q68.0, Q68.1, Q68.2, Q68.3, Q68.4, Q68.5, Q68.6, Q68.8, Q69, Q69.0, Q69.1, Q69.2, Q69.9, Q70, Q70.0, Q70.00, Q70.01, Q70.02, Q70.03, Q70.1, Q70.10, Q70.11, Q70.12, Q70.13, Q70.2, Q70.20, Q70.21, Q70.22, Q70.23, Q70.3, Q70.30, Q70.31, Q70.32, Q70.33, Q70.4, Q70.9, Q71, Q71.0, Q71.00, Q71.01, Q71.02, Q71.03, Q71.1, Q71.10, Q71.11, Q71.12, Q71.13, Q71.2, Q71.20, Q71.21, Q71.22, Q71.23, Q71.3, Q71.30, Q71.31, Q71.32, Q71.33, Q71.4, Q71.40, Q71.41, Q71.42, Q71.43, Q71.5, Q71.50, Q71.51, Q71.52, Q71.53, Q71.6, Q71.60, Q71.61, Q71.62, Q71.63, Q71.8, Q71.81, Q71.811, Q71.812, Q71.813, Q71.819, Q71.89, Q71.891, Q71.892, Q71.893, Q71.899, Q71.9, Q71.90, Q71.91, Q71.92, Q71.93, Q72, Q72.0, Q72.00, Q72.01, Q72.02, Q72.03, Q72.1, Q72.10, Q72.11, Q72.12, Q72.13, Q72.2, Q72.20, Q72.21, Q72.22, Q72.23, Q72.3, Q72.30, Q72.31, Q72.32, Q72.33, Q72.4, Q72.40, Q72.41, Q72.42, Q72.43, Q72.5, Q72.50, Q72.51, Q72.52, Q72.53, Q72.6, Q72.60, Q72.61, Q72.62, Q72.63, Q72.7, Q72.70, Q72.71, Q72.72, Q72.73, Q72.8, Q72.81, Q72.811, Q72.812, Q72.813, Q72.819, Q72.89, Q72.891, Q72.892, Q72.893, Q72.899, Q72.9, Q72.90, Q72.91, Q72.92, Q72.93, Q73, Q73.0, Q73.1, Q73.8, Q74, Q74.0, Q74.1, Q74.2, Q74.3, Q74.8, Q74.9, Q75, Q75.0, Q75.1, Q75.2, Q75.3, Q75.4, Q75.5, Q75.8, Q75.9, Q76, Q76.0, Q76.1, Q76.2, Q76.3, Q76.4, Q76.41, Q76.411, Q76.412, Q76.413, Q76.414, Q76.415, Q76.419, Q76.42, Q76.425, Q76.426, Q76.427, Q76.428, Q76.429, Q76.49, Q76.5, Q76.6, Q76.7, Q76.8, Q76.9, Q77, Q77.0, Q77.1, Q77.2, Q77.3, Q77.4, Q77.5, Q77.6, Q77.7, Q77.8, Q77.9, Q78, Q78.0, Q78.1, Q78.2, Q78.3, Q78.4, Q78.5, Q78.6, Q78.8, Q78.9, Q79, Q79.6, Q79.8, Q79.9	742.5, 754, 754.0, 754.1, 754.2, 754.3, 754.30, 754.31, 754.32, 754.33, 754.35, 754.4, 754.40, 754.41, 754.42, 754.43, 754.44, 754.5, 754.50, 754.51, 754.52, 754.53, 754.59, 754.6, 754.60, 754.61, 754.62, 754.69, 754.7, 754.70, 754.71, 754.79, 754.8, 754.81, 754.82, 754.89, 754.9, 755, 755.0, 755.00, 755.01, 755.02, 755.1, 755.10, 755.11, 755.12, 755.13, 755.14, 755.2, 755.20, 755.21, 755.22, 755.23, 755.24, 755.25, 755.26, 755.27, 755.28, 755.29, 755.3, 755.30, 755.31, 755.32, 755.33, 755.34, 755.35, 755.36, 755.37, 755.38, 755.39, 755.4, 755.5, 755.50, 755.51, 755.52, 755.53, 755.54, 755.55, 755.56, 755.57, 755.58, 755.59, 755.6, 755.60, 755.61, 755.62, 755.63, 755.64, 755.65, 755.66, 755.67, 755.69, 755.8, 755.9, 756, 756.0, 756.1, 756.10, 756.11, 756.12, 756.13, 756.14, 756.15, 756.16, 756.17, 756.19, 756.2, 756.3, 756.4, 756.5, 756.50, 756.51, 756.52, 756.53, 756.54, 756.55, 756.56, 756.59, 756.8, 756.81, 756.82, 756.83, 756.89, 756.9
COVID-19	U07, U07.1, U07.2	
Crohn's disease	K50, K50.0, K50.00, K50.01, K50.011, K50.012, K50.013, K50.014, K50.018, K50.019, K50.1, K50.10, K50.11, K50.111, K50.112, K50.113, K50.114, K50.118, K50.119, K50.8, K50.80, K50.81, K50.811, K50.812, K50.813, K50.814, K50.818, K50.819, K50.9, K50.90, K50.91, K50.911, K50.912, K50.913, K50.914, K50.918, K50.919, M09.1	555, 555.0, 555.1, 555.2, 555.3, 555.9
Cystic echinococcosis	B67, B67.0, B67.1, B67.2, B67.3, B67.31, B67.32, B67.39, B67.4, B67.8, B67.9, B67.90, B67.99	122, 122.0, 122.1, 122.2, 122.3, 122.4, 122.8, 122.9
Cysticercosis	B69, B69.0, B69.1, B69.8, B69.81, B69.89, B69.9	123.1
Decubitus ulcer	L89, L89.0, L89.00, L89.000, L89.001, L89.002, L89.003, L89.004, L89.009, L89.01, L89.010, L89.011, L89.012, L89.013, L89.014, L89.019, L89.02, L89.020, L89.021, L89.022, L89.023, L89.024, L89.029, L89.1, L89.10, L89.100, L89.101, L89.102, L89.103, L89.104, L89.109, L89.11, L89.110, L89.111, L89.112, L89.113, L89.114, L89.119, L89.12, L89.120, L89.121, L89.122, L89.123, L89.124, L89.129, L89.13, L89.130, L89.131, L89.132, L89.133, L89.134, L89.139, L89.14, L89.140, L89.141, L89.142, L89.143, L89.144, L89.149, L89.15, L89.150, L89.151, L89.152, L89.153, L89.154, L89.159, L89.2, L89.20, L89.200, L89.201, L89.202, L89.203, L89.204, L89.209, L89.21, L89.210, L89.211, L89.212, L89.213, L89.214, L89.219, L89.22, L89.220, L89.221, L89.222, L89.223, L89.224, L89.229, L89.3, L89.30, L89.300, L89.301, L89.302, L89.303, L89.304, L89.309, L89.31, L89.310, L89.311, L89.312, L89.313, L89.314, L89.319, L89.32, L89.320, L89.321, L89.322, L89.323, L89.324, L89.329, L89.4, L89.40, L89.41, L89.42, L89.43, L89.44, L89.45, L89.5, L89.50, L89.500, L89.501, L89.502, L89.503, L89.504, L89.509, L89.51, L89.510, L89.511, L89.512, L89.513, L89.514, L89.519, L89.52, L89.520, L89.521, L89.522, L89.523, L89.524, L89.529, L89.6, L89.60, L89.600, L89.601, L89.602, L89.603, L89.604, L89.609, L89.61, L89.610, L89.611, L89.612, L89.613, L89.614, L89.619, L89.62, L89.620, L89.621, L89.622, L89.623, L89.624, L89.629, L89.8, L89.81, L89.810, L89.811, L89.812, L89.813, L89.814, L89.819, L89.89, L89.890, L89.891, L89.892, L89.893, L89.894, L89.899, L89.9, L89.90, L89.91, L89.92, L89.93, L89.94, L89.95	707, 707.0, 707.00, 707.01, 707.02, 707.03, 707.04, 707.05, 707.06, 707.07, 707.09, 707.1, 707.10, 707.11, 707.12, 707.13, 707.14, 707.15, 707.19, 707.2, 707.20, 707.21, 707.22, 707.23, 707.24, 707.25, 707.7, 707.8, 707.9
Dengue	A90, A90.0, A91, A91.0, A91.9	061, 061.0, 061.1, 061.6, 061.8

Table S4. List of infectious syndromes mapped to International Classification of Diseases (ICD) codes

Cause Name	ICD 10 Codes	ICD 9 Codes
Diabetes mellitus type 1	E10, E10.0, E10.1, E10.10, E10.11, E10.3, E10.31, E10.311, E10.319, E10.32, E10.321, E10.329, E10.33, E10.331, E10.339, E10.34, E10.341, E10.349, E10.35, E10.351, E10.359, E10.36, E10.39, E10.4, E10.40, E10.41, E10.42, E10.43, E10.44, E10.49, E10.5, E10.51, E10.52, E10.59, E10.6, E10.61, E10.610, E10.618, E10.62, E10.620, E10.621, E10.622, E10.628, E10.63, E10.630, E10.638, E10.64, E10.641, E10.649, E10.65, E10.69, E10.7, E10.8, E10.9, P70.2	250.01, 250.03, 250.11, 250.13, 250.21, 250.23, 250.31, 250.33, 250.51, 250.53, 250.61, 250.63, 250.71, 250.73, 250.81, 250.83, 250.91, 250.93, 775.1
Diabetes mellitus type 2	E11, E11.0, E11.00, E11.01, E11.1, E11.3, E11.31, E11.311, E11.319, E11.32, E11.321, E11.329, E11.33, E11.331, E11.339, E11.34, E11.341, E11.349, E11.35, E11.351, E11.359, E11.36, E11.39, E11.4, E11.40, E11.41, E11.42, E11.43, E11.44, E11.49, E11.5, E11.51, E11.52, E11.59, E11.6, E11.61, E11.610, E11.618, E11.62, E11.620, E11.621, E11.622, E11.628, E11.63, E11.630, E11.638, E11.64, E11.641, E11.649, E11.65, E11.69, E11.7, E11.8, E11.9	250.00, 250.02, 250.10, 250.12, 250.20, 250.22, 250.30, 250.32, 250.50, 250.52, 250.60, 250.62, 250.70, 250.72, 250.80, 250.82, 250.90, 250.92
Diarrheal diseases		558.2, 558.3, 558.4, 558.41, 558.42, 558.9
Digestive congenital anomalies	Q38, Q38.0, Q38.1, Q38.2, Q38.3, Q38.4, Q38.5, Q38.6, Q38.7, Q38.8, Q39, Q39.0, Q39.1, Q39.2, Q39.3, Q39.4, Q39.5, Q39.6, Q39.8, Q39.9, Q40, Q40.0, Q40.1, Q40.2, Q40.3, Q40.8, Q40.9, Q41, Q41.0, Q41.1, Q41.2, Q41.8, Q41.9, Q42, Q42.0, Q42.1, Q42.2, Q42.3, Q42.8, Q42.9, Q43, Q43.0, Q43.1, Q43.2, Q43.3, Q43.4, Q43.5, Q43.6, Q43.7, Q43.8, Q43.9, Q44, Q44.0, Q44.1, Q44.2, Q44.3, Q44.4, Q44.5, Q44.6, Q44.7, Q45, Q45.0, Q45.1, Q45.2, Q45.3, Q45.8, Q45.9, Q79.0, Q79.1, Q79.2, Q79.3, Q79.4, Q79.5, Q79.51, Q79.59	750, 750.0, 750.1, 750.10, 750.11, 750.12, 750.13, 750.15, 750.16, 750.19, 750.2, 750.21, 750.22, 750.23, 750.24, 750.25, 750.26, 750.27, 750.29, 750.3, 750.4, 750.5, 750.6, 750.7, 750.8, 750.9, 751, 751.0, 751.1, 751.2, 751.3, 751.4, 751.5, 751.6, 751.60, 751.61, 751.62, 751.69, 751.7, 751.8, 751.9, 756.6, 756.7, 756.71, 756.79
Diphtheria	A36, A36.0, A36.1, A36.2, A36.3, A36.8, A36.81, A36.82, A36.83, A36.84, A36.85, A36.86, A36.89, A36.9	032, 032.0, 032.1, 032.2, 032.3, 032.6, 032.8, 032.81, 032.82, 032.83, 032.84, 032.85, 032.89, 032.9
Down syndrome	Q90, Q90.0, Q90.1, Q90.2, Q90.9	758
Drowning	W65, W65.0, W65.1, W65.2, W65.3, W65.4, W65.5, W65.6, W65.7, W65.8, W65.9, W66, W66.0, W66.1, W66.2, W66.3, W66.4, W66.5, W66.6, W66.7, W66.8, W66.9, W67, W67.0, W67.1, W67.2, W67.3, W67.4, W67.5, W67.6, W67.7, W67.8, W67.9, W68, W68.0, W68.1, W68.2, W68.3, W68.4, W68.5, W68.6, W68.7, W68.8, W68.9, W69, W69.0, W69.1, W69.2, W69.3, W69.4, W69.5, W69.6, W69.7, W69.8, W69.9, W70, W70.0, W70.1, W70.2, W70.3, W70.4, W70.5, W70.6, W70.7, W70.8, W70.9, W73, W73.0, W73.1, W73.2, W73.3, W73.4, W73.5, W73.6, W73.7, W73.8, W73.9, W74, W74.0, W74.1, W74.2, W74.3, W74.4, W74.5, W74.6, W74.7, W74.8, W74.9	E910, E910.0, E910.00, E910.01, E910.02, E910.03, E910.04, E910.05, E910.06, E910.07, E910.08, E910.09, E910.1, E910.10, E910.11, E910.12, E910.13, E910.14, E910.15, E910.16, E910.17, E910.18, E910.19, E910.2, E910.20, E910.21, E910.22, E910.23, E910.24, E910.25, E910.26, E910.27, E910.28, E910.29, E910.3, E910.30, E910.31, E910.32, E910.33, E910.34, E910.35, E910.36, E910.37, E910.38, E910.39, E910.4, E910.40, E910.41, E910.42, E910.43, E910.44, E910.45, E910.46, E910.47, E910.48, E910.49, E910.8, E910.80, E910.81, E910.82, E910.83, E910.84, E910.85, E910.86, E910.87, E910.88, E910.89, E910.9, E910.90, E910.91, E910.92, E910.93, E910.94, E910.95, E910.96, E910.97, E910.98, E910.99
Ebola	A98.4	
Ectopic pregnancy	O00, O00.0, O00.1, O00.2, O00.8, O00.9	633, 633.0, 633.00, 633.01, 633.1, 633.10, 633.11, 633.2, 633.20, 633.21, 633.8, 633.80, 633.81, 633.9, 633.90, 633.91
Electrocution	W85, W85.0, W85.1, W85.2, W85.3, W85.4, W85.5, W85.6, W85.7, W85.8, W85.9, W86, W86.0, W86.1, W86.2, W86.3, W86.4, W86.5, W86.6, W86.7, W86.8, W86.9, W87, W87.0, W87.1, W87.2, W87.3, W87.4, W87.5, W87.6, W87.7, W87.8, W87.9	E925, E925.0, E925.00, E925.01, E925.02, E925.03, E925.04, E925.05, E925.06, E925.07, E925.08, E925.09, E925.1, E925.10, E925.11, E925.12, E925.13, E925.14, E925.15, E925.16, E925.17, E925.18, E925.19, E925.2, E925.20, E925.21, E925.22, E925.23, E925.24, E925.25, E925.26, E925.27, E925.28, E925.29, E925.8, E925.80, E925.81, E925.82, E925.83, E925.84, E925.85, E925.86, E925.87, E925.88, E925.89, E925.9, E925.90, E925.91, E925.92, E925.93, E925.94, E925.95, E925.96, E925.97, E925.98, E925.99
Encephalitis	A83, A83.0, A83.1, A83.2, A83.3, A83.4, A83.5, A83.6, A83.8, A83.9, A84, A84.0, A84.1, A84.8, A84.9, A85, A85.0, A85.1, A85.2, A85.3, A85.4, A85.8, A86, A86.0, A86.4, B94.1, F07.1, G04, G04.0, G04.00, G04.01, G04.02, G04.1, G04.2, G04.3, G04.30, G04.31, G04.32, G04.39, G04.8, G04.81, G04.89, G04.9, G04.90, G04.91, G05, G05.0, G05.1, G05.2, G05.3, G05.4, G05.8, G21.3	062, 062.0, 062.1, 062.2, 062.3, 062.4, 062.5, 062.6, 062.7, 062.8, 062.9, 063, 063.0, 063.1, 063.2, 063.3, 063.4, 063.7, 063.8, 063.9, 064, 064.0, 064.1, 064.3, 064.4, 064.9, 139.0, 323, 323.4, 323.41, 323.42, 323.5, 323.51, 323.52, 323.6, 323.61, 323.62, 323.63, 323.7, 323.71, 323.72, 323.8, 323.81, 323.82, 323.9
Endocarditis	B33.21, I33, I33.0, I33.9, I38, I38.0, I38.9, I39, I39.0, I39.1, I39.2, I39.3, I39.4, I39.8, I39.9	421, 421.0, 421.1, 421.9
Endometriosis	N80, N80.0, N80.1, N80.2, N80.3, N80.4, N80.5, N80.6, N80.8, N80.9	617, 617.0, 617.1, 617.2, 617.3, 617.4, 617.5, 617.6, 617.8, 617.9

Table S4. List of infectious syndromes mapped to International Classification of Diseases (ICD) codes

Cause Name	ICD 10 Codes	ICD 9 Codes
Environmental heat and cold exposure	L55, L55.0, L55.1, L55.2, L55.8, L55.9, L56.3, L56.8, L56.9, L58, L58.0, L58.1, L58.9, W88, W88.0, W88.1, W88.2, W88.4, W88.5, W88.6, W88.7, W88.8, W88.9, W89, W89.0, W89.1, W89.2, W89.3, W89.4, W89.5, W89.6, W89.7, W89.8, W89.9, W90, W90.0, W90.1, W90.2, W90.3, W90.5, W90.6, W90.7, W90.8, W90.9, W91, W91.0, W91.1, W91.2, W91.3, W91.4, W91.6, W91.7, W91.8, W91.9, W92, W92.0, W92.1, W92.2, W92.3, W92.4, W92.5, W92.6, W92.7, W92.8, W92.9, W93, W93.0, W93.01, W93.02, W93.1, W93.11, W93.12, W93.2, W93.3, W93.4, W93.5, W93.6, W93.7, W93.8, W93.9, W94, W94.0, W94.1, W94.11, W94.12, W94.2, W94.21, W94.22, W94.23, W94.29, W94.3, W94.31, W94.32, W94.39, W94.4, W94.5, W94.6, W94.7, W94.8, W94.9, W97.9, W99, W99.0, W99.1, W99.2, W99.3, W99.4, W99.6, W99.7, W99.8, W99.9, X30, X30.0, X30.1, X30.2, X30.3, X30.4, X30.5, X30.6, X30.7, X30.8, X30.9, X31, X31.0, X31.1, X31.2, X31.3, X31.4, X31.5, X31.6, X31.7, X31.8, X31.9, X32, X32.0, X32.1, X32.3, X32.4, X32.5, X32.6, X32.7, X32.8, X32.9, X39, X39.0, X39.01, X39.08, X39.1, X39.2, X39.3, X39.4, X39.5, X39.6, X39.7, X39.8, X39.9	E900, E900.0, E900.00, E900.01, E900.02, E900.03, E900.04, E900.05, E900.06, E900.07, E900.08, E900.09, E900.1, E900.10, E900.11, E900.12, E900.13, E900.14, E900.15, E900.16, E900.17, E900.18, E900.19, E900.9, E900.90, E900.91, E900.92, E900.93, E900.94, E900.95, E900.96, E900.97, E900.98, E900.99, E901, E901.0, E901.00, E901.01, E901.02, E901.03, E901.04, E901.05, E901.06, E901.07, E901.08, E901.09, E901.1, E901.10, E901.11, E901.12, E901.13, E901.14, E901.15, E901.16, E901.17, E901.18, E901.19, E901.8, E901.80, E901.81, E901.82, E901.83, E901.84, E901.85, E901.86, E901.87, E901.88, E901.89, E901.9, E901.90, E901.91, E901.92, E901.93, E901.94, E901.95, E901.96, E901.97, E901.98, E901.99, E902, E902.0, E902.00, E902.01, E902.02, E902.03, E902.04, E902.05, E902.06, E902.07, E902.08, E902.09, E902.1, E902.10, E902.11, E902.12, E902.13, E902.14, E902.15, E902.16, E902.17, E902.18, E902.19, E902.2, E902.20, E902.21, E902.22, E902.23, E902.24, E902.25, E902.26, E902.27, E902.28, E902.29, E902.8, E902.80, E902.81, E902.82, E902.83, E902.84, E902.85, E902.86, E902.87, E902.88, E902.89, E902.9, E902.90, E902.91, E902.92, E902.93, E902.94, E902.95, E902.96, E902.97, E902.98, E902.99, E926, E926.0, E926.00, E926.01, E926.02, E926.03, E926.04, E926.05, E926.06, E926.07, E926.08, E926.09, E926.1, E926.10, E926.11, E926.12, E926.13, E926.14, E926.15, E926.16, E926.17, E926.18, E926.19, E926.2, E926.20, E926.21, E926.22, E926.23, E926.24, E926.25, E926.26, E926.27, E926.28, E926.29, E926.3, E926.30, E926.31, E926.32, E926.33, E926.34, E926.35, E926.36, E926.37, E926.38, E926.39, E926.4, E926.40, E926.41, E926.42, E926.43, E926.44, E926.45, E926.46, E926.47, E926.48, E926.49, E926.5, E926.50, E926.51, E926.52, E926.53, E926.54, E926.55, E926.56, E926.57, E926.58, E926.59, E926.8, E926.80, E926.81, E926.82, E926.83, E926.84, E926.85, E926.86, E926.87, E926.88, E926.89, E926.9, E926.90, E926.91, E926.92, E926.93, E926.94, E926.95, E926.96, E926.97, E926.98, E926.99, E929.5
Esophageal cancer	C15, C15.0, C15.1, C15.2, C15.3, C15.4, C15.5, C15.8, C15.9, D00.1, D13.0	150, 150.0, 150.1, 150.2, 150.3, 150.4, 150.5, 150.6, 150.7, 150.8, 150.9, 211.0, 230.1
Exposure to forces of nature		
Falls	W00, W00.0, W00.1, W00.2, W00.3, W00.4, W00.5, W00.6, W00.7, W00.8, W00.9, W01, W01.0, W01.1, W01.10, W01.11, W01.110, W01.111, W01.118, W01.119, W01.19, W01.190, W01.198, W01.2, W01.3, W01.4, W01.5, W01.6, W01.7, W01.8, W01.9, W02, W02.0, W02.1, W02.2, W02.3, W02.4, W02.5, W02.6, W02.7, W02.8, W02.9, W03, W03.0, W03.1, W03.2, W03.3, W03.4, W03.5, W03.6, W03.7, W03.8, W03.9, W04, W04.0, W04.1, W04.2, W04.3, W04.4, W04.5, W04.6, W04.7, W04.8, W04.9, W05, W05.0, W05.1, W05.2, W05.3, W05.4, W05.5, W05.6, W05.7, W05.8, W05.9, W06, W06.0, W06.1, W06.2, W06.3, W06.4, W06.5, W06.6, W06.7, W06.8, W06.9, W07, W07.0, W07.1, W07.2, W07.3, W07.4, W07.5, W07.6, W07.7, W07.8, W07.9, W08, W08.0, W08.1, W08.2, W08.3, W08.4, W08.5, W08.6, W08.7, W08.8, W08.9, W09, W09.0, W09.1, W09.2, W09.3, W09.4, W09.5, W09.6, W09.7, W09.8, W09.9, W10, W10.0, W10.1, W10.2, W10.3, W10.4, W10.5, W10.6, W10.7, W10.8, W10.9, W11, W11.0, W11.1, W11.2, W11.3, W11.4, W11.5, W11.6, W11.7, W11.8, W11.9, W12, W12.0, W12.1, W12.2, W12.3, W12.4, W12.5, W12.6, W12.7, W12.8, W12.9, W13, W13.0, W13.1, W13.2, W13.3, W13.4, W13.5, W13.6, W13.7, W13.8, W13.9, W14, W14.0, W14.1, W14.2, W14.3, W14.4, W14.5, W14.6, W14.7, W14.8, W14.9, W15, W15.0, W15.1, W15.2, W15.3, W15.4, W15.5, W15.6, W15.7, W15.8, W15.9, W16, W16.0, W16.01, W16.011, W16.012, W16.02, W16.021, W16.022, W16.03, W16.031, W16.032, W16.1, W16.11, W16.111, W16.112, W16.12, W16.121, W16.122, W16.13, W16.131, W16.132, W16.2, W16.21, W16.211, W16.212, W16.22, W16.221, W16.222, W16.3, W16.31, W16.311, W16.312, W16.32, W16.321, W16.322, W16.33, W16.331, W16.332, W16.4, W16.41, W16.42, W16.5, W16.51, W16.511, W16.512, W16.52, W16.521, W16.522, W16.53, W16.531, W16.532, W16.6, W16.61, W16.611, W16.612, W16.62, W16.621, W16.622, W16.7, W16.71, W16.711, W16.712, W16.72, W16.721, W16.722, W16.8, W16.81, W16.811, W16.812, W16.82, W16.821, W16.822, W16.83, W16.831, W16.832, W16.9, W16.91, W16.92, W17, W17.0, W17.1, W17.2, W17.3, W17.4, W17.5, W17.6, W17.7, W17.8, W17.81, W17.82, W17.89, W17.9, W18, W18.0, W18.00, W18.01, W18.02, W18.09, W18.1, W18.11, W18.12, W18.2, W18.3, W18.30, W18.31, W18.39, W18.4, W18.40, W18.41, W18.42, W18.43, W18.49, W18.5, W18.6, W18.7, W18.8, W18.9, W19, W19.0, W19.1, W19.2, W19.3, W19.4, W19.5, W19.6, W19.7, W19.8, W19.9	E880, E880.0, E880.00, E880.01, E880.02, E880.03, E880.04, E880.05, E880.06, E880.07, E880.08, E880.09, E880.1, E880.9, E880.90, E880.91, E880.92, E880.93, E880.94, E880.95, E880.96, E880.97, E880.98, E880.99, E881, E881.0, E881.00, E881.01, E881.02, E881.03, E881.04, E881.05, E881.06, E881.07, E881.08, E881.09, E881.1, E881.10, E881.11, E881.12, E881.13, E881.14, E881.15, E881.16, E881.17, E881.18, E881.19, E882, E882.0, E882.00, E882.01, E882.02, E882.03, E882.04, E882.05, E882.06, E882.07, E882.08, E882.09, E883, E883.0, E883.00, E883.01, E883.02, E883.03, E883.04, E883.05, E883.06, E883.07, E883.08, E883.09, E883.1, E883.10, E883.11, E883.12, E883.13, E883.14, E883.15, E883.16, E883.17, E883.18, E883.19, E883.2, E883.20, E883.21, E883.22, E883.23, E883.24, E883.25, E883.26, E883.27, E883.28, E883.29, E883.9, E883.90, E883.91, E883.92, E883.93, E883.94, E883.95, E883.96, E883.97, E883.98, E883.99, E884, E884.0, E884.00, E884.01, E884.02, E884.03, E884.04, E884.05, E884.06, E884.07, E884.08, E884.09, E884.1, E884.10, E884.11, E884.12, E884.13, E884.14, E884.15, E884.16, E884.17, E884.18, E884.19, E884.2, E884.20, E884.21, E884.22, E884.23, E884.24, E884.25, E884.26, E884.27, E884.28, E884.29, E884.3, E884.4, E884.5, E884.6, E884.9, E884.90, E884.91, E884.92, E884.93, E884.94, E884.95, E884.96, E884.97, E884.98, E884.99, E885, E885.0, E885.00, E885.01, E885.02, E885.03, E885.04, E885.05, E885.06, E885.07, E885.08, E885.09, E885.1, E885.2, E885.3, E885.4, E885.9, E886, E886.0, E886.00, E886.01, E886.02, E886.03, E886.04, E886.05, E886.06, E886.07, E886.08, E886.09, E886.9, E886.90, E886.91, E886.92, E886.93, E886.94, E886.95, E886.96, E886.97, E886.98, E886.99, E888, E888.0, E888.00, E888.01, E888.02, E888.03, E888.04, E888.05, E888.06, E888.07, E888.08, E888.09, E888.1, E888.8, E888.9, E929.3

Table S4. List of infectious syndromes mapped to International Classification of Diseases (ICD) codes

Cause Name	ICD 10 Codes	ICD 9 Codes
Fire, heat, and hot substances	X00, X00.0, X00.1, X00.2, X00.3, X00.4, X00.5, X00.6, X00.7, X00.8, X00.9, X01, X01.0, X01.1, X01.2, X01.3, X01.4, X01.5, X01.6, X01.7, X01.8, X01.9, X02, X02.0, X02.1, X02.2, X02.3, X02.4, X02.5, X02.6, X02.7, X02.8, X02.9, X03, X03.0, X03.1, X03.2, X03.3, X03.4, X03.5, X03.6, X03.7, X03.8, X03.9, X04, X04.0, X04.1, X04.2, X04.3, X04.4, X04.5, X04.6, X04.7, X04.8, X04.9, X05, X05.0, X05.1, X05.2, X05.3, X05.4, X05.5, X05.7, X05.8, X05.9, X06, X06.0, X06.1, X06.2, X06.3, X06.4, X06.5, X06.6, X06.7, X06.8, X06.9, X08, X08.0, X08.00, X08.01, X08.09, X08.1, X08.10, X08.11, X08.19, X08.2, X08.20, X08.21, X08.29, X08.3, X08.4, X08.5, X08.6, X08.7, X08.8, X08.9, X09, X09.0, X09.1, X09.2, X09.3, X09.4, X09.5, X09.6, X09.7, X09.8, X09.9, X10, X10.0, X10.1, X10.2, X10.3, X10.4, X10.5, X10.6, X10.7, X10.8, X10.9, X11, X11.0, X11.1, X11.2, X11.4, X11.5, X11.6, X11.7, X11.8, X11.9, X12, X12.0, X12.1, X12.2, X12.4, X12.5, X12.6, X12.7, X12.8, X12.9, X13, X13.0, X13.1, X13.2, X13.3, X13.4, X13.5, X13.6, X13.7, X13.8, X13.9, X14, X14.0, X14.1, X14.2, X14.3, X14.4, X14.5, X14.6, X14.7, X14.8, X14.9, X15, X15.0, X15.1, X15.2, X15.3, X15.4, X15.5, X15.6, X15.7, X15.8, X15.9, X16, X16.0, X16.1, X16.2, X16.4, X16.5, X16.6, X16.7, X16.8, X16.9, X17, X17.0, X17.1, X17.4, X17.5, X17.6, X17.7, X17.8, X17.9, X18, X18.0, X18.1, X18.2, X18.4, X18.5, X18.6, X18.7, X18.8, X18.9, X19, X19.0, X19.1, X19.2, X19.3, X19.4, X19.5, X19.6, X19.7, X19.8, X19.9	E890, E890.0, E890.00, E890.01, E890.02, E890.03, E890.04, E890.05, E890.06, E890.07, E890.08, E890.09, E890.1, E890.10, E890.11, E890.12, E890.13, E890.14, E890.15, E890.16, E890.17, E890.18, E890.19, E890.2, E890.20, E890.21, E890.22, E890.23, E890.24, E890.25, E890.26, E890.27, E890.28, E890.29, E890.3, E890.30, E890.31, E890.32, E890.33, E890.34, E890.35, E890.36, E890.37, E890.38, E890.39, E890.8, E890.80, E890.81, E890.82, E890.83, E890.84, E890.85, E890.86, E890.87, E890.88, E890.89, E890.9, E890.90, E890.91, E890.92, E890.93, E890.94, E890.95, E890.96, E890.97, E890.98, E890.99, E891, E891.0, E891.00, E891.01, E891.02, E891.03, E891.04, E891.05, E891.06, E891.07, E891.08, E891.09, E891.1, E891.10, E891.11, E891.12, E891.13, E891.14, E891.15, E891.16, E891.17, E891.18, E891.19, E891.2, E891.20, E891.21, E891.22, E891.23, E891.24, E891.25, E891.26, E891.27, E891.28, E891.29, E891.3, E891.30, E891.31, E891.32, E891.33, E891.34, E891.35, E891.36, E891.37, E891.38, E891.39, E891.8, E891.80, E891.81, E891.82, E891.83, E891.84, E891.85, E891.86, E891.87, E891.88, E891.89, E891.9, E891.90, E891.91, E891.92, E891.93, E891.94, E891.95, E891.96, E891.97, E891.98, E891.99, E892, E892.0, E892.00, E892.01, E892.02, E892.03, E892.04, E892.05, E892.06, E892.07, E892.08, E892.09, E893, E893.0, E893.00, E893.01, E893.02, E893.03, E893.04, E893.05, E893.06, E893.07, E893.08, E893.09, E893.1, E893.10, E893.11, E893.12, E893.13, E893.14, E893.15, E893.16, E893.17, E893.18, E893.19, E893.2, E893.20, E893.21, E893.22, E893.23, E893.24, E893.25, E893.26, E893.27, E893.28, E893.29, E893.8, E893.80, E893.81, E893.82, E893.83, E893.84, E893.85, E893.86, E893.87, E893.88, E893.89, E893.9, E893.90, E893.91, E893.92, E893.93, E893.94, E893.95, E893.96, E893.97, E893.98, E893.99, E894, E894.0, E894.00, E894.01, E894.02, E894.03, E894.04, E894.05, E894.06, E894.07, E894.08, E894.09, E895, E895.0, E895.00, E895.01, E895.02, E895.03, E895.04, E895.05, E895.06, E895.07, E895.08, E895.09, E896, E896.0, E896.00, E896.01, E896.02, E896.03, E896.04, E896.05, E896.06, E896.07, E896.08, E896.09, E897, E897.0, E897.00, E897.01, E897.02, E897.03, E897.04, E897.05, E897.06, E897.07, E897.08, E897.09, E898, E898.0, E898.00, E898.01, E898.02, E898.03, E898.04, E898.05, E898.06, E898.07, E898.08, E898.09, E898.1, E898.10, E898.11, E898.12, E898.13, E898.14, E898.15, E898.16, E898.17, E898.18, E898.19
Foreign body in other body part	W44, W44.0, W44.1, W44.2, W44.3, W44.4, W44.5, W44.6, W44.7, W44.8, W44.9, W45, W45.3, W45.4, W45.5, W45.6, W45.7, W45.8, W45.9	E914, E914.0, E914.00, E914.01, E914.02, E914.03, E914.04, E914.05, E914.06, E914.07, E914.08, E914.09, E915, E915.0, E915.00, E915.01, E915.02, E915.03, E915.04, E915.05, E915.06, E915.07, E915.08, E915.09
G6PD deficiency	D55, D55.0, D55.1, D55.2	282.2, 282.3
Gallbladder and biliary diseases	K80, K80.0, K80.00, K80.01, K80.1, K80.10, K80.11, K80.12, K80.13, K80.18, K80.19, K80.2, K80.20, K80.21, K80.3, K80.30, K80.31, K80.32, K80.33, K80.34, K80.35, K80.36, K80.37, K80.4, K80.40, K80.41, K80.42, K80.43, K80.44, K80.45, K80.46, K80.47, K80.5, K80.50, K80.51, K80.6, K80.60, K80.61, K80.62, K80.63, K80.64, K80.65, K80.66, K80.67, K80.7, K80.70, K80.71, K80.8, K80.80, K80.81, K80.9, K81, K81.0, K81.1, K81.2, K81.8, K81.9, K82, K82.0, K82.1, K82.2, K82.3, K82.4, K82.8, K82.9, K83, K83.0, K83.1, K83.2, K83.3, K83.4, K83.5, K83.8, K83.9	574, 574.0, 574.00, 574.01, 574.1, 574.10, 574.11, 574.2, 574.20, 574.21, 574.3, 574.30, 574.31, 574.4, 574.40, 574.41, 574.5, 574.50, 574.51, 574.6, 574.60, 574.61, 574.7, 574.70, 574.71, 574.8, 574.80, 574.81, 574.9, 574.90, 574.91, 575, 575.0, 575.1, 575.10, 575.11, 575.12, 575.2, 575.3, 575.4, 575.5, 575.6, 575.8, 575.9, 576, 576.0, 576.1, 576.2, 576.3, 576.4, 576.5, 576.8, 576.9
Gallbladder and biliary tract cancer	C23, C23.0, C23.9, C24, C24.0, C24.1, C24.4, C24.8, C24.9, D13.5	156, 156.0, 156.1, 156.2, 156.3, 156.8, 156.9, 209.65, 209.66, 209.67
Gastritis and duodenitis	K29, K29.0, K29.00, K29.01, K29.1, K29.2, K29.20, K29.21, K29.3, K29.30, K29.31, K29.4, K29.40, K29.41, K29.5, K29.50, K29.51, K29.6, K29.60, K29.61, K29.7, K29.70, K29.71, K29.8, K29.80, K29.81, K29.9, K29.90, K29.91	535, 535.0, 535.00, 535.01, 535.1, 535.10, 535.11, 535.2, 535.20, 535.21, 535.3, 535.30, 535.31, 535.4, 535.40, 535.41, 535.5, 535.50, 535.51, 535.6, 535.60, 535.61, 535.7, 535.70, 535.71, 535.9
Genital prolapse	N81, N81.0, N81.1, N81.10, N81.11, N81.12, N81.2, N81.3, N81.4, N81.5, N81.6, N81.8, N81.81, N81.82, N81.83, N81.84, N81.85, N81.89, N81.9	618, 618.0, 618.00, 618.01, 618.02, 618.03, 618.04, 618.05, 618.09, 618.1, 618.2, 618.3, 618.4, 618.5, 618.6, 618.7, 618.8, 618.81, 618.82, 618.83, 618.84, 618.89, 618.9
Gonococcal infection	A54, A54.0, A54.00, A54.01, A54.02, A54.03, A54.09, A54.1, A54.2, A54.21, A54.22, A54.23, A54.24, A54.29, A54.3, A54.30, A54.31, A54.32, A54.33, A54.39, A54.4, A54.40, A54.41, A54.42, A54.43, A54.49, A54.5, A54.6, A54.8, A54.81, A54.82, A54.83, A54.84, A54.85, A54.86, A54.89, A54.9, K67.1, M73.0	098, 098.0, 098.1, 098.10, 098.11, 098.12, 098.13, 098.14, 098.15, 098.16, 098.17, 098.19, 098.2, 098.3, 098.30, 098.31, 098.32, 098.33, 098.34, 098.35, 098.36, 098.37, 098.39, 098.4, 098.40, 098.41, 098.42, 098.43, 098.49, 098.5, 098.50, 098.51, 098.52, 098.53, 098.59, 098.6, 098.7, 098.8, 098.81, 098.82, 098.83, 098.84, 098.85, 098.86, 098.89, 098.9
Hemolytic disease and other neonatal jaundice	P55, P55.0, P55.1, P55.8, P55.9, P56, P56.0, P56.9, P56.90, P56.99, P57, P57.0, P57.8, P57.9, P58, P58.0, P58.1, P58.2, P58.3, P58.4, P58.41, P58.42, P58.5, P58.8, P58.9, P59, P59.0, P59.1, P59.2, P59.20, P59.29, P59.3, P59.8, P59.9	773, 773.0, 773.1, 773.2, 773.3, 773.4, 773.5, 773.9, 774, 774.0, 774.1, 774.2, 774.3, 774.30, 774.31, 774.39, 774.4, 774.5, 774.6, 774.7, 774.9
HIV/AIDS		

Table S4. List of infectious syndromes mapped to International Classification of Diseases (ICD) codes		
**Cause Name**	**ICD 10 Codes**	**ICD 9 Codes**
Hodgkin lymphoma	C81, C81.0, C81.00, C81.01, C81.02, C81.03, C81.04, C81.05, C81.06, C81.07, C81.08, C81.09, C81.1, C81.10, C81.11, C81.12, C81.13, C81.14, C81.15, C81.16, C81.17, C81.18, C81.19, C81.2, C81.20, C81.21, C81.22, C81.23, C81.24, C81.25, C81.26, C81.27, C81.28, C81.29, C81.3, C81.30, C81.31, C81.32, C81.33, C81.34, C81.35, C81.36, C81.37, C81.38, C81.39, C81.4, C81.40, C81.41, C81.42, C81.43, C81.44, C81.45, C81.46, C81.47, C81.48, C81.49, C81.5, C81.6, C81.7, C81.70, C81.71, C81.72, C81.73, C81.74, C81.75, C81.76, C81.77, C81.78, C81.79, C81.8, C81.9, C81.90, C81.91, C81.92, C81.93, C81.94, C81.95, C81.96, C81.97, C81.98, C81.99	201, 201.0, 201.00, 201.01, 201.02, 201.03, 201.04, 201.05, 201.06, 201.07, 201.08, 201.1, 201.10, 201.11, 201.12, 201.13, 201.14, 201.15, 201.16, 201.17, 201.18, 201.2, 201.20, 201.21, 201.22, 201.23, 201.24, 201.25, 201.26, 201.27, 201.28, 201.4, 201.40, 201.41, 201.42, 201.43, 201.44, 201.45, 201.46, 201.47, 201.48, 201.5, 201.50, 201.51, 201.52, 201.53, 201.54, 201.55, 201.56, 201.57, 201.58, 201.6, 201.60, 201.61, 201.62, 201.63, 201.64, 201.65, 201.66, 201.67, 201.68, 201.7, 201.70, 201.71, 201.72, 201.73, 201.74, 201.75, 201.76, 201.77, 201.78, 201.9, 201.90, 201.91, 201.92, 201.93, 201.94, 201.95, 201.96, 201.97, 201.98
Hypertensive heart disease	I11, I11.0, I11.2, I11.6, I11.9	402, 402.0, 402.00, 402.01, 402.1, 402.10, 402.11, 402.3, 402.7, 402.9, 402.90, 402.91
Idiopathic epilepsy	G40, G40.0, G40.00, G40.001, G40.009, G40.01, G40.011, G40.019, G40.1, G40.10, G40.101, G40.109, G40.11, G40.111, G40.119, G40.2, G40.20, G40.201, G40.209, G40.21, G40.211, G40.219, G40.3, G40.30, G40.301, G40.309, G40.31, G40.311, G40.319, G40.4, G40.40, G40.401, G40.409, G40.41, G40.411, G40.419, G40.5, G40.50, G40.501, G40.509, G40.6, G40.7, G40.8, G40.80, G40.801, G40.802, G40.803, G40.804, G40.81, G40.811, G40.812, G40.813, G40.814, G40.82, G40.821, G40.822, G40.823, G40.824, G40.89, G40.9, G40.90, G40.901, G40.909, G40.91, G40.911, G40.919, G41, G41.0, G41.1, G41.2, G41.8, G41.9	345, 345.0, 345.00, 345.01, 345.1, 345.10, 345.11, 345.2, 345.3, 345.4, 345.40, 345.41, 345.5, 345.50, 345.51, 345.6, 345.60, 345.61, 345.7, 345.70, 345.71, 345.8, 345.80, 345.81, 345.9, 345.90, 345.91
Inguinal, femoral, and abdominal hernia		552.4, 552.8, 552.9, 553, 553.8, 553.9
Interstitial lung disease and pulmonary sarcoidosis	D86, D86.0, D86.1, D86.2, D86.89, D86.9, J84, J84.0, J84.01, J84.02, J84.03, J84.09, J84.1, J84.10, J84.11, J84.111, J84.112, J84.113, J84.114, J84.115, J84.116, J84.117, J84.2, J84.8, J84.81, J84.82, J84.83, J84.84, J84.841, J84.842, J84.843, J84.848, J84.89, J84.9	135, 135.0, 135.2, 135.9, 515, 516, 516.0, 516.1, 516.2, 516.3, 516.30, 516.31, 516.32, 516.33, 516.34, 516.35, 516.36, 516.37, 516.4, 516.5, 516.6, 516.61, 516.62, 516.63, 516.64, 516.69, 516.8, 516.9
Intestinal nematode infections		
Intracerebral hemorrhage	I61, I61.0, I61.1, I61.2, I61.3, I61.4, I61.5, I61.6, I61.8, I61.9, I62, I62.1, I62.9, I68.1, I68.2, I69.1, I69.10, I69.11, I69.12, I69.120, I69.121, I69.122, I69.123, I69.128, I69.13, I69.131, I69.132, I69.133, I69.134, I69.139, I69.14, I69.141, I69.142, I69.143, I69.144, I69.149, I69.15, I69.151, I69.152, I69.153, I69.154, I69.159, I69.16, I69.161, I69.162, I69.163, I69.164, I69.165, I69.169, I69.19, I69.190, I69.191, I69.192, I69.193, I69.198, I69.2, I69.20, I69.21, I69.22, I69.220, I69.221, I69.222, I69.223, I69.228, I69.23, I69.231, I69.232, I69.233, I69.234, I69.239, I69.24, I69.241, I69.242, I69.243, I69.244, I69.249, I69.25, I69.251, I69.252, I69.253, I69.254, I69.259, I69.26, I69.261, I69.262, I69.263, I69.264, I69.265, I69.269, I69.29, I69.290, I69.291, I69.292, I69.293, I69.298	431, 431.0, 431.1, 431.2, 431.9, 432, 432.0, 432.1, 432.4, 432.5, 432.7, 432.9, 437.2
Invasive Non-typhoidal Salmonella (iNTS)	A02.1, A02.2, A02.20, A02.21, A02.22, A02.23, A02.24, A02.25, A02.29	003, 003.0, 003.1, 003.2, 003.20, 003.21, 003.22, 003.23, 003.24, 003.29, 003.4, 003.5, 003.6, 003.7
Ischemic heart disease		

Table S4. List of infectious syndromes mapped to International Classification of Diseases (ICD) codes		
**Cause Name**	**ICD 10 Codes**	**ICD 9 Codes**
Ischemic stroke	G45, G45.0, G45.1, G45.2, G45.3, G45.4, G45.8, G45.9, G46, G46.0, G46.1, G46.2, G46.3, G46.4, G46.5, G46.6, G46.7, G46.8, I63, I63.0, I63.00, I63.01, I63.011, I63.012, I63.019, I63.02, I63.03, I63.031, I63.032, I63.039, I63.09, I63.1, I63.10, I63.11, I63.111, I63.112, I63.119, I63.12, I63.13, I63.131, I63.132, I63.139, I63.19, I63.2, I63.20, I63.21, I63.211, I63.212, I63.219, I63.22, I63.23, I63.231, I63.232, I63.239, I63.29, I63.3, I63.30, I63.31, I63.311, I63.312, I63.319, I63.32, I63.321, I63.322, I63.329, I63.33, I63.331, I63.332, I63.339, I63.34, I63.341, I63.342, I63.349, I63.39, I63.4, I63.40, I63.41, I63.411, I63.412, I63.419, I63.42, I63.421, I63.422, I63.429, I63.43, I63.431, I63.432, I63.439, I63.44, I63.441, I63.442, I63.449, I63.49, I63.5, I63.50, I63.51, I63.511, I63.512, I63.519, I63.52, I63.521, I63.522, I63.529, I63.53, I63.531, I63.532, I63.539, I63.54, I63.541, I63.542, I63.549, I63.59, I63.6, I63.7, I63.8, I63.9, I65, I65.0, I65.01, I65.02, I65.03, I65.09, I65.1, I65.2, I65.21, I65.22, I65.23, I65.29, I65.3, I65.8, I65.9, I66, I66.0, I66.01, I66.02, I66.03, I66.09, I66.1, I66.11, I66.12, I66.13, I66.19, I66.2, I66.21, I66.22, I66.23, I66.29, I66.3, I66.4, I66.8, I66.9, I67.2, I67.3, I67.5, I67.6, I69.3, I69.30, I69.31, I69.32, I69.320, I69.321, I69.322, I69.323, I69.328, I69.33, I69.331, I69.332, I69.333, I69.334, I69.339, I69.34, I69.341, I69.342, I69.343, I69.344, I69.349, I69.35, I69.351, I69.352, I69.353, I69.354, I69.359, I69.36, I69.361, I69.362, I69.363, I69.364, I69.365, I69.369, I69.39, I69.390, I69.391, I69.392, I69.393, I69.398	433, 433.0, 433.00, 433.01, 433.1, 433.10, 433.11, 433.2, 433.20, 433.21, 433.3, 433.30, 433.31, 433.8, 433.80, 433.81, 433.9, 433.90, 433.91, 434, 434.0, 434.00, 434.01, 434.1, 434.10, 434.11, 434.3, 434.4, 434.6, 434.7, 434.9, 434.90, 434.91, 435, 435.0, 435.1, 435.2, 435.3, 435.8, 435.9, 437.0, 437.1, 437.5, 437.6, 437.7, 437.8
Kidney cancer	C64, C64.0, C64.1, C64.2, C64.4, C64.5, C64.6, C64.8, C64.9, C65, C65.0, C65.1, C65.2, C65.9, D30.0, D30.00, D30.01, D30.02, D30.1, D30.10, D30.11, D30.12, D41.0, D41.00, D41.01, D41.02, D41.1, D41.10, D41.11, D41.12	189.0, 189.1, 189.5, 189.6, 209.24, 209.64, 223.0, 223.1, 236.91
Larynx cancer	C32, C32.0, C32.1, C32.2, C32.3, C32.8, C32.9, D02.0, D14.1, D38.0	161, 161.0, 161.1, 161.2, 161.3, 161.8, 161.9, 212.1, 231.0, 235.6
Late maternal deaths	O96, O96.0, O96.1, O96.9, O97, O97.0, O97.1, O97.9	
Leishmaniasis		
Leukemia	C91, C92, C93, C94, C95, C95.0, C95.00, C95.01, C95.02, C95.1, C95.10, C95.11, C95.12, C95.2, C95.4, C95.6, C95.7, C95.9, C95.90, C95.91, C95.92	204, 205, 206, 207, 208, 208.0, 208.00, 208.01, 208.02, 208.1, 208.10, 208.11, 208.12, 208.2, 208.20, 208.21, 208.22, 208.4, 208.7, 208.8, 208.80, 208.81, 208.82, 208.9, 208.90, 208.91, 208.92
Lip and oral cavity cancer	C00, C00.0, C00.1, C00.2, C00.3, C00.4, C00.5, C00.6, C00.8, C00.9, C01, C01.9, C02, C02.0, C02.1, C02.2, C02.3, C02.4, C02.8, C02.9, C03, C03.0, C03.1, C03.9, C04, C04.0, C04.1, C04.8, C04.9, C05, C05.0, C05.1, C05.2, C05.8, C05.9, C06, C06.0, C06.1, C06.2, C06.8, C06.80, C06.89, C06.9, C07, C07.0, C07.9, C08, C08.0, C08.1, C08.8, C08.9, D00.00, D00.01, D00.02, D00.03, D00.04, D00.05, D00.06, D00.07, D10.0, D10.1, D10.2, D10.3, D10.30, D10.39, D10.4, D10.5, D11, D11.0, D11.7, D11.9, D37.01, D37.02, D37.03, D37.030, D37.031, D37.032, D37.039, D37.04, D37.09	140, 140.0, 140.1, 140.2, 140.3, 140.4, 140.5, 140.6, 140.7, 140.8, 140.9, 141, 141.0, 141.1, 141.2, 141.3, 141.4, 141.5, 141.6, 141.8, 141.9, 142, 142.0, 142.1, 142.2, 142.3, 142.8, 142.9, 143, 143.0, 143.1, 143.8, 143.9, 144, 144.0, 144.1, 144.4, 144.8, 144.9, 145, 145.0, 145.1, 145.2, 145.3, 145.4, 145.5, 145.6, 145.8, 145.9, 210.0, 210.1, 210.2, 210.3, 210.4, 210.5, 210.6, 235.0
Liver cancer	C22, C22.0, C22.1, C22.3, C22.4, C22.5, C22.7, C22.8, D13.4	155, 155.0, 155.1, 155.3, 155.5, 155.9, 211.5

Table S4. List of infectious syndromes mapped to International Classification of Diseases (ICD) codes

Cause Name	ICD 10 Codes	ICD 9 Codes
Lower extremity peripheral arterial disease	I70.2, I70.20, I70.201, I70.202, I70.203, I70.208, I70.209, I70.21, I70.211, I70.212, I70.213, I70.218, I70.219, I70.22, I70.221, I70.222, I70.223, I70.228, I70.229, I70.23, I70.231, I70.232, I70.233, I70.234, I70.235, I70.238, I70.239, I70.24, I70.241, I70.242, I70.243, I70.244, I70.245, I70.248, I70.249, I70.25, I70.26, I70.261, I70.262, I70.263, I70.268, I70.269, I70.29, I70.291, I70.292, I70.293, I70.298, I70.299, I70.3, I70.30, I70.301, I70.302, I70.303, I70.308, I70.309, I70.31, I70.311, I70.312, I70.313, I70.318, I70.319, I70.32, I70.321, I70.322, I70.323, I70.328, I70.329, I70.33, I70.331, I70.332, I70.333, I70.334, I70.335, I70.338, I70.339, I70.34, I70.341, I70.342, I70.343, I70.344, I70.345, I70.348, I70.349, I70.35, I70.36, I70.361, I70.362, I70.363, I70.368, I70.369, I70.39, I70.391, I70.392, I70.393, I70.398, I70.399, I70.4, I70.40, I70.401, I70.402, I70.403, I70.408, I70.409, I70.41, I70.411, I70.412, I70.413, I70.418, I70.419, I70.42, I70.421, I70.422, I70.423, I70.428, I70.429, I70.43, I70.431, I70.432, I70.433, I70.434, I70.435, I70.438, I70.439, I70.44, I70.441, I70.442, I70.443, I70.444, I70.445, I70.448, I70.449, I70.45, I70.46, I70.461, I70.462, I70.463, I70.468, I70.469, I70.49, I70.491, I70.492, I70.493, I70.498, I70.499, I70.5, I70.50, I70.501, I70.502, I70.503, I70.508, I70.509, I70.51, I70.511, I70.512, I70.513, I70.518, I70.519, I70.52, I70.521, I70.522, I70.523, I70.528, I70.529, I70.53, I70.531, I70.532, I70.533, I70.534, I70.535, I70.538, I70.539, I70.54, I70.541, I70.542, I70.543, I70.544, I70.545, I70.548, I70.549, I70.55, I70.56, I70.561, I70.562, I70.563, I70.568, I70.569, I70.59, I70.591, I70.592, I70.593, I70.598, I70.599, I70.6, I70.60, I70.601, I70.602, I70.603, I70.608, I70.609, I70.61, I70.611, I70.612, I70.613, I70.618, I70.619, I70.62, I70.621, I70.622, I70.623, I70.628, I70.629, I70.63, I70.631, I70.632, I70.633, I70.634, I70.635, I70.638, I70.639, I70.64, I70.641, I70.642, I70.643, I70.644, I70.645, I70.648, I70.649, I70.65, I70.66, I70.661, I70.662, I70.663, I70.668, I70.669, I70.69, I70.691, I70.692, I70.693, I70.698, I70.699, I70.7, I70.70, I70.701, I70.702, I70.703, I70.708, I70.709, I70.71, I70.711, I70.712, I70.713, I70.718, I70.719, I70.72, I70.721, I70.722, I70.723, I70.728, I70.729, I70.73, I70.731, I70.732, I70.733, I70.734, I70.735, I70.738, I70.739, I70.74, I70.741, I70.742, I70.743, I70.744, I70.745, I70.748, I70.749, I70.75, I70.76, I70.761, I70.762, I70.763, I70.768, I70.769, I70.79, I70.791, I70.792, I70.793, I70.798, I70.799, I70.8, I73, I73.0, I73.00, I73.01, I73.1, I73.8, I73.81, I73.89, I73.9	440.2, 440.20, 440.21, 440.22, 440.23, 440.24, 440.29, 440.4, 443.0, 443.1, 443.2, 443.21, 443.22, 443.23, 443.24, 443.29, 443.8, 443.81, 443.82, 443.89, 443.9
Lower respiratory infections		
Malaria	B50, B50.0, B50.1, B50.8, B50.9, B51, B51.0, B51.8, B51.9, B52, B52.0, B52.1, B52.8, B52.9, B53, B53.0, B53.1, B53.3, B53.8	084, 084.0, 084.1, 084.2, 084.3, 084.4, 084.5, 084.6, 084.7, 084.8, 084.9
Malignant neoplasm of bone and articular cartilage	C40, C40.0, C40.00, C40.01, C40.02, C40.1, C40.10, C40.11, C40.12, C40.2, C40.20, C40.21, C40.22, C40.3, C40.30, C40.31, C40.32, C40.8, C40.80, C40.81, C40.82, C40.9, C40.90, C40.91, C40.92, C41, C41.0, C41.1, C41.2, C41.3, C41.4, C41.5, C41.6, C41.7, C41.8, C41.9	170, 170.0, 170.1, 170.2, 170.3, 170.4, 170.5, 170.6, 170.7, 170.8, 170.9
Malignant skin melanoma	C43, C43.0, C43.1, C43.10, C43.11, C43.12, C43.2, C43.20, C43.21, C43.22, C43.3, C43.30, C43.31, C43.39, C43.4, C43.5, C43.51, C43.52, C43.59, C43.6, C43.60, C43.61, C43.62, C43.7, C43.70, C43.71, C43.72, C43.8, C43.9, D03, D03.0, D03.1, D03.10, D03.11, D03.12, D03.2, D03.20, D03.21, D03.22, D03.3, D03.30, D03.39, D03.4, D03.5, D03.51, D03.52, D03.59, D03.6, D03.60, D03.61, D03.62, D03.7, D03.70, D03.71, D03.72, D03.8, D03.9, D22, D22.0, D22.1, D22.10, D22.11, D22.12, D22.2, D22.20, D22.21, D22.22, D22.3, D22.30, D22.39, D22.4, D22.5, D22.6, D22.60, D22.61, D22.62, D22.7, D22.70, D22.71, D22.72, D22.9, D23, D23.0, D23.1, D23.10, D23.11, D23.12, D23.2, D23.20, D23.21, D23.22, D23.3, D23.30, D23.39, D23.4, D23.5, D23.6, D23.60, D23.61, D23.62, D23.7, D23.70, D23.71, D23.72, D23.9, D48.5	172, 172.0, 172.1, 172.2, 172.3, 172.4, 172.5, 172.6, 172.7, 172.8, 172.9
Maternal abortion and miscarriage		
Maternal hemorrhage	O20, O20.0, O20.8, O20.9, O43.2, O43.21, O43.211, O43.212, O43.213, O43.219, O43.22, O43.221, O43.222, O43.223, O43.229, O43.23, O43.231, O43.232, O43.233, O43.239, O44, O44.0, O44.00, O44.01, O44.02, O44.03, O44.1, O44.10, O44.11, O44.12, O44.13, O45, O45.0, O45.00, O45.001, O45.002, O45.003, O45.009, O45.01, O45.011, O45.012, O45.013, O45.019, O45.02, O45.021, O45.022, O45.023, O45.029, O45.09, O45.091, O45.092, O45.093, O45.099, O45.8, O45.9, O45.90, O45.91, O45.92, O45.93, O46, O46.0, O46.00, O46.001, O46.002, O46.003, O46.009, O46.01, O46.011, O46.012, O46.013, O46.019, O46.02, O46.021, O46.022, O46.023, O46.029, O46.09, O46.091, O46.092, O46.093, O46.099, O46.8, O46.9, O46.90, O46.91, O46.92, O46.93, O62, O62.0, O62.1, O62.2, O62.3, O62.4, O62.8, O62.9, O67, O67.0, O67.8, O67.9, O70, O72, O72.0, O72.1, O72.2, O72.3	640, 640.0, 640.00, 640.01, 640.03, 640.8, 640.80, 640.81, 640.83, 640.9, 640.90, 640.91, 640.93, 641, 641.0, 641.00, 641.01, 641.03, 641.1, 641.10, 641.11, 641.13, 641.2, 641.20, 641.21, 641.23, 641.3, 641.30, 641.31, 641.33, 641.8, 641.80, 641.81, 641.83, 641.9, 641.90, 641.91, 641.93, 661, 661.0, 661.00, 661.01, 661.03, 661.1, 661.10, 661.11, 661.13, 661.2, 661.20, 661.21, 661.23, 661.3, 661.30, 661.31, 661.33, 661.4, 661.40, 661.41, 661.43, 661.9, 661.90, 661.91, 661.93, 665, 666, 666.0, 666.00, 666.02, 666.04, 666.1, 666.10, 666.12, 666.14, 666.2, 666.20, 666.22, 666.24, 666.3, 666.30, 666.32, 666.34, 666.9

Table S4. List of infectious syndromes mapped to International Classification of Diseases (ICD) codes		
**Cause Name**	**ICD 10 Codes**	**ICD 9 Codes**
Maternal hypertensive disorders	O10, O10.0, O10.01, O10.011, O10.012, O10.013, O10.019, O10.02, O10.03, O10.1, O10.11, O10.111, O10.112, O10.113, O10.119, O10.12, O10.13, O10.2, O10.21, O10.211, O10.212, O10.213, O10.219, O10.22, O10.23, O10.3, O10.31, O10.311, O10.312, O10.313, O10.319, O10.32, O10.33, O10.4, O10.41, O10.411, O10.412, O10.413, O10.419, O10.42, O10.43, O10.9, O10.91, O10.911, O10.912, O10.913, O10.919, O10.92, O10.93, O11, O11.1, O11.2, O11.3, O11.9, O12, O12.0, O12.00, O12.01, O12.02, O12.03, O12.1, O12.10, O12.11, O12.12, O12.13, O12.2, O12.20, O12.21, O12.22, O12.23, O13, O13.1, O13.2, O13.3, O13.9, O14, O14.0, O14.00, O14.02, O14.03, O14.1, O14.10, O14.12, O14.13, O14.2, O14.20, O14.22, O14.23, O14.9, O14.90, O14.92, O14.93, O15, O15.0, O15.00, O15.02, O15.03, O15.1, O15.2, O15.9, O16, O16.1, O16.2, O16.3, O16.9	642, 642.0, 642.00, 642.01, 642.02, 642.03, 642.04, 642.1, 642.10, 642.11, 642.12, 642.13, 642.14, 642.2, 642.20, 642.21, 642.22, 642.23, 642.24, 642.3, 642.30, 642.31, 642.32, 642.33, 642.34, 642.4, 642.40, 642.41, 642.42, 642.43, 642.44, 642.5, 642.50, 642.51, 642.52, 642.53, 642.54, 642.6, 642.60, 642.61, 642.62, 642.63, 642.64, 642.7, 642.70, 642.71, 642.72, 642.73, 642.74, 642.9, 642.90, 642.91, 642.92, 642.93, 642.94
Maternal obstructed labor and uterine rupture	O32, O32.0, O32.1, O32.2, O32.3, O32.4, O32.5, O32.6, O32.8, O32.9, O33, O33.0, O33.1, O33.2, O33.3, O33.4, O33.5, O33.6, O33.7, O33.8, O33.9, O64, O64.0, O64.1, O64.2, O64.3, O64.4, O64.5, O64.8, O64.9, O65, O65.0, O65.1, O65.2, O65.3, O65.4, O65.5, O65.8, O65.9, O66, O66.0, O66.1, O66.2, O66.3, O66.4, O66.40, O66.41, O66.5, O66.6, O66.8, O66.9, O71, O71.0, O71.00, O71.02, O71.03, O71.1, O71.2, O71.3, O71.4, O71.5, O71.6, O71.7, O71.8, O71.81, O71.82, O71.89, O71.9	652, 652.0, 652.00, 652.01, 652.03, 652.1, 652.10, 652.11, 652.13, 652.2, 652.20, 652.21, 652.23, 652.3, 652.30, 652.31, 652.33, 652.4, 652.40, 652.41, 652.43, 652.5, 652.50, 652.51, 652.53, 652.6, 652.60, 652.61, 652.63, 652.7, 652.70, 652.71, 652.73, 652.8, 652.80, 652.81, 652.83, 652.9, 652.90, 652.91, 652.93, 653, 653.0, 653.00, 653.01, 653.03, 653.1, 653.10, 653.11, 653.13, 653.2, 653.20, 653.21, 653.23, 653.3, 653.30, 653.31, 653.33, 653.4, 653.40, 653.41, 653.43, 653.5, 653.50, 653.51, 653.53, 653.6, 653.60, 653.61, 653.63, 653.7, 653.70, 653.71, 653.73, 653.8, 653.80, 653.81, 653.83, 653.9, 653.90, 653.91, 653.93, 660, 660.0, 660.00, 660.01, 660.03, 660.1, 660.10, 660.11, 660.13, 660.2, 660.20, 660.21, 660.23, 660.3, 660.30, 660.31, 660.33, 660.4, 660.40, 660.41, 660.43, 660.5, 660.50, 660.51, 660.53, 660.6, 660.60, 660.61, 660.63, 660.7, 660.70, 660.71, 660.73, 660.8, 660.80, 660.81, 660.83, 660.9, 660.90, 660.91, 660.93, 665.0, 665.00, 665.01, 665.03, 665.1, 665.10, 665.11, 665.2, 665.20, 665.22, 665.24, 665.3, 665.30, 665.31, 665.34
Maternal sepsis and other maternal infections	O23, O23.0, O23.00, O23.01, O23.02, O23.03, O23.1, O23.10, O23.11, O23.12, O23.13, O23.2, O23.20, O23.21, O23.22, O23.23, O23.3, O23.30, O23.31, O23.32, O23.33, O23.4, O23.40, O23.41, O23.42, O23.43, O23.5, O23.51, O23.511, O23.512, O23.513, O23.519, O23.52, O23.521, O23.522, O23.523, O23.529, O23.59, O23.591, O23.592, O23.593, O23.599, O23.9, O23.90, O23.91, O23.92, O23.93, O85, O85.0, O86, O86.0, O86.1, O86.11, O86.12, O86.13, O86.19, O86.2, O86.20, O86.21, O86.22, O86.29, O86.3, O86.4, O86.8, O86.81, O86.89, O91, O91.0, O91.01, O91.011, O91.012, O91.013, O91.019, O91.02, O91.03, O91.1, O91.11, O91.111, O91.112, O91.113, O91.119, O91.12, O91.13, O91.2, O91.21, O91.211, O91.212, O91.213, O91.219, O91.22, O91.23	659.3, 659.30, 659.31, 659.33, 670, 670.0, 670.00, 670.02, 670.04, 670.1, 670.10, 670.12, 670.14, 670.2, 670.20, 670.22, 670.24, 670.3, 670.30, 670.32, 670.34, 670.8, 670.80, 670.82, 670.84, 670.9
Measles	B05, B05.0, B05.1, B05.2, B05.3, B05.4, B05.8, B05.81, B05.89, B05.9	055, 055.0, 055.1, 055.2, 055.3, 055.5, 055.6, 055.7, 055.71, 055.79, 055.8, 055.9, 484.0
Meningitis		
Mesothelioma	C45, C45.0, C45.1, C45.2, C45.3, C45.4, C45.5, C45.6, C45.7, C45.8, C45.9	
Motor neuron disease	G12.2, G12.20, G12.21, G12.22, G12.29, G12.8, G12.9	335, 335.0, 335.1, 335.10, 335.11, 335.19, 335.2, 335.20, 335.21, 335.22, 335.23, 335.24, 335.29, 335.8, 335.9
Multiple myeloma	C88, C88.0, C88.1, C88.2, C88.3, C88.4, C88.7, C88.8, C88.9, C90, C90.0, C90.00, C90.01, C90.02, C90.1, C90.10, C90.11, C90.12, C90.2, C90.20, C90.21, C90.22, C90.3, C90.30, C90.31, C90.32, C90.4, C90.5, C90.6, C90.7, C90.8, C90.9	203, 203.0, 203.00, 203.01, 203.02, 203.1, 203.10, 203.11, 203.12, 203.8, 203.80, 203.81, 203.82, 203.9
Multiple sclerosis	G35, G35.0, G35.9	340, 340.0, 340.9
Myelodysplastic, myeloproliferative, and other hematopoietic neoplasms	D45, D45.0, D45.9, D46, D46.0, D46.1, D46.2, D46.20, D46.21, D46.22, D46.3, D46.4, D46.5, D46.7, D46.9, D47, D47.0, D47.1, D47.2, D47.3, D47.4, D47.5, D47.7, D47.9	238.4, 238.5, 238.6, 238.7, 238.71, 238.72, 238.73, 238.74, 238.75, 238.76, 238.77, 238.79, 238.8, 238.9
Myocarditis	B33.2, B33.20, B33.22, B33.23, B33.24, D86.85, I40, I40.0, I40.1, I40.8, I40.9, I41, I41.0, I41.1, I41.2, I41.8, I41.9, I51.4	422, 422.0, 422.4, 422.5, 422.9, 422.90, 422.91, 422.92, 422.93, 422.99
Nasopharynx cancer	C11, C11.0, C11.1, C11.2, C11.3, C11.8, C11.9, D00.08, D10.6, D37.05	147, 147.0, 147.1, 147.2, 147.3, 147.8, 147.9, 210.7, 210.8, 210.9

Table S4. List of infectious syndromes mapped to International Classification of Diseases (ICD) codes

Cause Name	ICD 10 Codes	ICD 9 Codes
Neonatal encephalopathy due to birth asphyxia and trauma	P01.7, P02, P02.0, P02.1, P02.2, P02.20, P02.29, P02.3, P02.4, P02.5, P02.6, P02.60, P02.69, P02.7, P02.8, P02.9, P03, P03.0, P03.1, P03.2, P03.3, P03.4, P03.5, P03.6, P03.8, P03.81, P03.810, P03.811, P03.819, P03.82, P03.89, P03.9, P10, P10.0, P10.1, P10.2, P10.3, P10.4, P10.8, P10.9, P11, P11.0, P11.1, P11.2, P11.3, P11.4, P11.5, P11.9, P12, P12.0, P12.1, P12.2, P12.3, P12.4, P12.8, P12.81, P12.89, P12.9, P13, P13.0, P13.1, P13.2, P13.3, P13.4, P13.8, P13.9, P14, P14.0, P14.1, P14.2, P14.3, P14.8, P14.9, P15, P15.0, P15.1, P15.2, P15.3, P15.4, P15.5, P15.6, P15.8, P15.9, P20, P20.0, P20.1, P20.9, P21, P21.0, P21.1, P21.9, P24, P24.0, P24.00, P24.01, P24.1, P24.10, P24.11, P24.2, P24.20, P24.21, P24.3, P24.30, P24.31, P24.8, P24.80, P24.81, P24.9, P90, P90.0, P91, P91.0, P91.1, P91.2, P91.3, P91.4, P91.5, P91.6, P91.60, P91.61, P91.62, P91.63, P91.8, P91.9	761.7, 761.8, 761.9, 762, 762.0, 762.1, 762.2, 762.3, 762.4, 762.5, 762.6, 762.7, 762.8, 762.9, 763, 763.0, 763.1, 763.2, 763.3, 763.4, 763.5, 763.6, 763.7, 763.8, 763.81, 763.82, 763.83, 763.84, 763.89, 763.9, 767, 767.0, 767.1, 767.11, 767.19, 767.2, 767.3, 767.4, 767.5, 767.6, 767.7, 767.8, 767.9, 768, 768.2, 768.3, 768.4, 768.5, 768.6, 768.7, 768.70, 768.71, 768.72, 768.73, 768.9, 770.1, 770.10, 770.11, 770.12, 770.13, 770.14, 770.15, 770.16, 770.17, 770.18, 779.0, 779.1, 779.2
Neonatal preterm birth	P01.0, P01.1, P05, P05.0, P05.00, P05.01, P05.02, P05.03, P05.04, P05.05, P05.06, P05.07, P05.08, P05.1, P05.10, P05.11, P05.12, P05.13, P05.14, P05.15, P05.16, P05.17, P05.18, P05.2, P05.9, P07, P07.0, P07.00, P07.01, P07.02, P07.03, P07.1, P07.10, P07.14, P07.15, P07.16, P07.17, P07.18, P07.2, P07.20, P07.21, P07.22, P07.23, P07.24, P07.25, P07.26, P07.3, P07.30, P07.31, P07.32, P07.33, P07.34, P07.35, P07.36, P07.37, P07.38, P07.39, P22, P22.0, P22.1, P22.8, P22.9, P25, P25.0, P25.1, P25.2, P25.3, P25.8, P26, P26.0, P26.1, P26.8, P26.9, P27, P27.0, P27.1, P27.8, P27.9, P28, P28.0, P28.1, P28.10, P28.11, P28.19, P28.2, P28.3, P28.4, P28.5, P28.8, P28.81, P28.89, P28.9, P52, P52.0, P52.1, P52.2, P52.21, P52.22, P52.3, P52.4, P52.5, P52.6, P52.8, P52.9, P61.2, P77, P77.0, P77.1, P77.2, P77.3, P77.9, P78.0, P78.1, P78.2, P78.3, P78.8, P78.81, P78.82, P78.83, P78.89, P78.9	761.0, 761.1, 764, 764.0, 764.00, 764.01, 764.02, 764.03, 764.04, 764.05, 764.06, 764.07, 764.08, 764.09, 764.1, 764.10, 764.11, 764.12, 764.13, 764.14, 764.15, 764.16, 764.17, 764.18, 764.19, 764.2, 764.20, 764.21, 764.22, 764.23, 764.24, 764.25, 764.26, 764.27, 764.28, 764.29, 764.8, 764.9, 764.90, 764.91, 764.92, 764.93, 764.94, 764.95, 764.96, 764.97, 764.98, 764.99, 765, 765.0, 765.00, 765.01, 765.02, 765.03, 765.04, 765.05, 765.06, 765.07, 765.08, 765.09, 765.1, 765.10, 765.11, 765.12, 765.13, 765.14, 765.15, 765.16, 765.17, 765.18, 765.19, 765.2, 765.20, 765.21, 765.22, 765.23, 765.24, 765.25, 765.26, 765.27, 765.28, 765.29, 765.5, 765.6, 765.9, 769, 769.0, 769.9, 770.2, 770.3, 770.4, 770.5, 770.6, 770.7, 770.8, 770.81, 770.82, 770.83, 770.84, 770.85, 770.86, 770.87, 770.88, 770.89, 770.9, 772.1, 772.10, 772.11, 772.12, 772.13, 772.14, 772.2, 772.3, 772.4, 772.5, 772.6, 772.8, 772.9, 776.6, 777.0, 777.1, 777.2, 777.3, 777.4, 777.5, 777.50, 777.51, 777.52, 777.53, 777.6, 777.7, 777.8, 777.9
Neonatal sepsis and other neonatal infections	P36, P36.0, P36.1, P36.10, P36.19, P36.2, P36.3, P36.30, P36.39, P36.4, P36.5, P36.8, P36.9, P38, P38.0, P38.1, P38.9, P39, P39.0, P39.1, P39.2, P39.3, P39.4, P39.8, P39.9	771.4, 771.5, 771.6, 771.7, 771.8, 771.81, 771.82, 771.83, 771.89, 771.9
Neural tube defects	Q00, Q00.0, Q00.1, Q00.2, Q01, Q01.0, Q01.1, Q01.2, Q01.8, Q01.9, Q05, Q05.0, Q05.1, Q05.2, Q05.3, Q05.4, Q05.5, Q05.6, Q05.7, Q05.8, Q05.9	740, 740.0, 740.1, 740.2, 740.4, 740.9, 741, 741.0, 741.01, 741.02, 741.03, 741.9, 741.91, 741.92, 741.93, 742.0
Neuroblastoma and other peripheral nervous cell tumors	C47, C47.0, C47.1, C47.10, C47.11, C47.12, C47.2, C47.20, C47.21, C47.22, C47.3, C47.4, C47.5, C47.6, C47.8, C47.9, C74.90	
Non-Hodgkin lymphoma		
Non-melanoma skin cancer		
Non-rheumatic valvular heart disease		

Table S4. List of infectious syndromes mapped to International Classification of Diseases (ICD) codes		
**Cause Name**	**ICD 10 Codes**	**ICD 9 Codes**
Non-venomous animal contact	W52.0, W52.1, W52.2, W52.3, W52.4, W52.5, W52.6, W52.7, W52.8, W52.9, W53, W53.0, W53.01, W53.09, W53.1, W53.11, W53.19, W53.2, W53.21, W53.29, W53.3, W53.8, W53.81, W53.89, W53.9, W54, W54.0, W54.1, W54.2, W54.3, W54.4, W54.5, W54.6, W54.7, W54.8, W54.9, W55, W55.0, W55.01, W55.03, W55.09, W55.1, W55.11, W55.12, W55.19, W55.2, W55.21, W55.22, W55.29, W55.3, W55.31, W55.32, W55.39, W55.4, W55.41, W55.42, W55.49, W55.5, W55.51, W55.52, W55.59, W55.6, W55.7, W55.8, W55.81, W55.82, W55.89, W55.9, W56, W56.0, W56.01, W56.02, W56.09, W56.1, W56.11, W56.12, W56.19, W56.2, W56.21, W56.22, W56.29, W56.3, W56.31, W56.32, W56.39, W56.4, W56.41, W56.42, W56.49, W56.5, W56.51, W56.52, W56.59, W56.6, W56.7, W56.8, W56.81, W56.82, W56.89, W56.9, W57, W57.0, W57.1, W57.2, W57.4, W57.5, W57.6, W57.7, W57.8, W57.9, W58, W58.0, W58.01, W58.02, W58.03, W58.09, W58.1, W58.11, W58.12, W58.13, W58.19, W58.4, W58.7, W58.8, W58.9, W59, W59.0, W59.01, W59.02, W59.09, W59.1, W59.11, W59.12, W59.13, W59.19, W59.2, W59.21, W59.22, W59.29, W59.4, W59.5, W59.6, W59.7, W59.8, W59.81, W59.82, W59.83, W59.89, W59.9, W60, W60.0, W60.1, W60.2, W60.4, W60.5, W60.6, W60.7, W60.8, W60.9, W61, W61.0, W61.01, W61.02, W61.09, W61.1, W61.11, W61.12, W61.19, W61.2, W61.21, W61.22, W61.29, W61.3, W61.32, W61.33, W61.39, W61.4, W61.42, W61.43, W61.49, W61.5, W61.51, W61.52, W61.59, W61.6, W61.61, W61.62, W61.69, W61.9, W61.91, W61.92, W61.99, W62, W62.0, W62.1, W62.9, W64, W64.0, W64.1, W64.2, W64.3, W64.4, W64.5, W64.6, W64.7, W64.8, W64.9	E906, E906.0, E906.00, E906.01, E906.02, E906.03, E906.04, E906.05, E906.06, E906.07, E906.08, E906.09, E906.1, E906.10, E906.11, E906.12, E906.13, E906.14, E906.15, E906.16, E906.17, E906.18, E906.19, E906.2, E906.20, E906.21, E906.22, E906.23, E906.24, E906.25, E906.26, E906.27, E906.28, E906.29, E906.3, E906.30, E906.31, E906.32, E906.33, E906.34, E906.35, E906.36, E906.37, E906.38, E906.39, E906.4, E906.40, E906.41, E906.42, E906.43, E906.44, E906.45, E906.46, E906.47, E906.48, E906.49, E906.5, E906.8, E906.80, E906.81, E906.82, E906.83, E906.84, E906.85, E906.86, E906.87, E906.88, E906.89, E906.9, E906.90, E906.91, E906.92, E906.93, E906.94, E906.95, E906.96, E906.97, E906.98, E906.99
Opioid use disorders	F11, F11.0, F11.1, F11.10, F11.12, F11.120, F11.121, F11.122, F11.129, F11.14, F11.15, F11.150, F11.151, F11.159, F11.18, F11.181, F11.182, F11.188, F11.19, F11.2, F11.20, F11.21, F11.22, F11.220, F11.221, F11.222, F11.229, F11.23, F11.24, F11.25, F11.250, F11.251, F11.259, F11.28, F11.281, F11.282, F11.288, F11.29, F11.3, F11.4, F11.5, F11.6, F11.7, F11.8, F11.9, F11.90, F11.92, F11.920, F11.921, F11.922, F11.929, F11.93, F11.94, F11.95, F11.950, F11.951, F11.959, F11.98, F11.981, F11.982, F11.988, F11.99, P96.1, R78.1	304.0, 304.00, 304.01, 304.02, 304.03, 305.5, 305.50, 305.51, 305.52, 305.53, E850.0, E850.00, E850.01, E850.02, E850.03, E850.04, E850.05, E850.06, E850.07, E850.08, E850.09, E850.1, E850.10, E850.11, E850.12, E850.13, E850.14, E850.15, E850.16, E850.17, E850.18, E850.19, E850.2, E850.20, E850.21, E850.22, E850.23, E850.24, E850.25, E850.26, E850.27, E850.28, E850.29
Orofacial clefts	Q35, Q35.0, Q35.1, Q35.2, Q35.3, Q35.4, Q35.5, Q35.6, Q35.7, Q35.8, Q35.9, Q36, Q37, Q37.0, Q37.1, Q37.2, Q37.3, Q37.4, Q37.5, Q37.8, Q37.9	749, 749.0, 749.00, 749.01, 749.02, 749.03, 749.04, 749.2, 749.20, 749.21, 749.22, 749.23, 749.24, 749.25, 749.6, 749.9
Other benign and in situ neoplasms	N60, N60.0, N60.01, N60.02, N60.09, N60.1, N60.11, N60.12, N60.19, N60.2, N60.21, N60.22, N60.29, N60.3, N60.31, N60.32, N60.39, N60.4, N60.41, N60.42, N60.49, N60.8, N60.81, N60.82, N60.89, N60.9, N60.91, N60.92, N60.99	
Other cardiomyopathy	I42.1, I42.2, I42.3, I42.4, I42.5, I42.7, I42.8, I43, I43.0, I43.1, I43.2, I43.8, I43.9	425.0, 425.1, 425.11, 425.18, 425.2, 425.3, 425.4, 425.7, 425.8, 429.0
Other cardiovascular and circulatory diseases	I27.2, I28, I28.0, I28.1, I28.8, I28.9, I30, I30.0, I30.1, I30.8, I30.9, I31, I31.0, I31.1, I31.8, I31.9, I32, I32.0, I32.1, I32.8, I47, I47.0, I47.1, I47.2, I47.9, I51.0, I51.1, I51.2, I51.3, I68.0, I72, I72.0, I72.1, I72.2, I72.3, I72.4, I72.5, I72.8, I72.9, I77, I77.0, I77.1, I77.2, I77.3, I77.4, I77.5, I77.6, I77.7, I77.71, I77.72, I77.73, I77.74, I77.79, I77.8, I77.81, I77.810, I77.811, I77.812, I77.819, I77.89, I77.9, I78, I78.0, I78.1, I78.8, I78.9, I79, I79.0, I79.1, I79.2, I79.8, I86, I86.0, I86.4, I86.8, I87, I87.1, I87.2, I87.3, I87.30, I87.301, I87.302, I87.303, I87.309, I87.31, I87.311, I87.312, I87.313, I87.319, I87.32, I87.321, I87.322, I87.323, I87.329, I87.33, I87.331, I87.332, I87.333, I87.339, I87.39, I87.391, I87.392, I87.393, I87.399, I87.8, I87.9, I88, I88.0, I88.1, I88.8, I88.9, I89, I89.0, I89.9, I98	417, 417.0, 417.1, 417.8, 417.9, 420, 420.0, 420.1, 420.9, 420.90, 420.91, 420.99, 423, 423.1, 423.2, 423.3, 423.7, 423.8, 423.9, 427.0, 427.1, 427.2, 427.6, 427.60, 427.61, 427.69, 427.8, 427.81, 427.89, 442, 442.0, 442.1, 442.2, 442.3, 442.4, 442.8, 442.81, 442.82, 442.83, 442.84, 442.89, 442.9, 443, 447, 447.0, 447.1, 447.2, 447.3, 447.4, 447.5, 447.6, 447.7, 447.70, 447.71, 447.72, 447.73, 447.8, 447.9, 448, 448.0, 448.1, 448.9, 449, 450, 456, 456.3, 456.8, 456.9, 457, 457.1, 457.8, 457.9, 459, 459.2, 459.3, 459.30, 459.31, 459.32, 459.33, 459.39
Other chromosomal abnormalities	Q87, Q87.0, Q87.1, Q87.2, Q87.3, Q87.4, Q87.40, Q87.41, Q87.410, Q87.418, Q87.42, Q87.43, Q87.5, Q87.8, Q87.81, Q87.89, Q91, Q91.0, Q91.1, Q91.2, Q91.3, Q91.4, Q91.5, Q91.6, Q91.7, Q92, Q92.0, Q92.1, Q92.2, Q92.3, Q92.4, Q92.5, Q92.6, Q92.61, Q92.62, Q92.7, Q92.8, Q92.9, Q93, Q93.0, Q93.1, Q93.2, Q93.3, Q93.4, Q93.5, Q93.6, Q93.7, Q93.8, Q93.81, Q93.88, Q93.89, Q93.9, Q95, Q95.0, Q95.1, Q95.2, Q95.3, Q95.4, Q95.5, Q95.8, Q95.9, Q97, Q97.0, Q97.1, Q97.2, Q97.3, Q97.8, Q97.9, Q99, Q99.0, Q99.1, Q99.146, Q99.2, Q99.8	758, 758.1, 758.2, 758.3, 758.33, 758.39, 758.4, 758.5, 758.6, 758.8, 758.81, 758.89, 758.9

Table S4. List of infectious syndromes mapped to International Classification of Diseases (ICD) codes

Cause Name	ICD 10 Codes	ICD 9 Codes
Other chronic respiratory diseases	J34.1, J34.2, J34.3, J34.8, J34.81, J34.89, J34.9, J38, J38.0, J38.00, J38.01, J38.02, J38.1, J38.2, J38.3, J38.4, J38.5, J38.6, J38.7, J39, J39.2, J39.3, J39.8, J39.9, J66, J66.0, J66.1, J66.2, J66.8, J67, J67.0, J67.1, J67.2, J67.3, J67.4, J67.5, J67.6, J67.7, J67.8, J67.9, J68, J68.0, J68.1, J68.2, J68.3, J68.4, J68.8, J68.9, J70, J70.8, J70.9, J82, J91, J91.8, J91.9, J92, J92.9	470, 470.9, 478.3, 478.30, 478.31, 478.32, 478.33, 478.34, 478.4, 478.5, 478.6, 478.7, 478.70, 478.71, 478.74, 478.75, 478.79, 478.8, 478.9, 479, 495, 495.0, 495.1, 495.2, 495.3, 495.4, 495.5, 495.6, 495.7, 495.8, 495.9, 506, 506.0, 506.1, 506.2, 506.3, 506.4, 506.9, 508, 508.0, 508.1, 508.2, 508.8, 508.9, 509, 517, 517.0, 517.1, 517.2, 517.3, 517.8, 518.6, 518.9, 519.1, 519.11, 519.19, 519.2, 519.3, 519.4
Other congenital birth defects	G71.2, Q02, Q02.0, Q02.8, Q02.9, Q03, Q03.0, Q03.1, Q03.8, Q03.9, Q04, Q04.0, Q04.1, Q04.2, Q04.3, Q04.4, Q04.5, Q04.6, Q04.8, Q04.9, Q06, Q06.0, Q06.1, Q06.2, Q06.3, Q06.4, Q06.8, Q06.9, Q07, Q07.0, Q07.00, Q07.01, Q07.02, Q07.03, Q07.8, Q07.9, Q10.4, Q10.5, Q10.6, Q10.7, Q11, Q11.0, Q11.1, Q11.2, Q11.3, Q12, Q12.0, Q12.1, Q12.2, Q12.3, Q12.4, Q12.8, Q12.9, Q13, Q13.0, Q13.1, Q13.2, Q13.3, Q13.4, Q13.5, Q13.8, Q13.81, Q13.89, Q13.9, Q14, Q14.0, Q14.1, Q14.2, Q14.3, Q14.8, Q14.9, Q15, Q15.0, Q15.8, Q15.9, Q16, Q16.0, Q16.1, Q16.2, Q16.3, Q16.4, Q16.5, Q16.9, Q17, Q17.0, Q17.1, Q17.2, Q17.3, Q17.4, Q17.5, Q17.8, Q17.9, Q18, Q18.0, Q18.1, Q18.2, Q18.3, Q18.4, Q18.5, Q18.6, Q18.7, Q18.8, Q18.9, Q30, Q30.0, Q30.1, Q30.2, Q30.3, Q30.8, Q30.9, Q31, Q31.0, Q31.1, Q31.2, Q31.3, Q31.4, Q31.5, Q31.8, Q31.9, Q32, Q32.0, Q32.1, Q32.2, Q32.3, Q32.4, Q32.9, Q33, Q33.0, Q33.1, Q33.2, Q33.3, Q33.4, Q33.5, Q33.6, Q33.8, Q33.9, Q34, Q34.0, Q34.1, Q34.8, Q34.9, Q80, Q80.0, Q80.1, Q80.2, Q80.3, Q80.4, Q80.8, Q80.9, Q81, Q81.0, Q81.1, Q81.2, Q81.8, Q81.9, Q82, Q82.0, Q82.1, Q82.2, Q82.3, Q82.4, Q82.5, Q82.8, Q82.9, Q83, Q83.0, Q83.1, Q83.2, Q83.3, Q83.8, Q83.9, Q84, Q84.0, Q84.1, Q84.2, Q84.3, Q84.4, Q84.5, Q84.6, Q84.8, Q84.9, Q85, Q85.0, Q85.00, Q85.01, Q85.02, Q85.03, Q85.09, Q85.1, Q85.8, Q85.9, Q86, Q86.1, Q86.2, Q86.8, Q89, Q89.0, Q89.01, Q89.09, Q89.1, Q89.2, Q89.3, Q89.4, Q89.7, Q89.8	742, 742.1, 742.2, 742.3, 742.4, 742.51, 742.53, 742.59, 742.8, 742.9, 743, 743.0, 743.00, 743.03, 743.06, 743.1, 743.10, 743.11, 743.12, 743.2, 743.20, 743.21, 743.22, 743.3, 743.30, 743.31, 743.32, 743.33, 743.34, 743.35, 743.36, 743.37, 743.39, 743.4, 743.41, 743.42, 743.43, 743.44, 743.45, 743.46, 743.47, 743.48, 743.49, 743.5, 743.51, 743.52, 743.53, 743.54, 743.55, 743.56, 743.57, 743.58, 743.59, 743.6, 743.61, 743.62, 743.63, 743.64, 743.65, 743.66, 743.69, 743.8, 743.9, 744, 744.0, 744.00, 744.01, 744.02, 744.03, 744.04, 744.05, 744.09, 744.1, 744.2, 744.21, 744.22, 744.23, 744.24, 744.29, 744.3, 744.4, 744.41, 744.42, 744.43, 744.46, 744.47, 744.49, 744.5, 744.8, 744.81, 744.82, 744.83, 744.84, 744.89, 744.9, 748, 748.0, 748.1, 748.2, 748.3, 748.4, 748.5, 748.6, 748.60, 748.61, 748.69, 748.7, 748.8, 748.9, 757, 757.0, 757.1, 757.2, 757.3, 757.31, 757.32, 757.33, 757.39, 757.4, 757.5, 757.6, 757.8, 757.9, 759.0, 759.1, 759.2, 759.3, 759.4, 759.5, 759.6, 759.7, 759.8, 759.81, 759.82, 759.83, 759.89
Other digestive diseases	K31, K31.0, K31.1, K31.2, K31.3, K31.4, K31.5, K31.6, K31.7, K31.8, K31.81, K31.811, K31.819, K31.82, K31.83, K31.84, K31.89, K38, K38.0, K38.1, K38.2, K58, K58.0, K59, K59.1, K59.2, K59.4, K59.8, K59.9, K66.8, K67, K68, K77, K90, K90.1, K90.2, K90.3, K90.4, K90.8, K90.81, K90.89, K90.9, K92.8, K92.81, K92.89, K93.8	536, 536.0, 536.1, 537, 537.0, 537.1, 537.2, 537.3, 537.4, 537.5, 537.6, 537.8, 537.81, 537.82, 537.83, 537.84, 538, 543, 543.0, 543.9, 564.1, 564.5, 569.7, 569.71, 579, 579.1, 579.2, 579.8, 579.9
Other direct maternal disorders	C58, C58.0, N98, N98.0, N98.1, N98.2, N98.3, N98.8, N98.9, O09, O09.0, O09.00, O09.01, O09.02, O09.03, O09.1, O09.10, O09.11, O09.12, O09.13, O09.2, O09.21, O09.211, O09.212, O09.213, O09.219, O09.29, O09.291, O09.292, O09.293, O09.299, O09.3, O09.30, O09.31, O09.32, O09.33, O09.4, O09.40, O09.41, O09.42, O09.43, O09.5, O09.51, O09.511, O09.512, O09.513, O09.519, O09.52, O09.521, O09.522, O09.523, O09.529, O09.6, O09.61, O09.611, O09.612, O09.613, O09.619, O09.62, O09.621, O09.622, O09.623, O09.629, O09.7, O09.70, O09.71, O09.72, O09.73, O09.8, O09.81, O09.811, O09.812, O09.813, O09.819, O09.82, O09.821, O09.822, O09.823, O09.829, O09.89, O09.891, O09.892, O09.893, O09.899, O09.9, O09.90, O09.91, O09.92, O09.93, O21, O21.0, O21.1, O21.2, O21.8, O21.9, O22, O22.0, O22.00, O22.01, O22.02, O22.03, O22.1, O22.10, O22.11, O22.12, O22.13, O22.2, O22.20, O22.21, O22.22, O22.23, O22.3, O22.30, O22.31, O22.32, O22.33, O22.4, O22.40, O22.41, O22.42, O22.43, O22.5, O22.50, O22.51, O22.52, O22.53, O22.8, O22.9, O22.90, O22.91, O22.92, O22.93, O26, O26.0, O26.00, O26.01, O26.02, O26.03, O26.1, O26.10, O26.11, O26.12, O26.13, O26.2, O26.20, O26.21, O26.22, O26.23, O26.3, O26.30, O26.31, O26.32, O26.33, O26.4, O26.40, O26.41, O26.42, O26.43, O26.5, O26.50, O26.51, O26.52, O26.53, O26.6, O26.61, O26.611, O26.612, O26.613, O26.619, O26.62, O26.63, O26.7, O26.71, O26.711, O26.712, O26.713, O26.719, O26.72, O26.73, O26.8, O26.81, O26.811, O26.812, O26.813, O26.819, O26.82, O26.821, O26.822, O26.823, O26.829, O26.83, O26.831, O26.832, O26.833, O26.839, O26.84, O26.841, O26.842, O26.843, O26.849, O26.85, O26.851, O26.852, O26.853, O26.859, O26.86, O26.87, O26.872, O26.873, O26.879, O26.89, O26.891, O26.892, O26.893, O26.899, O26.9, O26.90, O26.91, O26.92, O26.93, O28, O28.0, O28.1, O28.2, O28.3, O28.4, O28.5, O28.8, O28.9, O29, O29.0, O29.01, O29.011, O29.012, O29.013, O29.019, O29.02, O29.021, O29.022, O29.023, O29.029, O29.09, O29.091, O29.092, O29.093, O29.099, O29.1, O29.11, O29.111, O29.112, O29.113, O29.119, O29.12, O29.121, O29.122, O29.123, O29.129, O29.19, O29.191, O29.192, O29.193, O29.199, O29.2, O29.21, O29.211, O29.212, O29.213, O29.219, O29.29, O29.291, O29.292, O29.293, O29.299, O29.3, O29.4, O29.40, O29.41, O29.42, O29.43, O29.5, O29.6, O29.60, O29.61, O29.62, O29.63, O29.8, O29.9, O29.90, O29.91, O29.92, O29.93, O30, O30.0, O30.00, O30.001, O30.002	181, 181.0, 181.9, 643, 643.0, 643.00, 643.01, 643.03, 643.1, 643.10, 643.11, 643.13, 643.2, 643.20, 643.21, 643.23, 643.8, 643.80, 643.81, 643.83, 643.9, 643.90, 643.91, 643.93, 644, 644.0, 644.00, 644.03, 644.1, 644.10, 644.13, 644.2, 644.20, 644.21, 645, 645.0, 645.01, 645.03, 645.1, 645.10, 645.11, 645.13, 645.2, 645.20, 645.21, 645.23, 650, 650.0, 650.5, 651, 651.0, 651.00, 651.01, 651.03, 651.1, 651.10, 651.11, 651.13, 651.2, 651.20, 651.21, 651.23, 651.3, 651.30, 651.31, 651.33, 651.4, 651.40, 651.41, 651.43, 651.5, 651.50, 651.51, 651.53, 651.6, 651.60, 651.61, 651.63, 651.7, 651.70, 651.71, 651.73, 651.8, 651.80, 651.81, 651.83, 651.9, 651.90, 651.91, 651.93, 654, 654.0, 654.00, 654.01, 654.02, 654.03, 654.04, 654.1, 654.10, 654.11, 654.12, 654.13, 654.14, 654.2, 654.20, 654.21, 654.23, 654.24, 654.3, 654.30, 654.31, 654.32, 654.33, 654.34, 654.4, 654.40, 654.41, 654.42, 654.43, 654.44, 654.5, 654.50, 654.51, 654.52, 654.53, 654.54, 654.6, 654.60, 654.61, 654.62, 654.63, 654.64, 654.7, 654.70, 654.71, 654.72, 654.73, 654.74, 654.8, 654.80, 654.81, 654.82, 654.83, 654.84, 654.9, 654.90, 654.91, 654.92, 654.93, 654.94, 655, 655.0, 655.00, 655.01, 655.03, 655.1, 655.10, 655.11, 655.13, 655.2, 655.20, 655.21, 655.23, 655.3, 655.30, 655.31, 655.33, 655.4, 655.40, 655.41, 655.43, 655.5, 655.50, 655.51, 655.53, 655.6, 655.60, 655.61, 655.63, 655.7, 655.70, 655.71, 655.73, 655.8, 655.80, 655.81, 655.83, 655.9, 655.90, 655.91, 655.93, 656, 656.0, 656.00, 656.01, 656.03, 656.1, 656.10, 656.11, 656.13, 656.2, 656.20, 656.21, 656.23, 656.3, 656.30, 656.31, 656.33, 656.4, 656.40, 656.41, 656.43, 656.5, 656.50, 656.51, 656.53, 656.6, 656.60, 656.61, 656.63, 656.7, 656.70, 656.71, 656.73, 656.8, 656.80, 656.81, 656.83, 656.9, 656.90, 656.91, 656.93, 657, 657.0, 657.00, 657.01, 657.03, 657.1, 658, 658.0, 658.00, 658.01, 658.03, 658.1, 658.10, 658.11, 658.13, 658.2, 658.20, 658.21, 658.23, 658.3, 658.30, 658.31, 658.33, 658.4, 658.40, 658.41, 658.43, 658.8, 658.80, 658.81, 658.83, 658.9, 658.90, 658.91, 658.93, 659, 659.0, 659.00, 659.01, 659.03, 659.1, 659.10, 659.11, 659.13, 659.2, 659.20, 659.21, 659.23, 659.4, 659.40, 659.41, 659.43, 659.5, 659.50, 659.51, 659.53, 659.6, 659.60, 659.61, 659.63, 659.7, 659.70, 659.71, 659.73, 659.8, 659.80, 659.81, 659.83, 659.9, 659.90, 659.91, 659.93, 662, 662.0, 662.00, 662.01, 662.03, 662.1, 662.10, 662.11, 662.13, 662.2, 662.20, 662.21, 662.23, 662.3, 662.30, 662.31, 662.33, 662.8, 663, 663.0, 663.00, 663.01, 663.03, 663.1, 663.10, 663.11, 663.13, 663.2, 663.20, 663.21, 663.23, 663.3, 663.30, 663.31, 663.33, 663.4, 663.40, 663.41, 663.43, 663.5, 663.50, 663.51, 663.53, 663.6, 663.60

Table S4. List of infectious syndromes mapped to International Classification of Diseases (ICD) codes

Cause Name	ICD 10 Codes	ICD 9 Codes
Other drug use disorders	F13, F13.0, F13.1, F13.10, F13.12, F13.120, F13.121, F13.129, F13.14, F13.15, F13.150, F13.151, F13.159, F13.18, F13.180, F13.181, F13.182, F13.188, F13.19, F13.2, F13.20, F13.21, F13.22, F13.220, F13.221, F13.229, F13.23, F13.230, F13.231, F13.232, F13.239, F13.24, F13.25, F13.250, F13.251, F13.259, F13.26, F13.27, F13.28, F13.280, F13.281, F13.282, F13.288, F13.29, F13.3, F13.4, F13.5, F13.6, F13.7, F13.8, F13.9, F13.90, F13.92, F13.920, F13.921, F13.929, F13.93, F13.930, F13.931, F13.932, F13.939, F13.94, F13.95, F13.950, F13.951, F13.959, F13.96, F13.97, F13.98, F13.980, F13.981, F13.982, F13.988, F13.99, F16, F16.0, F16.1, F16.10, F16.12, F16.120, F16.121, F16.122, F16.129, F16.14, F16.15, F16.150, F16.151, F16.159, F16.18, F16.180, F16.183, F16.188, F16.19, F16.2, F16.20, F16.21, F16.22, F16.220, F16.221, F16.229, F16.24, F16.25, F16.250, F16.251, F16.259, F16.28, F16.280, F16.283, F16.288, F16.29, F16.3, F16.4, F16.5, F16.6, F16.7, F16.8, F16.9, F16.90, F16.92, F16.920, F16.921, F16.929, F16.94, F16.95, F16.950, F16.951, F16.959, F16.98, F16.980, F16.983, F16.988, F16.99, F18, F18.0, F18.1, F18.10, F18.12, F18.120, F18.121, F18.129, F18.14, F18.15, F18.150, F18.151, F18.159, F18.17, F18.18, F18.180, F18.188, F18.19, F18.2, F18.20, F18.21, F18.22, F18.220, F18.221, F18.229, F18.24, F18.25, F18.250, F18.251, F18.259, F18.27, F18.28, F18.280, F18.288, F18.29, F18.3, F18.4, F18.5, F18.6, F18.7, F18.8, F18.9, F18.90, F18.92, F18.920, F18.921, F18.929, F18.94, F18.95, F18.950, F18.951, F18.959, F18.97, F18.98, F18.980, F18.988, F18.99, P04.4, P04.41, P04.49, R78.3, R78.4, R78.5	292, 292.0, 292.1, 292.11, 292.12, 292.2, 292.8, 292.81, 292.82, 292.83, 292.84, 292.85, 292.89, 292.9, 304.1, 304.10, 304.11, 304.12, 304.13, 304.5, 304.50, 304.51, 304.52, 304.53, 304.6, 304.60, 304.61, 304.62, 304.63, 304.7, 304.70, 304.71, 304.72, 304.73, 304.8, 304.80, 304.81, 304.82, 304.83, 305.3, 305.30, 305.31, 305.32, 305.33, 305.4, 305.40, 305.41, 305.42, 305.43, 305.8, 305.80, 305.81, 305.82, 305.83, 760.7, 760.70, 760.71, 760.72, 760.73, 760.74, 760.75, 760.76, 760.77, 760.78, 760.79
Other endocrine, metabolic, blood, and immune disorders	D66, D66.0, D67, D68.0, D68.1, D68.2, D68.3, D68.31, D68.311, D68.312, D68.318, D68.32, D68.4, D68.5, D68.51, D68.52, D68.59, D68.6, D68.61, D68.62, D68.69, D68.8, D68.9, D69, D69.0, D69.1, D69.2, D69.3, D69.4, D69.41, D69.42, D69.49, D69.6, D69.8, D70, D70.0, D70.4, D70.8, D70.9, D71, D72, D72.0, D72.1, D72.8, D72.81, D72.810, D72.818, D72.819, D72.82, D72.820, D72.821, D72.822, D72.823, D72.824, D72.825, D72.828, D72.829, D72.89, D72.9, D73, D73.0, D73.1, D73.2, D73.3, D73.4, D73.5, D73.8, D73.81, D73.89, D73.9, D74, D74.0, D74.8, D74.9, D75, D75.0, D75.1, D75.2, D75.8, D75.81, D75.89, D76, D76.0, D76.1, D76.2, D76.3, D77, D86.8, D86.82, D86.83, D86.84, D86.86, D86.87, D89, D89.0, D89.1, D89.2, E07.0, E16.1, E16.2, E16.3, E16.4, E16.8, E16.9, E20, E20.0, E20.1, E20.8, E20.9, E21, E21.0, E21.1, E21.2, E21.3, E21.4, E21.5, E22, E22.0, E22.1, E22.2, E22.8, E22.9, E23, E23.0, E23.2, E23.3, E23.6, E23.7, E24, E24.0, E24.1, E24.3, E24.8, E24.9, E25, E25.0, E25.8, E25.9, E26, E26.0, E26.01, E26.02, E26.09, E26.1, E26.8, E26.81, E26.89, E26.9, E27, E27.0, E27.1, E27.2, E27.4, E27.40, E27.49, E27.5, E27.8, E27.9, E28, E28.0, E28.1, E28.3, E28.31, E28.310, E28.319, E28.39, E28.8, E28.9, E29, E29.0, E29.1, E29.8, E29.9, E30, E30.0, E30.1, E30.8, E30.9, E31, E31.0, E31.1, E31.2, E31.20, E31.21, E31.22, E31.23, E31.8, E31.9, E32, E32.0, E32.1, E32.8, E32.9, E33, E34, E34.1, E34.2, E34.3, E34.4, E34.5, E34.50, E34.51, E34.52, E34.8, E67, E67.0, E67.1, E67.2, E67.3, E67.8, E68, E70, E70.0, E70.1, E70.2, E70.20, E70.21, E70.29, E70.3, E70.30, E70.31, E70.310, E70.311, E70.318, E70.319, E70.32, E70.320, E70.321, E70.328, E70.329, E70.33, E70.330, E70.331, E70.338, E70.339, E70.39, E70.4, E70.40, E70.41, E70.49, E70.5, E70.8, E70.9, E71, E71.0, E71.1, E71.11, E71.110, E71.113, E71.118, E71.12, E71.120, E71.121, E71.128, E71.19, E71.2, E71.3, E71.30, E71.31, E71.310, E71.311, E71.312, E71.313, E71.314, E71.318, E71.32, E71.39, E71.4, E71.40, E71.41, E71.42, E71.44, E71.440, E71.448, E71.5, E71.50, E71.51, E71.510, E71.511, E71.518, E71.52, E71.520, E71.521, E71.522, E71.528, E71.529, E71.53, E71.54, E71.540, E71.541, E71.542, E71.548, E72, E72.0, E72.00, E72.01, E72.02, E72.03, E72.04, E72.09, E72.1, E72.10, E72.11, E72.12, E72.19, E72.2, E72.20, E72.21, E72.22, E72.23, E72.29, E72.3, E72.4, E72.5, E72.50, E72.51, E72.52, E72.53, E72.59, E72.8, E72.9, E73, E73.0, E73.1, E73.8, E73.9, E74, E74.0, E74.00, E74.01, E74.02, E74.03, E74.04, E74.09, E74.1, E74.10	246, 246.0, 246.1, 246.2, 246.3, 246.8, 246.9, 251, 251.0, 251.1, 251.2, 251.4, 251.5, 251.8, 251.9, 252, 252.0, 252.00, 252.01, 252.02, 252.08, 252.1, 252.8, 252.9, 253, 253.0, 253.1, 253.2, 253.3, 253.4, 253.5, 253.6, 253.8, 253.9, 254, 254.0, 254.1, 254.4, 254.5, 254.8, 254.9, 255, 255.0, 255.1, 255.10, 255.11, 255.12, 255.13, 255.14, 255.2, 255.3, 255.4, 255.41, 255.42, 255.5, 255.6, 255.8, 255.9, 256, 256.0, 256.1, 256.2, 256.3, 256.31, 256.39, 256.8, 256.9, 257, 257.0, 257.1, 257.2, 257.8, 257.9, 258, 258.0, 258.01, 258.02, 258.03, 258.1, 258.8, 258.9, 259, 259.0, 259.1, 259.3, 259.4, 259.5, 259.50, 259.51, 259.52, 259.8, 259.9, 270, 270.0, 270.1, 270.2, 270.3, 270.4, 270.5, 270.6, 270.7, 270.8, 270.9, 271, 271.0, 271.1, 271.2, 271.3, 271.4, 271.5, 271.8, 271.9, 273, 273.0, 273.1, 273.2, 273.3, 273.4, 273.7, 273.8, 273.9, 275, 275.0, 275.01, 275.02, 275.03, 275.09, 275.1, 275.2, 275.3, 275.4, 275.40, 275.41, 275.42, 275.49, 275.5, 275.8, 275.9, 276, 277, 277.1, 277.2, 277.4, 277.5, 277.6, 277.7, 277.8, 277.81, 277.82, 277.83, 277.84, 277.85, 277.86, 277.87, 277.88, 277.89, 277.9, 278.2, 278.3, 278.4, 278.5, 278.8, 286, 286.0, 286.1, 286.2, 286.3, 286.4, 286.5, 286.7, 286.9, 287, 287.0, 287.1, 287.2, 287.3, 287.31, 287.32, 287.39, 287.4, 287.5, 287.8, 287.9, 288, 288.0, 288.1, 288.2, 288.3, 288.8, 288.9, 289, 289.0, 289.4, 289.5, 289.51, 289.52, 289.59, 289.6, 289.7

Table S4. List of infectious syndromes mapped to International Classification of Diseases (ICD) codes

Cause Name	ICD 10 Codes	ICD 9 Codes
Other exposure to mechanical forces	W20, W20.0, W20.1, W20.2, W20.3, W20.4, W20.5, W20.6, W20.7, W20.8, W20.9, W21, W21.0, W21.00, W21.01, W21.02, W21.03, W21.04, W21.05, W21.06, W21.07, W21.09, W21.1, W21.11, W21.12, W21.13, W21.19, W21.2, W21.21, W21.210, W21.211, W21.22, W21.220, W21.221, W21.3, W21.31, W21.32, W21.39, W21.4, W21.5, W21.6, W21.7, W21.8, W21.81, W21.89, W21.9, W22, W22.0, W22.01, W22.02, W22.03, W22.04, W22.041, W22.042, W22.09, W22.1, W22.10, W22.11, W22.12, W22.19, W22.2, W22.3, W22.4, W22.5, W22.6, W22.7, W22.8, W22.9, W23, W23.0, W23.1, W23.2, W23.3, W23.4, W23.5, W23.6, W23.7, W23.8, W23.9, W24, W24.0, W24.1, W24.2, W24.3, W24.4, W24.5, W24.6, W24.7, W24.8, W24.9, W25, W25.0, W25.1, W25.2, W25.3, W25.4, W25.5, W25.6, W25.7, W25.8, W25.9, W26, W26.0, W26.1, W26.2, W26.3, W26.4, W26.5, W26.6, W26.7, W26.8, W26.9, W27, W27.0, W27.1, W27.2, W27.3, W27.4, W27.5, W27.6, W27.7, W27.8, W27.9, W28, W28.0, W28.1, W28.2, W28.3, W28.4, W28.5, W28.6, W28.7, W28.8, W28.9, W29, W29.0, W29.1, W29.2, W29.3, W29.4, W29.5, W29.6, W29.7, W29.8, W29.9, W30, W30.0, W30.1, W30.2, W30.3, W30.4, W30.5, W30.6, W30.7, W30.8, W30.81, W30.89, W30.9, W31, W31.0, W31.1, W31.2, W31.3, W31.4, W31.5, W31.6, W31.7, W31.8, W31.81, W31.82, W31.83, W31.89, W31.9, W35, W35.0, W35.1, W35.2, W35.3, W35.4, W35.5, W35.6, W35.7, W35.8, W35.9, W36, W36.0, W36.1, W36.2, W36.3, W36.4, W36.5, W36.6, W36.7, W36.8, W36.9, W37, W37.0, W37.1, W37.2, W37.3, W37.4, W37.5, W37.6, W37.7, W37.8, W37.9, W38, W38.0, W38.1, W38.2, W38.3, W38.4, W38.5, W38.6, W38.7, W38.8, W38.9, W40, W40.0, W40.1, W40.2, W40.3, W40.4, W40.5, W40.6, W40.7, W40.8, W40.9, W41, W41.0, W41.1, W41.2, W41.3, W41.4, W41.5, W41.6, W41.7, W41.8, W41.9, W42, W42.0, W42.2, W42.3, W42.9, W43, W43.0, W43.1, W43.2, W43.3, W43.4, W43.5, W43.6, W43.7, W43.8, W43.9, W45.0, W45.1, W45.2, W46, W46.0, W46.1, W46.2, W49, W49.0, W49.01, W49.02, W49.03, W49.04, W49.09, W49.1, W49.2, W49.3, W49.4, W49.5, W49.6, W49.7, W49.8, W49.9, W50, W50.0, W50.1, W50.2, W50.3, W50.4, W50.5, W50.6, W50.7, W50.8, W50.9, W51, W51.0, W51.1, W51.2, W51.3, W51.4, W51.5, W51.6, W51.7, W51.8, W51.9, W52	E916, E916.0, E916.00, E916.01, E916.02, E916.03, E916.04, E916.05, E916.06, E916.07, E916.08, E916.09, E917, E917.0, E917.00, E917.01, E917.02, E917.03, E917.04, E917.05, E917.06, E917.07, E917.08, E917.09, E917.1, E917.10, E917.11, E917.12, E917.13, E917.14, E917.15, E917.16, E917.17, E917.18, E917.19, E917.2, E917.20, E917.21, E917.22, E917.23, E917.24, E917.25, E917.26, E917.27, E917.28, E917.29, E917.3, E917.4, E917.5, E917.6, E917.7, E917.8, E917.9, E917.90, E917.91, E917.92, E917.93, E917.94, E917.95, E917.96, E917.97, E917.98, E917.99, E918, E918.0, E918.00, E918.01, E918.02, E918.03, E918.04, E918.05, E918.06, E918.07, E918.08, E918.09, E919, E919.0, E919.00, E919.01, E919.02, E919.03, E919.04, E919.05, E919.06, E919.07, E919.08, E919.09, E919.1, E919.10, E919.11, E919.12, E919.13, E919.14, E919.15, E919.16, E919.17, E919.18, E919.19, E919.2, E919.20, E919.21, E919.22, E919.23, E919.24, E919.25, E919.26, E919.27, E919.28, E919.29, E919.3, E919.30, E919.31, E919.32, E919.33, E919.34, E919.35, E919.36, E919.37, E919.38, E919.39, E919.4, E919.40, E919.41, E919.42, E919.43, E919.44, E919.45, E919.46, E919.47, E919.48, E919.49, E919.5, E919.50, E919.51, E919.52, E919.53, E919.54, E919.55, E919.56, E919.57, E919.58, E919.59, E919.6, E919.60, E919.61, E919.62, E919.63, E919.64, E919.65, E919.66, E919.67, E919.68, E919.69, E919.7, E919.70, E919.71, E919.72, E919.73, E919.74, E919.75, E919.76, E919.77, E919.78, E919.79, E919.8, E919.80, E919.81, E919.82, E919.83, E919.84, E919.85, E919.86, E919.87, E919.88, E919.89, E919.9, E919.90, E919.91, E919.92, E919.93, E919.94, E919.95, E919.96, E919.97, E919.98, E919.99, E920, E920.0, E920.00, E920.01, E920.02, E920.03, E920.04, E920.05, E920.06, E920.07, E920.08, E920.09, E920.1, E920.10, E920.11, E920.12, E920.13, E920.14, E920.15, E920.16, E920.17, E920.18, E920.19, E920.2, E920.20, E920.21, E920.22, E920.23, E920.24, E920.25, E920.26, E920.27, E920.28, E920.29, E920.3, E920.30, E920.31, E920.32, E920.33, E920.34, E920.35, E920.36, E920.37, E920.38, E920.39, E920.4, E920.40, E920.41, E920.42, E920.43, E920.44, E920.45, E920.46, E920.47, E920.48, E920.49, E920.5, E920.8, E920.80, E920.81, E920.82, E920.83, E920.84, E920.85, E920.86, E920.87, E920.88, E920.89, E920.9, E920.90, E920.91, E920.92, E920.93, E920.94, E920.95, E920.96, E920.97, E920.98, E920.99, E921, E921.0, E921.00, E921.01, E921.02, E921.03, E921.04, E921.05
Other eye cancers	C69.0, C69.00, C69.01, C69.02, C69.1, C69.10, C69.11, C69.12, C69.3, C69.30, C69.31, C69.32, C69.4, C69.40, C69.41, C69.42, C69.5, C69.50, C69.51, C69.52, C69.6, C69.60, C69.61, C69.62, C69.7, C69.8, C69.80, C69.81, C69.82	190, 190.0, 190.1, 190.2, 190.3, 190.4, 190.6, 190.7, 190.8
Other gynecological diseases	N83, N83.0, N83.1, N83.2, N83.20, N83.29, N83.3, N83.31, N83.32, N83.33, N83.4, N83.5, N83.51, N83.52, N83.53, N83.6, N83.7, N83.8, N83.9	620, 620.0, 620.1, 620.2, 620.3, 620.4, 620.5, 620.6, 620.7, 620.8, 620.9, 621.4, 621.5, 621.6, 621.7, 621.8, 621.9, 622.3, 622.4, 622.5, 622.6, 629, 629.0, 629.1, 629.2, 629.20, 629.21, 629.22, 629.23, 629.29, 629.3, 629.31, 629.32, 629.8, 629.81
Other hemoglobinopathies and hemolytic anemias	D55.3, D55.8, D55.9, D58, D58.0, D58.1, D58.2, D58.8, D58.9, D59.1, D59.3, D59.5, D60, D60.0, D60.1, D60.8, D60.9, D64.0	282, 282.0, 282.1, 282.69, 282.7, 282.8, 282.9, 283, 283.0, 283.1, 283.10, 283.11, 283.19, 283.2, 283.9
Other intestinal infectious diseases	A07.0, A07.1, A07.8, A07.9	007, 007.0, 007.1, 007.2, 007.3, 007.9, 008, 008.0, 008.00, 008.03, 008.09, 008.1

Table S4. List of infectious syndromes mapped to International Classification of Diseases (ICD) codes

Cause Name	ICD 10 Codes	ICD 9 Codes
Other malignant neoplasms	C17, C17.0, C17.1, C17.2, C17.3, C17.8, C17.9, C30, C30.0, C30.1, C30.2, C30.3, C30.5, C30.8, C30.9, C31, C31.0, C31.1, C31.2, C31.3, C31.8, C31.9, C37, C37.0, C37.1, C37.2, C37.3, C38, C38.0, C38.1, C38.2, C38.3, C38.4, C38.8, C48, C48.0, C48.1, C48.2, C48.8, C48.9, C51, C51.0, C51.1, C51.2, C51.8, C51.9, C52, C52.0, C52.9, C57, C57.0, C57.00, C57.01, C57.02, C57.1, C57.10, C57.11, C57.12, C57.2, C57.20, C57.21, C57.22, C57.3, C57.4, C57.7, C57.8, C60, C60.0, C60.1, C60.2, C60.8, C60.9, C63, C63.0, C63.00, C63.01, C63.02, C63.1, C63.10, C63.11, C63.12, C63.2, C63.7, C63.8, C66, C66.0, C66.1, C66.2, C66.9, C68.0, C68.1, C68.8, C75, C75.0, C75.4, C75.5, C75.6, C75.8, D07.4, D09.2, D09.20, D09.21, D09.22, D13.2, D13.3, D13.30, D13.39, D14.0, D15, D15.0, D15.1, D15.2, D15.7, D15.9, D16, D16.0, D16.00, D16.01, D16.02, D16.1, D16.10, D16.11, D16.12, D16.2, D16.20, D16.21, D16.22, D16.3, D16.30, D16.31, D16.32, D16.4, D16.5, D16.6, D16.7, D16.8, D16.9, D28.0, D28.1, D28.7, D29.0, D30.2, D30.20, D30.21, D30.22, D30.4, D30.7, D30.8, D31, D31.0, D31.00, D31.01, D31.02, D31.1, D31.10, D31.11, D31.12, D31.2, D31.20, D31.21, D31.22, D31.3, D31.30, D31.31, D31.32, D31.4, D31.40, D31.41, D31.42, D31.5, D31.50, D31.51, D31.52, D31.6, D31.60, D31.61, D31.62, D31.9, D31.90, D31.91, D31.92, D35, D35.0, D35.00, D35.01, D35.02, D35.1, D35.2, D35.5, D35.6, D35.7, D35.8, D35.9, D36, D36.1, D36.10, D36.11, D36.12, D36.13, D36.14, D36.15, D36.16, D36.17, D36.7, D37.2, D38.2, D38.3, D38.4, D38.5, D39.2, D39.8, D41.2, D41.20, D41.21, D41.22, D41.3, D44.1, D44.10, D44.11, D44.12, D44.2, D44.3, D44.4, D44.5, D44.6, D44.7, D44.8, D48.0, D48.1, D48.2, D48.3, D48.4, D49.81	152, 152.0, 152.1, 152.2, 152.3, 152.4, 152.6, 152.8, 152.9, 158, 158.0, 158.3, 158.4, 158.5, 158.6, 158.8, 158.9, 160, 160.0, 160.1, 160.2, 160.3, 160.4, 160.5, 160.6, 160.8, 160.9, 163, 163.0, 163.1, 163.3, 163.5, 163.8, 163.9, 164, 164.0, 164.1, 164.2, 164.3, 164.8, 164.9, 183.2, 183.3, 183.4, 183.5, 183.8, 184.0, 184.1, 184.2, 184.3, 184.4, 184.8, 187.1, 187.2, 187.3, 187.4, 187.5, 187.6, 187.7, 187.8, 189.2, 189.3, 189.4, 189.8, 194.1, 194.5, 194.6, 194.8, 209.0, 209.00, 209.01, 209.02, 209.03, 209.22, 209.25, 209.26, 209.27, 209.31, 209.32, 209.33, 209.34, 209.35, 209.36, 209.4, 209.40, 209.41, 209.42, 209.43, 211.2, 211.8, 212.0, 212.4, 212.5, 212.6, 212.7, 212.8, 213, 213.0, 213.1, 213.2, 213.3, 213.4, 213.5, 213.6, 213.7, 213.8, 213.9, 221.0, 221.1, 221.2, 221.8, 222.1, 222.8, 223.2, 223.8, 223.81, 223.89, 224, 224.0, 224.1, 224.2, 224.3, 224.4, 224.5, 224.6, 224.7, 224.8, 224.9, 227, 227.0, 227.1, 227.3, 227.4, 227.5, 227.6, 227.8, 227.9, 228, 228.0, 228.00, 228.01, 228.02, 228.03, 228.04, 228.09, 228.1, 228.9, 229.0, 229.8, 230.7, 230.8, 233.31, 233.32, 233.4, 233.5, 234.0, 234.5, 234.8, 235.4, 235.8, 236.1, 236.99, 238.0, 238.1, 239.2
Other musculoskeletal disorders	I27.1, I67.7, M07, M07.0, M07.1, M07.2, M07.3, M07.4, M07.5, M07.6, M07.60, M07.61, M07.611, M07.612, M07.619, M07.62, M07.621, M07.622, M07.629, M07.63, M07.631, M07.632, M07.639, M07.64, M07.641, M07.642, M07.649, M07.65, M07.651, M07.652, M07.659, M07.66, M07.661, M07.662, M07.669, M07.67, M07.671, M07.672, M07.679, M07.68, M07.69, M08, M30, M30.0, M30.1, M30.2, M30.3, M30.8, M31, M31.0, M31.1, M31.2, M31.3, M31.30, M31.31, M31.4, M31.5, M31.6, M31.7, M31.8, M31.9, M34, M34.0, M34.1, M34.2, M34.8, M34.81, M34.82, M34.83, M34.89, M34.9, M35, M35.0, M35.00, M35.01, M35.02, M35.03, M35.04, M35.09, M35.1, M35.2, M35.3, M35.4, M35.5, M35.6, M35.7, M35.8, M35.9, M36, M36.0, M36.1, M36.2, M36.3, M36.4, M36.8, M40, M40.0, M40.00, M40.03, M40.04, M40.05, M40.1, M40.10, M40.12, M40.13, M40.14, M40.15, M40.2, M40.20, M40.202, M40.203, M40.204, M40.205, M40.209, M40.29, M40.292, M40.293, M40.294, M40.295, M40.299, M40.3, M40.30, M40.35, M40.36, M40.37, M40.4, M40.40, M40.45, M40.46, M40.47, M40.5, M40.50, M40.55, M40.56, M40.57, M41, M41.0, M41.00, M41.02, M41.03, M41.04, M41.05, M41.06, M41.07, M41.08, M41.1, M41.11, M41.112, M41.113, M41.114, M41.115, M41.116, M41.117, M41.119, M41.12, M41.122, M41.123, M41.124, M41.125, M41.126, M41.127, M41.129, M41.2, M41.20, M41.22, M41.23, M41.24, M41.25, M41.26, M41.27, M41.3, M41.30, M41.34, M41.35, M41.4, M41.40, M41.41, M41.42, M41.43, M41.44, M41.45, M41.46, M41.47, M41.5, M41.50, M41.52, M41.53, M41.54, M41.55, M41.56, M41.57, M41.8, M41.80, M41.82, M41.83, M41.84, M41.85, M41.86, M41.87, M41.9, M42, M42.0, M42.00, M42.01, M42.02, M42.03, M42.04, M42.05, M42.06, M42.07, M42.08, M42.09, M42.1, M42.10, M42.11, M42.12, M42.13, M42.14, M42.15, M42.16, M42.17, M42.18, M42.19, M42.9, M43, M43.0, M43.00, M43.01, M43.02, M43.03, M43.04, M43.05, M43.06, M43.07, M43.08, M43.09, M43.1, M43.10, M43.11, M43.12, M43.13, M43.14, M43.15, M43.16, M43.17, M43.18, M43.19, M87, M87.0, M87.00, M87.01, M87.011, M87.012, M87.019, M87.02, M87.021, M87.022, M87.029, M87.03, M87.031, M87.032, M87.033, M87.034, M87.035, M87.036, M87.037, M87.038, M87.039, M87.04, M87.041, M87.042, M87.043, M87.044, M87.045, M87.046, M87.05, M87.050, M87.051, M87.052, M87.059, M87.06, M87.061, M87.062	416.1, 437.4, 446, 446.0, 446.1, 446.2, 446.20, 446.21, 446.29, 446.3, 446.4, 446.5, 446.6, 446.7, 446.9, 710, 710.1, 710.2, 710.3, 710.4, 710.5, 710.8, 710.9, 732, 732.0, 732.1, 732.2, 732.3, 732.4, 732.5, 732.6, 732.7, 732.8, 732.9

Table S4. List of infectious syndromes mapped to International Classification of Diseases (ICD) codes		
**Cause Name**	**ICD 10 Codes**	**ICD 9 Codes**
Other neglected tropical diseases	A68, A68.0, A68.1, A68.9, A69.2, A69.20, A69.21, A69.22, A69.23, A69.29, A69.5, A69.8, A69.9, A75, A75.0, A75.1, A75.2, A75.3, A75.9, A77, A77.0, A77.1, A77.2, A77.3, A77.4, A77.40, A77.41, A77.49, A77.8, A77.9, A78, A79, A79.0, A79.1, A79.8, A79.81, A79.89, A79.9, A92, A92.0, A92.1, A92.2, A92.3, A92.30, A92.31, A92.32, A92.39, A92.4, A92.8, A92.9, A93, A93.0, A93.1, A93.2, A93.8, A94, A94.0, A96, A96.0, A96.1, A96.2, A96.8, A96.9, A98, A98.0, A98.1, A98.2, A98.3, A98.5, A98.8, B33.0, B33.1, B60, B60.0, B60.1, B60.10, B60.11, B60.12, B60.13, B60.19, B60.2, B60.8, B67.5, B67.6, B67.61, B67.69, B67.7, B70, B70.0, B70.1, B71, B71.0, B71.1, B71.8, B71.9, B74.3, B74.4, B74.8, B74.9, B75, B83, B83.0, B83.1, B83.2, B83.3, B83.4, B83.8, P37.1	065, 065.0, 065.1, 065.2, 065.3, 065.4, 065.6, 065.8, 065.9, 066, 066.0, 066.1, 066.2, 066.3, 066.4, 066.40, 066.41, 066.42, 066.49, 066.5, 066.8, 066.9, 080, 080.0, 080.2, 080.5, 080.7, 080.8, 081, 081.0, 081.1, 081.2, 081.3, 081.5, 081.6, 081.9, 082, 082.0, 082.1, 082.2, 082.3, 082.4, 082.40, 082.41, 082.49, 082.5, 082.7, 082.8, 082.9, 083, 083.0, 083.1, 083.2, 083.3, 083.6, 083.8, 083.9, 087, 087.0, 087.1, 087.3, 087.9, 088, 088.8, 088.81, 088.82, 088.89, 088.9, 122.5, 122.6, 122.7, 123, 123.0, 123.2, 123.3, 123.4, 123.5, 123.6, 123.8, 123.9, 124, 124.0, 124.9, 125.4, 125.5, 125.6, 125.9, 127, 127.1, 128, 128.0, 128.1, 128.4, 128.5, 128.8, 128.9, 129, 129.0
Other neonatal disorders	P00, P00.0, P00.1, P00.2, P00.3, P00.4, P00.5, P00.6, P00.7, P00.8, P00.81, P00.89, P00.9, P01, P01.2, P01.3, P01.4, P01.5, P01.6, P01.8, P01.9, P04, P04.0, P04.1, P04.2, P04.5, P04.6, P04.8, P04.9, P08, P08.0, P08.1, P08.2, P08.21, P08.22, P09, P19, P19.0, P19.1, P19.2, P19.9, P29, P29.0, P29.1, P29.11, P29.12, P29.2, P29.3, P29.4, P29.8, P29.81, P29.89, P29.9, P50, P50.0, P50.1, P50.2, P50.3, P50.4, P50.5, P50.8, P50.9, P51, P51.0, P51.8, P51.9, P53, P53.0, P54, P54.0, P54.1, P54.2, P54.3, P54.4, P54.5, P54.6, P54.8, P54.9, P60, P60.0, P61, P61.0, P61.1, P61.3, P61.4, P61.5, P61.6, P61.8, P61.9, P70, P70.0, P70.1, P70.3, P70.4, P70.8, P70.9, P71, P71.0, P71.1, P71.2, P71.3, P71.4, P71.8, P71.9, P72, P72.0, P72.1, P72.2, P72.8, P72.9, P74, P74.0, P74.1, P74.2, P74.3, P74.4, P74.5, P74.6, P74.8, P74.9, P75, P75.0, P76, P76.0, P76.1, P76.2, P76.8, P76.9, P78, P80, P80.0, P80.8, P80.9, P81, P81.0, P81.8, P81.9, P83, P83.0, P83.1, P83.2, P83.3, P83.30, P83.39, P83.4, P83.5, P83.6, P83.8, P83.9, P84, P92, P92.0, P92.01, P92.09, P92.1, P92.2, P92.3, P92.4, P92.5, P92.6, P92.8, P92.9, P94, P94.0, P94.1, P94.2, P94.8, P94.9, P96, P96.3, P96.4, P96.8, P96.81, P96.82, P96.83, P96.89	760, 760.0, 760.1, 760.2, 760.3, 760.4, 760.5, 760.6, 760.61, 760.62, 760.63, 760.64, 760.8, 760.9, 761, 761.2, 761.3, 761.4, 761.5, 761.6, 766, 766.0, 766.1, 766.2, 766.21, 766.22, 766.9, 770, 771, 772, 772.0, 775, 775.0, 775.4, 775.5, 775.6, 775.7, 775.8, 775.81, 775.89, 775.9, 776, 776.0, 776.1, 776.2, 776.3, 776.4, 776.5, 776.7, 776.8, 776.9, 777, 778, 778.0, 778.1, 778.2, 778.3, 778.4, 778.5, 778.6, 778.7, 778.8, 778.9, 779, 779.3, 779.31, 779.32, 779.33, 779.34, 779.6, 779.7, 779.8, 779.81, 779.82, 779.83, 779.84, 779.85, 779.89
Other neurological disorders	F02.2, G10, G10.0, G10.2, G10.9, G11, G11.0, G11.1, G11.2, G11.3, G11.4, G11.8, G11.9, G12, G12.0, G12.1, G13, G13.0, G13.1, G13.2, G13.8, G23, G23.0, G23.1, G23.2, G23.5, G23.8, G23.9, G24, G24.1, G24.2, G24.3, G24.4, G24.5, G24.8, G24.9, G25, G25.0, G25.2, G25.3, G25.5, G25.8, G25.81, G25.82, G25.83, G25.89, G25.9, G26, G26.0, G36, G36.0, G36.1, G36.8, G36.9, G37, G37.0, G37.1, G37.2, G37.3, G37.4, G37.5, G37.8, G37.9, G61, G61.0, G61.1, G61.8, G61.81, G61.89, G61.9, G70, G70.0, G70.00, G70.01, G70.1, G70.2, G70.8, G70.80, G70.81, G70.89, G70.9, G71, G71.0, G71.1, G71.11, G71.12, G71.13, G71.14, G71.19, G71.3, G71.8, G71.9, G73, G73.0, G73.1, G73.2, G73.3, G73.4, G73.5, G73.6, G73.7, G90, G90.0, G90.01, G90.09, G90.1, G90.2, G90.3, G90.4, G90.5, G90.50, G90.51, G90.511, G90.512, G90.513, G90.519, G90.52, G90.521, G90.522, G90.523, G90.529, G90.59, G90.8, G90.9, G95, G95.0, G95.1, G95.11, G95.19, G95.2, G95.20, G95.29, G95.3, G95.6, G95.8, G95.81, G95.89, G95.9	330, 330.0, 330.1, 330.2, 330.3, 330.4, 330.8, 330.9, 331.5, 331.6, 331.7, 331.8, 331.82, 331.83, 331.89, 331.9, 333, 333.0, 333.1, 333.2, 333.3, 333.4, 333.5, 333.6, 333.7, 333.71, 333.72, 333.79, 333.8, 333.81, 333.82, 333.83, 333.84, 333.85, 333.89, 333.9, 333.90, 333.91, 333.92, 333.93, 333.94, 333.99, 334, 334.0, 334.1, 334.2, 334.3, 334.4, 334.8, 334.9, 335.3, 336, 336.0, 336.1, 336.2, 336.3, 336.8, 336.9, 337, 337.0, 337.00, 337.01, 337.09, 337.1, 337.2, 337.20, 337.21, 337.22, 337.29, 337.3, 337.9, 341, 341.0, 341.1, 341.2, 341.20, 341.21, 341.22, 341.8, 341.9, 349, 349.2, 349.3, 349.39, 349.8, 353.8, 353.9, 356, 356.0, 356.1, 356.2, 356.3, 356.4, 356.8, 356.9, 357.0, 357.1, 357.3, 357.4, 357.7, 358, 358.0, 358.00, 358.01, 358.1, 358.2, 358.3, 358.30, 358.31, 358.39, 358.8, 358.9, 359, 359.0, 359.1, 359.2, 359.21, 359.22, 359.23, 359.29, 359.3, 359.4, 359.5, 359.6, 359.7, 359.71, 359.79, 359.8, 359.81, 359.89, 359.9, 775.2
Other nutritional deficiencies	D51, D51.0, D51.1, D51.2, D51.3, D51.8, D51.9, D52, D52.0, D52.8, D52.9, D53, D53.0, D53.1, D53.2, D53.8, D53.9, E00, E00.0, E00.1, E00.2, E00.9, E01, E01.0, E01.1, E01.2, E01.8, E02, E51, E51.1, E51.11, E51.12, E51.2, E51.8, E51.9, E52, E53, E53.0, E53.1, E53.8, E53.9, E54, E54.0, E55, E55.0, E55.9, E56, E56.0, E56.1, E56.8, E56.9, E57, E58, E59, E59.0, E60, E61, E61.0, E61.1, E61.2, E61.3, E61.4, E61.5, E61.6, E61.7, E61.8, E61.9, E63, E63.0, E63.1, E63.8, E63.9, E64, E64.2, E64.3, E64.8, E64.9, M12.1, M12.10, M12.11, M12.111, M12.112, M12.119, M12.12, M12.121, M12.122, M12.129, M12.13, M12.131, M12.132, M12.139, M12.14, M12.141, M12.142, M12.149, M12.15, M12.151, M12.152, M12.159, M12.16, M12.161, M12.162, M12.169, M12.17, M12.171, M12.172, M12.179, M12.18, M12.19	244.2, 265, 265.0, 265.1, 265.2, 265.9, 266, 266.0, 266.1, 266.2, 266.9, 267, 267.0, 267.9, 268, 268.0, 268.1, 268.2, 268.9, 269, 269.0, 269.1, 269.2, 269.3, 269.8, 269.9, 281.0, 281.1, 281.2, 281.3, 281.4, 281.8, 281.9, 716.0, 716.00, 716.01, 716.02, 716.03, 716.04, 716.05, 716.06, 716.07, 716.08, 716.09
Other pharynx cancer	C09, C09.0, C09.1, C09.8, C09.9, C10, C10.0, C10.1, C10.2, C10.3, C10.4, C10.8, C10.9, C12, C12.0, C12.9, C13, C13.0, C13.1, C13.2, C13.8, C13.9, D10.7	146, 146.0, 146.1, 146.2, 146.3, 146.4, 146.5, 146.6, 146.7, 146.8, 146.9, 148, 148.0, 148.1, 148.2, 148.3, 148.4, 148.5, 148.8, 148.9
Other sexually transmitted infections	A57, A57.0, A58, A63, A63.0, A63.8, B63	099, 099.0, 099.1, 099.2, 099.3, 099.4, 099.40, 099.41, 099.49, 099.5, 099.50, 099.51, 099.52, 099.53, 099.54, 099.55, 099.56, 099.59, 099.6, 099.8, 099.9

Table S4. List of infectious syndromes mapped to International Classification of Diseases (ICD) codes

Cause Name	ICD 10 Codes	ICD 9 Codes
Other skin and subcutaneous diseases	D86.3, L10, L10.0, L10.1, L10.2, L10.3, L10.4, L10.5, L10.8, L10.81, L10.89, L10.9, L11, L11.0, L11.1, L11.8, L11.9, L12, L12.0, L12.1, L12.2, L12.3, L12.30, L12.31, L12.35, L12.8, L12.9, L13, L13.0, L13.1, L13.8, L13.9, L14, L14.0, L51, L51.0, L51.1, L51.2, L51.3, L51.8, L51.9	694, 694.0, 694.1, 694.2, 694.3, 694.4, 694.5, 694.6, 694.60, 694.61, 694.8, 694.9, 695, 695.0, 695.1, 695.10, 695.11, 695.12, 695.13, 695.14, 695.15, 695.19, 695.2, 695.3
Other unintentional injuries (internal)	W39, W39.0, W39.1, W39.2, W39.3, W39.4, W39.5, W39.6, W39.7, W39.8, W39.9, W77, W77.0, W77.1, W77.2, W77.3, W77.4, W77.5, W77.6, W77.7, W77.8, W77.9, W81, W81.0, W81.1, W81.2, W81.3, W81.4, W81.5, W81.6, W81.7, W81.8, W81.9, X50, X50.0, X50.1, X50.2, X50.3, X50.4, X50.5, X50.6, X50.7, X50.8, X50.9, X51, X51.0, X51.1, X51.2, X51.3, X51.4, X51.5, X51.6, X51.8, X51.9, X52, X52.0, X52.1, X52.4, X52.6, X52.7, X52.8, X52.9, X53, X53.0, X53.1, X53.2, X53.3, X53.4, X53.5, X53.6, X53.7, X53.8, X53.9, X54, X54.0, X54.1, X54.2, X54.3, X54.4, X54.5, X54.8, X54.9, X57, X57.0, X57.1, X57.2, X57.4, X57.5, X57.6, X57.8, X57.9, X58, X58.0, X58.1, X58.2, X58.3, X58.4, X58.5, X58.6, X58.7, X58.8, X58.9	E903, E903.0, E903.00, E903.01, E903.02, E903.03, E903.04, E903.05, E903.06, E903.07, E903.08, E903.09, E904, E904.0, E904.00, E904.01, E904.02, E904.03, E904.04, E904.05, E904.06, E904.07, E904.08, E904.09, E904.1, E904.10, E904.11, E904.12, E904.13, E904.14, E904.15, E904.16, E904.17, E904.18, E904.19, E904.2, E904.20, E904.21, E904.22, E904.23, E904.24, E904.25, E904.26, E904.27, E904.28, E904.29, E904.3, E904.30, E904.31, E904.32, E904.33, E904.34, E904.35, E904.36, E904.37, E904.38, E904.39, E904.9, E904.90, E904.91, E904.92, E904.93, E904.94, E904.95, E904.96, E904.97, E904.98, E904.99, E913.2, E913.20, E913.21, E913.22, E913.23, E913.24, E913.25, E913.26, E913.27, E913.28, E913.29, E913.3, E913.30, E913.31, E913.32, E913.33, E913.34, E913.35, E913.36, E913.37, E913.38, E913.39, E923, E923.0, E923.00, E923.01, E923.02, E923.03, E923.04, E923.05, E923.06, E923.07, E923.08, E923.09, E923.1, E923.10, E923.11, E923.12, E923.13, E923.14, E923.15, E923.16, E923.17, E923.18, E923.19, E923.2, E923.20, E923.21, E923.22, E923.23, E923.24, E923.25, E923.26, E923.27, E923.28, E923.29, E923.8, E923.80, E923.81, E923.82, E923.83, E923.84, E923.85, E923.86, E923.87, E923.88, E923.89, E923.9, E923.90, E923.91, E923.92, E923.93, E923.94, E923.95, E923.96, E923.97, E923.98, E923.99, E927, E927.0, E927.00, E927.01, E927.02, E927.03, E927.04, E927.05, E927.06, E927.07, E927.08, E927.09, E927.1, E927.2, E927.3, E927.4, E927.8, E927.9, E928, E928.0, E928.00, E928.01, E928.02, E928.03, E928.04, E928.05, E928.06, E928.07, E928.08, E928.09, E928.8, E928.80, E928.81, E928.82, E928.83, E928.84, E928.85, E928.86, E928.87, E928.88, E928.89
Other unspecified infectious diseases	A32, A32.0, A32.1, A32.11, A32.12, A32.7, A32.8, A32.81, A32.82, A32.89, A32.9, A38, A38.0, A38.1, A38.8, A38.9, A48.2, A48.4, A48.5, A48.51, A48.52, A65, A65.0, A69, A69.0, A69.1, A74, A74.8, A74.81, A74.89, A74.9, A81, A81.0, A81.00, A81.01, A81.09, A81.1, A81.2, A81.8, A81.81, A81.82, A81.83, A81.89, A81.9, A88, A88.0, A88.1, A88.8, A89, A89.0, A89.5, A89.9, B03, B04, B25, B25.0, B25.1, B25.2, B25.8, B25.9, B27, B27.0, B27.00, B27.01, B27.02, B27.09, B27.1, B27.10, B27.11, B27.12, B27.19, B27.8, B27.80, B27.81, B27.82, B27.89, B27.9, B27.90, B27.91, B27.92, B27.99, B29.4, B33, B33.3, B33.4, B33.8, B47, B47.0, B47.1, B47.4, B47.9, B48, B48.0, B48.1, B48.2, B48.3, B48.4, B48.7, B48.8, B91, B95, B95.0, B95.1, B95.2, B95.3, B95.4, B95.5, D70.3, D89.3, F02.1, G14, G14.6, I00, I02, I02.9, I98.1, K67.8, K75.3, K76.3, K77.0, M89.6, M89.60, M89.61, M89.611, M89.612, M89.619, M89.62, M89.621, M89.622, M89.629, M89.63, M89.631, M89.632, M89.639, M89.64, M89.641, M89.642, M89.649, M89.65, M89.651, M89.652, M89.659, M89.66, M89.661, M89.662, M89.669, M89.67, M89.671, M89.672, M89.679, M89.68, M89.69, P35, P35.1, P35.2, P35.9, P37, P37.2, P37.5, P37.8, P37.9	034, 034.1, 034.4, 034.8, 034.9, 040, 040.1, 040.2, 040.3, 040.4, 040.41, 040.42, 040.5, 040.8, 040.81, 040.82, 040.89, 040.9, 041, 041.0, 041.00, 041.01, 041.02, 041.03, 041.04, 041.05, 041.09, 046, 046.0, 046.1, 046.11, 046.19, 046.2, 046.3, 046.6, 046.7, 046.71, 046.72, 046.79, 046.8, 046.9, 050, 050.0, 050.1, 050.2, 050.3, 050.4, 050.5, 050.6, 050.9, 051, 051.0, 051.01, 051.02, 051.1, 051.2, 051.4, 051.5, 051.8, 051.9, 057, 057.0, 057.1, 057.2, 057.4, 057.5, 057.6, 057.8, 057.9, 058, 058.1, 058.10, 058.11, 058.12, 058.2, 058.21, 058.29, 058.4, 058.5, 058.6, 058.8, 058.81, 058.82, 058.89, 059, 059.0, 059.00, 059.01, 059.09, 059.1, 059.10, 059.11, 059.12, 059.19, 059.2, 059.20, 059.21, 059.22, 059.3, 059.5, 059.6, 059.8, 059.9, 074, 074.0, 074.1, 074.2, 074.20, 074.21, 074.22, 074.23, 074.3, 074.4, 074.8, 075, 075.0, 075.2, 075.3, 075.4, 075.5, 075.6, 075.7, 075.9, 078.4, 078.5, 078.6, 078.7, 079, 079.0, 079.1, 079.2, 079.3, 079.4, 079.5, 079.50, 079.51, 079.52, 079.53, 079.59, 079.7, 101, 101.0, 101.3, 101.4, 101.6, 104, 104.0, 104.1, 104.2, 104.3, 104.8, 104.9, 136, 136.0, 136.1, 136.2, 136.21, 136.29, 139, 323.0, 323.01, 323.02, 323.1, 323.2, 323.3, 390, 390.0, 390.9, 392, 392.9, 771.0, 771.1, 771.2
Other urinary diseases	N25, N25.0, N25.1, N25.8, N25.81, N25.89, N25.9, N26, N26.0, N26.1, N26.2, N26.9, N27, N27.0, N27.1, N27.9, N28, N28.0, N28.1, N29, N29.0, N29.1, N29.8, N31, N31.0, N31.1, N31.2, N31.8, N31.9, N32, N32.0, N32.3, N32.4, N36, N36.0, N36.1, N36.2, N36.3, N36.4, N36.41, N36.42, N36.43, N36.44, N36.5, N36.8, N36.9, N39, N44, N44.0, N44.00, N44.01, N44.02, N44.03, N44.04	588, 588.0, 588.1, 588.8, 588.81, 588.89, 588.9, 593, 593.0, 593.1, 593.2, 593.3, 593.4, 593.5, 593.6, 593.7, 593.70, 593.71, 593.72, 593.73, 593.8, 593.81, 593.82, 593.89, 596, 596.0, 596.1, 596.2, 596.3, 596.4, 596.5, 596.51, 596.52, 596.53, 596.54, 596.55, 596.59, 596.6, 596.7, 596.8, 596.81, 596.89, 596.9, 599.8, 599.81, 599.82, 599.83, 599.84, 599.89, 608.2, 608.20, 608.21, 608.22, 608.23, 608.24

Table S4. List of infectious syndromes mapped to International Classification of Diseases (ICD) codes

Cause Name	ICD 10 Codes	ICD 9 Codes
Otitis media	H70, H70.0, H70.00, H70.001, H70.002, H70.003, H70.009, H70.01, H70.011, H70.012, H70.013, H70.019, H70.09, H70.091, H70.092, H70.093, H70.099, H70.1, H70.10, H70.11, H70.12, H70.13, H70.2, H70.20, H70.201, H70.202, H70.203, H70.209, H70.21, H70.211, H70.212, H70.213, H70.219, H70.22, H70.221, H70.222, H70.223, H70.229, H70.8, H70.81, H70.811, H70.812, H70.813, H70.819, H70.89, H70.891, H70.892, H70.893, H70.899, H70.9, H70.90, H70.91, H70.92, H70.93	381, 381.0, 381.00, 381.01, 381.02, 381.03, 381.04, 381.05, 381.06, 381.1, 381.10, 381.19, 381.2, 381.20, 381.29, 381.3, 381.4, 381.5, 381.50, 381.51, 381.52, 381.6, 381.60, 381.61, 381.62, 381.63, 381.7, 381.8, 381.81, 381.89, 381.9, 382, 382.0, 382.00, 382.01, 382.02, 382.1, 382.2, 382.3, 382.4, 382.9, 383, 383.0, 383.00, 383.01, 383.02, 383.1, 383.2, 383.20, 383.21, 383.22, 383.3, 383.30, 383.31, 383.32, 383.33, 383.8, 383.81, 383.89, 383.9
Ovarian cancer	C56, C56.0, C56.1, C56.2, C56.4, C56.9, D27, D27.0, D27.1, D27.9, D39.1, D39.10, D39.11, D39.12	183, 183.0, 220, 220.0, 220.9, 236.2
Pancreatic cancer	C25, C25.0, C25.1, C25.2, C25.3, C25.4, C25.7, C25.8, C25.9, D13.6, D13.7	157, 157.0, 157.1, 157.2, 157.3, 157.4, 157.5, 157.7, 157.8, 157.9, 211.6, 211.7
Pancreatitis	K85, K85.0, K85.1, K85.2, K85.3, K85.8, K85.9, K86, K86.0, K86.1, K86.2, K86.3, K86.8, K86.9	577, 577.0, 577.1, 577.2, 577.3, 577.8, 577.9, 579.4
Paralytic ileus and intestinal obstruction	K56, K56.0, K56.1, K56.2, K56.3, K56.4, K56.41, K56.49, K56.5, K56.6, K56.60, K56.69, K56.7, K56.8, K56.9	560, 560.0, 560.1, 560.2, 560.3, 560.30, 560.31, 560.32, 560.39, 560.8, 560.81, 560.89, 560.9
Paratyphoid fever	A01.1, A01.2, A01.3, A01.4	002.1, 002.2, 002.3, 002.4, 002.9
Parkinson's disease	F02.3, G20, G20.0, G20.3, G20.9	332, 332.0
Peptic ulcer disease	K25, K25.0, K25.1, K25.2, K25.3, K25.4, K25.5, K25.6, K25.7, K25.8, K25.9, K26, K26.0, K26.1, K26.2, K26.3, K26.4, K26.5, K26.6, K26.7, K26.9, K27, K27.0, K27.1, K27.2, K27.3, K27.4, K27.5, K27.6, K27.7, K27.8, K27.9, K28, K28.0, K28.1, K28.2, K28.3, K28.4, K28.5, K28.6, K28.7, K28.9	531, 531.0, 531.00, 531.01, 531.1, 531.10, 531.11, 531.2, 531.20, 531.21, 531.3, 531.30, 531.31, 531.4, 531.40, 531.41, 531.5, 531.50, 531.51, 531.6, 531.60, 531.61, 531.7, 531.70, 531.71, 531.8, 531.9, 531.90, 531.91, 532, 532.0, 532.00, 532.01, 532.1, 532.10, 532.11, 532.2, 532.20, 532.21, 532.3, 532.30, 532.31, 532.4, 532.40, 532.41, 532.5, 532.50, 532.51, 532.6, 532.60, 532.61, 532.7, 532.70, 532.71, 532.8, 532.9, 532.90, 532.91, 533, 533.0, 533.00, 533.01, 533.1, 533.10, 533.11, 533.2, 533.20, 533.21, 533.3, 533.30, 533.31, 533.4, 533.40, 533.41, 533.5, 533.50, 533.51, 533.6, 533.60, 533.61, 533.7, 533.70, 533.71, 533.8, 533.9, 533.90, 533.91, 534, 534.0, 534.00, 534.01, 534.1, 534.10, 534.11, 534.2, 534.20, 534.21, 534.3, 534.30, 534.31, 534.4, 534.40, 534.41, 534.5, 534.50, 534.51, 534.6, 534.60, 534.61, 534.7, 534.70, 534.71, 534.9, 534.90, 534.91
Pertussis	A37, A37.0, A37.00, A37.01, A37.1, A37.10, A37.11, A37.8, A37.80, A37.81, A37.9, A37.90, A37.91	033, 033.0, 033.1, 033.3, 033.8, 033.9, 484.3
Physical violence by firearm	X93, X93.0, X93.1, X93.2, X93.3, X93.4, X93.5, X93.6, X93.7, X93.8, X93.9, X94, X94.0, X94.1, X94.2, X94.3, X94.4, X94.5, X94.6, X94.7, X94.8, X94.9, X95, X95.0, X95.01, X95.02, X95.09, X95.1, X95.2, X95.3, X95.4, X95.5, X95.6, X95.7, X95.8, X95.9	E965, E965.0, E965.1, E965.2, E965.3, E965.4
Physical violence by other means	X85, X85.0, X85.1, X85.2, X85.3, X85.4, X85.5, X85.6, X85.7, X85.8, X85.9, X86, X86.0, X86.1, X86.2, X86.4, X86.5, X86.6, X86.8, X86.9, X87, X87.0, X87.1, X87.2, X87.3, X87.4, X87.5, X87.6, X87.7, X87.8, X87.9, X88, X88.0, X88.1, X88.2, X88.3, X88.4, X88.5, X88.6, X88.7, X88.8, X88.9, X89, X89.0, X89.1, X89.2, X89.3, X89.4, X89.5, X89.6, X89.7, X89.8, X89.9, X90, X90.0, X90.1, X90.2, X90.3, X90.4, X90.5, X90.6, X90.7, X90.8, X90.9, X91, X91.0, X91.1, X91.2, X91.3, X91.4, X91.5, X91.6, X91.7, X91.8, X91.9, X92, X92.0, X92.1, X92.2, X92.3, X92.4, X92.5, X92.6, X92.7, X92.8, X92.9, X96, X96.0, X96.1, X96.2, X96.3, X96.4, X96.5, X96.6, X96.7, X96.8, X96.9, X97, X97.0, X97.1, X97.2, X97.3, X97.4, X97.5, X97.6, X97.7, X97.8, X97.9, X98, X98.0, X98.1, X98.2, X98.3, X98.4, X98.5, X98.7, X98.8, X98.9, Y00, Y00.0, Y00.1, Y00.2, Y00.3, Y00.4, Y00.5, Y00.6, Y00.7, Y00.8, Y00.9, Y01, Y01.0, Y01.1, Y01.2, Y01.3, Y01.4, Y01.5, Y01.6, Y01.7, Y01.8, Y01.9, Y02, Y02.0, Y02.1, Y02.2, Y02.3, Y02.4, Y02.5, Y02.6, Y02.7, Y02.8, Y02.9, Y03, Y03.0, Y03.1, Y03.2, Y03.3, Y03.4, Y03.5, Y03.6, Y03.7, Y03.8, Y03.9, Y04, Y04.0, Y04.1, Y04.2, Y04.3, Y04.4, Y04.5, Y04.6, Y04.7, Y04.8, Y04.9, Y06, Y06.0, Y06.1, Y06.2, Y06.8, Y06.9, Y07, Y07.0, Y07.01, Y07.02, Y07.03, Y07.04, Y07.1, Y07.11, Y07.12, Y07.13, Y07.14, Y07.2, Y07.3, Y07.4, Y07.41, Y07.410, Y07.411, Y07.42, Y07.420, Y07.421, Y07.43, Y07.430, Y07.432, Y07.433, Y07.434, Y07.435, Y07.436, Y07.49, Y07.490, Y07.491, Y07.499, Y07.5, Y07.50, Y07.51, Y07.510, Y07.511, Y07.512, Y07.513, Y07.519, Y07.52, Y07.521, Y07.528, Y07.529, Y07.53, Y07.59, Y07.8, Y07.9, Y08, Y08.0, Y08.01, Y08.02, Y08.09, Y08.1, Y08.2, Y08.3, Y08.4, Y08.5, Y08.6, Y08.7, Y08.8, Y08.81, Y08.89, Y08.9, Y87.1	E961, E962, E962.0, E962.1, E962.2, E962.9, E963, E964, E965.5, E965.6, E965.7, E965.8, E965.9, E967, E967.0, E967.1, E967.2, E967.3, E967.4, E967.5, E967.6, E967.7, E967.8, E967.9, E968, E968.0, E968.1, E968.2, E968.3, E968.4, E968.5, E968.6, E968.7, E968.8, E968.9, E969
Physical violence by sharp object	X99, X99.0, X99.1, X99.2, X99.3, X99.4, X99.5, X99.6, X99.7, X99.8, X99.9	E966

Table S4. List of infectious syndromes mapped to International Classification of Diseases (ICD) codes		
**Cause Name**	**ICD 10 Codes**	**ICD 9 Codes**
Pneumoconiosis		
Poisoning by carbon monoxide	X47, X47.0, X47.1, X47.2, X47.3, X47.4, X47.5, X47.6, X47.7, X47.8, X47.9	E862, E862.0, E862.00, E862.01, E862.02, E862.03, E862.04, E862.05, E862.06, E862.07, E862.08, E862.09, E862.1, E862.10, E862.11, E862.12, E862.13, E862.14, E862.15, E862.16, E862.17, E862.18, E862.19, E862.2, E862.20, E862.21, E862.22, E862.23, E862.24, E862.25, E862.26, E862.27, E862.28, E862.29, E862.3, E862.30, E862.31, E862.32, E862.33, E862.34, E862.35, E862.36, E862.37, E862.38, E862.39, E862.4, E862.40, E862.41, E862.42, E862.43, E862.44, E862.45, E862.46, E862.47, E862.48, E862.49, E862.9, E862.90, E862.91, E862.92, E862.93, E862.94, E862.95, E862.96, E862.97, E862.98, E862.99, E868, E868.0, E868.00, E868.01, E868.02, E868.03, E868.04, E868.05, E868.06, E868.07, E868.08, E868.09, E868.1, E868.10, E868.11, E868.12, E868.13, E868.14, E868.15, E868.16, E868.17, E868.18, E868.19, E868.2, E868.20, E868.21, E868.22, E868.23, E868.24, E868.25, E868.26, E868.27, E868.28, E868.29, E868.3, E868.30, E868.31, E868.32, E868.33, E868.34, E868.35, E868.36, E868.37, E868.38, E868.39, E868.8, E868.80, E868.81, E868.82, E868.83, E868.84, E868.85, E868.86, E868.87, E868.88, E868.89, E868.9, E868.90, E868.91, E868.92, E868.93, E868.94, E868.95, E868.96, E868.97, E868.98, E868.99, E869, E869.9, E869.90, E869.91, E869.92, E869.93, E869.94, E869.95, E869.96, E869.97, E869.98, E869.99
Poisoning by other means		E856, E856.0, E856.00, E856.01, E856.02, E856.03, E856.04, E856.05, E856.06, E856.07, E856.08, E856.09, E857, E857.0, E857.00, E857.01, E857.02, E857.03, E857.04, E857.05, E857.06, E857.07, E857.08, E857.09, E860.2, E860.20, E860.21, E860.22, E860.23, E860.24, E860.25, E860.26, E860.27, E860.28, E860.29, E860.3, E860.30, E860.31, E860.32, E860.33, E860.34, E860.35, E860.36, E860.37, E860.38, E860.39, E860.4, E860.40, E860.41, E860.42, E860.43, E860.44, E860.45, E860.46, E860.47, E860.48, E860.49, E860.8, E860.80, E860.81, E860.82, E860.83, E860.84, E860.85, E860.86, E860.87, E860.88, E860.89, E860.9, E860.90, E860.91, E860.92, E860.93, E860.94, E860.95, E860.96, E860.97, E860.98, E860.99, E861, E861.0, E861.00, E861.01, E861.02, E861.03, E861.04, E861.05, E861.06, E861.07, E861.08, E861.09, E861.1, E861.10, E861.11, E861.12, E861.13, E861.14, E861.15, E861.16, E861.17, E861.18, E861.19, E861.2, E861.20, E861.21, E861.22, E861.23, E861.24, E861.25, E861.26, E861.27, E861.28, E861.29, E861.3, E861.30, E861.31, E861.32, E861.33, E861.34, E861.35, E861.36, E861.37, E861.38, E861.39, E861.4, E861.40, E861.41, E861.42, E861.43, E861.44, E861.45, E861.46, E861.47, E861.48, E861.49, E861.5, E861.50, E861.51, E861.52, E861.53, E861.54, E861.55, E861.56, E861.57, E861.58, E861.59, E861.6, E861.60, E861.61, E861.62, E861.63, E861.64, E861.65, E861.66, E861.67, E861.68, E861.69, E861.9, E861.90, E861.91, E861.92, E861.93, E861.94, E861.95, E861.96, E861.97, E861.98, E861.99, E864, E864.0, E864.00, E864.01, E864.02, E864.03, E864.04, E864.05, E864.06, E864.07, E864.08, E864.09, E864.1, E864.10, E864.11, E864.12, E864.13, E864.14, E864.15, E864.16, E864.17, E864.18, E864.19, E864.2, E864.20, E864.21, E864.22, E864.23, E864.24, E864.25, E864.26, E864.27, E864.28, E864.29, E864.3, E864.30, E864.31, E864.32, E864.33, E864.34, E864.35, E864.36, E864.37, E864.38, E864.39, E864.4, E864.40, E864.41, E864.42, E864.43, E864.44, E864.45, E864.46, E864.47, E864.48, E864.49, E865, E865.0, E865.00, E865.01, E865.02, E865.03, E865.04, E865.05, E865.06, E865.07, E865.08, E865.09, E865.1, E865.10, E865.11, E865.12, E865.13, E865.14, E865.15, E865.16, E865.17, E865.18, E865.19, E865.2, E865.20, E865.21, E865.22, E865.23, E865.24, E865.25, E865.26, E865.27, E865.28, E865.29, E865.3, E865.30, E865.31, E865.32, E865.33, E865.34, E865.35, E865.36, E865.37, E865.38, E865.39, E865.4, E865.40, E865.41, E865.42

Table S4. List of infectious syndromes mapped to International Classification of Diseases (ICD) codes

Cause Name	ICD 10 Codes	ICD 9 Codes
Police conflict and executions	Y35, Y35.0, Y35.00, Y35.001, Y35.002, Y35.003, Y35.01, Y35.011, Y35.012, Y35.013, Y35.02, Y35.021, Y35.022, Y35.023, Y35.03, Y35.031, Y35.032, Y35.033, Y35.04, Y35.041, Y35.042, Y35.043, Y35.09, Y35.091, Y35.092, Y35.093, Y35.1, Y35.10, Y35.101, Y35.102, Y35.103, Y35.11, Y35.111, Y35.112, Y35.113, Y35.12, Y35.121, Y35.122, Y35.123, Y35.19, Y35.191, Y35.192, Y35.193, Y35.2, Y35.20, Y35.201, Y35.202, Y35.203, Y35.21, Y35.211, Y35.212, Y35.213, Y35.29, Y35.291, Y35.292, Y35.293, Y35.3, Y35.30, Y35.301, Y35.302, Y35.303, Y35.31, Y35.311, Y35.312, Y35.313, Y35.39, Y35.391, Y35.392, Y35.393, Y35.4, Y35.40, Y35.401, Y35.402, Y35.403, Y35.41, Y35.411, Y35.412, Y35.413, Y35.49, Y35.491, Y35.492, Y35.493, Y35.5, Y35.6, Y35.7, Y35.8, Y35.81, Y35.811, Y35.812, Y35.813, Y35.89, Y35.891, Y35.892, Y35.893, Y35.9, Y35.91, Y35.92, Y35.93, Y89.0	E970, E971, E972, E973, E974, E975, E976, E977, E978
Prostate cancer	C61, C61.0, C61.9, D07.5, D29.1, D40.0	185, 185.0, 185.9, 222.2, 236.5
Protein-energy malnutrition	E40, E40.0, E41, E41.0, E41.9, E42, E43, E43.0, E43.9, E44, E44.0, E44.1, E45, E45.0, E46, E46.0, E46.9, E64.0	260, 260.0, 260.9, 261, 261.0, 261.9, 262, 262.0, 262.9, 263, 263.0, 263.1, 263.2, 263.4, 263.8, 263.9
Pulmonary Arterial Hypertension	I27.0	416
Pulmonary aspiration and foreign body in airway	W75, W75.0, W75.1, W75.2, W75.3, W75.4, W75.5, W75.6, W75.7, W75.8, W75.9, W78, W78.0, W78.1, W78.2, W78.3, W78.4, W78.5, W78.6, W78.7, W78.8, W78.9, W79, W79.0, W79.1, W79.2, W79.3, W79.4, W79.5, W79.6, W79.7, W79.8, W79.9, W80, W80.0, W80.1, W80.2, W80.3, W80.4, W80.5, W80.6, W80.7, W80.8, W80.9, W83, W83.0, W83.1, W83.2, W83.3, W83.4, W83.5, W83.6, W83.7, W83.8, W83.9, W84, W84.0, W84.1, W84.2, W84.3, W84.4, W84.5, W84.6, W84.7, W84.8, W84.9	E911, E911.0, E911.00, E911.01, E911.02, E911.03, E911.04, E911.05, E911.06, E911.07, E911.08, E911.09, E912, E912.0, E912.00, E912.01, E912.02, E912.03, E912.04, E912.05, E912.06, E912.07, E912.08, E912.09, E913, E913.0, E913.00, E913.01, E913.02, E913.03, E913.04, E913.05, E913.06, E913.07, E913.08, E913.09, E913.1, E913.10, E913.11, E913.12, E913.13, E913.14, E913.15, E913.16, E913.17, E913.18, E913.19, E913.8, E913.80, E913.81, E913.82, E913.83, E913.84, E913.85, E913.86, E913.87, E913.88, E913.89, E913.9, E913.90, E913.91, E913.92, E913.93, E913.94, E913.95, E913.96, E913.97, E913.98, E913.99
Pyoderma	A46, A46.0, A66, A66.0, A66.1, A66.2, A66.3, A66.4, A66.5, A66.6, A66.7, A66.8, A66.9, A67, A67.0, A67.1, A67.2, A67.3, A67.7, A67.9, H05.04, H05.041, H05.042, H05.043, H05.049, H05.1, H05.10, H05.11, H05.111, H05.112, H05.113, H05.119, I89.1, I89.8, L00, L01, L01.0, L01.00, L01.01, L01.02, L01.03, L01.09, L01.1, L02, L02.0, L02.01, L02.02, L02.03, L02.1, L02.11, L02.12, L02.13, L02.2, L02.21, L02.211, L02.212, L02.213, L02.214, L02.215, L02.216, L02.219, L02.22, L02.221, L02.222, L02.223, L02.224, L02.225, L02.226, L02.229, L02.23, L02.231, L02.232, L02.233, L02.234, L02.235, L02.236, L02.239, L02.3, L02.31, L02.32, L02.33, L02.4, L02.41, L02.411, L02.412, L02.413, L02.414, L02.415, L02.416, L02.419, L02.42, L02.421, L02.422, L02.423, L02.424, L02.425, L02.426, L02.429, L02.43, L02.431, L02.432, L02.433, L02.434, L02.435, L02.436, L02.439, L02.5, L02.51, L02.511, L02.512, L02.519, L02.52, L02.521, L02.522, L02.529, L02.53, L02.531, L02.532, L02.539, L02.6, L02.61, L02.611, L02.612, L02.619, L02.62, L02.621, L02.622, L02.629, L02.63, L02.631, L02.632, L02.639, L02.8, L02.81, L02.811, L02.818, L02.82, L02.821, L02.828, L02.83, L02.831, L02.838, L02.9, L02.91, L02.92, L02.93, L04, L04.0, L04.1, L04.2, L04.3, L04.8, L04.9, L05, L05.0, L05.01, L05.02, L05.9, L05.91, L05.92, L08, L08.0, L08.1, L08.8, L08.81, L08.82, L08.89, L08.9, L88, L97, L97.0, L97.1, L97.10, L97.101, L97.102, L97.103, L97.104, L97.109, L97.11, L97.111, L97.112, L97.113, L97.114, L97.119, L97.12, L97.121, L97.122, L97.123, L97.124, L97.129, L97.2, L97.20, L97.201, L97.202, L97.203, L97.204, L97.209, L97.21, L97.211, L97.212, L97.213, L97.214, L97.219, L97.22, L97.221, L97.222, L97.223, L97.224, L97.229, L97.3, L97.30, L97.301, L97.302, L97.303, L97.304, L97.309, L97.31, L97.311, L97.312, L97.313, L97.314, L97.319, L97.32, L97.321, L97.322, L97.323, L97.324, L97.329, L97.4, L97.40, L97.401, L97.402, L97.403, L97.404, L97.409, L97.41, L97.411, L97.412, L97.413, L97.414, L97.419, L97.42, L97.421, L97.422, L97.423, L97.424, L97.429, L97.5, L97.50, L97.501, L97.502, L97.503, L97.504, L97.509, L97.51, L97.511, L97.512, L97.513, L97.514, L97.519, L97.52, L97.521, L97.522, L97.523, L97.524, L97.529, L97.8, L97.80, L97.801, L97.802, L97.803, L97.804, L97.809, L97.81, L97.811, L97.812, L97.813, L97.814, L97.819, L97.82, L97.821, L97.822, L97.823, L97.824, L97.829, L97.9, L97.90, L97.901, L97.902, L97.903, L97.904, L97.909	035, 035.0, 035.1, 035.4, 035.5, 035.8, 035.9, 102, 102.0, 102.1, 102.2, 102.3, 102.4, 102.5, 102.6, 102.7, 102.8, 102.9, 103, 103.0, 103.1, 103.2, 103.3, 103.9, 376.0, 376.00, 376.01, 376.02, 376.03, 376.04, 376.1, 457.2, 457.3, 680, 680.0, 680.1, 680.2, 680.3, 680.4, 680.5, 680.6, 680.7, 680.8, 680.9, 683, 683.0, 683.9, 684, 684.0, 684.9, 685, 685.0, 685.1, 685.7, 685.9, 686, 686.0, 686.00, 686.01, 686.09, 686.1, 686.8, 686.9, 687, 688, 689
Rabies	A82, A82.0, A82.1, A82.9	071, 071.0, 071.1, 071.4, 071.5, 071.6, 071.7, 071.8, 071.9
Retinoblastoma	C69.2, C69.20, C69.21, C69.22	190.5
Rheumatic heart disease	I01, I01.0, I01.1, I01.2, I01.8, I01.9, I02.0, I05, I05.0, I05.1, I05.2, I05.8, I05.9, I06, I06.0, I06.1, I06.2, I06.8, I06.9, I07, I07.0, I07.1, I07.2, I07.8, I07.9, I08, I08.0, I08.1, I08.2, I08.3, I08.8, I08.9, I09, I09.0, I09.1, I09.2, I09.8, I09.81, I09.89, I09.9	391, 391.0, 391.1, 391.2, 391.4, 391.8, 391.9, 392.0, 393, 393.0, 393.9, 394, 394.0, 394.1, 394.2, 394.5, 394.9, 395, 395.0, 395.1, 395.2, 395.9, 396, 396.0, 396.1, 396.2, 396.3, 396.8, 396.9, 397, 397.0, 397.1, 397.9, 398, 398.0, 398.8, 398.9, 398.90, 398.91, 398.99

Table S4. List of infectious syndromes mapped to International Classification of Diseases (ICD) codes

Cause Name	ICD 10 Codes	ICD 9 Codes
Rheumatoid arthritis	M05, M05.0, M05.00, M05.01, M05.011, M05.012, M05.019, M05.02, M05.021, M05.022, M05.029, M05.03, M05.031, M05.032, M05.039, M05.04, M05.041, M05.042, M05.049, M05.05, M05.051, M05.052, M05.059, M05.06, M05.061, M05.062, M05.069, M05.07, M05.071, M05.072, M05.079, M05.09, M05.1, M05.10, M05.11, M05.111, M05.112, M05.119, M05.12, M05.121, M05.122, M05.129, M05.13, M05.131, M05.132, M05.139, M05.14, M05.141, M05.142, M05.149, M05.15, M05.151, M05.152, M05.159, M05.16, M05.161, M05.162, M05.169, M05.17, M05.171, M05.172, M05.179, M05.19, M05.2, M05.20, M05.21, M05.211, M05.212, M05.219, M05.22, M05.221, M05.222, M05.229, M05.23, M05.231, M05.232, M05.239, M05.24, M05.241, M05.242, M05.249, M05.25, M05.251, M05.252, M05.259, M05.26, M05.261, M05.262, M05.269, M05.27, M05.271, M05.272, M05.279, M05.29, M05.3, M05.30, M05.31, M05.311, M05.312, M05.319, M05.32, M05.321, M05.322, M05.329, M05.33, M05.331, M05.332, M05.339, M05.34, M05.341, M05.342, M05.349, M05.35, M05.351, M05.352, M05.359, M05.36, M05.361, M05.362, M05.369, M05.37, M05.371, M05.372, M05.379, M05.39, M05.4, M05.40, M05.41, M05.411, M05.412, M05.419, M05.42, M05.421, M05.422, M05.429, M05.43, M05.431, M05.432, M05.439, M05.44, M05.441, M05.442, M05.449, M05.45, M05.451, M05.452, M05.459, M05.46, M05.461, M05.462, M05.469, M05.47, M05.471, M05.472, M05.479, M05.49, M05.5, M05.50, M05.51, M05.511, M05.512, M05.519, M05.52, M05.521, M05.522, M05.529, M05.53, M05.531, M05.532, M05.539, M05.54, M05.541, M05.542, M05.549, M05.55, M05.551, M05.552, M05.559, M05.56, M05.561, M05.562, M05.569, M05.57, M05.571, M05.572, M05.579, M05.59, M05.6, M05.60, M05.61, M05.611, M05.612, M05.619, M05.62, M05.621, M05.622, M05.629, M05.63, M05.631, M05.632, M05.639, M05.64, M05.641, M05.642, M05.649, M05.65, M05.651, M05.652, M05.659, M05.66, M05.661, M05.662, M05.669, M05.67, M05.671, M05.672, M05.679, M05.69, M05.7, M05.70, M05.71, M05.711, M05.712, M05.719, M05.72, M05.721, M05.722, M05.729, M05.73, M05.731, M05.732, M05.739, M05.74, M05.741, M05.742, M05.749, M05.75, M05.751, M05.752, M05.759, M05.76, M05.761, M05.762, M05.769, M05.77, M05.771, M05.772, M05.779, M05.79, M05.8, M05.80, M05.81, M05.811, M05.812, M05.819, M05.82, M05.821, M05.822, M05.829, M05.83, M05.831, M05.832, M05.839, M05.84	714, 714.0, 714.1, 714.2, 714.3, 714.30, 714.31, 714.32, 714.33, 714.8, 714.81, 714.89, 714.9
Road injuries		
Schistosomiasis	B65, B65.0, B65.1, B65.2, B65.3, B65.8, B65.9	120, 120.0, 120.1, 120.2, 120.3, 120.4, 120.5, 120.6, 120.8, 120.9
Self-harm by firearm	X72, X72.0, X72.1, X72.2, X72.3, X72.4, X72.5, X72.6, X72.7, X72.8, X72.9, X73, X73.0, X73.1, X73.2, X73.3, X73.4, X73.5, X73.6, X73.7, X73.8, X73.9, X74, X74.0, X74.01, X74.02, X74.09, X74.1, X74.2, X74.3, X74.4, X74.5, X74.6, X74.7, X74.8, X74.9	E955, E955.0, E955.1, E955.2, E955.3, E955.4, E955.5, E955.6, E955.7, E955.9
Self-harm by other specified means	X60, X60.0, X60.1, X60.2, X60.3, X60.4, X60.5, X60.6, X60.7, X60.8, X60.9, X61, X61.0, X61.1, X61.2, X61.3, X61.4, X61.5, X61.6, X61.7, X61.8, X61.9, X62, X62.0, X62.1, X62.2, X62.3, X62.4, X62.5, X62.6, X62.7, X62.8, X62.9, X63, X63.0, X63.1, X63.2, X63.3, X63.4, X63.5, X63.6, X63.7, X63.8, X63.9, X64, X64.0, X64.1, X64.2, X64.3, X64.4, X64.5, X64.6, X64.7, X64.8, X64.9, X66, X66.0, X66.1, X66.2, X66.3, X66.4, X66.5, X66.6, X66.7, X66.8, X66.9, X67, X67.0, X67.1, X67.2, X67.3, X67.4, X67.5, X67.6, X67.7, X67.8, X67.9, X69, X69.0, X69.1, X69.2, X69.3, X69.4, X69.5, X69.6, X69.7, X69.8, X69.9, X70, X70.0, X70.1, X70.2, X70.3, X70.4, X70.5, X70.6, X70.7, X70.8, X70.9, X71, X71.0, X71.1, X71.2, X71.3, X71.4, X71.5, X71.6, X71.7, X71.8, X71.9, X75, X75.0, X75.1, X75.2, X75.3, X75.4, X75.5, X75.6, X75.7, X75.8, X75.9, X77, X77.0, X77.1, X77.2, X77.3, X77.4, X77.5, X77.6, X77.7, X77.8, X77.9, X78, X78.0, X78.1, X78.2, X78.3, X78.4, X78.5, X78.6, X78.7, X78.8, X78.9, X79, X79.0, X79.1, X79.2, X79.3, X79.4, X79.5, X79.6, X79.7, X79.8, X79.9, X80, X80.0, X80.1, X80.2, X80.3, X80.4, X80.5, X80.6, X80.7, X80.8, X80.9, X81, X81.0, X81.1, X81.2, X81.3, X81.4, X81.5, X81.6, X81.7, X81.8, X81.9, X82, X82.0, X82.1, X82.2, X82.3, X82.4, X82.5, X82.6, X82.7, X82.8, X82.9, X83, X83.0, X83.1, X83.2, X83.3, X83.4, X83.5, X83.6, X83.7, X83.8, X83.9, Y87.0	E950, E950.0, E950.1, E950.2, E950.3, E950.4, E950.5, E950.6, E950.7, E950.8, E950.9, E951, E951.0, E951.1, E951.8, E952, E952.0, E952.1, E952.8, E952.9, E954, E956, E957, E957.0, E957.1, E957.2, E957.9, E958, E958.0, E958.2, E958.3, E958.4, E958.5, E958.6, E958.7, E958.8, E958.9, E959
Sickle cell disorders	D57, D57.0, D57.00, D57.01, D57.02, D57.1, D57.2, D57.20, D57.21, D57.211, D57.212, D57.219, D57.3, D57.4, D57.40, D57.41, D57.411, D57.412, D57.419, D57.8, D57.80, D57.81, D57.811, D57.812, D57.819	282.6, 282.61, 282.62, 282.63, 282.64, 282.68
Soft tissue and other extraosseous sarcomas	C49, C49.0, C49.1, C49.10, C49.11, C49.12, C49.2, C49.20, C49.21, C49.22, C49.3, C49.4, C49.5, C49.6, C49.8, C49.9	171, 171.0, 171.2, 171.3, 171.4, 171.5, 171.6, 171.7, 171.8, 171.9
Stomach cancer	C16, C16.0, C16.1, C16.2, C16.3, C16.4, C16.5, C16.6, C16.7, C16.8, C16.9, D00.2, D13.1, D37.1	151, 151.0, 151.1, 151.2, 151.3, 151.4, 151.5, 151.6, 151.8, 151.9, 209.23, 209.63, 211.1, 230.2

Table S4. List of infectious syndromes mapped to International Classification of Diseases (ICD) codes		
**Cause Name**	**ICD 10 Codes**	**ICD 9 Codes**
Subarachnoid hemorrhage	I60, I60.0, I60.00, I60.01, I60.02, I60.1, I60.10, I60.11, I60.12, I60.2, I60.20, I60.21, I60.22, I60.3, I60.30, I60.31, I60.32, I60.4, I60.5, I60.50, I60.51, I60.52, I60.6, I60.7, I60.8, I60.9, I62.0, I62.00, I62.01, I62.02, I62.03, I67.0, I67.1, I69.0, I69.00, I69.01, I69.02, I69.020, I69.021, I69.022, I69.023, I69.028, I69.03, I69.031, I69.032, I69.033, I69.034, I69.039, I69.04, I69.041, I69.042, I69.043, I69.044, I69.049, I69.05, I69.051, I69.052, I69.053, I69.054, I69.059, I69.06, I69.061, I69.062, I69.063, I69.064, I69.065, I69.069, I69.09, I69.090, I69.091, I69.092, I69.093, I69.098	430, 430.0, 430.1, 430.4, 430.6, 430.9
Syphilis	A50, A50.0, A50.01, A50.02, A50.03, A50.04, A50.05, A50.06, A50.07, A50.08, A50.09, A50.1, A50.2, A50.3, A50.30, A50.31, A50.32, A50.39, A50.4, A50.40, A50.41, A50.42, A50.43, A50.44, A50.45, A50.49, A50.5, A50.51, A50.52, A50.53, A50.54, A50.55, A50.56, A50.57, A50.59, A50.6, A50.7, A50.9, A51, A51.0, A51.1, A51.2, A51.3, A51.31, A51.32, A51.39, A51.4, A51.41, A51.42, A51.43, A51.44, A51.45, A51.46, A51.49, A51.5, A51.9, A52, A52.0, A52.00, A52.01, A52.02, A52.03, A52.04, A52.05, A52.06, A52.09, A52.1, A52.10, A52.11, A52.12, A52.13, A52.14, A52.15, A52.16, A52.17, A52.19, A52.2, A52.3, A52.7, A52.71, A52.72, A52.73, A52.74, A52.75, A52.76, A52.77, A52.78, A52.79, A52.8, A52.9, A53, A53.0, A53.9, I98.0, K67.2, M03.1, M73.1	090, 090.0, 090.1, 090.2, 090.3, 090.4, 090.40, 090.41, 090.42, 090.49, 090.5, 090.6, 090.7, 090.8, 090.9, 091, 091.0, 091.1, 091.2, 091.3, 091.4, 091.5, 091.50, 091.51, 091.52, 091.6, 091.61, 091.62, 091.69, 091.7, 091.8, 091.81, 091.82, 091.89, 091.9, 092, 092.0, 092.4, 092.5, 092.7, 092.8, 092.9, 093, 093.0, 093.1, 093.2, 093.20, 093.21, 093.22, 093.23, 093.24, 093.5, 093.8, 093.81, 093.82, 093.89, 093.9, 094, 094.0, 094.1, 094.2, 094.3, 094.8, 094.81, 094.82, 094.83, 094.84, 094.85, 094.86, 094.87, 094.89, 094.9, 095, 095.0, 095.1, 095.2, 095.3, 095.4, 095.5, 095.6, 095.7, 095.8, 095.9, 096, 096.0, 096.3, 096.4, 096.5, 096.6, 096.8, 097, 097.0, 097.1, 097.2, 097.3, 097.9
Testicular cancer	C62, C62.0, C62.00, C62.01, C62.02, C62.1, C62.10, C62.11, C62.12, C62.9, C62.90, C62.91, C62.92, D29.2, D29.20, D29.21, D29.22, D29.3, D29.30, D29.31, D29.32, D29.4, D29.7, D29.8, D40.1, D40.10, D40.11, D40.12, D40.7, D40.8	186, 186.0, 186.9, 222.0, 222.3, 236.4
Tetanus	A33, A33.0, A34, A34.0, A35, A35.0	037, 037.0, 037.4, 037.6, 037.8, 037.9, 771.3
Thalassemias	D56, D56.0, D56.1, D56.2, D56.3, D56.4, D56.5, D56.8, D56.9	282.4, 282.41, 282.42, 282.49, 282.5, 282.44
Thyroid cancer	C73, C73.0, C73.1, C73.2, C73.3, C73.4, C73.5, C73.8, C73.9, D09.3, D09.8, D34, D34.0, D34.9, D44.0	193, 193.0, 193.2, 193.9, 226, 226.0, 226.9
Thyroid diseases		
Tracheal, bronchus, and lung cancer	C33, C33.0, C33.2, C33.9, C34, C34.0, C34.00, C34.01, C34.02, C34.1, C34.10, C34.11, C34.12, C34.2, C34.3, C34.30, C34.31, C34.32, C34.4, C34.7, C34.8, C34.80, C34.81, C34.82, C34.9, C34.90, C34.91, C34.92, D02.1, D02.2, D02.20, D02.21, D02.22, D02.3, D14.2, D14.3, D14.30, D14.31, D14.32, D38.1	162, 162.0, 162.1, 162.2, 162.3, 162.4, 162.5, 162.8, 162.9, 209.21, 209.61, 212.2, 212.3, 231.1, 231.2, 235.7
Tuberculosis		
Typhoid fever	A01.0, A01.00, A01.01, A01.02, A01.03, A01.04, A01.05, A01.09	002.0
Ulcerative colitis	K51, K51.0, K51.00, K51.01, K51.011, K51.012, K51.013, K51.014, K51.018, K51.019, K51.1, K51.2, K51.20, K51.21, K51.211, K51.212, K51.213, K51.214, K51.218, K51.219, K51.3, K51.30, K51.31, K51.311, K51.312, K51.313, K51.314, K51.318, K51.319, K51.4, K51.40, K51.41, K51.411, K51.412, K51.413, K51.414, K51.418, K51.419, K51.5, K51.50, K51.51, K51.511, K51.512, K51.513, K51.514, K51.518, K51.519, K51.8, K51.80, K51.81, K51.811, K51.812, K51.813, K51.814, K51.818, K51.819, K51.9, K51.90, K51.91, K51.911, K51.912, K51.913, K51.914, K51.918, K51.919, K52, K52.8, K52.81, K52.82, K52.89, K52.9	556, 556.0, 556.1, 556.2, 556.3, 556.4, 556.5, 556.6, 556.8, 556.9, 558.0
Unintentional firearm injuries	W32, W32.0, W32.1, W32.2, W32.3, W32.4, W32.5, W32.6, W32.7, W32.8, W32.9, W33, W33.0, W33.00, W33.01, W33.02, W33.03, W33.09, W33.1, W33.10, W33.11, W33.12, W33.13, W33.19, W33.2, W33.3, W33.4, W33.5, W33.6, W33.7, W33.8, W33.9, W34, W34.0, W34.00, W34.01, W34.010, W34.011, W34.018, W34.09, W34.1, W34.10, W34.11, W34.110, W34.111, W34.118, W34.19, W34.2, W34.3, W34.4, W34.5, W34.6, W34.7, W34.8, W34.9	E922, E922.0, E922.00, E922.01, E922.02, E922.03, E922.04, E922.05, E922.06, E922.07, E922.08, E922.09, E922.1, E922.10, E922.11, E922.12, E922.13, E922.14, E922.15, E922.16, E922.17, E922.18, E922.19, E922.2, E922.20, E922.21, E922.22, E922.23, E922.24, E922.25, E922.26, E922.27, E922.28, E922.29, E922.3, E922.30, E922.31, E922.32, E922.33, E922.34, E922.35, E922.36, E922.37, E922.38, E922.39, E922.4, E922.5, E922.8, E922.80, E922.81, E922.82, E922.83, E922.84, E922.85, E922.86, E922.87, E922.88, E922.89, E922.9, E922.90, E922.91, E922.92, E922.93, E922.94, E922.95, E922.96, E922.97, E922.98, E922.99, E928.7
Upper respiratory infections	J00, J00.0, J01, J01.0, J01.00, J01.01, J01.1, J01.10, J01.11, J01.2, J01.20, J01.21, J01.3, J01.30, J01.31, J01.4, J01.40, J01.41, J01.8, J01.80, J01.81, J01.9, J01.90, J01.91, J02, J02.0, J02.8, J03, J03.0, J03.00, J03.01, J03.8, J03.80, J03.81, J04, J04.0, J04.1, J04.10, J04.11, J04.2, J05, J05.0, J05.1, J05.10, J05.11, J06.0, J06.8, J36, J36.0	034.0, 460, 460.0, 460.9, 461, 461.0, 461.1, 461.2, 461.3, 461.8, 461.9, 462, 462.0, 462.9, 463, 463.0, 463.9, 464, 464.0, 464.00, 464.01, 464.1, 464.10, 464.11, 464.2, 464.20, 464.21, 464.3, 464.30, 464.31, 464.4, 464.8, 464.9, 465.0, 465.1, 465.8, 475, 475.0, 475.9, 476.9

Table S4. List of infectious syndromes mapped to International Classification of Diseases (ICD) codes

Cause Name	ICD 10 Codes	ICD 9 Codes
Urinary tract infections and interstitial nephritis	N10, N10.0, N10.9, N11, N11.0, N11.1, N11.8, N11.9, N12, N12.0, N12.9, N13.6, N15, N15.1, N15.8, N15.9, N16, N16.0, N16.1, N16.2, N16.3, N16.4, N16.5, N16.8, N30, N30.0, N30.00, N30.01, N30.1, N30.10, N30.11, N30.2, N30.20, N30.21, N30.3, N30.30, N30.31, N30.8, N30.80, N30.81, N30.9, N30.90, N30.91, N34, N34.0, N34.1, N34.2, N34.3, N39.0, N39.1, N39.2	590, 590.0, 590.00, 590.01, 590.1, 590.10, 590.11, 590.2, 590.3, 590.8, 590.80, 590.81, 590.9, 595, 595.0, 595.1, 595.2, 595.3, 595.4, 595.8, 595.81, 595.89, 595.9, 597, 597.0, 597.8, 597.80, 597.81, 597.89, 597.9, 599.0
Urogenital congenital anomalies	P96.0, Q50, Q50.0, Q50.01, Q50.02, Q50.1, Q50.2, Q50.3, Q50.31, Q50.32, Q50.39, Q50.4, Q50.5, Q50.6, Q51, Q51.0, Q51.1, Q51.10, Q51.11, Q51.2, Q51.3, Q51.4, Q51.5, Q51.6, Q51.7, Q51.8, Q51.81, Q51.810, Q51.811, Q51.818, Q51.82, Q51.820, Q51.821, Q51.828, Q51.9, Q52, Q52.0, Q52.1, Q52.10, Q52.11, Q52.12, Q52.2, Q52.3, Q52.4, Q52.5, Q52.6, Q52.7, Q52.70, Q52.71, Q52.79, Q52.8, Q52.9, Q53, Q53.0, Q53.00, Q53.01, Q53.02, Q53.1, Q53.10, Q53.11, Q53.12, Q53.2, Q53.20, Q53.21, Q53.22, Q53.9, Q54, Q54.0, Q54.1, Q54.2, Q54.3, Q54.4, Q54.8, Q54.9, Q55, Q55.0, Q55.1, Q55.2, Q55.20, Q55.21, Q55.22, Q55.23, Q55.29, Q55.3, Q55.4, Q55.5, Q55.6, Q55.61, Q55.62, Q55.63, Q55.64, Q55.69, Q55.7, Q55.8, Q55.9, Q56, Q56.0, Q56.1, Q56.2, Q56.3, Q56.4, Q60, Q60.0, Q60.1, Q60.2, Q60.3, Q60.4, Q60.5, Q60.6, Q63, Q63.0, Q63.1, Q63.2, Q63.3, Q63.8, Q63.9, Q64, Q64.0, Q64.1, Q64.10, Q64.11, Q64.12, Q64.19, Q64.2, Q64.3, Q64.31, Q64.32, Q64.33, Q64.39, Q64.4, Q64.5, Q64.6, Q64.7, Q64.70, Q64.71, Q64.72, Q64.73, Q64.74, Q64.75, Q64.79, Q64.8, Q64.9, Q57	752, 752.0, 752.1, 752.10, 752.11, 752.19, 752.2, 752.3, 752.4, 752.40, 752.41, 752.42, 752.49, 752.5, 752.51, 752.52, 752.6, 752.61, 752.62, 752.63, 752.65, 752.69, 752.7, 752.8, 752.89, 752.9, 753.4, 753.5, 753.6, 753.7, 753.8, 753.9
Urolithiasis	N20, N20.0, N20.1, N20.2, N20.9, N21, N21.0, N21.1, N21.8, N21.9, N22, N22.0, N22.8, N23, N23.0	592, 592.0, 592.1, 592.9, 594, 594.0, 594.1, 594.2, 594.8, 594.9, 788.0
Uterine cancer	C54, C54.0, C54.1, C54.2, C54.3, C54.4, C54.8, C54.9, D07.0, D07.1, D07.2, D26.1, D26.7, D26.9	182, 182.0, 182.1, 182.8, 182.9, 233.2
Uterine fibroids	D25, D25.0, D25.1, D25.2, D25.9, D26, D28.2	218, 218.0, 218.1, 218.2, 218.9, 219, 219.1, 219.8, 219.9, 236.0
Vascular intestinal disorders	K55, K55.0, K55.1, K55.2, K55.20, K55.21, K55.6, K55.8, K55.9	557, 557.0, 557.1, 557.9
Venomous animal contact		E905
Yellow fever	A95, A95.0, A95.1, A95.9	060, 060.0, 060.1, 060.2, 060.3, 060.4, 060.5, 060.6, 060.7, 060.8, 060.9
Zika virus	U06, U06.9	
