# Supplementary material for: Global burden of bacterial antimicrobial resistance 1990–2021: a systematic analysis with forecasts to 2050
Source: Lancet. 2024 Sep 28;404(10459):1199–226. doi: 10.1016/S0140-6736(24)01867-1 (PMC11718157; doi:10.1016/S0140-6736(24)01867-1)
Supplement: Supplementary appendix 3 [file mmc3.pdf]

THE LANCET

Supplementary appendix 3

This appendix formed part of the original submission and has been peer reviewed. We post it as supplied by the authors.

Supplement to: GBD 2021 Antimicrobial Resistance Collaborators. Global burden of bacterial antimicrobial resistance 1990–2021: a systematic analysis with forecasts to 2050. *Lancet* 2024; published online Sept 16. https://doi.org/10.1016/S0140-6736(24)01867-1.

Appendix 3: Authorship appendix to "Global burden of bacterial antimicrobial resistance 1990–2021: a systematic analysis with forecasts to 2050"

This appendix provides further authorship detail for "Global burden of bacterial antimicrobial resistance 1990–2021: a systematic analysis with forecasts to 2050"

Table of Contents

GBD 2021 Global Antimicrobial Resistance Collaborators **2**

Affiliations **4**

Authors' Contributions **17**

Managing the overall research enterprise 17

Writing the first draft of the manuscript 18

Primary responsibility for applying analytical methods to produce estimates 18

Primary responsibility for seeking, cataloguing, extracting, or cleaning data; designing or coding figures and tables 18

Providing data or critical feedback on data sources 18

Developing methods or computational machinery 19

Providing critical feedback on methods or results 20

Drafting the work or revising it critically for important intellectual content 21

Managing the estimation or publications process 23

GBD 2021 Global Antimicrobial Resistance Collaborators

Mohsen Naghavi*, Stein Emil Vollset*, Kevin S Ikuta*, Lucien R Swetschinski, Authia P Gray, Eve E Wool, Gisela Robles Aguilar, Tomislav Mestrovic, Georgia Smith, Chieh Han, Rebecca L Hsu, Julian Chalek, Daniel T Araki, Erin Chung, Catalina Raggi, Anna Gershberg Hayoon, Nicole Davis Weaver, Paulina A Lindstedt, Amanda E Smith, Umut Altay, Natalia V Bhattacharjee, Konstantinos Giannakis, Frederick Fell, Barney McManigal, Nattwut Ekapirat, Jessica Andretta Mendes, Tilleye Runghien, Oraya Srimokla, Atef Abdelkader, Sherief Abd-Elsalam, Richard Gyan Aboagye, Hassan Abolhassani, Hasan Abualruz, Usman Abubakar, Hana J Abukhadijah, Salahdein Aburuz, Ahmed Abu-Zaid, Sureerak Achalapong, Isaac Yeboah Addo, Victor Adekanmbi, Temitayo Esther Adeyeoluwa, Qorinah Estiningtyas Sakilah Adnani, Leticia Akua Adzigbli, Muhammad Sohail Afzal, Saira Afzal, Antonella Agodi, Austin J Ahlstrom, Aqeel Ahmad, Sajjad Ahmad, Tauseef Ahmad, Ali Ahmadi, Ayman Ahmed, Haroon Ahmed, Ibrar Ahmed, Mohammed Ahmed, Saeed Ahmed, Syed Anees Ahmed, Mohammed Ahmed Akkaif, Salah Al Awaidy, Yazan Al Thaher, Samer O Alalalmeh, Mohammad T AlBataineh, Wafa A Aldhaleei, Adel Ali Saeed Al-Gheethi, Nma Bida Alhaji, Abid Ali, Liaqat Ali, Syed Shujait Ali, Waad Ali, Kasim Allel, Sabah Al-Marwani, Ahmad Alrawashdeh, Awais Altaf, Alaa B Al-Tammemi, Jaffar A Al-Tawfiq, Karem H Alzoubi, Walid Adnan Al-Zyoud, Ben Amos, John H Amuasi, Robert Ancuceanu, Jason R Andrews, Abhishek Anil, Iyadunni Adesola Anuoluwa, Saeid Anvari, Anayochukwu Edward Anyasodor, Geminn Louis Carace Apostol, Jalal Arabloo, Mosab Arafat, Aleksandr Y Aravkin, Demelash Areda, Abdulfatai Aremu, Anton A Artamonov, Elizabeth A Ashley, Marvellous O Asika, Seyyed Shamsadin Athari, Maha Moh'd Wahbi Atout, Tewachew Awoke, Sina Azadnajafabad, James Mba Azam, Shahkaar Aziz, Ahmed Y Azzam, Mahsa Babaei, Francois-Xavier Babin, Muhammad Badar, Atif Amin Baig, Milica Bajcetic, Stephen Baker, Mainak Bardhan, Hiba Jawdat Barqawi, Zarrin Basharat, Afisu Basiru, Mathieu Bastard, Saurav Basu, Nebiyou Simegnew Bayleyegn, Melaku Ashagrie Belete, Olorunjuwon Omolaja Bello, Apostolos Beloukas, James A Berkley, Akshaya Srikanth Bhagavathula, Sonu Bhaskar, Soumitra S Bhuyan, Julia A Bielicki, Nikolay Ivanovich Briko, Colin Stewart Brown, Annie J Browne, Danilo Buonsenso, Yasser Bustanji, Cristina G Carvalheiro, Carlos A Castañeda-Orjuela, Muthia Cenderadewi, Joshua Chadwick, Sandip Chakraborty, Rama Mohan Chandika, Sara Chandy, Vilada Chansamouth, Vijay Kumar Chattu, Anis Ahmad Chaudhary, Patrick R Ching, Hitesh Chopra, Fazle Rabbi Chowdhury, Dinh-Toi Chu, Muhammad Chutiyami, Natalia Cruz-Martins, Alanna Gomes da Silva, Omid Dadras, Xiaochen Dai, Samuel D Darcho, Saswati Das, Fernando Pio De la Hoz, Denise Myriam Dekker, Kuldeep Dhama, Daniel Diaz, Benjamin Felix Rothschild Dickson, Serge Ghislain Djorie, Milad Dodangeh, Sushil Dohare, Klara Georgieva Dokova, Ojas Prakashbhai Doshi, Robert Kokou Dowou, Haneil Larson Dsouza, Susanna J Dunachie, Arkadiusz Marian Dziedzic, Tim Eckmanns, Abdelaziz Ed-Dra, Aziz Eftekharimehrabad, Temitope Cyrus Ekundayo, Iman El Sayed, Muhammed Elhadi, Waseem El-Huneidi, Christelle Elias, Sally J Ellis, Randa Elsheikh, Ibrahim Elsohaby, Chadi Eltaha, Babak Eshrati, Majid Eslami, David William Eyre, Adewale Oluwaseun Fadaka, Adeniyi Francis Fagbamigbe, Ayesha Fahim, Aliasghar Fakhri-Demeshghieh, Folorunso Oludayo Fasina, Modupe Margaret Fasina, Ali Fatehizadeh, Nicholas A Feasey, Alireza Feizkhah, Ginenus Fekadu, Florian Fischer, Ida Fitriana, Karen M Forrest, Celia Fortuna Rodrigues, John E Fuller, Muktar A Gadanya, Márió Gajdács, Aravind P Gandhi, Esteban E Garcia-Gallo, Denise O Garrett, Rupesh K Gautam, Miglas Welay Gebregergis, Mesfin Gebrehiwot, Teferi Gebru Gebremeskel, Christine Geffers, Leonidas Georgalis, Ramy Mohamed Ghazy, Mahaveer Golechha, Davide Golinelli, Melita Gordon, Snigdha Gulati, Rajat Das Gupta, Sapna Gupta, Vijai Kumar Gupta, Awoke Derbie Habteyohannes, Sebastian Haller, Harapan Harapan, Michelle L Harrison, Ahmed I Hasaballah, Ikramul Hasan, Rumina Syeda Hasan, Hamidreza Hasani, Andrea Haekyung Haselbeck, Md Saquib Hasnain, Ikrama Ibrahim Hassan, Shoaib Hassan, Mahgol Sadat Hassan

Zadeh Tabatabaei, Khezar Hayat, Jiawei He, Omar E Hegazi, Mohammad Heidari, Kamal Hezam, Ramesh Holla, Marianne Holm, Heidi Hopkins, Md Mahbub Hossain, Mehdi Hosseinzadeh, Sorin Hostiuc, Nawfal R Hussein, Le Duc Huy, Elsa D Ibáñez-Prada, Adalia Ikiroma, Irena M Ilic, Sheikh Mohammed Shariful Islam, Faisal Ismail, Nahlah Elkudssiah Ismail, Chidozie Declan Iwu, Chinwe Juliana Iwu-Jaja, Abdollah Jafarzadeh, Fatoumatta Jaiteh, Reza Jalilzadeh Yengejeh, Roland Dominic G Jamora, Javad Javidnia, Talha Jawaid, Adam W J Jenney, Hyon Jin Jeon, Mohammad Jokar, Nabi Jomehzadeh, Tamas Joo, Nitin Joseph, Zul Kamal, Kehinde Kazeem Kanmodi, Rami S Kantar, James Apollo Kapisi, Ibraheem M Karaye, Yousef Saleh Khader, Himanshu Khajuria, Nauman Khalid, Faham Khamesipour, Ajmal Khan, Mohammad Jobair Khan, Muhammad Tariq Khan, Vishnu Khanal, Feriha Fatima Khidri, Jagdish Khubchandani, Suwimon Khusuwan, Min Seo Kim, Adnan Kisa, Vladimir Andreevich Korshunov, Fiorella Krapp, Ralf Krumkamp, Mohammed Kuddus, Mukhtar Kulimbet, Dewesh Kumar, Emmanuelle A P Kumaran, Ambily Kuttikkattu, Hmwe Hmwe Kyu, Iván Landires, Basira Kankia Lawal, Thao Thi Thu Le, Ingeborg Maria Lederer, Munjae Lee, Seung Won Lee, Alain Lepape, Temesgen Leka Lerango, Virendra S Ligade, Cherry Lim, Stephen S Lim, Liknaw Workie Limenh, Chaojie Liu, Xiaofeng Liu, Xuefeng Liu, Michael J Loftus, Hawraz Ibrahim M Amin, Kelsey Lynn Maass, Sandeep B Maharaj, Mansour Adam Mahmoud, Panagiota Maikanti-Charalampous, Omar M Makram, Kashish Malhotra, Ahmad Azam Malik, Georgia D Mandilara, Florian Marks, Bernardo Alfonso Martinez-Guerra, Miquel Martorell, Hossein Masoumi-Asl, Alexander G Mathioudakis, Juergen May, Theresa A McHugh, James Meiring, Hadush Negash Meles, Addisu Melese, Endalkachew Belayneh Melese, Giuseppe Minervini, Nouh Saad Mohamed, Shafiu Mohammed, Syam Mohan, Ali H Mokdad, Lorenzo Monasta, AmirAli Moodi Ghalibaf, Catrin E Moore, Yousef Moradi, Elias Mossialos, Vincent Mougin, George Duke Mukoro, Francesk Mulita, Berit Muller-Pebody, Efren Murillo-Zamora, Sani Musa, Patrick Musicha, Lillian A Musila, Saravanan Muthupandian, Ahamarshan Jayaraman Nagarajan, Pirouz Naghavi, Firzan Nainu, Tapas Sadasivan Nair, Hastyar Hama Rashid Najmuldeen, Zuhair S Natto, Javaid Nauman, Biswa Prakash Nayak, G Takop Nchanji, Pacifique Ndishimye, Ionut Negoi, Ruxandra Irina Negoi, Seyed Aria Nejadghaderi, QuynhAnh P Nguyen, Efaq Ali Noman, Davis C Nwakanma, Seamus O'Brien, Theresa J Ochoa, Ismail A Odetokun, Oluwaseun Adeolu Ogundijo, Tolulope R Ojo-Akosile, Sylvester Reuben Okeke, Osaretin Christabel Okonji, Andrew T Olagunju, Antonio Olivas-Martinez, Abdulhakeem Abayomi Olorukooba, Peter Olwoch, Kenneth Ikenna Onyedibe, Edgar Ortiz-Brizuela, Olayinka Osuolale, Pradthana Ounchanum, Oyetunde T Oyeyemi, Mahesh Padukudru P A, Jose L Paredes, Romil R Parikh, Jay Patel, Shankargouda Patil, Shrikant Pawar, Anton Y Peleg, Prince Peprah, João Perdigão, Carlo Perrone, Ionela-Roxana Petcu, Koukeo Phommasone, Zahra Zahid Piracha, Dimitri Poddighe, Andrew J Pollard, Ramesh Poluru, Alfredo Ponce-De-Leon, Jagadeesh Puvvula, Farah Naz Qamar, Nameer Hashim Qasim, Clotaire Donatien Rafai, Pankaja Raghav, Leila Rahbarnia, Fakher Rahim, Vafa Rahimi-Movaghar, Mosiur Rahman, Muhammad Aziz Rahman, Hazem Ramadan, Shakthi Kumaran Ramasamy, Pushkal Sinduvadi Ramesh, Pramod W Ramteke, Rishabh Kumar Rana, Usha Rani, Mohammad-Mahdi Rashidi, Devarajan Rathish, Sayaphet Rattanavong, Salman Rawaf, Elrashdy Moustafa Mohamed Redwan, Luis Felipe Reyes, Tamalee Roberts, Julie V Robotham, Victor Daniel Rosenthal, Allen Guy Ross, Nitai Roy, Kristina E Rudd, Cameron John Sabet, Basema Ahmad Saddik, Mohammad Reza Saeb, Umar Saeed, Sahar Saeedi Moghaddam, Weeravoot Saengchan, Mohsen Safaei, Amene Saghazadeh, Narjes Saheb Sharif-Askari, Amirhossein Sahebkar, Soumya Swaroop Sahoo, Maitreyi Sahu, Morteza Saki, Nasir Salam, Zikria Saleem, Mohamed A Saleh, Yoseph Leonardo Samodra, Abdallah M Samy, Aswini Saravanan, Maheswar Satpathy, Austin E Schumacher, Mansour Sedighi, Samroeng Seekaew, Mahan Shafie, Pritik A Shah, Samiah Shahid, Moyad Jamal Shahwan, Sadia Shakoor, Noga Shalev, Muhammad Aaqib Shamim, Mohammad Ali Shamshirgaran, Anas Shamsi, Amin Sharifan,

Rajesh P Shastry, Mahabalesh Shetty, Aminu Shittu, Sunil Shrestha, Emmanuel Edwar Siddig, Theologia Sideroglou, Jose Sifuentes-Osornio, Luís Manuel Lopes Rodrigues Silva, Eric A F Simões, Andrew J H Simpson, Amit Singh, Surjit Singh, Robert Sinto, Sameh S M Soliman, Soroush Soraneh, Nicole Stoesser, Temenuga Zhekova Stoeva, Chandan Kumar Swain, Lukasz Szarpak, Sree Sudha T Y, Shima Tabatabai, Celine Tabche, Zanan Mohammed-Ameen Taha, Ker-Kan Tan, Nidanuch Tasak, Nathan Y Tat, Areerat Thaiprakong, Pugazhenthan Thangaraju, Caroline Chepngeno Tigoi, Krishna Tiwari, Marcos Roberto Tovani-Palone, Thang Huu Tran, Munkhtuya Tumurkhuu, Paul Turner, Aniefiok John Udoakang, Arit Udoh, Noor Ullah, Saeed Ullah, Asokan Govindaraj Vaithinathan, Mario Valenti, Theo Vos, Huong T L Vu, Yasir Waheed, Ann Sarah Walker, Judd L Walson, Tri Wangrangsimakul, Kosala Gayan Weerakoon, Heiman F L Wertheim, Phoebe C M Williams, Asrat Arja Wolde, Teresa M Wozniak, Felicia Wu, Zenghong Wu, Mukesh Kumar Kumar Yadav, Sajad Yaghoubi, Zwanden Sule Yahaya, Amir Yarahmadi, Saber Yezli, Yazachew Engida Yismaw, Dong Keon Yon, Chun-Wei Yuan, Hadiza Yusuf, Fathiah Zakham, Giulia Zamagni, Haijun Zhang, Zhi-Jiang Zhang, Magdalena Zielińska, Alimuddin Zumla, Sa'ed H H Zyoud, Samer H Zyoud, Simon I Hay, Andy Stergachis, Benn Sartorius, Ben S Cooper, Christiane Dolecek, and Christopher J L Murray.

*Joint first authors

Affiliations

Institute for Health Metrics and Evaluation (Prof M Naghavi PhD, Prof S E Vollset DrPH, K S Ikuta MD, L R Swetschinski MSc, A P Gray BSc, E E Wool MPH, T Mestrovic PhD, G Smith MS, C Han BA, R L Hsu MPA, J Chalek BS, D T Araki MPH, E Chung MD, C Raggi MS, A Gershberg Hayoon MSc, N Davis Weaver MPH, P A Lindstedt MPH, A E Smith MPA, N V Bhattacharjee PhD, T Runghien MSc, A J Ahlstrom MSc, A Y Aravkin PhD, X Dai PhD, J E Fuller MLIS, J He MSc, H H Kyu PhD, Prof S S Lim PhD, K L Maass PhD, T A McHugh PhD, Prof A H Mokdad PhD, V Mougin BA, Q P Nguyen BS, M Sahu MS, A E Schumacher PhD, N Shalev MD, Prof T Vos PhD, A A Wolde MPH, C Yuan PhD, Prof S I Hay FMedSci, Prof C J L Murray DPhil), Department of Health Metrics Sciences, School of Medicine (Prof M Naghavi PhD, Prof S E Vollset DrPH, A Y Aravkin PhD, X Dai PhD, H H Kyu PhD, Prof S S Lim PhD, Prof A H Mokdad PhD, Prof T Vos PhD, Prof S I Hay FMedSci, Prof A Stergachis PhD, B Sartorius PhD, Prof C J L Murray DPhil), Department of Pediatrics (E Chung MD), Department of Applied Mathematics (A J Ahlstrom MSc, A Y Aravkin PhD), School of Health Systems and Public Health (C Iwu MPH), Department of Biostatistics (A Olivas-Martinez MD), Department of Global Health (Prof J L Walson MD), Department of Pharmacy (Prof A Stergachis PhD), University of Washington, Seattle, WA, USA; GBD Collaborating Unit (Prof S E Vollset DrPH, K Giannakis PhD), Norwegian Institute of Public Health, Bergen, Norway (U Altay PhD); Division of Infectious Diseases (K S Ikuta MD), Veterans Affairs Greater Los Angeles, Los Angeles, CA, USA; Nuffield Department of Medicine (G Robles Aguilar DPhil, B McManigal PhD, N Ekapirat MSc, J A Mendes PhD, T Runghien MSc, O Srimokla MSc, Prof E A Ashley FRCP, E A P Kumaran MSc, C Lim PhD, A J H Simpson FRCPath, Prof N Stoesser DPhil, Prof A Walker PhD, T Wangrangsimakul MB, B Sartorius PhD, Prof B S Cooper PhD, Prof C Dolecek PhD), Big Data Institute (F Fell MSc, A J Browne MPH), The Global Research on Antimicrobial Resistance Project (O Srimokla MSc), Nuffield Department of Population Health (K Allel PhD, Prof D W Eyre DPhil), Centre for Tropical Medicine and Global Health (Prof J A Berkley PhD, S J Dunachie PhD, T Roberts PhD, Prof P Turner PhD), International Severe Acute Respiratory and Emerging Infection Consortium (ISARIC) (E E Garcia-Gallo PhD), Nuffield Department of Clinical Medicine (C Perrone MD), Oxford Vaccine Group (Prof A J Pollard FRS), University of Oxford, Oxford, UK; University Centre Varazdin (T Mestrovic PhD), University North, Varazdin, Croatia; University of Texas Health Science Center,

Houston, TX, USA (D T Araki MPH); Department of Mathematics and Sciences (A Abdelkader PhD), Department of Clinical Sciences (S O Alalalmeh BPharm, O E Hegazi BPharm), Center for Medical and Bio-allied Health Sciences Research (Prof M J Shahwan PhD, A Shamsi PhD, S H Zyoud PhD), Ajman University, Ajman, United Arab Emirates; Department of Tropical Medicine and Infectious Diseases (S Abd-Elsalam PhD), Tanta University, Tanta, Egypt; Department of Family and Community Health (R G Aboagye MPH), Department of Epidemiology and Biostatistics (L A Adzigbli BSc, R K Dowou MPhil), University of Health and Allied Sciences, Ho, Ghana; Research Center for Immunodeficiencies (H Abolhassani PhD, A Saghazadeh MD), Non-communicable Diseases Research Center (S Azadnajafabad MD, M Rashidi MD, S Saeedi Moghaddam MSc), Neurosicence Institute (M Babaei MD), Iranian Research Center for HIV/AIDS (IRCHA) (O Dadras PhD), Sina Trauma and Surgery Research Center (M Hassan Zadeh Tabatabaei MD, Prof V Rahimi-Movaghar MD), Department of Neurology (M Shafie MD), Research Center for Rational Use of Drugs (A Sharifan PharmD), Tehran University of Medical Sciences, Tehran, Iran; Department of Medical Biochemistry and Biophysics (H Abolhassani PhD), Karolinska Institute, Stockholm, Sweden; Department of Nursing (H Abualruz PhD), Al Zaytoonah University of Jordan, Amman, Jordan; Department of Clinical Pharmacy and Practice (U Abubakar PhD), Qatar University, Doha, Qatar; Academic Health System (H J Abukhadijah MPH), Hamad Medical Corporation, Doha, Qatar; Department of Therapeutics (Prof S Aburuz PhD), College of Medicine and Health Sciences (J Nauman PhD), United Arab Emirates University, Al Ain, United Arab Emirates; College of Pharmacy (Prof S Aburuz PhD), University of Jordan, Amman, Jordan; Department of Biochemistry and Molecular Medicine (A Abu-Zaid PhD), Alfaisal University, Riyadh, Saudi Arabia; College of Graduate Health Sciences (A Abu-Zaid PhD), University of Tennessee, Memphis, TN, USA; Chiang Rai Prachanukroh Hospital (S Achalapong PhD, S Khusuwan MD, P Ounchanum MD, W Saengchan BSc, S Seekaew MD), Ministry of Public Health, Chiang Rai, Thailand; School of Medicine (I Y Addo PhD), Faculty of Medicine (B F R Dickson MBBS), Sydney Medical School (S Islam PhD), School of Chemical & Biomolecular Engineering (E A Noman PhD), Sydney Institute for Infectious Diseases (P C M Williams DPhil), University of Sydney, Sydney, NSW, Australia (M L Harrison MGH, S R Okeke PhD); Centre for Social Research in Health (I Y Addo PhD, S R Okeke PhD), School of Population Health (Prof B A Saddik PhD), University of New South Wales, Sydney, NSW, Australia; Department of Obstetrics and Gynecology (V Adekanmbi PhD), University of Texas Medical Branch, Galveston, TX, USA; Department of Pharmacology and Therapeutics (T E Adeyeoluwa PhD), Department of Microbiology (I A Anuoluwa PhD, O O Bello PhD, T C Ekundayo PhD), Department of Biosciences and Biotechnology (O T Oyeyemi PhD, A J Udoakang PhD), University of Medical Sciences, Ondo, Ondo, Nigeria; Department of Veterinary Medicine (T E Adeyeoluwa PhD), Department of Epidemiology and Medical Statistics (A F Fagbamigbe PhD), Department of Veterinary Public Health and Preventive Medicine (O A Ogundijo MSc), University of Ibadan, Ibadan, Nigeria; Department of Public Health (Q Adnani PhD), Universitas Padjadjaran (Padjadjaran University), Bandung, Indonesia; Department of Life Sciences (M S Afzal PhD), University of Management and Technology, Lahore, Pakistan; Department of Community Medicine (Prof S Afzal PhD), King Edward Memorial Hospital, Lahore, Pakistan; Department of Public Health (Prof S Afzal PhD), Public Health Institute, Lahore, Pakistan; Department of Medical and Surgical Sciences and Advanced Technologies "GF Ingrassia" (Prof A Agodi PhD), University of Catania, Catania, Italy; Department of Medical Biochemistry (A Ahmad PhD), Shaqra University, Shaqra, Saudi Arabia; Department of Health and Biological Sciences (S Ahmad PhD), Abasyn University, Peshawar, Pakistan; Department of Natural Sciences (S Ahmad PhD), Lebanese American University, Beirut, Lebanon; School of Public Health (T Ahmad PhD), Zhejiang University, Hangzhou, China; Department of Epidemiology and Biostatistics (A

Ahmadi PhD), Community-Oriented Nursing Midwifery Research Center (M Heidari PhD), Shahrekord University of Medical Sciences, Shahrekord, Iran; Department of Epidemiology (A Ahmadi PhD), Social Determinants of Health Research Center (M Rashidi MD), Department of Medical Education (S Tabatabai PhD), Shahid Beheshti University of Medical Sciences, Tehran, Iran; Institute of Endemic Diseases (A Ahmed MSc), Unit of Basic Medical Sciences (E E Siddig MD), University of Khartoum, Khartoum, Sudan; Swiss Tropical and Public Health Institute (A Ahmed MSc), Department of Paediatric Infectious Diseases (J A Bielicki PhD), University of Basel, Basel, Switzerland; Department of Biosciences (H Ahmed PhD), COMSATS Institute of Information Technology, Islamabad, Pakistan; Research and Development Department (I Ahmed PhD), Alpha Genomics Private Limited, Islamabad, Pakistan (Z Basharat PhD); Microbiological Analysis Team (I Ahmed PhD), Korea Research Institute of Standards and Science (KRISS), Daejeon, South Korea; Department of Medical Microbiology (M Ahmed MSc), Department of Public Health (S D Darcho MPH), Haramaya University, Harar, Ethiopia; Department of Biological Sciences (S Ahmed PhD, L Ali PhD), National University of Medical Sciences (NUMS), Rawalpindi, Pakistan; Brody School of Medicine (S Ahmed PhD), East Carolina University, Greenville, NC, USA; Department of Cardiology (M Akkaif PhD), Fudan University, Shanghai, China; Department of Communicable Diseases (S Al Awaidy MSc), Ministry of Health, Muscat, Oman; Middle East, Eurasia, and Africa Influenza Stakeholders Network, Muscat, Oman (S Al Awaidy MSc); Faculty of Pharmacy (Y Al Thaher PhD), Faculty of Nursing (M M W Atout PhD), Philadelphia University, Amman, Jordan; School of Pharmacy (Y Al Thaher PhD), Cardiff University, Cardiff, UK; Faculty of Medicine (Prof M T AlBataineh PhD), Yarmouk University, Irbid, Jordan; Division of Gastroenterology and Hepatology (W A Aldhaleei MD, A S Bhagavathula PhD), Mayo Clinic, Jacksonville, FL, USA; Global Centre for Environmental Remediation (A A S Al-Gheethi PhD), University of Newcastle, Newcastle, NSW, Australia; Cooperative Research Centre for Contamination Assessment and Remediation of the Environment, Newcastle, NSW, Australia (A A S Al-Gheethi PhD); Africa Center of Excellence for Mycotoxin and Food Safety, Minna, Nigeria (N B Alhaji PhD); Department of Zoology (A Ali PhD), Abdul Wali Khan University Mardan, Mardan, Pakistan; Center for Biotechnology and Microbiology (S S Ali PhD), University of Swat, Swat, Pakistan; Department of Geography (W Ali PhD), Sultan Qaboos University, Muscat, Oman; Institute for Global Health (K Allel PhD), Medical Research Council Clinical Trials Unit (MRC CTU) (Prof A Walker PhD), Center for Clinical Microbiology (Prof A Zumla PhD), University College London, London, UK; Department of Dentistry (S Al-Marwani MSc), Sana'a University, Sana'a, Yemen; Independent Consultant, Irbid, Jordan (S Al-Marwani MSc); Department of Allied Medical Sciences (A Alrawashdeh PhD), Department of Clinical Pharmacy (Prof K H Alzoubi PhD), Department of Public Health (Prof Y S Khader PhD), Jordan University of Science and Technology, Irbid, Jordan; Institute of Molecular Biology and Biotechnology (A Altaf PhD, S Shahid PhD), Research Centre for Health Sciences (RCHS) (S Shahid PhD), The University of Lahore, Lahore, Pakistan; Research, Policy, and Training Directorate (A Al-Tammemi MPH), Jordan Center for Disease Control, Amman, Jordan; Applied Science Research Center (A Al-Tammemi MPH), Applied Science Private University, Amman, Jordan; Department of Specialty Internal Medicine (Prof J A Al-Tawfiq MD), Johns Hopkins Aramco Healthcare, Dhahran, Saudi Arabia; Department of Medicine (Prof J A Al-Tawfiq MD), Indiana University School of Medicine, Indianapolis, IN, USA; Department of Pharmacy Practice and Pharmacotherapeutics (Prof K H Alzoubi PhD), Clinical Sciences Department (H J Barqawi MPhil, N Saheb Sharif-Askari PhD), Department of Basic Biomedical Sciences (Prof Y Bustanji PhD), Department of Basic Medical Sciences (W El-Huneidi PhD), College of Medicine (Prof B A Saddik PhD, Prof M A Saleh PhD), Department of Medicinal Chemistry (S S M Soliman PhD), University of Sharjah, Sharjah, United Arab Emirates; Department of Biomedical Engineering (W A Al-Zyoud PhD), German Jordanian University,

Amman, Jordan; Infectious Diseases Surveillance (J A Kapisi MA), Infectious Diseases Research Collaboration (IDRC), Kampala, Uganda (B Amos PhD, P Olwoch MPH); Department of Global Health (J H Amuasi PhD), Kwame Nkrumah University of Science and Technology, Kumasi, Ghana; Global Health and Infectious Diseases (J H Amuasi PhD), Kumasi Center for Collaborative Research in Tropical Medicine, Kumasi, Ghana; Faculty of Pharmacy (Prof R Ancuceanu PhD), Department of Legal Medicine and Bioethics (Prof S Hostiuc PhD), Department of General Surgery (I Negoi PhD), Department of Anatomy and Embryology (R I Negoi PhD), Carol Davila University of Medicine and Pharmacy, Bucharest, Romania; Division of Infectious Disease and Geographic Medicine (J R Andrews MD), Department of Medicine (M Babaei MD), Department of Radiology (S Ramasamy MD), Stanford University, Stanford, CA, USA; Department of Pharmacology (A Anil MD, M Shamim MBBS, S Singh MD, K Tiwari MBBS), Department of Community Medicine and Family Medicine (Prof P Raghav MD), Department of Pharmacology and Research (A Saravanan MD), All India Institute of Medical Sciences, Jodhpur, India; All India Institute of Medical Sciences, Bhubaneswar, India (A Anil MD); Regenerative Medicine, Organ Procurement and Transplantation Multi-disciplinary Center (S Anvari MD), Department of Social Medicine and Epidemiology (A Feizkhah MD), Guilan University of Medical Sciences, Rasht, Iran; Rural Health Research Institute (A E Anyasodor PhD), Rural Health research Institute (Prof A G Ross MD), Charles Sturt University, Orange, NSW, Australia; School of Medicine and Public Health (G C Apostol MD), Ateneo De Manila University, Pasig City, Philippines; Inter-Agency Committee on Environmental Health (G C Apostol MD), Department of Health Philippines, Manila, Philippines; Health Management and Economics Research Center (J Arabloo PhD), School of Medicine (M Dodangeh MD), Preventive Medicine and Public Health Research Center (B Eshrati PhD), Department of Ophthalmology (H Hasani MD), Research Center of Pediatric Infectious Diseases (F Khamesipour PhD), Department of Pediatrics (H Masoumi-Asl MD), Iran University of Medical Sciences, Tehran, Iran; College of Pharmacy (M Arafat PhD), Al Ain University, Abu Dhabi, United Arab Emirates; College of Art and Science (D Areda PhD), Ottawa University, Surprise, AZ, USA; School of Life Sciences (D Areda PhD), Arizona State University, Tempe, AZ, USA; Department of Veterinary Pharmacology and Toxicology (A Aremu PhD), Department of Veterinary Physiology and Biochemistry (A Basiru PhD), Department of Veterinary Public Health and Preventive Medicine (I A Odetokun PhD), University of Ilorin, Ilorin, Nigeria; Institute for Biomedical Problems (A A Artamonov PhD), Russian Academy of Sciences, Moscow, Russia; Microbiology Laboratory (V Chansamouth MSc, K Phommasone PhD), Department of Microbiology (T Roberts PhD), Lao-Oxford-Mahosot Hospital-Wellcome Trust Research Unit (LOMWRU), Vientiane, Laos (Prof E A Ashley FRCP); Department of Medical Laboratory Sciences (M O Asika BMLS), University of Nigeria Nsukka, Enugu, Nigeria; Department of Telemedicine (M O Asika BMLS), Society for Disease Prevention, Inc, Hummelstown, PA, USA; Department of Immunology (S Athari PhD), Zanjan University of Medical Sciences, Zanjan, Iran; Department of Medical Laboratory Sciences (T Awoke MSc, A Melese MSc), Department of Medical Microbiology (A D Habteyohannes PhD), Department of Pharmacology (Y Yismaw MSc), Bahir Dar University, Bahir Dar, Ethiopia; Department of Surgery (S Azadnajafabad MD), Washington University in St Louis, St Louis, MO, USA; Department of Infectious Disease Epidemiology (J M Azam PhD), Faculty of Infectious and Tropical Diseases (Prof R S Hasan PhD), Department of Biological Sciences (D C Nwakanma PhD), London School of Hygiene & Tropical Medicine, London, UK (H Hopkins MD); Department of Applied Mathematics (J M Azam PhD), Stellenbosch University, Stellenbosch, South Africa; Institute of Biotechnology and Genetic Engineering (S Aziz MS), The University of Agriculture, Peshawar, Pakistan; Montefiore-Einstein Cerebrovascular Research Lab (A Azzam MBBCh), Albert Einstein College of Medicine, Bronx, NY, USA; Faculty of Medicine (A Azzam MBBCh), Department of Cardiology (O M

Makram MD), October 6 University, 6th of October City, Egypt; Laboratory (F Babin PharmD), Fondation Merieux, Lyon, France; Gomal Center of Biochemistry and Biotechnology (M Badar PhD), Gomal University, Dera Ismail Khan, Pakistan; International Medical School (A A Baig PhD), Management and Science University, Alam, Malaysia; Department of Pharmacology, Clinical Pharmacology and Toxicology (Prof M Bajcetic PhD), Faculty of Medicine (I M Ilic PhD), University of Belgrade, Belgrade, Serbia; Department for Clinical Pharmacology (Prof M Bajcetic PhD), University Children Hospital, Belgrade, Serbia; Department of Medicine (Prof S Baker PhD), Cambridge Institute of Therapeutic Immunology and Infectious Disease (CITIID) (Prof F Marks PhD), University of Cambridge, Cambridge, UK (H Jeon PhD); Miller School of Medicine (M Bardhan MD), Department of Public Health Sciences (V D Rosenthal MD), University of Miami, Miami, FL, USA; World Health Organization, Geneva, Switzerland (M Bastard MSc); Department of Academics (S Basu MD), Indian Institute of Public Health, Gurgaon, India; Department of Surgery (N S Bayleyegn MD), Jimma University, Jimma, Ethiopia; Department of Medical Laboratory Science (M A Belete MSc), Department of Environmental Health (M Gebrehiwot DSc), Wollo University, Dessie, Ethiopia; Department of Biomedical Sciences (Prof A Beloukas PhD), National AIDS Reference Center of Southern Greece (Prof A Beloukas PhD), School of Public Health (Prof G D Mandilara PhD), University of West Attica, Athens, Greece; Centre for Clinical Research (Prof J A Berkley PhD, L A Musila PhD), Kenya Medical Research Institute, Nairobi, Kenya (C C Tigoi DPhil); Department of Public Health (A S Bhagavathula PhD), North Dakota State University, Fargo, ND, USA; Global Health Neurology Lab (S Bhaskar MD), NSW Brain Clot Bank, Sydney, NSW, Australia; Division of Cerebrovascular Medicine and Neurology (S Bhaskar MD), National Cerebral and Cardiovascular Center, Suita, Japan; Department of Health Administration (S S Bhuyan PhD), Rutgers University, New Brunswick, NJ, USA; Institute for Infection and Immunity (J A Bielicki PhD), Centre for Neonatal and Paediatric Infection (C E Moore PhD), St George's University of London, London, UK; Department of Epidemiology and Evidence-Based Medicine (Prof N I Briko DSc, V A Korshunov PhD), IM Sechenov First Moscow State Medical University, Moscow, Russia; HCAI, Fungal, AMR, AMU, & Sepsis Division (C S Brown MD), United Kingdom Health Security Agency, London, UK; Department of Infection (C S Brown MD), Department of Surgery and Cancer (Prof E Mossialos PhD), Department of Primary Care and Public Health (Prof S Rawaf MD, C Tabche MSc), Imperial College London, London, UK; Department of Woman and Child Health and Public Health (D Buonsenso MD), Fondazione Policlinico Universitario A Gemelli IRCCS (Agostino Gemelli University Polyclinic IRCCS), Rome, Italy; Global Health Research Institute (D Buonsenso MD), Università Cattolica del Sacro Cuore (Catholic University of Sacred Heart), Rome, Italy; School of Pharmacy (Prof Y Bustanji PhD), The University of Jordan, Amman, Jordan; Department of Pediatrics (C G Carvalheiro PhD), University of São Paulo, Ribeirao Preto, Brazil; Department of Pediatrics (C G Carvalheiro PhD), Clinical Hospital of Ribeirão Preto, Ribeirão Preto, Brazil; Colombian National Health Observatory (C A Castañeda-Orjuela PhD), Instituto Nacional de Salud (National Institute of Health), Bogota, Colombia; Epidemiology and Public Health Evaluation Group (C A Castañeda-Orjuela PhD), Department of Public Health (Prof F P De la Hoz PhD), National University of Colombia, Bogota, Colombia; College of Public Health, Medical, and Veterinary Sciences (M Cenderadewi MPHTM), James Cook University, Townsville, QLD, Australia; Department of Public Health (M Cenderadewi MPHTM), University of Mataram, Mataram, Indonesia; Non-communicable Diseases Division (J Chadwick MD), National Institute of Epidemiology, Chennai, India; State Disease Investigation Laboratory (S Chakraborty MVSc), Animal Resources Development Department, Agartala, India; Department of Clinical Nutrition (R M Chandika PhD), Department of Epidemiology (S Dohare MD), Substance Abuse and Toxicology Research Center (S Mohan PhD), Jazan University, Jazan, Saudi Arabia; Department of Pediatrics (S Chandy PhD), The Childs

Trust Medical Research Foundation (CTMRF), Chennai, India; Microbiology Laboratory (V Chansamouth MSc), Ministry of Health, Vientiane, Laos; Temerty Faculty of Medicine (V Chattu MD), University of Toronto, Toronto, ON, Canada; Department of Community Medicine (V Chattu MD), Datta Meghe Institute of Medical Sciences, Sawangi, India; Department of Biology (A A Chaudhary PhD), Al-Imam Mohammad Ibn Saud Islamic University, Riyadh, Saudi Arabia; Division of Infectious Diseases (P R Ching MD), Virginia Commonwealth University, Richmond, VA, USA; Centre for Research Impact & Outcome (H Chopra PhD), Chitkara University, Rajpura, India; Department of Internal Medicine (F R Chowdhury PhD), Bangabandhu Sheikh Mujib Medical University, Dhaka, Bangladesh; Center for Biomedicine and Community Health (D Chu PhD), International School, Vietnam National University Hanoi (VNUIS), Hanoi, Vietnam; School of Nursing and Midwifery (M Chutiyami PhD), University of Technology Sydney, Sydney, NSW, Australia; Department of Diagnostic and Therapeutic Technologies (Prof N Cruz-Martins PhD), Cooperativa de Ensino Superior Politécnico e Universitário (Polytechnic and University Higher Education Cooperative), Vila Nova de Famalicão, Portugal; Institute for Research and Innovation in Health (i3S) (Prof N Cruz-Martins PhD), Associate Laboratory Institute for Health and Bioeconomy (i4HB) (Prof C Fortuna Rodrigues PhD), Faculty of Engineering (Prof C Fortuna Rodrigues PhD), University of Porto, Porto, Portugal; School of Nursing (A da Silva PhD), Federal University of Minas Gerais, Belo Horizonte, Brazil; Department of Global Public Health and Primary Care (O Dadras PhD), Center for International Health (CIH) (S Hassan MPhil), Bergen Center for Ethics and Priority Setting (BCEPS) (S Hassan MPhil), University of Bergen, Bergen, Norway; Department of Biochemistry (S Das MD), Ministry of Health and Welfare, New Delhi, India; Department of Implementation Research (D M Dekker PhD), Department of Infectious Disease Epidemiology (R Krumkamp DrPH, Prof J May MD), Bernhard Nocht Institute for Tropical Medicine, Hamburg, Germany; Division of Pathology (K Dhama PhD), ICAR-Indian Veterinary Research Institute, Bareilly, India; Faculty of Science (Prof D Diaz PhD), National Autonomous University of Mexico, Mexico City, Mexico; Sydney Institute of Infectious Diseases, Sydney, NSW, Australia (B F R Dickson MBBS); Laboratoire National de Biologie Clinique et de Santé (National Laboratory of Clinical Biology and Health) (S Djorie Dr, C D Rafai MD), Ministère de la Santé Publique et de la Population (Ministry of Public Health and Population), Bangui, Central African Republic; Department of Social Medicine and Health Care Organisation (Prof K G Dokova PhD), Department of Microbiology and Virology (Prof T Z Stoeva DSc), Medical University of Varna, Varna, Bulgaria; Independent Consultant, South Plainfield, NJ, USA (O P Doshi MSc); Kasturba Medical College Mangalore (R Holla MD), Department of Pharmaceutical Regulatory Affairs and Management (V S Ligade PhD), Manipal Academy of Higher Education, Manipal, India (H L Dsouza MD); Department of Forensic Medicine and Toxicology (H L Dsouza MD), Kasturba Medical College Mangalore, Mangalore, India; Mahidol Oxford Tropical Medicine Research Unit (S J Dunachie PhD, Prof C Dolecek PhD), Mahidol University, Bangkok, Thailand; Department of Conservative Dentistry with Endodontics (A M Dziedzic DSc), Medical University of Silesia, Katowice, Poland; Department of Infectious Disease Epidemiology (S Haller MD), Robert Koch Institute, Berlin, Germany (T Eckmanns Dr); Higher School of Technology (Prof A Ed-Dra PhD), Sultan Moulay Slimane University, Beni Mellal, Morocco; Department of Biochemistry (A Eftekharimehrabad PhD), Ege University, Izmir, Turkiye; Azerbaijan State University of Economics (UNEC), Baku, Azerbaijan (A Eftekharimehrabad PhD); Biomedical Informatics and Medical Statistics Department (I El Sayed PhD), Tropical Health Department (R M Ghazy PhD), Alexandria University, Alexandria, Egypt; Faculty of Medicine (M Elhadi MD), University of Tripoli, Tripoli, Libya; Houston Methodist Hospital, Houston, TX, USA (M Elhadi MD); Infection Prevention and Control Unit (C Elias PharmD), REA-REZO (A Lepape MD), Hospices Civils de Lyon, Lyon, France; Public Health, Epidemiology and Evolutionary Ecology of Infectious

Diseases (PHE3ID) (C Elias PharmD, A Lepape MD), Centre International de Recherche en Infectiologie (CIRI), Lyon, France; Research and Development Department (S J Ellis MSc), Global Antibiotic Research and Development Partnership (GARDP), Geneva, Switzerland (S O'Brien PhD); Deanery of Biomedical Sciences (R Elsheikh MD), Global Health Governance Programme (J Patel BSc), University of Edinburgh, Edinburgh, UK; Department of Infectious Diseases and Public Health (I Elsohaby PhD), Department of Public Health and Infectious Diseases (G Fekadu PhD), City University of Hong Kong, Hong Kong, China; Department of Animal Medicine (I Elsohaby PhD), Zagazig University, Zagazig, Egypt; Department of Pediatrics (C Eltaha MD), Texas A&M University, Dallas, TX, USA; Department of Bacteriology and Virology (M Eslami PhD), Cancer Research Center (M Eslami PhD), Semnan University of Medical Sciences, Semnan, Iran; Department of Anesthesia (A O Fadaka PhD), Cincinnati Children's Hospital Medical Center, Cincinnati, OH, USA; Department of Biotechnology (A O Fadaka PhD), School of Pharmacy (O C Okonji MSc), University of the Western Cape, Cape Town, South Africa; Research Centre for Healthcare and Community (A F Fagbamigbe PhD), Coventry University, Coventry, UK; Department of Oral Biology (A Fahim PhD), Riphah International University, Islamabad, Pakistan; Department of Food Hygiene and Quality Control (A Fakhri-Demeshghieh DVM), University of Tehran, Tehran, Iran; Department of Veterinary Tropical Diseases (Prof F O Fasina PhD), University of Pretoria, Pretoria, South Africa; Animal Production and Health Division (EMPRES) (Prof F O Fasina PhD), Food and Agriculture Organization of the United Nations, Rome, Italy; Institute of International Health (M M Fasina MSc), Institute of Public Health (F Fischer PhD), Institute of Hygiene and Environmental Medicine (Prof C Geffers MD), Charité Universitätsmedizin Berlin (Charité Medical University Berlin), Berlin, Germany; Department of Environmental Health Engineering (A Fatehizadeh PhD), Isfahan University of Medical Sciences, Isfahan, Iran; Department of Clinical Sciences (Prof N A Feasey PhD), Liverpool School of Tropical Medicine, Liverpool, UK; Bacteria and Drug Resistant Infections Group (Prof N A Feasey PhD), Malawi Liverpool Wellcome Trust Clinical Research Programme, Blantyre, Malawi; Department of Pharmacy (G Fekadu PhD), Wollega University, Nekemte, Ethiopia; Department of Pharmacology (I Fitriana PhD), Gadjah Mada University, Yogyakarta, Indonesia; Clinical Services Department (K M Forrest FRCP), Medical Research Council Unit, The Gambia, Fajara, The Gambia; Department of Community Medicine (Prof M A Gadanya MD), Bayero University Kano, Kano, Nigeria; Department of Community Medicine (Prof M A Gadanya MD), Aminu Kano Teaching Hospital, Kano, Nigeria; Department of Oral Biology and Experimental Dental Research (M Gajdács PhD), University of Szeged, Szeged, Hungary; Department of Community Medicine and Family Medicine (A P Gandhi MD), All India Institute of Medical Sciences, Nagpur, India; Unisabana Center for Translational Science (E E Garcia-Gallo PhD, L Reyes PhD), Universidad de La Sabana (Savannah University), Chia, Colombia; Applied Epidemiology Program (D O Garrett MD), Sabin Vaccine Institute, Washington, DC, USA; Department of Pharmacology (Prof R K Gautam PhD), Indore Institute of Pharmacy, Indore, India; Department of Midwifery (M W Gebregergis MSc), Department of Medical Laboratory Sciences (H N Meles MSc), Adigrat University, Adigrat, Ethiopia; Department of Reproductive and Family Health (T G Gebremeskel PhD), Axum College of Health Science, Axum, Ethiopia; College of Medicine and Public Health (T G Gebremeskel PhD), Flinders University, Adelaide, SA, Australia; Hellenic National Public Health Organization, Athens, Greece (L Georgalis PhD); Family and Community Medicine Department (R M Ghazy PhD), King Khalid University Hospital, Abha, Saudi Arabia; Department of Health Systems and Policy Research (Prof M Golechha PhD), Indian Institute of Public Health, Gandhinagar, India; Department of Life Sciences, Health and Healthcare Professions (Prof D Golinelli MD), Link Campus University, Rome, Italy; Health Services Research, Evaluation and Policy Unit (Prof D Golinelli MD), AUSL della Romagna, Ravenna, Italy; Institute

of Infection, Veterinary and Ecological Sciences (Prof M Gordon MA), University of Liverpool, Liverpool, UK; Department of Thoracic Surgery (S Gulati MD), Lerner Research Institute (X Liu PhD), Cleveland Clinic, Cleveland, OH, USA; Department of Epidemiology and Biostatistics (R Gupta MPH), University of South Carolina, Columbia, SC, USA; Centre for Noncommunicable Diseases and Nutrition (R Gupta MPH), BRAC University, Dhaka, Bangladesh; Department of Toxicology (S Gupta MSc), Shriram Institute for Industrial Research, Delhi, India; School of Biotechnology (V Gupta PhD), Dublin City University, Dublin, Ireland; Department of Public Health (S Haller MD), Charité Institute of Public Health, Berlin, Germany; Medical Research Unit (H Harapan PhD), Universitas Syiah Kuala (Syiah Kuala University), Banda Aceh, Indonesia; Sydney Infectious Diseases Institute, Sydney, NSW, Australia (M L Harrison MGH); Department of Zoology and Entomology (A I Hasaballah PhD), Al-Azhar University, Cairo, Egypt; Department of Pharmaceutical Technology (I Hasan MPharm), University of Dhaka, Dhaka, Bangladesh; Department of Pathology and Laboratory Medicine (Prof R S Hasan PhD, S Shakoor MD), Department of Paediatrics and Child Health (Prof F N Qamar FRCP), Aga Khan University, Karachi, Pakistan; Interventions and Implementation Research Department (A H Haselbeck DSc), Epidemiology and Public Health Research Department (M Holm PhD), Epidemiology, Public Health, Impact Unit (EPIC) (Prof F Marks PhD), International Vaccine Institute, Seoul, South Korea (H Jeon PhD); Department of Pharmacy (Prof M S Hasnain PhD), Palamau Institute of Pharmacy, Daltonganj, India; Public Health Department (I I Hassan PhD), Dalhatu Araf Specialist Hospital, Lafia, Nigeria; Department of Public Health (I I Hassan PhD), Federal University of Lafia, Lafia, Nigeria; Institute of Pharmaceutical Sciences (K Hayat MS), University of Veterinary and Animal Sciences, Lahore, Pakistan; Department of Pharmacy Administration and Clinical Pharmacy (K Hayat MS), Xian Jiaotong University, Xian, China; Department of Microbiology (K Hezam PhD), Taiz University, Taiz, Yemen; School of Medicine (K Hezam PhD), Nankai University, Tianjin, China; Department of Decision and Information Sciences (M Hossain DrPH), University of Houston, Houston, TX, USA; Public Health Research Group (M Hossain DrPH), Nature Study Society of Bangladesh, Khulna, Bangladesh; Institute of Research and Development (Prof M Hosseinzadeh PhD), Duy Tan University, Da Nang, Vietnam; Department of Computer Science (Prof M Hosseinzadeh PhD), University of Human Development, Sulaymaniyah, Iraq; Department of Clinical Legal Medicine (Prof S Hostiuc PhD), National Institute of Legal Medicine Mina Minovici, Bucharest, Romania; Department of Biomolecular Sciences (Prof N R Hussein PhD), University of Zakho, Zakho, Iraq; College of Health Sciences (L D Huy MBA), VinUniversity, Hanoi, Vietnam; School of Health Care Administration (L D Huy MBA), Taipei Medical University, Taipei, Taiwan; Critical Care Department (L Reyes PhD), Clinica Universidad De La Sabana (Savannah University Clinic), Chia, Colombia (E D Ibáñez-Prada MSc); The National Centre for Remote and Rural Health and Care (A Ikiroma PhD), NHS National Services Scotland, Edinburgh, Scotland; Institute for Physical Activity and Nutrition (S Islam PhD), Deakin University, Burwood, VIC, Australia; Clinical Laboratory Department (F Ismail PhD), Tobruk University, Tobruk, Libya; Department of Blood Transmitted Diseases (F Ismail PhD), National Centre for Disease Control (NCDC), Tobruk, Libya; Department of Clinical Pharmacy & Pharmacy Practice (Prof N Ismail PhD), Asian Institute of Medicine, Science and Technology, Bedong, Malaysia; Malaysian Academy of Pharmacy, Puchong, Malaysia (Prof N Ismail PhD); Department of Global Health (C J Iwu-Jaja PhD), South African Medical Research Council, Cape Town, South Africa; Department of Global Health (C J Iwu-Jaja PhD), Stellenbosch University, Cape Town, South Africa; Department of Immunology (Prof A Jafarzadeh PhD), HIV/STI Surveillance Research Center (S Nejadghaderi MD), Kerman University of Medical Sciences, Kerman, Iran; Department of Immunology (Prof A Jafarzadeh PhD), Rafsanjan University of Medical Sciences, Rafsanjan, Iran; Clinical Services Department (F Jaiteh BSc), Medical Research Council Unit, The

Gambia, Banjul, Gambia; Department of Environmental Engineering (Prof R Jalilzadeh Yengejeh PhD), Islamic Azad University, Ahvaz, Iran; Department of Neurosciences (Prof R G Jamora PhD), University of the Philippines Manila, Manila, Philippines; Institute for Neurosciences (Prof R G Jamora PhD), St. Luke's Medical Center, Bonifacio Global City, Philippines; Invasive Fungi Research Center (J Javidnia PhD), Mazandaran University of Medical Sciences, Sari, Iran; Department of Pharmacology (T Jawaid PhD), Imam Mohammad Ibn Saud Islamic University, Riyadh, Saudi Arabia; Department of Infectious Diseases (A W J Jenney PhD), The Alfred Hospital, Melbourne, VIC, Australia; Faculty of Veterinary Medicine (M Jokar DVM), University of Calgary, Calgary, AB, Canada; Young Researchers and Elite Club (M Jokar DVM), Islamic Azad University, Karaj, Iran; Department of Microbiology (N Jomehzadeh PhD, M Saki PhD), Ahvaz Jundishapur University of Medical Sciences, Ahvaz, Iran; Department of Microbiology (N Jomehzadeh PhD), Abadan School of Medical Sciences, Abadan, Iran; Health Services Management Training Centre (T Joo PhD), Semmelweis University, Budapest, Hungary; Hungarian Health Management Association, Budapest, Hungary (T Joo PhD); Department of Community Medicine (N Joseph MD), Manipal Academy of Higher Education, Mangalore, India; Department of Pharmacy (Z Kamal PhD, M T Khan PharmD, N Ullah PharmD), Shaheed Benazir Bhutto University, Dir Upper, Pakistan; School of Pharmacy (Z Kamal PhD), Shanghai Jiao Tong University, Shanghai, China; Faculty of Dentistry (K K Kanmodi MPH), University of Puthisastra, Phnom Penh, Cambodia; Office of the Executive Director (K K Kanmodi MPH), Cephas Health Research Initiative Inc, Ibadan, Nigeria; The Hansjörg Wyss Department of Plastic and Reconstructive Surgery (R S Kantar MD), NYU Langone Health, New York, NY, USA; Cleft Lip and Palate Surgery Division (R S Kantar MD), Global Smile Foundation, Norwood, MA, USA; School of Health Professions and Human Services (I M Karaye MD), Hofstra University, Hempstead, NY, USA; Department of Anesthesiology (I M Karaye MD), Montefiore Medical Center, Bronx, NY, USA; Amity Institute of Forensic Sciences (H Khajuria PhD, B P Nayak PhD), Amity University, Noida, India; College of Health Sciences (N Khalid PhD), Abu Dhabi University, Adu Dhabi, United Arab Emirates; Halal Research Center of the Islamic Republic of Iran (F Khamesipour PhD), Iran Food and Drug Administration, Tehran, Iran; Natural and Medical Sciences Research Center (A Khan PhD), University of Nizwa, Nizwa, Oman; Department of Rehabilitation Sciences (M J Khan MPH), Hong Kong Polytechnic University, Hong Kong, China; Department of Health (V Khanal PhD), Nepal Development Society, Chitwan, Nepal; Department of Preventable Non-communicable Disease (V Khanal PhD), Menzies School of Health Research, Alice Springs, NT, Australia; Department of Biochemistry (F Khidri PhD), Liaquat University of Medical and Health Sciences, Jamshoro, Pakistan; Department of Public Health (J Khubchandani PhD), New Mexico State University, Las Cruces, NM, USA; Broad Institute of MIT and Harvard, Cambridge, MA, USA (M Kim MD); Department of Radiology (X Liu PhD), Massachusetts General Hospital, Boston, MA, USA (M Kim MD); School of Health Sciences (Prof A Kisa PhD), Kristiania University College, Oslo, Norway; Department of International Health and Sustainable Development (Prof A Kisa PhD), Tulane University, New Orleans, LA, USA; Instituto de Medicina Tropical Alexander von Humboldt (Alexander von Humboldt Institute of Tropical Medicine) (F Krapp MD, J L Paredes MD), Department of Pediatrics (T J Ochoa PhD), Universidad Peruana Cayetano Heredia (Cayetano Heredia University), Lima, Peru; Doctoral School of Biomedical Sciences (F Krapp MD), Katholieke Universiteit Leuven, Leuven, Belgium; Department of Biochemistry (Prof M Kuddus PhD), University of Hail, Hail, Saudi Arabia; Atchabarov Scientific-Research Institute of Fundamental and Applied Medicine (M Kulimbet MSc), Kazakh National Medical University, Almaty, Kazakhstan; Center of Medicine and Public Health (M Kulimbet MSc), Asfendiyarov Kazakh National Medical University, Almaty, Kazakhstan; Department of Community Medicine (D Kumar MD), Rajendra Institute of Medical Sciences, Ranchi, India; Department of

Nephrology (A Kuttikkattu MD), Pushpagiri Institute of Medical Sciences and Research Centre, Thiruvalla, India; Unidad de Genética y Salud Pública (Prof I Landires MD), Instituto de Ciencias Médicas, Las Tablas, Panama; Ministry of Health (Prof I Landires MD), Hospital Joaquín Pablo Franco Sayas, Las Tablas, Panama; Department of Clinical Pharmacy and Pharmacy Management (B K Lawal PhD), Department of Pharmaceutics and Industrial Pharmacy (Z S Yahaya PhD), Kaduna State University, Kaduna, Nigeria; Department of Internal Medicine (T H Tran MD), University of Medicine and Pharmacy at Ho Chi Minh City, Ho Chi Minh City, Vietnam (T T T Le MD); Reference Centres and Reference Laboratories (I M Lederer DSc), Austrian Agency for Health and Food Safety (AGES), Graz, Austria; Department of Medical Science (M Lee PhD), Ajou University School of Medicine, Suwon, South Korea; Department of Precision Medicine (Prof S Lee MD), Sungkyunkwan University, Suwon, South Korea; Department of Public Health (T Lerango MPH), Dilla University, Dilla, Ethiopia; Department of Microbiology (C Lim PhD, C Perrone MD), Mahidol Oxford Tropical Medicine Research Unit, Bangkok, Thailand; Department of Pharmaceutics (L W Limenh MSc), Department of Internal Medicine (E Melese MD), University of Gondar, Gondar, Ethiopia; Department of Public Health (Prof C Liu PhD), School of Nursing and Midwifery (Prof M Rahman PhD), La Trobe University, Melbourne, VIC, Australia; Department of Radiology and Biomedical Imaging (X Liu PhD), Department of Genetics (S Pawar PhD), Yale University, New Haven, CT, USA; Department of Quantitative Health Science (X Liu PhD), Case Western Reserve University, Cleveland, OH, USA; Department of Infectious Diseases (M J Loftus MBBS, Prof A Y Peleg PhD), Monash University, Melbourne, VIC, Australia; Department of Infectious Diseases (M J Loftus MBBS), Alfred Health, Melbourne, VIC, Australia; Department of Chemistry (H I M Amin PhD), Salahaddin University-Erbil, Erbil, Iraq; Department of Medical Biochemical Analysis (H I M Amin PhD), Cihan University-Erbil, Erbil, Iraq; School of Pharmacy (S B Maharaj DBA), University of the West Indies, St Augustine, Trinidad and Tobago; Planetary Health Alliance, Boston, MA, USA (S B Maharaj DBA); Department of Clinical and Hospital Pharmacy (M A Mahmoud PhD), Ashok and Rita Patel Institute of Physiotherapy, Al-Madinah Al-Munawarrah, Saudi Arabia; Microbiology Department (P Maikanti-Charalampous MD), Nicosia General Hospital, Nicosia, Cyprus; Department of Medicine (O M Makram MD), Medical College of Georgia at Augusta University, Augusta, GA, USA; Rama Medical College Hospital and Research Centre, Uttar Pradesh, India (K Malhotra MBBS); Institute of Applied Health Research (K Malhotra MBBS), University of Birmingham, Birmingham, UK; Rabigh Faculty of Medicine (Prof A Malik PhD), Department of Dental Public Health (Z S Natto DrPH), King Abdulaziz University, Jeddah, Saudi Arabia; Department of Infectious Diseases (B A Martinez-Guerra MSc, E Ortiz-Brizuela MD, Prof A Ponce-De-Leon MD), Department of Medicine (A Olivas-Martinez MD), Instituto Nacional de Nutrición Salvador Zubirán (Salvador Zubiran National Institute of Medical Sciences and Nutrition), Mexico City, Mexico; Department of Nutrition and Dietetics (M Martorell PhD), Centre for Healthy Living (M Martorell PhD), University of Concepción, Concepción, Chile; Division of Immunology, Immunity to Infection and Respiratory Medicine (A G Mathioudakis PhD), University of Manchester, Manchester, UK; North West Lung Centre (A G Mathioudakis PhD), Manchester University NHS Foundation Trust, Manchester, UK; Department of Tropical Medicine (Prof J May MD), Medical Center Hamburg-Eppendorf (UKE), Hamburg, Germany; Department of Infection, Immunity, and Cardiovascular Disease (J Meiring PhD), University of Sheffield, Sheffield, UK; Department of International Health (H Zhang MS), Johns Hopkins University, Baltimore, MD, USA (E Melese MD); Multidisciplinary Department of Medical-Surgical and Dental Specialties (G Minervini PhD), University of Campania "Luigi Vanvitelli", Naples, Italy; Saveetha Dental College and Hospitals (G Minervini PhD, M Tovani-Palone PhD), Department of Pharmacology (S Muthupandian PhD), Centre of Molecular Medicine and Diagnostics (COMManD) (Prof

S Patil PhD), Center for Global Health Research (Prof A Sahebkar PhD), Saveetha University, Chennai, India; Molecular Biology Unit (N S Mohamed MSc), Bio-Statistical and Molecular Biology Department (N S Mohamed MSc), Sirius Training and Research Centre, Khartoum, Sudan; Health Systems and Policy Research Unit (Prof S Mohammed PhD), Department of Paediatrics (S Musa MSc), Department of Community Medicine (A A Olorukooba MD), Ahmadu Bello University, Zaria, Nigeria; Heidelberg Institute of Global Health (HIGH) (Prof S Mohammed PhD), Heidelberg University, Heidelberg, Germany; School of Health Sciences (S Mohan PhD), University of Petroleum and Energy Studies, Dehradun, India; Clinical Epidemiology and Public Health Research Unit (L Monasta DSc, G Zamagni MSc), Burlo Garofolo Institute for Maternal and Child Health, Trieste, Italy; Faculty of Medicine (A Moodi Ghalibaf MD), Birjand University of Medical Sciences, Birjand, Iran; Department of Epidemiology and Biostatistics (Y Moradi PhD), Department of Microbiology (M Sedighi PhD), Kurdistan University of Medical Sciences, Sanandaj, Iran; Department of Health Policy (Prof E Mossialos PhD), London School of Economics and Political Science, London, UK; Department of Surgery (G D Mukoro MD), Ahmadu Bello University Teaching Hospital, Zaria, Nigeria; Department of Surgery (F Mulita PhD), General University Hospital of Patras, Patras, Greece; Faculty of Medicine (F Mulita PhD), University of Thessaly, Larissa, Greece; Clinical and Public Health Group (Prof J V Robotham PhD), UK Health Security Agency, London, UK (B Muller-Pebody PhD); Clinical Epidemiology Research Unit (E Murillo-Zamora PhD), Mexican Institute of Social Security, Villa de Alvarez, Mexico; Postgraduate in Medical Sciences (E Murillo-Zamora PhD), Universidad de Colima (Colima University), Colima, Mexico; Pathogen and Microbes Programme (P Musicha PhD), Wellcome Sanger Institute, Cambridge, UK; Department of Emerging Infectious Diseases (L A Musila PhD), United States Army Medical Research Institute, Kenya, Nairobi, Kenya; Prince Fahad bin Sultan Chair for Biomedical Research (S Muthupandian PhD), University of Tabuk, Tabuk, Saudi Arabia; Research and Analytics Department (A J Nagarajan MTech), Initiative for Financing Health and Human Development, Chennai, India; Department of Research and Analytics (A J Nagarajan MTech), Bioinsilico Technologies, Chennai, India; Department of Computer Science (P Naghavi MS), University of Illinois Urbana-Champaign, Urbana, IL, USA; Faculty of Pharmacy (F Nainu PhD), Hasanuddin University, Makassar, Indonesia; Department of Community Medicine (T S Nair MD), MOSC Medical College, Kolenchery, India; Department of Medical Laboratory Analysis (H H R Najmuldeen PhD), Cihan University Sulaymaniya, Sulaymaniyah, Iraq; Department of Health Policy and Oral Epidemiology (Z S Natto DrPH), Harvard University, Boston, MA, USA; Department of Circulation and Medical Imaging (J Nauman PhD), Norwegian University of Science and Technology, Trondheim, Norway; Department of Research (G Nchanji MSc), TroDDIVaT Initiative, Buea, Cameroon; Department of Microbiology and Parasitology (G Nchanji MSc), University of Buea, Buea, Cameroon; Research and Innovation Center (P Ndishimye PhD), Dalhousie University, Kigali, Rwanda; African Institute for Mathematical Sciences, Kigali, Rwanda (P Ndishimye PhD); Department of General Surgery (I Negoi PhD), Emergency University Hospital Bucharest, Bucharest, Romania; Department of Cardiology (R I Negoi PhD), Cardio-Aid, Bucharest, Romania; Department of Epidemiology (S Nejadghaderi MD), Non-Communicable Diseases Research Center (NCDRC), Tehran, Iran; Faculty of Applied Sciences and Technology (E A Noman PhD), Universiti Tun Hussein Onn Malaysia, Johor, Malaysia; Department of Health (D C Nwakanma PhD), Medical Research Council Unit, The Gambia, Banjul, The Gambia; Department of Gynecology and Obstetrics (T R Ojo-Akosile MD), Emory University, Atlanta, GA, USA; Department of Psychiatry and Behavioural Neurosciences (A T Olagunju MD), McMaster University, Hamilton, ON, Canada; Department of Psychiatry (A T Olagunju MD), University of Lagos, Lagos, Nigeria; Department of Biomedical Sciences (K I Onyedibe PhD), Mercer University School of Medicine, Macon, GA, USA; Dirección de Prestaciones

Económicas y Sociales (E Ortiz-Brizuela MD), Instituto Mexicano del Seguro Social, Mexico City, Mexico; Department of Biological Sciences (O Osuolale PhD), Elizade University, Ilara-Mokin, Nigeria; Department of Respiratory Medicine (Prof M P P A DNB), Jagadguru Sri Shivarathreeswara University, Mysore, India; Department of Epidemiology and Community Health (R R Parikh MD), University of Minnesota, Minneapolis, MN, USA; School of Dentistry (J Patel BSc), University of Leeds, Leeds, UK; College of Dental Medicine (Prof S Patil PhD), Roseman University of Health Sciences, South Jordan, UT, USA; Monash Biomedicine Discovery Institute (Prof A Y Peleg PhD), Monash University, Clayton, VIC, Australia; Australian Institute of Health Innovation (P Peprah MSc), Macquarie University, Sydney, NSW, Australia; Research Institute for Medicines (Prof J Perdigão PhD), Universidade de Lisboa (University of Lisbon), Lisbon, Portugal; Department of Statistics and Econometrics (I Petcu PhD), Bucharest University of Economic Studies, Bucharest, Romania; International Center of Medical Sciences Research, Islamabad, Pakistan (Z Z Piracha PhD); Department of Medicine (Prof D Poddighe PhD), Nazarbayev University, Astana, Kazakhstan; Clinical Academic Department of Pediatrics (Prof D Poddighe PhD), University Medical Center (UMC), Astana, Kazakhstan; NIHR Oxford Biomedical Research Centre (Prof A J Pollard FRS), Oxford University Hospitals, Oxford, UK; Department of Data Management and Analysis (R Poluru PhD), The International Clinical Epidemiology Network (INCLEN) Trust International, New Delhi, India; Department of Biostatistics, Epidemiology, and Informatics (J Puvvula PhD), Department of Otorhinolaryngology (P S Ramesh PhD), University of Pennsylvania, Philadelphia, PA, USA; Cihan University-Sulaimaniya Research Center (N H Qasim DSc), Cihan University-Sulaimaniya, Sulaymaniyah, Iraq; Infectious and Tropical Diseases Research Center (L Rahbarnia PhD), Tabriz University of Medical Sciences, Tabriz, Iran; Department of Medical Laboratory Technologies (Prof F Rahim PhD), Al-Noor Center of Research and Innovation (Prof F Rahim PhD), Alnoor University, Mousl, Iraq; Department of Population Science and Human Resource Development (Prof M Rahman DrPH), University of Rajshahi, Rajshahi, Bangladesh; Institute of Health and Wellbeing (Prof M Rahman PhD), Federation University Australia, Berwick, VIC, Australia; Department of Hygiene and Zoonoses (H Ramadan PhD), Faculty of Pharmacy (Prof M A Saleh PhD), Mansoura University, Mansoura, Egypt; Department of Biotechnology (Prof P W Ramteke PhD), Hislop College, Nagpur, India; Department of Molecular Biology & Genetic Engineering (Prof P W Ramteke PhD), RTM Nagpur University, Nagpur, India; Department of Community Medicine (R K Rana MD), Shaheed Nirmal Mahto Medical College and Hospital, Dhanbad, India; Department of Health Innovation (U Rani PhD), Manipal Academy of Higher Education, Udupi, India; Department of Family Medicine (Prof D Rathish MPH), Department of Parasitology (Prof K G Weerakoon PhD), Rajarata University of Sri Lanka, Anuradhapura, Sri Lanka; Emergency Department (S Rattanavong MD), Savannakhet Provincial Hospital, Savannakhet, Lao, Laos; Academic Public Health England (Prof S Rawaf MD), Public Health England, London, UK; Department of Biological Sciences (Prof E M M Redwan PhD), King Abdulaziz University, Jeddah, Egypt; Department of Protein Research (Prof E M M Redwan PhD), Research and Academic Institution, Alexandria, Egypt; Department of Biochemistry and Food Analysis (N Roy PhD), Patuakhali Science and Technology University, Patuakhali, Bangladesh; Department of Critical Care Medicine (K E Rudd MD), University of Pittsburgh, Pittsburgh, PA, USA; Department of Medicine (C J Sabet MA), Georgetown University, Washington, DC, USA; Department of Pharmaceutical Chemistry (Prof M Saeb PhD), International Medical University, Gdańsk, Poland; Clinical and Biomedical Research Center (Prof U Saeed PhD), Foundation University Islamabad, Islamabad, Pakistan; International Center of Medical Sciences Research (ICMSR), Islamabad, Pakistan (Prof U Saeed PhD); Kiel Institute for the World Economy, Kiel, Germany (S Saeedi Moghaddam MSc); School of Dentistry (Prof M Safaei PhD), Kermanshah University of Medical Sciences, Kermanshah, Iran;

Biotechnology Research Center (Prof A Sahebkar PhD), Department of Medicine (A Yarahmadi PhD), Mashhad University of Medical Sciences, Mashhad, Iran; Department of Community Medicine and Family Medicine (S S Sahoo MD), All India Institute of Medical Sciences, Bathinda, India; Department of Biosciences (N Salam PhD), Centre for Interdisciplinary Research In Basic Sciences (CIRBSc) (A Shamsi PhD), Jamia Millia Islamia, New Delhi, India; Department of Pharmacy Practice (Z Saleem PhD), Bahauddin Zakariya University, Multan, Pakistan; Institute of Epidemiology and Preventive Medicine (Y L Samodra PhD), National Taiwan University, Taipei, Taiwan; Benang Merah Research Center (BMRC), Minahasa Utara, Indonesia (Y L Samodra PhD); Department of Entomology (A M Samy PhD), Medical Ain Shams Research Institute (MASRI) (A M Samy PhD), Ain Shams University, Cairo, Egypt; Indira Gandhi Medical College and Research Institute, Puducherry, India (A Saravanan MD); UGC Centre of Advanced Study in Psychology (M Satpathy PhD), Department of Analytical and Applied Economics (C Swain MPhil), Utkal University, Bhubaneswar, India; Udyam-Global Association for Sustainable Development, Bhubaneswar, India (M Satpathy PhD); Department of Microbiology (P A Shah MBBS), Rajiv Gandhi University of Health Sciences, Bangalore, India; Department of Pathobiology (M Shamshirgaran PhD), Shahid Bahonar University of Kerman, Kerman, Iran; Department for Evidence-based Medicine and Evaluation (A Sharifan PharmD), University for Continuing Education Krems, Krems, Austria; Yenepoya Research Center (R P Shastry PhD), Yenepoya University, Mangalore, India; K S Hegde Medical Academy (Prof M Shetty MD), Nitte University, Mangalore, India; Department of Veterinary Public Health and Preventive Medicine (A Shittu MSc), Usmanu Danfodiyo University, Sokoto, Sokoto, Nigeria; Department of Research and Academics (S Shrestha PharmD), Kathmandu Cancer Center, Bhaktapur, Nepal; Department of Medical Microbiology and Infectious Diseases (E E Siddig MD), Erasmus University, Rotterdam, Netherlands; Department of Foodborne and Waterborne Diseases (T Sideroglou MSc), National Public Health Organization, Athens, Greece; Department of Medicine (Prof J Sifuentes-Osornio MD), National Institute of Nutrition, Tlalpan, Mexico; Sport Physical Activity and Health Research & Innovation Center (SPRINT) (Prof L M R Silva PhD), Polytechnic Institute of Guarda, Guarda, Portugal; CICS-UBI Health Sciences Research Center (Prof L M R Silva PhD), University of Beira Interior, Covilhã, Portugal; Division of Infectious Diseases (Prof E A F Simões MD), University of Colorado School of Medicine, Aurora, CO, USA; Samshoma Medical Research Inc, Denver, CO, USA (Prof E A F Simões MD); Mahosot Hospital (A J H Simpson FRCPath), Lao-Oxford-Mahosot Hospital-Wellcome Trust Research Unit (LOMWRU), Vientiane, Loas; Department of Microbiology (A Singh PhD, M K Yadav PhD), Central University of Punjab, Bathinda, India; Department of Laboratory Medicine (A Singh PhD), All India Institute of Medical Sciences, New Delhi, India; Department of Internal Medicine (R Sinto MD), University of Indonesia, Jakarta Pusat, Indonesia; Department of Internal Medicine (R Sinto MD), Dr. Cipto Mangunkusumo National Hospital, Jakarta Pusat, Indonesia; Urmia University of Medical Sciences, Urmia, Iran (S Soraneh MD); Department of Infectious Diseases and Microbiology (T Wangrangsimakul MB), Oxford University Hospitals NHS Foundation Trust, Oxford, UK (Prof N Stoesser DPhil); Microbiology Laboratory (Prof T Z Stoeva DSc), University Hospital, Varna, Bulgaria; Henry JN Taub Department of Emergency Medicine (Prof L Szarpak PhD), Baylor College of Medicine, Houston, TX, USA; Department of Clinical Research and Development (Prof L Szarpak PhD), LUXMED Group, Warsaw, Poland; Department of Pharmacology (S T Y MD), All India Institute of Medical Sciences, Deoghar, India; Duhok Research Centre (Z M Taha BMedSc), University of Duhok, Duhok, Iraq; Department of Surgery (K Tan PhD), National University of Singapore, Singapore, Singapore; Department of Microbiology (N Tasak MPH), Chiang Rai Clinical Research Unit (A Thaiprakong BSc), Mahidol Oxford Tropical Medicine Research Unit, Chiang Rai, Thailand; Department of Economics (N Y Tat MS), Rice University, Houston,

TX, USA; Department of Research and Innovation (N Y Tat MS), Enventure Medical Innovation, Houston, TX, USA; Department of Pharmacology (P Thangaraju MD), All India Institute of Medical Sciences, Raipur, India; Childhood Acute Illness & Nutrition (CHAIN) Network, Nairobi, Kenya (C C Tigoi DPhil); Department of Business Analytics (T H Tran MD), University of Massachusetts Dartmouth, Dartmouth, MA, USA; Department of Internal Medicine (M Tumurkhuu PhD), Wake Forest University, Winston-Salem, NC, USA; Angkor Hospital for Children (Prof P Turner PhD), Cambodia Oxford Medical Research Unit, Siem Reap, Cambodia; Faculty of Health and Life Sciences (A Udoh PhD), University of Exeter, Exeter, UK; International Center for Chemical and Biological Sciences (S Ullah MSc), University of Karachi, Karachi, Pakistan; College of Health and Sport Sciences (A G Vaithinathan MSc), University of Bahrain, Zallaq, Bahrain; Department of Biomedical Sciences (M Valenti MD), Humanitas University, Milan, Italy; Dermatology Unit (M Valenti MD), IRCCS Humanitas Research Hospital, Milan, Italy; Oxford University Clinical Research Unit Vietnam, Hanoi, Vietnam (H T L Vu PhD); School of Health Sciences (Prof Y Waheed PhD), National University of Sciences and Technology (NUST), Islamabad, Pakistan; Lebanese American University, Byblos, Lebanon (Prof Y Waheed PhD); Department of Medical Microbiology (Prof H F L Wertheim PhD), Radboud University Medical Center, Nijmegen, Netherlands; Department of Infectious Diseases (P C M Williams DPhil) Sydney Children's Hospital Network, Randwick, NSW, Australia; National Data Management Center for Health (NDMC) (A A Wolde MPH), Ethiopian Public Health Institute, Addis Ababa, Ethiopia; Australian e-Health Research Centre (T M Wozniak PhD), Commonwealth Scientific and Industrial Research Organisation, Herston, QLD, Australia; Global and Tropical Health Division (T M Wozniak PhD), Menzies School of Health Research, Darwin, NT, Australia; Department of Food Science and Human Nutrition (Prof F Wu PhD), Michigan State University, East Lansing, MI, USA; Division of Gastroenterology (Prof Z Wu PhD), Huazhong University of Science and Technology, Wuhan, China; Department of Basic Medical Sciences (S Yaghoubi PhD), Neyshabur University of Medical Sciences, Neyshabur, Iran; Department of Medicine (A Yarahmadi PhD), Shiraz University of Medical Sciences, Shiraz, Iran; Biostatics, Epidemiology, and Science Computing Department (S Yezli PhD), King Faisal Specialist Hospital & Research Center, Riyadh, Saudi Arabia; Pharmacy Department (Y Yismaw MSc), Alkan Health Science, Business and Technology College, Bahir Dar, Ethiopia; Department of Pediatrics (Prof D Yon MD), Kyung Hee University, Seoul, South Korea; Department of Clinical Pharmacy and Pharmacy Administration (H Yusuf PhD), University of Maiduguri, Maiduguri, Nigeria; Faculty of Medicine and Health Sciences (F Zakham PhD), Hodeidah University, Hodeidah, Yemen; Department of Virology (F Zakham PhD), University of Helsinki, Helsinki, Finland; School of Public Health (H Zhang MS), Peking University, Beijing, China; School of Public Health (Prof Z Zhang PhD), Wuhan University, Wuhan, China; Department of Biochemistry and Pharmacogenomics (M Zielińska MPharm), Medical University of Warsaw, Warsaw, Poland; NIHR-Biomedical Research Centre (NIHR-BRC) (Prof A Zumla PhD), University College London Hospitals, London, UK; Department of Clinical and Community Pharmacy (Prof S H Zyoud PhD), An-Najah National University Hospital, Clinical Research Centre (Prof S H Zyoud PhD), An-Najah National University, Nablus, Palestine; Faculty of Medicine (B Sartorius PhD), The University of Queensland, Brisbane, QLD, Australia.

Authors' Contributions

Managing the overall research enterprise

Ben S Cooper, Christiane Dolecek, Simon I Hay, Paulina A Lindstedt, Barney McManigal, Christopher J L Murray, Mohsen Naghavi, Amanda E Smith, Andy Stergachis, Stein Emil Vollset, and Eve E Wool.

Writing the first draft of the manuscript

Natalia V Bhattacharjee, Nicole Davis Weaver, Kevin S Ikuta, Authia P Gray, Paulina A Lindstedt, Tomislav Mestrovic, Mohsen Naghavi, Georgia Smith, Amanda E Smith, Lucien R Swetschinski, Stein Emil Vollset, and Eve E Wool.

Primary responsibility for applying analytical methods to produce estimates

Umut Altay, Daniel T Araki, Natalia V Bhattacharjee, Julian Chalek, Erin Chung, Ben S Cooper, Christiane Dolecek, Nattwut Ekaphirat, Frederick Fell, Anna Gershberg Hayoon, Konstantinos Giannakis, Authia P Gray, Chieh Han, Rebecca L Hsu, Jessica Andretta Mendes, Mohsen Naghavi, Catalina Raggi, Gisela Robles Aguilar, Tilleye Runghien, Georgia Smith, Oraya Srimokla, Lucien R Swetschinski, and Stein Emil Vollset.

Primary responsibility for seeking, cataloguing, extracting, or cleaning data; designing or coding figures and tables

Umut Altay, Daniel T Araki, Julian Chalek, Anna Gershberg Hayoon, Konstantinos Giannakis, Authia P Gray, Chieh Han, Rebecca L Hsu, Barney McManigal, Gisela Robles Aguilar, Georgia Smith, and Lucien R Swetschinski.

Providing data or critical feedback on data sources

Richard Gyan Aboagye, Hassan Abolhassani, Ahmed Abu-Zaid, Sureerak Achalapong, Victor Adekanmbi, Temitayo Esther Adeyeoluwa, Qorinah Estiningtyas Sakilah Adnani, Leticia Akua Adzigbli, Muhammad Sohail Afzal, Saira Afzal, Antonella Agodi, Sajjad Ahmad, Tauseef Ahmad, Ali Ahmadi, Ayman Ahmed, Haroon Ahmed, Mohammed Ahmed, Saeed Ahmed, Syed Anees Ahmed, Salah Al Awaidy, Mohammad T AlBataineh, Abid Ali, Liaqat Ali, Syed Shujait Ali, Kasim Allel, Awais Altaf, Alaa B Al-Tammemi, Ben Amos, John H Amuasi, Jason R Andrews, Saeid Anvari, Geminn Louis Carace Apostol, Jalal Arabloo, Anton A Artamonov, Elizabeth A Ashley, Marvellous O Asika, Seyyed Shamsadin Athari, James Mba Azam, Ahmed Y Azzam, Mahsa Babaei, Francois-Xavier Babin, Atif Amin Baig, Stephen Baker, Mainak Bardhan, Hiba Jawdat Barqawi, Mathieu Bastard, Nebiyou Simegnew Bayleyegn, Melaku Ashagrie Belete, Olorunjuwon Omolaja Bello, Apostolos Beloukas, James A Berkley, Akshaya Srikanth Bhagavathula, Sonu Bhaskar, Soumitra S Bhuyan, Julia A Bielicki, Colin Stewart Brown, Annie J Browne, Danilo Buonsenso, Cristina G Carvalheiro, Carlos A Castañeda-Orjuela, Rama Mohan Chandika, Sara Chandy, Vilada Chansamouth, Vijay Kumar Chattu, Hitesh Chopra, Fazle Rabbi Chowdhury, Dinh-Toi Chu, Ben S Cooper, Natalia Cruz-Martins, Xiaochen Dai, Samuel D Darcho, Saswati Das, Fernando Pio De la Hoz, Denise Myriam Dekker, Benjamin Felix Rothschild Dickson, Serge Ghislain Djorie, Milad Dodangeh, Klara Georgieva Dokova, Christiane Dolecek, Ojas Prakashbhai Doshi, Robert Kokou Dowou, Haneil Larson Dsouza, Susanna J Dunachie, Tim Eckmanns, Aziz Eftekharimehrabad, Temitope Cyrus Ekundayo, Christelle Elias, Sally J Ellis, Chadi Eltaha, Majid Eslami, David William Eyre, Adewale Oluwaseun Fadaka, Adeniyi Francis Fagbamigbe, Ali Fatehizadeh, Nicholas A Feasey, Alireza Feizkhah, Ginenus Fekadu, Frederick Fell, Karen M Forrest, Celia Fortuna Rodrigues, John E Fuller, Muktar A Gadanya, Esteban E Garcia-Gallo, Denise O Garrett, Teferi Gebru Gebremeskel, Christine Geffers, Leonidas Georgalis, Mahaveer Golechha, Melita Gordon, Authia P Gray, Rajat Das Gupta, Sapna Gupta, Vijai Kumar Gupta, Sebastian Haller, Chieh Han, Harapan Harapan, Michelle L Harrison, Rumina Syeda Hasan, Andrea Haekyung Haselbeck, Shoaib Hassan, Mahgol Sadat Hassan Zadeh Tabatabaei, Simon I Hay, Marianne Holm, Heidi Hopkins, Md Mahbub Hossain, Mehdi Hosseinzadeh, Nawfal R Hussein, Elsa D Ibáñez-Prada, Kevin S Ikuta, Nahlah Elkudssiah Ismail, Chidozie Declan Iwu, Fatoumatta Jaiteh, Adam W J Jenney, Hyon Jin Jeon, Tamas Joo,

Zul Kamal, Rami S Kantar, James Apollo Kapisi, Yousef Saleh Khader, Himanshu Khajuria, Nauman Khalid, Faham Khamesipour, Ajmal Khan, Mohammad Jobair Khan, Muhammad Tariq Khan, Jagdish Khubchandani, Suwimon Khusuwan, Min Seo Kim, Adnan Kisa, Fiorella Krapp, Ralf Krumkamp, Emmanuelle A P Kumaran, Hmwe Hmwe Kyu, Iván Landires, Thao Thi Thu Le, Ingeborg Maria Lederer, Munjae Lee, Seung Won Lee, Alain Lepape, Virendra S Ligade, Cherry Lim, Stephen S Lim, Xuefeng Liu, Michael J Loftus, Panagiota Maikanti-Charalampous, Omar M Makram, Kashish Malhotra, Georgia D Mandilara, Florian Marks, Bernardo Alfonso Martinez-Guerra, Hossein Masoumi-Asl, Alexander G Mathioudakis, Juergen May, Barney McManigal, Endalkachew Belayneh Melese, Jessica Andretta Mendes, Tomislav Mestrovic, Nouh Saad Mohamed, Shafiu Mohammed, Syam Mohan, Ali H Mokdad, Lorenzo Monasta, Catrin E Moore, Yousef Moradi, Vincent Mougin, George Duke Mukoro, Francesk Mulita, Berit Muller-Pebody, Efren Murillo-Zamora, Christopher J L Murray, Patrick Musicha, Lillian A Musila, Saravanan Muthupandian, Ahamarshan Jayaraman Nagarajan, Mohsen Naghavi, Zuhair S Natto, Biswa Prakash Nayak, Ionut Negoi, Ruxandra Irina Negoi, QuynhAnh P Nguyen, Davis C Nwakanma, Seamus O'Brien, Theresa J Ochoa, Ismail A Odetokun, Oluwaseun Adeolu Ogundijo, Osaretin Christabel Okonji, Andrew T Olagunju, Antonio Olivas-Martinez, Peter Olwoch, Edgar Ortiz-Brizuela, Olayinka Osuolale, Pradthana Ounchanum, Mahesh Padukudru P A, Jose L Paredes, Romil R Parikh, Jay Patel, Shankargouda Patil, Shrikant Pawar, Anton Y Peleg, Prince Peprah, Carlo Perrone, Koukeo Phommasone, Zahra Zahid Piracha, Andrew J Pollard, Ramesh Poluru, Alfredo Ponce-De-Leon, Jagadeesh Puvvula, Clotaire Donatien Rafai, Pankaja Raghav, Fakher Rahim, Vafa Rahimi-Movaghar, Shakthi Kumaran Ramasamy, Pushkal Sinduvadi Ramesh, Rishabh Kumar Rana, Sayaphet Rattanavong, Salman Rawaf, Luis Felipe Reyes, Tamalee Roberts, Gisela Robles Aguilar, Julie V Robotham, Victor Daniel Rosenthal, Kristina E Rudd, Tilleye Runghien, Cameron John Sabet, Basema Ahmad Saddik, Umar Saeed, Weeravoot Saengchan, Maitreyi Sahu, Zikria Saleem, Abdallah M Samy, Benn Sartorius, Maheswar Satpathy, Mansour Sedighi, Samroeng Seekaew, Pritik A Shah, Sadia Shakoor, Muhammad Aaqib Shamim, Anas Shamsi, Amin Sharifan, Rajesh P Shastry, Aminu Shittu, Sunil Shrestha, Theologia Sideroglou, Jose Sifuentes-Osornio, Luís Manuel Lopes Rodrigues Silva, Eric A F Simões, Andrew J H Simpson, Oraya Srimokla, Andy Stergachis, Nicole Stoesser, Temenuga Zhekova Stoeva, Chandan Kumar Swain, Sree Sudha T Y, Shima Tabatabai, Zanan Mohammed-Ameen Taha, Ker-Kan Tan, Nidanuch Tasak, Areerat Thaiprakong, Pugazhenthan Thangaraju, Caroline Chepngeno Tigoi, Marcos Roberto Tovani-Palone, Paul Turner, Noor Ullah, Theo Vos, Huong T L Vu, Yasir Waheed, Ann Sarah Walker, Judd L Walson, Tri Wangrangsimakul, Kosala Gayan Weerakoon, Heiman F L Wertheim, Phoebe C M Williams, Teresa M Wozniak, Felicia Wu, Sajad Yaghoubi, Amir Yarahmadi, Dong Keon Yon, Magdalena Zielińska, and Alimuddin Zumla.

Developing methods or computational machinery

Qorinah Estiningtyas Sakilah Adnani, Saira Afzal, Austin J Ahlstrom, Ali Ahmadi, Mohammed Ahmed, Daniel T Araki, Aleksandr Y Aravkin, Ahmed Y Azzam, Atif Amin Baig, Hiba Jawdat Barqawi, Akshaya Srikanth Bhagavathula, Natalia V Bhattacharjee, Annie J Browne, Julian Chalek, Hitesh Chopra, Dinh-Toi Chu, Erin Chung, Xiaochen Dai, Christiane Dolecek, Susanna J Dunachie, Iman El Sayed, Adeniyi Francis Fagbamigbe, Ali Fatehizadeh, Anna Gershberg Hayoon, Authia P Gray, Chieh Han, Mahgol Sadat Hassan Zadeh Tabatabaei, Jiawei He, Mohammad Heidari, Mehdi Hosseinzadeh, Rebecca L Hsu, Elsa D Ibáñez-Prada, Kevin S Ikuta, Reza Jalilzadeh Yengejeh, Adnan Kisa, Emmanuelle A P Kumaran, Thao Thi Thu Le, Kelsey Lynn Maass, Ali H Mokdad, Yousef Moradi, Vincent Mougin, Francesk Mulita, Christopher J L

Murray, Mohsen Naghavi, Pirouz Naghavi, QuynhAnh P Nguyen, Zahra Zahid Piracha, Catalina Raggi, Gisela Robles Aguilar, Julie V Robotham, Kristina E Rudd, Tilleye Runghien, Umar Saeed, Abdallah M Samy, Benn Sartorius, Maheswar Satpathy, Austin E Schumacher, Georgia Smith, Chandan Kumar Swain, Lucien R Swetschinski, Zanan Mohammed-Ameen Taha, Stein Emil Vollset, and Chun-Wei Yuan.

Providing critical feedback on methods or results

Atef Abdelkader, Sherief Abd-Elsalam, Richard Gyan Aboagye, Hassan Abolhassani, Hasan Abualruz, Hana J Abukhadijah, Salahdein Aburuz, Ahmed Abu-Zaid, Isaac Yeboah Addo, Victor Adekanmbi, Temitayo Esther Adeyeoluwa, Qorinah Estiningtyas Sakilah Adnani, Leticia Akua Adzigbli, Muhammad Sohail Afzal, Saira Afzal, Antonella Agodi, Aqeel Ahmad, Sajjad Ahmad, Tauseef Ahmad, Ali Ahmadi, Ayman Ahmed, Haroon Ahmed, Ibrar Ahmed, Mohammed Ahmed, Saeed Ahmed, Syed Anees Ahmed, Mohammed Ahmed Akkaif, Salah Al Awaidy, Yazan Al Thaher, Samer O Alalalmeh, Mohammad T AlBataineh, Adel Ali Saeed Al-Gheethi, Nma Bida Alhaji, Abid Ali, Liaqat Ali, Syed Shujait Ali, Waad Ali, Kasim Allel, Sabah Al-Marwani, Awais Altaf, Alaa B Al-Tammemi, Jaffar A Al-Tawfiq, Umut Altay, Karem H Alzoubi, Walid Adnan Al-Zyoud, John H Amuasi, Robert Ancuceanu, Iyadunni Adesola Anuoluwa, Saeid Anvari, Anayochukwu Edward Anyasodor, Geminn Louis Carace Apostol, Jalal Arabloo, Mosab Arafat, Daniel T Araki, Demelash Areda, Anton A Artamonov, Elizabeth A Ashley, Marvellous O Asika, Seyyed Shamsadin Athari, Maha Moh'd Wahbi Atout, Tewachew Awoke, Sina Azadnajafabad, James Mba Azam, Shahkaar Aziz, Ahmed Y Azzam, Mahsa Babaei, Muhammad Badar, Atif Amin Baig, Mainak Bardhan, Hiba Jawdat Barqawi, Zarrin Basharat, Mathieu Bastard, Saurav Basu, Nebiyou Simegnew Bayleyegn, Melaku Ashagrie Belete, Olorunjuwon Omolaja Bello, Apostolos Beloukas, James A Berkley, Akshaya Srikanth Bhagavathula, Sonu Bhaskar, Natalia V Bhattacharjee, Soumitra S Bhuyan, Colin Stewart Brown, Annie J Browne, Danilo Buonsenso, Yasser Bustanji, Carlos A Castañeda-Orjuela, Muthia Cenderadewi, Joshua Chadwick, Rama Mohan Chandika, Sara Chandy, Vijay Kumar Chattu, Hitesh Chopra, Dinh-Toi Chu, Erin Chung, Muhammad Chutiyami, Natalia Cruz-Martins, Alanna Gomes da Silva, Omid Dadras, Xiaochen Dai, Samuel D Darcho, Saswati Das, Nicole Davis Weaver, Fernando Pio De la Hoz, Kuldeep Dhama, Daniel Diaz, Benjamin Felix Rothschild Dickson, Milad Dodangeh, Christiane Dolecek, Ojas Prakashbhai Doshi, Robert Kokou Dowou, Haneil Larson Dsouza, Susanna J Dunachie, Arkadiusz Marian Dziedzic, Tim Eckmanns, Abdelaziz Ed-Dra, Aziz Eftekharimehrabad, Nattwut Ekapirat, Temitope Cyrus Ekundayo, Iman El Sayed, Muhammed Elhadi, Waseem El-Huneidi, Randa Elsheikh, Ibrahim Elsohaby, Chadi Eltaha, Babak Eshrati, Majid Eslami, Adewale Oluwaseun Fadaka, Adeniyi Francis Fagbamigbe, Ayesha Fahim, Aliasghar Fakhri-Demeshghieh, Ali Fatehizadeh, Nicholas A Feasey, Alireza Feizkhah, Ginenus Fekadu, Florian Fischer, Ida Fitriana, Celia Fortuna Rodrigues, Muktar A Gadanya, Márió Gajdács, Aravind P Gandhi, Rupesh K Gautam, Miglas Welay Gebregergis, Mesfin Gebrehiwot, Teferi Gebru Gebremeskel, Leonidas Georgalis, Ramy Mohamed Ghazy, Konstantinos Giannakis, Mahaveer Golechha, Davide Golinelli, Authia P Gray, Rajat Das Gupta, Sapna Gupta, Vijai Kumar Gupta, Awoke Derbie Habteyohannes, Sebastian Haller, Harapan Harapan, Ahmed I Hasaballah, Ikramul Hasan, Rumina Syeda Hasan, Hamidreza Hasani, Md Saquib Hasnain, Ikrama Ibrahim Hassan, Shoaib Hassan, Mahgol Sadat Hassan Zadeh Tabatabaei, Simon I Hay, Khezar Hayat, Mohammad Heidari, Kamal Hezam, Ramesh Holla, Md Mahbub Hossain, Mehdi Hosseinzadeh, Rebecca L Hsu, Nawfal R Hussein, Le Duc Huy, Elsa D Ibáñez-Prada, Kevin S Ikuta, Irena M Ilic, Sheikh Mohammed Shariful Islam, Faisal Ismail, Nahlah Elkudssiah Ismail, Chidozie Declan Iwu, Chinwe Juliana Iwu-Jaja, Reza Jalilzadeh Yengejeh, Roland Dominic G Jamora, Javad Javidnia, Talha Jawaid, Adam W J Jenney, Mohammad Jokar, Nabi Jomehzadeh, Tamas Joo, Nitin Joseph, Kehinde Kazeem Kanmodi, Rami S Kantar, Ibraheem M Karaye, Yousef Saleh Khader, Himanshu Khajuria, Nauman Khalid, Faham Khamesipour, Mohammad Jobair Khan, Vishnu Khanal, Feriha Fatima Khidri, Jagdish

Khubchandani, Min Seo Kim, Adnan Kisa, Vladimir Andreevich Korshunov, Mohammed Kuddus, Dewesh Kumar, Ambily Kuttikkattu, Hmwe Hmwe Kyu, Iván Landires, Basira Kankia Lawal, Thao Thi Thu Le, Munjae Lee, Seung Won Lee, Temesgen Leka Lerango, Virendra S Ligade, Stephen S Lim, Liknaw Workie Limenh, Xiaofeng Liu, Xuefeng Liu, Hawraz Ibrahim M Amin, Kelsey Lynn Maass, Sandeep B Maharaj, Mansour Adam Mahmoud, Omar M Makram, Kashish Malhotra, Ahmad Azam Malik, Bernardo Alfonso Martinez-Guerra, Miquel Martorell, Alexander G Mathioudakis, James Meiring, Hadush Negash Meles, Addisu Melese, Endalkachew Belayneh Melese, Tomislav Mestrovic, Giuseppe Minervini, Nouh Saad Mohamed, Shafiu Mohammed, Syam Mohan, Ali H Mokdad, AmirAli Moodi Ghalibaf, Catrin E Moore, Yousef Moradi, Elias Mossialos, George Duke Mukoro, Francesk Mulita, Efren Murillo-Zamora, Christopher J L Murray, Sani Musa, Saravanan Muthupandian, Ahamarshan Jayaraman Nagarajan, Mohsen Naghavi, Pirouz Naghavi, Firzan Nainu, Tapas Sadasivan Nair, Hastyar Hama Rashid Najmuldeen, Zuhair S Natto, Javaid Nauman, Biswa Prakash Nayak, G Takop Nchanji, Pacifique Ndishimye, Ionut Negoi, Ruxandra Irina Negoi, Seyed Aria Nejadghaderi, Efaq Ali Noman, Seamus O'Brien, Theresa J Ochoa, Ismail A Odetokun, Oluwaseun Adeolu Ogundijo, Tolulope R Ojo-Akosile, Osaretin Christabel Okonji, Andrew T Olagunju, Antonio Olivas-Martinez, Abdulhakeem Abayomi Olorukooba, Kenneth Ikenna Onyedibe, Edgar Ortiz-Brizuela, Oyetunde T Oyeyemi, Mahesh Padukudru P A, Romil R Parikh, Jay Patel, Shankargouda Patil, Shrikant Pawar, Prince Peprah, Ionela-Roxana Petcu, Koukeo Phommasone, Zahra Zahid Piracha, Ramesh Poluru, Jagadeesh Puvvula, Nameer Hashim Qasim, Pankaja Raghav, Leila Rahbarnia, Fakher Rahim, Vafa Rahimi-Movaghar, Mosiur Rahman, Muhammad Aziz Rahman, Shakthi Kumaran Ramasamy, Pushkal Sinduvadi Ramesh, Pramod W Ramteke, Rishabh Kumar Rana, Usha Rani, Mohammad-Mahdi Rashidi, Devarajan Rathish, Salman Rawaf, Elrashdy Moustafa Mohamed Redwan, Luis Felipe Reyes, Victor Daniel Rosenthal, Allen Guy Ross, Kristina E Rudd, Tilleye Runghien, Cameron John Sabet, Basema Ahmad Saddik, Mohammad Reza Saeb, Umar Saeed, Sahar Saeedi Moghaddam, Amene Saghazadeh, Narjes Saheb Sharif-Askari, Soumya Swaroop Sahoo, Maitreyi Sahu, Morteza Saki, Zikria Saleem, Mohamed A Saleh, Yoseph Leonardo Samodra, Abdallah M Samy, Benn Sartorius, Maheswar Satpathy, Mansour Sedighi, Mahan Shafie, Pritik A Shah, Samiah Shahid, Sadia Shakoor, Muhammad Aaqib Shamim, Mohammad Ali Shamshirgaran, Anas Shamsi, Amin Sharifan, Rajesh P Shastry, Aminu Shittu, Sunil Shrestha, Emmanuel Edwar Siddig, Jose Sifuentes-Osornio, Luís Manuel Lopes Rodrigues Silva, Eric A F Simões, Amit Singh, Amanda E Smith, Georgia Smith, Sameh S M Soliman, Soroush Soraneh, Andy Stergachis, Temenuga Zhekova Stoeva, Chandan Kumar Swain, Lucien R Swetschinski, Lukasz Szarpak, Sree Sudha T Y, Shima Tabatabai, Celine Tabche, Zanan Mohammed-Ameen Taha, Ker-Kan Tan, Pugazhenthan Thangaraju, Krishna Tiwari, Marcos Roberto Tovani-Palone, Thang Huu Tran, Munkhtuya Tumurkhuu, Paul Turner, Aniefiok John Udoakang, Arit Udoh, Saeed Ullah, Mario Valenti, Stein Emil Vollset, Theo Vos, Yasir Waheed, Judd L Walson, Kosala Gayan Weerakoon, Phoebe C M Williams, Asrat Arja Wolde, Eve E Wool, Felicia Wu, Zenghong Wu, Mukesh Kumar Kumar Yadav, Zwanden Sule Yahaya, Amir Yarahmadi, Saber Yezli, Yazachew Engida Engida Engida Yismaw, Dong Keon Yon, Hadiza Yusuf, Fathiah Zakham, Giulia Zamagni, Haijun Zhang, Zhi-Jiang Zhang, Magdalena Zielińska, Alimuddin Zumla, and Sa'ed H H Zyoud.

Drafting the work or revising it critically for important intellectual content

Atef Abdelkader, Sherief Abd-Elsalam, Hassan Abolhassani, Hasan Abualruz, Usman Abubakar, Hana J Abukhadijah, Salahdein Aburuz, Ahmed Abu-Zaid, Isaac Yeboah Addo, Victor Adekanmbi, Qorinah Estiningtyas Sakilah Adnani, Muhammad Sohail Afzal, Saira Afzal, Antonella Agodi, Ali Ahmadi, Ayman Ahmed, Haroon Ahmed, Ibrar Ahmed, Mohammed Ahmed, Saeed Ahmed, Syed Anees Ahmed, Mohammed Ahmed Akkaif, Yazan Al Thaher, Samer O Alalalmeh, Mohammad T AlBataineh, Wafa A

Aldhaleei, Nma Bida Alhaji, Abid Ali, Liaqat Ali, Syed Shujait Ali, Waad Ali, Kasim Allel, Ahmad Alrawashdeh, Awais Altaf, Alaa B Al-Tammemi, Jaffar A Al-Tawfiq, Umut Altay, Karem H Alzoubi, Walid Adnan Al-Zyoud, John H Amuasi, Robert Ancuceanu, Jason R Andrews, Abhishek Anil, Iyadunni Adesola Anuoluwa, Saeid Anvari, Anayochukwu Edward Anyasodor, Geminn Louis Carace Apostol, Jalal Arabloo, Daniel T Araki, Abdulfatai Aremu, Marvellous O Asika, Seyyed Shamsadin Athari, Maha Moh'd Wahbi Atout, Sina Azadnajafabad, Shahkaar Aziz, Ahmed Y Azzam, Mahsa Babaei, Muhammad Badar, Atif Amin Baig, Milica Bajcetic, Mainak Bardhan, Hiba Jawdat Barqawi, Zarrin Basharat, Afisu Basiru, Olorunjuwon Omolaja Bello, Apostolos Beloukas, Akshaya Srikanth Bhagavathula, Sonu Bhaskar, Natalia V Bhattacharjee, Soumitra S Bhuyan, Julia A Bielicki, Nikolay Ivanovich Briko, Colin Stewart Brown, Annie J Browne, Danilo Buonsenso, Yasser Bustanji, Cristina G Carvalheiro, Carlos A Castañeda-Orjuela, Muthia Cenderadewi, Joshua Chadwick, Sandip Chakraborty, Rama Mohan Chandika, Sara Chandy, Vijay Kumar Chattu, Anis Ahmad Chaudhary, Patrick R Ching, Erin Chung, Muhammad Chutiyami, Ben S Cooper, Natalia Cruz-Martins, Alanna Gomes da Silva, Samuel D Darcho, Nicole Davis Weaver, Daniel Diaz, Milad Dodangeh, Sushil Dohare, Christiane Dolecek, Ojas Prakashbhai Doshi, Robert Kokou Dowou, Haneil Larson Dsouza, Arkadiusz Marian Dziedzic, Aziz Eftekharimehrabad, Nattwut Ekapirat, Iman El Sayed, Muhammed Elhadi, Christelle Elias, Sally J Ellis, Randa Elsheikh, Chadi Eltaha, Adeniyi Francis Fagbamigbe, Ayesha Fahim, Aliasghar Fakhri-Demeshghieh, Folorunso Oludayo Fasina, Modupe Margaret Fasina, Ali Fatehizadeh, Nicholas A Feasey, Florian Fischer, Ida Fitriana, Muktar A Gadanya, Márió Gajdács, Rupesh K Gautam, Miglas Welay Gebregergis, Christine Geffers, Anna Gershberg Hayoon, Ramy Mohamed Ghazy, Konstantinos Giannakis, Davide Golinelli, Melita Gordon, Authia P Gray, Snigdha Gulati, Rajat Das Gupta, Sapna Gupta, Awoke Derbie Habteyohannes, Sebastian Haller, Chieh Han, Harapan Harapan, Ahmed I Hasaballah, Rumina Syeda Hasan, Hamidreza Hasani, Andrea Haekyung Haselbeck, Md Saquib Hasnain, Shoaib Hassan, Mahgol Sadat Hassan Zadeh Tabatabaei, Simon I Hay, Khezar Hayat, Omar E Hegazi, Kamal Hezam, Ramesh Holla, Marianne Holm, Md Mahbub Hossain, Sorin Hostiuc, Rebecca L Hsu, Elsa D Ibáñez-Prada, Adalia Ikiroma, Kevin S Ikuta, Irena M Ilic, Sheikh Mohammed Shariful Islam, Faisal Ismail, Nahlah Elkudssiah Ismail, Chidozie Declan Iwu, Chinwe Juliana Iwu-Jaja, Abdollah Jafarzadeh, Tamas Joo, Nitin Joseph, Kehinde Kazeem Kanmodi, Rami S Kantar, Himanshu Khajuria, Nauman Khalid, Ajmal Khan, Mohammad Jobair Khan, Feriha Fatima Khidri, Jagdish Khubchandani, Min Seo Kim, Adnan Kisa, Fiorella Krapp, Ralf Krumkamp, Mohammed Kuddus, Mukhtar Kulimbet, Dewesh Kumar, Iván Landires, Basira Kankia Lawal, Thao Thi Thu Le, Paulina A Lindstedt, Chaojie Liu, Xuefeng Liu, Michael J Loftus, Omar M Makram, Kashish Malhotra, Ahmad Azam Malik, Georgia D Mandilara, Bernardo Alfonso Martinez-Guerra, Miquel Martorell, Hossein Masoumi-Asl, Alexander G Mathioudakis, Juergen May, Theresa A McHugh, Hadush Negash Meles, Addisu Melese, Jessica Andretta Mendes, Tomislav Mestrovic, Nouh Saad Mohamed, Shafiu Mohammed, Ali H Mokdad, Lorenzo Monasta, AmirAli Moodi Ghalibaf, Catrin E Moore, Yousef Moradi, George Duke Mukoro, Francesk Mulita, Efren Murillo-Zamora, Christopher J L Murray, Sani Musa, Saravanan Muthupandian, Ahamarshan Jayaraman Nagarajan, Mohsen Naghavi, Zuhair S Natto, Javaid Nauman, Biswa Prakash Nayak, G Takop Nchanji, Pacifique Ndishimye, Ionut Negoi, Ruxandra Irina Negoi, Seyed Aria Nejadghaderi, Theresa J Ochoa, Ismail A Odetokun, Oluwaseun Adeolu Ogundijo, Sylvester Reuben Okeke, Osaretin Christabel Okonji, Andrew T Olagunju, Antonio Olivas-Martinez, Abdulhakeem Abayomi Olorukooba, Kenneth Ikenna Onyedibe, Edgar Ortiz-Brizuela, Mahesh Padukudru P A, Jose L Paredes, Romil R Parikh, Jay Patel, Shankargouda Patil, Shrikant Pawar, João Perdigão, Ionela-Roxana Petcu, Zahra Zahid Piracha, Dimitri Poddighe, Andrew J Pollard, Alfredo Ponce-De-Leon, Jagadeesh Puvvula, Farah Naz Qamar, Nameer Hashim Qasim, Catalina Raggi, Pankaja Raghav, Fakher Rahim, Vafa Rahimi-Movaghar,

Hazem Ramadan, Shakthi Kumaran Ramasamy, Rishabh Kumar Rana, Mohammad-Mahdi Rashidi, Devarajan Rathish, Salman Rawaf, Elrashdy Moustafa Mohamed Redwan, Luis Felipe Reyes, Victor Daniel Rosenthal, Allen Guy Ross, Nitai Roy, Kristina E Rudd, Tilleye Runghien, Cameron John Sabet, Basema Ahmad Saddik, Umar Saeed, Sahar Saeedi Moghaddam, Mohsen Safaei, Narjes Saheb Sharif-Askari, Amirhossein Sahebkar, Soumya Swaroop Sahoo, Morteza Saki, Nasir Salam, Zikria Saleem, Abdallah M Samy, Aswini Saravanan, Maheswar Satpathy, Mahan Shafie, Pritik A Shah, Samiah Shahid, Moyad Jamal Shahwan, Noga Shalev, Muhammad Aaqib Shamim, Anas Shamsi, Amin Sharifan, Rajesh P Shastry, Mahabalesh Shetty, Aminu Shittu, Emmanuel Edwar Siddig, Theologia Sideroglou, Jose Sifuentes-Osornio, Luís Manuel Lopes Rodrigues Silva, Eric A F Simões, Amit Singh, Surjit Singh, Robert Sinto, Amanda E Smith, Georgia Smith, Sameh S M Soliman, Chandan Kumar Swain, Lucien R Swetschinski, Lukasz Szarpak, Sree Sudha T Y, Shima Tabatabai, Celine Tabche, Zanan Mohammed-Ameen Taha, Ker-Kan Tan, Nathan Y Tat, Pugazhenthan Thangaraju, Marcos Roberto Tovani-Palone, Thang Huu Tran, Munkhtuya Tumurkhuu, Paul Turner, Aniefiok John Udoakang, Arit Udoh, Asokan Govindaraj Vaithinathan, Mario Valenti, Stein Emil Vollset, Kosala Gayan Weerakoon, Heiman F L Wertheim, Phoebe C M Williams, Eve E Wool, Teresa M Wozniak, Mukesh Kumar Kumar Yadav, Zwanden Sule Yahaya, Amir Yarahmadi, Saber Yezli, Hadiza Yusuf, Fathiah Zakham, Haijun Zhang, Zhi-Jiang Zhang, Magdalena Zielińska, Alimuddin Zumla, Sa'ed H H Zyoud, and Samer H Zyoud.

Managing the estimation or publications process

Nicole Davis Weaver, Christiane Dolecek, Simon I Hay, Kevin S Ikuta, Paulina A Lindstedt, Ali H Mokdad, Christopher J L Murray, Mohsen Naghavi, Amanda E Smith, Lucien R Swetschinski, Stein Emil Vollset, and Eve E Wool.
